# The burden of bacterial antimicrobial resistance in the WHO African region in 2019: a cross-country systematic analysis

**DOI:** 10.1016/S2214-109X(23)00539-9

**Published:** 2023-12-19

**Authors:** Antimicrobial Resistance Collaborators, Benn Sartorius, Authia P Gray, Nicole Davis Weaver, Gisela Robles Aguilar, Lucien R Swetschinski, Kevin S Ikuta, Tomislav Mestrovic, Erin Chung, Eve E Wool, Chieh Han, Anna Gershberg Hayoon, Daniel T Araki, Sherief Abd-Elsalam, Richard Gyan Aboagye, Lawan Hassan Adamu, Abiola Victor Adepoju, Ayman Ahmed, Gizachew Taddesse Akalu, Wuraola Akande-Sholabi, John H Amuasi, Ganiyu Adeniyi Amusa, Ayele Mamo Argaw, Raphael Taiwo Aruleba, Tewachew Awoke, Melese Kitu Ayalew, Ahmed Y Azzam, Francois-Xavier Babin, Indrajit Banerjee, Afisu Basiru, Nebiyou Simegnew Bayileyegn, Melaku Ashagrie Belete, James A Berkley, Julia A Bielicki, Denise Dekker, Dessalegn Demeke, Desalegn Getnet Demsie, Anteneh Mengist Dessie, Susanna J Dunachie, Abdelaziz Ed-Dra, Michael Ekholuenetale, Temitope Cyrus Ekundayo, Iman El Sayed, Muhammed Elhadi, Ibrahim Elsohaby, David Eyre, Adeniyi Francis Fagbamigbe, Nicholas A Feasey, Ginenus Fekadu, Frederick Fell, Karen M Forrest, Mesfin Gebrehiwot, Kebede Embaye Gezae, Ramy Mohamed Ghazy, Tewodros Tesfa Hailegiyorgis, Georgina Haines-Woodhouse, Ahmed I Hasaballah, Andrea Haekyung Haselbeck, Yingfen Hsia, Arnaud Iradukunda, Kenneth Chukwuemeka Iregbu, Chidozie C D Iwu, Chinwe Juliana Iwu-Jaja, Assefa N Iyasu, Fatoumatta Jaiteh, Hyonjin Jeon, Charity Ehimwenma Joshua, Gebrehiwot G Kassa, Patrick DMC Katoto, Ralf Krumkamp, Emmanuelle A P Kumaran, Hmwe Hmwe Kyu, Aseer Manilal, Florian Marks, Jürgen May, Susan A McLaughlin, Barney McManigal, Addisu Melese, Kebede Haile Misgina, Nouh Saad Mohamed, Mustapha Mohammed, Shafiu Mohammed, Shikur Mohammed, Ali H Mokdad, Catrin E Moore, Vincent Mougin, Neema Mturi, Temesgen Mulugeta, Fungai Musaigwa, Patrick Musicha, Lillian A Musila, Saravanan Muthupandian, Pirouz Naghavi, Hadush Negash, Dooshanveer C Nuckchady, Christina W Obiero, Ismail A Odetokun, Oluwaseun Adeolu Ogundijo, Lawrence Okidi, Osaretin Christabel Okonji, Andrew T Olagunju, Isaac Iyinoluwa Olufadewa, Gi Deok Pak, Olga Perovic, Andrew Pollard, Mathieu Raad, Clotaire Rafaï, Hazem Ramadan, Elrashdy Moustafa Mohamed Redwan, Anna Roca, Victor Daniel Rosenthal, Mohamed A Saleh, Abdallah M Samy, M Sharland, Aminu Shittu, Emmanuel Edwar Siddig, Eskinder Ayalew Sisay, Andy Stergachis, Wegen Beyene Tesfamariam, Caroline Tigoi, Marius Belmondo Tincho, Tenaw Yimer Tiruye, Chukwuma David Umeokonkwo, Timothy Walsh, Judd L Walson, Hadiza Yusuf, Naod Gebrekrstos Zeru, Simon I Hay, Christiane Dolecek, Christopher J L Murray, Mohsen Naghavi

## Abstract

**Background:**

A critical and persistent challenge to global health and modern health care is the threat of antimicrobial resistance (AMR). Previous studies have reported a disproportionate burden of AMR in low-income and middle-income countries, but there remains an urgent need for more in-depth analyses across Africa. This study presents one of the most comprehensive sets of regional and country-level estimates of bacterial AMR burden in the WHO African region to date.

**Methods:**

We estimated deaths and disability-adjusted life-years (DALYs) attributable to and associated with AMR for 23 bacterial pathogens and 88 pathogen–drug combinations for countries in the WHO African region in 2019. Our methodological approach consisted of five broad components: the number of deaths in which infection had a role, the proportion of infectious deaths attributable to a given infectious syndrome, the proportion of infectious syndrome deaths attributable to a given pathogen, the percentage of a given pathogen resistant to an antimicrobial drug of interest, and the excess risk of mortality (or duration of an infection) associated with this resistance. These components were then used to estimate the disease burden by using two counterfactual scenarios: deaths attributable to AMR (considering an alternative scenario where infections with resistant pathogens are replaced with susceptible ones) and deaths associated with AMR (considering an alternative scenario where drug-resistant infections would not occur at all). We obtained data from research hospitals, surveillance networks, and infection databases maintained by private laboratories and medical technology companies. We generated 95% uncertainty intervals (UIs) for final estimates as the 25th and 975th ordered values across 1000 posterior draws, and models were cross-validated for out-of-sample predictive validity.

**Findings:**

In the WHO African region in 2019, there were an estimated 1·05 million deaths (95% UI 829 000–1 316 000) associated with bacterial AMR and 250 000 deaths (192 000–325 000) attributable to bacterial AMR. The largest fatal AMR burden was attributed to lower respiratory and thorax infections (119 000 deaths [92 000–151 000], or 48% of all estimated bacterial pathogen AMR deaths), bloodstream infections (56 000 deaths [37 000–82 000], or 22%), intra-abdominal infections (26 000 deaths [17 000–39 000], or 10%), and tuberculosis (18 000 deaths [3850–39 000], or 7%). Seven leading pathogens were collectively responsible for 821 000 deaths (636 000–1 051 000) associated with resistance in this region, with four pathogens exceeding 100 000 deaths each: *Streptococcus pneumoniae, Klebsiella pneumoniae, Escherichia coli*, and *Staphylococcus aureus*. Third-generation cephalosporin-resistant *K pneumoniae* and meticillin-resistant *S aureus* were shown to be the leading pathogen–drug combinations in 25 and 16 countries, respectively (53% and 34% of the whole region, comprising 47 countries) for deaths attributable to AMR.

**Interpretation:**

This study reveals a high level of AMR burden for several bacterial pathogens and pathogen–drug combinations in the WHO African region. The high mortality rates associated with these pathogens demonstrate an urgent need to address the burden of AMR in Africa. These estimates also show that quality and access to health care and safe water and sanitation are correlated with AMR mortality, with a higher fatal burden found in lower resource settings. Our cross-country analyses within this region can help local governments to leverage domestic and global funding to create stewardship policies that target the leading pathogen–drug combinations.

**Funding:**

Bill & Melinda Gates Foundation, Wellcome Trust, and Department of Health and Social Care using UK aid funding managed by the Fleming Fund.

## Introduction

The rapid acceleration of antimicrobial resistance (AMR) is a major concern for the future of global health and modern health care. The first comprehensive global assessment of AMR burden estimated 4·95 million deaths associated with bacterial AMR and 1·27 million deaths attributable to bacterial AMR in 2019.^1^ The study found that while the threat of AMR is substantial across the globe, its burden is disproportionately high in low-income and middle-income countries (LMICs).^1^ Many LMICs, including those in sub-Saharan Africa, lack access to crucial, more effective antibiotics, which might contribute to increased AMR burden.^2^

Many LMICs, such as those in sub-Saharan Africa, face unique obstacles when implementing AMR surveillance programmes.^3^, ^4^ These surveillance obstacles range from lack of national action plans (NAPs), limited infrastructure and institutional capacity, reduced investment and human resources, underutilisation of available data, and inadequate dissemination of necessary information to regulatory bodies.^5^ Consequently, AMR burden in LMICs such as those in sub-Saharan Africa has largely remained undefined. AMR surveillance relies on data from local hospitals, small cohort studies in neonatal and adult wards, laboratory samples collected from patients with suspected infections, and documentation of hospital-acquired infections.^4^, ^6^, ^7^ There are major gaps in AMR data in Africa, including measurement of AMR burden in community and hospital settings, animals, and the environment; microbial acquisition of AMR; transmission patterns; genotypic evolution of AMR mechanisms; clonal spread; and asymptomatic carriage.^4^ Furthermore, inadequate AMR preparedness, evidenced by few implemented NAPs, suggests that AMR has been a low priority for most sub-Saharan Africa countries,^4^ and more concerted efforts are urgently needed.

**Research in context**

**Evidence before this study**

Antimicrobial resistance (AMR) is widely considered to be a serious threat to human health. In 2016, the *Review on Antimicrobial Resistance* published a study reporting that 10 million deaths could occur annually from AMR by 2050. In 2022, the Global Burden of Diseases, Injuries, and Risk Factors Study (GBD) published the results of their global burden of AMR study, which found that nearly 5 million deaths had occurred in 2019 where a resistant bacterial infection was present. This work corroborated the findings of the 2016 report, identifying AMR as a leading cause of death globally and highlighting a need for more granular, country-level estimates of AMR mortality. The GBD's first regional analysis of the burden of AMR in the WHO European region found that more than 500 000 deaths were linked to resistant bacteria throughout Europe in 2019. There are currently very few studies in the WHO African region estimating the attributable and associated burden of bacterial AMR, based on an in-depth search of the articles available in PubMed, MEDLINE, Web of Science, and Scopus covering exposure to resistant bacterial agents. Therefore, country-level estimates generated from comprehensive data and a rigorous analytic framework are urgently needed to provide a timely pan-African overview of bacterial AMR burden. Our literature reviews were conducted between Nov 1, 2019, and Sept 30, 2020. Additional details, including the search terms used, are available in the appendix (pp 5–8).

**Added value of this study**

This study is the most comprehensive analysis of the burden of bacterial AMR in the WHO African region to date, providing estimates for 47 countries, 23 bacterial pathogens, and 88 pathogen–drug combinations in 2019. The burden of AMR in sub-Saharan Africa has largely remained undefined, and this study presents an extensive set of estimates for bacterial AMR burden from priority pathogen–drug combinations in this region. We use the major methodological innovations from the global burden of bacterial AMR study to describe the magnitude of the problem within the WHO African region by estimating two different counterfactual scenarios: deaths attributable to AMR (considering an alternative scenario where infections with resistant pathogens are replaced with susceptible ones) and deaths associated with AMR (considering an alternative scenario where drug-resistant infections would not occur at all). Our approach builds on estimates of disease incidence, prevalence, and mortality generated by GBD 2019, which allows for the comparability of AMR with other leading causes of death. The current study focuses only on bacterial AMR burden. In future studies, we plan to expand to include priority non-bacterial pathogen-related AMR burden.

**Implications of all the available evidence**

The results of this study show that bacterial AMR is an urgent health threat in the WHO African region, with notable and important differences between countries. Rigorous estimates of AMR morbidity and mortality are essential to inform policy and public health investments that are tailored to the needs of each country within this region. This study provides insight into the pathogens and pathogen–drug combinations that are responsible for the highest estimated burden throughout Africa, which can be vital in guiding mitigation efforts to address AMR. Such efforts should include expanding antimicrobial stewardship and infection prevention and control programmes as well as furthering antibiotic and vaccine development research.

This study presents, to our knowledge, the first regional and country-level AMR estimates for the WHO African region in 2019, covering an extensive set of bacterial pathogens and bacterium–drug combinations with the use of consistent methods for two counterfactual scenarios. This information will help raise the profile of AMR in this region by highlighting countries with the highest estimated burden where effective NAPs need to be implemented. This manuscript was produced as part of the Global Burden of Diseases, Injuries, and Risk Factors Study (GBD) Collaborator Network and in accordance with the GBD Protocol.^8^

## Methods

### Overview and input data

This paper is part of a collection of studies that aim to describe the regional burden of bacterial AMR. The findings presented here extend the results of our original study while using the same methodology. Therefore, parts of this Methods text are taken directly from our previously published study.^9^ This study is a major extension of GBD, which the University of Washington Institutional Review Board has approved under IRB ID 9060.

This study extends the results of the global burden of AMR study^1^ by providing more granular and country-specific estimates within the WHO African region. More specifically, based on this estimation of all-age and age-specific deaths and disability-adjusted life-years (DALYs) for 204 countries and territories, we present aggregated estimates for the WHO African region in 2019 and its countries. Disease burden associated with and attributable to AMR was estimated for 12 major infectious syndromes and one residual category, 23 bacterial pathogens, and 88 pathogen–drug combinations (included data sources indicated in appendix pp 4–5, 8–16, 19, 24; infectious syndromes listed in appendix p 11; and pathogen–drug combinations provided in appendix pp 19–20).

The global input data for the estimation process consisted of 343 million individual records or isolates covering 11 361 study-location-years obtained from surveillance systems, hospital systems, systematic literature reviews, and other sources (appendix p 88). All data inputs for the models used in this study were empirical data, except for a custom vaccine probe data meta-analysis which we used to estimate the fraction of pneumonia caused by *Streptococcus pneumoniae*. We used clinical breakpoints and methods from the Clinical and Laboratory Standards Institute (CLSI) as guidance for classification of isolates into categories of susceptible or resistant.^10^

Our overall approach can be divided into five broad components: the number of deaths involving infection, the proportion of infectious deaths attributable to a given infectious syndrome, the proportion of infectious syndrome deaths attributable to a given pathogen, the percentage of a given pathogen resistant to an antimicrobial drug of interest, and the excess risk of death or duration of an infection associated with this resistance.

In addition, we used the Socio-demographic Index (SDI);^11^ access to safe water, sanitation, and hygiene (WaSH); antibiotic consumption; and AMR NAP status (based on an internal review of national documentation) in the correlation analysis against the AMR burden estimates (appendix p 29).

### Estimation steps

In our approach, ten broad estimation steps took place within the five broad modelling components (appendix pp 9–29). In estimation steps one and two, we defined the number of deaths where infection plays a role by using the GBD 2019 cause of death estimates^12^ to determine the number of deaths by age, sex, and location for which either the underlying cause of death was of infectious origin, or for which the pathway to death was via sepsis. We then attributed these deaths to their respective infectious syndromes. In estimation steps three and four, we estimated the pathogen distribution for each infectious syndrome for deaths and incident cases for each age, sex, and location. In estimation steps five, six, and seven, we estimated the prevalence of phenotypic resistance by country for each of the 88 pathogen–drug combinations. In estimations steps eight and nine, we estimated the relative risk of death for each pathogen–drug combination for a resistant infection compared with that of a drug-sensitive infection. The availability of input data for the above estimation steps is documented in the appendix (p 88). Data points for sub-Saharan Africa included in each primary modelling step are shown in table 1.

**Table 1:** Data points (cases or deaths) included in each primary modelling step by GBD region, and the fraction of countries represented in each GBD region

	**(1) Sepsis and infectious syndrome models**	**Fraction of countries represented in (1)**	**(2) Case fatality rate**	**Fraction of countries represented in (2)**	**(3) Pathogen distribution**	**Fraction of countries represented in (3)**	**(4) Fraction of resistance**	**Fraction of countries represented in (4)**	**(5) Relative risk**	**Fraction of countries represented in (5)**
Central sub-Saharan Africa	NA	0/6	NA	0/6	29 853	5/6	40 291	6/6	NA	0/6
Eastern sub-Saharan Africa	482	2/15	223	4/15	191 291	12/15	519 737	14/15	3436	2/15
Southern sub-Saharan Africa	4 696 889	1/6	44	1/6	1 130 418	6/6	1 003 898	6/6	22 178	1/6
Western sub-Saharan Africa	100	1/19	17 770	9/19	302 143	15/19	367 790	18/19	14 880	2/19

Data points are sourced from a variety of sources including, but not limited to, multiple cause of death data, hospital discharges, literature studies, and microbiology data with and without outcome. Several data sources inform multiple modelling steps. Therefore, data points should not be summed across a row as that will lead to duplication. For more information on the data types used and the modelling steps that they inform, see section 2 of the appendix (pp 4–8). GBD=Global Burden of Diseases, Injuries, and Risk Factors Study. NA=not applicable (zero data sources).

Finally, in estimation step ten, we computed two plausible counterfactual scenarios to answer the question of what would happen if we eliminated all resistant infections, estimating the drug-resistant burden as deaths and DALYs directly attributable to bacterial AMR based on the counterfactual of drug-sensitive infection, and deaths and DALYs associated with bacterial AMR based on the counterfactual of no infection (appendix pp 26–29).

### AMR burden calculation approach

In a scenario in which drug-resistant infections are replaced with drug-susceptible ones, we considered the excess risk of resistance, known as the attributable to AMR counterfactual scenario. Deaths attributable to AMR were calculated by multiplying the number of deaths for each underlying cause by the fraction of these deaths in which infection was implicated, followed by multiplying the fraction of infectious deaths attributable to each infectious syndrome. This was then multiplied by the fraction of infectious syndrome deaths attributable to each pathogen and by a mutually exclusive risk-weighted estimate of burden attributable to resistance that takes into account patterns of co-resistance among different antibiotics for each location-year and pathogen–drug combination (risk-weighted estimate described in appendix pp 22–23, 27–28).

Under the no-infection counterfactual scenario, infections that are resistant would not occur; this is the associated with AMR scenario. Calculations here closely follow the process described for the attributable to AMR counterfactual, except the risk-weighted estimate was replaced with the prevalence of resistance for each location-year and pathogen–drug combination. We used a similar approach to calculate DALYs for both counterfactual scenarios (appendix pp 26–29).

### Modelling tools and framework

Details on our modelling approach can be found in the global burden of AMR study^1^ and in the appendix (pp 9–29). Briefly, for estimation steps three and four we used the Bayesian meta-regression tool MR-BRT to estimate case-fatality rates as a function of the Healthcare Access and Quality Index and various bias covariates. We used multinomial estimation with partial and composite observations to incorporate heterogeneous data in the estimation of pathogen distributions for each infectious syndrome. In estimation steps five to seven, we used a two-stage spatiotemporal modelling framework to estimate the prevalence of resistance in each pathogen–drug combination.

Given the relationship between antibiotic consumption levels and AMR rates,^13^ we modelled antibiotic consumption at the national level to use as a covariate in the stacked ensemble model of prevalence of resistance, with the addition of an ensemble spatiotemporal Gaussian process regression model to combine antibiotic usage estimates with pharmaceutical sales data for LMICs.^14^ In cross-country comparisons, the indicator metric “defined daily doses (DDD) per 1000 inhabitants per day” was used to report antibiotic consumption in the community and within the hospital setting (in accordance with the WHO Anatomical Therapeutic Chemical classification) by providing a rough estimate of the proportion of the population treated with antimicrobials on a daily basis. We used MR-BRT and a two-stage nested mixed effects meta-regression model in the estimation of both relative risk of death and excess risk of hospital stay for each pathogen–drug combination (appendix pp 13–26). The software used for these analyses is described in the appendix (p 22).

### Uncertainty analysis

Consistent with previously described GBD methods,^12^ we propagated uncertainty from each step of the analysis into the final estimates of deaths and infections attributable to and associated with drug resistance by taking the 25th and 975th of 1000 draws from the posterior distribution of each quantity of interest. The models were cross-validated for out-of-sample predictive validity.

This study complies with the Guidelines for Accurate and Transparent Health Estimates Reporting (GATHER).^15^ The full GATHER checklist is provided in the appendix (pp 101–103).

### Role of the funding source

The funders of the study had no role in study design, data collection, data analysis, data interpretation, or writing of the report.

## Results

We estimated 3·83 million (95% uncertainty interval [UI] 3·17–4·67) deaths in 2019 involving infection in the WHO African region. 1·86 million (1·53–2·27) of those deaths were caused by both susceptible and resistant bacteria. Of these, 1·05 million deaths (829 000–1 316 000) were associated with AMR and 250 000 deaths (192 000–325 000) were attributable to AMR (table 2). The WHO African region has the largest fatal and non-fatal burden of AMR compared with any other WHO region (appendix p 89). Infection-related mortality rates are highest in the WHO African region (348·3 [288·2–425·1] per 100 000), while the fraction of deaths associated with resistance (27% [25–29]) and attributable to resistance (7% [6–8]) are the lowest. Despite the relatively low prevalence of resistance in the region, the sheer number of infections yields high AMR mortality.

**Table 2:** The overall AMR burden by infectious syndrome in the WHO African region in 2019

	**Deaths that involved infection (counts)**	**Deaths caused by bacteria (counts)**	**Deaths associated with AMR (counts)**	**Deaths associated with AMR (all-age rate per 100 000)**	**DALYs associated with AMR (counts)**	**DALYs associated with AMR (all-age rate per 100 000)**	**Deaths attributable to AMR (counts)**	**Deaths attributable to AMR (all-age rate per 100 000)**	**DALYs attributable to AMR (counts)**	**DALYs attributable to AMR (all-age rate per 100 000)**
All	3 830 000 (3 169 000–4 674 000)	1 859 000 (1 530 000–2 267 000)	1 046 000 (829 000–1 316 000)	95·2 (75·4–119·7)	64 344 000 (50 451 000–81 427 000)	5851·8 (4588·4–7405·4)	250 000 (192 000–325 000)	22·7 (17·5–29·5)	15 031 000 (11 392 000–19 454 000)	1367 (1036·1–1769·2)
Bloodstream infections	557 000 (382 000–800 000)	338 000 (228 000–489 000)	236 000 (159 000–342 000)	21·5 (14·5–31·1)	15 512 000 (10 530 000–21 970 000)	1410·7 (957·6–1998·1)	56 000 (37 000–82 000)	5·1 (3·3–7·4)	3 620 000 (2 414 000–5 294 000)	329·2 (219·5–481·5)
Bone and joint infections	3950 (1240–9020)	3520 (1080–8070)	2180 (672–4990)	0·2 (0·1–0·5)	78 000 (22 000–188 000)	7·1 (2·0–17·1)	493 (152–1160)	0·0 (0·0–0·1)	18 000 (4 910–45 000)	1·6 (0·4–4·1)
Cardiac infections	5810 (4300–7760)	5100 (3760–6790)	3610 (2660–4810)	0·3 (0·2–0·4)	153 000 (114 000–208 000)	13·9 (10·4–18·9)	850 (613–1140)	0·1 (0·1–0·1)	35 000 (25 000–48 000)	3·2 (2·3–4·4)
Gonorrhoea and chlamydia	684 (510–852)	NA	NA	NA	11 000 (6180–17 000)	1·0 (0·6–1·6)	NA	NA	1060 (282–2150)	0·1 (0·0–0·2)
CNS infections	135 000 (106 000–173 000)	82 000 (64 000–107 000)	65 000 (51 000–86 000)	5·9 (4·6–7·8)	4 665 000 (3 523 000–6 277 000)	424·3 (320·4–570·9)	15 000 (11 000–21 000)	1·4 (1–1·9)	1 079 000 (770 000–1 508 000)	98·2 (70–137·2)
Diarrhoea	589 000 (444 000–776 000)	115 000 (61 000–186 000)	27 000 (16 000–41 000)	2·4 (1·4–3·7)	2 086 000 (1 211 000–3 273 000)	189·7 (110·1–297·6)	6280 (3270–10 000)	0·6 (0·3–1)	489 000 (256 000–821 000)	44·5 (23·2–74·7)
Other infections	863 000 (620 000–1 206 000)	NA	NA	NA	NA	NA	NA	NA	NA	NA
Intra-abdominal infections	159 000 (102 000–225 000)	129 000 (85 000–182 000)	106 000 (69 000–150 000)	9·6 (6·3–13·7)	3 556 000 (2 190 000–5 255 000)	323·4 (199·2–477·9)	26 000 (17 000–39 000)	2·4 (1·5–3·5)	872 000 (521 000–1 347 000)	79·3 (47·4–122·5)
Lower respiratory and thorax infections	1 000 000 (827 000–1 209 000)	677 000 (552 000–831 000)	521 000 (423 000–642 000)	47·4 (38·5–58·4)	34 412 000 (27 074 000–43 494 000)	3129·7 (2462·2–3955·6)	119 000 (92 000–151 000)	10·8 (8·4–13·8)	7 786 000 (5 819 000–10 087 000)	708·1 (529·3–917·4)
Bacterial skin infections	44 000 (17 000–86 000)	37 000 (14 000–75 000)	18 000 (6880–36 000)	1·6 (0·6–3·3)	580 000 (256 000–1 206 000)	52·7 (23·3–109·7)	3720 (1400–7660)	0·3 (0·1–0·7)	120 000 (49 000–258 000)	10·9 (4·5–23·5)
Tuberculosis	369 000 (319 000–429 000)	369 000 (319 000–429 000)	42 000 (27 000–64 000)	3·8 (2·5–5·8)	1 847 000 (1 193 000–2 800 000)	168 (108·5–254·6)	18 000 (3850–39 000)	1·6 (0·3–3·6)	748 000 (163 000–1 604 000)	68 (14·8–145·8)
Typhoid, paratyphoid, and iNTS	89 000 (55 000–132 000)	89 000 (55 000–132 000)	14 000 (6680–25 000)	1·3 (0·6–2·3)	1 067 000 (507 000–1 959 000)	97·1 (46·1–178·1)	2270 (488–5250)	0·2 (0·0–0·5)	173 000 (38 000–401 000)	15·7 (3·4–36·5)
Urinary tract infections	16 000 (9770–23 000)	14 000 (8680–21 000)	12 000 (7160–17 000)	1·1 (0·7–1·5)	376 000 (245 000–534 000)	34·2 (22·3–48·5)	2790 (1700–4020)	0·3 (0·2–0·4)	89 000 (59 000–125 000)	8·1 (5·3–11·4)

Data are estimates (95% uncertainty interval). Estimates were aggregated across drugs, accounting for the co-occurrence of resistance to multiple drugs. For gonorrhoea and chlamydia, we did not estimate the fatal burden, thus only the DALY burden is presented. AMR=antimicrobial resistance. DALY=disability-adjusted life-year. iNTS=invasive non-typhoidal salmonellae. NA=not applicable.

Of the 3·83 million infection-related deaths, 1·93 million (95% UI 1·57–2·37) involved one of three syndromes as an underlying or an intermediate cause of death: lower respiratory and thorax infection, bloodstream infection, or tuberculosis infection (table 2). Lower respiratory and thorax infections were responsible for the greatest fatal burden (677 000 deaths [552 000–831 000], or 36% of all estimated bacterial deaths), followed by tuberculosis (369 000 deaths [319 000–429 000], or 20%), and bloodstream infections (338 000 deaths [228 000–489 000], or 18%). Collectively, these three infectious syndromes accounted for approximately 75% of the fatal burden from bacterial infections in the WHO African region.

Among the top-ranking infectious syndromes, the proportion of associated and attributable AMR deaths was estimated as follows: among 677 000 deaths involving lower respiratory and thorax infections, a total of 521 000 deaths (95% UI 423 000–642 000, or 77%) were associated with and 119 000 deaths (92 000–151 000, or 18%) were attributable to any resistant pathogen–drug combination. Likewise, for the 369 000 deaths involving tuberculosis, 11% (42 000 deaths [27 000–64 000]) were associated with and 5% (18 000 deaths [3850–39 000]) were attributable to any resistant pathogen–drug combination. Among 338 000 deaths involving bloodstream infections, 236 000 deaths (159 000–342 000) were associated with and 56 000 deaths (37 000–82 000) were attributable to any resistant pathogen–drug combination. Among 129 000 deaths involving intra-abdominal infections, 106 000 deaths (69 000–150 000) were associated with and 26 000 deaths (17 000–39 000) were attributable to any resistant pathogen–drug combination. Table 2 shows the complete breakdown per infectious syndrome. This is further decomposed by country in the appendix (pp 84–87), which shows considerable heterogeneity in the fraction of deaths due to infection across the region, ranging from 14% in Algeria to 64% in Chad. Additionally, we estimated that 48% of infection-related deaths were associated with resistance in Algeria (highest in the region), while Chad was estimated at 25%, and the lowest fractions of infection-related deaths associated with resistance were in Lesotho (17%) and South Africa (17%).

**Figure 1:**
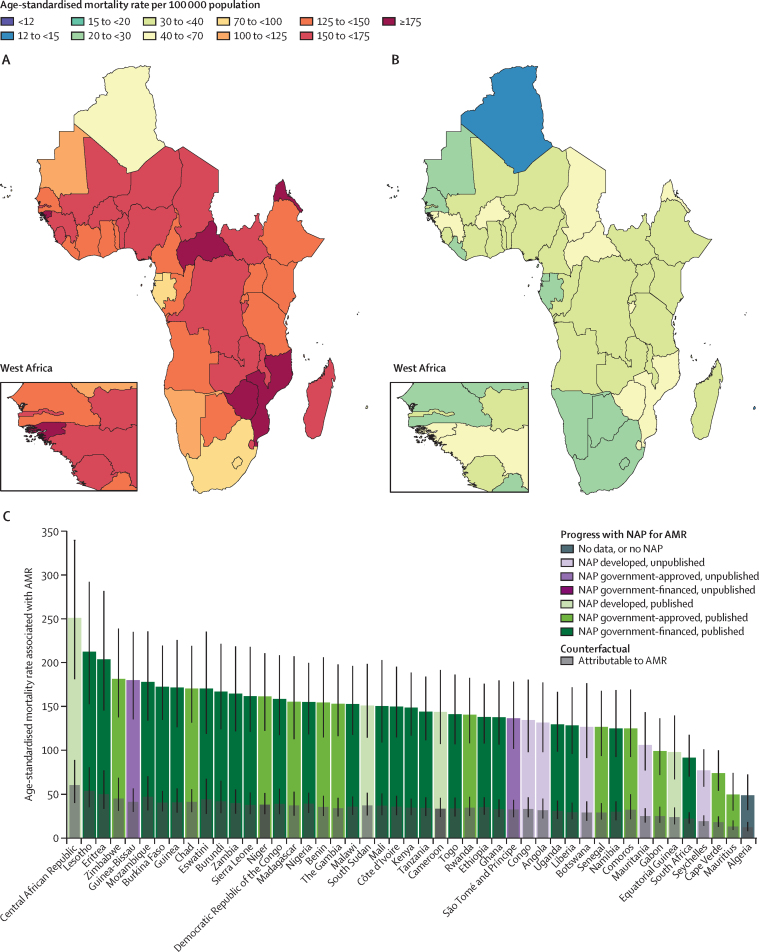
Age-standardised mortality associated with and attributable to AMR in Africa in 2019 (A) Age-standardised mortality associated with AMR. (B) Age-standardised mortality attributable to AMR. (C) Age-standardised mortality associated with and attributable to AMR in relation to the status of NAPs for the countries in the WHO African region. Data from Angola on AMR NAPs were last reported in the 2018−19 survey responses. Algeria last reported on the 2021−22 survey responses. AMR=antimicrobial resistance. NAP=national action plan.

Figure 1 shows the overall age-standardised mortality rates per 100 000 population associated with and attributable to AMR by country in the WHO African region in 2019. The highest rates were estimated in Central African Republic, which had an estimated 251·3 deaths (95% UI 181·2–339·8) associated with and 60·2 deaths (40·2–88·8) attributable to AMR per 100 000 (figure 1A, B). Two other countries also exceeded 200 associated deaths per 100 000 in 2019: Lesotho (212·5 [152·8–292·4]) and Eritrea (203·8 [145·5–281·8]). Both Lesotho and Eritrea have an NAP that is approved and currently being implemented. Guinea-Bissau is another country with a relatively high estimated rate of associated (180·2 [135·5–235·2]) and attributable (41·5 [29·9–56·5]) mortality that also currently has an NAP. A full breakdown of all pathogen and antibiotic AMR-associated and attributable death counts and age-standardised rates by country in 2019 are in the appendix (pp 52–71).

Four pathogens were each responsible for more than 100 000 deaths associated with AMR in the WHO African region in 2019: *Streptococcus pneumoniae* (195 000 deaths [95% UI 159 000–239 000]), *Klebsiella pneumoniae* (184 000 deaths [142 000–236 000]), *Escherichia coli* (147 000 deaths [112 000–189 000]), and *Staphylococcus aureus* (136 000 deaths [109 000–172 000]; table 3). These four pathogens were each estimated to be directly attributable to more than 25 000 deaths in 2019: *K pneumoniae* (50 000 deaths [33 000–71 000]), followed by *S pneumoniae* (39 000 deaths [25 000–54 000]), *E coli* (37 000 deaths [27 000–50 000]), and *S aureus* (30 000 deaths [19 000–43 000]). Two other pathogens were responsible for more than 40 000 deaths associated with AMR in 2019: *Acinetobacter baumannii* (48 000 deaths [31 000–71 000]) and *Mycobacterium tuberculosis* (42 000 deaths [27 000–64 000]). In total, these six pathogens were responsible for 752 000 deaths associated with (or 72% of all associated deaths) and 188 000 deaths attributable to (or 75% of all attributable deaths) AMR in the region in 2019.

**Table 3:** Summary of deaths and DALYs by pathogen for the WHO African region 2019

	**Associated with AMR**				**Attributable to AMR**			
	Deaths		DALYs		Deaths		DALYs	
	Counts	Rate per 100 000	Counts	Rate per 100 000	Counts	Rate per 100 000	Counts	Rate per100 000
All	1 046 000 (829 000–1 316 000)	95·2 (75·4–119·7)	64 344 000 (50 451 000–81 427 000)	5851·8 (4588·4–7405·4)	250 000 (192 000–325 000)	22·7 (17·5–29·5)	15 031 000 (11 392 000–19 454 000)	1367·0 (1036·1–1769·2)
*Acinetobacter baumannii*	48 000 (31 000–71 000)	4·4 (2·8–6·5)	2 093 000 (1 396 000–3 005 000)	190·4 (127·0–273·3)	14 000 (9000–22 000)	1·3 (0·8–2·0)	620 000 (393 000–940 000)	56·4 (35·7–85·5)
*Citrobacter* spp	6000 (4000–9000)	0·6 (0·4–0·9)	381 000 (229 000–588 000)	34·6 (20·8–53·5)	2000 (1000–3000)	0·2 (0·1–0·3)	113 000 (61 000–187 000)	10·3 (5·6–17·0)
*Enterobacter* spp	35 000 (25 000–48 000)	3·2 (2·3–4·4)	2 042 000 (1 471 000–2 812 000)	185·7 (133·8–255·8)	8000 (6000–12 000)	0·8 (0·5–1·1)	484 000 (327 000–681 000)	44·0 (29·7–61·9)
*Enterococcus faecalis*	18 000 (11 000–25 000)	1·6 (1·0–2·3)	937 000 (625 000–1 332 000)	85·2 (56·9–121·1)	5000 (2000–8000)	0·4 (0·2–0·7)	247 000 (126 000–398 000)	22·4 (11·5–36·2)
*Enterococcus faecium*	20 000 (12 000–31 000)	1·8 (1·1–2·8)	840 000 (528 000–1 297 000)	76·4 (48·0–118·0)	5000 (2000–9000)	0·4 (0·2–0·8)	211 000 (106 000–377 000)	19·2 (9·6–34·3)
Other enterococci	9000 (6000–13 000)	0·8 (0·5–1·2)	491 000 (313 000–752 000)	44·6 (28·5–68·4)	2000 (444–4000)	0·2 (0·0–0·3)	101 000 (23 000–196 000)	9·2 (2·1–17·8)
*Escherichia coli*	147 000 (112 000–189 000)	13·4 (10·2–17·2)	8 761 000 (6 609 000–11 523 000)	796·8 (601·0–1047·9)	37 000 (27 000–50 000)	3·4 (2·4–4·6)	2 212 000 (1 560 000–3 036 000)	201·1 (141·9–276·1)
Group A *Streptococcus*	4000 (3000–7000)	0·4 (0·2–0·7)	238 000 (155 000–360 000)	21·6 (14·1–32·8)	412 (0–1000)	0·0 (0·0–0·1)	23 000 (0–75 000)	2·0 (0·0–6·8)
Group B *Streptococcus*	55 000 (41 000–72 000)	5·0 (3·8–6·6)	4 063 000 (2 996 000–5 458 000)	369·5 (272·4–496·4)	9000 (2000–17 000)	0·8 (0·2–1·6)	682 000 (184 000–1 296 000)	62·0 (16·7–117·9)
*Haemophilus influenzae*	13 000 (10 000–17 000)	1·2 (0·9–1·5)	984 000 (738 000–1 285 000)	89·5 (67·1–116·9)	3000 (1000–5000)	0·3 (0·1–0·4)	217 000 (90 000–358 000)	19·7 (8·2–32·6)
*Klebsiella pneumoniae*	184 000 (142 000–236 000)	16·7 (12·9–21·5)	11 600 000 (8 919 000–14 972 000)	1054·9 (811·2–1361·7)	50 000 (33 000–71 000)	4·6 (3·0–6·5)	3 173 000 (2 112 000–4 540 000)	288·6 (192·1–412·9)
*Morganella* spp	185 (100–295)	0·0 (0·0–0·0)	5000 (3000–8000)	0·5 (0·3–0·7)	43 (19–76)	0·0 (0·0–0·0)	1000 (566–2000)	0·1 (0·1–0·2)
*Mycobacterium tuberculosis*	42 000 (27 000–64 000)	3·8 (2·5–5·8)	1 847 000 (1 193 000–2 800 000)	168·0 (108·5–254·6)	18 000 (3850–39 000)	1·6 (0·3–3·6)	748 000 (163 000–1 604 000)	68·0 (14·8–145·8)
*Neisseria gonorrhoeae*	NA	NA	11 000 (6180–17 000)	1·0 (0·6–1·6)	NA	NA	1060 (282–2150)	0·1 (0·0–0·2)
Non-typhoidal *Salmonellae*	1000 (687–2000)	0·1 (0·1–0·2)	100 000 (51 000–177 000)	9·1 (4·7–16·1)	272 (39–647)	0·0 (0·0–0·1)	20 000 (3000–48 000)	1·8 (0·3–4·4)
*Proteus* spp	11 000 (7000–16 000)	1·0 (0·6–1·5)	410 000 (265 000–612 000)	37·3 (24·1–55·7)	1000 (788–2000)	0·1 (0·1–0·2)	56 000 (31 000–92 000)	5·1 (2·8–8·4)
*Pseudomonas aeruginosa*	56 000 (42 000–72 000)	5·1 (3·9–6·6)	3 313 000 (2 534 000–4 304 000)	301·3 (230·5–391·4)	14 000 (9000–21 000)	1·3 (0·8–1·9)	824 000 (541 000–1 227 000)	74·9 (49·2–111·6)
*Salmonella enteritica* serotype Paratyphi	89 (28–216)	0·0 (0·0–0·0)	7000 (2000–17 000)	0·6 (0·2–1·5)	17 (2–49)	0·0 (0·0–0·0)	1000 (224–4000)	0·1 (0·0–0·4)
*Salmonella enteritica* serotype Typhi	42 000 (29 000–60 000)	3·9 (2·6–5·5)	3 294 000 (2 227 000–4 752 000)	299·6 (202·6–432·2)	7000 (1000–13 000)	0·6 (0·1–1·2)	534 000 (105 000–1 035 000)	48·5 (9·6–94·1)
*Serratia* spp	14 000 (9000–21 000)	1·3 (0·8–2·0)	907 000 (592 000–1 354 000)	82·5 (53·8–123·1)	3000 (2000–6000)	0·3 (0·2–0·5)	223 000 (124 000–363 000)	20·3 (11·3–33·0)
*Shigella* spp	10 000 (4000–19 000)	0·9 (0·4–1·8)	799 000 (332 000–1 489 000)	72·6 (30·2–135·4)	2000 (278–5000)	0·2 (0·0–0·5)	165 000 (21 000–396 000)	15·0 (1·9–36·0)
*Staphylococcus aureus*	136 000 (109 000–172 000)	12·3 (9·9–15·7)	7 582 000 (5 888 000–9 678 000)	689·5 (535·4–880·2)	30 000 (19 000–43 000)	2·7 (1·7–3·9)	1 663 000 (1 061 000–2 436 000)	151·2 (96·5–221·6)
*Streptococcus pneumoniae*	195 000 (159 000–239 000)	17·7 (14·5–21·7)	13 640 000 (10 804 000–17 051 000)	1240·5 (982·6–1550·7)	39 000 (25 000–54 000)	3·5 (2·3–4·9)	2 713 000 (1 710 000–3 846 000)	246·7 (155·5–349·8)

Data are estimates (95% uncertainty interval). AMR=antimicrobial resistance. DALY=disability-adjusted life-year. NA=not applicable.

**Figure 2:**
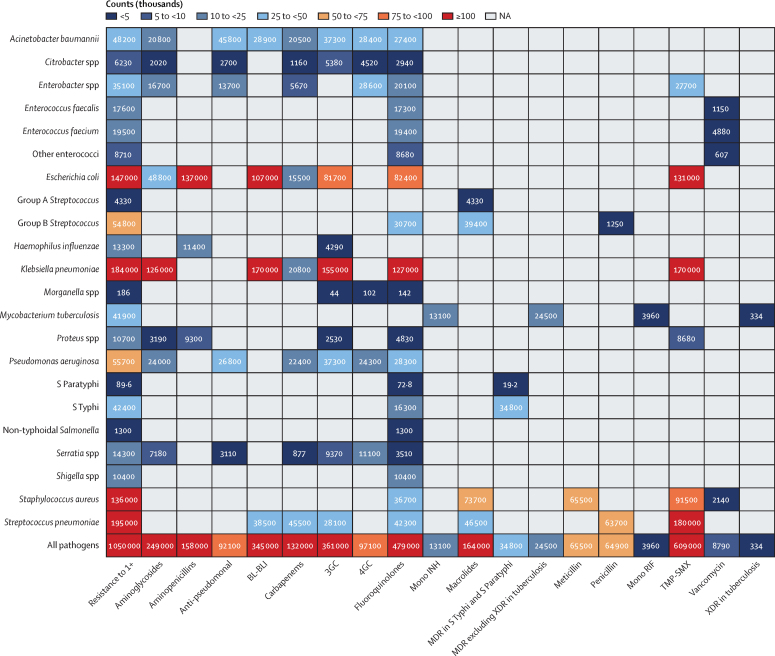
Deaths (count) associated with bacterial antimicrobial resistance by pathogen−drug combinations, 2019 3GC=third-generation cephalosporins. 4GC=fourth-generation cephalosporins. Anti-pseudomonal=anti-pseudomonal penicillin or β-lactamase inhibitors. BL-BLI=β-lactam or β-lactamase inhibitors. Group A *Streptococcus=Streptococcus pyogenes*. Group B *Streptococcus=Streptococcus agalactiae*. MDR=multidrug resistance. Mono INH=isoniazid mono-resistance. Mono RIF=rifampicin mono-resistance. NA=not applicable. Penicillin=natural pencillins susceptible to penicillinases (benzylpenicillin, phenoxymethylpenicillin, procaine benzylpenicillin, and benzathine benzylpenicillin). Resistance to 1+=resistance to one or more drugs. S Paratyphi=*Salmonella enterica* serotype Paratyphi. S Typhi=*Salmonella enterica* serotype Typhi. TMP-SMX=trimethoprim-sulfamethoxazole. XDR=extensive drug resistance.

**Figure 3:**
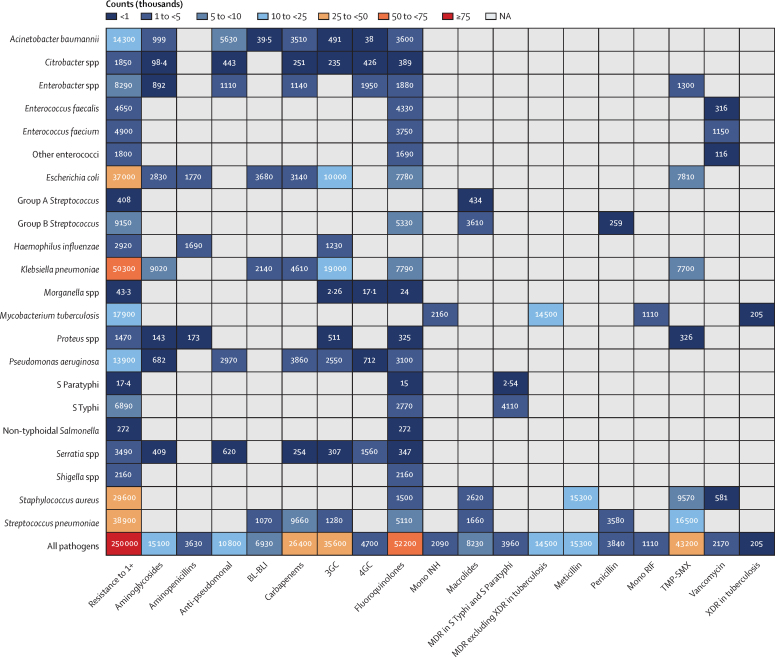
Deaths (count) attributable to bacterial antimicrobial resistance by pathogen−drug combinations, 2019 3GC=third-generation cephalosporins. 4GC=fourth-generation cephalosporins. Anti-pseudomonal=anti-pseudomonal penicillin or β-lactamase inhibitors. BL-BLI=β-lactam or β-lactamase inhibitors. Group A *Streptococcus=Streptococcus pyogenes*. Group B *Streptococcus=Streptococcus agalactiae*. MDR=multidrug resistance. Mono INH=isoniazid mono-resistance. Mono RIF=rifampicin mono-resistance. NA=not applicable. Penicillin=natural pencillins susceptible to penicillinases (benzylpenicillin, phenoxymethylpenicillin, procaine benzylpenicillin, and benzathine benzylpenicillin). Resistance to 1+=resistance to one or more drugs. S Paratyphi=*Salmonella enterica* serotype Paratyphi. S Typhi=*Salmonella enterica* serotype Typhi. TMP-SMX=trimethoprim-sulfamethoxazole. XDR=extensive drug resistance.

Nine pathogen–drug combinations were each associated with more than 100 000 AMR deaths in the WHO African region in 2019. *S pneumoniae* resistant to trimethoprim–sulfamethoxazole (TMP-SMX) was the combination with the largest number of associated AMR deaths (180 000 [95% UI 147 000–220 000]), followed by *K pneumoniae* resistant to β-lactam or β-lactamase inhibitors (170 000 [131 000–218 000]; figure 2). The leading three combinations responsible for the largest attributable AMR deaths were *K pneumoniae* resistant to third-generation cephalosporins (19 000 [95% UI 7320–34 000]), *S pneumoniae* resistant to TMP-SMX (16 500 [2230–33 000]), and meticillin-resistant *S aureus* (MRSA; 15 300 [6550–26 000]; figure 3).

Two pathogen–antibiotic combinations, namely *K pneumoniae* resistant to third-generation cephalosporins and MRSA, predominated as the leading pathogen–drug combinations in 25 (53%) and 11 (23%) countries, respectively, of the 47 countries in the WHO African region, for AMR-attributable deaths (appendix pp 52–62). TMP-SMX-resistant *K pneumoniae* was shown to be the leading combination in 16 (34%) of the 47 countries for AMR-associated death rates, followed by TMP-SMX-resistant *S pneumoniae* in 12 countries (26%), and β-lactam or β-lactamase inhibitor resistant *K pneumoniae* in ten (21%) of 47 countries in the region (appendix pp 63–71). Within TMP-SMX resistance, *S pneumoniae* was the highest ranked pathogen in terms of attributable mortality in 42 (89%) of 47 countries in the region. There is a significant correlation between the number of deaths directly attributed to TMP-SMX-resistant *S pneumoniae* in 2019 and the number of deaths due to HIV/AIDS in 2019 (*r*=0·77 [95% CI 0·62 to 0·87]; appendix pp 72–80).

A detailed breakdown of age-standardised mortality rates by age in each country of the region for males and females is available in the appendix (p 100). For most countries, the number of deaths related to AMR—either attributable or associated—was greater among neonates (those aged 28 days and younger) than in older age groups. This was especially true in central and western Africa, particularly in Benin, Burkina Faso, Mali, Côte d'Ivoire, Sierra Leone, Chad, and Central African Republic. There were a few exceptions to this pattern, with lower neonatal mortality rates relative to some of the older age groups in Seychelles, Mauritius, Cabo Verde, and Algeria.

Neonatal AMR mortality and SDI showed a significant inverse relationship where increases in SDI correlated with sharp decreases in neonatal AMR-associated and attributable mortality (*r*=–0·85 [95% CI –0·85 to –0·58] and –0·73 [–0·84 to –0·56]; appendix pp 96–97). Countries in the WHO African region with more advanced stages of NAP development and implementation did not appear to be significantly associated with reduced age-standardised mortality rates attributable to and associated with AMR when compared with countries without an NAP or one currently under development (figure 1C). A comparison of associated and attributable AMR mortality rates does not suggest the highest burden in countries without an NAP, and there was no consistent decreasing trend with increasing stages of NAP development.

Antibiotic consumption, SDI, and water and sanitation indices were all negatively correlated with AMR-attributable mortality burden (correlation coefficient *r*=–0·60 [95% CI –0·75 to –0·37], –0·61 [–0·76 to –0·39], and –0·77 [–0·87 to –0·62], respectively; appendix pp 98–99). Therefore, countries using more antibiotics per capita, with a higher development score or better access to safe water and hygiene, have a lower AMR mortality burden. However, there is a strong positive correlation between DDD per 1000 and SDI as well as between DDD per 1000 and access to water and sanitation (*r*=0·72 [95% CI 0·54 to 0·83]) and 0·67 [95% CI 0·47 to 0·80], respectively)—ie, as SDI and access to improved water and sanitation increases, we observe an increase in antibiotic consumption per capita. However, these demographic features are intercorrelated so their effects cannot be interpreted as independent.

## Discussion

To our knowledge, this study provides the most comprehensive estimates to date describing the burden of AMR in the WHO African region for an extensive list of priority bacterium–antibiotic combinations. The region continues to have a high burden of infection related mortality with an estimated 3·83 million (95% UI 3·17–4·67) deaths in 2019, and the highest age-standardised mortality rate due to 33 priority bacterial pathogens was 230 deaths (185–285) per 100 000 population.^16^ Of the 3·83 million deaths due to infection and 1·86 million deaths due to 33 priority bacterial pathogens in the region, we estimated 1·05 million deaths (829 000–1 316 000) associated with bacterial AMR and 250 000 deaths (192 000–325 000) directly attributable to bacterial AMR. To contextualise in terms of other leading causes of infectious deaths in the region, which have far greater investment, the burden of deaths associated with bacterial AMR exceeds the estimated number of deaths in 2019 due to HIV/AIDS (639 554^12^) and malaria (GBD 2019: 594 348,^12^ World Malaria Report: 386 000^17^).

We have demonstrated that four pathogens, *S pneumoniae, K pneumoniae, E coli,* and *S aureus*, were each responsible for more than 100 000 deaths associated with AMR in this region. Two of these pathogens, *S aureus* and *E coli,* also represent a large burden globally and are considered part of the Sustainable Development Goal indicator for AMR (3.d.2). However, the estimated burden arising from resistant *S pneumoniae* and *K pneumoniae* (195 000 and 184 000 associated deaths, respectively) exceeded that of *E coli* and *S aureus* (147 000 and 136 000 associated deaths, respectively). This differs from the ranking estimated in other regions, such as the high-income super-region and western Europe,^9^ where approximately half of the fatal AMR burden was linked to *S aureus* and *E coli.*^1^

Another difference from other regions was the high prevalence of deaths with TMP-SMX-resistant infections, especially in combination with *S pneumoniae.* TMP-SMX prophylaxis is recommended to prevent opportunistic infections (eg, *Pneumocystis jirovecii* pneumonia and toxoplasmosis) and can also prevent or treat other infections like malaria or those caused by pathogens including *S pneumoniae* and enteric bacteria.^18^ Previous studies suggest that an increase in the use of prophylactic TMP-SMX for people with HIV probably increased TMP-SMX resistance^19^ and could partially explain our findings and the high resistance rates noted in other recent studies.^20^, ^21^ TMP-SMX resistance in *S pneumoniae* could thus increase vulnerability to pneumococcal infections in people with HIV, leading to higher mortality.

TMP-SMX is widely used in Africa as an oral agent for community-acquired pneumonia and urinary tract infections; its high resistance burden in settings where parenteral antibiotic options and timely access to higher acuity care might be limited suggests that it could be the driver of the high AMR-associated and attributable mortality observed.^22^, ^23^, ^24^ Previously, use of amoxicillin or ampicillin and co-selection between these antibiotics and trimethoprim was suggested to explain more trimethoprim resistance variance and be an important driver of population-level trimethoprim resistance.^25^ Even if either proposed driver of resistance is true, the lack of medicines registration and fragile supply chains pose challenges to sparing TMP-SMX or amoxicillin and ampicillin use as a means of mitigating resistance,^26^ especially in LMIC settings such as Africa. There remains an urgent need to streamline and strengthen medicines registration processes, enhance supply chain management, promote, and support local production, and address affordability to reduce AMR burden.^26^

Surprisingly, high antibiotic consumption was associated with lower AMR burden in some countries but this relationship might represent the ameliorating effects of increased quality health-care access and infrastructure, which is associated with higher antibiotic use, rather than direct effects from antibiotic consumption. This finding suggests that we need to look beyond antibiotic use to identify AMR causes. High infection rates have long been acknowledged as drivers of immense mortality and morbidity throughout Africa,^12^, ^16^ and given that prevalence of resistance is comparatively low in this region, our findings implicate these disproportionate rates, especially for specific pathogens, as primary drivers of AMR burden. To reduce AMR burden, renewed investment in vaccine development and distribution to prevent infections is vital, especially against *S pneumoniae, K pneumoniae, E coli*, and *S aureus*. Additionally, our findings identified a higher burden of AMR mortality among neonates (particularly in central and western Africa) and increasing neonatal AMR burden in countries with lower SDI. Expanded access to primary health care and effective antibiotics are equally necessary in vulnerable populations and settings to reduce mortality from infections contributing to AMR burden.

During the COVID-19 pandemic, the Institute of Pathogen Genomics of the Africa Centres for Disease Control and Prevention was successful in sequencing SARS-CoV-2 strains and partnering with organisations such as the WHO Regional Office for Africa and the South African National Bioinformatics Institute.^27^ Given the high burden of AMR in the WHO African region, we suggest that existing programmes such as the Institute of Pathogen Genomics can support efforts to mitigate AMR burden by expanding to include resistance testing of isolates as part of their routine procedures.^28^, ^29^ Examples include adding the four leading pathogens in AMR burden (*S pneumoniae, K pneumoniae, E coli*, and *S aureus*) as priorities for resistance testing and increasing support for specimen collection in regional collaboration centres. In this way, the dual benefit of increasing access to treatment and expanding available data to improve future studies on AMR in the WHO African region can be achieved.

As resources permit, NAPs can be helpful in mobilising cohesive national responses to the AMR burden. In our assessment of AMR in the WHO European region,^9^ development of NAPs was associated with decreases in AMR burden. NAPs are expensive, however, and often require a diverse host of invested parties to create, finance, and evaluate them.^30^, ^31^ This might help explain why only 23 countries in the region had approved, financed, and implemented NAPs for AMR at the time of this analysis. An important challenge is ineffective implementation.^32^ Notwithstanding the limitations of the crude classification of NAP status used in these analyses, the ineffective implementation of NAPs in some countries is demonstrated by the variable progress regarding the development, implementation, or financing of NAPs that has been made in 2019 by countries in the region (figure 1C). The lack of a clear pattern between weaker NAP implementation and higher AMR burden in this analysis is unlike that seen in the European region^9^ and further complicates this comparison. We note that many countries with financed and implemented NAPs were in some instances also among the highest-burden countries in the region (eg, Mozambique, Eritrea, and Zimbabwe). This heterogeneous pattern of AMR-attributable mortality by NAP status might also reflect excessive bureaucracy and related partisanship.^33^, ^34^ We observe that with limited access to safe water and sanitation there is increasing mortality attributable to AMR and that AMR burden is driven primarily by the high infectious disease burden in the region. This highlights an urgent need to scale up action to combat infectious disease and AMR burden in the region by improving access to and use of crucial front-line antibiotics, enhancing government engagement, leveraging infection control measures, and expanding health-care access and WaSH infrastructure. Additionally, even with a well designed NAP, challenges might arise due to limited resources, weak health-care infrastructure, inadequate surveillance systems, and insufficient enforcement of regulations. Improving the development, approval, sustainable financing, and implementation of effective NAPs is essential for the region, especially as existing interventions can now be assessed in terms of their cost and the potential impact on reducing AMR burden. In countries with low access to quality health care and antibiotics, reducing infection is the greatest tool for reducing AMR. Conversely, in countries where access to health care and antibiotics are established, AMR can be reduced through improved antimicrobial stewardship and governance. To elicit a sustained response, it is necessary to have awareness and collaboration between key stakeholders such as policy makers, local communities, and non-governmental organisations.

To effectively tackle AMR, we must first be able to accurately quantify its burden. Comprehensive population-based surveillance-based AMR data are still needed in some countries in Africa, as demonstrated by the available input data used in this analysis. This paucity of data is largely due to insufficiently strong laboratory infrastructure and capacity, limited or missing microbiological facilities, poor-performing health systems governance and information systems, and constrained resources.^35^, ^36^ Available data in some countries are often fragmented, undersampled, or not necessarily representative of the general underlying population. Furthermore, without accessible and well equipped microbiological facilities capable of culturing specimens, we lose access to data from medically underserved populations. This means that burden estimates for data-sparse countries and pathogen–antimicrobial combinations can, in some instances, be inaccurate. In low-resource settings, the scarcity of medical resources and proper interpretative skills for conducting precise microbiological testing poses substantial challenges for estimating AMR burden.^33^ Microbiology laboratories in these settings encounter a multitude of obstacles in terms of infrastructure and technical capabilities. It is crucial to emphasise the importance of investing in high-quality clinical microbiology services to improve not only routine clinical care but also early detection of hospital outbreaks and potential pandemics.^34^ This absence of reliable information hampers health-care professionals' ability to make informed decisions, further contributing to antimicrobial resistance. Addressing this gap is crucial for improving antimicrobial susceptibility data, leading to better treatment strategies and patient outcomes. Notwithstanding the general issue of data scarcity, especially data points that link AMR with mortality and DALYs, there was a notable data paucity on the pathogen distribution by infectious syndrome and the prevalence of resistance data for key pathogen–drug combinations, particularly in central sub-Saharan Africa. Such limited data in some countries was consequential for the prevalence of resistance and relative risk modelling components of our work, and thus we have made several methodological assumptions, as explained in a previous publication.^1^ We cannot exclude the possibility of overestimation or underestimation of prevalence of resistance (eg, data sparse pathogen–drug combinations could be influenced by outliers in neighbouring country-years). However, the estimation process is robust as it is informed by all available data from all the countries across the globe. When data for a specific country are lacking, the estimates can steadily rely on regional patterns, covariates, and out-of-sample predictive validity assessment. Despite our efforts to mitigate and adjust for biases, we cannot discount the impact of selection bias in passive microbial surveillance data. Bias and misclassification can also arise when combining and standardising data from various providers and origins of infection. Additionally, antimicrobial usage might differ between private and public health-care sectors, resulting in an incomplete accounting of AMR patterns. Co-infections and comorbidities can exacerbate the risk, severity, and outcomes of AMR infections, but further research is needed to clarify their roles and specific effects. Finally, despite our use of the most recent CLSI breakpoint guidelines whenever possible,^10^ there are no universal laboratory standards to distinguish resistance versus susceptibility, and deferring to source laboratory interpretation for classifying the isolates might have resulted in heterogeneous classification.

Despite these limitations, our analysis represents one of the most comprehensive analyses of bacterial AMR burden in the WHO African region to date, reflecting the widest and most extensive available range of data, as well as the use of models that have been implemented and refined specifically for incorporating disparate data sources for the GBD analysis. We are confident that both the most pervasive bacterial pathogens and the most effective antimicrobials against them can be more easily identified through this study. This information, when applied with our estimates of pathogen–drug burden, can be used to inform empirical treatment guidelines within specific countries and regions. We echo other studies that highlight critical AMR data gaps in certain parts of the world, which will be of utmost importance to further refine these estimates.

Bacterial AMR is an urgent public health threat in the WHO African region that is compounded by high rates of infection, difficulty implementing NAPs, and stark health-care inequalities across countries throughout the region. Stakeholders will have to contend with these barriers to reduce AMR burden in the coming years. Future studies should include estimates of other important drug-resistant microorganisms, such as additional priority viruses and fungi, to paint a more comprehensive picture of AMR and address critical data gaps. This will be necessary to fully understand the impact of AMR in this region as well as address the contribution of COVID-19 in 2020 and beyond. Currently, our approach serves as a comprehensive foundation upon which pertinent health-care policies can hopefully be made. Our results can aid local governments in the drafting of locally tailored stewardship and infection prevention and control policies to mitigate leading pathogen–drug combinations using the resources available in their country. Addressing AMR in the WHO African region will necessitate targeted efforts and investments that consider the complex drivers behind the disproportionate AMR burden there.

## Data sharing

This study follows the Guidelines for Accurate and Transparent Health Estimates Reporting (GATHER). To download the data used in these analyses, please visit the Global Health Data Exchange (https://ghdx.healthdata.org/record/ihme-data/who-region-africa-amr-2019).

## Declaration of interests

E Chung reports support for the present manuscript from the National Institutes of Health, training grant NICHD T32HD007233. S J Dunachie reports support for the present manuscript from the UK Fleming Fund at the Department of Health and Social Care, the Bill & Melinda Gates Foundation, and the Wellcome Trust; grants or contracts from the UK National Institute for Health and Care Research (NIHR); participation on a Data Safety Monitoring Committee for the UK STABILISE study of BCG vaccine in COPD as a committee member; and leadership or fiduciary roles in board, society, committee or advocacy groups, as a paid member of the Wellcome Trust Vaccines Advisory Selection Panel for “Vaccines and AMR” (November, 2019), a paid member of the Interview Committee of the Wellcome Early Career Awards (2022–25), an unpaid expert advisor to WHO's Global Antimicrobial Resistance Surveillance System (GLASS; November 2018–2022), an unpaid member of the WHO Guidelines Development Group on the treatment of Ebola (2021–23), a paid scientific advisor on COVID-19 immunology to the Scottish Parliament (2021–23), an unpaid member of the Variant Technical Group for SARS-CoV-2 (invited as T-cell specialist) for the UK Health Security Agency (2021 to present), and an unpaid member for UK Government (immunologist) on the New and Emerging Respiratory Virus Threats Advisory Group (NERVTAG; 2023 to present). N A Feasey reports grants or contracts from the Environmental Surveillance for Vaccine Impact Assessment (2022–25), National Institute for Health and Care Research (NIHR) Global Health Professorship (2022–27), NeoTrack from the Bill & Melinda Gates Foundation (2021–23), Chatinkha Seq from the Bill & Melinda Gates Foundation (2020–22), and as a co-investigator for Strength in Places (UK Research and Innovation; 2020–25). C E Moore reports support for the present manuscript from the Wellcome Trust grant for The Global Research on Antimicrobial Resistance (GRAM) Project (R52354 CN001). A Pollard reports grants or contracts from the Bill & Melinda Gates Foundation, Wellcome, Cepi, UK Medical Research Council, NIHR, Serum Institute of India, AstraZeneca, and European Commission; royalties or licenses from AstraZeneca (as a contributor to intellectual property licensed by Oxford University Innovation to AstraZeneca); consulting fees from Shionogi; leadership or fiduciary roles in board, society, committee or advocacy groups unpaid with the UK Department of Health and Social Care's Joint Committee on Vaccination and Immunisation as the Chair, and as a member of WHO's Strategic Advisory Group of Experts on Immunization until 2022. J L Walson reports grants from the Bill & Melinda Gates Foundation to support AMR research in Africa. All other authors declare no competing interests.
