# Supplementary material for: The burden of bacterial antimicrobial resistance in the WHO African region in 2019: a cross-country systematic analysis
Source: Lancet Glob Health. 2023 Dec 19;12(2):e201–16. doi: 10.1016/S2214-109X(23)00539-9 (PMC10805005; doi:10.1016/S2214-109X(23)00539-9)
Supplement: Supplementary appendix [file mmc1.pdf]

THE LANCET
Global Health

Supplementary appendix

This appendix formed part of the original submission and has been peer reviewed. We post it as supplied by the authors.

Supplement to: Antimicrobial Resistance Collaborators. The burden of bacterial antimicrobial resistance in the WHO African region in 2019: a cross-country systematic analysis. *Lancet Glob Health* 2023; published online Dec 19. https://doi.org/10.1016/S2214-109X(23)00539-9.

Table of Contents

Section 1: Abbreviations 3

Section 2: Data sources..4

Section 2.1: Data sources for the WHO African region 4

Section 2.2: Literature review details 5

Section 2.2.1: Maternal sepsis, neonatal sepsis, and LRI aetiology..5

Section 2.2.2: Urinary tract infections aetiology 6

Section 2.2.3: Skin infections aetiology..6

Section 2.2.4: Intra-abdominal infection aetiology 6

Section 2.2.5: Bone and joint infections aetiology...7

Section 2.2.6: Meningitis infection aetiology 7

Section 2.2.7: Relative risk studies for specific drug-pathogen combinations...7

Section 2.2.8: Prevalence of resistance for specific organisms..7

Section 2.3: Exclusion criteria for literature reviews 8

Section 3: Supplementary methods: a summary of the estimation process 8

Section 3.1: GBD 2019 framework..8

Section 3.2: Deaths where infection plays a role and infectious syndrome estimation 9

Section 3.2.1: Input data 9

Section 3.2.2: Data processing and mapping 9

Section 3.2.3: Intermediate cause and infectious syndrome mapping hierarchy with modelling pathways 9

Section 3.2.4: First pathway – deaths where infection plays a role 10

Section 3.2.5: Second pathway – fraction of deaths where infection plays a role by infectious syndrome in each GBD cause 11

Section 3.2.6: Model validation 12

Section 3.3: Case fatality ratios 12

Section 3.3.1: Input data 12

Section 3.3.2: Models ran for each infectious syndrome 12

Section 3.3.3: Modelling framework 13

Section 3.3.4: Predictions and uncertainty..14

Section 3.4: Pathogen distribution..14

Section 3.4.1: Input data 14

Section 3.4.2: Data processing and analysis 14

Section 3.4.3: Dealing with challenges in pathogen distribution appraisal..15

Section 3.4.4: Age-sex splitting and standardizing measures 16

Section 3.4.5: Modelling framework 17

Section 3.4.6: Exceptions and special handling 18

Section 3.4.7: Model validation 19
Section 3.5: Prevalence of resistance 19
Section 3.5.1: Input data 19
Table 3.5.1.1: Core pathogen–drug combinations 19
Table 3.5.1.2: Supplementary pathogen–drug combinations 20
Section 3.5.2: Data processing 20
Table 3.5.2.1: Pathogens in each pathogen super group 21
Section 3.5.3: Modelling framework 22
Section 3.5.4: Resistance profiles 22
Section 3.5.5: Model validation 23
Section 3.6: Relative risk 24
Section 3.6.1: Input data and data processing 24
Section 3.6.2: Modelling overview 24
Section 3.6.3: Model validation 25
Section 3.7: Counterfactuals and AMR estimation 26
Section 3.7.1: Estimating AMR burden with counterfactual of no infection 26
Section 3.7.2: Estimating AMR burden with counterfactual of infection with susceptible organism 27
Section 3.7.3: Excluded combinations 28
Section 3.8: Correlation Analysis 29
Section 3.8.1: Input Data 29
Section 3.8.2: Correlation Method 29
Section 4: Supplementary Tables and Figures 30
Section 5: GATHER Compliance: Guidelines for Accurate and Transparent Health Estimates Reporting 101
Section 6: References 104
Authors' Contributions 106
Managing the overall research enterprise 106
Writing the first draft of the manuscript 106
Primary responsibility for applying analytical methods to produce estimates 106
Primary responsibility for seeking, cataloguing, extracting, or cleaning data; designing or coding figures and tables 106
Providing data or critical feedback on data sources 106
Developing methods or computational machinery 106
Providing critical feedback on methods or results 106
Drafting the work or revising it critically for important intellectual content 107
Managing the estimation or publications process 107

This appendix provides further methodological details and supplementary figures/tables for "*The burden of bacterial antimicrobial resistance in the WHO African Region in 2019: a cross-country systematic analysis*". Parts of this appendix are taken directly from the appendix of the paper "*Global burden of bacterial antimicrobial resistance in 2019: a systematic analysis*"[1], which is also referenced throughout the text.

Section 1: Abbreviations

**Abbreviation**	**Full phrase**
AFRO	WHO African region
AMASS	AutoMated tool for Antimicrobial resistance Surveillance System
AMR	antimicrobial resistance
ATLAS	Antimicrobial Testing Leadership and Surveillance
AWARE	Assessing Worldwide Antimicrobial Resistance Evaluation
BSI	bloodstream infections
CAI	community-acquired infection
CFR	case fatality ratio
cIAI	complicated intra-abdominal infection
cUTI	complicated urinary tract infection
DALYs	Disability-adjusted life-years
DDD	Defined Daily Dose
GAM	generalised additive models
GBD	Global Burden of Diseases, Injuries, and Risk Factors Study
GBS	group B *Streptococcus*
GLASS	Global Antimicrobial Resistance Surveillance System
GLM	generalised linear model
GPR	Gaussian process regression
HAI	hospital-acquired infection
HAQ Index	Healthcare Access and Quality Index
ICD	International Classification of Diseases
ICU	intensive care unit
INFORM	International Network for Optimal Resistance Monitoring
INICC	International Nosocomial Infection Control Consortium
iNTS	invasive non-typhoidal Salmonella
LRI	lower respiratory infection
MCoD	multiple causes of death data
MEPCO	multinomial estimation of partial and composite observations
MICS	Multiple Indicators Cluster Surveys
MR-BRT	meta-regression—Bayesian, regularised, trimmed
MRC	Medical Research Council

PPS HAI	Point Prevalence Survey on Nosocomial Infections and Antibiotic Use
SDI	Socio-demographic Index
SEV	summary exposure value
SOAR	Survey on Antibiotic Resistance
ST-GPR	spatiotemporal Gaussian process regression
TB	tuberculosis
TEST	Tigecycline Evaluation Surveillance Trial
TSAP	Typhoid Fever Surveillance in Africa Program
UI	uncertainty interval
UTI	urinary tract infection
VR	vital registration
WHO	World Health Organization
YLDs	years lived with disability
YLLs	years of life lost

Section 2: Data sources

The data used for this study can be categorised into the following types: multiple causes of death (MCoD), hospital discharge, mortality surveillance, linkage data (mortality only), literature reviews, microbial data with and without outcome, single drug-resistance profiles, pharmaceutical sales, and antibiotic use data;[1] as well as estimates from the Global Burden of Diseases, Injuries, and Risk Factors Study (GBD) 2019.[2] Here we provide principal sources for data stemming from the WHO African region and information on how we have conducted literature review. More detailed information on data inputs and sources are available in the appendix of Murray et al. (2022)[1] and http://ghdx.healthdata.org/record/ihme-data/global-bacterial-antimicrobial-resistance-burden-estimates-2019

Section 2.1: Data sources for the WHO African region

- **Burden of Antibiotic Resistance in Neonates from Developing Societies (BARNARDS):** BARNARDS includes locations in Nigeria, South Africa, Pakistan, Rwanda, Bangladesh, Ethiopia and India from 2015 to 2018.
- **Central African Republic National Laboratory of Clinical Biology and Public Health:** data collected by the Laboratoire National de Biologie Clinique et de Sante Publique in Central African Republic between 2017 and 2020.
- **Childhood Acute Illness and Nutrition (CHAIN) Network antimicrobial resistance data**: CHAIN Network study informs on hospitalised children under 2 years old with acute illness in Bangladesh, BurkinaFaso, Pakistan, Kenya, Malawi, and Uganda.
- **International Nosocomial Infection Control Consortium (INICC) surveillance online system:** data from the INICC data collection software. ICU patient microbiology and hospital data from 50 countries across Latin America, Asia, the Middle East, eastern Europe, and Africa from 2009 to 2020.
- **Invasive Salmonella infections at multiple surveillance sites in the Democratic Republic of the Congo study:** data published as part of the study on invasive Salmonella
- **KEMRI/US Army Medical Research Directorate**
- **Kumasi Centre for Collaborative Research in Tropical Medicine (KCCR), Kumasi, Ghana together with the Bernhard Nocht Institute for Tropical Medicine.** Data on children and adults admitted in hospital with fever: information from children and adults with fever admitted as inpatients at the Bernhard Nocht Institute for Tropical Medicine in Ghana between 2007 and 2015.

- **Lancet Labs:** data obtained from Lancet Laboratories, a network of private laboratories across different sites in Africa.
- **Madagascar – Fondation Merieux:** data collected from inpatients with positive culture admitted in three hospital sites in Madagascar, funded by Fondation Merieux.
- **Malawi Queen Elizabeth Hospital microbiology tests of blood specimens:** microbiology tests of blood specimens from inpatients at the Queen Elizabeth Hospital in Malawi from 1998 to 2016, part of the Institute of Infection and Global Health, University of Liverpool in collaboration with the Malawi-Liverpool-Wellcome Trust and the Wellcome Trust Sanger Institute.
- **Medical Research Council (MRC) Unit The Gambia. Diagnostic antimicrobial susceptibility testing:** information on hospital admission and discharge, pathogens cultured, resistance susceptibility test and antibiotics prescribed between 2005 and 2015 from the MCR Unit The Gambia, now part of the London
- **Pfizer ATLAS Programme:** the Antimicrobial Testing Leadership and Surveillance (ATLAS) database includes the Tigecycline Evaluation Surveillance Trial (TEST), the Assessing Worldwide Antimicrobial Resistance Evaluation (AWARE) and the International Network for Optimal Resistance Monitoring (INFORM) programs. The study spans in coverage across more than 70 countries between 2004 and 2017.
- **World Health Organization (WHO) Global Tuberculosis Programme**
- **GLASS:** Global Antimicrobial Resistance Surveillance System by WHO
- **SOAR:** Survey on Antibiotic Resistance (SOAR) sponsored by GSK.
- **SMART:** Study for Monitoring Antimicrobial Resistance Trends which monitors complicated intra-abdominal infections (cIAIs), complicated urinary tract infections (cUTIs) and respiratory infections worldwide, funded by Merck & Co.
- **South Africa National Institute for Communicable Diseases (NICD):** Aggregated data from South Africa's AMR surveillance in public healthcare centres which is submitted to the GLASS.
- **The Ethiopian AMR surveillance:** conducted from July 2018 to July 2020 across sentinel surveillance sites and the National AMR Surveillance Coordinating Centre for the Ethiopian Public Health Institute.
- **The Typhoid Fever Surveillance in Africa Program (TSAP):** was established by the International Vaccine Institute to obtain comparable incidence data on typhoid fever and invasive non-typhoidal Salmonella disease in Ghana, Burkina Faso, Ethiopia, Guinea Bissau, Kenya, Madagascar, Senegal, South Africa, Sudan, and Tanzania.
- **WHO Meningitis surveillance:** sentinel hospital surveillance of suspected meningitis cases among children under 5 years old and positive cultures, provided by the World Health Organization (WHO) Global Rotavirus, Invasive Bacterial Vaccine Preventable Diseases Surveillance Network Collaboration from 2008 to 2020.
- **St. George's Hospital, University of London - Global Antimicrobial Resistance, Prescribing and Efficacy Among Neonates and Children (SGUL-GARPEC) Project bloodstream infection data:** Penta-sponsored global surveillance network focusing on neonatal and paediatric antimicrobial resistance and the organisms causing blood stream infections.

Section 2.2: Literature review details

We conducted literature searches to obtain input data for the following components in the analysis: maternal and neonatal sepsis aetiology, lower respiratory infections (LRIs) aetiology, urinary tract infections (UTIs) aetiology, skin infections aetiology, meningitis aetiology and case fatality, intra-abdominal infection aetiology, bone and joint infections aetiology, prevalence of resistance, relative risk and length of stay. Literature searches were performed on PubMed using the following search strings, and extracted studies covered the time range 1980–2019. The search string for these searches can be found below. Literature was used in the case fatality ratio, pathogen distribution, prevalence of resistance and relative risk component models and data processing, with details on modelling methods provided here and in the appendix of Murray et al. (2022).[1] Literature studies were also used as input into the modelling of the antibiotic usage covariate.[2]

Section 2.2.1: Maternal sepsis, neonatal sepsis, and LRI aetiology

Aetiology terms, combined with OR:

- Infection (Infect*)
- Microbiology (Microbiolog*
- Aetiology (Aetiolog*)
- Etiology (Etiolog*)
- Virology (Virolog*)
- Bacteriology (Bacteriolog*)
- Fungus (fung*)

AND

Syndrome terms, combined with OR:

Maternal Sepsis

- puerperal sepsis (puerper* sepsis)
- maternal sepsis (matern* sepsis)
- puerperal septicaemia (puerper* septicaemia, American spelling too - septicemia)
- maternal septicaemia (matern* septicaemia, American spelling too - septicemia)
- puerperal infection (puerper* infection)
- maternal infection (matern* infection)
- puerperal bacteraemia (puerper* bacteraemia, American spelling too - bacteremia)
- maternal bacteraemia (matern* bacteraemia, American spelling too - bacteremia)

Neonatal Sepsis

- Neonatal sepsis (Neonat* sepsis within 3 or 5 words of each other)
- Neonatal septicaemia (Neonat* septicaemia within 3 or 5 words of each other, American spelling too - septicemia)
- Infant sepsis (Infant* sepsis)
- Infant septicaemia (Infant* septicaemia, American spelling too - septicemia)
- Neonatal bacteraemia (Neonat* bacteraemia, American spelling too - bacteremia)
- Infant bacteraemia (Infant* bacteraemia, American spelling too - bacteremia)

Lower respiratory infections

- LRI
- Lower respiratory infection
- LRTI
- Lower respiratory tract infection
- Pneumonia

Section 2.2.2: Urinary tract infections aetiology

("complicated"[Title/Abstract] OR "uncomplicated"[Title/Abstract]) AND (("Cystitis/etiology"[majr:noexp] OR "Cystitis/microbiology"[majr:noexp]) OR ("Pyelonephritis/etiology"[marj:noexp] OR "Pyelonephritis/microbiology"[majr:noexp]) OR ("Urinary Tract Infections/etiology"[majr:noexp] OR "Urinary Tract Infections/microbiology"[majr:noexp])) OR ("Urinary tract infections"[tiab] AND ("etiology"[tiab] OR "microbiology"[tiab]))

Section 2.2.3: Skin infections aetiology

(("Cellulitis/epidemiology"[majr:noexp] OR "Cellulitis/etiology"[majr:noexp] OR "Cellulitis/microbiology"[majr:noexp]) OR ("Pyoderma/epidemiology"[majr:noexp] OR "Pyoderma/etiology"[marj:noexp] OR "Pyoderma/microbiology"[majr:noexp]) OR "Pressure Ulcer/microbiology"[majr:noexp])

Section 2.2.4: Intra-abdominal infection aetiology

(("Peritonitis/epidemiology"[majr:noexp] OR "Peritonitis /etiology"[majr:noexp] OR "Peritonitis /microbiology"[majr:noexp]) OR ("Intraabdominal infections/epidemiology"[majr:noexp] OR "Intraabdominal infections /etiology"[marj:noexp] OR "Intraabdominal infections /microbiology"[majr:noexp]) OR ("abdominal

abscess/epidemiology"[majr:noexp] OR " abdominal abscess /etiology"[majr:noexp] OR "abdominal abscess/microbiology"[majr:noexp]))

Section 2.2.5: Bone and joint infections aetiology

("Osteomyelitis/etiology"[majr:noexp] OR "Osteomyelitis/microbiology"[majr:noexp] NOT 'chronic') OR ("Arthritis, infectious/etiology"[marj:noexp] OR "Arthritis, infectious/microbiology"[majr:noexp] NOT ‘lyme’)

Section 2.2.6: Meningitis infection aetiology

((meningitis[title]) AND (1990/05/01[PDat] : 2018/12/31[PDat]) AND ((etiolog*[title/abstract]) AND Humans[MeSH Terms])

Section 2.2.7: Relative risk studies for specific drug-pathogen combinations

("Acinetobacter baumannii"[MeSH Terms] AND "carbapenem resistance"[All Fields]) OR ("Acinetobacter baumannii"[MeSH Terms] AND "carbapenem resistant"[All Fields])

('Escherichia coli'[MeSH Terms] AND 'carbapenem resistance'[All Fields]) OR ('Escherichia coli'[MeSH Terms] AND 'carbapenem resistant'[All Fields])

('Escherichia coli'[MeSH Terms] AND 'fluoroquinolone resistance'[All Fields]) OR ('Escherichia coli'[MeSH Terms] AND 'fluoroquinolone resistant'[All Fields])

('Escherichia coli'[MeSH Terms] AND 'third generation cephalosporin'[All Fields]) OR ('Escherichia coli'[MeSH Terms] AND ESBL OR extended-spectrum beta lactamase'[All Fields])

('Klebsiella pneumoniae'[MeSH Terms] AND 'third generation cephalosporin'[All Fields]) OR ('Klebsiella pneumoniae'[MeSH Terms] AND 'ESBL OR extended-spectrum beta lactamase'[All Fields])

('Klebsiella pneumoniae'[MeSH Terms] AND 'carbapenem resistance'[All Fields]) OR ('Klebsiella pneumoniae'[MeSH Terms] AND 'carbapenem resistant'[All Fields])

('Streptococcus pneumoniae'[MeSH Terms] AND 'penicillin resistance'[All Fields]) OR ('Streptococcus pneumoniae'[MeSH Terms] AND 'penicillin resistant'[All Fields])

('Pseudomonas aeruginosa'[MeSH Terms] AND 'carbapenem resistant'[All Fields] AND 'mortality' [MeSH Terms]) OR ('Pseudomonas aeruginosa'[MeSH Terms] AND 'carbapenem resistant' AND 'mortality' [All Fields])

('Enterococcus faec*'[MeSH Terms] AND 'vancomycin-resistant'[All Fields])

("haemophilus influenzae"[MeSH Terms] AND ("penicillin resistance"[MeSH Terms] OR ("penicillin"[All Fields] AND "resistance"[All Fields]) OR "penicillin resistance"[All Fields])) AND ("mortality"[Subheading] OR "mortality"[All Fields] OR "mortality"[MeSH Terms])

("streptococcus agalactiae"[MeSH Terms] AND ("azithromycin resistance"[MeSH Terms] OR ("azithromycin "[All Fields] AND "resistance"[All Fields]) OR " azithromycin resistance"[All Fields] OR "penicillin resistance"[MeSH Terms] OR ("penicillin"[All Fields] AND "resistance"[All Fields]) OR "penicillin resistance"[All Fields] OR "clindamycin resistance"[MeSH Terms] OR ("clindamycin"[All Fields] AND "resistance"[All Fields]) OR "erythromycin resistance"[All Fields] OR "erythromycin resistance"[MeSH Terms] OR ("erythromycin"[All Fields] AND "resistance"[All Fields]) OR "clindamycin resistance"[All Fields]) AND ("mortality"[Subheading] OR "mortality"[All Fields] OR "mortality"[MeSH Terms])

Section 2.2.8: Prevalence of resistance for specific organisms

Medical Subject Heading (MeSH) terms with free text terms in the title and abstract fields for Escherichia coli, Klebsiella pneumoniae, Streptococcus pneumoniae and Staphylococcus aureus with the terms for antimicrobial drug resistance (resistan*, suscept*, surveil*, etc), limited from 1990 up to the search date. The search was undertaken on MEDLINE, Ovid Embase, Global Health, Cochrane Library.

Medical Subject Headings (MeSH) and free text terms for the pathogens of interest (e.g. S. Typhi, S. Paratyphi A, enteric fever) with terms for antimicrobial resistance (e.g. resistan*, suscept*, surveil*). The search was undertaken on MEDLINE, Ovid Embase, Global Health, Cochrane Library, Scopus, Web of Science-Core Collection and LILACS regional WHO database.

Medical Subject Heading (MeSH) terms with free text terms in the title and abstract fields for non-typhoidal Salmonella or Salmonellosis (non-typhi or nontyph or non-typh Salmonel…) with the terms for antimicrobial drug resistance (resistan*, suscept*, surveil*, etc) and invasive (blood stream infection, septicaemia etc), limited from 1990 up to the search date. The search was undertaken on MEDLINE, Ovid Embase, Global Health, Cochrane Library, Scopus, Web of Science-Core Collection and LILACS regional WHO.

Medical Subject Heading (MeSH) terms with free text terms in the title and abstract fields for Shigella or Shigellosis with the terms for antimicrobial drug resistance (resistan*, suscept*, surveil*, etc), limited from 1990 up to the search date. The search was undertaken on MEDLINE, Ovid Embase, Global Health, Cochrane Library, Scopus, Web of Science-Core Collection and LILACS regional WHO database.

Medical Subject Heading (MeSH) terms with free text terms in the title and abstract fields for Neisseria gonorrhoeae, with the terms for antimicrobial drug resistance (resistan*, suscept*, surveil*, etc), MDR, XDR, limited from 1990 up to the search date. The search was undertaken on MEDLINE, Ovid Embase, Global Health, Cochrane Library, Scopus, Web of Science-Core Collection and LILACS regional WHO database.

Section 2.3: Exclusion criteria for literature reviews

Studies were excluded from full text review if:

- The study did not include at least one of the following: *E.coli, K.pneumoniae, S.pneumoniae, S.aureus or S.typhi/paratyphi*
- The entire study was conducted before 1990
- Samples were collected before 1990
- Did not perform resistance testing
- Sample is non-representative (lab strains, only resistant strains)
- Included non-human samples
- Article type was a case study
- Article type was a commentary, editorial or review with no primary data
- Isolates were not from blood culture
- There were duplicated isolates
- Travellers/non-endemic country/ no location information
- Study did not test susceptibility to antimicrobials
- There were fewer than 10 consecutive isolates used for susceptibility testing
- Could not locate the full text
- The study was uninterpretable due to poor data quality
- Studies where data was aggregated with other pathogens
- Studies using non-sterile site/mixed isolates
- Studies with no iNTS AST data

Section 3: Supplementary methods: a summary of the estimation process

Section 3.1: GBD 2019 framework

The study relies on Global Burden of Disease (GBD) 2019 fatal and non-fatal estimates, and a comprehensive description of data sources, data quality, statistical modelling and analyses for GBD 2019 have been reported elsewhere.[2] A brief summary of the fatal and non-fatal estimation processes can be found in the appendix of Murray et al. (2022).[1]

Section 3.2: Deaths where infection plays a role and infectious syndrome estimation

Section 3.2.1: Input data

Multiple causes of death (MCoD) data are individual-based records that provide underlying causes of death and two or more intermediate causes in the chain of death. Additionally, each record includes age, sex, residence, and the date of death.

Hospital record with multiple diagnoses and discharge status of death represents an individual-based hospital record of a patient that provides the main diagnosis and two or more additional diagnoses. Additionally, each record includes age, sex, residence, date of admission, date of discharge, and outcome (dead or alive). Only hospital discharges with discharge status of death were used in this component model, since we aimed to estimate the fraction of deaths that involve infection and the infectious syndrome distribution of those deaths.

Linkage data are generated using probabilistic methods in a defined population that link individual-based hospital data to individual-based MCoD data. Linkage data offer a wider dataset that includes main diagnosis, other diagnoses, underlying cause of death, and intermediate causes of death in the chain.

Section 3.2.2: Data processing and mapping

Within the WHO European region, data for Italy has been extracted at the subnational level by GBD 2019 age groups, sex, year, and causes of death and/or diagnoses, while data for the remaining countries have been analysed at the national level. This allowed us to expand the location-years of data that we had for each Socio-demographic Index (SDI)[3] value.

Prepared data were mapped to GBD causes. The GBD cause list is a mutually exclusive and collectively exhaustive list of diseases and injuries. The GBD cause list is organised hierarchically to accommodate different purposes and needs of various users. The first two levels aggregate causes into general groupings. At Level 1, there are three cause groups: communicable, maternal, neonatal, and nutritional diseases (Group 1 diseases); non-communicable diseases (Group 2); and injuries (Group 3). These Level 1 aggregates are subdivided at Level 2 of the hierarchy into 22 cause groupings (eg, neonatal disorders, neurological disorders, and transport injuries). The disaggregation into Levels 3 and 4 contains the finest level of detail for causes captured in GBD 2019. See section 14, table S1 for the full GBD cause hierarchy by level.

The underlying cause of death or main diagnosis for each record in the data was mapped to a GBD cause. After the mapping of underlying cause, we used the GBD 2019 garbage code redistribution algorithm (see appendix 1, section 2.4 in Vos et al.[2]) to ensure that all deaths had a plausible and specific underlying cause of death. The redistribution of garbage codes for underlying causes of death followed the same age and sex restrictions as GBD 2019. We did not redistribute garbage codes in the chain causes because the concept of a garbage code applies only to plausible underlying cause of death (see Rudd et al.[4] and appendix 1, section 2.5 in Vos et al.[2]).

Section 3.2.3: Intermediate cause and infectious syndrome mapping hierarchy with modelling pathways

Within our modelling framework, an infectious syndrome is the infection directly responsible for sepsis and serves as the bridge between the underlying cause of death and sepsis. Infectious syndromes can be both underlying causes of death and intermediate causes of death.

For mapping underlying and intermediate causes of death and hospital diagnoses to sepsis and infectious syndromes, we designed a new map, called "*AMR, sepsis, and infectious syndrome map*". This map is a list of mutually exclusive and collectively exhaustive infectious syndromes that we divided into four levels to form the infectious syndrome hierarchy.

Each level of infectious syndrome is mutually exclusive and collectively exhaustive. Furthermore, the infectious syndrome hierarchy is internally consistent across any metric (eg, number, cause fraction)—aggregating across Level 3 syndromes gives us Level 2 syndromes, aggregating the Level 2 syndromes gives us Level 1 syndromes, and the total of Level 1 syndromes is equal to the value of sepsis (figure 4.4.2.1).

Level 0: All International Classification of Diseases 9th (ICD-9) or 10th revision (ICD-10) coded deaths divided into three groups: explicit sepsis (any death with the specific ICD code for sepsis in the MCoD chain or hospital

diagnoses), implicit sepsis (any death with an infectious disease code in the underlying cause or cause chain, as well as with a specific organ dysfunction) and non-sepsis (any death that does not meet either of the two aforementioned criteria). More information can be found in the appendix of Murray et al. (2022).[1]

Explicit sepsis (A40, R65.2 in ICD-10 and 039 in ICD-9): Any death has specific ICD code for sepsis in the MCoD chain or hospital diagnoses was considered explicit sepsis.[4]

- Implicit sepsis: Any death that has an infectious disease code in the underlying cause or cause chain and a specific organ dysfunction code was considered implicit sepsis
- Non-sepsis: Any death that does not meet either of the two above criteria (section 14, tables S2, S3)

Of the estimated infection-related deaths with explicit sepsis or implicit sepsis and infectious diseases, 59.4% occur with communicable, maternal, neonatal, and nutritional underlying causes of death. 38.9% infection related deaths occur with non-communicable disease as the underlying cause of death, and 1.7% occur with injuries as the underlying cause of death.

Level 1: All implicit and explicit sepsis deaths were divided into 12 Level 1 infectious syndromes and an "other" category. These are as follows: 1) Bacterial infections of the skin and subcutaneous systems; 2) Bloodstream infections; 3) Gonorrhoea and chlamydia; 4) Diarrhoea; 5) Endocarditis and other cardiac infections; 6) Infections of bones, joints and related organs; 7) Lower respiratory infections and all related infections in the thorax; 8) Meningitis and other bacterial central nervous system infections; 9) Peritoneal and intra-abdominal infections; 10) Tuberculosis; 11) Typhoid, paratyphoid, and invasive non-typhoidal *Salmonella;* 12) Urinary tract infection and pyelonephritis; 13) Other infections

Level 2: Each Level 1 infectious syndrome was divided into Level 2 infectious syndromes based on the pathogen type (eg, bacterial, fungal, viral) causing the infection. Examples include specified bacterial, unspecified bacterial, fungal, viral, and unspecified pathogen.

Level 3: Each specified bacterial infectious syndrome in Level 2 was divided to Level 3 infectious syndromes by the culprit bacterial pathogen. Table S3 (section 14) shows this list and bacterial hierarchy.

Due to our data often having multiple diagnoses associated with each record, a single case of sepsis could potentially map to multiple candidate infectious syndromes. Because multiple infectious syndrome assignments pose a risk of double counting, we employed an informative ranking hierarchy. The informative ranking allowed us to determine the infectious syndrome that provided the most information on the culprit pathogen. The goal of this hierarchy was to produce the most accurate pathogen burden estimate such that when there were multiple infectious syndromes, we prioritised the syndrome with the most distinctive distribution. For example, bloodstream infections (BSIs) are common infections in sepsis but there is often an earlier source of the infection such as a UTI, cellulitis, or LRI, and each has a unique pathogen distribution that provides more information than the distribution of BSI. In the event that a patient record reflected both BSI and LRI, we would assign the infectious syndrome based on the pathogen distribution that would be the most proximal aetiologic syndrome, LRI (please refer to the appendix of Murray et al. (2022)[1] for more information).

After mapping the underlying and chain causes of death, our database went through two separate modelling pathways. The first model estimated the fraction of deaths that are sepsis-related in each GBD cause; these sepsis-related deaths for non-infectious GBD causes were combined with GBD deaths for infectious causes to create the total envelope of all deaths where infection plays a role. The second pathway estimated each infectious syndrome as a fraction of sepsis-related mortality in each GBD cause. In the last step of infectious syndrome estimation, the fractions of sepsis by Level 1 infectious syndromes were squeezed to sum to one so as to not exceed the sepsis mortality envelope and multiplied by the sepsis estimate in each GBD cause by country and territory, age, and sex in 2019.

Section 3.2.4: First pathway – deaths where infection plays a role

We used a mixed-effects binomial logistic regression to model the logit of the fraction of sepsis-related deaths by GBD cause-age-sex-location, consistent with the modelling approach used by Rudd et al.[4] Sex and Healthcare

Access and Quality Index (HAQ Index)[2] were included as covariates and a nested random effect on underlying cause of death was included. A separate model was run for each GBD 2019 age group (0–6, 7–27, 28-364 [days], 1–4, 5–9, 10–14, 15–19, 20–24, 25–29, 30–34, 35–39, 40–44, 45–49, 50–54, 55–59, 60–64, 65–69, 70–74, 75–79, 80–84, 85–89, 90–94, 95+ [years]):

$$sepsis\ related\ deaths \sim B(total\ deaths, sepsis\ fraction) \tag{3.2.4.1}$$

$$logit(sepsis\ fraction) = \beta_0 + \beta_1 * HAQ\ Index + \beta_2 * sex + \pi_{level\ 1, level\ 2}$$

Where $\pi_{level\ 1, level\ 2}$ is a nested random effect on underlying cause of death. The nested random-effect's structure in the model on underlying cause of death allowed the prediction of sepsis fractions where data were limited by borrowing information from diseases within the same group. There were 22 groups of underlying causes of death, each categorised by physiological relatedness. We produced our predictions and uncertainty intervals (UIs) by generating 1000 draws from the normal distribution of the fixed coefficients, separately for each GBD location, age group, sex, and cause in 2019. The means of our results were used for the point estimates and the 95% UIs were delineated using the 2.5th and 97.5th percentiles of the draws. Uncertainty is attributable to sample size variability between data sources, data availability, and model specifications.

All underlying causes of death that are infectious diseases were included in the model; however, for these causes we used the GBD death estimates rather than the modelled sepsis estimate, since infection inherently plays a role in these deaths even if the pathway doesn't include sepsis. These causes and their associated infectious syndromes are available in the appendix of Murray et al. (2022).[1] For all other causes, we calculated the number of sepsis-related deaths in 2019 by multiplying our predictions of cause-, age group-, sex-, year-, and location-specific sepsis fractions by GBD 2019 death estimates. Finally, we aggregated our results to arrive at regional and global sepsis-related mortality in non-infectious underlying causes of death, which we combined with the GBD infectious disease deaths estimates to create the mortality envelope of all deaths related to infection.

Section 3.2.5: Second pathway – fraction of deaths where infection plays a role by infectious syndrome in each GBD cause

We used a mixed-effects binomial logistic regression to model the logit of the infectious syndrome fraction of sepsis-related mortality by GBD cause. The model covariates varied by infectious syndrome, and all models included HAQ Index as a covariate and most included a summary exposure value (SEV) scalar calculated for GBD 2019. To more accurately estimate the burden of pathogens responsible for infection, we separated infectious syndromes into hospital-acquired and community-acquired for LRI+ and UTI. More details on the infectious syndrome model covariates and age groups are found in the appendix of Murray et al. (2022).[1]

The infectious syndrome models were specified as mixed-effects binomial logistic regressions, one for each infectious syndrome and age group:

$$syndrome\ related\ deaths \sim B(total\ sepsis\ deaths, syndrome\ fraction) \tag{3.2.5.1}$$

$$logit(syndrome\ fraction) = \beta_0 + \beta * X + \pi_{level\ 1, level\ 2}$$

where β and X are vectors of length $n + 1$ for n covariates and $\pi_{level\ 1, level\ 2}$ is a nested random effect on underlying cause of death. The granularity of the age groups estimated for each infectious syndrome was chosen based on the age pattern of the infectious syndrome and the limitations of data sparsity.

As in the first pathway, we derived our predictions and UIs by generating 1000 draws from the normal distribution of the fixed coefficients separately for each GBD location, age group, sex, and cause in 2019. We used the means of our results for the point estimates and the 95% UIs were delineated using the 2.5th and 97.5th percentiles of the draws.

We calculated the number of deaths attributable to each infectious syndrome in 2019 by multiplying our predictions of cause-, age group-, sex-, year-, and location-specific infectious syndrome fractions by our sepsis-mortality

estimates from the first pathway. All infectious syndrome fractions were squeezed to sum to one prior to multiplication in order to ensure that we did not exceed the sepsis mortality envelope.

Out of the 12 explicit Level 1 infectious syndromes included in our hierarchy, we excluded (i) tuberculosis (TB), (ii) typhoid, paratyphoid, and invasive non-typhoidal Salmonella, and (iii) gonorrhoea and chlamydia from our binomial mixed-effects linear regression model. Instead, we used the published results from GBD 2019[5] for these causes of death, as we believe the GBD 2019 estimates fully represent these infectious syndromes because they are usually not intermediate causes of death.

Section 3.2.6: Model validation

Infectious syndrome modelling aims to predict which cases of infection belong to a specific infectious syndrome, which is a multi-class classification problem. We therefore use the Area Under the Receiver Operating Characteristics (ROC) Curve (AUC) to evaluate model performance. The ROC Curve is determined by the sensitivity (or true positive rate) and the specificity (or false positive rate) of the model, and a higher AUC score indicates that the model is capable of discerning between the different categories. Accuracy is a related measure which considers the proportion of true positives and true negatives predicted by the model with respect to the total number of predictions. More information on this can be found in the appendix of Murray et al. (2022).[1]

Section 3.3: Case fatality ratios

Section 3.3.1: Input data

Case fatality ratios (CFRs) were modelled for the pathogens and infectious syndromes of interest using all available data detailing the organism responsible for infection, the infectious syndrome, and patient outcome, which included hospital and microbial data. Input data for the CFR models were aggregated based on data source, year, GBD location, and age group (as well as hospital/community acquired status, in the case of the lower respiratory and urogenital infectious models). For lower respiratory and blood stream infections, for which CFRs could be vastly different in neonates, we modelled the following age groups: neonatal, post-neonatal–5 years, 5–50 years, 50–70 years, and 70 years and older. For all other infectious syndromes, we modelled the following age groups: neonatal–5 years, 5–50 years, 50–70 years, and 70 years and older. We excluded from the analysis any source-location-year-age with fewer than five cases and zero deaths.

To allow us to implement linear models, CFRs were logit-transformed. We used the delta method to compute the standard error of CFRs in logit space. To incorporate data with zero deaths, or with an equal number of deaths and cases, we applied a 1% offset, such that the CFRs for data with zero deaths was represented as 1% and the CFR for data with an equal number of deaths and cases was represented as 99%.

Pathogen-specific CFRs were modelled separately by infectious syndrome and were calculated as a function of HAQ Index and age. To account for heterogeneity across the sources of input data, we implemented a mixed-effects meta-regression framework, modelling data source as a random effect. We further incorporated a binary fixed-effect denoting whether the data source only included intensive care unit (ICU) patients, for which CFRs were expected to be higher. The pathogens of interest for each infectious syndrome were determined by prevalence in the data and expert opinion, with the goal of modelling approximately 90% of specified-pathogens associated with each infectious syndrome.

Section 3.3.2: Models ran for each infectious syndrome

The interaction of the HAQ Index fixed-effect with the pathogen-specific fixed-effect allowed the relative deadliness of pathogens to vary depending on a location's HAQ Index – this is termed an 'interaction model'. For those pathogens with fewer than ten high quality data points below 0.7 HAQ Index, or those whose results in the interaction models indicated an unrealistically large influence of HAQ Index (eg, 70% CFR in low HAQ Index countries, 1% CFR in high HAQ Index countries), we modelled a pathogen-specific intercept with an HAQ Index fixed-effect shared across the pathogens. As a consequence of the single fixed-effect on HAQ Index, a pathogen that was predicted to be the deadliest in low HAQ Index countries would also be predicted to be the deadliest in high HAQ Index countries in these 'intercept models.' To estimate the CFRs for other known bacteria, which either were not selected as a pathogen of interest or lacked sufficient data for inclusion in the intercept models, we pooled all

bacterial data together and estimated a single CFR curve from age, HAQ Index, and the data source heterogeneity covariates. Thus, up to three models were run for each infectious syndrome:

1) an interaction model including data for all data rich pathogens and 'other specified bacteria' (which was included to inform the overall influence of HAQ Index on CFR, predictions were only generated for the data rich pathogens),
2) an intercept model including data for data rich and data sparse pathogens, as well as 'other specified bacteria' (predictions were only generated for the data sparse pathogens), and
3) an 'other bacteria' model that included data for all bacterial pathogens (predictions were generated by HAQ Index and age, without any pathogen specific term).

For some infectious syndromes, the relative deadliness of a pathogen may be strongly determined by either the age of the patient or whether the infection was community- or hospital-acquired. For bloodstream infections, we ran two distinct sets of CFR models, one for neonates (0–27 days) and another for post neonates, to capture the differing dynamics of pathogen deadliness in these two populations. As is done for our other modelling processes, we also separate community-acquired and hospital-acquired cases in our CFR models for lower respiratory and urogenital infections. Because some data sources did not provide enough information to infer whether an infection was community- or hospital-acquired, but still included important information on the relative pathogenesis and the difference in CFRs across varying HAQ indices, infections of unknown origin were included in both the community-acquired and hospital-acquired models for these two syndromes. Any bias in these 'unknown origin' infections was adjusted for using a binary fixed-effect representing an 'unknown origin' infection, and predictions were generated for the community- and hospital-acquired infections only.

Section 3.3.3: Modelling framework

The data were analysed using a meta-analytic mixed effects structure. The main model can be specified as follows:

$$logit(y_i) = X_i\beta + u_i 1 + \epsilon_i, \qquad \epsilon_i \sim N(0, \Sigma_i), \qquad u_i \sim N(0, \gamma) \tag{3.3.3.1}$$

where

- y_i contains CFRs for data source i
- Design matrix X_i contains as columns the following covariates
    - in all models:
        - HAQ Index
        - dummy-coded indicator for age group
        - dummy-coded ICU indicator for data source (1 if data source only compiles information on ICU patients, 0 if a mix between ICU/non-ICU patients)
    - in 'interaction' and 'intercept' models:
        - dummy-coded indicator for pathogen
    - in 'interaction' models only:
        - interaction between pathogen and HAQ Index (product of dummy-coded pathogen columns and HAQ Index)
    - in models evaluating community/hospital acquired infection (LRI+, UTI):
        - dummy-coded variable indicating source of infection (1 if unknown source, 0 if community OR hospital acquired, depending on whether the model is evaluating community or hospital infections)
- β are fixed effect multipliers
- ϵ_i are observation error terms with known variances
- u_i are data source-specific random intercepts with unknown covariance γ

The underlying program used to fit the model (meta-regression, Bayesian, regularized, trimmed [MR-BRT]) is described elsewhere.[6] The program allows specification of priors on γ and β.

Section 3.3.4: Predictions and uncertainty

Predictions for 2019 CFRs were generated for each country, age group, and pathogen as a function of each country's HAQ Index, assuming mixed ICU/non-ICU patients and, in the case of models for UTI and LRI+, that the infection was community- or hospital-acquired (in contrast to infections of unknown origin). For pathogens with insufficient data to estimate a syndrome-specific CFR, we predicted out using the 'other bacteria' CFR associated with the infectious syndrome. Importantly, all of the CFRs we calculate by infectious syndrome are independent of that syndrome's underlying cause.

Uncertainty estimates were generated using asymptotic uncertainty intervals. Specifically, for the model, the posterior uncertainty for the coefficients β is Gaussian, with mean and variance given below:

$$\hat{\beta} = \left(\sum_i X_i^T V_i^{-1} X_i\right)^{-1} \left(\sum_i X_i^T V_i^{-1} y_i\right) \quad (3.3.4.1)$$

$$Var(\hat{\beta}) = \left(\sum_i X_i^T V_i^{-1} X_i\right)^{-1} \quad (3.3.4.2)$$

where

$$V_i = 11^T + \hat{\gamma} I \quad (3.3.4.2)$$

The variance-covariance matrix was used to obtain 1000 draws for the coefficients, which are then used to get intervals for the predictions.

Section 3.4: Pathogen distribution

Section 3.4.1: Input data

With this model, we aimed to estimate the distribution of pathogens causing each infectious syndrome. To get input data for this model, we gathered all available data sources described in section 2 that meet the following criteria:

- Sufficient diagnosis (for patient- or admission-level datasets) or sample specimen type (for isolate- or culture-level datasets) information for us to determine the infectious syndrome
- Information on which pathogen(s) caused the infection or which pathogen(s) were detected in an infectious sample, as determined through culture or genomic-based methods
- Did not have a strongly biased sampling framework across pathogens (for example, did not deliberately sample until 100 cases of every pathogen of interest had been obtained)

The input data source types that met these criteria in this study were:

- Multiple causes of death data
- Hospital discharge
- Linkage data
- Microbial data with and without outcome information
- Literature studies from the aetiology literature reviews

Section 3.4.2: Data processing and analysis

We extracted and standardised the location, year, age, sex, diagnoses, specimen type, pathogens, and hospital- and community-acquired (HAI and CAI) status of each record in every dataset. These datasets report a variety of metrics, including deaths, admissions, cases, cultures, and isolates. While these metrics are not completely comparable (for example, a single patient may often have multiple cultures taken during a single hospital admission), we chose to standardise them into two categories: "deaths," for any unit associated with an outcome of death, and "cases," for any unit regardless of outcome. After standardising the data, we mapped every sample ID or tabulated figure in the data to infectious syndrome based on its diagnoses and specimen type. More details on this process can be found the appendix of Murray et al. (2022).[1]

Some pathogens cause disease so rarely or are so commonly contaminants that we considered them to be contaminants, unlikely to be the true cause of disease. Examples include many *Corynebacterium* species and

Staphylococcus epidermidis. We dropped all such contaminants from the analysis, as well as any record listed by treating clinicians in the data as a contaminant. We also dropped from the analysis all records where no pathogen was detected, or the patient diagnosis indicated an unspecified bacterium. This assumes that the distribution of pathogens among cases with known aetiology are the same as those with unknown aetiology; in other words that the probability of detection is the same for every pathogen. This assumption may break down if certain pathogens are more difficult to detect than others, or in cases where a pathogen is irregularly tested for within a laboratory.

For data sources where multiple pathogens were listed per sample ID, we classified these cases according to the following criteria. First, if a case contained more than one of "unspecified bacteria," "virus," "fungus," and another pathogen(s), we chose to drop all these pathogens except the one(s) most likely to be responsible for disease, with the following ranking from most to least likely: 1. Another pathogen(s); 2. Unspecified bacteria; 3. Virus; 4. Fungus. This was to drop co-occurrence profiles that we consider to be uninformative, like a viral infection co-occurring with a fungal infection. After applying this drop, we considered any sample ID that contained more than one pathogen to be polymicrobial. Polymicrobial was treated as a distinct pathogen category in all further analysis, and we were unable to include any AMR burden from polymicrobial infections in our final results, which possibly underestimates the burden of AMR by hiding infections caused by resistant pathogens of interest in the polymicrobial category.

Furthermore, in our approach we chose to assume that the relative prevalences of pathogens in datasets that do not report co-occurrence would be comparable to their mono-pathogenic counterparts in datasets that do report co-occurrence. This assumes that the co-occurrence of pathogens is random and is not correlated for certain pathogens. We did not have sufficient data to fully test the validity of this assumption, given that few datasets report the full universe of pathogens which may co-occur. When selecting pathogens for estimation, we took into account that the set of estimated pathogens for each infectious syndrome is mutually exclusive and collectively exhaustive of all possible aetiologies. Polymicrobial infections were either estimated explicitly or included in the "other" category, making all explicitly estimated individual pathogens mono-pathogenic. Additional factors that were considered can be found in the appendix of Murray et al. (2022).[1]

Section 3.4.3: Dealing with challenges in pathogen distribution appraisal

One of the central challenges of estimating pathogen distributions was that not every data source tested for or reported every possible aetiology of a given infectious syndrome. For example, many literature studies on the aetiologies of meningitis only report on bacterial aetiologies, and some surveillance systems only collect data on certain pathogens of interest. Only certain pathogens are referenced explicitly in the International Classification of Diseases (ICD), limiting which pathogens can be identified from ICD-based data types like MCoD and hospital discharge. Finally, some datasets reported only a subset of the pathogens that we are interested in for a given infectious syndrome, reporting the remaining aetiologies in an aggregate "other" category. These practices have led to inconsistencies in the "other" and "polymicrobial" categories across data sources. Datasets can either over or under-report "other," and datasets that report fewer specific pathogens will automatically report fewer polymicrobial infections.

To address this problem, we maintained a list of data sources that we believe have sufficient testing and reporting to give unbiased estimates of other and polymicrobial for all syndromes, dropping any data on polymicrobial or other that did not come from these data sources. These data sources all had a complete sampling framework (eg, they do not limit the scope of aetologies that they test for) and reported their results without any deliberate aggregation. While we believe this list provided an accurate starting place for the estimation of other and polymicrobial, future work to improve this method would involve a more detailed analysis of sampling framework and reporting categories in each dataset, specific to each infectious syndrome.

There were two major exceptions to this method for handling "other specified pathogens." First, determining the pathogenic aetiology of LRI with microbiology represents challenges that have been well described previously.[7,8] In order to account for this limitation, we utilised a vaccine probe design to inform the *Streptococcus pneumoniae* cause fraction of LRI, consistent with the approach used in the GBD aetiology estimation process.[9,10] In brief, we extracted the vaccine efficacy of the pneumococcal vaccine against all pneumonia from 18 vaccine probe studies with randomised-control trial, before-after, and cohort designs among children and adults. We then calculated the

PAF of pneumonia due to *S. pneumoniae* in each study ($Strep\ Base\ PAF$) based on these vaccine efficacies ($VE_{all\ pnuemonia}$), the vaccine efficacy of pneumococcal vaccine against vaccine-type pneumococcal pneumonia as pooled from three studies (two in children and one in adults) (VE_{vtpp}), the percentage of the population covered by the pneumococcal vaccine as modelled in GBD (100% for RCTs) (Cov_{PCV3}),[10] and the percent of serotypes covered by the vaccine[11] ($Cov_{serotype}$) (equation 6.2.6.1). We modelled a global age-specific PAF for *S. pneumoniae* based on these data in the MR-BRT environment and finally adjusted this PAF based on the vaccine coverage in children in every GBD location in 2019 and optimal vaccine efficacy in children ($Strep\ Final\ PAF$) (equation 3.4.3.2). In adults (age 5+), we assumed the effects of vaccination on adults would be primarily indirect from vaccination in children, and included an adjustment factor on the vaccine efficacy to account for this, derived from Grijalva et al.[12]

$$Strep\ Base\ PAF = \frac{VE_{all\ pneumonia}}{VE_{vtpp}Cov_{PCV3}Cov_{serotype}} \quad (3.4.3.1)$$

$$Strep\ Final\ PAF = \frac{Strep\ Base\ PAF\left(1 - Cov_{PCV3}Cov_{serotype}VE_{PCV3\ Optimal}\right)}{1 - (Strep\ Base\ PAF)Cov_{PCV3}Cov_{serotype}VE_{PCV3\ Optimal}} \quad (3.4.3.2)$$

In this vaccine probe analysis, ($1 - Strep\ Final\ PAF$) is not consistent with the "other" category in our model, since it includes all non-*S. pneumoniae* aetiologies. We retained all of the data from the vaccine probe analysis as two categories, *S. pneumoniae* and "not *S. pneumoniae*" and addressed the inconsistencies between them and our other data using our modelling framework.

The second major exception involves several literature studies on the proportion of neonatal bacterial meningitis caused by *Streptococcus agalactiae* (Group B *Streptococcus;* GBS). We found that these literature studies were important to our estimation of the pathogen distribution of neonatal meningitis, which is distinct from other age groups because of its high proportion of GBS. However, these studies either only reported or were only extracted with two categories, GBS and "other bacterial, not GBS." We retained both these categories and addressed the inconsistencies between them and our other data using our modelling framework.

Section 3.4.4: Age-sex splitting and standardizing measures

We standardised age and sex across all datasets to the following most-detailed groups using the GBD causes of death age-sex splitting algorithm for age:[2] 0–6, 7–27, and 28–364 days, and 1–4, 5–9, 10–14, 15–19, 20–24, 25–29, 30–34, 35–39, 40–44, 45–49, 50–54, 55–59, 60–64, 65–69, 70–74, 75–79, 80–84, 85–89, 90–94, 95+ years; and sex: male and female. This algorithm assumes that age-sex pattern of the death or case rate for a given infectious syndrome or pathogen is inherent to the pathology of the disease and is therefore constant across location and year. Details on how the algorithm was applied can be found in the the appendix of Murray et al. (2022).[1]

The input data sources reported a variety of combinations of measures, including some that reported deaths only, some that reported cases only, and some that reported both cases and deaths. In order to standardise these measures to cases, we estimated infectious syndrome- and pathogen-specific CFRs (see section 5) and used these CFRs to convert all deaths-only datasets to cases. For any infectious syndrome or pathogen combination for which we did not have enough data to estimate plausible CFRs, we used a set of all-bacteria CFRs for that infectious syndrome instead. All modelling was done in case space.

Several of our microbial databases came exclusively from ICUs and were therefore heavily biased towards severe illness. In order to mitigate this bias, we dropped all information on cases in ICU-only datasets and recalculated implied cases based on reported deaths and our CFRs. No similar adjustment was made to attempt to account for biases between hospitalised and un-hospitalised populations, although we did account for HAI versus CAI for two infectious syndromes – LRI and thorax infections and UTI – within our modelling framework. The use of hospital-based data to calculate both pathogen-specific case fatality ratios and pathogen distributions biases our estimate of the distribution of pathogens in incident cases towards more severe disease, particularly for less-severe infectious syndromes like lower respiratory infections; adjusting for this bias would improve the accuracy of our non-fatal estimates.

Section 3.4.5: Modelling framework

To model the distribution of pathogens for each infectious syndrome, we developed a method for the multinomial estimation of partial and compositional observations (MEPCO). We assumed that the aetiologies of a given infectious syndrome followed a multinomial distribution. Due to inconsistencies in which pathogens are tested for and reported by different data sources, each data source contained partial observations of the possible outcomes of the underlying multinomial distribution. Certain data sources like the vaccine probe estimates and the GBS neonatal meningitis studies represent compositional observations, where pathogens like "not *S. pneumoniae*" and "other bacterial, not GBS" represent aggregates of more detailed pathogens.

In order to use both partial and compositional data, we constructed a network model with the dependent variable as the log ratio of cases between different pathogens and estimated over a flexible parameterisation of multinomial parameters using a maximum likelihood approach. Consider a given infectious syndrome with a multinomial distribution of n mutually exclusive, collectively exhaustive aetiologies with probabilities $p = (p_1, \dots, p_n)$, so that each $p_j \in (0,1)$ and $\sum_j p_j = 1$. The likelihood of an observation of $c = (c_1, \dots, c_n)$, where c_j = number of cases of pathogen j in a total sample of N infections ($\sum_j c_j = N$), is:

$$P(c|p) = N! \prod_{j=1}^{n} \frac{p_j^{c_j}}{c_j!} \qquad (3.4.5.1)$$

We modelled the probabilities using a composition of a link function with a linear predictor:

$$p_{i,j} = \exp\left(x_{i,j}^T \beta_j\right) \qquad (3.4.5.2)$$

for observations i, a vector of covariates $x_{i,j}$, and a vector of coefficientsβ_j for each pathogen j. the appendix of Murray et al. (2022)[1] contains a table with the covariates used for infectious syndrome model, which included a typical specification included an intercept term, HAQ Index, a categorical age group dummy for large age bins, and any relevant vaccine coverage proportions by country. However, we did not observe these probabilities directly. Rather, we observed ratios between sums of these probabilities, which reduce to ratios between sums of cases within each study. These observations therefore take the form:

$$y_i = \frac{cases\ of\ pathogen\ A}{cases\ of\ pathogen\ B} = \frac{\sum_{j=1}^{n} w_{i,j}^a \exp\left(x_{i,j}^T \beta_j\right)}{\sum_{j=1}^{n} w_{i,j}^b \exp\left(x_{i,j}^T \beta_j\right)} \qquad (3.4.5.3)$$

where $w_{i,j}^a$ is a weight of 0 or 1 that selects the mutually exclusive, collectively exhaustive most-detailed pathogens that make up observed pathogen A, which may be a composite observation. For example, for the "other bacterial, non-GBS" pathogen, $w_{i,j}$ would be 1 for *Staphyloccocus aureus, S. pneumoniae, Haemophilus influenzae, Neisseria meningitidis, Listeria monocytogenes, K. pneumoniae, E. coli*, and other pathogens and 0 for GBS and virus. We dropped all observations where either the numerator or denominator had 0 observed cases in order to make this calculation and a forthcoming log transform possible. This may bias the model towards overestimating less common pathogens.

It is not possible to infer all coefficients β_j from the observations since they are all relative. However, if we fix all of the coefficients for one pathogen to 0 as a reference group, then we obtain a well-posed inverse problem, as long as there is enough data to estimate the remaining coefficients. Without loss of generality, we assumed $\beta_1 = 0$ for all elements and obtain estimates of the remaining $\beta_2, \dots, \beta_n$ by minimising the sum of the residuals between log-transformed observations y and corresponding log-transformed predictions from equation 3.3.5.4:

$$\min_{\beta_2,\dots,\beta_n} f(\beta) \coloneqq \sum_i \frac{1}{\sigma_i^2} \left[\ln(y_i) - \ln\left(\sum_{j=1}^{n} w_{i,j}^a\, exp\left(x_{i,j}^T \beta_j\right) \right) + \ln\left(\sum_{j=1}^{n} w_{i,j}^b\, exp\left(x_{i,j}^T \beta_j\right) \right) \right]^2 \qquad (3.4.5.4)$$

where σ_i^2 are variances corresponding to the data points. Equation 3.3.5.4 is a nonlinear likelihood minimisation problem that that we optimised using a standard implementation of the Gauss-Newton method.[13] We then re-normalised the optimal coefficients to obtain final predictions of the probabilities of each pathogen:

$$p_{i,j} = \frac{\exp\left(x_{i,j}^T \beta_j\right)}{\sum_{\hat{j}} \exp\left(x_{i,\hat{j}}^T \beta_{\hat{j}}\right)} \tag{3.4.5.5}$$

To quantify the uncertainty of this estimate, we used asymptotic statistics to obtain the posterior distribution of $(\beta_2, \ldots, \beta_n)$. Specifically, using the Gauss-Newton Hessian approximation gave us the asymptotic information matrix for all β_j except for the reference pathogen, allowing us to sample draws of $\beta = (\beta_1 = 0, \beta_2, \ldots, \beta_n)$. For each β draw and given feature x, we obtained a corresponding draw of p using equation 3.3.3.5.

Finally, to convert $p_{i,j}$ for a given demographic group i from case space to deaths space, we transformed using our CFR estimate for demographic i:

$$p_{i,j}^{deaths} = \frac{p_{i,j} \times CFR_i}{\sum_{\hat{j}} p_{i,\hat{j}} \times CFR_i} \tag{3.4.5.6}$$

This network regression with covariates framework allowed us to use partial and composite data that reported on one or only a few pathogens, or that reported multiple pathogens aggregated together. Networks, however, can be unstable with sparse data and stable estimates have in some cases required the use of Bayesian priors in these models. In particular, we imposed Gaussian priors with mean 0 and non-zero variance on all coefficients except intercepts, to bias the model away from spurious effects driven by data sparsity. These priors were based on expert opinion and can improved with further empirical validation in the future (appendix of Murray et al.[1]).

Section 3.4.6: Exceptions and special handling

There were several notable exceptions and special handling decisions made for each individual pathogen distribution model, which we hope to address with more sustainable approaches in our future work. For example, for cardiac infections, we used the pathogen distribution for bloodstream infections rather than estimating specific distributions for these syndromes, due to a lack of complete literature reviews on the aetiologies and case-fatality rates of these syndromes. We consider this to be a serious limitation of our methodology, but do not anticipate that is seriously impactful on our final estimates.

In diarrhoea patients, cultures of specimens taken from the gastrointestinal tract, bowels, rectum, or stool are almost always affected by contaminants or pathogens that are not the cause of diarrhoea. For this reason, we believe that our input data and modelling framework are not able to accurately capture the aetiologies of diarrhoea. We chose to use GBD estimates of the aetiologies of diarrhoea in deaths instead of running our own model.[14] Nonetheless, a major limitation of using such approach is that the GBD diarrhoea aetiology estimates are population attributable fractions (PAFs) for each pathogen. These PAFs may add to greater than 1 and the authors made no attempt to quantify the extent of co-occurrence of pathogens; the latter is inconsistent with the pathogen distribution estimation method used in our study, which quantifies polymicrobial infections and estimates all pathogens as mono-infections. Hence, in order to avoid duplication of cases in our framework, we had to make some assumptions about the co-occurrence of pathogens in diarrhoea (details provided in the appendix of Murray et al.[1]).

Certain skin and subcutaneous samples are easily affect by contaminants, colonization, and other pathogens that are not the cause of infection. For this reason, we considered microbial data and mortality surveillance to be too difficult to extract meaningful aetiology information from, and instead used only ICD-coded databases (multiple cause of death, hospital discharge, and linkage data) and literature studies as inputs into our model of the pathogen distribution of skin infections.

We dropped all data on *S. pneumoniae* for community-acquired LRI and thorax infections in non-neonatal age groups except our estimates from the vaccine probe analysis. Because dedicated anaerobic cultures were not routinely performed for peritoneal samples, we dropped all anaerobes observed in the data for and excluded anaerobes as an etiology of intra-abdominal infections. Moreover, due to the unique pattern of meningitis in

neonates, particularly the high prevalence of GBS, we modeled neonatal and adult central nervous syndrome infections separately.

For three infectious syndromes, we did not run a pathogen distribution model – these are "Typhoid, paratyphoid, and invasive non-typhoidal *Salmonella*", "Tuberculosis" and "Gonorrhoea and chlamydia" infectious syndromes. They are all caused by distinct pathogens whose individual burdens are already estimated in GBD as separate causes of death. Therefore, for these syndromes, we simply used GBD estimates.

Section 3.4.7: Model validation

To assess model validity, we calculated the root mean square error (RMSE) and coefficient of determination (R^2) for each pathogen distribution model in proportion space for both in-sample and out-of-sample predictions. Proportions were predicted for each observation using the specific denominator observed from that study. For example, if a given study reported on only *E. coli* and *S. pneumoniae*, the predictions for model validation for this study were calculated as proportions of the total for *E. coli* and *S. pneumoniae.* In order to calculate out-of-sample fit, we perform non-exhaustive cross-validation, with each round of the validation holding out 1 country of data at a time. This leave-one-country-out approach simulates the prediction task of estimating the pathogen distribution of a country for which we have no data. As evidenced in the appendix of Murray et al. (2022),[1] it was shown that our models have a good fit and good out-of-sample predictive ability.

Section 3.5: Prevalence of resistance

Section 3.5.1: Input data

We identified line level and aggregate data on the prevalence of resistance in bacterial pathogens, which were linked to the country and year in which the infection was acquired, from datasets obtained from pharmaceutical companies, surveillance networks, academic institutions, and individual hospitals (see section 2). We supplemented microbiological data with systematic reviews following the Preferred Reporting Items for Systematic Reviews and Meta-Analyses (PRISMA) guidelines,[15] to collect resistance data published from countries and territories where surveillance systems do not routinely collect data to ensure extensive coverage of the pathogen–drug combinations thought to contribute the greatest burden of drug resistant infections, which we termed core pathogen–drug combinations (table 3.5.1.1). Data on the prevalence of AMR in these pathogen–drug combinations were extracted from published literature and compiled into comprehensive datasets. The systematic reviews followed similar methodologies; a detailed description can be found either in published literature (*S.* Typhi and *S.* Paratyphi[16]) or in the corresponding PROSPERO records (*E.* coli, *K. pneumoniae, S. aureus* and *S. pneumoniae* PROSPERO registration CRD42019145148; *Shigella* species PROSPERO registration CRD42019127603; iNTS PROSPERO registration CRD42020189935; *N. gonorrhoeae* SPF unique identifier osf.io/4vy5n). The *S.* Typhi and *S.* Paratyphi A systematic review was expanded to include non-blood culture isolates for the current analysis. Forms were created, and screening and data extraction were completed using web-based systematic review software (DistillerSR, Evidence Partners, Ottawa, Canada) for all pathogens except *Salmonella,* for which a smaller number of manuscripts were identified.

For the prevalence of drug resistance in *Mycobacterium tuberculosis* for multi-drug resistance (MDR, characterised by isoniazid and rifampicin co-resistance) excluding extensive drug resistance (XDR, characterised by resistance to isoniazid, rifampicin, and fluoroquinolone, as well as either aminoglycosides or capreomycin) and XDR, we used previously published GBD results.[2] To more comprehensively account for the burden of AMR in bacteria, we also estimated the prevalence of resistance for 71 supplementary pathogen–drug combinations for which we did not conduct a systematic literature review. Data for these supplementary combinations were extracted from the datasets obtained from pharmaceutical companies, academic institutes, and individual hospitals using the same processing procedure as was used for the core pathogen–drug combinations. The list of supplementary combinations is presented in table 3.5.1.2.

Table 3.5.1.1: Core pathogen–drug combinations

Pathogen	Antimicrobial
Escherichia coli	Third-generation cephalosporins<br>Fluoroquinolones

Klebsiella pneumoniae	Third-generation cephalosporins<br>Carbapenems
Staphylococcus aureus	Methicillin
Streptococcus pneumoniae	Penicillin
Salmonella Typhi & Paratyphi A	Multidrug resistance<br>Fluoroquinolones
Invasive non-typhoidal *Salmonella*	Fluoroquinolones
Shigella species	Fluoroquinolones
Neisseria gonorrhoeae	Third-generation cephalosporins
Mycobacterium tuberculosis	Isoniazid mono-resistance, Rifampicin mono-resistance

Table 3.5.1.2: Supplementary pathogen–drug combinations

Pathogen	Antimicrobial
Acinetobacter baumannii	Aminoglycosides, Anti-pseudomonal penicillin/Beta-lactamase inhibitors, Beta-lactam/Beta-lactamase inhibitors, Carbapenems, Third-generation cephalosporins, Fourth-generation cephalosporins, Fluoroquinolones
Citrobacter species	Aminoglycosides, Anti-pseudomonal penicillin/Beta-lactamase inhibitors, Carbapenems, Third-generation cephalosporins, Fourth-generation cephalosporins, Fluoroquinolones
Enterobacter species	Aminoglycosides, Anti-pseudomonal penicillin/Beta-lactamase inhibitors, Carbapenems, Fourth-generation cephalosporins, Fluoroquinolones, Trimethoprim-Sulfamethoxazole
Enterococcus faecalis	Fluoroquinolones, Vancomycin
Enterococcus faecium	Fluoroquinolones, Vancomycin
Enterococcus species	Fluoroquinolones, Vancomycin
Escherichia coli	Aminoglycosides, Aminopenicillin, Beta-lactam/Beta-lactamase inhibitors, Carbapenems, Trimethoprim-Sulfamethoxazole
Group A *Streptococcus*	Macrolide
Group B *Streptococcus*	Fluoroquinolones, Macrolide, Penicillin
Haemophilus influenzae	Aminopenicillin, Third-generation cephalosporins
Klebsiella pneumoniae	Aminoglycosides, Beta-lactam/Beta-lactamase inhibitors, Fluoroquinolones, Trimethoprim-Sulfamethoxazole
Morganella species	Third-generation cephalosporins, Fourth-generation cephalosporins, Fluoroquinolones
Neisseria gonorrhoeae	Fluoroquinolones
Proteus species	Aminoglycosides, Aminopenicillins, Third-generation cephalosporins, Fluoroquinolones, Trimethoprim-Sulfamethoxazole
Pseudomonas aeruginosa	Aminoglycosides, Anti-pseudomonal penicillin/Beta-lactamase inhibitors, Carbapenems, Third-generation cephalosporins, Fourth-generation cephalosporins, Fluoroquinolones
Serratia species	Aminoglycosides, Anti-pseudomonal penicillin/Beta-lactamase inhibitors, Carbapenems, Third-generation cephalosporins, Fourth-generation cephalosporins, Fluoroquinolones
Staphylococcus aureus	Fluoroquinolones, Macrolide, Trimethoprim-Sulfamethoxazole, Vancomycin
Streptococcus pneumoniae	Beta-lactam/Beta-lactamase inhibitors, Carbapenems, Third-generation cephalosporins, Fluoroquinolones, Macrolide, Trimethoprim-Sulfamethoxazole

Group A *Streptococcus* = *Streptococcus pyogenes.* Group B *Streptococcus* = *Streptococcus agalactiae*

Section 3.5.2: Data processing

The prevalence of resistance for each pathogen–drug combination was calculated for each data source, by country and year. Whenever possible, we classified resistance using the most recent CLSI guidelines based on the MICs provided in the data. When MICs were unavailable, we deferred to lab interpretation to classify the isolates. All isolates determined to have intermediate resistance were classified as resistant. To determine the prevalence of resistance to a class of antibiotics (eg, fluoroquinolones), resistance to any one of the antibiotics in the class was sufficient to classify an isolate as resistant for line level data (ie, susceptibility data for individual isolates). For aggregate data (ie, the proportion of isolates resistant to various antibiotics), the highest prevalence of resistance to

any antibiotic in the class was selected. Multidrug resistance in *Salmonella* species was defined as concurrent resistance to ampicillin/amoxicillin, chloramphenicol, and trimethoprim-sulfamethoxazole; and fluoroquinolone resistance was defined as ciprofloxacin minimum inhibitory concentration of 0.125 μg/ml or higher, or nalidixic acid resistance (CLSI breakpoint for *Salmonella* spp. were updated in 2012 to include 0.125 μg/ml as isolates with 'decreased ciprofloxacin susceptibility', and we have considered these as resistant). Nalidixic acid resistance was also used as a proxy for fluoroquinolone non-susceptibility for *Shigella* species.

To account for biased level of resistance found in tertiary care settings, we reviewed all input data used for the prevalence of resistance estimation and classified each data source as either tertiary, non-tertiary, or unknown/mixed designation, which was a commonly used classification for large resistance surveillance networks which don't report on the hospitals they collect data from. We located datasets that either provided facility information at the line-level or reported samples from exclusively tertiary or non-tertiary facilities. Where possible, we used tertiary/non-tertiary assignments from the data providers. When no assignments were available, we classified sites as primary, secondary and following the definitions provided by Jamison et al.,[17] as described in the appendix of Murray et al. (2022).[1]

Because the degree of bias in resistance between tertiary and non-tertiary data could vary, we ran a separate crosswalk for each super region and pathogen–drug *super group* combination. Certain bacteria and antimicrobials were clustered into super groups to provide the models with more robust input data, though, crucially, while a given model would contain several pathogen–drug combinations in its inputs, every matched pair was made comparing tertiary and non-tertiary values for the same combination. Bacteria were classified as follows (excluding those that would be robust to tertiary care bias, as well as *Morganella* spp. due to no input data for that pathogen from tertiary facilities):

Table 3.5.2.1: Pathogens in each pathogen super group

**Pathogen super group**	**Incorporated pathogens**
Gram-positives	*Enterococcus faecalis, Enterococcus faecium, Enterococcus* spp., Group A *Streptococcus*, Group B *Streptococcus, Staphylococcus aureus, Streptococcus pneumoniae*
Enterobacterales	*Citrobacter* spp., *Enterobacter* spp., *Escherichia coli, Haemophilus influenzae, Klebsiella pneumoniae, Proteus* spp., *Serratia* spp.
Pseudomonadales	*Acinetobacter baumannii, Pseudomonas aeruginosa*

Only one group of antimicrobials was clustered to create an antimicrobial super group, the β-lactam group, which was comprised of: aminopenicillin, anti-pseudomonal penicillin, β-lactamase inhibitor combinations, carbapenems, third and fourth generation cephalosporins, methicillin, and penicillin. All other antibiotic classes (aminoglycosides, fluoroquinolones, macrolides, sulfanoamides, and vancomycin) each individually comprised their own antimicrobial super group.

To allow us to implement linear models, resistance values were logit-transformed. We used the delta method to compute the standard error of the prevalence of resistance in logit space. To incorporate data with zero resistance, or with complete resistance, we applied a 0.1% offset, such that the prevalence of resistance for data with zero resistance was represented as 0.1% and the prevalence of resistance for data with total resistance was represented as 99.9%. We then used the MR-BRT modelling framework to estimate the logit difference of tertiary and non-tertiary data for each super region-pathogen/antimicrobial 'super combination', including a random effect for each pathogen–drug combination within the super combination and employing a positivity prior to enforce the constraint that the tertiary data exceed or be equal to the non-tertiary data.

After modelling the difference between tertiary and non-tertiary data, we implemented the models to adjust all the country-level tertiary input data that was indicated as biased. We then used the adjusted prevalence of resistance estimates from tertiary care facilities and unadjusted prevalence of resistance from non-tertiary/mixed care facilities as data inputs for the prevalence of resistance models. As was done before, resistance values were offset prior to logit-transformation to allow the use of linear models; data with zero resistance or complete resistance was offset by 2%. Exceptions to this offset were made for two combinations, *Staphylococcus aureus*/vancomycin and Group B *Streptococcus*/penicillin, which were anticipated to often have values beneath 2% resistance. For these combinations, we applied a 0.5% offset instead.

Section 3.5.3: Modelling framework

The prevalence of AMR in each pathogen–drug combination was modelled separately. For the core combinations, excluding *N. gonorrhoeae*/3GC, we selected a range of spatially- and temporally-explicit health and socio-demographic-related covariates with biologically plausible associations to the prevalence of AMR in each pathogen from the Global Health Data Exchange (http://ghdx.healthdata.org/), and from published literature.[18] This list was narrowed down by fitting a lasso penalised regression model between the data and the covariates for each dataset (using the 'glmnet' package version 3.0.2 in R version 3.6.1) and selecting the most influential covariates in each of the pathogen–drug models to be taken forward. For the supplementary pathogen–drug combinations and *N. gonorrhoeae*/3GC, we utilised a standard set of covariates for all models: HAQ Index, pigs per capita (as a proxy for antibiotic use in animal husbandry), mean temperature, and antibiotic consumption of the antibiotic class relevant to each pathogen–drug combination. Determining more individualised sets of covariates for each of these supplementary pathogen–drug combinations is an ongoing focus for future extensions of this research. All of the covariates used in our models are available in the appendix of Murray et al. (2022).[1]

Due to the high heterogeneity of the input datasets, we classified data points found to have the most extreme values for the prevalence of resistance as outliers. An initial generalised linear model (GLM) was fit to the data and covariates and input data points that lay outside of two times the median absolute deviation from the modelled estimate for each location were determined to be outliers and removed. The GLM was fit with nested random effects based on the location to capture spatial effects and was fit using the 'lme4' package version 1.1-21 in R version 3.6.1.

After the removal of extreme values, the datasets were used to fit spatiotemporal statistical models of the prevalence of AMR. Firstly, we used a stacked ensemble model to fit the associations between selected covariates and data. For each of the pathogen–drug combinations, we considered the following child models for inclusion: generalised additive models (GAM), penalised regression models (elastic-net, ridge, lasso), random forest, cubist, and neural-networks. Models were fit in R version 3.6.1, using the packages 'CARET' version 6.085, 'mgcv' version 1.8.31, and 'glmnet' version 3.0.2. We fit the child models using five-fold cross validation for each combination and selected the best performing, non-correlated child models based on the out-of-sample predictive performance (final covariates for each pathogen–drug combination are shown in table S8). We then calculated the R^2-weighted mean of the estimates of the child models, constraining the coefficients to sum to one, and used these ensemble estimates to fit a spatiotemporal Gaussian process regression (ST-GPR) model for each pathogen–drug combination.

ST-GPR is described in detail elsewhere.[1,2] In brief, spatial and temporal weights were applied to the residuals of the stacked ensemble model; these were then added to the modelled estimates to smooth them in time and space. A Gaussian process regression (GPR) was then fit, and the mean prevalence of AMR was calculated from 1000 draws of the GPR for each location and year with endemic disease. The 1000 draws of the model were taken through to the next stage of calculations to propagate uncertainty throughout.

Section 3.5.4: Resistance profiles

To accurately assess the burden associated with resistance to each antibiotic, we needed to first understand the landscape of multidrug-resistant bacteria, for which the burden would be shared across several antibiotics. We therefore estimated, for each bacteria studied, a set of 'resistance profiles' characterised as the probabilities for each possible combination of resistance/susceptibility for all of the antibiotics analyzed. For example, for a bacterium for which we assessed three antibiotics, we would estimate eight probabilities: SSS, SSR, SRS, RSS, SRR, RSR, RRS, and RRR (S – susceptible, R – resistant). These probabilities encompass the entire set of possibilities of resistance for the bacterium, and sum to 1.

For a pathogen for which we assessed *n* antibiotics, resistance profiles were estimated by optimising over a 2^n - *1*-dimensional probability simplex with $\frac{n(n+1)}{2}$ linear constraints. Every such set of resistance profiles corresponds to a full specification of a multivariate binomial distribution. The target set of constraints were as follows:

- The inferred marginal probability of resistance for each antibiotic (the prevalence of resistance to an antibiotic irrespective of all others analyzed) exactly matches the estimates from our prevalence of resistance models. Since there are *n* antibiotics, this set comprises *n* constraints.
- The inferred pairwise likelihood of co-resistance for each pair of antibiotics exactly matches the likelihood inferred from the marginal probability of each antibiotic in the pair, and the Pearson correlation of resistance between the two antibiotics observed across all of the laboratory data we compiled. These represent $\frac{n^2-n}{2}$ additional constraints.

The input format for these constraints with an example case can be found in the appendix of Murray et al. (2022).[1] However, there is no *a priori* guarantee that the observables generate a feasible solution. To prevent the constraints from delineating an infeasible probability simplex (for example, an input suggesting the individual resistances to antibiotics A and B are both above 90% but the probability of co-resistance to A and B is below 10%), we solved an optimization problem that identified, for each input matrix, the closest feasible set of input constraints and a corresponding set of resistance profiles that fits these constraints. The 1-simplex in any dimension is specified by

$$\Delta \coloneqq \{p: \quad 0 \le p_i \le 1,\ \sum p_i = 1\} \qquad (3.5.5.1)$$

Each marginal observation and each pairwise co-resistance corresponds to a linear constraint, where a sum over a subset of the p in the simplex should be a given value v_i:

$$\mathrm{m}_\mathrm{i}^\mathrm{T} p = v_i \qquad (3.5.5.2)$$

where m_i is a ‘mask vector’ of zeros and ones, used to pick out the appropriate summands. Overall, there are $\frac{n(n+1)}{2}$ such affine constraints. The optimisation problem we solve is to find the nearest feasible simplex given these constraints:

$$\min_{p \in \Delta} f(p) \coloneqq \sum_{i=1}^{n(n+1)/2} \frac{1}{\sigma_i^2} (\, m_i^T p - v_i)\text{^}2 \qquad (3.5.5.3)$$

Where $\frac{1}{\sigma_i^2}$ can be used to provide importance weights for the data. This is a least squares problem with linear equality and inequality constraints (corresponding to the simplex) and can be solved very efficiently even for relatively large n (such as 10 co-occurring antibiotic classes). The result is guaranteed to return the probability simplex closest to the specified constraint, even if the original set of constraints is infeasible, and corresponding set of resistance profiles that fits this nearest simplex.

To propagate uncertainty, we repeat this procedure for each of the 1,000 draws we estimate for prevalence of antibiotic resistance. To generate the *i*-th draw of our resistance profiles, we input the *i*-th draw of the marginal probability of resistance for each antibiotic analyzed for a given pathogen into the probability simplex optimization algorithm. Updating the marginal probabilities of resistance in turn influences the probabilities of co-resistance, and each element of the input we feed the algorithm is unique to the *i*-th draw. The optimization is also initialised randomly for every draw. This process is implemented for each country, resulting in 1000 resistance profiles for each country for each pathogen in our analysis. The Pearson correlations of co-resistance that we derive from the input data are assumed to be constant across location, sex, and infectious syndrome.

Section 3.5.5: Model validation

Validation of prevalence of resistance modelling occurs in two instances. For the ensemble estimates, machine-learning candidate models are validated using five random holdout sets, and we select models correlated below a Pearson correlation coefficient threshold of 0.8 which showed the best performance based on the R^2 predictive validity for the out-of-sample predictions. These intermediary results are not reported in this paper because they do not pertain to the final prevalence of resistance estimate.

We then validate the entire ensemble ST-GPR process by calculating in-sample and out-of-sample accuracy metrics. Accuracy is measured as the proportion of correctly classified resistant/susceptible isolates based on the modelled estimate and the raw data's prevalence of resistance. As a written example, if there were 10 isolates with 50% resistance in the raw data and the model predicted 60% resistance for that location, we would have 5 correctly classified resistant samples (true positives), 1 incorrectly classified resistant sample (false positive), and 4 correctly classified susceptible samples (true negatives), for 90% accuracy. For out-of-sample cross-validation, we withheld, at the outset of the ensemble modelling process, a set of countries with data as a holdout group: for the core-combinations we withheld 20% of countries each iteration, for 5 total holdout sets, while for the supplementary-combinations we withheld 10% of countries each iteration, for 10 holdout sets. By holding out all of the data for a set of countries, our out-of-sample accuracy metrics reflect the potential model fit we have for countries that have no input data in the entire prevalence of resistance process. The detailed reports on the accuracy metric for each pathogen–drug combination can be found in the appendix of Murray et al. (2022).[1]

Section 3.6: Relative risk

Section 3.6.1: Input data and data processing

The input data for the relative risk estimation step included literature data that provided relative risk of death for resistant and susceptible organisms and hospital-based microbiology surveillance data linked to outcomes, as well as other clinical parameters (eg, demographics, diagnoses). Published studies were identified from a recent meta-analysis performed by Cassini and colleagues.[18]

The data inputs for the excess duration estimates were literature data that reported on length of stay for resistant and susceptible organisms and hospital-based microbiology surveillance data that were linked to outcomes as well as various other clinical parameters (eg, demographics, diagnoses). The number of days between a positive specimen date and discharge date was used to obtain the mean duration of infection. We took into account days elapsed between admission and discharge as mean duration of stay if this was the only piece of information provided in the study. We also considered median duration of infection or median duration of stay if the study only provided this piece of information.

Relative risk estimates were extracted from primary literature as were study characteristics that described the adjustments made by the study. When no adjustments were made, or an adjusted odds ratio was presented, we extracted the crude relative risk. For hospital data that contained admission diagnoses, diagnoses were mapped to GBD Level 2 causes. Admission diagnoses were mapped to GBD causes using ICD codes when provided; when admission diagnoses were free-text entries, they were mapped using two expert reviews.

Section 3.6.2: Modelling overview

The measure of excess risk used to estimate the fatal burden of AMR was the relative risk of death from an infection with a pathogen resistant to the antibiotic of interest as compared to an infection of the same site with the same organism that was susceptible to the antibiotic of interest. The relative risk estimate was produced after adjusting for various potential confounders including age, admission diagnosis (mapped to GBD causes), site of culture, and hospital versus community onset. Because of data sparsity, a single measure of relative risk was estimated for each pathogen–drug combination, representing a global estimate for all sites of infection and all underlying causes.

When data availability allowed it, relative risk from hospital-based microbiology surveillance data was estimated after adjusting for age, admission diagnosis, site of culture, and hospital- versus community-acquired infection, otherwise a crude relative risk was used. The adjusted estimates of relative risks were then included with the crude relative risks in a two-stage nested mixed effects meta-regression model using MR-BRT. The stage one model was a meta-regression for each antibiotic class, which was used to produce a prior for the stage two model. We considered study-specific adjustments such as age of patients, admission diagnosis, site of culture and hospital-versus community acquired infection as potential covariates to be included in the second stage. Covariate selection was based on a set of log-linear models with a range of Lasso penalty parameters, and only statistically significant covariates were selected. The stage two model was run for each antibiotic class with a random effect for pathogen and fixed effects for study level characteristics that described whether the relative risk estimate from a study or dataset adjusted for each parameter using the prior from the stage one model for the antibiotic class.

$$Relative\ Risk_{pathogen_n drug_d} = \beta_0 + \beta_d \cdot x + u_{pathogen_n} + \epsilon_d \quad (3.6.2.1)$$

Where x is a bias covariate, $u_{pathogen_n}$ is a random effect for pathogen *n* within an antibiotic class, ϵ_K is the measurement error, d is antibiotic class and β and X are vectors of length $i + 1$ for i covariates. From this stage two model, we produced 1000 draws to estimate the relative risk of death and uncertainty attributable to resistance for each pathogen–drug combination.

For non-fatal burden estimation, we estimated the excess duration attributable to resistance – comparing the length of hospital stay for an infection with a pathogen resistant to the antibiotic of interest to an infection of the same site with the same organism that was susceptible to the antibiotic of interest. For community-acquired infections the entire duration of length of stay was attributed to the infection, whereas for hospital-acquired infections we used the time from first positive culture to time of discharge to estimate length of stay. To address the potential confounding effect of longer admissions resulting in higher probability of acquiring resistant infections, we adjusted the relative length of stay obtained from patient level data for the number of hospital days prior to culture positivity. We observed a generally lower relative length of stay when we applied this adjustment, which was expected. We then used the same two-stage nested mixed effects meta-regression modelling framework described for fatal estimation to produce a relative length of stay attributable to resistance for each pathogen–drug combination. One exception to this estimation process was *Neisseria gonorrhoeae*, which had too little data to produce an estimate on the impact of resistance on duration of illness. As a result, we produced a YLD estimate based on the excess duration of illness for a given antibiotic class.

The analysis of relative risk followed the definitions of the prevalence of resistance step (section 3.5) as closely as possible. Both analyses identified resistance to a given antibiotics class if the isolate had an intermediate or resistant interpretation to any one of the antibiotics in that given class. But the analysis of relative risk diverged from the analysis of prevalence of resistance in the following circumstances. First, the relative risk step included molecular resistance testing if this was the only data provided by a study, eg, β-lactamase or *mecA* positive pathogens; this could potentially misclassify some resistant organisms as sensitive if they had an alternate mechanism for resistance, such as a porin alteration leading to carbapenem resistance. Second, the relative risk estimate produced was for sterile sites of infection, as there was limited data from non-sterile sites. Third, it was not possible to assess relative risk of multidrug-resistant pathogens because of limited data availability and because it did not fit in the modelling strategy at the antibiotic class level. Instead, the relative risk of each of the components of multidrug-resistant pathogens was calculated and the antibiotic class with the highest relative risk was used; for *Salmonella* Typhi this was relative risk to Trimethoprim-Sulfamethoxazole. Fourth, we had limited availability of data on fatalities attributable to *Salmonella* Paratyphi and *Shigella* species; as a result, we used fatal relative risk estimates from *Salmonella* Typhi as a proxy. Fifth, there were limited data on fatalities attributable to resistant *N. gonorrhoeae*, so we excluded the fatal estimate for this pathogen. Finally, the relative risk of *Mycobacterium tuberculosis* was assessed for multidrug and extensively drug-resistant infections as reported previously in GBD. Estimates of relative risk of death for sterile sources of specimen across 88 pathogen–drug combinations can be viewed in the appendix of Murray et al. (2022).[1]

Section 3.6.3: Model validation

We report three summary metrics to evaluate the relative risk of death models: the root-mean squared error (RMSE), the Mean Average Error (MAE) and the percent coverage of observed data within the full variance of the model. These three metrics were calculated using the real relative risk ratio in the whole sample of data and also by holding out 25% of the sample within antibiotic class in 4 iterations. The details on in-sample and out-of-sample performance metrics for relative risk of death models can be seen in the appendix of Murray et al. (2022).[1]

This approach for relative risk estimation had several limitations, most were attributable to data sparsity. First, it is likely that the impact of resistance on mortality is different across locations. In locations where overall health-care access and quality are lower, the impact of resistance may be smaller because the management of susceptible infections is sub-optimal. Conversely, in locations where broad, second- and third-line antimicrobials are not available, one would expect the impact of resistance to be greater. Second, it is possible that the relative risk of death

attributable to resistance is different across anatomical sites of infection because of variable penetrance of antibiotics to different anatomical locations. As we continue efforts to expand data collection and reporting, we hope to be able to address these limitations in future iterations.

Section 3.7: Counterfactuals and AMR estimation

Section 3.7.1: Estimating AMR burden with counterfactual of no infection

We computed two counterfactuals to estimate the drug-resistant burden. First, we estimated the burden of AMR using the counterfactual of no infection. We estimated the fatal burden of individual pathogen–drug combinations by taking the product of the deaths for each underlying cause, fraction of deaths related to infection, infectious syndrome fraction, fatal pathogen fraction, and fatal prevalence of resistance and then summed across all infectious syndromes and underlying causes:

$$Deaths\ with\ Resistance_{Kd} = \sum_J \sum_L D_J \times S_J \times M_{LJ} \times P_{LK} \times R_{Kd} \quad (3.7.1.1)$$

where D = deaths, S = fraction related to infection, M = infectious syndrome fraction, P = fatal pathogen fraction, R = fatal prevalence of resistance, J = cause, L = syndrome, K = pathogen, d = drug. To produce an estimate of deaths with resistance to any antibiotic estimated, we employed the same formula but used the fatal prevalence of resistance to any antibiotic using the resistance profiles, described previously. We calculated the fatal prevalence of resistance R for a given drug d based on the non-fatal prevalence of resistance R' and relative risk of death RR for this drug:

$$R_{Kd} = \frac{R'_{Kd} RR_{Kd}}{(1 - R'_{kd}) + R'_{Kd} RR_{Kd}} \quad (3.7.1.2)$$

We calculated the fatal prevalence of resistance to any antibiotic estimated based on the non-fatal prevalences of each resistance profile, incorporating all resistance profiles δ that are resistant to at least 1 drug with corresponding relative risks RR_{Kd^*}, determined by the method described below (section 3.7.2):

$$R_{K,all\ drugs} = \frac{\sum_\delta R'_{K\delta} RR_{Kd^*}}{\left(1 - \sum_\delta R'_{K\delta}\right) + \sum_\delta R'_{K\delta} RR_{Kd^*}} \quad (3.7.1.3)$$

We then estimated YLLs using standard GBD methods to convert age-sex specific deaths into YLLs.[3]

For the non-fatal estimate, we first estimated the incidence of each infectious syndrome in each underlying cause. For infectious underlying causes, we simply used the incidence estimated in GBD. For non-infectious underlying causes, we divided the infectious syndrome deaths ($D_j \times S_J \times M_{LJ}$) by the syndrome- and pathogen-specific CFRs calculated in section 5, aggregated across pathogen using the nonfatal pathogen distribution P' calculated above.

$$Incidence_{JL} = \frac{D_J S_J M_{LJ}}{\sum_K CFR_{LK} P'_{LK}} \quad (3.7.1.4)$$

We then took the product of the infectious syndrome incidence, the non-fatal pathogen fraction, and the non-fatal prevalence of resistance and summed across all infectious syndromes and underlying causes to get incidence with resistance for every pathogen and drug. As with the fatal estimate, to produce an estimate of incident infections with resistance to any antibiotic, we used the same formula and used the non-fatal prevalence of resistance to any antibiotic estimated from the resistance profiles.

We then calculated YLDs for each pathogen. For some GBD causes, we simply used the GBD YLD estimates and multiplied them by the corresponding nonfatal pathogen distribution (table 8.1.2) For all other causes, we multiplied together the infectious syndrome incidence, the non-fatal pathogen fraction, and a syndrome-specific YLDs per incident case rate, calculated using a proxy cause from GBD.[3] To estimate the YLDs per incident case rate, we extracted GBD incidence and YLD estimates for the proxy causes and divided the YLDs by the incidence for each age, sex, and location. Three infectious syndromes are not estimated in the GBD, and therefore have no standard

sequelae or disability weights: bloodstream infections, intra-abdominal infections, and bone and joint infections. For the proxy causes for these three syndromes, we used the closest approximate disease as determined by a group of experts in infectious diseases and epidemiology. This approach is a significant limitation of the study and should be improved in future work.

To get the YLDs associated with resistance for each pathogen, we used the non-fatal prevalences of resistance for each drug and resistance profile and relative length of stay (LOS) for each pathogen–drug combination to calculate the fraction of YLDs associated with resistance for each pathogen, using equations analogous to equations 3.7.1.2 and 3.7.1.3. We multiplied this fraction by the YLDs for each pathogen to get YLDs associated with resistance to each pathogen–drug combination and YLDs associated with resistance any antibiotics estimated. We then added YLLs and YLDs to produce the DALY estimate for burden associated with resistance.

Section 3.7.2: Estimating AMR burden with counterfactual of infection with susceptible organism

For the second counterfactual – comparing resistant to susceptible infections – we calculated mutually exclusive pathogen–drug estimates. To do this, we first estimated the population attributable fraction of deaths ($Mortality\ PAF$) for each resistance profile with resistance to at least 1 drug, δ. The inputs for the PAF were the non-fatal prevalence of the given resistance profile, $R'_{K\delta}$, and the relative risk of death for resistant infection compared to susceptible infection for each drug, RR_{kd}. Because of data sparsity, we were unable to calculate the relative risk for every possible resistance profile, and so instead used the highest relative risk of all of the drugs in the resistance profile. For example, if for a resistance profile of resistant to penicillin and fluoroquinolones, the relative risk was 1.1 for penicillin and 1.4 for fluoroquinolones, we would use a relative risk of 1.4 for this profile. The mortality PAF is calculated as a multi-category exposure:

$$Mortality\ PAF_{K\delta} = \frac{R'_{K\delta}(RR_{Kd^*} - 1)}{1 + \sum_{\delta} R'_{K\delta}(RR_{Kd^*} - 1)} \qquad (3.7.2.1)$$

where d* is the drug in the resistance profile δ with the highest relative risk.

We then took the product of the deaths for each underlying cause, fraction of deaths related to infection, infectious syndrome fraction, fatal pathogen fraction, and the mortality PAF for each resistance profile to get the deaths attributable to resistance for every resistance profile:

$$Deaths\ due\ to\ Resistance_{K\delta} = \sum_{J}\sum_{L} D_J \times S_J \times M_{LJ} \times P_{LK} \times Mortality\ PAF_{K\delta} \qquad (3.7.2.2)$$

When the resistance profile described resistance to more than one antibiotic, the deaths were then distributed to the component pathogen–drug combinations based on the excess risk of the pathogen–drug combination divided by the sum of the excess risk of all pathogen–drug combinations in the resistance profile. For a resistance profile δ with resistance to drugs $i = 1, \ldots, n$:

$$Redistribution\ Weight_{Kd_i} = \frac{RR_{Kd_i} - 1}{\sum_{\hat{i}}(RR_{Kd_i} - 1)} \qquad (3.7.2.3)$$

For co-resistance amongst beta-lactam antibiotics (ie, carbapenems, 4GC, 3GC, antipseudomonal, BL/BLI, aminopenicillins, and penicillin), we used a different approach to redistributing burden. Similar to Cassini et al., we applied a hierarchy such that the burden was categorically attributed to the broadest beta-lactam antibiotic, rather than split the burden between multiple beta-lactam antibiotics.[4] When a pathogen was resistant to multiple beta-lactams and a non-beta-lactam antibiotic, we first applied the hierarchy to determine the ‘highest’ beta-lactam resistance and then generated redistribution weights using only the ‘highest’ beta-lactam and the non-beta-lactams. We then used these attributable death estimates to estimate YLLs using standard GBD methods to convert age-sex specific deaths to YLLs.

A similar approach was taken to estimate non-fatal burden for the counterfactual of antibiotic-susceptible infection. We first assumed that antibiotic resistance has no effect on the attack rate of pathogens; therefore, there are 0

incident cases attributable to resistance and all non-fatal burden comes from increased length of illness. To quantify the extent of this increased length of illness, we first produced a length of stay (LOS) PAF for each resistance profile using the non-fatal prevalence of resistance and relative LOS for resistant infections as compared to susceptible infections in a method analogous to equation 3.7.2.1. Because of data sparsity, we were unable to calculate the relative LOS for every resistance profile, and so instead used the relative LOS for the drug with the highest relative LOS in the profile. We then took the product of the YLDs for each infectious syndrome, the non-fatal pathogen distribution, and the LOS PAF to produce attributable YLD estimates. This assumes that the attributable LOS PAF is equally applicable to all sequelae, which is an assumption made because of a lack of data on the impact of resistance on the likelihood of different sequelae and the duration of specific sequelae. Specifically for AMR, this assumption fails to account for the fact that patients with resistant infections are more prone to re-infection, treatment failure and long term sequalae as compared to patients with susceptible ones, and we acknowledge this is a significant limitation that should be improved in future work. We then added YLLs and YLDs to produce an estimate of DALYs attributable to resistance.

Because of the optimisation approach used to derive each resistance profile, the prevalence of resistance to for a given pathogen–drug as modelled using ensemble ST-GPR (section 3.5.3), R'_{Kd}, will not necessarily be exactly equal to the sum of all resistance profiles $R'_{K\delta}$ that include resistance to drug d. Due to this inconsistency, in extremely rare cases, an estimate of AMR burden in the susceptible counterfactual may slightly exceed the corresponding estimate of AMR burden in the no infection counterfactual for a specific pathogen–drug. We consider the ensemble ST-GPR estimate to be more accurate than the resistance profiles, since the latter are based on Pearson correlations of multidrug resistance that are calculated from limited microdata and generalised to all locations. For this reason, we cap all individual pathogen–drug estimates of burden for the susceptible counterfactual, which are based on the resistance profiles, to the burden for the no infection counterfactual, which are based on the ensemble ST-GPR estimates.

Section 3.7.3: Excluded combinations

Although our approach attempted to be exhaustive and include all clinically-relevant pathogen–drug combinations, there are two combinations included in the WHO priority list for which we could not produce an estimate. The first is clarithromycin resistance in *Helicobacter pylori* and the second is fluoroquinolone resistance in *Campylobacter* species. These were excluded due to limited data availability, as highlighted by a recent study in the European Union that found that, as of 2019, no member countries had implemented publicly accessible, mandatory reporting surveillance programmes for these two pathogen–drug combinations.[19] *H. pylori* and *Campylobacter* spp. are commonly diagnosed without culture so resistance profiles are uncommon in passive surveillance systems. The burden of *H. pylori* is not currently estimated in GBD, though some of the consequent diseases are, like peptic ulcer disease and gastric cancer. Producing a burden estimate of *H. pylori was* outside the scope of this work, and without a pathogen burden estimate, we could not produce an estimate of the burden attributable to clarithromycin-resistant *H. pylori*. In contrast, GBD does produce an estimate on the burden of *Campylobacter* spp. There were, however, too few data to produce an estimate on the excess risk of death or duration associated with fluoroquinolone resistance and limited data to inform a global prevalence of resistance estimate. Given these limitations, we did not produce burden estimates for clarithromycin-resistant *H. pylori* or fluoroquinolone-resistant *Campylobacter* spp. Because of the lack of data on risk of death associated with drug-resistant *Neisseria gonorrhoeae*, we were unable to produce an estimate of the fatal burden of resistance so produce only a non-fatal estimate. Many potential pathogen–drug combinations were excluded due to the spectrum of antimicrobial activity (ie, vancomycin and *E. coli*), intrinsic resistance (eg, BL/BLI resistance in *Pseudomonas aeruginosa*) or resistance that is exceedingly common (eg, penicillin resistance in *S. aureus*); these combinations were decided by a group of experts in infectious diseases, microbiology, epidemiology, and population health. A final constraint was the computational burden of estimating more than seven antibiotic classes for a single pathogen. Because of the approach to co-resistance described in section 3.5, each antibiotic class added led to an exponential increase in the computation needs and anything above seven antibiotic classes was not tenable. As additional data are made available, we plan to add clinically relevant combinations and iterate on the computational approach so that we can describe the burden of bacterial AMR more comprehensively.

Section 3.8: Correlation Analysis

Section 3.8.1: Input Data

We calculated the correlation between our estimates of AMR burden and the following variables: socio-demographic index (SDI), access to safe water, sanitation, and hygiene (WaSH), antibiotic consumption, and AMR national action plan (NAP) status. The included data sources for each are listed below:

- Socio-demographic Index (SDI)
  - **Global Burden of Disease:** A covariate produced as part of the Global Burden of Disease project which incorporates total fertility rate under the age of 25, mean education of those ages 15 and older, and lag distributed income per capita.[20]
- Access to safe water, sanitation, and hygiene (WaSH)
  - **Global Burden of Disease:** A covariate produced as part of the Global Burden of Disease project which incorporates variables such as access to a handwashing station with soap and water, primary drinking water source, and type of water treatment used.[3]
- Antibiotic consumption

  These data were used to model the antibiotic use covariate,[21] which was used as an input in the prevalence of resistance models and as part of our correlation analysis.

  - **IMS Health and Quintiles (IQVIA)**: antibiotic sales data for 77 countries between 2000 and 2018.
  - **Demographic Health Surveys (DH)**: households health surveys carried out across more than 90 countries, they include questions on antibiotic usage among those who had cough or diarrhoea in a period of two weeks before the survey.
  - **Multiple Indicators Cluster Surveys (MICS)**: households health surveys carried out across more than 90 countries, they include questions on antibiotic usage among those who had cough or diarrhoea in a period of two weeks before the survey.
  - **European Surveillance of Antimicrobial Consumption Network (ESAC-NET)**: antibiotic consumption data for 5 countries over 101 country-years.
  - **WHO report on surveillance of antibiotic consumption**: 2016-2018 early implementation: report on antibiotic consumption for 21 countries over 21 country-years.
- AMR national action plan status
  - **Global Database for Tracking Antimicrobial Resistance (AMR) Country Self-Assessment Survey (TrACSS)**: country self-assessment surveys carried out by 166 countries on their progress tracking AMR as it relates to human health, animal health, food, agricultural, and environment sectors from 2017-2022.[22]

Section 3.8.2: Correlation Method

To correlate both associated and attributable AMR burden to the 4 variables mentioned above (SDI, WaSH, NAPs, antibiotic consumption), we utilized the Pearson correlation coefficient of the form:

$$r = \frac{\sum(x_i - \bar{x})(y_i - \bar{y})}{\sqrt{\sum(x_i - \bar{x})^2(y_i - \bar{y})^2}}$$

Where r is the Pearson correlation coefficient, x_i are the values of the given covariate, $\bar{x}$ is the mean value of the given covariate, y_i are the estimates of associated or attributable AMR burden (crude mortality rate per 100,000), and $\bar{y}$ is the mean estimate of associated or attributable AMR burden.

We calculated uncertainty at the 95% confidence interval using the 2.5th and 97.5th ordered values across 1000 posterior draws.

Section 4: Supplementary Tables and Figures

Supplementary Table 1. Deaths attributable to and associated with antimicrobial resistance (expressed as counts and age-standardised rates (ASMR) per 100 000 with 95% uncertainty intervals) per country in the WHO African region.

Country	Deaths associated with AMR (counts)	Deaths associated with AMR (age-standardised rate per 100,000)	Deaths attributable to AMR (counts)	Deaths attributable to AMR (age-standardised rate per 100,000)
Algeria	14,000 (20,000 - 8,790)	48.9 (72.4 - 31.9)	3,410 (5,180 - 2,180)	12.2 (18.5 - 7.9)
Angola	23,000 (30,000 - 16,000)	131.6 (177.5 - 97.9)	5,350 (7,550 - 3,770)	31.8 (45.1 - 22.5)
Central African Republic	9,480 (13,000 - 6,920)	251.3 (339.8 - 181.2)	2,240 (3,210 - 1,520)	60.2 (88.8 - 40.2)
Congo	3,620 (4,880 - 2,630)	134.4 (180.7 - 97.8)	890 (1,250 - 620)	33.3 (46.7 - 23.9)
Democratic Republic of the	76,000 (98,000 - 57,000)	158.5 (208.4 - 117.5)	18,000 (26,000 - 13,000)	39.1 (56.0 - 27.5)
Equatorial Guinea	620 (910 - 420)	97.9 (139.7 - 66.9)	150 (220 - 100)	23.9 (34.9 - 15.9)
Gabon	1,020 (1,410 - 740)	99.2 (136.4 - 71.9)	260 (380 - 180)	25.0 (35.8 - 17.5)
Burundi	11,000 (15,000 - 8,340)	167.0 (221.4 - 124.1)	2,750 (4,150 - 1,910)	41.9 (65.1 - 28.5)
Comoros	640 (880 - 480)	124.8 (169.3 - 92.4)	170 (250 - 110)	32.3 (50.0 - 21.5)
Eritrea	6,610 (9,350 - 4,690)	203.8 (281.8 - 145.5)	1,610 (2,490 - 1,060)	50.2 (77.0 - 33.6)
Ethiopia	85,000 (109,000 - 66,000)	138.1 (175.9 - 107.9)	21,000 (28,000 - 16,000)	35.3 (46.5 - 26.7)
Kenya	37,000 (47,000 - 29,000)	148.8 (188.8 - 116.9)	8,540 (11,000 - 6,570)	34.5 (45.0 - 26.4)
Madagascar	23,000 (30,000 - 17,000)	155.4 (207.3 - 112.6)	5,390 (7,400 - 3,800)	37.1 (52.7 - 25.7)
Malawi	16,000 (20,000 - 12,000)	152.7 (196.3 - 117.6)	3,610 (4,770 - 2,680)	36.0 (47.3 - 26.6)
Mauritius	770 (1,150 - 480)	49.9 (74.3 - 31.3)	200 (310 - 120)	13.1 (20.0 - 8.0)
Mozambique	31,000 (41,000 - 24,000)	177.9 (235.8 - 134.1)	7,830 (11,000 - 5,430)	47.1 (70.3 - 31.8)
Rwanda	9,760 (13,000 - 7,430)	140.7 (182.7 - 108.1)	2,370 (3,200 - 1,750)	34.7 (47.2 - 25.5)
Seychelles	80 (100 - 60)	77.0 (101.0 - 58.4)	20 (30 - 10)	19.3 (25.9 - 14.1)
Tanzania	54,000 (70,000 - 41,000)	144.2 (184.1 - 111.6)	13,000 (17,000 - 9,370)	34.5 (46.5 - 25.3)
Uganda	31,000 (40,000 - 23,000)	129.5 (166.6 - 98.4)	7,110 (9,660 - 5,070)	30.8 (42.7 - 21.9)
Zambia	16,000 (21,000 - 12,000)	164.7 (218.8 - 121.7)	3,670 (5,080 - 2,600)	39.9 (55.4 - 28.1)
Botswana	1,790 (2,490 - 1,280)	126.7 (176.6 - 91.3)	420 (610 - 280)	29.2 (41.9 - 19.7)
Lesotho	2,850 (3,880 - 2,050)	212.5 (292.4 - 152.8)	720 (1,110 - 470)	53.5 (80.6 - 35.2)
Namibia	1,880 (2,550 - 1,370)	124.9 (168.7 - 92.4)	450 (640 - 310)	29.9 (42.0 - 20.6)
South Africa	39,000 (50,000 - 30,000)	91.4 (117.7 - 70.0)	9,490 (13,000 - 7,130)	22.2 (29.6 - 16.6)
Eswatini	1,120 (1,540 - 790)	170.4 (235.5 - 118.8)	300 (470 - 180)	44.3 (67.5 - 27.6)
Zimbabwe	16,000 (20,000 - 12,000)	181.5 (239.1 - 137.8)	3,880 (5,840 - 2,700)	45.2 (68.6 - 31.3)
Benin	14,000 (19,000 - 10,000)	154.5 (206.3 - 114.8)	3,120 (4,320 - 2,200)	35.5 (48.2 - 25.5)
Burkina Faso	30,000 (39,000 - 23,000)	172.5 (219.4 - 134.8)	6,960 (9,160 - 5,100)	40.5 (53.5 - 30.5)
Cameroon	25,000 (32,000 - 18,000)	143.7 (191.8 - 107.3)	5,590 (7,520 - 4,010)	33.5 (45.8 - 24.2)
Cape Verde	320 (430 - 230)	74.0 (99.9 - 53.8)	80 (110 - 60)	18.2 (24.9 - 13.0)
Chad	25,000 (32,000 - 19,000)	170.6 (219.3 - 131.6)	5,820 (7,680 - 4,330)	41.5 (55.6 - 29.7)
Cote d'Ivoire	25,000 (32,000 - 18,000)	149.8 (195.4 - 114.7)	5,790 (7,810 - 4,160)	35.9 (48.3 - 26.2)
The Gambia	1,840 (2,380 - 1,390)	153.2 (198.0 - 116.3)	400 (540 - 300)	34.1 (45.9 - 24.7)
Ghana	25,000 (33,000 - 19,000)	137.8 (179.7 - 106.5)	5,920 (8,090 - 4,280)	32.8 (44.3 - 24.2)
Guinea	17,000 (23,000 - 13,000)	171.6 (225.9 - 126.3)	4,000 (5,560 - 2,840)	40.8 (56.3 - 29.1)
Guinea-Bissau	1,820 (2,370 - 1,370)	180.2 (235.2 - 135.5)	410 (560 - 300)	41.5 (56.5 - 29.9)
Liberia	3,420 (4,550 - 2,540)	128.3 (171.9 - 95.8)	770 (1,060 - 550)	29.5 (40.5 - 21.5)
Mali	30,000 (40,000 - 22,000)	150.4 (203.1 - 109.0)	7,070 (9,610 - 5,070)	37.0 (50.9 - 26.3)
Mauritania	2,600 (3,580 - 1,840)	106.1 (143.4 - 77.1)	610 (850 - 420)	25.2 (34.1 - 17.7)
Niger	31,000 (41,000 - 24,000)	161.4 (210.8 - 122.3)	7,090 (9,530 - 5,050)	38.1 (51.4 - 27.5)
Nigeria	263,000 (336,000 - 203,000)	155.1 (199.9 - 118.2)	64,000 (84,000 - 48,000)	39.2 (51.7 - 29.0)
Sao Tome and Principe	140 (190 - 100)	136.6 (178.2 - 101.7)	30 (50 - 20)	32.6 (43.7 - 23.8)

Senegal	12,000 (16,000 - 8,790)	126.6 (167.9 - 93.7)	2,700 (3,630 - 1,950)	29.2 (39.8 - 21.3)
Sierra Leone	9,730 (13,000 - 7,080)	161.8 (218.0 - 121.2)	2,210 (3,100 - 1,560)	37.8 (52.1 - 27.5)
Togo	6,280 (8,270 - 4,650)	141.1 (186.3 - 106.8)	1,480 (2,020 - 1,080)	33.9 (46.2 - 24.8)
South Sudan	12,000 (15,000 - 8,840)	151.2 (198.6 - 114.5)	2,710 (3,650 - 1,960)	37.2 (51.9 - 26.1)

Supplementary Table 2. Deaths attributable to and associated with antimicrobial resistance (expressed as counts and age-standardised rates (ASMR) per 100 000 with 95% uncertainty intervals) per country in the WHO African region and pathogen.

Country	Pathogen	Deaths associated with AMR (counts)	Deaths associated with AMR (age-standardised	Deaths attributable to AMR (counts)	Deaths attributable to AMR (age-standardised rate per 100,000)
Algeria	Acinetobacter baumannii	1,100 (615 - 1,800)	4 (2 - 7)	357 (189 - 609)	1 (1 - 2)
Algeria	All	14,000 (8,800 - 20,000)	49 (32 - 72)	3,400 (2,200 - 5,200)	12 (8 - 19)
Algeria	Citrobacter spp	59 (34 - 96)	0 (0 - 0)	17 (9 - 29)	0 (0 - 0)
Algeria	Enterobacter spp	332 (205 - 529)	1 (1 - 2)	76 (45 - 123)	0 (0 - 0)
Algeria	Enterococcus faecalis	100 (50 - 176)	0 (0 - 1)	28 (12 - 54)	0 (0 - 0)
Algeria	Enterococcus faecium	695 (403 - 1,100)	2 (1 - 4)	203 (111 - 344)	1 (0 - 1)
Algeria	Enterococcus spp	259 (145 - 422)	1 (1 - 2)	61 (29 - 110)	0 (0 - 0)
Algeria	Escherichia coli	2,300 (1,500 - 3,500)	9 (5 - 13)	552 (336 - 872)	2 (1 - 3)
Algeria	Group A Strep	20 (9 - 41)	0 (0 - 0)	2 (0 - 7)	0 (0 - 0)
Algeria	Group B Strep	411 (253 - 642)	1 (1 - 2)	50 (0 - 130)	0 (0 - 0)
Algeria	Haemophilus influenzae	53 (34 - 79)	0 (0 - 0)	11 (3 - 21)	0 (0 - 0)
Algeria	Klebsiella pneumoniae	2,100 (1,400 - 3,300)	8 (5 - 12)	603 (332 - 1,000)	2 (1 - 4)
Algeria	Morganella spp	6 (2 - 11)	0 (0 - 0)	1 (0 - 3)	0 (0 - 0)
Algeria	Mycobacterium tuberculosis	26 (5 - 103)	0 (0 - 0)	14 (1 - 61)	0 (0 - 0)
Algeria	Neisseria Gonorrheae				
Algeria	Non Typhoidal Salmonellae	4 (1 - 10)	0 (0 - 0)	1 (0 - 3)	0 (0 - 0)
Algeria	Proteus spp	258 (151 - 410)	1 (1 - 2)	44 (22 - 77)	0 (0 - 0)
Algeria	Pseudomonas aeruginosa	1,200 (757 - 1,900)	4 (3 - 7)	314 (171 - 523)	1 (1 - 2)
Algeria	Salmonella Paratyphi	1 (0 - 3)	0 (0 - 0)	0 (0 - 1)	0 (0 - 0)
Algeria	Salmonella Typhi	10 (2 - 43)	0 (0 - 0)	2 (0 - 9)	0 (0 - 0)
Algeria	Serratia spp	91 (48 - 155)	0 (0 - 1)	24 (11 - 45)	0 (0 - 0)
Algeria	Shigella spp	4 (1 - 12)	0 (0 - 0)	1 (0 - 3)	0 (0 - 0)
Algeria	Staphylococcus Aureus	2,800 (1,900 - 4,100)	11 (7 - 15)	656 (363 - 1,100)	2 (1 - 4)
Algeria	Streptococcus pneumoniae	1,700 (1,200 - 2,200)	6 (4 - 8)	388 (238 - 584)	1 (1 - 2)
Angola	Acinetobacter baumannii	1,000 (635 - 1,600)	9 (5 - 14)	306 (179 - 484)	3 (2 - 4)
Angola	All	23,000 (17,000 - 30,000)	132 (98 - 178)	5,300 (3,800 - 7,600)	32 (23 - 45)
Angola	Citrobacter spp	170 (101 - 261)	1 (1 - 2)	49 (26 - 82)	0 (0 - 1)
Angola	Enterobacter spp	971 (669 - 1,400)	6 (4 - 9)	229 (149 - 336)	1 (1 - 2)
Angola	Enterococcus faecalis	412 (244 - 656)	3 (2 - 5)	109 (53 - 186)	1 (0 - 1)
Angola	Enterococcus faecium	454 (261 - 758)	4 (2 - 6)	114 (53 - 207)	1 (0 - 2)
Angola	Enterococcus spp	202 (126 - 320)	1 (1 - 2)	42 (10 - 85)	0 (0 - 1)
Angola	Escherichia coli	3,100 (2,300 - 4,100)	19 (14 - 26)	722 (496 - 997)	5 (3 - 6)
Angola	Group A Strep	114 (59 - 198)	1 (0 - 2)	11 (0 - 37)	0 (0 - 0)
Angola	Group B Strep	1,600 (1,200 - 2,200)	7 (5 - 9)	268 (69 - 505)	1 (0 - 2)
Angola	Haemophilus influenzae	380 (256 - 531)	2 (1 - 2)	80 (28 - 147)	0 (0 - 1)
Angola	Klebsiella pneumoniae	3,800 (2,800 - 5,100)	22 (16 - 30)	987 (602 - 1,500)	6 (4 - 9)
Angola	Morganella spp	4 (2 - 7)	0 (0 - 0)	1 (0 - 2)	0 (0 - 0)
Angola	Mycobacterium tuberculosis	1,200 (396 - 3,000)	8 (3 - 21)	523 (50 - 1,800)	4 (0 - 13)
Angola	Neisseria Gonorrheae				
Angola	Non Typhoidal Salmonellae	4 (1 - 9)	0 (0 - 0)	1 (0 - 2)	0 (0 - 0)
Angola	Proteus spp	247 (149 - 387)	2 (2 - 4)	32 (16 - 55)	0 (0 - 1)
Angola	Pseudomonas aeruginosa	1,200 (817 - 1,600)	7 (5 - 11)	303 (188 - 468)	2 (1 - 3)
Angola	Salmonella Paratyphi	0 (0 - 0)	0 (0 - 0)	0 (0 - 0)	0 (0 - 0)
Angola	Salmonella Typhi	409 (152 - 808)	1 (0 - 2)	79 (14 - 193)	0 (0 - 1)
Angola	Serratia spp	323 (199 - 491)	2 (1 - 3)	75 (40 - 128)	0 (0 - 1)
Angola	Shigella spp	13 (3 - 38)	0 (0 - 0)	3 (0 - 10)	0 (0 - 0)
Angola	Staphylococcus Aureus	2,200 (1,600 - 3,100)	15 (11 - 20)	459 (292 - 698)	3 (2 - 5)
Angola	Streptococcus pneumoniae	4,800 (3,500 - 6,700)	21 (17 - 27)	957 (569 - 1,500)	4 (3 - 6)

Benin	Acinetobacter baumannii	609 (369 - 925)	11 (7 - 17)	174 (100 - 279)	3 (2 - 5)
Benin	All	14,000 (10,000 - 19,000)	155 (115 - 206)	3,100 (2,200 - 4,300)	36 (26 - 48)
Benin	Citrobacter spp	73 (41 - 118)	1 (1 - 1)	22 (11 - 37)	0 (0 - 0)
Benin	Enterobacter spp	425 (288 - 612)	5 (4 - 8)	102 (66 - 152)	1 (1 - 2)
Benin	Enterococcus faecalis	220 (130 - 347)	3 (2 - 5)	58 (28 - 102)	1 (0 - 1)
Benin	Enterococcus faecium	219 (129 - 362)	4 (2 - 6)	54 (25 - 103)	1 (0 - 2)
Benin	Enterococcus spp	108 (65 - 173)	1 (1 - 2)	22 (5 - 44)	0 (0 - 1)
Benin	Escherichia coli	1,900 (1,400 - 2,600)	23 (16 - 31)	463 (314 - 658)	5 (4 - 8)
Benin	Group A Strep	42 (24 - 71)	1 (0 - 1)	4 (0 - 14)	0 (0 - 0)
Benin	Group B Strep	720 (496 - 989)	6 (4 - 8)	129 (37 - 250)	1 (0 - 2)
Benin	Haemophilus influenzae	155 (106 - 221)	1 (1 - 2)	37 (16 - 62)	0 (0 - 1)
Benin	Klebsiella pneumoniae	2,700 (1,900 - 3,600)	28 (21 - 38)	702 (424 - 1,100)	7 (5 - 11)
Benin	Morganella spp	2 (1 - 3)	0 (0 - 0)	0 (0 - 1)	0 (0 - 0)
Benin	Mycobacterium tuberculosis	313 (152 - 588)	5 (3 - 10)	100 (7 - 300)	2 (0 - 5)
Benin	Neisseria Gonorrheae				
Benin	Non Typhoidal Salmonellae	1 (0 - 2)	0 (0 - 0)	0 (0 - 0)	0 (0 - 0)
Benin	Proteus spp	119 (71 - 186)	2 (1 - 3)	16 (8 - 28)	0 (0 - 1)
Benin	Pseudomonas aeruginosa	772 (555 - 1,100)	9 (7 - 12)	180 (117 - 267)	2 (1 - 3)
Benin	Salmonella Paratyphi	0 (0 - 0)	0 (0 - 0)	0 (0 - 0)	0 (0 - 0)
Benin	Salmonella Typhi	748 (417 - 1,200)	5 (3 - 7)	113 (0 - 256)	1 (0 - 2)
Benin	Serratia spp	207 (124 - 326)	2 (1 - 4)	54 (27 - 91)	1 (0 - 1)
Benin	Shigella spp	23 (5 - 67)	0 (0 - 1)	5 (0 - 18)	0 (0 - 0)
Benin	Staphylococcus Aureus	2,000 (1,500 - 2,700)	25 (19 - 33)	411 (255 - 610)	5 (3 - 8)
Benin	Streptococcus pneumoniae	2,600 (1,800 - 3,600)	23 (17 - 30)	471 (256 - 753)	4 (2 - 6)
Botswana	Acinetobacter baumannii	117 (69 - 185)	10 (6 - 15)	37 (21 - 61)	3 (2 - 5)
Botswana	All	1,800 (1,300 - 2,500)	127 (91 - 177)	417 (276 - 615)	29 (20 - 42)
Botswana	Citrobacter spp	7 (4 - 11)	1 (0 - 1)	2 (1 - 4)	0 (0 - 0)
Botswana	Enterobacter spp	56 (38 - 82)	4 (3 - 6)	10 (6 - 16)	1 (0 - 1)
Botswana	Enterococcus faecalis	25 (14 - 43)	2 (1 - 3)	7 (3 - 13)	1 (0 - 1)
Botswana	Enterococcus faecium	60 (36 - 96)	4 (3 - 7)	15 (7 - 27)	1 (1 - 2)
Botswana	Enterococcus spp	21 (13 - 34)	2 (1 - 3)	4 (1 - 9)	0 (0 - 1)
Botswana	Escherichia coli	270 (187 - 386)	21 (14 - 29)	58 (38 - 85)	4 (3 - 7)
Botswana	Group A Strep	3 (1 - 5)	0 (0 - 0)	0 (0 - 1)	0 (0 - 0)
Botswana	Group B Strep	52 (37 - 72)	3 (2 - 5)	7 (0 - 16)	1 (0 - 1)
Botswana	Haemophilus influenzae	12 (8 - 16)	1 (1 - 1)	3 (1 - 5)	0 (0 - 0)
Botswana	Klebsiella pneumoniae	304 (216 - 422)	22 (16 - 30)	76 (43 - 122)	6 (3 - 9)
Botswana	Morganella spp	0 (0 - 0)	0 (0 - 0)	0 (0 - 0)	0 (0 - 0)
Botswana	Mycobacterium tuberculosis	140 (60 - 311)	8 (4 - 18)	59 (10 - 183)	3 (1 - 10)
Botswana	Neisseria Gonorrheae				
Botswana	Non Typhoidal Salmonellae	0 (0 - 0)	0 (0 - 0)	0 (0 - 0)	0 (0 - 0)
Botswana	Proteus spp	24 (14 - 37)	2 (1 - 3)	3 (1 - 5)	0 (0 - 0)
Botswana	Pseudomonas aeruginosa	133 (91 - 191)	10 (7 - 14)	32 (21 - 48)	2 (2 - 4)
Botswana	Salmonella Paratyphi	0 (0 - 0)	0 (0 - 0)	0 (0 - 0)	0 (0 - 0)
Botswana	Salmonella Typhi	13 (5 - 25)	1 (0 - 1)	2 (0 - 5)	0 (0 - 0)
Botswana	Serratia spp	12 (8 - 20)	1 (1 - 1)	2 (1 - 4)	0 (0 - 0)
Botswana	Shigella spp	2 (1 - 5)	0 (0 - 0)	0 (0 - 1)	0 (0 - 0)
Botswana	Staphylococcus Aureus	239 (173 - 327)	18 (13 - 24)	36 (23 - 52)	3 (2 - 4)
Botswana	Streptococcus pneumoniae	300 (224 - 407)	19 (14 - 25)	63 (39 - 94)	4 (3 - 6)
Burkina Faso	Acinetobacter baumannii	1,200 (739 - 1,800)	11 (6 - 17)	343 (199 - 548)	3 (2 - 5)
Burkina Faso	All	30,000 (23,000 - 39,000)	173 (135 - 219)	7,000 (5,100 - 9,200)	41 (31 - 54)
Burkina Faso	Citrobacter spp	144 (84 - 229)	1 (1 - 1)	42 (22 - 73)	0 (0 - 0)
Burkina Faso	Enterobacter spp	794 (562 - 1,100)	5 (4 - 7)	192 (126 - 274)	1 (1 - 2)
Burkina Faso	Enterococcus faecalis	484 (296 - 716)	3 (2 - 5)	127 (62 - 215)	1 (0 - 2)
Burkina Faso	Enterococcus faecium	458 (279 - 748)	4 (2 - 6)	113 (53 - 206)	1 (0 - 2)
Burkina Faso	Enterococcus spp	216 (134 - 335)	2 (1 - 3)	45 (10 - 89)	0 (0 - 1)
Burkina Faso	Escherichia coli	4,300 (3,300 - 5,700)	27 (20 - 34)	1,100 (752 - 1,500)	7 (5 - 9)
Burkina Faso	Group A Strep	95 (54 - 161)	1 (0 - 1)	9 (0 - 33)	0 (0 - 0)

Burkina Faso	Group B Strep	1,300 (945 - 1,800)	6 (4 - 8)	232 (67 - 445)	1 (0 - 2)
Burkina Faso	Haemophilus influenzae	454 (313 - 624)	2 (1 - 2)	106 (46 - 176)	0 (0 - 1)
Burkina Faso	Klebsiella pneumoniae	6,200 (4,800 - 8,200)	33 (26 - 43)	1,700 (1,000 - 2,500)	9 (6 - 13)
Burkina Faso	Morganella spp	6 (3 - 10)	0 (0 - 0)	1 (1 - 2)	0 (0 - 0)
Burkina Faso	Mycobacterium tuberculosis	659 (204 - 1,700)	6 (2 - 15)	317 (29 - 1,100)	3 (0 - 9)
Burkina Faso	Neisseria Gonorrheae				
Burkina Faso	Non Typhoidal Salmonellae	38 (11 - 97)	0 (0 - 0)	8 (1 - 24)	0 (0 - 0)
Burkina Faso	Proteus spp	241 (150 - 372)	2 (1 - 4)	32 (17 - 55)	0 (0 - 1)
Burkina Faso	Pseudomonas aeruginosa	1,500 (1,100 - 1,900)	9 (7 - 12)	355 (236 - 526)	2 (1 - 3)
Burkina Faso	Salmonella Paratyphi	15 (3 - 43)	0 (0 - 0)	3 (0 - 11)	0 (0 - 0)
Burkina Faso	Salmonella Typhi	1,300 (480 - 2,600)	4 (2 - 8)	207 (12 - 518)	1 (0 - 2)
Burkina Faso	Serratia spp	413 (255 - 644)	2 (1 - 4)	108 (58 - 180)	1 (0 - 1)
Burkina Faso	Shigella spp	39 (9 - 105)	0 (0 - 1)	8 (1 - 26)	0 (0 - 0)
Burkina Faso	Staphylococcus Aureus	4,800 (3,700 - 6,100)	31 (25 - 38)	943 (618 - 1,400)	6 (4 - 8)
Burkina Faso	Streptococcus pneumoniae	5,800 (4,300 - 7,700)	25 (20 - 31)	1,100 (600 - 1,700)	5 (3 - 7)
Burundi	Acinetobacter baumannii	598 (360 - 916)	14 (8 - 22)	179 (101 - 298)	4 (2 - 7)
Burundi	All	11,000 (8,300 - 15,000)	167 (124 - 221)	2,700 (1,900 - 4,100)	42 (29 - 65)
Burundi	Citrobacter spp	51 (30 - 81)	1 (0 - 1)	15 (8 - 26)	0 (0 - 0)
Burundi	Enterobacter spp	296 (198 - 429)	5 (3 - 7)	71 (43 - 108)	1 (1 - 2)
Burundi	Enterococcus faecalis	202 (118 - 314)	3 (2 - 5)	53 (25 - 92)	1 (0 - 2)
Burundi	Enterococcus faecium	193 (111 - 320)	4 (2 - 6)	49 (23 - 92)	1 (0 - 2)
Burundi	Enterococcus spp	59 (34 - 95)	1 (1 - 2)	12 (3 - 25)	0 (0 - 1)
Burundi	Escherichia coli	1,700 (1,200 - 2,400)	25 (18 - 33)	385 (262 - 557)	6 (4 - 8)
Burundi	Group A Strep	81 (44 - 141)	2 (1 - 3)	8 (0 - 27)	0 (0 - 1)
Burundi	Group B Strep	463 (327 - 645)	5 (4 - 7)	69 (11 - 138)	1 (0 - 2)
Burundi	Haemophilus influenzae	219 (150 - 320)	2 (2 - 3)	43 (12 - 79)	1 (0 - 1)
Burundi	Klebsiella pneumoniae	2,000 (1,400 - 2,700)	29 (21 - 38)	497 (302 - 787)	7 (4 - 11)
Burundi	Morganella spp	2 (1 - 4)	0 (0 - 0)	1 (0 - 1)	0 (0 - 0)
Burundi	Mycobacterium tuberculosis	707 (162 - 2,300)	13 (3 - 41)	379 (33 - 1,500)	7 (1 - 26)
Burundi	Neisseria Gonorrheae				
Burundi	Non Typhoidal Salmonellae	1 (0 - 2)	0 (0 - 0)	0 (0 - 1)	0 (0 - 0)
Burundi	Proteus spp	119 (70 - 183)	3 (2 - 4)	16 (9 - 27)	0 (0 - 1)
Burundi	Pseudomonas aeruginosa	495 (351 - 677)	8 (5 - 11)	130 (78 - 209)	2 (1 - 3)
Burundi	Salmonella Paratyphi	0 (0 - 0)	0 (0 - 0)	0 (0 - 0)	0 (0 - 0)
Burundi	Salmonella Typhi	615 (272 - 1,000)	4 (2 - 8)	101 (9 - 243)	1 (0 - 2)
Burundi	Serratia spp	157 (95 - 245)	2 (1 - 3)	40 (20 - 66)	1 (0 - 1)
Burundi	Shigella spp	176 (41 - 496)	2 (0 - 4)	36 (3 - 113)	0 (0 - 1)
Burundi	Staphylococcus Aureus	1,400 (1,000 - 1,800)	24 (18 - 31)	285 (176 - 421)	5 (3 - 7)
Burundi	Streptococcus pneumoniae	1,800 (1,300 - 2,600)	23 (18 - 29)	378 (218 - 589)	5 (3 - 7)
Cameroon	Acinetobacter baumannii	1,200 (726 - 1,800)	10 (6 - 16)	355 (209 - 566)	3 (2 - 5)
Cameroon	All	25,000 (18,000 - 32,000)	144 (107 - 192)	5,600 (4,000 - 7,500)	34 (24 - 46)
Cameroon	Citrobacter spp	129 (76 - 208)	1 (0 - 1)	38 (20 - 64)	0 (0 - 0)
Cameroon	Enterobacter spp	786 (548 - 1,100)	5 (3 - 7)	186 (120 - 268)	1 (1 - 2)
Cameroon	Enterococcus faecalis	368 (221 - 578)	2 (1 - 4)	98 (45 - 173)	1 (0 - 1)
Cameroon	Enterococcus faecium	458 (264 - 755)	3 (2 - 6)	116 (55 - 215)	1 (0 - 2)
Cameroon	Enterococcus spp	231 (145 - 356)	2 (1 - 3)	47 (11 - 94)	0 (0 - 1)
Cameroon	Escherichia coli	3,500 (2,500 - 4,700)	21 (15 - 29)	849 (581 - 1,200)	5 (4 - 7)
Cameroon	Group A Strep	99 (52 - 175)	1 (0 - 1)	9 (0 - 35)	0 (0 - 0)
Cameroon	Group B Strep	1,000 (743 - 1,400)	5 (3 - 6)	171 (43 - 323)	1 (0 - 2)
Cameroon	Haemophilus influenzae	218 (150 - 318)	1 (1 - 1)	49 (20 - 86)	0 (0 - 0)
Cameroon	Klebsiella pneumoniae	4,400 (3,300 - 5,800)	25 (18 - 34)	1,000 (666 - 1,500)	6 (4 - 9)
Cameroon	Morganella spp	5 (3 - 8)	0 (0 - 0)	1 (1 - 2)	0 (0 - 0)
Cameroon	Mycobacterium tuberculosis	921 (445 - 1,800)	7 (3 - 13)	344 (83 - 972)	3 (1 - 7)
Cameroon	Neisseria Gonorrheae				
Cameroon	Non Typhoidal Salmonellae	3 (1 - 9)	0 (0 - 0)	1 (0 - 2)	0 (0 - 0)
Cameroon	Proteus spp	241 (148 - 375)	2 (1 - 3)	31 (16 - 53)	0 (0 - 1)
Cameroon	Pseudomonas aeruginosa	1,500 (1,100 - 2,100)	10 (7 - 13)	381 (247 - 579)	2 (2 - 4)

Cameroon	Salmonella Paratyphi	1 (0 - 3)	0 (0 - 0)	0 (0 - 1)	0 (0 - 0)
Cameroon	Salmonella Typhi	1,200 (634 - 1,800)	4 (2 - 6)	176 (0 - 395)	1 (0 - 1)
Cameroon	Serratia spp	361 (230 - 558)	2 (1 - 3)	96 (52 - 165)	1 (0 - 1)
Cameroon	Shigella spp	168 (41 - 461)	1 (0 - 2)	35 (3 - 114)	0 (0 - 0)
Cameroon	Staphylococcus Aureus	3,500 (2,600 - 4,600)	23 (18 - 31)	793 (479 - 1,200)	5 (3 - 8)
Cameroon	Streptococcus pneumoniae	4,300 (3,100 - 5,900)	21 (16 - 27)	787 (444 - 1,200)	4 (2 - 6)
Cape Verde	Acinetobacter baumannii	47 (29 - 72)	11 (7 - 17)	15 (9 - 23)	4 (2 - 6)
Cape Verde	All	321 (233 - 433)	74 (54 - 100)	79 (56 - 108)	18 (13 - 25)
Cape Verde	Citrobacter spp	3 (2 - 5)	1 (0 - 1)	1 (0 - 2)	0 (0 - 0)
Cape Verde	Enterobacter spp	21 (13 - 31)	5 (3 - 7)	4 (3 - 7)	1 (1 - 2)
Cape Verde	Enterococcus faecalis	5 (3 - 8)	1 (1 - 2)	1 (1 - 3)	0 (0 - 1)
Cape Verde	Enterococcus faecium	11 (7 - 17)	3 (2 - 4)	3 (1 - 5)	1 (0 - 1)
Cape Verde	Enterococcus spp	5 (3 - 8)	1 (1 - 2)	1 (0 - 2)	0 (0 - 1)
Cape Verde	Escherichia coli	43 (30 - 59)	10 (7 - 14)	10 (7 - 14)	2 (2 - 3)
Cape Verde	Group A Strep	1 (1 - 3)	0 (0 - 1)	0 (0 - 1)	0 (0 - 0)
Cape Verde	Group B Strep	6 (5 - 9)	2 (1 - 2)	1 (0 - 2)	0 (0 - 0)
Cape Verde	Haemophilus influenzae	1 (1 - 2)	0 (0 - 0)	0 (0 - 0)	0 (0 - 0)
Cape Verde	Klebsiella pneumoniae	43 (31 - 58)	10 (7 - 14)	11 (7 - 17)	3 (2 - 4)
Cape Verde	Morganella spp	0 (0 - 0)	0 (0 - 0)	0 (0 - 0)	0 (0 - 0)
Cape Verde	Mycobacterium tuberculosis	6 (1 - 15)	1 (0 - 3)	3 (0 - 10)	1 (0 - 2)
Cape Verde	Neisseria Gonorrheae				
Cape Verde	Non Typhoidal Salmonellae	0 (0 - 0)	0 (0 - 0)	0 (0 - 0)	0 (0 - 0)
Cape Verde	Proteus spp	5 (3 - 8)	1 (1 - 2)	1 (0 - 1)	0 (0 - 0)
Cape Verde	Pseudomonas aeruginosa	24 (17 - 33)	6 (4 - 8)	6 (4 - 9)	1 (1 - 2)
Cape Verde	Salmonella Paratyphi	0 (0 - 0)	0 (0 - 0)	0 (0 - 0)	0 (0 - 0)
Cape Verde	Salmonella Typhi	5 (2 - 9)	1 (0 - 2)	1 (0 - 2)	0 (0 - 0)
Cape Verde	Serratia spp	4 (2 - 7)	1 (1 - 2)	1 (1 - 2)	0 (0 - 1)
Cape Verde	Shigella spp	0 (0 - 0)	0 (0 - 0)	0 (0 - 0)	0 (0 - 0)
Cape Verde	Staphylococcus Aureus	44 (33 - 58)	10 (8 - 14)	11 (6 - 16)	2 (1 - 4)
Cape Verde	Streptococcus pneumoniae	46 (38 - 55)	10 (9 - 12)	9 (6 - 13)	2 (1 - 3)
Central African	Acinetobacter baumannii	346 (217 - 536)	17 (10 - 27)	101 (58 - 167)	5 (3 - 8)
Central African	All	9,500 (6,900 - 13,000)	251 (181 - 340)	2,200 (1,500 - 3,200)	60 (40 - 89)
Central African	Citrobacter spp	34 (19 - 56)	1 (1 - 2)	10 (5 - 18)	0 (0 - 1)
Central African	Enterobacter spp	234 (154 - 345)	7 (5 - 11)	57 (36 - 88)	2 (1 - 3)
Central African	Enterococcus faecalis	124 (72 - 198)	4 (2 - 8)	33 (16 - 59)	1 (1 - 2)
Central African	Enterococcus faecium	97 (52 - 178)	4 (2 - 7)	25 (11 - 51)	1 (0 - 2)
Central African	Enterococcus spp	30 (16 - 50)	1 (1 - 2)	6 (2 - 13)	0 (0 - 1)
Central African	Escherichia coli	1,100 (783 - 1,500)	29 (21 - 39)	260 (184 - 362)	7 (5 - 10)
Central African	Group A Strep	23 (12 - 41)	1 (0 - 2)	2 (0 - 8)	0 (0 - 0)
Central African	Group B Strep	769 (528 - 1,100)	14 (9 - 20)	138 (40 - 264)	3 (1 - 5)
Central African	Haemophilus influenzae	259 (180 - 364)	5 (3 - 7)	58 (24 - 97)	1 (1 - 2)
Central African	Klebsiella pneumoniae	1,400 (1,000 - 1,900)	36 (26 - 49)	358 (216 - 539)	9 (6 - 15)
Central African	Morganella spp	2 (1 - 3)	0 (0 - 0)	0 (0 - 1)	0 (0 - 0)
Central African	Mycobacterium tuberculosis	1,100 (502 - 2,100)	36 (17 - 67)	308 (0 - 917)	10 (0 - 30)
Central African	Neisseria Gonorrheae				
Central African	Non Typhoidal Salmonellae	1 (0 - 4)	0 (0 - 0)	0 (0 - 1)	0 (0 - 0)
Central African	Proteus spp	62 (34 - 103)	3 (2 - 5)	8 (4 - 15)	0 (0 - 1)
Central African	Pseudomonas aeruginosa	296 (209 - 407)	9 (6 - 12)	76 (48 - 114)	2 (1 - 3)
Central African	Salmonella Paratyphi	0 (0 - 0)	0 (0 - 0)	0 (0 - 0)	0 (0 - 0)
Central African	Salmonella Typhi	376 (124 - 710)	6 (2 - 12)	72 (10 - 187)	1 (0 - 3)
Central African	Serratia spp	98 (58 - 156)	3 (2 - 5)	25 (13 - 42)	1 (0 - 1)
Central African	Shigella spp	2 (0 - 5)	0 (0 - 0)	0 (0 - 1)	0 (0 - 0)
Central African	Staphylococcus Aureus	964 (698 - 1,300)	29 (21 - 41)	177 (110 - 259)	5 (3 - 8)
Central African	Streptococcus pneumoniae	2,200 (1,600 - 3,100)	45 (34 - 60)	522 (339 - 786)	11 (7 - 16)
Chad	Acinetobacter baumannii	1,000 (705 - 1,500)	12 (8 - 19)	297 (189 - 448)	3 (2 - 5)
Chad	All	25,000 (19,000 - 32,000)	171 (132 - 219)	5,800 (4,300 - 7,700)	42 (30 - 56)
Chad	Citrobacter spp	242 (141 - 382)	2 (1 - 3)	74 (40 - 122)	1 (0 - 1)

Chad	Enterobacter spp	1,100 (753 - 1,500)	8 (6 - 12)	264 (178 - 372)	2 (1 - 3)
Chad	Enterococcus faecalis	278 (171 - 428)	3 (2 - 5)	74 (36 - 128)	1 (0 - 2)
Chad	Enterococcus faecium	272 (161 - 468)	4 (2 - 7)	68 (31 - 136)	1 (0 - 2)
Chad	Enterococcus spp	162 (98 - 256)	2 (1 - 3)	33 (8 - 66)	0 (0 - 1)
Chad	Escherichia coli	3,400 (2,500 - 4,400)	24 (18 - 31)	864 (609 - 1,200)	6 (4 - 8)
Chad	Group A Strep	66 (37 - 109)	1 (0 - 2)	6 (0 - 22)	0 (0 - 0)
Chad	Group B Strep	2,100 (1,400 - 2,900)	10 (7 - 14)	376 (106 - 722)	2 (1 - 3)
Chad	Haemophilus influenzae	556 (381 - 762)	2 (2 - 3)	129 (58 - 214)	1 (0 - 1)
Chad	Klebsiella pneumoniae	4,000 (3,000 - 5,300)	27 (21 - 36)	1,100 (671 - 1,700)	8 (5 - 11)
Chad	Morganella spp	3 (2 - 5)	0 (0 - 0)	1 (0 - 1)	0 (0 - 0)
Chad	Mycobacterium tuberculosis	679 (230 - 1,700)	9 (3 - 22)	295 (30 - 1,100)	4 (0 - 14)
Chad	Neisseria Gonorrheae				
Chad	Non Typhoidal Salmonellae	4 (1 - 10)	0 (0 - 0)	1 (0 - 3)	0 (0 - 0)
Chad	Proteus spp	167 (100 - 267)	3 (2 - 4)	24 (12 - 42)	0 (0 - 1)
Chad	Pseudomonas aeruginosa	942 (688 - 1,300)	7 (5 - 10)	233 (150 - 349)	2 (1 - 3)
Chad	Salmonella Paratyphi	0 (0 - 1)	0 (0 - 0)	0 (0 - 0)	0 (0 - 0)
Chad	Salmonella Typhi	372 (111 - 841)	2 (1 - 4)	60 (3 - 188)	0 (0 - 1)
Chad	Serratia spp	450 (287 - 682)	3 (2 - 5)	120 (64 - 207)	1 (1 - 2)
Chad	Shigella spp	162 (39 - 439)	1 (0 - 2)	34 (3 - 117)	0 (0 - 1)
Chad	Staphylococcus Aureus	2,000 (1,500 - 2,500)	16 (12 - 22)	392 (250 - 569)	3 (2 - 5)
Chad	Streptococcus pneumoniae	7,100 (5,200 - 9,300)	35 (28 - 44)	1,400 (782 - 2,000)	7 (4 - 10)
Comoros	Acinetobacter baumannii	68 (42 - 104)	15 (9 - 23)	20 (11 - 32)	4 (3 - 7)
Comoros	All	644 (478 - 878)	125 (92 - 169)	165 (110 - 251)	32 (22 - 50)
Comoros	Citrobacter spp	7 (4 - 11)	1 (1 - 2)	2 (1 - 3)	0 (0 - 1)
Comoros	Enterobacter spp	34 (23 - 49)	7 (4 - 9)	8 (5 - 13)	2 (1 - 2)
Comoros	Enterococcus faecalis	12 (7 - 20)	2 (1 - 4)	3 (1 - 6)	1 (0 - 1)
Comoros	Enterococcus faecium	14 (8 - 23)	3 (2 - 5)	3 (1 - 7)	1 (0 - 1)
Comoros	Enterococcus spp	6 (4 - 10)	1 (1 - 2)	1 (0 - 3)	0 (0 - 1)
Comoros	Escherichia coli	84 (63 - 108)	17 (13 - 22)	20 (14 - 29)	4 (3 - 6)
Comoros	Group A Strep	3 (2 - 7)	1 (0 - 2)	0 (0 - 1)	0 (0 - 0)
Comoros	Group B Strep	23 (16 - 32)	4 (3 - 6)	3 (1 - 7)	1 (0 - 1)
Comoros	Haemophilus influenzae	7 (5 - 9)	1 (1 - 2)	1 (1 - 3)	0 (0 - 0)
Comoros	Klebsiella pneumoniae	93 (69 - 124)	18 (13 - 24)	23 (15 - 35)	5 (3 - 7)
Comoros	Morganella spp	0 (0 - 0)	0 (0 - 0)	0 (0 - 0)	0 (0 - 0)
Comoros	Mycobacterium tuberculosis	54 (10 - 151)	11 (2 - 30)	29 (2 - 101)	6 (0 - 20)
Comoros	Neisseria Gonorrheae				
Comoros	Non Typhoidal Salmonellae	0 (0 - 0)	0 (0 - 0)	0 (0 - 0)	0 (0 - 0)
Comoros	Proteus spp	10 (6 - 15)	2 (1 - 3)	1 (1 - 2)	0 (0 - 1)
Comoros	Pseudomonas aeruginosa	31 (22 - 43)	6 (4 - 9)	8 (5 - 12)	2 (1 - 3)
Comoros	Salmonella Paratyphi	0 (0 - 0)	0 (0 - 0)	0 (0 - 0)	0 (0 - 0)
Comoros	Salmonella Typhi	11 (5 - 21)	2 (1 - 3)	2 (0 - 4)	0 (0 - 1)
Comoros	Serratia spp	10 (6 - 17)	2 (1 - 3)	3 (1 - 4)	1 (0 - 1)
Comoros	Shigella spp	1 (0 - 3)	0 (0 - 1)	0 (0 - 1)	0 (0 - 0)
Comoros	Staphylococcus Aureus	63 (48 - 84)	13 (10 - 17)	12 (8 - 18)	2 (2 - 4)
Comoros	Streptococcus pneumoniae	114 (88 - 143)	20 (15 - 24)	23 (14 - 32)	4 (3 - 6)
Congo	Acinetobacter baumannii	251 (145 - 399)	12 (7 - 19)	77 (43 - 123)	4 (2 - 6)
Congo	All	3,600 (2,600 - 4,900)	134 (98 - 181)	890 (620 - 1,300)	33 (24 - 47)
Congo	Citrobacter spp	23 (13 - 36)	1 (1 - 1)	7 (4 - 12)	0 (0 - 0)
Congo	Enterobacter spp	141 (95 - 207)	5 (4 - 8)	34 (22 - 51)	1 (1 - 2)
Congo	Enterococcus faecalis	78 (45 - 125)	3 (2 - 5)	21 (10 - 37)	1 (0 - 1)
Congo	Enterococcus faecium	91 (51 - 153)	4 (2 - 6)	23 (10 - 41)	1 (0 - 2)
Congo	Enterococcus spp	39 (24 - 63)	2 (1 - 3)	8 (2 - 17)	0 (0 - 1)
Congo	Escherichia coli	521 (375 - 714)	20 (15 - 27)	130 (88 - 190)	5 (3 - 7)
Congo	Group A Strep	15 (8 - 29)	1 (0 - 1)	1 (0 - 5)	0 (0 - 0)
Congo	Group B Strep	177 (125 - 243)	5 (4 - 7)	30 (9 - 57)	1 (0 - 2)
Congo	Haemophilus influenzae	32 (22 - 44)	1 (1 - 1)	7 (3 - 12)	0 (0 - 0)
Congo	Klebsiella pneumoniae	629 (455 - 864)	23 (17 - 32)	165 (100 - 254)	6 (4 - 10)

Congo	Morganella spp	1 (1 - 2)	0 (0 - 0)	0 (0 - 0)	0 (0 - 0)
Congo	Mycobacterium tuberculosis	164 (54 - 438)	5 (2 - 14)	68 (6 - 263)	2 (0 - 9)
Congo	Neisseria Gonorrheae				
Congo	Non Typhoidal Salmonellae	0 (0 - 1)	0 (0 - 0)	0 (0 - 0)	0 (0 - 0)
Congo	Proteus spp	52 (30 - 81)	2 (2 - 4)	7 (4 - 12)	0 (0 - 1)
Congo	Pseudomonas aeruginosa	218 (151 - 309)	9 (6 - 12)	55 (34 - 88)	2 (1 - 3)
Congo	Salmonella Paratyphi	0 (0 - 0)	0 (0 - 0)	0 (0 - 0)	0 (0 - 0)
Congo	Salmonella Typhi	82 (29 - 147)	2 (1 - 3)	15 (3 - 36)	0 (0 - 1)
Congo	Serratia spp	51 (32 - 80)	2 (1 - 3)	12 (6 - 21)	0 (0 - 1)
Congo	Shigella spp	3 (1 - 9)	0 (0 - 0)	1 (0 - 2)	0 (0 - 0)
Congo	Staphylococcus Aureus	491 (363 - 664)	20 (15 - 26)	115 (69 - 177)	5 (3 - 7)
Congo	Streptococcus pneumoniae	563 (423 - 738)	19 (14 - 24)	113 (72 - 164)	4 (2 - 5)
Cote d'Ivoire	Acinetobacter baumannii	1,200 (711 - 1,700)	11 (6 - 17)	332 (196 - 513)	3 (2 - 5)
Cote d'Ivoire	All	25,000 (18,000 - 32,000)	150 (115 - 195)	5,800 (4,200 - 7,800)	36 (26 - 48)
Cote d'Ivoire	Citrobacter spp	133 (77 - 210)	1 (1 - 1)	39 (22 - 69)	0 (0 - 0)
Cote d'Ivoire	Enterobacter spp	821 (564 - 1,200)	5 (4 - 8)	199 (130 - 290)	1 (1 - 2)
Cote d'Ivoire	Enterococcus faecalis	418 (249 - 646)	3 (2 - 5)	110 (53 - 192)	1 (0 - 1)
Cote d'Ivoire	Enterococcus faecium	407 (238 - 673)	3 (2 - 5)	101 (45 - 193)	1 (0 - 2)
Cote d'Ivoire	Enterococcus spp	210 (133 - 334)	2 (1 - 2)	43 (9 - 87)	0 (0 - 1)
Cote d'Ivoire	Escherichia coli	3,400 (2,500 - 4,600)	22 (16 - 29)	854 (581 - 1,200)	5 (4 - 8)
Cote d'Ivoire	Group A Strep	82 (45 - 144)	1 (0 - 1)	8 (0 - 29)	0 (0 - 0)
Cote d'Ivoire	Group B Strep	1,500 (1,100 - 2,100)	7 (5 - 9)	265 (77 - 503)	1 (0 - 2)
Cote d'Ivoire	Haemophilus influenzae	247 (166 - 350)	1 (1 - 2)	59 (25 - 99)	0 (0 - 0)
Cote d'Ivoire	Klebsiella pneumoniae	4,700 (3,500 - 6,100)	27 (20 - 36)	1,200 (755 - 1,900)	7 (4 - 11)
Cote d'Ivoire	Morganella spp	5 (2 - 7)	0 (0 - 0)	1 (0 - 2)	0 (0 - 0)
Cote d'Ivoire	Mycobacterium tuberculosis	781 (276 - 1,900)	6 (2 - 15)	349 (37 - 1,200)	3 (0 - 10)
Cote d'Ivoire	Neisseria Gonorrheae				
Cote d'Ivoire	Non Typhoidal Salmonellae	2 (1 - 7)	0 (0 - 0)	1 (0 - 2)	0 (0 - 0)
Cote d'Ivoire	Proteus spp	222 (134 - 353)	2 (1 - 3)	32 (16 - 54)	0 (0 - 1)
Cote d'Ivoire	Pseudomonas aeruginosa	1,400 (1,000 - 1,900)	9 (7 - 12)	348 (227 - 515)	2 (1 - 3)
Cote d'Ivoire	Salmonella Paratyphi	0 (0 - 1)	0 (0 - 0)	0 (0 - 0)	0 (0 - 0)
Cote d'Ivoire	Salmonella Typhi	1,200 (610 - 1,900)	4 (2 - 6)	169 (0 - 398)	1 (0 - 1)
Cote d'Ivoire	Serratia spp	409 (256 - 635)	2 (1 - 4)	108 (58 - 180)	1 (0 - 1)
Cote d'Ivoire	Shigella spp	11 (2 - 32)	0 (0 - 0)	2 (0 - 8)	0 (0 - 0)
Cote d'Ivoire	Staphylococcus Aureus	3,300 (2,500 - 4,300)	23 (18 - 30)	683 (439 - 998)	5 (3 - 7)
Cote d'Ivoire	Streptococcus pneumoniae	4,400 (3,200 - 5,800)	22 (17 - 28)	855 (496 - 1,300)	4 (3 - 6)
Democratic	Acinetobacter baumannii	4,000 (2,500 - 6,000)	12 (7 - 20)	1,200 (692 - 1,800)	4 (2 - 6)
Democratic	All	76,000 (57,000 - 98,000)	159 (118 - 208)	18,000 (13,000 - 26,000)	39 (28 - 56)
Democratic	Citrobacter spp	353 (207 - 557)	1 (0 - 1)	101 (54 - 176)	0 (0 - 0)
Democratic	Enterobacter spp	2,200 (1,500 - 3,200)	5 (3 - 7)	538 (352 - 798)	1 (1 - 2)
Democratic	Enterococcus faecalis	1,300 (791 - 2,100)	3 (2 - 5)	350 (172 - 610)	1 (0 - 1)
Democratic	Enterococcus faecium	1,300 (772 - 2,200)	3 (2 - 6)	332 (149 - 620)	1 (0 - 2)
Democratic	Enterococcus spp	522 (318 - 845)	1 (1 - 2)	108 (25 - 223)	0 (0 - 1)
Democratic	Escherichia coli	9,500 (7,100 - 13,000)	21 (15 - 28)	2,400 (1,600 - 3,400)	5 (4 - 7)
Democratic	Group A Strep	431 (231 - 761)	1 (1 - 2)	41 (0 - 146)	0 (0 - 0)
Democratic	Group B Strep	4,100 (2,900 - 5,500)	6 (5 - 9)	706 (206 - 1,400)	1 (0 - 2)
Democratic	Haemophilus influenzae	1,300 (893 - 1,700)	2 (2 - 3)	259 (93 - 468)	0 (0 - 1)
Democratic	Klebsiella pneumoniae	13,000 (9,400 - 16,000)	26 (19 - 35)	3,300 (2,000 - 5,000)	7 (4 - 10)
Democratic	Morganella spp	16 (8 - 26)	0 (0 - 0)	4 (2 - 7)	0 (0 - 0)
Democratic	Mycobacterium tuberculosis	6,700 (3,500 - 14,000)	15 (8 - 32)	2,300 (238 - 7,300)	5 (1 - 17)
Democratic	Neisseria Gonorrheae				
Democratic	Non Typhoidal Salmonellae	327 (165 - 577)	0 (0 - 1)	69 (11 - 173)	0 (0 - 0)
Democratic	Proteus spp	792 (476 - 1,300)	2 (1 - 4)	110 (56 - 188)	0 (0 - 1)
Democratic	Pseudomonas aeruginosa	3,700 (2,600 - 5,000)	8 (6 - 11)	958 (607 - 1,500)	2 (1 - 3)
Democratic	Salmonella Paratyphi	0 (0 - 0)	0 (0 - 0)	0 (0 - 0)	0 (0 - 0)
Democratic	Salmonella Typhi	3,700 (2,400 - 5,300)	4 (3 - 6)	690 (229 - 1,300)	1 (0 - 1)
Democratic	Serratia spp	952 (598 - 1,500)	2 (1 - 3)	224 (120 - 381)	0 (0 - 1)

Democratic	Shigella spp	87 (18 - 264)	0 (0 - 0)	18 (1 - 68)	0 (0 - 0)
Democratic	Staphylococcus Aureus	8,700 (6,600 - 11,000)	21 (16 - 28)	1,900 (1,200 - 2,800)	5 (3 - 7)
Democratic	Streptococcus pneumoniae	13,000 (9,900 - 17,000)	24 (18 - 31)	2,800 (1,800 - 4,100)	5 (3 - 7)
Equatorial	Acinetobacter baumannii	62 (38 - 96)	11 (7 - 17)	19 (11 - 32)	4 (2 - 6)
Equatorial	All	624 (416 - 913)	98 (67 - 140)	150 (99 - 224)	24 (16 - 35)
Equatorial	Citrobacter spp	9 (5 - 14)	1 (1 - 2)	3 (1 - 4)	0 (0 - 1)
Equatorial	Enterobacter spp	47 (30 - 71)	6 (4 - 10)	10 (6 - 16)	1 (1 - 2)
Equatorial	Enterococcus faecalis	11 (6 - 18)	2 (1 - 3)	3 (1 - 5)	1 (0 - 1)
Equatorial	Enterococcus faecium	16 (9 - 26)	3 (2 - 5)	4 (2 - 8)	1 (0 - 1)
Equatorial	Enterococcus spp	11 (7 - 18)	2 (1 - 3)	2 (0 - 5)	0 (0 - 1)
Equatorial	Escherichia coli	74 (49 - 110)	13 (9 - 19)	18 (11 - 27)	3 (2 - 5)
Equatorial	Group A Strep	3 (1 - 5)	1 (0 - 1)	0 (0 - 1)	0 (0 - 0)
Equatorial	Group B Strep	33 (21 - 50)	4 (3 - 6)	5 (1 - 11)	1 (0 - 1)
Equatorial	Haemophilus influenzae	4 (3 - 7)	1 (0 - 1)	1 (0 - 2)	0 (0 - 0)
Equatorial	Klebsiella pneumoniae	90 (60 - 132)	14 (10 - 20)	22 (13 - 37)	4 (2 - 6)
Equatorial	Morganella spp	0 (0 - 0)	0 (0 - 0)	0 (0 - 0)	0 (0 - 0)
Equatorial	Mycobacterium tuberculosis	22 (7 - 54)	4 (1 - 9)	8 (1 - 29)	1 (0 - 5)
Equatorial	Neisseria Gonorrheae				
Equatorial	Non Typhoidal Salmonellae	0 (0 - 0)	0 (0 - 0)	0 (0 - 0)	0 (0 - 0)
Equatorial	Proteus spp	8 (5 - 13)	2 (1 - 3)	1 (1 - 2)	0 (0 - 0)
Equatorial	Pseudomonas aeruginosa	39 (25 - 57)	7 (4 - 10)	10 (6 - 15)	2 (1 - 3)
Equatorial	Salmonella Paratyphi	0 (0 - 0)	0 (0 - 0)	0 (0 - 0)	0 (0 - 0)
Equatorial	Salmonella Typhi	10 (4 - 18)	1 (0 - 1)	2 (0 - 4)	0 (0 - 0)
Equatorial	Serratia spp	11 (7 - 17)	2 (1 - 2)	3 (1 - 4)	0 (0 - 1)
Equatorial	Shigella spp	0 (0 - 1)	0 (0 - 0)	0 (0 - 0)	0 (0 - 0)
Equatorial	Staphylococcus Aureus	60 (39 - 88)	11 (7 - 15)	15 (8 - 24)	3 (1 - 4)
Equatorial	Streptococcus pneumoniae	114 (74 - 170)	16 (11 - 22)	24 (14 - 37)	3 (2 - 5)
Eritrea	Acinetobacter baumannii	386 (230 - 624)	17 (10 - 28)	114 (62 - 190)	5 (3 - 8)
Eritrea	All	6,600 (4,700 - 9,300)	204 (146 - 282)	1,600 (1,100 - 2,500)	50 (34 - 77)
Eritrea	Citrobacter spp	28 (16 - 48)	1 (1 - 2)	8 (4 - 16)	0 (0 - 1)
Eritrea	Enterobacter spp	173 (110 - 258)	6 (4 - 9)	42 (26 - 65)	2 (1 - 2)
Eritrea	Enterococcus faecalis	107 (59 - 176)	4 (2 - 6)	28 (13 - 51)	1 (0 - 2)
Eritrea	Enterococcus faecium	110 (60 - 188)	4 (2 - 7)	27 (12 - 53)	1 (0 - 2)
Eritrea	Enterococcus spp	28 (16 - 47)	1 (1 - 2)	6 (1 - 12)	0 (0 - 1)
Eritrea	Escherichia coli	885 (629 - 1,200)	28 (20 - 39)	218 (145 - 319)	7 (5 - 10)
Eritrea	Group A Strep	33 (17 - 61)	1 (1 - 3)	3 (0 - 12)	0 (0 - 0)
Eritrea	Group B Strep	237 (157 - 351)	6 (4 - 9)	38 (9 - 76)	1 (0 - 2)
Eritrea	Haemophilus influenzae	103 (67 - 149)	3 (2 - 4)	21 (7 - 40)	1 (0 - 1)
Eritrea	Klebsiella pneumoniae	1,100 (758 - 1,600)	34 (24 - 48)	274 (165 - 439)	8 (5 - 14)
Eritrea	Morganella spp	1 (1 - 2)	0 (0 - 0)	0 (0 - 1)	0 (0 - 0)
Eritrea	Mycobacterium tuberculosis	642 (258 - 1,600)	19 (8 - 48)	281 (40 - 939)	8 (1 - 28)
Eritrea	Neisseria Gonorrheae				
Eritrea	Non Typhoidal Salmonellae	0 (0 - 1)	0 (0 - 0)	0 (0 - 0)	0 (0 - 0)
Eritrea	Proteus spp	69 (40 - 112)	3 (2 - 5)	10 (5 - 17)	0 (0 - 1)
Eritrea	Pseudomonas aeruginosa	299 (200 - 439)	10 (7 - 14)	78 (44 - 129)	3 (1 - 4)
Eritrea	Salmonella Paratyphi	0 (0 - 0)	0 (0 - 0)	0 (0 - 0)	0 (0 - 0)
Eritrea	Salmonella Typhi	255 (98 - 488)	4 (2 - 7)	44 (5 - 105)	1 (0 - 2)
Eritrea	Serratia spp	74 (43 - 118)	2 (1 - 3)	18 (9 - 32)	1 (0 - 1)
Eritrea	Shigella spp	31 (7 - 84)	1 (0 - 2)	6 (1 - 21)	0 (0 - 1)
Eritrea	Staphylococcus Aureus	954 (650 - 1,400)	33 (23 - 49)	169 (99 - 268)	6 (4 - 10)
Eritrea	Streptococcus pneumoniae	1,100 (753 - 1,600)	28 (19 - 41)	226 (126 - 354)	6 (3 - 9)
Ethiopia	Acinetobacter baumannii	4,100 (2,600 - 6,100)	9 (5 - 14)	1,300 (765 - 2,000)	3 (2 - 5)
Ethiopia	All	85,000 (66,000 - 109,000)	138 (108 - 176)	21,000 (16,000 - 28,000)	35 (27 - 47)
Ethiopia	Citrobacter spp	643 (405 - 973)	1 (1 - 2)	190 (106 - 309)	0 (0 - 1)
Ethiopia	Enterobacter spp	3,500 (2,500 - 4,800)	6 (4 - 8)	816 (535 - 1,200)	1 (1 - 2)
Ethiopia	Enterococcus faecalis	1,700 (1,000 - 2,500)	3 (2 - 5)	440 (215 - 766)	1 (0 - 2)
Ethiopia	Enterococcus faecium	1,800 (1,100 - 2,800)	4 (2 - 6)	456 (221 - 824)	1 (1 - 2)

Ethiopia	Enterococcus spp	773 (490 - 1,200)	1 (1 - 2)	161 (39 - 312)	0 (0 - 1)
Ethiopia	Escherichia coli	13,000 (9,900 - 17,000)	23 (17 - 29)	3,400 (2,400 - 4,800)	6 (4 - 8)
Ethiopia	Group A Strep	443 (252 - 741)	1 (0 - 2)	42 (0 - 152)	0 (0 - 0)
Ethiopia	Group B Strep	4,700 (3,500 - 6,400)	6 (4 - 8)	701 (109 - 1,400)	1 (0 - 2)
Ethiopia	Haemophilus influenzae	973 (694 - 1,300)	1 (1 - 2)	199 (64 - 350)	0 (0 - 0)
Ethiopia	Klebsiella pneumoniae	16,000 (12,000 - 21,000)	25 (19 - 32)	5,000 (3,500 - 6,900)	8 (5 - 11)
Ethiopia	Morganella spp	18 (9 - 30)	0 (0 - 0)	4 (2 - 8)	0 (0 - 0)
Ethiopia	Mycobacterium tuberculosis	2,700 (958 - 7,200)	6 (2 - 15)	1,400 (177 - 4,600)	3 (0 - 9)
Ethiopia	Neisseria Gonorrheae				
Ethiopia	Non Typhoidal Salmonellae	475 (175 - 1,000)	1 (0 - 1)	99 (12 - 271)	0 (0 - 0)
Ethiopia	Proteus spp	1,100 (691 - 1,600)	3 (2 - 4)	149 (83 - 240)	0 (0 - 1)
Ethiopia	Pseudomonas aeruginosa	3,900 (2,800 - 5,300)	7 (5 - 9)	1,000 (621 - 1,600)	2 (1 - 3)
Ethiopia	Salmonella Paratyphi	0 (0 - 0)	0 (0 - 0)	0 (0 - 0)	0 (0 - 0)
Ethiopia	Salmonella Typhi	2,500 (912 - 5,100)	2 (1 - 4)	446 (62 - 1,200)	0 (0 - 1)
Ethiopia	Serratia spp	1,100 (687 - 1,700)	2 (1 - 2)	259 (139 - 430)	0 (0 - 1)
Ethiopia	Shigella spp	714 (226 - 1,600)	1 (0 - 2)	148 (17 - 434)	0 (0 - 1)
Ethiopia	Staphylococcus Aureus	10,000 (7,900 - 13,000)	19 (15 - 24)	2,300 (1,400 - 3,300)	4 (3 - 6)
Ethiopia	Streptococcus pneumoniae	15,000 (12,000 - 19,000)	20 (17 - 24)	2,900 (1,700 - 4,200)	4 (2 - 5)
Gabon	Acinetobacter baumannii	131 (79 - 204)	14 (9 - 22)	40 (24 - 65)	4 (3 - 7)
Gabon	All	1,000 (739 - 1,400)	99 (72 - 136)	257 (179 - 376)	25 (18 - 36)
Gabon	Citrobacter spp	10 (6 - 16)	1 (1 - 2)	3 (2 - 5)	0 (0 - 0)
Gabon	Enterobacter spp	61 (40 - 90)	6 (4 - 8)	14 (8 - 21)	1 (1 - 2)
Gabon	Enterococcus faecalis	24 (14 - 38)	2 (1 - 4)	6 (3 - 12)	1 (0 - 1)
Gabon	Enterococcus faecium	32 (18 - 53)	3 (2 - 5)	8 (4 - 15)	1 (0 - 2)
Gabon	Enterococcus spp	16 (10 - 26)	2 (1 - 3)	3 (1 - 7)	0 (0 - 1)
Gabon	Escherichia coli	142 (100 - 195)	14 (10 - 20)	34 (23 - 49)	3 (2 - 5)
Gabon	Group A Strep	4 (2 - 9)	1 (0 - 1)	0 (0 - 2)	0 (0 - 0)
Gabon	Group B Strep	30 (21 - 43)	3 (2 - 4)	4 (1 - 9)	0 (0 - 1)
Gabon	Haemophilus influenzae	7 (5 - 11)	1 (0 - 1)	2 (1 - 3)	0 (0 - 0)
Gabon	Klebsiella pneumoniae	152 (110 - 211)	15 (11 - 20)	38 (23 - 61)	4 (2 - 6)
Gabon	Morganella spp	0 (0 - 1)	0 (0 - 0)	0 (0 - 0)	0 (0 - 0)
Gabon	Mycobacterium tuberculosis	45 (14 - 124)	4 (1 - 11)	21 (2 - 79)	2 (0 - 7)
Gabon	Neisseria Gonorrheae				
Gabon	Non Typhoidal Salmonellae	0 (0 - 0)	0 (0 - 0)	0 (0 - 0)	0 (0 - 0)
Gabon	Proteus spp	16 (10 - 25)	2 (1 - 3)	2 (1 - 3)	0 (0 - 0)
Gabon	Pseudomonas aeruginosa	61 (41 - 87)	6 (4 - 9)	15 (10 - 23)	2 (1 - 2)
Gabon	Salmonella Paratyphi	0 (0 - 0)	0 (0 - 0)	0 (0 - 0)	0 (0 - 0)
Gabon	Salmonella Typhi	9 (3 - 18)	1 (0 - 1)	2 (0 - 4)	0 (0 - 0)
Gabon	Serratia spp	16 (10 - 25)	1 (1 - 2)	4 (2 - 7)	0 (0 - 1)
Gabon	Shigella spp	0 (0 - 1)	0 (0 - 0)	0 (0 - 0)	0 (0 - 0)
Gabon	Staphylococcus Aureus	104 (75 - 145)	10 (8 - 14)	28 (16 - 44)	3 (2 - 4)
Gabon	Streptococcus pneumoniae	161 (122 - 212)	14 (11 - 18)	32 (20 - 45)	3 (2 - 4)
Ghana	Acinetobacter baumannii	1,500 (906 - 2,300)	10 (6 - 16)	420 (246 - 655)	3 (2 - 5)
Ghana	All	25,000 (19,000 - 33,000)	138 (107 - 180)	5,900 (4,300 - 8,100)	33 (24 - 44)
Ghana	Citrobacter spp	148 (89 - 227)	1 (0 - 1)	44 (24 - 75)	0 (0 - 0)
Ghana	Enterobacter spp	831 (577 - 1,200)	5 (3 - 7)	207 (137 - 303)	1 (1 - 2)
Ghana	Enterococcus faecalis	449 (268 - 691)	2 (1 - 4)	119 (58 - 213)	1 (0 - 1)
Ghana	Enterococcus faecium	658 (399 - 1,100)	4 (2 - 6)	164 (75 - 292)	1 (0 - 2)
Ghana	Enterococcus spp	255 (158 - 406)	1 (1 - 2)	52 (11 - 107)	0 (0 - 1)
Ghana	Escherichia coli	3,600 (2,600 - 4,800)	21 (16 - 28)	934 (617 - 1,300)	6 (4 - 8)
Ghana	Group A Strep	97 (51 - 174)	1 (0 - 1)	9 (0 - 33)	0 (0 - 0)
Ghana	Group B Strep	674 (482 - 906)	3 (2 - 4)	101 (14 - 204)	1 (0 - 1)
Ghana	Haemophilus influenzae	125 (88 - 173)	1 (0 - 1)	29 (13 - 50)	0 (0 - 0)
Ghana	Klebsiella pneumoniae	4,600 (3,500 - 6,000)	25 (19 - 32)	1,200 (739 - 1,800)	6 (4 - 9)
Ghana	Morganella spp	4 (2 - 7)	0 (0 - 0)	1 (0 - 2)	0 (0 - 0)
Ghana	Mycobacterium tuberculosis	786 (217 - 2,100)	5 (1 - 12)	391 (32 - 1,400)	2 (0 - 8)
Ghana	Neisseria Gonorrheae				

Ghana	Non Typhoidal Salmonellae	0 (0 - 0)	0 (0 - 0)	0 (0 - 0)	0 (0 - 0)
Ghana	Proteus spp	310 (189 - 474)	2 (1 - 3)	59 (30 - 100)	0 (0 - 1)
Ghana	Pseudomonas aeruginosa	1,600 (1,200 - 2,200)	9 (7 - 12)	336 (226 - 503)	2 (1 - 3)
Ghana	Salmonella Paratyphi	2 (0 - 7)	0 (0 - 0)	0 (0 - 1)	0 (0 - 0)
Ghana	Salmonella Typhi	1,400 (728 - 2,300)	4 (2 - 7)	190 (0 - 478)	1 (0 - 1)
Ghana	Serratia spp	483 (305 - 725)	2 (1 - 4)	136 (74 - 226)	1 (0 - 1)
Ghana	Shigella spp	3 (1 - 8)	0 (0 - 0)	1 (0 - 2)	0 (0 - 0)
Ghana	Staphylococcus Aureus	3,700 (2,900 - 4,700)	22 (18 - 28)	792 (521 - 1,200)	5 (3 - 7)
Ghana	Streptococcus pneumoniae	4,200 (3,200 - 5,200)	21 (17 - 25)	748 (433 - 1,100)	4 (2 - 5)
Guinea	Acinetobacter baumannii	1,000 (629 - 1,500)	15 (9 - 22)	302 (181 - 458)	4 (3 - 7)
Guinea	All	17,000 (13,000 - 23,000)	172 (126 - 226)	4,000 (2,800 - 5,600)	41 (29 - 56)
Guinea	Citrobacter spp	179 (102 - 282)	2 (1 - 3)	55 (29 - 91)	1 (0 - 1)
Guinea	Enterobacter spp	791 (535 - 1,100)	8 (6 - 12)	194 (127 - 287)	2 (1 - 3)
Guinea	Enterococcus faecalis	253 (149 - 403)	3 (2 - 5)	67 (32 - 117)	1 (0 - 2)
Guinea	Enterococcus faecium	225 (129 - 387)	3 (2 - 6)	56 (24 - 108)	1 (0 - 2)
Guinea	Enterococcus spp	154 (96 - 244)	2 (1 - 3)	32 (7 - 64)	0 (0 - 1)
Guinea	Escherichia coli	2,000 (1,400 - 2,700)	21 (16 - 28)	492 (334 - 681)	5 (4 - 8)
Guinea	Group A Strep	62 (32 - 110)	1 (0 - 2)	6 (0 - 21)	0 (0 - 0)
Guinea	Group B Strep	1,300 (945 - 1,900)	10 (7 - 14)	247 (76 - 477)	2 (1 - 4)
Guinea	Haemophilus influenzae	372 (254 - 521)	3 (2 - 4)	86 (39 - 144)	1 (0 - 1)
Guinea	Klebsiella pneumoniae	2,700 (2,000 - 3,600)	27 (20 - 36)	720 (437 - 1,100)	7 (4 - 11)
Guinea	Morganella spp	3 (2 - 6)	0 (0 - 0)	1 (0 - 1)	0 (0 - 0)
Guinea	Mycobacterium tuberculosis	370 (126 - 939)	6 (2 - 14)	157 (13 - 523)	2 (0 - 8)
Guinea	Neisseria Gonorrheae				
Guinea	Non Typhoidal Salmonellae	2 (0 - 4)	0 (0 - 0)	0 (0 - 1)	0 (0 - 0)
Guinea	Proteus spp	151 (90 - 242)	3 (2 - 4)	21 (11 - 37)	0 (0 - 1)
Guinea	Pseudomonas aeruginosa	747 (525 - 1,000)	8 (6 - 12)	187 (117 - 287)	2 (1 - 3)
Guinea	Salmonella Paratyphi	0 (0 - 0)	0 (0 - 0)	0 (0 - 0)	0 (0 - 0)
Guinea	Salmonella Typhi	653 (359 - 1,000)	4 (2 - 7)	92 (0 - 222)	1 (0 - 1)
Guinea	Serratia spp	318 (198 - 509)	3 (2 - 6)	82 (42 - 141)	1 (1 - 2)
Guinea	Shigella spp	10 (2 - 28)	0 (0 - 0)	2 (0 - 7)	0 (0 - 0)
Guinea	Staphylococcus Aureus	1,400 (1,000 - 1,900)	16 (12 - 22)	296 (183 - 442)	3 (2 - 5)
Guinea	Streptococcus pneumoniae	4,700 (3,300 - 6,200)	36 (27 - 47)	905 (509 - 1,400)	7 (4 - 10)
Guinea-Bissau	Acinetobacter baumannii	102 (60 - 158)	15 (9 - 23)	29 (16 - 46)	4 (2 - 7)
Guinea-Bissau	All	1,800 (1,400 - 2,400)	180 (136 - 235)	413 (300 - 556)	42 (30 - 57)
Guinea-Bissau	Citrobacter spp	10 (6 - 16)	1 (1 - 2)	3 (2 - 5)	0 (0 - 1)
Guinea-Bissau	Enterobacter spp	61 (41 - 89)	7 (4 - 10)	15 (10 - 22)	2 (1 - 2)
Guinea-Bissau	Enterococcus faecalis	33 (20 - 51)	3 (2 - 6)	9 (4 - 16)	1 (0 - 2)
Guinea-Bissau	Enterococcus faecium	34 (19 - 57)	4 (2 - 7)	9 (4 - 16)	1 (1 - 2)
Guinea-Bissau	Enterococcus spp	12 (7 - 18)	1 (1 - 2)	2 (1 - 5)	0 (0 - 1)
Guinea-Bissau	Escherichia coli	260 (191 - 347)	26 (20 - 35)	63 (43 - 88)	6 (4 - 9)
Guinea-Bissau	Group A Strep	7 (4 - 13)	1 (0 - 2)	1 (0 - 2)	0 (0 - 0)
Guinea-Bissau	Group B Strep	97 (70 - 134)	7 (5 - 9)	16 (4 - 32)	1 (0 - 2)
Guinea-Bissau	Haemophilus influenzae	35 (24 - 46)	3 (2 - 3)	8 (3 - 13)	1 (0 - 1)
Guinea-Bissau	Klebsiella pneumoniae	350 (261 - 461)	33 (25 - 44)	89 (55 - 135)	8 (5 - 13)
Guinea-Bissau	Morganella spp	0 (0 - 1)	0 (0 - 0)	0 (0 - 0)	0 (0 - 0)
Guinea-Bissau	Mycobacterium tuberculosis	50 (18 - 126)	6 (2 - 14)	21 (2 - 72)	2 (0 - 8)
Guinea-Bissau	Neisseria Gonorrheae				
Guinea-Bissau	Non Typhoidal Salmonellae	0 (0 - 0)	0 (0 - 0)	0 (0 - 0)	0 (0 - 0)
Guinea-Bissau	Proteus spp	18 (10 - 29)	3 (1 - 4)	2 (1 - 4)	0 (0 - 1)
Guinea-Bissau	Pseudomonas aeruginosa	81 (57 - 111)	8 (6 - 12)	20 (13 - 31)	2 (1 - 3)
Guinea-Bissau	Salmonella Paratyphi	0 (0 - 0)	0 (0 - 0)	0 (0 - 0)	0 (0 - 0)
Guinea-Bissau	Salmonella Typhi	40 (13 - 90)	2 (1 - 4)	6 (0 - 19)	0 (0 - 1)
Guinea-Bissau	Serratia spp	33 (20 - 51)	3 (2 - 5)	9 (5 - 15)	1 (0 - 1)
Guinea-Bissau	Shigella spp	3 (1 - 8)	0 (0 - 1)	1 (0 - 2)	0 (0 - 0)
Guinea-Bissau	Staphylococcus Aureus	272 (206 - 352)	31 (24 - 39)	48 (32 - 69)	5 (4 - 8)
Guinea-Bissau	Streptococcus pneumoniae	325 (249 - 422)	27 (22 - 35)	63 (38 - 92)	5 (3 - 8)

Kenya	Acinetobacter baumannii	2,000 (1,200 - 3,000)	10 (6 - 16)	617 (364 - 962)	3 (2 - 5)
Kenya	All	37,000 (30,000 - 47,000)	149 (117 - 189)	8,500 (6,600 - 11,000)	35 (26 - 45)
Kenya	Citrobacter spp	237 (143 - 375)	1 (1 - 2)	71 (39 - 121)	0 (0 - 1)
Kenya	Enterobacter spp	1,300 (896 - 1,900)	6 (4 - 8)	317 (200 - 475)	1 (1 - 2)
Kenya	Enterococcus faecalis	939 (547 - 1,500)	4 (2 - 7)	245 (117 - 440)	1 (1 - 2)
Kenya	Enterococcus faecium	1,000 (581 - 1,700)	5 (3 - 8)	258 (119 - 493)	1 (1 - 2)
Kenya	Enterococcus spp	313 (197 - 471)	1 (1 - 2)	64 (13 - 128)	0 (0 - 1)
Kenya	Escherichia coli	5,900 (4,500 - 7,700)	25 (19 - 33)	1,400 (987 - 2,000)	6 (4 - 9)
Kenya	Group A Strep	158 (85 - 281)	1 (0 - 1)	15 (0 - 55)	0 (0 - 0)
Kenya	Group B Strep	1,400 (1,100 - 1,900)	5 (4 - 6)	229 (46 - 435)	1 (0 - 1)
Kenya	Haemophilus influenzae	412 (305 - 542)	1 (1 - 2)	85 (29 - 153)	0 (0 - 1)
Kenya	Klebsiella pneumoniae	6,200 (4,800 - 8,000)	25 (19 - 33)	1,600 (1,000 - 2,300)	6 (4 - 9)
Kenya	Morganella spp	9 (5 - 14)	0 (0 - 0)	2 (1 - 4)	0 (0 - 0)
Kenya	Mycobacterium tuberculosis	1,400 (775 - 2,300)	5 (3 - 9)	496 (99 - 1,200)	2 (0 - 5)
Kenya	Neisseria Gonorrheae				
Kenya	Non Typhoidal Salmonellae	4 (1 - 8)	0 (0 - 0)	1 (0 - 2)	0 (0 - 0)
Kenya	Proteus spp	565 (353 - 855)	3 (2 - 5)	77 (41 - 128)	0 (0 - 1)
Kenya	Pseudomonas aeruginosa	1,800 (1,300 - 2,400)	8 (6 - 10)	469 (309 - 695)	2 (1 - 3)
Kenya	Salmonella Paratyphi	4 (1 - 8)	0 (0 - 0)	1 (0 - 2)	0 (0 - 0)
Kenya	Salmonella Typhi	2,400 (1,500 - 3,600)	5 (3 - 7)	430 (136 - 846)	1 (0 - 2)
Kenya	Serratia spp	444 (287 - 675)	2 (1 - 3)	104 (58 - 171)	0 (0 - 1)
Kenya	Shigella spp	213 (71 - 452)	1 (0 - 2)	45 (4 - 121)	0 (0 - 0)
Kenya	Staphylococcus Aureus	5,000 (4,000 - 6,200)	22 (18 - 28)	883 (589 - 1,300)	4 (3 - 6)
Kenya	Streptococcus pneumoniae	5,600 (4,600 - 6,800)	19 (16 - 23)	1,200 (756 - 1,600)	4 (3 - 6)
Lesotho	Acinetobacter baumannii	223 (128 - 358)	21 (12 - 34)	68 (37 - 110)	6 (4 - 10)
Lesotho	All	2,800 (2,100 - 3,900)	213 (153 - 292)	725 (469 - 1,100)	54 (35 - 81)
Lesotho	Citrobacter spp	7 (4 - 11)	1 (0 - 1)	2 (1 - 4)	0 (0 - 0)
Lesotho	Enterobacter spp	56 (37 - 84)	4 (3 - 7)	11 (7 - 17)	1 (1 - 1)
Lesotho	Enterococcus faecalis	44 (24 - 72)	3 (2 - 6)	12 (5 - 21)	1 (0 - 2)
Lesotho	Enterococcus faecium	60 (35 - 101)	5 (3 - 8)	15 (7 - 28)	1 (1 - 2)
Lesotho	Enterococcus spp	19 (10 - 33)	2 (1 - 3)	4 (1 - 8)	0 (0 - 1)
Lesotho	Escherichia coli	342 (240 - 472)	27 (19 - 38)	76 (49 - 111)	6 (4 - 9)
Lesotho	Group A Strep	8 (3 - 15)	1 (0 - 1)	1 (0 - 3)	0 (0 - 0)
Lesotho	Group B Strep	85 (57 - 120)	6 (4 - 8)	13 (2 - 26)	1 (0 - 2)
Lesotho	Haemophilus influenzae	26 (18 - 36)	2 (1 - 2)	5 (1 - 10)	0 (0 - 1)
Lesotho	Klebsiella pneumoniae	473 (339 - 656)	36 (25 - 50)	128 (74 - 204)	10 (6 - 16)
Lesotho	Morganella spp	1 (0 - 1)	0 (0 - 0)	0 (0 - 0)	0 (0 - 0)
Lesotho	Mycobacterium tuberculosis	403 (202 - 772)	26 (13 - 49)	178 (36 - 488)	11 (2 - 31)
Lesotho	Neisseria Gonorrheae				
Lesotho	Non Typhoidal Salmonellae	0 (0 - 1)	0 (0 - 0)	0 (0 - 0)	0 (0 - 0)
Lesotho	Proteus spp	33 (19 - 54)	3 (2 - 5)	4 (2 - 7)	0 (0 - 1)
Lesotho	Pseudomonas aeruginosa	153 (106 - 222)	12 (8 - 18)	38 (25 - 59)	3 (2 - 5)
Lesotho	Salmonella Paratyphi	0 (0 - 0)	0 (0 - 0)	0 (0 - 0)	0 (0 - 0)
Lesotho	Salmonella Typhi	65 (23 - 122)	4 (1 - 7)	11 (1 - 26)	1 (0 - 1)
Lesotho	Serratia spp	20 (12 - 33)	2 (1 - 2)	4 (2 - 7)	0 (0 - 1)
Lesotho	Shigella spp	10 (3 - 25)	1 (0 - 2)	2 (0 - 6)	0 (0 - 1)
Lesotho	Staphylococcus Aureus	424 (315 - 563)	33 (25 - 44)	71 (47 - 104)	6 (4 - 8)
Lesotho	Streptococcus pneumoniae	399 (308 - 503)	26 (20 - 33)	81 (53 - 114)	5 (4 - 8)
Liberia	Acinetobacter baumannii	198 (119 - 299)	10 (6 - 16)	56 (32 - 88)	3 (2 - 5)
Liberia	All	3,400 (2,500 - 4,500)	128 (96 - 172)	772 (552 - 1,100)	30 (22 - 41)
Liberia	Citrobacter spp	23 (14 - 36)	1 (1 - 1)	7 (3 - 12)	0 (0 - 0)
Liberia	Enterobacter spp	117 (81 - 169)	5 (3 - 7)	28 (18 - 43)	1 (1 - 2)
Liberia	Enterococcus faecalis	64 (38 - 99)	2 (1 - 4)	17 (8 - 30)	1 (0 - 1)
Liberia	Enterococcus faecium	87 (51 - 140)	4 (2 - 6)	22 (10 - 40)	1 (0 - 2)
Liberia	Enterococcus spp	31 (20 - 48)	1 (1 - 2)	6 (1 - 13)	0 (0 - 1)
Liberia	Escherichia coli	533 (383 - 727)	21 (15 - 29)	129 (89 - 183)	5 (3 - 7)
Liberia	Group A Strep	15 (8 - 27)	1 (0 - 1)	1 (0 - 5)	0 (0 - 0)

Liberia	Group B Strep	112 (81 - 156)	3 (3 - 5)	17 (3 - 33)	1 (0 - 1)
Liberia	Haemophilus influenzae	24 (16 - 34)	1 (1 - 1)	5 (2 - 9)	0 (0 - 0)
Liberia	Klebsiella pneumoniae	669 (494 - 896)	24 (18 - 33)	173 (107 - 266)	6 (4 - 10)
Liberia	Morganella spp	1 (0 - 1)	0 (0 - 0)	0 (0 - 0)	0 (0 - 0)
Liberia	Mycobacterium tuberculosis	68 (20 - 215)	3 (1 - 9)	32 (2 - 135)	1 (0 - 6)
Liberia	Neisseria Gonorrheae				
Liberia	Non Typhoidal Salmonellae	0 (0 - 0)	0 (0 - 0)	0 (0 - 0)	0 (0 - 0)
Liberia	Proteus spp	44 (26 - 68)	2 (1 - 4)	6 (3 - 10)	0 (0 - 1)
Liberia	Pseudomonas aeruginosa	215 (153 - 293)	8 (6 - 12)	51 (32 - 77)	2 (1 - 3)
Liberia	Salmonella Paratyphi	0 (0 - 0)	0 (0 - 0)	0 (0 - 0)	0 (0 - 0)
Liberia	Salmonella Typhi	184 (90 - 307)	4 (2 - 6)	26 (0 - 62)	1 (0 - 1)
Liberia	Serratia spp	69 (45 - 103)	2 (1 - 3)	19 (10 - 30)	1 (0 - 1)
Liberia	Shigella spp	2 (0 - 4)	0 (0 - 0)	0 (0 - 1)	0 (0 - 0)
Liberia	Staphylococcus Aureus	464 (346 - 612)	20 (15 - 26)	86 (55 - 127)	4 (2 - 5)
Liberia	Streptococcus pneumoniae	503 (369 - 672)	16 (13 - 21)	91 (49 - 146)	3 (2 - 5)
Madagascar	Acinetobacter baumannii	1,400 (819 - 2,200)	15 (8 - 23)	405 (227 - 647)	4 (2 - 7)
Madagascar	All	23,000 (17,000 - 30,000)	155 (113 - 207)	5,400 (3,800 - 7,400)	37 (26 - 53)
Madagascar	Citrobacter spp	113 (66 - 178)	1 (0 - 1)	33 (18 - 56)	0 (0 - 0)
Madagascar	Enterobacter spp	675 (450 - 974)	5 (3 - 8)	160 (100 - 238)	1 (1 - 2)
Madagascar	Enterococcus faecalis	471 (287 - 727)	4 (2 - 6)	123 (60 - 212)	1 (0 - 2)
Madagascar	Enterococcus faecium	351 (201 - 593)	3 (2 - 5)	90 (42 - 174)	1 (0 - 2)
Madagascar	Enterococcus spp	113 (67 - 186)	1 (1 - 2)	24 (5 - 49)	0 (0 - 0)
Madagascar	Escherichia coli	3,400 (2,500 - 4,500)	22 (16 - 30)	787 (559 - 1,100)	5 (4 - 7)
Madagascar	Group A Strep	180 (99 - 328)	2 (1 - 3)	17 (0 - 61)	0 (0 - 1)
Madagascar	Group B Strep	1,300 (896 - 1,700)	6 (4 - 8)	179 (18 - 361)	1 (0 - 2)
Madagascar	Haemophilus influenzae	493 (360 - 659)	3 (2 - 3)	97 (25 - 171)	1 (0 - 1)
Madagascar	Klebsiella pneumoniae	3,800 (2,800 - 4,900)	25 (18 - 34)	958 (591 - 1,400)	6 (4 - 10)
Madagascar	Morganella spp	4 (2 - 7)	0 (0 - 0)	1 (0 - 2)	0 (0 - 0)
Madagascar	Mycobacterium tuberculosis	888 (243 - 2,700)	7 (2 - 20)	463 (39 - 1,700)	4 (0 - 13)
Madagascar	Neisseria Gonorrheae				
Madagascar	Non Typhoidal Salmonellae	4 (1 - 14)	0 (0 - 0)	1 (0 - 4)	0 (0 - 0)
Madagascar	Proteus spp	248 (149 - 389)	3 (2 - 4)	29 (15 - 51)	0 (0 - 1)
Madagascar	Pseudomonas aeruginosa	1,400 (1,000 - 1,900)	10 (7 - 14)	362 (216 - 569)	3 (2 - 4)
Madagascar	Salmonella Paratyphi	0 (0 - 0)	0 (0 - 0)	0 (0 - 0)	0 (0 - 0)
Madagascar	Salmonella Typhi	592 (187 - 1,300)	2 (1 - 5)	100 (6 - 285)	0 (0 - 1)
Madagascar	Serratia spp	345 (216 - 527)	2 (1 - 3)	91 (50 - 151)	1 (0 - 1)
Madagascar	Shigella spp	12 (3 - 35)	0 (0 - 0)	3 (0 - 9)	0 (0 - 0)
Madagascar	Staphylococcus Aureus	3,100 (2,300 - 4,000)	24 (18 - 31)	570 (368 - 852)	4 (3 - 7)
Madagascar	Streptococcus pneumoniae	4,100 (3,200 - 5,300)	23 (18 - 29)	896 (540 - 1,300)	5 (3 - 7)
Malawi	Acinetobacter baumannii	874 (528 - 1,300)	12 (7 - 19)	262 (148 - 421)	4 (2 - 6)
Malawi	All	16,000 (12,000 - 20,000)	153 (118 - 196)	3,600 (2,700 - 4,800)	36 (27 - 47)
Malawi	Citrobacter spp	90 (54 - 140)	1 (1 - 1)	27 (14 - 48)	0 (0 - 1)
Malawi	Enterobacter spp	466 (326 - 669)	5 (4 - 7)	112 (73 - 165)	1 (1 - 2)
Malawi	Enterococcus faecalis	349 (212 - 525)	4 (2 - 6)	91 (44 - 157)	1 (0 - 2)
Malawi	Enterococcus faecium	305 (185 - 501)	4 (2 - 6)	78 (38 - 142)	1 (0 - 2)
Malawi	Enterococcus spp	103 (62 - 167)	1 (1 - 2)	21 (5 - 45)	0 (0 - 1)
Malawi	Escherichia coli	2,400 (1,800 - 3,100)	25 (19 - 32)	557 (401 - 745)	6 (4 - 8)
Malawi	Group A Strep	127 (69 - 223)	1 (1 - 3)	12 (0 - 43)	0 (0 - 1)
Malawi	Group B Strep	477 (345 - 657)	4 (3 - 5)	79 (19 - 153)	1 (0 - 1)
Malawi	Haemophilus influenzae	270 (202 - 356)	2 (2 - 2)	54 (15 - 98)	0 (0 - 1)
Malawi	Klebsiella pneumoniae	2,800 (2,100 - 3,700)	27 (20 - 35)	751 (455 - 1,100)	7 (4 - 11)
Malawi	Morganella spp	3 (2 - 5)	0 (0 - 0)	1 (0 - 1)	0 (0 - 0)
Malawi	Mycobacterium tuberculosis	523 (206 - 1,200)	6 (2 - 14)	245 (38 - 790)	3 (0 - 9)
Malawi	Neisseria Gonorrheae				
Malawi	Non Typhoidal Salmonellae	0 (0 - 1)	0 (0 - 0)	0 (0 - 0)	0 (0 - 0)
Malawi	Proteus spp	185 (115 - 283)	3 (2 - 4)	30 (16 - 50)	0 (0 - 1)
Malawi	Pseudomonas aeruginosa	786 (568 - 1,000)	8 (6 - 11)	201 (124 - 321)	2 (1 - 3)

Malawi	Salmonella Paratyphi	0 (0 - 0)	0 (0 - 0)	0 (0 - 0)	0 (0 - 0)
Malawi	Salmonella Typhi	893 (582 - 1,300)	5 (3 - 7)	134 (0 - 290)	1 (0 - 2)
Malawi	Serratia spp	184 (114 - 281)	2 (1 - 3)	39 (20 - 66)	0 (0 - 1)
Malawi	Shigella spp	15 (4 - 43)	0 (0 - 0)	3 (0 - 11)	0 (0 - 0)
Malawi	Staphylococcus Aureus	2,000 (1,600 - 2,600)	22 (18 - 28)	380 (252 - 543)	4 (3 - 6)
Malawi	Streptococcus pneumoniae	2,800 (2,300 - 3,600)	22 (18 - 27)	530 (335 - 782)	4 (3 - 6)
Mali	Acinetobacter baumannii	1,200 (771 - 1,800)	10 (6 - 16)	355 (211 - 579)	3 (2 - 5)
Mali	All	30,000 (22,000 - 40,000)	150 (109 - 203)	7,100 (5,100 - 9,600)	37 (26 - 51)
Mali	Citrobacter spp	233 (132 - 368)	1 (1 - 2)	71 (38 - 120)	0 (0 - 1)
Mali	Enterobacter spp	1,000 (696 - 1,400)	6 (4 - 8)	247 (163 - 362)	1 (1 - 2)
Mali	Enterococcus faecalis	574 (361 - 878)	4 (2 - 6)	152 (73 - 256)	1 (0 - 2)
Mali	Enterococcus faecium	512 (308 - 835)	4 (2 - 7)	127 (61 - 232)	1 (1 - 2)
Mali	Enterococcus spp	321 (199 - 504)	2 (1 - 3)	66 (14 - 129)	0 (0 - 1)
Mali	Escherichia coli	4,400 (3,200 - 6,000)	25 (18 - 33)	1,100 (763 - 1,600)	6 (4 - 9)
Mali	Group A Strep	120 (70 - 193)	1 (0 - 2)	11 (0 - 38)	0 (0 - 0)
Mali	Group B Strep	1,900 (1,300 - 2,600)	7 (5 - 10)	343 (99 - 661)	1 (0 - 2)
Mali	Haemophilus influenzae	362 (247 - 512)	1 (1 - 2)	90 (40 - 149)	0 (0 - 1)
Mali	Klebsiella pneumoniae	6,500 (4,800 - 8,800)	31 (22 - 42)	1,800 (1,100 - 2,700)	8 (5 - 13)
Mali	Morganella spp	4 (2 - 7)	0 (0 - 0)	1 (0 - 2)	0 (0 - 0)
Mali	Mycobacterium tuberculosis	491 (154 - 1,300)	5 (2 - 12)	227 (22 - 833)	2 (0 - 8)
Mali	Neisseria Gonorrheae				
Mali	Non Typhoidal Salmonellae	2 (1 - 5)	0 (0 - 0)	0 (0 - 1)	0 (0 - 0)
Mali	Proteus spp	282 (175 - 430)	3 (2 - 4)	40 (21 - 69)	0 (0 - 1)
Mali	Pseudomonas aeruginosa	1,600 (1,100 - 2,200)	8 (6 - 12)	382 (250 - 572)	2 (1 - 3)
Mali	Salmonella Paratyphi	0 (0 - 1)	0 (0 - 0)	0 (0 - 0)	0 (0 - 0)
Mali	Salmonella Typhi	1,800 (896 - 3,000)	6 (3 - 9)	258 (0 - 614)	1 (0 - 2)
Mali	Serratia spp	634 (397 - 992)	3 (2 - 5)	164 (89 - 277)	1 (0 - 1)
Mali	Shigella spp	32 (7 - 90)	0 (0 - 1)	7 (1 - 22)	0 (0 - 0)
Mali	Staphylococcus Aureus	3,500 (2,500 - 4,700)	19 (14 - 26)	789 (480 - 1,200)	4 (3 - 7)
Mali	Streptococcus pneumoniae	4,200 (3,100 - 5,700)	16 (12 - 21)	842 (503 - 1,300)	3 (2 - 5)
Mauritania	Acinetobacter baumannii	153 (91 - 238)	7 (4 - 12)	45 (26 - 71)	2 (1 - 3)
Mauritania	All	2,600 (1,800 - 3,600)	106 (77 - 143)	609 (421 - 851)	25 (18 - 34)
Mauritania	Citrobacter spp	18 (11 - 29)	1 (0 - 1)	5 (3 - 9)	0 (0 - 0)
Mauritania	Enterobacter spp	110 (74 - 159)	4 (3 - 6)	26 (17 - 38)	1 (1 - 2)
Mauritania	Enterococcus faecalis	51 (29 - 81)	2 (1 - 3)	14 (6 - 24)	1 (0 - 1)
Mauritania	Enterococcus faecium	69 (40 - 111)	3 (2 - 5)	17 (8 - 32)	1 (0 - 1)
Mauritania	Enterococcus spp	32 (20 - 50)	1 (1 - 2)	7 (2 - 13)	0 (0 - 1)
Mauritania	Escherichia coli	416 (290 - 578)	17 (12 - 24)	98 (65 - 141)	4 (3 - 6)
Mauritania	Group A Strep	10 (5 - 18)	0 (0 - 1)	1 (0 - 3)	0 (0 - 0)
Mauritania	Group B Strep	113 (79 - 156)	4 (3 - 5)	18 (4 - 36)	1 (0 - 1)
Mauritania	Haemophilus influenzae	16 (11 - 23)	1 (0 - 1)	4 (2 - 6)	0 (0 - 0)
Mauritania	Klebsiella pneumoniae	476 (332 - 655)	19 (14 - 26)	124 (72 - 188)	5 (3 - 7)
Mauritania	Morganella spp	1 (0 - 1)	0 (0 - 0)	0 (0 - 0)	0 (0 - 0)
Mauritania	Mycobacterium tuberculosis	38 (12 - 102)	2 (1 - 5)	18 (2 - 60)	1 (0 - 3)
Mauritania	Neisseria Gonorrheae				
Mauritania	Non Typhoidal Salmonellae	0 (0 - 0)	0 (0 - 0)	0 (0 - 0)	0 (0 - 0)
Mauritania	Proteus spp	35 (21 - 54)	2 (1 - 3)	5 (3 - 9)	0 (0 - 0)
Mauritania	Pseudomonas aeruginosa	180 (125 - 249)	8 (5 - 10)	42 (27 - 63)	2 (1 - 3)
Mauritania	Salmonella Paratyphi	0 (0 - 0)	0 (0 - 0)	0 (0 - 0)	0 (0 - 0)
Mauritania	Salmonella Typhi	71 (29 - 136)	2 (1 - 3)	11 (0 - 27)	0 (0 - 1)
Mauritania	Serratia spp	45 (28 - 70)	2 (1 - 3)	12 (6 - 20)	0 (0 - 1)
Mauritania	Shigella spp	7 (2 - 19)	0 (0 - 1)	1 (0 - 5)	0 (0 - 0)
Mauritania	Staphylococcus Aureus	376 (271 - 503)	17 (13 - 22)	90 (55 - 136)	4 (3 - 6)
Mauritania	Streptococcus pneumoniae	384 (267 - 545)	15 (11 - 19)	72 (39 - 117)	3 (2 - 4)
Mauritius	Acinetobacter baumannii	77 (41 - 128)	5 (3 - 8)	24 (12 - 41)	2 (1 - 3)
Mauritius	All	772 (481 - 1,200)	50 (31 - 74)	203 (124 - 312)	13 (8 - 20)
Mauritius	Citrobacter spp	5 (2 - 7)	0 (0 - 1)	1 (1 - 2)	0 (0 - 0)

Mauritius	Enterobacter spp	25 (14 - 38)	2 (1 - 3)	5 (3 - 9)	0 (0 - 1)
Mauritius	Enterococcus faecalis	19 (10 - 31)	1 (1 - 2)	5 (2 - 9)	0 (0 - 1)
Mauritius	Enterococcus faecium	43 (24 - 68)	3 (1 - 4)	11 (5 - 19)	1 (0 - 1)
Mauritius	Enterococcus spp	20 (12 - 31)	1 (1 - 2)	5 (2 - 8)	0 (0 - 1)
Mauritius	Escherichia coli	141 (85 - 214)	9 (6 - 14)	36 (21 - 55)	2 (1 - 4)
Mauritius	Group A Strep	7 (3 - 14)	1 (0 - 1)	1 (0 - 3)	0 (0 - 0)
Mauritius	Group B Strep	14 (8 - 22)	1 (1 - 2)	2 (0 - 5)	0 (0 - 0)
Mauritius	Haemophilus influenzae	3 (2 - 5)	0 (0 - 0)	1 (0 - 1)	0 (0 - 0)
Mauritius	Klebsiella pneumoniae	100 (61 - 155)	7 (4 - 10)	27 (14 - 48)	2 (1 - 3)
Mauritius	Morganella spp	0 (0 - 1)	0 (0 - 0)	0 (0 - 0)	0 (0 - 0)
Mauritius	Mycobacterium tuberculosis	1 (0 - 1)	0 (0 - 0)	0 (0 - 1)	0 (0 - 0)
Mauritius	Neisseria Gonorrheae				
Mauritius	Non Typhoidal Salmonellae	0 (0 - 0)	0 (0 - 0)	0 (0 - 0)	0 (0 - 0)
Mauritius	Proteus spp	18 (11 - 28)	1 (1 - 2)	3 (1 - 5)	0 (0 - 0)
Mauritius	Pseudomonas aeruginosa	58 (35 - 89)	4 (2 - 6)	14 (8 - 23)	1 (1 - 2)
Mauritius	Salmonella Paratyphi	0 (0 - 1)	0 (0 - 0)	0 (0 - 0)	0 (0 - 0)
Mauritius	Salmonella Typhi	1 (0 - 3)	0 (0 - 0)	0 (0 - 1)	0 (0 - 0)
Mauritius	Serratia spp	5 (2 - 8)	0 (0 - 1)	1 (1 - 2)	0 (0 - 0)
Mauritius	Shigella spp	0 (0 - 0)	0 (0 - 0)	0 (0 - 0)	0 (0 - 0)
Mauritius	Staphylococcus Aureus	166 (107 - 245)	11 (7 - 16)	51 (27 - 86)	3 (2 - 6)
Mauritius	Streptococcus pneumoniae	70 (49 - 98)	5 (3 - 7)	16 (9 - 23)	1 (1 - 2)
Mozambique	Acinetobacter baumannii	1,700 (974 - 2,600)	15 (8 - 24)	478 (261 - 774)	4 (2 - 7)
Mozambique	All	31,000 (24,000 - 41,000)	178 (134 - 236)	7,800 (5,400 - 11,000)	47 (32 - 70)
Mozambique	Citrobacter spp	153 (94 - 238)	1 (1 - 1)	45 (24 - 77)	0 (0 - 0)
Mozambique	Enterobacter spp	805 (565 - 1,200)	5 (4 - 8)	193 (126 - 283)	1 (1 - 2)
Mozambique	Enterococcus faecalis	591 (368 - 882)	4 (2 - 6)	157 (78 - 263)	1 (0 - 2)
Mozambique	Enterococcus faecium	482 (301 - 779)	3 (2 - 6)	122 (58 - 214)	1 (0 - 2)
Mozambique	Enterococcus spp	183 (113 - 298)	1 (1 - 2)	38 (8 - 78)	0 (0 - 1)
Mozambique	Escherichia coli	3,500 (2,600 - 4,700)	22 (16 - 29)	795 (561 - 1,100)	5 (3 - 7)
Mozambique	Group A Strep	273 (159 - 468)	2 (1 - 4)	26 (0 - 88)	0 (0 - 1)
Mozambique	Group B Strep	1,100 (834 - 1,600)	5 (4 - 7)	177 (34 - 346)	1 (0 - 2)
Mozambique	Haemophilus influenzae	536 (387 - 722)	2 (2 - 3)	108 (33 - 197)	0 (0 - 1)
Mozambique	Klebsiella pneumoniae	5,600 (4,300 - 7,300)	30 (22 - 40)	1,500 (915 - 2,300)	8 (5 - 12)
Mozambique	Morganella spp	7 (3 - 11)	0 (0 - 0)	2 (1 - 3)	0 (0 - 0)
Mozambique	Mycobacterium tuberculosis	2,700 (997 - 5,900)	20 (7 - 43)	1,400 (195 - 4,000)	11 (1 - 30)
Mozambique	Neisseria Gonorrheae				
Mozambique	Non Typhoidal Salmonellae	15 (5 - 34)	0 (0 - 0)	3 (0 - 9)	0 (0 - 0)
Mozambique	Proteus spp	317 (200 - 484)	3 (2 - 4)	42 (22 - 71)	0 (0 - 1)
Mozambique	Pseudomonas aeruginosa	1,300 (950 - 1,800)	8 (6 - 11)	355 (210 - 576)	2 (1 - 4)
Mozambique	Salmonella Paratyphi	0 (0 - 0)	0 (0 - 0)	0 (0 - 0)	0 (0 - 0)
Mozambique	Salmonella Typhi	2,200 (1,300 - 3,400)	6 (4 - 9)	329 (3 - 706)	1 (0 - 2)
Mozambique	Serratia spp	447 (291 - 679)	2 (1 - 3)	109 (60 - 185)	1 (0 - 1)
Mozambique	Shigella spp	35 (8 - 98)	0 (0 - 0)	7 (1 - 23)	0 (0 - 0)
Mozambique	Staphylococcus Aureus	4,100 (3,200 - 5,400)	27 (21 - 36)	892 (568 - 1,300)	6 (4 - 9)
Mozambique	Streptococcus pneumoniae	4,900 (3,700 - 6,300)	22 (18 - 28)	989 (595 - 1,500)	5 (3 - 7)
Namibia	Acinetobacter baumannii	132 (80 - 208)	10 (6 - 16)	41 (23 - 67)	3 (2 - 5)
Namibia	All	1,900 (1,400 - 2,600)	125 (92 - 169)	451 (306 - 644)	30 (21 - 42)
Namibia	Citrobacter spp	6 (4 - 10)	0 (0 - 1)	2 (1 - 3)	0 (0 - 0)
Namibia	Enterobacter spp	53 (35 - 76)	4 (2 - 5)	10 (6 - 15)	1 (0 - 1)
Namibia	Enterococcus faecalis	22 (12 - 36)	2 (1 - 2)	6 (3 - 11)	0 (0 - 1)
Namibia	Enterococcus faecium	55 (33 - 86)	4 (2 - 6)	13 (6 - 24)	1 (0 - 2)
Namibia	Enterococcus spp	20 (12 - 32)	1 (1 - 2)	4 (1 - 8)	0 (0 - 1)
Namibia	Escherichia coli	266 (186 - 373)	19 (13 - 26)	60 (39 - 87)	4 (3 - 6)
Namibia	Group A Strep	3 (1 - 5)	0 (0 - 0)	0 (0 - 1)	0 (0 - 0)
Namibia	Group B Strep	47 (33 - 67)	3 (2 - 4)	6 (0 - 14)	0 (0 - 1)
Namibia	Haemophilus influenzae	12 (8 - 18)	1 (1 - 1)	3 (1 - 5)	0 (0 - 0)
Namibia	Klebsiella pneumoniae	319 (226 - 439)	21 (15 - 29)	77 (45 - 123)	5 (3 - 8)

Namibia	Morganella spp	0 (0 - 1)	0 (0 - 0)	0 (0 - 0)	0 (0 - 0)
Namibia	Mycobacterium tuberculosis	177 (100 - 301)	11 (6 - 18)	74 (15 - 178)	5 (1 - 11)
Namibia	Neisseria Gonorrheae				
Namibia	Non Typhoidal Salmonellae	0 (0 - 0)	0 (0 - 0)	0 (0 - 0)	0 (0 - 0)
Namibia	Proteus spp	25 (15 - 39)	2 (1 - 3)	3 (2 - 5)	0 (0 - 0)
Namibia	Pseudomonas aeruginosa	128 (91 - 177)	9 (6 - 12)	31 (20 - 46)	2 (1 - 3)
Namibia	Salmonella Paratyphi	0 (0 - 0)	0 (0 - 0)	0 (0 - 0)	0 (0 - 0)
Namibia	Salmonella Typhi	16 (6 - 33)	1 (0 - 2)	3 (0 - 7)	0 (0 - 0)
Namibia	Serratia spp	15 (9 - 24)	1 (1 - 2)	3 (1 - 5)	0 (0 - 0)
Namibia	Shigella spp	6 (1 - 16)	0 (0 - 1)	1 (0 - 4)	0 (0 - 0)
Namibia	Staphylococcus Aureus	306 (226 - 411)	22 (16 - 28)	58 (36 - 87)	4 (3 - 6)
Namibia	Streptococcus pneumoniae	269 (191 - 358)	16 (12 - 21)	56 (34 - 84)	3 (2 - 5)
Niger	Acinetobacter baumannii	971 (634 - 1,400)	10 (6 - 15)	277 (169 - 437)	3 (2 - 4)
Niger	All	31,000 (24,000 - 41,000)	161 (122 - 211)	7,100 (5,000 - 9,500)	38 (28 - 51)
Niger	Citrobacter spp	182 (103 - 296)	1 (1 - 2)	56 (30 - 95)	0 (0 - 1)
Niger	Enterobacter spp	968 (681 - 1,300)	6 (4 - 8)	240 (158 - 340)	2 (1 - 2)
Niger	Enterococcus faecalis	370 (218 - 583)	3 (2 - 5)	99 (48 - 170)	1 (0 - 1)
Niger	Enterococcus faecium	391 (221 - 661)	4 (2 - 6)	100 (47 - 185)	1 (0 - 2)
Niger	Enterococcus spp	186 (113 - 296)	1 (1 - 2)	38 (9 - 75)	0 (0 - 1)
Niger	Escherichia coli	4,500 (3,300 - 6,100)	25 (18 - 33)	1,100 (740 - 1,500)	6 (4 - 8)
Niger	Group A Strep	95 (54 - 156)	1 (0 - 1)	9 (0 - 31)	0 (0 - 0)
Niger	Group B Strep	2,200 (1,600 - 3,000)	8 (6 - 11)	388 (110 - 738)	1 (0 - 3)
Niger	Haemophilus influenzae	590 (411 - 833)	2 (1 - 3)	138 (62 - 229)	1 (0 - 1)
Niger	Klebsiella pneumoniae	6,100 (4,500 - 8,100)	30 (22 - 40)	1,600 (977 - 2,400)	8 (5 - 12)
Niger	Morganella spp	3 (2 - 6)	0 (0 - 0)	1 (0 - 1)	0 (0 - 0)
Niger	Mycobacterium tuberculosis	614 (286 - 1,400)	6 (3 - 14)	253 (53 - 820)	3 (1 - 8)
Niger	Neisseria Gonorrheae				
Niger	Non Typhoidal Salmonellae	1 (0 - 2)	0 (0 - 0)	0 (0 - 1)	0 (0 - 0)
Niger	Proteus spp	216 (130 - 333)	2 (1 - 4)	32 (17 - 55)	0 (0 - 1)
Niger	Pseudomonas aeruginosa	1,400 (1,000 - 1,900)	8 (6 - 11)	337 (223 - 495)	2 (1 - 3)
Niger	Salmonella Paratyphi	1 (0 - 3)	0 (0 - 0)	0 (0 - 1)	0 (0 - 0)
Niger	Salmonella Typhi	898 (321 - 1,800)	3 (1 - 5)	139 (2 - 360)	0 (0 - 1)
Niger	Serratia spp	475 (295 - 727)	3 (2 - 4)	126 (68 - 208)	1 (0 - 1)
Niger	Shigella spp	20 (5 - 56)	0 (0 - 0)	4 (0 - 13)	0 (0 - 0)
Niger	Staphylococcus Aureus	3,300 (2,500 - 4,400)	20 (15 - 27)	749 (456 - 1,100)	5 (3 - 7)
Niger	Streptococcus pneumoniae	7,800 (5,600 - 10,000)	30 (23 - 38)	1,500 (810 - 2,200)	6 (3 - 8)
Nigeria	Acinetobacter baumannii	7,200 (4,700 - 10,000)	7 (4 - 10)	2,100 (1,300 - 3,200)	2 (1 - 3)
Nigeria	All	263,000 (203,000 -	155 (118 - 200)	64,000 (48,000 - 84,000)	39 (29 - 52)
Nigeria	Citrobacter spp	1,600 (964 - 2,400)	1 (1 - 2)	479 (259 - 789)	0 (0 - 1)
Nigeria	Enterobacter spp	9,300 (6,500 - 13,000)	6 (4 - 9)	2,200 (1,500 - 3,200)	1 (1 - 2)
Nigeria	Enterococcus faecalis	3,800 (2,300 - 5,800)	3 (2 - 5)	1,000 (485 - 1,700)	1 (0 - 2)
Nigeria	Enterococcus faecium	4,000 (2,300 - 6,500)	4 (2 - 6)	1,000 (463 - 1,900)	1 (0 - 2)
Nigeria	Enterococcus spp	2,100 (1,300 - 3,100)	2 (1 - 3)	419 (83 - 817)	0 (0 - 1)
Nigeria	Escherichia coli	38,000 (29,000 - 51,000)	24 (18 - 32)	10,000 (7,100 - 15,000)	7 (4 - 9)
Nigeria	Group A Strep	747 (425 - 1,300)	1 (0 - 1)	71 (0 - 241)	0 (0 - 0)
Nigeria	Group B Strep	17,000 (12,000 - 22,000)	7 (5 - 9)	2,900 (800 - 5,500)	1 (0 - 2)
Nigeria	Haemophilus influenzae	2,600 (1,900 - 3,700)	1 (1 - 1)	629 (271 - 1,000)	0 (0 - 0)
Nigeria	Klebsiella pneumoniae	44,000 (33,000 - 57,000)	26 (19 - 34)	13,000 (8,500 - 18,000)	8 (5 - 11)
Nigeria	Morganella spp	31 (17 - 51)	0 (0 - 0)	7 (3 - 13)	0 (0 - 0)
Nigeria	Mycobacterium tuberculosis	6,600 (3,200 - 14,000)	7 (3 - 14)	2,800 (483 - 8,500)	3 (1 - 9)
Nigeria	Neisseria Gonorrheae				
Nigeria	Non Typhoidal Salmonellae	395 (132 - 889)	0 (0 - 0)	82 (11 - 237)	0 (0 - 0)
Nigeria	Proteus spp	2,000 (1,200 - 3,100)	2 (1 - 4)	280 (135 - 470)	0 (0 - 1)
Nigeria	Pseudomonas aeruginosa	16,000 (12,000 - 20,000)	10 (7 - 14)	3,800 (2,500 - 5,600)	2 (2 - 4)
Nigeria	Salmonella Paratyphi	62 (15 - 167)	0 (0 - 0)	12 (2 - 37)	0 (0 - 0)
Nigeria	Salmonella Typhi	10,000 (6,500 - 15,000)	4 (3 - 6)	1,600 (265 - 3,200)	1 (0 - 1)
Nigeria	Serratia spp	3,400 (2,200 - 5,100)	2 (1 - 3)	823 (443 - 1,400)	1 (0 - 1)

Nigeria	Shigella spp	8,200 (3,300 - 16,000)	4 (2 - 7)	1,700 (209 - 4,100)	1 (0 - 2)
Nigeria	Staphylococcus Aureus	32,000 (25,000 - 41,000)	21 (16 - 28)	8,100 (4,900 - 12,000)	5 (3 - 8)
Nigeria	Streptococcus pneumoniae	54,000 (42,000 - 69,000)	24 (20 - 30)	11,000 (7,200 - 16,000)	5 (3 - 7)
Rwanda	Acinetobacter baumannii	563 (341 - 871)	11 (6 - 17)	174 (101 - 276)	3 (2 - 5)
Rwanda	All	9,800 (7,400 - 13,000)	141 (108 - 183)	2,400 (1,700 - 3,200)	35 (26 - 47)
Rwanda	Citrobacter spp	49 (30 - 78)	1 (0 - 1)	14 (8 - 24)	0 (0 - 0)
Rwanda	Enterobacter spp	308 (213 - 445)	5 (3 - 7)	73 (46 - 112)	1 (1 - 2)
Rwanda	Enterococcus faecalis	201 (119 - 308)	3 (2 - 5)	53 (25 - 91)	1 (0 - 1)
Rwanda	Enterococcus faecium	226 (136 - 366)	4 (2 - 6)	58 (27 - 102)	1 (0 - 2)
Rwanda	Enterococcus spp	78 (47 - 125)	1 (1 - 2)	16 (4 - 33)	0 (0 - 1)
Rwanda	Escherichia coli	1,400 (1,100 - 1,900)	21 (16 - 29)	344 (233 - 494)	5 (4 - 8)
Rwanda	Group A Strep	36 (19 - 65)	1 (0 - 1)	3 (0 - 12)	0 (0 - 0)
Rwanda	Group B Strep	313 (232 - 427)	4 (3 - 5)	49 (9 - 97)	1 (0 - 1)
Rwanda	Haemophilus influenzae	80 (56 - 112)	1 (1 - 1)	16 (5 - 30)	0 (0 - 0)
Rwanda	Klebsiella pneumoniae	1,800 (1,300 - 2,300)	25 (19 - 33)	495 (303 - 743)	7 (4 - 11)
Rwanda	Morganella spp	2 (1 - 4)	0 (0 - 0)	1 (0 - 1)	0 (0 - 0)
Rwanda	Mycobacterium tuberculosis	328 (133 - 752)	5 (2 - 11)	172 (21 - 516)	3 (0 - 7)
Rwanda	Neisseria Gonorrheae				
Rwanda	Non Typhoidal Salmonellae	1 (0 - 3)	0 (0 - 0)	0 (0 - 1)	0 (0 - 0)
Rwanda	Proteus spp	130 (79 - 200)	3 (2 - 4)	17 (9 - 27)	0 (0 - 1)
Rwanda	Pseudomonas aeruginosa	666 (485 - 906)	10 (7 - 14)	164 (97 - 260)	3 (1 - 4)
Rwanda	Salmonella Paratyphi	0 (0 - 0)	0 (0 - 0)	0 (0 - 0)	0 (0 - 0)
Rwanda	Salmonella Typhi	456 (272 - 722)	4 (2 - 5)	75 (11 - 155)	1 (0 - 1)
Rwanda	Serratia spp	100 (62 - 160)	1 (1 - 2)	24 (12 - 40)	0 (0 - 1)
Rwanda	Shigella spp	15 (3 - 41)	0 (0 - 1)	3 (0 - 11)	0 (0 - 0)
Rwanda	Staphylococcus Aureus	1,600 (1,300 - 2,100)	26 (20 - 33)	344 (219 - 510)	6 (4 - 8)
Rwanda	Streptococcus pneumoniae	1,400 (1,100 - 1,800)	17 (14 - 21)	273 (164 - 408)	3 (2 - 5)
Sao Tome and	Acinetobacter baumannii	10 (6 - 16)	11 (7 - 18)	3 (2 - 5)	4 (2 - 6)
Sao Tome and	All	142 (103 - 189)	137 (102 - 178)	34 (24 - 46)	33 (24 - 44)
Sao Tome and	Citrobacter spp	1 (0 - 1)	1 (0 - 1)	0 (0 - 0)	0 (0 - 0)
Sao Tome and	Enterobacter spp	5 (3 - 7)	4 (3 - 6)	1 (1 - 2)	1 (1 - 1)
Sao Tome and	Enterococcus faecalis	2 (1 - 4)	2 (1 - 4)	1 (0 - 1)	1 (0 - 1)
Sao Tome and	Enterococcus faecium	4 (2 - 7)	4 (2 - 6)	1 (0 - 2)	1 (1 - 2)
Sao Tome and	Enterococcus spp	2 (1 - 2)	2 (1 - 2)	0 (0 - 1)	0 (0 - 1)
Sao Tome and	Escherichia coli	20 (14 - 29)	21 (15 - 28)	5 (3 - 7)	5 (3 - 7)
Sao Tome and	Group A Strep	0 (0 - 1)	1 (0 - 1)	0 (0 - 0)	0 (0 - 0)
Sao Tome and	Group B Strep	4 (3 - 5)	3 (2 - 4)	1 (0 - 1)	1 (0 - 1)
Sao Tome and	Haemophilus influenzae	1 (0 - 1)	1 (0 - 1)	0 (0 - 0)	0 (0 - 0)
Sao Tome and	Klebsiella pneumoniae	25 (18 - 33)	24 (17 - 31)	6 (4 - 10)	6 (4 - 10)
Sao Tome and	Morganella spp	0 (0 - 0)	0 (0 - 0)	0 (0 - 0)	0 (0 - 0)
Sao Tome and	Mycobacterium tuberculosis	2 (0 - 6)	2 (1 - 5)	1 (0 - 4)	1 (0 - 3)
Sao Tome and	Neisseria Gonorrheae				
Sao Tome and	Non Typhoidal Salmonellae	0 (0 - 0)	0 (0 - 0)	0 (0 - 0)	0 (0 - 0)
Sao Tome and	Proteus spp	2 (1 - 3)	2 (2 - 4)	0 (0 - 1)	0 (0 - 1)
Sao Tome and	Pseudomonas aeruginosa	11 (8 - 15)	11 (8 - 15)	3 (2 - 4)	3 (2 - 4)
Sao Tome and	Salmonella Paratyphi	0 (0 - 0)	0 (0 - 0)	0 (0 - 0)	0 (0 - 0)
Sao Tome and	Salmonella Typhi	4 (2 - 7)	2 (1 - 3)	1 (0 - 1)	0 (0 - 1)
Sao Tome and	Serratia spp	2 (1 - 3)	1 (1 - 2)	0 (0 - 1)	0 (0 - 1)
Sao Tome and	Shigella spp	0 (0 - 0)	0 (0 - 0)	0 (0 - 0)	0 (0 - 0)
Sao Tome and	Staphylococcus Aureus	28 (22 - 36)	29 (23 - 36)	6 (4 - 10)	7 (4 - 10)
Sao Tome and	Streptococcus pneumoniae	19 (15 - 24)	17 (13 - 21)	4 (2 - 5)	3 (2 - 5)
Senegal	Acinetobacter baumannii	779 (459 - 1,200)	11 (6 - 18)	221 (124 - 354)	3 (2 - 5)
Senegal	All	12,000 (8,800 - 16,000)	127 (94 - 168)	2,700 (1,900 - 3,600)	29 (21 - 40)
Senegal	Citrobacter spp	67 (39 - 104)	1 (0 - 1)	20 (10 - 35)	0 (0 - 0)
Senegal	Enterobacter spp	390 (260 - 564)	4 (3 - 6)	94 (59 - 142)	1 (1 - 2)
Senegal	Enterococcus faecalis	249 (148 - 399)	3 (2 - 5)	66 (31 - 116)	1 (0 - 1)
Senegal	Enterococcus faecium	276 (160 - 454)	3 (2 - 6)	69 (31 - 126)	1 (0 - 2)

Senegal	Enterococcus spp	119 (73 - 188)	1 (1 - 2)	24 (5 - 50)	0 (0 - 1)
Senegal	Escherichia coli	1,900 (1,400 - 2,600)	20 (15 - 28)	459 (316 - 654)	5 (3 - 7)
Senegal	Group A Strep	45 (24 - 80)	1 (0 - 1)	4 (0 - 15)	0 (0 - 0)
Senegal	Group B Strep	483 (352 - 662)	4 (3 - 5)	85 (24 - 160)	1 (0 - 1)
Senegal	Haemophilus influenzae	87 (59 - 121)	1 (1 - 1)	21 (9 - 35)	0 (0 - 0)
Senegal	Klebsiella pneumoniae	2,300 (1,700 - 3,100)	24 (17 - 32)	540 (362 - 779)	5 (4 - 8)
Senegal	Morganella spp	4 (2 - 6)	0 (0 - 0)	1 (0 - 2)	0 (0 - 0)
Senegal	Mycobacterium tuberculosis	329 (148 - 630)	4 (2 - 8)	141 (24 - 397)	2 (0 - 5)
Senegal	Neisseria Gonorrheae				
Senegal	Non Typhoidal Salmonellae	1 (0 - 2)	0 (0 - 0)	0 (0 - 1)	0 (0 - 0)
Senegal	Proteus spp	152 (93 - 236)	2 (1 - 3)	22 (11 - 37)	0 (0 - 1)
Senegal	Pseudomonas aeruginosa	732 (529 - 992)	8 (6 - 11)	174 (111 - 257)	2 (1 - 3)
Senegal	Salmonella Paratyphi	0 (0 - 1)	0 (0 - 0)	0 (0 - 0)	0 (0 - 0)
Senegal	Salmonella Typhi	65 (13 - 237)	0 (0 - 1)	13 (1 - 53)	0 (0 - 0)
Senegal	Serratia spp	212 (130 - 331)	2 (1 - 3)	57 (31 - 93)	1 (0 - 1)
Senegal	Shigella spp	19 (5 - 46)	0 (0 - 0)	4 (0 - 12)	0 (0 - 0)
Senegal	Staphylococcus Aureus	1,900 (1,400 - 2,500)	22 (17 - 28)	372 (244 - 538)	4 (3 - 6)
Senegal	Streptococcus pneumoniae	1,700 (1,300 - 2,200)	16 (12 - 20)	311 (177 - 475)	3 (2 - 4)
Seychelles	Acinetobacter baumannii	8 (5 - 12)	8 (5 - 13)	3 (1 - 4)	3 (2 - 4)
Seychelles	All	75 (56 - 99)	77 (58 - 101)	19 (14 - 25)	19 (14 - 26)
Seychelles	Citrobacter spp	1 (0 - 1)	1 (0 - 1)	0 (0 - 0)	0 (0 - 0)
Seychelles	Enterobacter spp	3 (2 - 4)	3 (2 - 4)	1 (0 - 1)	1 (0 - 1)
Seychelles	Enterococcus faecalis	2 (1 - 3)	2 (1 - 3)	1 (0 - 1)	1 (0 - 1)
Seychelles	Enterococcus faecium	3 (2 - 5)	3 (2 - 5)	1 (0 - 1)	1 (0 - 1)
Seychelles	Enterococcus spp	2 (1 - 2)	2 (1 - 3)	0 (0 - 1)	0 (0 - 1)
Seychelles	Escherichia coli	14 (10 - 18)	14 (10 - 18)	3 (2 - 4)	3 (2 - 5)
Seychelles	Group A Strep	1 (0 - 1)	1 (0 - 1)	0 (0 - 0)	0 (0 - 0)
Seychelles	Group B Strep	1 (1 - 1)	1 (1 - 1)	0 (0 - 0)	0 (0 - 0)
Seychelles	Haemophilus influenzae	1 (1 - 1)	1 (1 - 1)	0 (0 - 0)	0 (0 - 0)
Seychelles	Klebsiella pneumoniae	9 (6 - 12)	9 (7 - 12)	2 (1 - 4)	3 (1 - 4)
Seychelles	Morganella spp	0 (0 - 0)	0 (0 - 0)	0 (0 - 0)	0 (0 - 0)
Seychelles	Mycobacterium tuberculosis	0 (0 - 0)	0 (0 - 0)	0 (0 - 0)	0 (0 - 0)
Seychelles	Neisseria Gonorrheae				
Seychelles	Non Typhoidal Salmonellae	0 (0 - 0)	0 (0 - 0)	0 (0 - 0)	0 (0 - 0)
Seychelles	Proteus spp	1 (1 - 2)	2 (1 - 2)	0 (0 - 0)	0 (0 - 0)
Seychelles	Pseudomonas aeruginosa	5 (3 - 6)	5 (4 - 7)	1 (1 - 2)	1 (1 - 2)
Seychelles	Salmonella Paratyphi	0 (0 - 0)	0 (0 - 0)	0 (0 - 0)	0 (0 - 0)
Seychelles	Salmonella Typhi	0 (0 - 1)	0 (0 - 1)	0 (0 - 0)	0 (0 - 0)
Seychelles	Serratia spp	1 (0 - 1)	1 (0 - 1)	0 (0 - 0)	0 (0 - 0)
Seychelles	Shigella spp	0 (0 - 0)	0 (0 - 0)	0 (0 - 0)	0 (0 - 0)
Seychelles	Staphylococcus Aureus	12 (10 - 16)	13 (10 - 16)	4 (2 - 6)	4 (2 - 6)
Seychelles	Streptococcus pneumoniae	12 (10 - 14)	13 (10 - 15)	2 (2 - 3)	3 (2 - 4)
Sierra Leone	Acinetobacter baumannii	446 (266 - 698)	11 (6 - 17)	128 (73 - 198)	3 (2 - 5)
Sierra Leone	All	9,700 (7,100 - 13,000)	162 (121 - 218)	2,200 (1,600 - 3,100)	38 (28 - 52)
Sierra Leone	Citrobacter spp	51 (28 - 83)	1 (1 - 1)	16 (8 - 28)	0 (0 - 1)
Sierra Leone	Enterobacter spp	274 (187 - 404)	5 (4 - 7)	67 (44 - 100)	1 (1 - 2)
Sierra Leone	Enterococcus faecalis	126 (74 - 204)	2 (1 - 4)	34 (16 - 60)	1 (0 - 1)
Sierra Leone	Enterococcus faecium	156 (91 - 273)	3 (2 - 6)	41 (20 - 74)	1 (0 - 2)
Sierra Leone	Enterococcus spp	72 (44 - 118)	1 (1 - 2)	15 (3 - 31)	0 (0 - 1)
Sierra Leone	Escherichia coli	1,300 (949 - 1,800)	24 (18 - 32)	314 (215 - 446)	6 (4 - 8)
Sierra Leone	Group A Strep	35 (19 - 59)	1 (0 - 1)	3 (0 - 11)	0 (0 - 0)
Sierra Leone	Group B Strep	405 (289 - 561)	6 (4 - 7)	66 (17 - 129)	1 (0 - 2)
Sierra Leone	Haemophilus influenzae	114 (77 - 162)	1 (1 - 2)	28 (12 - 47)	0 (0 - 1)
Sierra Leone	Klebsiella pneumoniae	1,900 (1,400 - 2,600)	30 (22 - 41)	469 (297 - 713)	8 (5 - 11)
Sierra Leone	Morganella spp	2 (1 - 3)	0 (0 - 0)	0 (0 - 1)	0 (0 - 0)
Sierra Leone	Mycobacterium tuberculosis	203 (73 - 522)	5 (2 - 12)	88 (8 - 323)	2 (0 - 7)
Sierra Leone	Neisseria Gonorrheae				

Sierra Leone	Non Typhoidal Salmonellae	0 (0 - 0)	0 (0 - 0)	0 (0 - 0)	0 (0 - 0)
Sierra Leone	Proteus spp	87 (51 - 138)	2 (1 - 4)	13 (6 - 22)	0 (0 - 1)
Sierra Leone	Pseudomonas aeruginosa	558 (398 - 762)	10 (7 - 14)	138 (86 - 212)	2 (2 - 4)
Sierra Leone	Salmonella Paratyphi	0 (0 - 0)	0 (0 - 0)	0 (0 - 0)	0 (0 - 0)
Sierra Leone	Salmonella Typhi	528 (292 - 834)	5 (3 - 8)	74 (0 - 178)	1 (0 - 2)
Sierra Leone	Serratia spp	145 (88 - 237)	2 (1 - 4)	39 (19 - 67)	1 (0 - 1)
Sierra Leone	Shigella spp	3 (1 - 9)	0 (0 - 0)	1 (0 - 2)	0 (0 - 0)
Sierra Leone	Staphylococcus Aureus	1,300 (982 - 1,800)	25 (19 - 32)	304 (185 - 454)	6 (4 - 9)
Sierra Leone	Streptococcus pneumoniae	2,000 (1,400 - 2,700)	27 (20 - 35)	373 (205 - 597)	5 (3 - 8)
South Africa	Acinetobacter baumannii	3,200 (2,000 - 4,800)	8 (5 - 12)	1,000 (592 - 1,600)	3 (2 - 4)
South Africa	All	39,000 (30,000 - 50,000)	91 (70 - 118)	9,500 (7,100 - 13,000)	22 (17 - 30)
South Africa	Citrobacter spp	108 (69 - 162)	0 (0 - 0)	32 (18 - 52)	0 (0 - 0)
South Africa	Enterobacter spp	1,300 (908 - 1,900)	3 (2 - 4)	213 (112 - 342)	1 (0 - 1)
South Africa	Enterococcus faecalis	553 (333 - 834)	1 (1 - 2)	149 (72 - 259)	0 (0 - 1)
South Africa	Enterococcus faecium	1,300 (782 - 1,900)	3 (2 - 4)	282 (107 - 515)	1 (0 - 1)
South Africa	Enterococcus spp	620 (391 - 947)	2 (1 - 2)	130 (38 - 255)	0 (0 - 1)
South Africa	Escherichia coli	5,400 (3,900 - 7,200)	13 (10 - 17)	1,200 (821 - 1,600)	3 (2 - 4)
South Africa	Group A Strep	53 (26 - 96)	0 (0 - 0)	5 (0 - 18)	0 (0 - 0)
South Africa	Group B Strep	1,200 (893 - 1,600)	3 (2 - 4)	146 (0 - 351)	0 (0 - 1)
South Africa	Haemophilus influenzae	172 (131 - 229)	0 (0 - 1)	37 (14 - 63)	0 (0 - 0)
South Africa	Klebsiella pneumoniae	6,700 (5,000 - 8,700)	16 (12 - 21)	1,900 (1,200 - 2,800)	5 (3 - 7)
South Africa	Morganella spp	9 (4 - 15)	0 (0 - 0)	2 (1 - 4)	0 (0 - 0)
South Africa	Mycobacterium tuberculosis	3,000 (2,000 - 4,800)	6 (4 - 10)	1,000 (247 - 2,600)	2 (1 - 5)
South Africa	Neisseria Gonorrheae				
South Africa	Non Typhoidal Salmonellae	10 (2 - 30)	0 (0 - 0)	2 (0 - 8)	0 (0 - 0)
South Africa	Proteus spp	599 (382 - 884)	2 (1 - 2)	69 (33 - 117)	0 (0 - 0)
South Africa	Pseudomonas aeruginosa	2,400 (1,700 - 3,200)	6 (4 - 8)	568 (393 - 801)	1 (1 - 2)
South Africa	Salmonella Paratyphi	0 (0 - 1)	0 (0 - 0)	0 (0 - 0)	0 (0 - 0)
South Africa	Salmonella Typhi	267 (100 - 533)	1 (0 - 1)	43 (2 - 110)	0 (0 - 0)
South Africa	Serratia spp	295 (178 - 446)	1 (0 - 1)	44 (19 - 76)	0 (0 - 0)
South Africa	Shigella spp	18 (5 - 45)	0 (0 - 0)	4 (0 - 12)	0 (0 - 0)
South Africa	Staphylococcus Aureus	6,000 (4,700 - 7,600)	15 (11 - 18)	1,400 (839 - 2,100)	3 (2 - 5)
South Africa	Streptococcus pneumoniae	5,900 (5,000 - 6,900)	13 (11 - 16)	1,300 (887 - 1,700)	3 (2 - 4)
South Sudan	Acinetobacter baumannii	582 (379 - 847)	12 (8 - 19)	168 (102 - 263)	4 (2 - 6)
South Sudan	All	12,000 (8,800 - 15,000)	151 (115 - 199)	2,700 (2,000 - 3,600)	37 (26 - 52)
South Sudan	Citrobacter spp	128 (72 - 202)	2 (1 - 3)	38 (20 - 64)	1 (0 - 1)
South Sudan	Enterobacter spp	495 (346 - 710)	7 (5 - 10)	121 (80 - 179)	2 (1 - 3)
South Sudan	Enterococcus faecalis	161 (97 - 251)	3 (2 - 5)	42 (20 - 73)	1 (0 - 2)
South Sudan	Enterococcus faecium	136 (76 - 226)	3 (2 - 5)	34 (15 - 67)	1 (0 - 2)
South Sudan	Enterococcus spp	74 (44 - 117)	1 (1 - 2)	15 (4 - 31)	0 (0 - 1)
South Sudan	Escherichia coli	1,400 (1,100 - 1,900)	21 (15 - 27)	338 (239 - 468)	5 (3 - 7)
South Sudan	Group A Strep	56 (31 - 97)	1 (1 - 2)	5 (0 - 19)	0 (0 - 0)
South Sudan	Group B Strep	779 (563 - 1,100)	7 (5 - 10)	132 (35 - 251)	1 (0 - 2)
South Sudan	Haemophilus influenzae	335 (239 - 454)	3 (2 - 4)	71 (26 - 120)	1 (0 - 1)
South Sudan	Klebsiella pneumoniae	1,800 (1,300 - 2,300)	23 (17 - 30)	440 (284 - 670)	6 (4 - 9)
South Sudan	Morganella spp	2 (1 - 3)	0 (0 - 0)	0 (0 - 1)	0 (0 - 0)
South Sudan	Mycobacterium tuberculosis	456 (114 - 1,200)	10 (3 - 26)	238 (20 - 748)	5 (0 - 16)
South Sudan	Neisseria Gonorrheae				
South Sudan	Non Typhoidal Salmonellae	0 (0 - 1)	0 (0 - 0)	0 (0 - 0)	0 (0 - 0)
South Sudan	Proteus spp	99 (57 - 155)	3 (2 - 4)	14 (7 - 25)	0 (0 - 1)
South Sudan	Pseudomonas aeruginosa	401 (284 - 542)	6 (4 - 8)	102 (61 - 162)	2 (1 - 2)
South Sudan	Salmonella Paratyphi	0 (0 - 0)	0 (0 - 0)	0 (0 - 0)	0 (0 - 0)
South Sudan	Salmonella Typhi	186 (55 - 442)	2 (1 - 4)	32 (2 - 100)	0 (0 - 1)
South Sudan	Serratia spp	210 (132 - 331)	3 (2 - 5)	53 (28 - 91)	1 (0 - 1)
South Sudan	Shigella spp	136 (39 - 322)	2 (1 - 5)	28 (3 - 83)	0 (0 - 1)
South Sudan	Staphylococcus Aureus	953 (714 - 1,300)	14 (10 - 20)	176 (113 - 256)	3 (2 - 4)
South Sudan	Streptococcus pneumoniae	3,100 (2,300 - 4,200)	29 (23 - 38)	656 (383 - 997)	6 (4 - 9)

Eswatini	Acinetobacter baumannii	85 (50 - 135)	16 (9 - 26)	26 (14 - 45)	5 (3 - 9)
Eswatini	All	1,100 (787 - 1,500)	170 (119 - 236)	296 (182 - 467)	44 (28 - 68)
Eswatini	Citrobacter spp	4 (2 - 6)	1 (0 - 1)	1 (1 - 2)	0 (0 - 0)
Eswatini	Enterobacter spp	27 (18 - 40)	4 (3 - 7)	5 (3 - 8)	1 (1 - 1)
Eswatini	Enterococcus faecalis	14 (8 - 24)	2 (1 - 4)	4 (2 - 7)	1 (0 - 1)
Eswatini	Enterococcus faecium	26 (16 - 42)	4 (3 - 7)	6 (3 - 11)	1 (1 - 2)
Eswatini	Enterococcus spp	9 (6 - 15)	2 (1 - 3)	2 (0 - 4)	0 (0 - 1)
Eswatini	Escherichia coli	138 (96 - 194)	23 (16 - 33)	32 (21 - 47)	5 (3 - 8)
Eswatini	Group A Strep	2 (1 - 4)	0 (0 - 1)	0 (0 - 1)	0 (0 - 0)
Eswatini	Group B Strep	28 (20 - 40)	4 (3 - 6)	4 (0 - 8)	1 (0 - 1)
Eswatini	Haemophilus influenzae	9 (6 - 13)	1 (1 - 2)	2 (1 - 3)	0 (0 - 0)
Eswatini	Klebsiella pneumoniae	186 (133 - 255)	28 (20 - 40)	48 (28 - 75)	7 (4 - 12)
Eswatini	Morganella spp	0 (0 - 0)	0 (0 - 0)	0 (0 - 0)	0 (0 - 0)
Eswatini	Mycobacterium tuberculosis	171 (69 - 328)	22 (9 - 43)	82 (14 - 213)	11 (2 - 28)
Eswatini	Neisseria Gonorrheae				
Eswatini	Non Typhoidal Salmonellae	0 (0 - 0)	0 (0 - 0)	0 (0 - 0)	0 (0 - 0)
Eswatini	Proteus spp	13 (8 - 20)	3 (2 - 4)	2 (1 - 3)	0 (0 - 1)
Eswatini	Pseudomonas aeruginosa	59 (41 - 85)	10 (7 - 14)	15 (10 - 23)	2 (2 - 4)
Eswatini	Salmonella Paratyphi	0 (0 - 0)	0 (0 - 0)	0 (0 - 0)	0 (0 - 0)
Eswatini	Salmonella Typhi	15 (5 - 29)	2 (1 - 3)	2 (0 - 6)	0 (0 - 1)
Eswatini	Serratia spp	9 (5 - 13)	1 (1 - 2)	2 (1 - 3)	0 (0 - 0)
Eswatini	Shigella spp	4 (1 - 10)	1 (0 - 1)	1 (0 - 3)	0 (0 - 0)
Eswatini	Staphylococcus Aureus	155 (113 - 209)	25 (18 - 34)	27 (17 - 41)	4 (3 - 7)
Eswatini	Streptococcus pneumoniae	169 (124 - 227)	21 (15 - 28)	34 (21 - 50)	4 (3 - 6)
Tanzania	Acinetobacter baumannii	2,600 (1,600 - 4,000)	10 (6 - 16)	816 (481 - 1,300)	3 (2 - 5)
Tanzania	All	54,000 (42,000 - 70,000)	144 (112 - 184)	13,000 (9,400 - 17,000)	35 (25 - 47)
Tanzania	Citrobacter spp	284 (172 - 444)	1 (0 - 1)	84 (46 - 144)	0 (0 - 0)
Tanzania	Enterobacter spp	1,500 (1,100 - 2,200)	5 (3 - 6)	373 (248 - 541)	1 (1 - 2)
Tanzania	Enterococcus faecalis	1,100 (710 - 1,700)	3 (2 - 5)	301 (149 - 511)	1 (0 - 2)
Tanzania	Enterococcus faecium	1,000 (619 - 1,600)	3 (2 - 6)	255 (128 - 447)	1 (0 - 2)
Tanzania	Enterococcus spp	450 (272 - 698)	1 (1 - 2)	92 (20 - 189)	0 (0 - 1)
Tanzania	Escherichia coli	6,900 (5,100 - 9,000)	21 (15 - 27)	1,700 (1,200 - 2,300)	5 (4 - 7)
Tanzania	Group A Strep	270 (147 - 466)	1 (0 - 2)	26 (0 - 90)	0 (0 - 0)
Tanzania	Group B Strep	2,100 (1,500 - 2,800)	4 (3 - 6)	334 (77 - 650)	1 (0 - 1)
Tanzania	Haemophilus influenzae	618 (439 - 859)	1 (1 - 2)	132 (49 - 236)	0 (0 - 0)
Tanzania	Klebsiella pneumoniae	9,500 (7,100 - 13,000)	24 (18 - 32)	2,300 (1,500 - 3,500)	6 (4 - 9)
Tanzania	Morganella spp	10 (5 - 16)	0 (0 - 0)	2 (1 - 4)	0 (0 - 0)
Tanzania	Mycobacterium tuberculosis	1,800 (648 - 4,900)	6 (2 - 17)	853 (147 - 2,900)	3 (1 - 10)
Tanzania	Neisseria Gonorrheae				
Tanzania	Non Typhoidal Salmonellae	0 (0 - 0)	0 (0 - 0)	0 (0 - 0)	0 (0 - 0)
Tanzania	Proteus spp	598 (368 - 914)	2 (1 - 4)	81 (44 - 134)	0 (0 - 1)
Tanzania	Pseudomonas aeruginosa	2,900 (2,200 - 3,900)	8 (6 - 11)	730 (459 - 1,100)	2 (1 - 3)
Tanzania	Salmonella Paratyphi	0 (0 - 1)	0 (0 - 0)	0 (0 - 0)	0 (0 - 0)
Tanzania	Salmonella Typhi	3,600 (2,300 - 5,400)	5 (3 - 8)	613 (111 - 1,200)	1 (0 - 2)
Tanzania	Serratia spp	617 (388 - 949)	2 (1 - 2)	146 (79 - 241)	0 (0 - 1)
Tanzania	Shigella spp	11 (3 - 32)	0 (0 - 0)	2 (0 - 8)	0 (0 - 0)
Tanzania	Staphylococcus Aureus	9,100 (7,200 - 12,000)	27 (22 - 34)	1,900 (1,300 - 2,800)	6 (4 - 8)
Tanzania	Streptococcus pneumoniae	9,000 (7,000 - 12,000)	19 (16 - 23)	1,800 (1,100 - 2,600)	4 (2 - 5)
The Gambia	Acinetobacter baumannii	126 (78 - 191)	13 (8 - 20)	36 (21 - 56)	4 (2 - 6)
The Gambia	All	1,800 (1,400 - 2,400)	153 (116 - 198)	402 (295 - 536)	34 (25 - 46)
The Gambia	Citrobacter spp	11 (7 - 16)	1 (1 - 1)	3 (2 - 5)	0 (0 - 0)
The Gambia	Enterobacter spp	60 (42 - 84)	5 (4 - 7)	15 (10 - 21)	1 (1 - 2)
The Gambia	Enterococcus faecalis	32 (19 - 49)	3 (2 - 4)	9 (4 - 15)	1 (0 - 1)
The Gambia	Enterococcus faecium	44 (27 - 71)	4 (2 - 7)	11 (5 - 19)	1 (1 - 2)
The Gambia	Enterococcus spp	18 (12 - 28)	2 (1 - 3)	4 (1 - 8)	0 (0 - 1)
The Gambia	Escherichia coli	276 (201 - 367)	24 (18 - 32)	60 (42 - 83)	5 (4 - 7)
The Gambia	Group A Strep	7 (4 - 13)	1 (0 - 1)	1 (0 - 3)	0 (0 - 0)

The Gambia	Group B Strep	64 (45 - 87)	4 (3 - 6)	10 (2 - 20)	1 (0 - 1)
The Gambia	Haemophilus influenzae	11 (8 - 15)	1 (1 - 1)	3 (1 - 4)	0 (0 - 0)
The Gambia	Klebsiella pneumoniae	353 (267 - 463)	28 (21 - 37)	81 (54 - 120)	7 (4 - 10)
The Gambia	Morganella spp	0 (0 - 1)	0 (0 - 0)	0 (0 - 0)	0 (0 - 0)
The Gambia	Mycobacterium tuberculosis	51 (14 - 148)	5 (1 - 14)	25 (2 - 97)	2 (0 - 9)
The Gambia	Neisseria Gonorrheae				
The Gambia	Non Typhoidal Salmonellae	0 (0 - 0)	0 (0 - 0)	0 (0 - 0)	0 (0 - 0)
The Gambia	Proteus spp	24 (15 - 36)	2 (2 - 4)	3 (2 - 5)	0 (0 - 1)
The Gambia	Pseudomonas aeruginosa	108 (80 - 144)	9 (7 - 12)	26 (17 - 39)	2 (2 - 3)
The Gambia	Salmonella Paratyphi	0 (0 - 0)	0 (0 - 0)	0 (0 - 0)	0 (0 - 0)
The Gambia	Salmonella Typhi	55 (22 - 104)	2 (1 - 4)	8 (0 - 22)	0 (0 - 1)
The Gambia	Serratia spp	34 (22 - 52)	3 (2 - 4)	9 (5 - 15)	1 (0 - 1)
The Gambia	Shigella spp	1 (0 - 3)	0 (0 - 0)	0 (0 - 1)	0 (0 - 0)
The Gambia	Staphylococcus Aureus	219 (170 - 280)	20 (16 - 26)	41 (27 - 58)	4 (3 - 5)
The Gambia	Streptococcus pneumoniae	341 (264 - 426)	25 (20 - 31)	57 (28 - 91)	4 (2 - 7)
Togo	Acinetobacter baumannii	333 (198 - 514)	10 (6 - 16)	96 (55 - 151)	3 (2 - 5)
Togo	All	6,300 (4,700 - 8,300)	141 (107 - 186)	1,500 (1,100 - 2,000)	34 (25 - 46)
Togo	Citrobacter spp	32 (18 - 52)	1 (0 - 1)	10 (5 - 17)	0 (0 - 0)
Togo	Enterobacter spp	199 (137 - 293)	5 (3 - 7)	47 (31 - 71)	1 (1 - 2)
Togo	Enterococcus faecalis	113 (65 - 177)	3 (1 - 4)	30 (14 - 53)	1 (0 - 1)
Togo	Enterococcus faecium	131 (76 - 225)	3 (2 - 6)	32 (14 - 60)	1 (0 - 2)
Togo	Enterococcus spp	53 (32 - 84)	1 (1 - 2)	11 (2 - 22)	0 (0 - 1)
Togo	Escherichia coli	963 (713 - 1,300)	22 (16 - 29)	236 (164 - 334)	5 (4 - 8)
Togo	Group A Strep	21 (11 - 38)	1 (0 - 1)	2 (0 - 7)	0 (0 - 0)
Togo	Group B Strep	243 (171 - 339)	4 (3 - 6)	42 (11 - 81)	1 (0 - 1)
Togo	Haemophilus influenzae	56 (39 - 76)	1 (1 - 1)	13 (6 - 21)	0 (0 - 0)
Togo	Klebsiella pneumoniae	1,200 (848 - 1,600)	26 (19 - 34)	308 (187 - 475)	7 (4 - 10)
Togo	Morganella spp	1 (1 - 2)	0 (0 - 0)	0 (0 - 0)	0 (0 - 0)
Togo	Mycobacterium tuberculosis	173 (55 - 448)	4 (1 - 11)	79 (7 - 264)	2 (0 - 7)
Togo	Neisseria Gonorrheae				
Togo	Non Typhoidal Salmonellae	0 (0 - 1)	0 (0 - 0)	0 (0 - 0)	0 (0 - 0)
Togo	Proteus spp	64 (38 - 102)	2 (1 - 3)	8 (4 - 15)	0 (0 - 0)
Togo	Pseudomonas aeruginosa	353 (251 - 479)	8 (6 - 11)	86 (55 - 128)	2 (1 - 3)
Togo	Salmonella Paratyphi	0 (0 - 0)	0 (0 - 0)	0 (0 - 0)	0 (0 - 0)
Togo	Salmonella Typhi	259 (131 - 419)	3 (2 - 5)	40 (1 - 92)	1 (0 - 1)
Togo	Serratia spp	93 (57 - 149)	2 (1 - 3)	25 (13 - 42)	1 (0 - 1)
Togo	Shigella spp	10 (2 - 28)	0 (0 - 0)	2 (0 - 7)	0 (0 - 0)
Togo	Staphylococcus Aureus	1,000 (800 - 1,400)	27 (21 - 34)	236 (153 - 342)	6 (4 - 9)
Togo	Streptococcus pneumoniae	974 (728 - 1,300)	19 (15 - 24)	180 (102 - 283)	4 (2 - 5)
Uganda	Acinetobacter baumannii	1,400 (867 - 2,100)	9 (5 - 14)	355 (195 - 566)	2 (1 - 4)
Uganda	All	31,000 (23,000 - 40,000)	130 (98 - 167)	7,100 (5,100 - 9,700)	31 (22 - 43)
Uganda	Citrobacter spp	221 (134 - 345)	1 (1 - 1)	65 (36 - 109)	0 (0 - 0)
Uganda	Enterobacter spp	1,100 (806 - 1,600)	5 (4 - 7)	267 (180 - 392)	1 (1 - 2)
Uganda	Enterococcus faecalis	584 (360 - 860)	3 (1 - 4)	154 (79 - 258)	1 (0 - 1)
Uganda	Enterococcus faecium	608 (382 - 947)	3 (2 - 5)	154 (77 - 270)	1 (0 - 2)
Uganda	Enterococcus spp	276 (168 - 419)	1 (1 - 2)	57 (12 - 115)	0 (0 - 1)
Uganda	Escherichia coli	4,200 (3,100 - 5,600)	20 (15 - 26)	1,100 (735 - 1,500)	5 (4 - 7)
Uganda	Group A Strep	223 (132 - 367)	1 (1 - 2)	21 (0 - 75)	0 (0 - 0)
Uganda	Group B Strep	1,400 (1,000 - 2,000)	4 (3 - 6)	208 (31 - 423)	1 (0 - 1)
Uganda	Haemophilus influenzae	483 (328 - 687)	2 (1 - 2)	98 (28 - 178)	0 (0 - 1)
Uganda	Klebsiella pneumoniae	5,700 (4,300 - 7,600)	23 (17 - 30)	1,500 (911 - 2,300)	6 (4 - 9)
Uganda	Morganella spp	5 (3 - 9)	0 (0 - 0)	1 (1 - 2)	0 (0 - 0)
Uganda	Mycobacterium tuberculosis	1,200 (353 - 3,100)	7 (2 - 18)	635 (57 - 2,200)	4 (0 - 12)
Uganda	Neisseria Gonorrheae				
Uganda	Non Typhoidal Salmonellae	1 (0 - 4)	0 (0 - 0)	0 (0 - 1)	0 (0 - 0)
Uganda	Proteus spp	355 (227 - 532)	2 (1 - 4)	46 (25 - 75)	0 (0 - 1)
Uganda	Pseudomonas aeruginosa	1,200 (842 - 1,600)	5 (4 - 7)	322 (188 - 527)	1 (1 - 2)

Uganda	Salmonella Paratyphi	0 (0 - 0)	0 (0 - 0)	0 (0 - 0)	0 (0 - 0)
Uganda	Salmonella Typhi	1,800 (1,100 - 2,800)	4 (2 - 5)	274 (3 - 607)	1 (0 - 1)
Uganda	Serratia spp	529 (337 - 789)	2 (1 - 3)	129 (68 - 215)	0 (0 - 1)
Uganda	Shigella spp	71 (19 - 179)	0 (0 - 1)	15 (1 - 45)	0 (0 - 0)
Uganda	Staphylococcus Aureus	4,200 (3,200 - 5,400)	21 (16 - 27)	659 (431 - 943)	3 (2 - 5)
Uganda	Streptococcus pneumoniae	5,100 (3,900 - 6,800)	18 (14 - 21)	1,100 (638 - 1,600)	4 (2 - 5)
Zambia	Acinetobacter baumannii	788 (478 - 1,200)	12 (7 - 19)	239 (138 - 390)	4 (2 - 6)
Zambia	All	16,000 (12,000 - 21,000)	165 (122 - 219)	3,700 (2,600 - 5,100)	40 (28 - 55)
Zambia	Citrobacter spp	87 (53 - 136)	1 (1 - 1)	26 (14 - 44)	0 (0 - 1)
Zambia	Enterobacter spp	467 (324 - 678)	6 (4 - 8)	110 (71 - 165)	1 (1 - 2)
Zambia	Enterococcus faecalis	299 (184 - 458)	3 (2 - 5)	78 (37 - 135)	1 (0 - 2)
Zambia	Enterococcus faecium	346 (209 - 564)	5 (3 - 8)	89 (43 - 157)	1 (1 - 2)
Zambia	Enterococcus spp	122 (77 - 196)	1 (1 - 2)	25 (6 - 50)	0 (0 - 1)
Zambia	Escherichia coli	2,200 (1,600 - 2,900)	25 (18 - 33)	574 (400 - 796)	7 (5 - 9)
Zambia	Group A Strep	77 (41 - 132)	1 (0 - 2)	7 (0 - 26)	0 (0 - 0)
Zambia	Group B Strep	501 (371 - 687)	4 (3 - 6)	72 (7 - 148)	1 (0 - 1)
Zambia	Haemophilus influenzae	176 (126 - 246)	1 (1 - 2)	36 (10 - 67)	0 (0 - 1)
Zambia	Klebsiella pneumoniae	2,800 (2,100 - 3,700)	29 (21 - 38)	720 (443 - 1,100)	7 (5 - 11)
Zambia	Morganella spp	3 (2 - 5)	0 (0 - 0)	1 (0 - 1)	0 (0 - 0)
Zambia	Mycobacterium tuberculosis	679 (201 - 1,900)	8 (2 - 22)	348 (37 - 1,200)	4 (0 - 14)
Zambia	Neisseria Gonorrheae				
Zambia	Non Typhoidal Salmonellae	1 (0 - 3)	0 (0 - 0)	0 (0 - 1)	0 (0 - 0)
Zambia	Proteus spp	189 (116 - 289)	3 (2 - 5)	25 (13 - 42)	0 (0 - 1)
Zambia	Pseudomonas aeruginosa	756 (540 - 1,100)	9 (6 - 12)	193 (119 - 309)	2 (1 - 4)
Zambia	Salmonella Paratyphi	0 (0 - 0)	0 (0 - 0)	0 (0 - 0)	0 (0 - 0)
Zambia	Salmonella Typhi	866 (558 - 1,300)	4 (3 - 6)	124 (0 - 275)	1 (0 - 1)
Zambia	Serratia spp	197 (123 - 302)	2 (1 - 3)	48 (26 - 80)	0 (0 - 1)
Zambia	Shigella spp	79 (19 - 195)	1 (0 - 2)	16 (2 - 51)	0 (0 - 0)
Zambia	Staphylococcus Aureus	2,600 (2,000 - 3,400)	32 (25 - 42)	485 (320 - 696)	6 (4 - 8)
Zambia	Streptococcus pneumoniae	2,400 (1,900 - 3,000)	20 (16 - 24)	457 (279 - 678)	4 (2 - 6)
Zimbabwe	Acinetobacter baumannii	970 (587 - 1,500)	15 (9 - 24)	297 (173 - 470)	5 (3 - 7)
Zimbabwe	All	16,000 (12,000 - 21,000)	182 (138 - 239)	3,900 (2,700 - 5,800)	45 (31 - 69)
Zimbabwe	Citrobacter spp	53 (32 - 86)	1 (0 - 1)	15 (8 - 27)	0 (0 - 0)
Zimbabwe	Enterobacter spp	401 (279 - 581)	5 (3 - 7)	83 (54 - 122)	1 (1 - 2)
Zimbabwe	Enterococcus faecalis	212 (123 - 342)	3 (2 - 4)	56 (26 - 101)	1 (0 - 1)
Zimbabwe	Enterococcus faecium	291 (171 - 495)	4 (2 - 7)	70 (30 - 133)	1 (0 - 2)
Zimbabwe	Enterococcus spp	101 (61 - 165)	1 (1 - 2)	21 (5 - 43)	0 (0 - 1)
Zimbabwe	Escherichia coli	2,100 (1,500 - 2,700)	25 (18 - 34)	495 (338 - 703)	6 (4 - 9)
Zimbabwe	Group A Strep	41 (21 - 76)	1 (0 - 1)	4 (0 - 14)	0 (0 - 0)
Zimbabwe	Group B Strep	588 (428 - 802)	5 (4 - 7)	80 (3 - 172)	1 (0 - 2)
Zimbabwe	Haemophilus influenzae	264 (193 - 351)	2 (2 - 3)	54 (17 - 96)	1 (0 - 1)
Zimbabwe	Klebsiella pneumoniae	2,900 (2,200 - 3,700)	32 (25 - 42)	750 (457 - 1,100)	8 (5 - 13)
Zimbabwe	Morganella spp	3 (1 - 4)	0 (0 - 0)	1 (0 - 1)	0 (0 - 0)
Zimbabwe	Mycobacterium tuberculosis	1,500 (461 - 3,700)	17 (5 - 43)	700 (68 - 2,300)	8 (1 - 27)
Zimbabwe	Neisseria Gonorrheae				
Zimbabwe	Non Typhoidal Salmonellae	2 (0 - 6)	0 (0 - 0)	0 (0 - 2)	0 (0 - 0)
Zimbabwe	Proteus spp	157 (92 - 249)	3 (2 - 4)	19 (9 - 33)	0 (0 - 1)
Zimbabwe	Pseudomonas aeruginosa	838 (623 - 1,100)	10 (7 - 13)	215 (144 - 321)	3 (2 - 4)
Zimbabwe	Salmonella Paratyphi	0 (0 - 0)	0 (0 - 0)	0 (0 - 0)	0 (0 - 0)
Zimbabwe	Salmonella Typhi	324 (126 - 568)	2 (1 - 4)	50 (0 - 132)	0 (0 - 1)
Zimbabwe	Serratia spp	120 (76 - 188)	1 (1 - 2)	24 (12 - 40)	0 (0 - 1)
Zimbabwe	Shigella spp	10 (3 - 26)	0 (0 - 0)	2 (0 - 7)	0 (0 - 0)
Zimbabwe	Staphylococcus Aureus	2,200 (1,700 - 2,700)	27 (21 - 35)	372 (246 - 523)	5 (3 - 7)
Zimbabwe	Streptococcus pneumoniae	2,800 (2,200 - 3,500)	27 (22 - 33)	576 (375 - 805)	6 (4 - 8)

Supplementary Table 3. Mortality attributable antimicrobial resistance (expressed as counts and age-standardised rates (ASMR) per 100 000 with 95% uncertainty intervals) per country in the WHO African region and top 10 pathogen–drug combinations.

Pathogen	Antibiotic class	Country	Deaths attributable to AMR (counts)	Deaths attributable to AMR (age-standardised rate per 100 thousand)
Staphylococcus aureus	Methicillin	Algeria	470.54 (206.12 - 839.79)	1.74 (0.77 - 3.09)
Klebsiella pneumoniae	Third-generation cephalosporins	Algeria	305.34 (84.41 - 649.09)	1.09 (0.3 - 2.3)
Streptococcus pneumoniae	Carbapenems	Algeria	190.64 (83.58 - 348.15)	0.65 (0.29 - 1.17)
Acinetobacter baumannii	Carbapenems	Algeria	159.88 (73.47 - 304.92)	0.59 (0.27 - 1.12)
Acinetobacter baumannii	Fluoroquinolones	Algeria	123.24 (56.51 - 215.03)	0.45 (0.21 - 0.78)
Enterococcus faecium	Fluoroquinolones	Algeria	113.93 (32.29 - 221.47)	0.4 (0.11 - 0.78)
Escherichia coli	Fluoroquinolones	Algeria	113.66 (65.79 - 187.75)	0.42 (0.24 - 0.68)
Escherichia coli	Trimethoprim-Sulfamethoxazole	Algeria	102.29 (59.64 - 164.5)	0.38 (0.22 - 0.6)
Pseudomonas aeruginosa	Carbapenems	Algeria	100.31 (53.45 - 165.43)	0.36 (0.2 - 0.59)
Staphylococcus aureus	Macrolide	Algeria	98.46 (36.61 - 183.92)	0.36 (0.14 - 0.67)
Streptococcus pneumoniae	Trimethoprim-Sulfamethoxazole	Angola	402.5 (57.37 - 809.48)	1.78 (0.26 - 3.46)
Klebsiella pneumoniae	Third-generation cephalosporins	Angola	384.69 (101.78 - 781.75)	2.22 (0.57 - 4.57)
Staphylococcus aureus	Methicillin	Angola	212.34 (87.62 - 381.6)	1.42 (0.6 - 2.57)
Streptococcus pneumoniae	Carbapenems	Angola	212.96 (91.85 - 411.72)	0.94 (0.42 - 1.73)
Klebsiella pneumoniae	Aminoglycosides	Angola	187.36 (108.08 - 299.12)	1.08 (0.63 - 1.74)
Escherichia coli	Trimethoprim-Sulfamethoxazole	Angola	179.44 (118.6 - 254.53)	1.14 (0.78 - 1.62)
Staphylococcus aureus	Trimethoprim-Sulfamethoxazole	Angola	171.6 (88.08 - 276.99)	1.15 (0.59 - 1.84)
Klebsiella pneumoniae	Fluoroquinolones	Angola	160.53 (91.48 - 251.43)	0.92 (0.53 - 1.47)
Klebsiella pneumoniae	Trimethoprim-Sulfamethoxazole	Angola	160.42 (77.54 - 267.36)	0.92 (0.44 - 1.56)
Acinetobacter baumannii	Anti-pseudomonal penicillin/Beta-Lactamase inhibitors	Angola	142.15 (68.5 - 241.01)	1.24 (0.58 - 2.2)
Klebsiella pneumoniae	Third-generation cephalosporins	Benin	270.91 (76.11 - 533.47)	2.86 (0.8 - 5.79)
Streptococcus pneumoniae	Trimethoprim-Sulfamethoxazole	Benin	253.83 (32.67 - 523.7)	2.22 (0.3 - 4.45)
Staphylococcus aureus	Methicillin	Benin	189.28 (75.31 - 338.88)	2.41 (0.96 - 4.25)
Staphylococcus aureus	Trimethoprim-Sulfamethoxazole	Benin	148.7 (75.95 - 234.6)	1.89 (0.99 - 2.92)
Streptococcus pneumoniae	Carbapenems	Benin	140.15 (56.62 - 273.45)	1.23 (0.52 - 2.3)
Klebsiella pneumoniae	Aminoglycosides	Benin	133.13 (77.3 - 204.9)	1.41 (0.81 - 2.16)
Klebsiella pneumoniae	Fluoroquinolones	Benin	120.67 (69.21 - 190.51)	1.27 (0.74 - 1.96)
Klebsiella pneumoniae	Trimethoprim-Sulfamethoxazole	Benin	112.72 (54.11 - 188.65)	1.19 (0.58 - 1.95)
Escherichia coli	Trimethoprim-Sulfamethoxazole	Benin	112.29 (73.45 - 160.39)	1.32 (0.88 - 1.86)
Escherichia coli	Third-generation cephalosporins	Benin	92.23 (35.7 - 188.51)	1.09 (0.42 - 2.21)
Klebsiella pneumoniae	Third-generation cephalosporins	Botswana	39.97 (11.33 - 85.76)	2.87 (0.8 - 6.19)
Streptococcus pneumoniae	Trimethoprim-Sulfamethoxazole	Botswana	23.83 (3.24 - 49.19)	1.49 (0.21 - 3.06)
Streptococcus pneumoniae	Carbapenems	Botswana	21.2 (9.43 - 38.78)	1.33 (0.6 - 2.38)
Staphylococcus aureus	Trimethoprim-Sulfamethoxazole	Botswana	16.83 (8.27 - 27.47)	1.27 (0.63 - 2.06)
Escherichia coli	Trimethoprim-Sulfamethoxazole	Botswana	15.99 (10.41 - 23.71)	1.22 (0.79 - 1.78)
Pseudomonas aeruginosa	Carbapenems	Botswana	15.77 (9.84 - 24.17)	1.17 (0.73 - 1.78)
Klebsiella pneumoniae	Trimethoprim-Sulfamethoxazole	Botswana	14.75 (7.24 - 25.05)	1.06 (0.52 - 1.8)
Acinetobacter baumannii	Carbapenems	Botswana	14.61 (7.22 - 26.12)	1.18 (0.59 - 2.13)
Acinetobacter baumannii	Fluoroquinolones	Botswana	13.01 (6.37 - 22.42)	1.05 (0.51 - 1.81)
Escherichia coli	Third-generation cephalosporins	Botswana	12.64 (4.61 - 25.66)	0.96 (0.35 - 1.94)
Klebsiella pneumoniae	Third-generation cephalosporins	Burkina Faso	697.56 (230.76 - 1390.68)	3.71 (1.21 - 7.24)
Streptococcus pneumoniae	Trimethoprim-Sulfamethoxazole	Burkina Faso	534.74 (68.91 - 1134.75)	2.31 (0.3 - 4.74)
Staphylococcus aureus	Methicillin	Burkina Faso	425.62 (176.74 - 768.62)	2.73 (1.16 - 4.8)

Staphylococcus aureus	Trimethoprim-Sulfamethoxazole	Burkina Faso	302.1 (151.47 - 457.73)	1.94 (0.99 - 2.96)
Klebsiella pneumoniae	Fluoroquinolones	Burkina Faso	281.43 (166.49 - 441.31)	1.49 (0.91 - 2.29)
Klebsiella pneumoniae	Aminoglycosides	Burkina Faso	280.93 (168.16 - 427.76)	1.49 (0.89 - 2.27)
Klebsiella pneumoniae	Trimethoprim-Sulfamethoxazole	Burkina Faso	273.86 (134.86 - 458.82)	1.45 (0.71 - 2.43)
Streptococcus pneumoniae	Carbapenems	Burkina Faso	272.23 (113.64 - 495.88)	1.18 (0.5 - 2.1)
Escherichia coli	Trimethoprim-Sulfamethoxazole	Burkina Faso	219.52 (148.38 - 310.69)	1.35 (0.93 - 1.88)
Escherichia coli	Third-generation cephalosporins	Burkina Faso	214.58 (83.4 - 441.63)	1.32 (0.53 - 2.67)
Klebsiella pneumoniae	Third-generation cephalosporins	Burundi	186.35 (46.71 - 426.51)	2.67 (0.68 - 6.07)
Streptococcus pneumoniae	Trimethoprim-Sulfamethoxazole	Burundi	147.49 (22.0 - 301.81)	1.8 (0.27 - 3.65)
Staphylococcus aureus	Methicillin	Burundi	129.7 (52.37 - 234.27)	2.24 (0.91 - 4.03)
Streptococcus pneumoniae	Fluoroquinolones	Burundi	126.22 (27.52 - 266.61)	1.54 (0.35 - 3.17)
Klebsiella pneumoniae	Aminoglycosides	Burundi	115.99 (69.17 - 181.37)	1.66 (0.99 - 2.58)
Staphylococcus aureus	Trimethoprim-Sulfamethoxazole	Burundi	110.83 (56.82 - 172.71)	1.92 (0.97 - 2.95)
Escherichia coli	Trimethoprim-Sulfamethoxazole	Burundi	91.65 (58.5 - 132.77)	1.33 (0.88 - 1.88)
Acinetobacter baumannii	Anti-pseudomonal penicillin/Beta-Lactamase inhibitors	Burundi	84.82 (41.71 - 144.35)	1.97 (0.95 - 3.42)
Klebsiella pneumoniae	Trimethoprim-Sulfamethoxazole	Burundi	80.49 (36.68 - 136.02)	1.15 (0.54 - 1.93)
Streptococcus pneumoniae	Carbapenems	Burundi	78.6 (32.18 - 149.49)	0.96 (0.4 - 1.75)
Staphylococcus aureus	Methicillin	Cameroon	437.42 (184.17 - 754.86)	2.92 (1.23 - 5.05)
Streptococcus pneumoniae	Trimethoprim-Sulfamethoxazole	Cameroon	416.68 (58.35 - 860.41)	2.01 (0.28 - 4.09)
Klebsiella pneumoniae	Third-generation cephalosporins	Cameroon	268.61 (64.6 - 669.0)	1.54 (0.37 - 3.83)
Staphylococcus aureus	Trimethoprim-Sulfamethoxazole	Cameroon	256.77 (136.29 - 398.11)	1.71 (0.89 - 2.61)
Escherichia coli	Third-generation cephalosporins	Cameroon	218.36 (87.67 - 420.84)	1.32 (0.51 - 2.55)
Klebsiella pneumoniae	Fluoroquinolones	Cameroon	200.44 (118.71 - 310.58)	1.15 (0.68 - 1.8)
Klebsiella pneumoniae	Aminoglycosides	Cameroon	198.95 (111.85 - 304.54)	1.14 (0.63 - 1.78)
Streptococcus pneumoniae	Carbapenems	Cameroon	196.72 (82.68 - 364.46)	0.95 (0.39 - 1.72)
Escherichia coli	Trimethoprim-Sulfamethoxazole	Cameroon	188.78 (122.96 - 274.7)	1.14 (0.75 - 1.67)
Klebsiella pneumoniae	Trimethoprim-Sulfamethoxazole	Cameroon	183.42 (89.66 - 304.08)	1.05 (0.51 - 1.74)
Staphylococcus aureus	Methicillin	Cape Verde	6.97 (2.94 - 11.91)	1.62 (0.68 - 2.77)
Acinetobacter baumannii	Carbapenems	Cape Verde	5.9 (2.99 - 10.53)	1.39 (0.71 - 2.49)
Acinetobacter baumannii	Fluoroquinolones	Cape Verde	5.1 (2.44 - 8.63)	1.2 (0.58 - 2.05)
Klebsiella pneumoniae	Third-generation cephalosporins	Cape Verde	4.81 (1.46 - 10.07)	1.12 (0.34 - 2.34)
Streptococcus pneumoniae	Trimethoprim-Sulfamethoxazole	Cape Verde	4.22 (0.56 - 8.29)	0.95 (0.13 - 1.87)
Streptococcus pneumoniae	Carbapenems	Cape Verde	3.87 (1.77 - 6.69)	0.87 (0.4 - 1.51)
Escherichia coli	Third-generation cephalosporins	Cape Verde	2.87 (1.13 - 5.39)	0.67 (0.27 - 1.25)
Escherichia coli	Trimethoprim-Sulfamethoxazole	Cape Verde	2.39 (1.52 - 3.43)	0.56 (0.36 - 0.8)
Pseudomonas aeruginosa	Carbapenems	Cape Verde	2.19 (1.38 - 3.3)	0.51 (0.32 - 0.77)
Acinetobacter baumannii	Anti-pseudomonal penicillin/Beta-Lactamase inhibitors	Cape Verde	2.19 (0.91 - 3.98)	0.52 (0.21 - 0.94)
Streptococcus pneumoniae	Fluoroquinolones	Central African Republic	203.61 (53.59 - 412.44)	4.19 (1.11 - 8.46)
Mycobacterium tuberculosis	Isoniazid mono-resistance	Central African Republic	124.64 (0.0 - 455.2)	4.05 (0.0 - 15.12)
Klebsiella pneumoniae	Third-generation cephalosporins	Central African Republic	121.2 (29.68 - 255.21)	3.18 (0.76 - 6.87)
Streptococcus pneumoniae	Trimethoprim-Sulfamethoxazole	Central African Republic	102.13 (16.28 - 214.22)	2.09 (0.32 - 4.31)
Group B Streptococcus	Fluoroquinolones	Central African Republic	92.97 (15.4 - 205.74)	1.7 (0.29 - 3.66)

Klebsiella pneumoniae	Aminoglycosides	Central African Republic	88.03 (51.55 - 135.69)	2.31 (1.36 - 3.66)
Klebsiella pneumoniae	Fluoroquinolones	Central African Republic	71.94 (40.77 - 109.64)	1.89 (1.07 - 2.92)
Staphylococcus aureus	Trimethoprim-Sulfamethoxazole	Central African Republic	65.78 (30.64 - 109.41)	2.01 (0.92 - 3.28)
Streptococcus pneumoniae	Beta Lactam/Beta-lactamase inhibitors	Central African Republic	64.93 (0.75 - 152.45)	1.34 (0.02 - 3.09)
Streptococcus pneumoniae	Carbapenems	Central African Republic	62.44 (25.39 - 123.2)	1.28 (0.52 - 2.47)
Streptococcus pneumoniae	Trimethoprim-Sulfamethoxazole	Chad	612.78 (86.57 - 1255.08)	3.02 (0.42 - 6.08)
Klebsiella pneumoniae	Third-generation cephalosporins	Chad	534.31 (192.73 - 998.52)	3.63 (1.28 - 6.86)
Streptococcus pneumoniae	Carbapenems	Chad	304.95 (114.8 - 561.94)	1.5 (0.58 - 2.68)
Streptococcus pneumoniae	Fluoroquinolones	Chad	301.54 (68.61 - 632.82)	1.49 (0.34 - 3.09)
Group B Streptococcus	Fluoroquinolones	Chad	243.99 (41.25 - 521.54)	1.14 (0.2 - 2.43)
Escherichia coli	Third-generation cephalosporins	Chad	220.74 (91.76 - 409.18)	1.57 (0.65 - 2.93)
Escherichia coli	Fluoroquinolones	Chad	218.53 (152.52 - 307.58)	1.55 (1.09 - 2.15)
Klebsiella pneumoniae	Aminoglycosides	Chad	211.45 (127.58 - 312.87)	1.44 (0.87 - 2.15)
Staphylococcus aureus	Methicillin	Chad	173.29 (69.13 - 302.79)	1.44 (0.59 - 2.55)
Acinetobacter baumannii	Anti-pseudomonal penicillin/Beta-Lactamase inhibitors	Chad	162.06 (85.67 - 262.75)	1.88 (0.93 - 3.1)
Acinetobacter baumannii	Anti-pseudomonal penicillin/Beta-Lactamase inhibitors	Comoros	10.72 (5.37 - 18.28)	2.37 (1.18 - 4.13)
Streptococcus pneumoniae	Trimethoprim-Sulfamethoxazole	Comoros	10.37 (1.38 - 20.27)	1.8 (0.24 - 3.47)
Klebsiella pneumoniae	Third-generation cephalosporins	Comoros	8.31 (2.22 - 18.1)	1.6 (0.43 - 3.44)
Streptococcus pneumoniae	Carbapenems	Comoros	7.18 (3.18 - 13.0)	1.24 (0.55 - 2.25)
Escherichia coli	Third-generation cephalosporins	Comoros	5.52 (2.27 - 10.8)	1.1 (0.45 - 2.14)
Staphylococcus aureus	Trimethoprim-Sulfamethoxazole	Comoros	5.0 (2.58 - 7.85)	1.01 (0.53 - 1.59)
Staphylococcus aureus	Methicillin	Comoros	4.79 (1.81 - 8.66)	0.96 (0.36 - 1.74)
Klebsiella pneumoniae	Aminoglycosides	Comoros	4.73 (2.79 - 7.36)	0.91 (0.53 - 1.43)
Escherichia coli	Trimethoprim-Sulfamethoxazole	Comoros	4.36 (2.99 - 6.08)	0.87 (0.6 - 1.2)
Klebsiella pneumoniae	Fluoroquinolones	Comoros	4.04 (2.39 - 6.33)	0.78 (0.46 - 1.22)
Klebsiella pneumoniae	Third-generation cephalosporins	Congo	64.09 (18.88 - 131.92)	2.37 (0.7 - 4.94)
Staphylococcus aureus	Methicillin	Congo	63.67 (26.39 - 113.41)	2.58 (1.06 - 4.54)
Streptococcus pneumoniae	Trimethoprim-Sulfamethoxazole	Congo	46.43 (6.8 - 97.17)	1.52 (0.23 - 3.09)
Staphylococcus aureus	Trimethoprim-Sulfamethoxazole	Congo	32.61 (16.67 - 51.95)	1.32 (0.68 - 2.08)
Escherichia coli	Third-generation cephalosporins	Congo	31.49 (12.2 - 62.25)	1.21 (0.46 - 2.41)
Klebsiella pneumoniae	Aminoglycosides	Congo	30.9 (18.22 - 49.84)	1.14 (0.67 - 1.83)
Klebsiella pneumoniae	Fluoroquinolones	Congo	30.28 (17.49 - 47.96)	1.12 (0.66 - 1.77)
Escherichia coli	Trimethoprim-Sulfamethoxazole	Congo	27.82 (18.43 - 40.48)	1.07 (0.72 - 1.54)
Acinetobacter baumannii	Anti-pseudomonal penicillin/Beta-Lactamase inhibitors	Congo	26.85 (12.88 - 47.24)	1.27 (0.6 - 2.23)
Escherichia coli	Fluoroquinolones	Congo	26.38 (16.84 - 39.45)	1.01 (0.66 - 1.5)
Klebsiella pneumoniae	Third-generation cephalosporins	Cote d'Ivoire	494.93 (143.72 - 993.63)	2.87 (0.82 - 5.66)
Streptococcus pneumoniae	Trimethoprim-Sulfamethoxazole	Cote d'Ivoire	424.85 (53.97 - 867.87)	2.13 (0.28 - 4.2)
Staphylococcus aureus	Methicillin	Cote d'Ivoire	310.64 (127.62 - 562.94)	2.15 (0.9 - 3.83)
Staphylococcus aureus	Trimethoprim-Sulfamethoxazole	Cote d'Ivoire	280.89 (146.15 - 432.65)	1.94 (1.03 - 2.97)
Streptococcus pneumoniae	Carbapenems	Cote d'Ivoire	236.38 (98.72 - 436.25)	1.18 (0.52 - 2.12)
Klebsiella pneumoniae	Aminoglycosides	Cote d'Ivoire	232.72 (136.62 - 350.31)	1.35 (0.79 - 2.03)
Escherichia coli	Third-generation cephalosporins	Cote d'Ivoire	219.72 (89.03 - 422.3)	1.39 (0.56 - 2.7)

Klebsiella pneumoniae	Fluoroquinolones	Cote d'Ivoire	208.56 (120.68 - 317.0)	1.21 (0.71 - 1.86)
Klebsiella pneumoniae	Trimethoprim-Sulfamethoxazole	Cote d'Ivoire	196.1 (94.23 - 320.85)	1.14 (0.55 - 1.9)
Escherichia coli	Trimethoprim-Sulfamethoxazole	Cote d'Ivoire	188.43 (124.04 - 265.05)	1.19 (0.79 - 1.66)
Klebsiella pneumoniae	Third-generation cephalosporins	Democratic Republic of the Congo	1356.12 (417.57 - 2777.6)	2.81 (0.85 - 5.79)
Streptococcus pneumoniae	Fluoroquinolones	Democratic Republic of the Congo	978.65 (243.68 - 2017.09)	1.76 (0.44 - 3.59)
Staphylococcus aureus	Methicillin	Democratic Republic of the Congo	930.88 (394.41 - 1691.53)	2.24 (0.93 - 3.97)
Streptococcus pneumoniae	Trimethoprim-Sulfamethoxazole	Democratic Republic of the Congo	884.09 (127.98 - 1797.26)	1.59 (0.24 - 3.17)
Staphylococcus aureus	Trimethoprim-Sulfamethoxazole	Democratic Republic of the Congo	652.17 (334.58 - 1025.97)	1.57 (0.81 - 2.48)
Klebsiella pneumoniae	Aminoglycosides	Democratic Republic of the Congo	629.17 (366.08 - 968.55)	1.3 (0.75 - 2.03)
Escherichia coli	Third-generation cephalosporins	Democratic Republic of the Congo	603.64 (251.6 - 1167.17)	1.31 (0.54 - 2.55)
Escherichia coli	Fluoroquinolones	Democratic Republic of the Congo	556.96 (373.22 - 805.72)	1.21 (0.81 - 1.77)
Mycobacterium tuberculosis	Isoniazid mono-resistance	Democratic Republic of the Congo	530.14 (0.0 - 1884.99)	1.19 (0.0 - 4.28)
Acinetobacter baumannii	Anti-pseudomonal penicillin/Beta-Lactamase inhibitors	Democratic Republic of the Congo	529.63 (269.78 - 887.91)	1.65 (0.79 - 2.85)
Streptococcus pneumoniae	Trimethoprim-Sulfamethoxazole	Equatorial Guinea	9.1 (1.35 - 19.02)	1.23 (0.19 - 2.45)
Staphylococcus aureus	Methicillin	Equatorial Guinea	8.58 (3.29 - 15.59)	1.52 (0.6 - 2.74)
Klebsiella pneumoniae	Third-generation cephalosporins	Equatorial Guinea	7.68 (1.79 - 18.48)	1.21 (0.28 - 2.86)
Acinetobacter baumannii	Carbapenems	Equatorial Guinea	6.86 (3.54 - 12.18)	1.22 (0.63 - 2.21)
Acinetobacter baumannii	Fluoroquinolones	Equatorial Guinea	6.84 (3.29 - 12.15)	1.22 (0.57 - 2.12)
Streptococcus pneumoniae	Carbapenems	Equatorial Guinea	5.37 (2.25 - 10.71)	0.73 (0.31 - 1.37)
Escherichia coli	Third-generation cephalosporins	Equatorial Guinea	4.74 (1.8 - 9.55)	0.85 (0.33 - 1.68)
Klebsiella pneumoniae	Fluoroquinolones	Equatorial Guinea	4.23 (2.28 - 6.99)	0.67 (0.36 - 1.08)
Klebsiella pneumoniae	Aminoglycosides	Equatorial Guinea	3.95 (2.08 - 6.78)	0.62 (0.34 - 1.08)
Escherichia coli	Fluoroquinolones	Equatorial Guinea	3.94 (2.37 - 6.19)	0.71 (0.44 - 1.09)
Klebsiella pneumoniae	Third-generation cephalosporins	Eritrea	98.32 (24.17 - 218.64)	3.02 (0.74 - 6.8)
Streptococcus pneumoniae	Trimethoprim-Sulfamethoxazole	Eritrea	88.39 (12.66 - 189.37)	2.23 (0.32 - 4.72)
Staphylococcus aureus	Trimethoprim-Sulfamethoxazole	Eritrea	82.09 (38.09 - 139.62)	2.87 (1.35 - 5.04)
Streptococcus pneumoniae	Fluoroquinolones	Eritrea	69.5 (15.53 - 148.9)	1.75 (0.4 - 3.75)

Acinetobacter baumannii	Anti-pseudomonal penicillin/Beta-Lactamase inhibitors	Eritrea	60.46 (29.82 - 107.7)	2.68 (1.29 - 4.59)
Klebsiella pneumoniae	Aminoglycosides	Eritrea	58.98 (33.86 - 95.28)	1.81 (1.04 - 2.91)
Escherichia coli	Third-generation cephalosporins	Eritrea	58.31 (23.91 - 118.23)	1.85 (0.77 - 3.64)
Staphylococcus aureus	Methicillin	Eritrea	56.37 (21.24 - 107.43)	1.97 (0.74 - 3.88)
Streptococcus pneumoniae	Carbapenems	Eritrea	47.47 (18.74 - 90.27)	1.2 (0.48 - 2.28)
Escherichia coli	Trimethoprim-Sulfamethoxazole	Eritrea	43.62 (28.17 - 65.25)	1.38 (0.92 - 1.99)
Klebsiella pneumoniae	Third-generation cephalosporins	Ethiopia	1703.02 (686.51 - 3166.63)	2.69 (1.08 - 4.98)
Streptococcus pneumoniae	Trimethoprim-Sulfamethoxazole	Ethiopia	1435.24 (203.61 - 2881.8)	1.89 (0.28 - 3.73)
Staphylococcus aureus	Methicillin	Ethiopia	1273.69 (551.89 - 2146.54)	2.36 (1.03 - 4.03)
Escherichia coli	Third-generation cephalosporins	Ethiopia	1183.36 (517.71 - 2165.81)	2.07 (0.93 - 3.73)
Klebsiella pneumoniae	Carbapenems	Ethiopia	1149.53 (684.34 - 1773.57)	1.82 (1.07 - 2.79)
Klebsiella pneumoniae	Aminoglycosides	Ethiopia	869.99 (548.43 - 1278.59)	1.38 (0.87 - 2.04)
Escherichia coli	Fluoroquinolones	Ethiopia	820.76 (586.0 - 1125.37)	1.44 (1.02 - 1.97)
Streptococcus pneumoniae	Carbapenems	Ethiopia	805.65 (350.7 - 1455.2)	1.06 (0.46 - 1.91)
Escherichia coli	Trimethoprim-Sulfamethoxazole	Ethiopia	659.68 (454.83 - 897.72)	1.15 (0.81 - 1.54)
Staphylococcus aureus	Trimethoprim-Sulfamethoxazole	Ethiopia	636.95 (331.95 - 984.64)	1.18 (0.62 - 1.85)
Staphylococcus aureus	Methicillin	Gabon	19.27 (8.54 - 33.22)	1.93 (0.87 - 3.3)
Klebsiella pneumoniae	Third-generation cephalosporins	Gabon	14.43 (3.91 - 32.39)	1.4 (0.37 - 3.11)
Acinetobacter baumannii	Fluoroquinolones	Gabon	13.29 (6.36 - 23.02)	1.45 (0.69 - 2.49)
Streptococcus pneumoniae	Trimethoprim-Sulfamethoxazole	Gabon	13.13 (1.86 - 27.66)	1.15 (0.18 - 2.38)
Acinetobacter baumannii	Carbapenems	Gabon	11.92 (5.93 - 21.41)	1.3 (0.66 - 2.34)
Acinetobacter baumannii	Anti-pseudomonal penicillin/Beta-Lactamase inhibitors	Gabon	10.57 (4.64 - 18.41)	1.15 (0.5 - 2.02)
Klebsiella pneumoniae	Fluoroquinolones	Gabon	7.46 (4.2 - 12.04)	0.72 (0.4 - 1.16)
Escherichia coli	Third-generation cephalosporins	Gabon	7.41 (2.78 - 14.76)	0.74 (0.28 - 1.49)
Streptococcus pneumoniae	Carbapenems	Gabon	6.83 (2.87 - 12.51)	0.6 (0.25 - 1.09)
Escherichia coli	Trimethoprim-Sulfamethoxazole	Gabon	6.71 (4.38 - 9.72)	0.67 (0.44 - 0.96)
Klebsiella pneumoniae	Third-generation cephalosporins	Ghana	470.06 (166.78 - 892.24)	2.52 (0.9 - 4.75)
Streptococcus pneumoniae	Trimethoprim-Sulfamethoxazole	Ghana	411.88 (52.84 - 822.78)	2.03 (0.27 - 3.88)
Staphylococcus aureus	Methicillin	Ghana	397.36 (171.39 - 702.77)	2.41 (1.03 - 4.28)
Escherichia coli	Third-generation cephalosporins	Ghana	296.52 (121.82 - 558.37)	1.73 (0.72 - 3.22)
Klebsiella pneumoniae	Aminoglycosides	Ghana	252.63 (154.3 - 384.04)	1.35 (0.84 - 2.04)
Staphylococcus aureus	Trimethoprim-Sulfamethoxazole	Ghana	250.91 (127.83 - 385.37)	1.52 (0.8 - 2.31)
Escherichia coli	Fluoroquinolones	Ghana	232.52 (161.4 - 329.66)	1.36 (0.94 - 1.93)
Klebsiella pneumoniae	Trimethoprim-Sulfamethoxazole	Ghana	191.42 (91.97 - 319.19)	1.02 (0.49 - 1.7)
Klebsiella pneumoniae	Fluoroquinolones	Ghana	191.82 (113.63 - 293.48)	1.03 (0.61 - 1.55)
Acinetobacter baumannii	Anti-pseudomonal penicillin/Beta-Lactamase inhibitors	Ghana	187.31 (85.18 - 335.96)	1.27 (0.56 - 2.3)
Streptococcus pneumoniae	Trimethoprim-Sulfamethoxazole	Guinea	403.35 (55.22 - 855.27)	3.09 (0.43 - 6.54)
Klebsiella pneumoniae	Third-generation cephalosporins	Guinea	292.59 (88.03 - 616.28)	2.9 (0.86 - 6.15)
Streptococcus pneumoniae	Carbapenems	Guinea	217.41 (89.94 - 415.63)	1.67 (0.71 - 3.15)
Streptococcus pneumoniae	Fluoroquinolones	Guinea	202.15 (45.61 - 429.9)	1.55 (0.36 - 3.22)
Group B Streptococcus	Fluoroquinolones	Guinea	174.61 (31.19 - 385.21)	1.32 (0.24 - 2.84)
Staphylococcus aureus	Methicillin	Guinea	138.47 (56.62 - 251.58)	1.58 (0.65 - 2.87)
Klebsiella pneumoniae	Aminoglycosides	Guinea	137.51 (80.78 - 206.85)	1.36 (0.78 - 2.06)
Acinetobacter baumannii	Anti-pseudomonal penicillin/Beta-Lactamase inhibitors	Guinea	124.01 (62.83 - 210.08)	1.82 (0.9 - 3.04)
Escherichia coli	Third-generation cephalosporins	Guinea	122.87 (47.85 - 241.81)	1.33 (0.52 - 2.6)

Staphylococcus aureus	Trimethoprim-Sulfamethoxazole	Guinea	119.81 (62.14 - 189.87)	1.37 (0.71 - 2.2)
Klebsiella pneumoniae	Third-generation cephalosporins	Guinea-Bissau	33.72 (9.41 - 71.49)	3.19 (0.9 - 6.87)
Streptococcus pneumoniae	Trimethoprim-Sulfamethoxazole	Guinea-Bissau	25.94 (3.69 - 53.92)	2.19 (0.31 - 4.41)
Staphylococcus aureus	Trimethoprim-Sulfamethoxazole	Guinea-Bissau	19.96 (10.07 - 31.53)	2.24 (1.13 - 3.53)
Klebsiella pneumoniae	Aminoglycosides	Guinea-Bissau	17.55 (10.48 - 27.21)	1.66 (0.98 - 2.56)
Staphylococcus aureus	Methicillin	Guinea-Bissau	16.33 (6.71 - 28.78)	1.83 (0.76 - 3.26)
Acinetobacter baumannii	Anti-pseudomonal penicillin/Beta-Lactamase inhibitors	Guinea-Bissau	16.19 (7.86 - 27.76)	2.34 (1.1 - 4.14)
Klebsiella pneumoniae	Trimethoprim-Sulfamethoxazole	Guinea-Bissau	15.26 (7.36 - 25.19)	1.44 (0.69 - 2.4)
Klebsiella pneumoniae	Fluoroquinolones	Guinea-Bissau	15.03 (8.65 - 23.07)	1.42 (0.82 - 2.2)
Streptococcus pneumoniae	Fluoroquinolones	Guinea-Bissau	13.84 (3.23 - 29.7)	1.17 (0.27 - 2.51)
Escherichia coli	Third-generation cephalosporins	Guinea-Bissau	13.31 (4.95 - 26.26)	1.35 (0.49 - 2.67)
Klebsiella pneumoniae	Third-generation cephalosporins	Kenya	549.96 (184.65 - 1143.69)	2.23 (0.72 - 4.61)
Streptococcus pneumoniae	Trimethoprim-Sulfamethoxazole	Kenya	482.89 (71.46 - 931.12)	1.69 (0.25 - 3.2)
Streptococcus pneumoniae	Carbapenems	Kenya	409.72 (185.27 - 722.96)	1.43 (0.65 - 2.46)
Escherichia coli	Third-generation cephalosporins	Kenya	408.69 (162.64 - 758.9)	1.74 (0.69 - 3.24)
Staphylococcus aureus	Trimethoprim-Sulfamethoxazole	Kenya	383.01 (196.05 - 596.63)	1.72 (0.88 - 2.69)
Staphylococcus aureus	Methicillin	Kenya	320.81 (129.37 - 579.11)	1.44 (0.58 - 2.61)
Escherichia coli	Trimethoprim-Sulfamethoxazole	Kenya	307.28 (213.14 - 419.78)	1.31 (0.9 - 1.81)
Klebsiella pneumoniae	Aminoglycosides	Kenya	297.28 (179.64 - 456.72)	1.2 (0.71 - 1.85)
Klebsiella pneumoniae	Trimethoprim-Sulfamethoxazole	Kenya	284.22 (139.69 - 466.37)	1.15 (0.56 - 1.88)
Klebsiella pneumoniae	Fluoroquinolones	Kenya	269.58 (156.96 - 404.82)	1.09 (0.63 - 1.63)
Klebsiella pneumoniae	Third-generation cephalosporins	Lesotho	73.09 (27.4 - 137.88)	5.5 (2.07 - 10.48)
Streptococcus pneumoniae	Trimethoprim-Sulfamethoxazole	Lesotho	31.69 (4.79 - 63.57)	2.09 (0.32 - 4.21)
Staphylococcus aureus	Methicillin	Lesotho	27.26 (11.07 - 50.44)	2.14 (0.87 - 3.96)
Staphylococcus aureus	Trimethoprim-Sulfamethoxazole	Lesotho	22.49 (11.11 - 35.97)	1.77 (0.88 - 2.84)
Acinetobacter baumannii	Anti-pseudomonal penicillin/Beta-Lactamase inhibitors	Lesotho	21.53 (9.46 - 39.88)	2.0 (0.86 - 3.67)
Acinetobacter baumannii	Carbapenems	Lesotho	21.25 (9.9 - 39.38)	1.97 (0.9 - 3.68)
Streptococcus pneumoniae	Carbapenems	Lesotho	20.81 (8.96 - 36.92)	1.37 (0.59 - 2.45)
Escherichia coli	Third-generation cephalosporins	Lesotho	19.5 (7.55 - 38.53)	1.54 (0.59 - 3.05)
Klebsiella pneumoniae	Trimethoprim-Sulfamethoxazole	Lesotho	19.46 (9.34 - 32.97)	1.46 (0.69 - 2.47)
Escherichia coli	Trimethoprim-Sulfamethoxazole	Lesotho	19.07 (12.64 - 27.57)	1.51 (1.0 - 2.18)
Klebsiella pneumoniae	Third-generation cephalosporins	Liberia	67.86 (20.71 - 140.86)	2.47 (0.75 - 5.05)
Streptococcus pneumoniae	Trimethoprim-Sulfamethoxazole	Liberia	49.91 (6.29 - 104.7)	1.63 (0.22 - 3.27)
Staphylococcus aureus	Trimethoprim-Sulfamethoxazole	Liberia	38.95 (20.27 - 62.14)	1.65 (0.86 - 2.63)
Klebsiella pneumoniae	Aminoglycosides	Liberia	34.0 (19.37 - 51.83)	1.24 (0.71 - 1.89)
Staphylococcus aureus	Methicillin	Liberia	33.17 (13.72 - 61.18)	1.41 (0.58 - 2.59)
Acinetobacter baumannii	Anti-pseudomonal penicillin/Beta-Lactamase inhibitors	Liberia	32.54 (16.52 - 54.9)	1.66 (0.81 - 2.86)
Klebsiella pneumoniae	Fluoroquinolones	Liberia	29.74 (17.46 - 46.89)	1.08 (0.64 - 1.67)
Escherichia coli	Third-generation cephalosporins	Liberia	28.46 (10.61 - 58.34)	1.12 (0.42 - 2.24)
Escherichia coli	Trimethoprim-Sulfamethoxazole	Liberia	27.32 (17.92 - 39.23)	1.07 (0.72 - 1.53)
Klebsiella pneumoniae	Trimethoprim-Sulfamethoxazole	Liberia	27.23 (13.1 - 45.69)	0.99 (0.48 - 1.66)
Klebsiella pneumoniae	Third-generation cephalosporins	Madagascar	394.27 (133.17 - 758.75)	2.59 (0.87 - 5.02)
Streptococcus pneumoniae	Fluoroquinolones	Madagascar	377.59 (97.3 - 772.25)	2.08 (0.5 - 4.2)

Streptococcus pneumoniae	Trimethoprim-Sulfamethoxazole	Madagascar	287.9 (44.46 - 581.7)	1.58 (0.25 - 3.18)
Staphylococcus aureus	Trimethoprim-Sulfamethoxazole	Madagascar	247.17 (127.46 - 390.9)	1.9 (0.99 - 3.08)
Staphylococcus aureus	Methicillin	Madagascar	211.05 (81.72 - 384.28)	1.62 (0.64 - 2.97)
Escherichia coli	Fluoroquinolones	Madagascar	186.2 (127.43 - 258.94)	1.24 (0.84 - 1.76)
Pseudomonas aeruginosa	Third-generation cephalosporins	Madagascar	179.2 (78.87 - 306.7)	1.27 (0.56 - 2.23)
Klebsiella pneumoniae	Aminoglycosides	Madagascar	177.23 (105.66 - 268.3)	1.17 (0.68 - 1.83)
Escherichia coli	Trimethoprim-Sulfamethoxazole	Madagascar	164.75 (109.79 - 229.58)	1.09 (0.73 - 1.54)
Streptococcus pneumoniae	Carbapenems	Madagascar	156.93 (66.85 - 289.26)	0.86 (0.38 - 1.59)
Klebsiella pneumoniae	Third-generation cephalosporins	Malawi	351.46 (116.7 - 670.43)	3.3 (1.06 - 6.25)
Streptococcus pneumoniae	Trimethoprim-Sulfamethoxazole	Malawi	271.63 (37.97 - 538.24)	2.13 (0.32 - 4.07)
Staphylococcus aureus	Trimethoprim-Sulfamethoxazole	Malawi	160.64 (84.81 - 247.32)	1.75 (0.91 - 2.69)
Klebsiella pneumoniae	Aminoglycosides	Malawi	153.98 (94.24 - 234.0)	1.45 (0.86 - 2.2)
Staphylococcus aureus	Methicillin	Malawi	152.04 (61.29 - 270.76)	1.66 (0.68 - 2.97)
Escherichia coli	Trimethoprim-Sulfamethoxazole	Malawi	148.29 (102.78 - 203.72)	1.54 (1.08 - 2.07)
Streptococcus pneumoniae	Carbapenems	Malawi	134.96 (57.44 - 249.33)	1.06 (0.46 - 1.91)
Klebsiella pneumoniae	Trimethoprim-Sulfamethoxazole	Malawi	126.62 (62.66 - 207.79)	1.19 (0.58 - 1.96)
Acinetobacter baumannii	Anti-pseudomonal penicillin/Beta-Lactamase inhibitors	Malawi	107.66 (49.71 - 188.12)	1.5 (0.66 - 2.64)
Escherichia coli	Beta Lactam/Beta-lactamase inhibitors	Malawi	102.38 (70.66 - 144.87)	1.06 (0.73 - 1.51)
Klebsiella pneumoniae	Third-generation cephalosporins	Mali	753.16 (266.32 - 1460.7)	3.55 (1.25 - 6.91)
Staphylococcus aureus	Methicillin	Mali	418.98 (170.48 - 753.27)	2.33 (0.94 - 4.16)
Streptococcus pneumoniae	Carbapenems	Mali	334.87 (145.01 - 633.19)	1.27 (0.55 - 2.36)
Streptococcus pneumoniae	Trimethoprim-Sulfamethoxazole	Mali	330.65 (46.28 - 681.45)	1.25 (0.17 - 2.54)
Klebsiella pneumoniae	Aminoglycosides	Mali	326.35 (186.21 - 508.63)	1.54 (0.89 - 2.43)
Klebsiella pneumoniae	Fluoroquinolones	Mali	294.85 (173.92 - 459.07)	1.39 (0.81 - 2.18)
Klebsiella pneumoniae	Trimethoprim-Sulfamethoxazole	Mali	273.88 (130.91 - 464.58)	1.29 (0.62 - 2.2)
Escherichia coli	Fluoroquinolones	Mali	264.35 (175.02 - 380.99)	1.48 (0.97 - 2.14)
Staphylococcus aureus	Trimethoprim-Sulfamethoxazole	Mali	251.07 (130.67 - 393.29)	1.4 (0.71 - 2.17)
Escherichia coli	Trimethoprim-Sulfamethoxazole	Mali	246.6 (161.02 - 353.43)	1.38 (0.9 - 1.97)
Staphylococcus aureus	Methicillin	Mauritania	53.99 (23.79 - 93.65)	2.41 (1.06 - 4.12)
Klebsiella pneumoniae	Third-generation cephalosporins	Mauritania	50.2 (15.46 - 98.93)	2.01 (0.63 - 3.95)
Streptococcus pneumoniae	Trimethoprim-Sulfamethoxazole	Mauritania	36.88 (4.86 - 77.5)	1.39 (0.19 - 2.84)
Streptococcus pneumoniae	Carbapenems	Mauritania	24.56 (10.37 - 47.68)	0.93 (0.4 - 1.78)
Escherichia coli	Trimethoprim-Sulfamethoxazole	Mauritania	22.28 (14.31 - 32.38)	0.93 (0.6 - 1.33)
Escherichia coli	Third-generation cephalosporins	Mauritania	21.15 (7.93 - 40.2)	0.88 (0.33 - 1.69)
Klebsiella pneumoniae	Trimethoprim-Sulfamethoxazole	Mauritania	20.83 (9.86 - 34.85)	0.83 (0.4 - 1.41)
Staphylococcus aureus	Trimethoprim-Sulfamethoxazole	Mauritania	20.67 (10.33 - 32.23)	0.92 (0.47 - 1.42)
Klebsiella pneumoniae	Fluoroquinolones	Mauritania	20.51 (11.74 - 32.04)	0.82 (0.47 - 1.27)
Klebsiella pneumoniae	Aminoglycosides	Mauritania	19.16 (10.67 - 30.51)	0.77 (0.43 - 1.22)
Staphylococcus aureus	Methicillin	Mauritius	41.44 (19.31 - 72.2)	2.68 (1.25 - 4.65)
Klebsiella pneumoniae	Third-generation cephalosporins	Mauritius	11.34 (1.81 - 29.26)	0.74 (0.12 - 1.9)
Acinetobacter baumannii	Carbapenems	Mauritius	9.95 (4.65 - 18.78)	0.63 (0.3 - 1.18)
Escherichia coli	Fluoroquinolones	Mauritius	8.81 (5.06 - 13.85)	0.56 (0.32 - 0.88)
Escherichia coli	Third-generation cephalosporins	Mauritius	8.58 (3.22 - 17.59)	0.55 (0.21 - 1.12)
Enterococcus faecium	Fluoroquinolones	Mauritius	8.27 (2.25 - 16.59)	0.5 (0.14 - 1.0)
Streptococcus pneumoniae	Carbapenems	Mauritius	8.26 (3.63 - 14.86)	0.57 (0.25 - 1.03)
Acinetobacter baumannii	Fluoroquinolones	Mauritius	8.09 (3.65 - 14.99)	0.51 (0.23 - 0.95)
Pseudomonas aeruginosa	Carbapenems	Mauritius	7.73 (4.24 - 12.67)	0.5 (0.27 - 0.81)
Escherichia coli	Trimethoprim-Sulfamethoxazole	Mauritius	5.67 (3.22 - 9.17)	0.36 (0.21 - 0.59)
Klebsiella pneumoniae	Third-generation cephalosporins	Mozambique	715.82 (259.74 - 1322.04)	3.83 (1.36 - 7.17)
Staphylococcus aureus	Trimethoprim-Sulfamethoxazole	Mozambique	407.66 (225.76 - 621.45)	2.68 (1.45 - 4.15)
Staphylococcus aureus	Methicillin	Mozambique	404.36 (167.41 - 711.61)	2.66 (1.13 - 4.66)

Streptococcus pneumoniae	Trimethoprim-Sulfamethoxazole	Mozambique	399.16 (51.37 - 811.77)	1.81 (0.26 - 3.68)
Streptococcus pneumoniae	Fluoroquinolones	Mozambique	292.08 (68.06 - 620.44)	1.33 (0.31 - 2.79)
Klebsiella pneumoniae	Aminoglycosides	Mozambique	285.96 (175.23 - 429.58)	1.53 (0.92 - 2.32)
Acinetobacter baumannii	Anti-pseudomonal penicillin/Beta-Lactamase inhibitors	Mozambique	248.14 (114.7 - 434.76)	2.24 (1.02 - 3.99)
Klebsiella pneumoniae	Trimethoprim-Sulfamethoxazole	Mozambique	246.73 (121.81 - 407.76)	1.32 (0.64 - 2.21)
Klebsiella pneumoniae	Fluoroquinolones	Mozambique	228.24 (134.59 - 353.86)	1.22 (0.7 - 1.89)
Streptococcus pneumoniae	Carbapenems	Mozambique	205.99 (88.04 - 387.7)	0.94 (0.41 - 1.7)
Klebsiella pneumoniae	Third-generation cephalosporins	Namibia	35.38 (9.14 - 78.36)	2.33 (0.6 - 5.16)
Staphylococcus aureus	Methicillin	Namibia	24.46 (9.47 - 44.79)	1.72 (0.67 - 3.13)
Streptococcus pneumoniae	Trimethoprim-Sulfamethoxazole	Namibia	21.59 (2.98 - 46.04)	1.29 (0.18 - 2.71)
Staphylococcus aureus	Trimethoprim-Sulfamethoxazole	Namibia	21.27 (10.58 - 33.79)	1.5 (0.75 - 2.37)
Streptococcus pneumoniae	Carbapenems	Namibia	17.79 (7.43 - 32.73)	1.06 (0.44 - 1.94)
Escherichia coli	Trimethoprim-Sulfamethoxazole	Namibia	15.95 (10.32 - 23.5)	1.12 (0.73 - 1.63)
Escherichia coli	Third-generation cephalosporins	Namibia	15.64 (6.18 - 30.21)	1.1 (0.43 - 2.11)
Klebsiella pneumoniae	Trimethoprim-Sulfamethoxazole	Namibia	15.59 (7.55 - 25.99)	1.03 (0.5 - 1.71)
Pseudomonas aeruginosa	Carbapenems	Namibia	15.04 (9.51 - 22.91)	1.02 (0.65 - 1.55)
Acinetobacter baumannii	Fluoroquinolones	Namibia	14.94 (7.36 - 25.91)	1.13 (0.56 - 1.94)
Streptococcus pneumoniae	Trimethoprim-Sulfamethoxazole	Niger	683.56 (92.58 - 1432.28)	2.58 (0.37 - 5.21)
Klebsiella pneumoniae	Third-generation cephalosporins	Niger	603.17 (181.93 - 1264.75)	2.97 (0.92 - 6.19)
Staphylococcus aureus	Methicillin	Niger	401.09 (173.99 - 715.31)	2.46 (1.06 - 4.29)
Streptococcus pneumoniae	Carbapenems	Niger	339.42 (139.75 - 631.2)	1.29 (0.54 - 2.31)
Klebsiella pneumoniae	Aminoglycosides	Niger	311.86 (186.13 - 475.01)	1.54 (0.89 - 2.32)
Streptococcus pneumoniae	Fluoroquinolones	Niger	309.12 (66.39 - 655.08)	1.17 (0.25 - 2.47)
Klebsiella pneumoniae	Fluoroquinolones	Niger	259.9 (151.65 - 404.67)	1.28 (0.74 - 1.98)
Escherichia coli	Trimethoprim-Sulfamethoxazole	Niger	253.31 (171.79 - 354.02)	1.39 (0.94 - 1.94)
Klebsiella pneumoniae	Trimethoprim-Sulfamethoxazole	Niger	249.46 (116.99 - 410.19)	1.23 (0.58 - 2.06)
Group B Streptococcus	Fluoroquinolones	Niger	247.48 (41.77 - 539.28)	0.89 (0.15 - 1.95)
Staphylococcus aureus	Methicillin	Nigeria	4925.23 (2073.76 - 8249.26)	3.26 (1.44 - 5.6)
Streptococcus pneumoniae	Trimethoprim-Sulfamethoxazole	Nigeria	4531.78 (667.63 - 8997.09)	2.04 (0.29 - 3.94)
Klebsiella pneumoniae	Third-generation cephalosporins	Nigeria	4152.5 (1357.22 - 7988.17)	2.41 (0.8 - 4.75)
Escherichia coli	Third-generation cephalosporins	Nigeria	3389.73 (1411.42 - 6037.45)	2.12 (0.88 - 3.86)
Escherichia coli	Fluoroquinolones	Nigeria	2504.61 (1719.16 - 3429.64)	1.56 (1.05 - 2.16)
Streptococcus pneumoniae	Carbapenems	Nigeria	2386.24 (1084.55 - 4284.6)	1.07 (0.49 - 1.87)
Klebsiella pneumoniae	Aminoglycosides	Nigeria	2323.91 (1484.17 - 3446.15)	1.35 (0.83 - 1.99)
Staphylococcus aureus	Trimethoprim-Sulfamethoxazole	Nigeria	2256.46 (1234.8 - 3390.19)	1.5 (0.78 - 2.35)
Klebsiella pneumoniae	Fluoroquinolones	Nigeria	2185.64 (1297.07 - 3323.3)	1.27 (0.75 - 1.93)
Klebsiella pneumoniae	Carbapenems	Nigeria	2183.88 (638.35 - 5252.13)	1.26 (0.36 - 3.01)
Klebsiella pneumoniae	Third-generation cephalosporins	Rwanda	234.19 (90.43 - 422.08)	3.3 (1.26 - 6.02)
Staphylococcus aureus	Methicillin	Rwanda	166.67 (67.79 - 294.19)	2.65 (1.09 - 4.64)
Streptococcus pneumoniae	Trimethoprim-Sulfamethoxazole	Rwanda	135.54 (19.1 - 268.02)	1.62 (0.24 - 3.11)
Staphylococcus aureus	Trimethoprim-Sulfamethoxazole	Rwanda	121.41 (62.51 - 185.66)	1.93 (1.01 - 2.99)
Escherichia coli	Third-generation cephalosporins	Rwanda	113.7 (46.59 - 210.23)	1.74 (0.72 - 3.32)
Klebsiella pneumoniae	Aminoglycosides	Rwanda	94.15 (57.95 - 140.98)	1.33 (0.81 - 1.99)

Streptococcus pneumoniae	Carbapenems	Rwanda	81.09 (34.94 - 151.53)	0.97 (0.43 - 1.78)
Escherichia coli	Trimethoprim-Sulfamethoxazole	Rwanda	73.47 (48.95 - 103.73)	1.12 (0.75 - 1.58)
Klebsiella pneumoniae	Trimethoprim-Sulfamethoxazole	Rwanda	67.37 (32.84 - 112.84)	0.95 (0.47 - 1.6)
Klebsiella pneumoniae	Fluoroquinolones	Rwanda	67.2 (39.21 - 103.48)	0.95 (0.55 - 1.45)
Staphylococcus aureus	Methicillin	Sao Tome and Principe	3.68 (1.57 - 6.4)	3.78 (1.64 - 6.52)
Klebsiella pneumoniae	Third-generation cephalosporins	Sao Tome and Principe	2.69 (0.73 - 5.44)	2.59 (0.72 - 5.26)
Streptococcus pneumoniae	Trimethoprim-Sulfamethoxazole	Sao Tome and Principe	1.82 (0.24 - 3.6)	1.59 (0.2 - 3.08)
Staphylococcus aureus	Trimethoprim-Sulfamethoxazole	Sao Tome and Principe	1.72 (0.88 - 2.7)	1.77 (0.91 - 2.73)
Streptococcus pneumoniae	Carbapenems	Sao Tome and Principe	1.36 (0.59 - 2.43)	1.19 (0.52 - 2.12)
Acinetobacter baumannii	Carbapenems	Sao Tome and Principe	1.18 (0.59 - 2.14)	1.29 (0.64 - 2.34)
Escherichia coli	Trimethoprim-Sulfamethoxazole	Sao Tome and Principe	1.13 (0.73 - 1.63)	1.14 (0.75 - 1.62)
Klebsiella pneumoniae	Aminoglycosides	Sao Tome and Principe	1.1 (0.65 - 1.69)	1.06 (0.62 - 1.63)
Acinetobacter baumannii	Fluoroquinolones	Sao Tome and Principe	1.09 (0.54 - 1.9)	1.2 (0.58 - 2.09)
Klebsiella pneumoniae	Fluoroquinolones	Sao Tome and Principe	1.07 (0.62 - 1.69)	1.03 (0.61 - 1.57)
Streptococcus pneumoniae	Trimethoprim-Sulfamethoxazole	Senegal	167.56 (22.63 - 338.34)	1.53 (0.21 - 3.06)
Staphylococcus aureus	Methicillin	Senegal	153.85 (63.46 - 272.91)	1.76 (0.74 - 3.08)
Staphylococcus aureus	Trimethoprim-Sulfamethoxazole	Senegal	143.98 (74.46 - 224.1)	1.65 (0.85 - 2.59)
Klebsiella pneumoniae	Third-generation cephalosporins	Senegal	119.53 (25.92 - 304.78)	1.2 (0.26 - 3.08)
Klebsiella pneumoniae	Aminoglycosides	Senegal	114.05 (66.08 - 178.63)	1.15 (0.66 - 1.85)
Acinetobacter baumannii	Anti-pseudomonal penicillin/Beta-Lactamase inhibitors	Senegal	113.06 (54.0 - 195.53)	1.59 (0.74 - 2.78)
Escherichia coli	Trimethoprim-Sulfamethoxazole	Senegal	106.3 (70.26 - 153.21)	1.15 (0.77 - 1.64)
Klebsiella pneumoniae	Fluoroquinolones	Senegal	102.99 (61.26 - 160.66)	1.04 (0.61 - 1.61)
Escherichia coli	Third-generation cephalosporins	Senegal	95.21 (37.47 - 182.34)	1.03 (0.4 - 2.01)
Escherichia coli	Fluoroquinolones	Senegal	94.88 (61.37 - 136.97)	1.03 (0.67 - 1.46)
Staphylococcus aureus	Methicillin	Seychelles	2.88 (1.31 - 4.71)	2.96 (1.35 - 4.86)
Acinetobacter baumannii	Carbapenems	Seychelles	1.05 (0.52 - 1.85)	1.08 (0.54 - 1.89)
Klebsiella pneumoniae	Third-generation cephalosporins	Seychelles	0.96 (0.16 - 2.37)	0.99 (0.16 - 2.43)
Streptococcus pneumoniae	Carbapenems	Seychelles	0.94 (0.42 - 1.6)	0.97 (0.44 - 1.66)
Streptococcus pneumoniae	Trimethoprim-Sulfamethoxazole	Seychelles	0.84 (0.12 - 1.71)	0.87 (0.12 - 1.78)
Acinetobacter baumannii	Fluoroquinolones	Seychelles	0.83 (0.4 - 1.44)	0.85 (0.41 - 1.48)
Escherichia coli	Third-generation cephalosporins	Seychelles	0.69 (0.28 - 1.33)	0.7 (0.29 - 1.36)
Escherichia coli	Fluoroquinolones	Seychelles	0.68 (0.46 - 0.98)	0.7 (0.48 - 1.0)
Enterococcus faecium	Fluoroquinolones	Seychelles	0.63 (0.19 - 1.22)	0.61 (0.18 - 1.16)
Pseudomonas aeruginosa	Carbapenems	Seychelles	0.63 (0.41 - 0.93)	0.65 (0.42 - 0.95)
Streptococcus pneumoniae	Trimethoprim-Sulfamethoxazole	Sierra Leone	198.75 (23.35 - 418.64)	2.66 (0.34 - 5.4)
Staphylococcus aureus	Methicillin	Sierra Leone	169.95 (71.52 - 297.18)	3.21 (1.36 - 5.62)
Klebsiella pneumoniae	Third-generation cephalosporins	Sierra Leone	148.77 (39.59 - 329.7)	2.39 (0.64 - 5.21)
Streptococcus pneumoniae	Carbapenems	Sierra Leone	108.72 (46.81 - 194.8)	1.46 (0.64 - 2.61)
Staphylococcus aureus	Trimethoprim-Sulfamethoxazole	Sierra Leone	105.76 (54.27 - 166.89)	2.0 (1.06 - 3.09)
Klebsiella pneumoniae	Aminoglycosides	Sierra Leone	93.81 (54.65 - 147.58)	1.51 (0.88 - 2.38)
Klebsiella pneumoniae	Fluoroquinolones	Sierra Leone	82.75 (47.8 - 133.7)	1.33 (0.78 - 2.12)
Klebsiella pneumoniae	Trimethoprim-Sulfamethoxazole	Sierra Leone	77.01 (35.93 - 130.18)	1.24 (0.59 - 2.11)
Escherichia coli	Trimethoprim-Sulfamethoxazole	Sierra Leone	68.93 (46.18 - 100.13)	1.25 (0.84 - 1.78)

Acinetobacter baumannii	Anti-pseudomonal penicillin/Beta-Lactamase inhibitors	Sierra Leone	64.92 (32.24 - 111.51)	1.61 (0.78 - 2.79)
Staphylococcus aureus	Methicillin	South Africa	929.05 (411.18 - 1523.23)	2.25 (1.0 - 3.67)
Klebsiella pneumoniae	Third-generation cephalosporins	South Africa	924.41 (359.02 - 1644.11)	2.2 (0.84 - 3.91)
Streptococcus pneumoniae	Carbapenems	South Africa	526.49 (254.29 - 862.52)	1.17 (0.57 - 1.92)
Streptococcus pneumoniae	Trimethoprim-Sulfamethoxazole	South Africa	406.35 (59.24 - 811.52)	0.91 (0.13 - 1.81)
Acinetobacter baumannii	Carbapenems	South Africa	394.35 (201.42 - 672.34)	0.97 (0.49 - 1.66)
Acinetobacter baumannii	Fluoroquinolones	South Africa	388.76 (192.86 - 631.61)	0.96 (0.47 - 1.58)
Klebsiella pneumoniae	Trimethoprim-Sulfamethoxazole	South Africa	363.33 (185.14 - 586.79)	0.86 (0.44 - 1.4)
Pseudomonas aeruginosa	Carbapenems	South Africa	357.48 (232.99 - 513.77)	0.86 (0.56 - 1.24)
Escherichia coli	Trimethoprim-Sulfamethoxazole	South Africa	334.88 (227.1 - 467.63)	0.82 (0.55 - 1.15)
Klebsiella pneumoniae	Carbapenems	South Africa	332.49 (220.93 - 479.31)	0.79 (0.53 - 1.15)
Streptococcus pneumoniae	Trimethoprim-Sulfamethoxazole	South Sudan	246.05 (33.7 - 519.34)	2.31 (0.32 - 4.82)
Streptococcus pneumoniae	Fluoroquinolones	South Sudan	203.74 (44.05 - 422.68)	1.92 (0.44 - 3.86)
Streptococcus pneumoniae	Carbapenems	South Sudan	160.33 (71.48 - 286.64)	1.51 (0.67 - 2.67)
Klebsiella pneumoniae	Third-generation cephalosporins	South Sudan	153.89 (37.49 - 345.26)	1.98 (0.48 - 4.39)
Acinetobacter baumannii	Anti-pseudomonal penicillin/Beta-Lactamase inhibitors	South Sudan	97.46 (50.33 - 164.91)	2.02 (0.99 - 3.46)
Klebsiella pneumoniae	Aminoglycosides	South Sudan	97.38 (57.55 - 149.97)	1.25 (0.73 - 1.96)
Staphylococcus aureus	Trimethoprim-Sulfamethoxazole	South Sudan	80.15 (40.72 - 127.08)	1.22 (0.62 - 1.97)
Group B Streptococcus	Fluoroquinolones	South Sudan	75.25 (12.07 - 168.03)	0.69 (0.11 - 1.51)
Escherichia coli	Trimethoprim-Sulfamethoxazole	South Sudan	73.98 (50.03 - 103.59)	1.07 (0.71 - 1.5)
Escherichia coli	Third-generation cephalosporins	South Sudan	66.07 (25.9 - 128.22)	0.96 (0.37 - 1.87)
Klebsiella pneumoniae	Third-generation cephalosporins	Eswatini	25.36 (8.77 - 49.94)	3.88 (1.32 - 7.62)
Staphylococcus aureus	Trimethoprim-Sulfamethoxazole	Eswatini	15.1 (7.8 - 24.18)	2.45 (1.25 - 3.98)
Streptococcus pneumoniae	Trimethoprim-Sulfamethoxazole	Eswatini	14.01 (2.03 - 28.12)	1.75 (0.26 - 3.51)
Streptococcus pneumoniae	Carbapenems	Eswatini	10.17 (4.38 - 19.3)	1.27 (0.55 - 2.37)
Klebsiella pneumoniae	Trimethoprim-Sulfamethoxazole	Eswatini	8.93 (4.46 - 14.81)	1.37 (0.66 - 2.32)
Escherichia coli	Third-generation cephalosporins	Eswatini	8.66 (3.21 - 17.12)	1.44 (0.54 - 2.85)
Acinetobacter baumannii	Anti-pseudomonal penicillin/Beta-Lactamase inhibitors	Eswatini	8.3 (3.9 - 15.2)	1.61 (0.75 - 2.92)
Acinetobacter baumannii	Fluoroquinolones	Eswatini	8.11 (3.81 - 14.72)	1.57 (0.73 - 2.85)
Acinetobacter baumannii	Carbapenems	Eswatini	8.02 (3.72 - 15.66)	1.55 (0.72 - 3.03)
Staphylococcus aureus	Methicillin	Eswatini	7.94 (3.0 - 14.98)	1.29 (0.49 - 2.41)
Staphylococcus aureus	Methicillin	Tanzania	889.9 (365.28 - 1563.84)	2.67 (1.09 - 4.66)
Streptococcus pneumoniae	Trimethoprim-Sulfamethoxazole	Tanzania	829.18 (113.99 - 1640.26)	1.75 (0.25 - 3.37)
Klebsiella pneumoniae	Third-generation cephalosporins	Tanzania	815.86 (215.22 - 1780.64)	2.09 (0.58 - 4.47)
Staphylococcus aureus	Trimethoprim-Sulfamethoxazole	Tanzania	610.36 (317.93 - 941.35)	1.83 (0.93 - 2.84)
Streptococcus pneumoniae	Carbapenems	Tanzania	497.65 (208.08 - 879.87)	1.05 (0.45 - 1.83)
Klebsiella pneumoniae	Aminoglycosides	Tanzania	471.67 (282.22 - 733.17)	1.21 (0.71 - 1.93)
Escherichia coli	Third-generation cephalosporins	Tanzania	452.32 (186.38 - 878.81)	1.37 (0.56 - 2.66)
Klebsiella pneumoniae	Fluoroquinolones	Tanzania	415.64 (244.29 - 656.17)	1.06 (0.64 - 1.66)
Klebsiella pneumoniae	Trimethoprim-Sulfamethoxazole	Tanzania	385.62 (186.41 - 636.81)	0.99 (0.48 - 1.63)
Escherichia coli	Trimethoprim-Sulfamethoxazole	Tanzania	381.48 (259.68 - 529.99)	1.15 (0.79 - 1.58)
Streptococcus pneumoniae	Trimethoprim-Sulfamethoxazole	The Gambia	36.67 (4.93 - 71.56)	2.73 (0.39 - 5.33)
Staphylococcus aureus	Trimethoprim-Sulfamethoxazole	The Gambia	22.19 (11.91 - 33.54)	2.06 (1.1 - 3.08)
Klebsiella pneumoniae	Third-generation cephalosporins	The Gambia	21.33 (4.63 - 54.39)	1.72 (0.36 - 4.26)
Acinetobacter baumannii	Anti-pseudomonal penicillin/Beta-Lactamase inhibitors	The Gambia	18.05 (8.99 - 30.38)	1.91 (0.92 - 3.24)
Klebsiella pneumoniae	Beta Lactam/Beta-lactamase inhibitors	The Gambia	17.27 (7.22 - 29.8)	1.39 (0.59 - 2.42)

Klebsiella pneumoniae	Aminoglycosides	The Gambia	16.89 (9.76 - 26.51)	1.36 (0.79 - 2.18)
Escherichia coli	Trimethoprim-Sulfamethoxazole	The Gambia	15.64 (10.62 - 22.1)	1.36 (0.93 - 1.9)
Streptococcus pneumoniae	Carbapenems	The Gambia	14.81 (6.26 - 27.4)	1.1 (0.47 - 2.02)
Escherichia coli	Beta Lactam/Beta-lactamase inhibitors	The Gambia	13.76 (9.21 - 19.99)	1.2 (0.8 - 1.74)
Staphylococcus aureus	Methicillin	The Gambia	12.34 (4.97 - 22.6)	1.15 (0.47 - 2.09)
Klebsiella pneumoniae	Third-generation cephalosporins	Togo	128.14 (38.05 - 255.26)	2.82 (0.85 - 5.63)
Staphylococcus aureus	Methicillin	Togo	124.05 (52.88 - 218.46)	3.14 (1.37 - 5.46)
Streptococcus pneumoniae	Trimethoprim-Sulfamethoxazole	Togo	93.2 (12.16 - 190.13)	1.83 (0.25 - 3.66)
Staphylococcus aureus	Trimethoprim-Sulfamethoxazole	Togo	74.57 (38.25 - 114.53)	1.89 (0.98 - 2.9)
Klebsiella pneumoniae	Fluoroquinolones	Togo	55.05 (32.58 - 86.67)	1.21 (0.72 - 1.9)
Klebsiella pneumoniae	Aminoglycosides	Togo	53.26 (31.13 - 82.66)	1.17 (0.69 - 1.79)
Escherichia coli	Third-generation cephalosporins	Togo	50.54 (20.26 - 99.95)	1.13 (0.45 - 2.25)
Klebsiella pneumoniae	Trimethoprim-Sulfamethoxazole	Togo	49.66 (23.39 - 84.53)	1.09 (0.52 - 1.86)
Escherichia coli	Trimethoprim-Sulfamethoxazole	Togo	49.13 (32.59 - 71.11)	1.1 (0.75 - 1.56)
Streptococcus pneumoniae	Carbapenems	Togo	48.37 (21.15 - 90.85)	0.95 (0.42 - 1.75)
Klebsiella pneumoniae	Third-generation cephalosporins	Uganda	641.88 (203.86 - 1283.61)	2.55 (0.8 - 5.07)
Streptococcus pneumoniae	Trimethoprim-Sulfamethoxazole	Uganda	461.99 (62.17 - 959.55)	1.59 (0.23 - 3.16)
Staphylococcus aureus	Trimethoprim-Sulfamethoxazole	Uganda	326.16 (167.87 - 511.31)	1.63 (0.83 - 2.57)
Klebsiella pneumoniae	Aminoglycosides	Uganda	290.55 (169.19 - 442.16)	1.15 (0.68 - 1.79)
Streptococcus pneumoniae	Carbapenems	Uganda	289.45 (124.88 - 509.47)	1.0 (0.43 - 1.73)
Escherichia coli	Third-generation cephalosporins	Uganda	277.01 (114.93 - 526.99)	1.29 (0.53 - 2.41)
Escherichia coli	Trimethoprim-Sulfamethoxazole	Uganda	268.57 (179.18 - 378.67)	1.25 (0.86 - 1.75)
Acinetobacter baumannii	Anti-pseudomonal penicillin/Beta-Lactamase inhibitors	Uganda	254.95 (112.55 - 450.23)	1.61 (0.7 - 2.88)
Klebsiella pneumoniae	Fluoroquinolones	Uganda	251.04 (147.08 - 387.0)	1.0 (0.59 - 1.52)
Klebsiella pneumoniae	Trimethoprim-Sulfamethoxazole	Uganda	225.54 (107.18 - 376.9)	0.89 (0.43 - 1.48)
Klebsiella pneumoniae	Third-generation cephalosporins	Zambia	274.77 (88.54 - 560.19)	2.82 (0.89 - 5.75)
Staphylococcus aureus	Trimethoprim-Sulfamethoxazole	Zambia	269.6 (141.93 - 414.81)	3.25 (1.7 - 5.04)
Streptococcus pneumoniae	Trimethoprim-Sulfamethoxazole	Zambia	220.63 (32.62 - 438.39)	1.85 (0.27 - 3.63)
Klebsiella pneumoniae	Aminoglycosides	Zambia	177.51 (108.74 - 267.26)	1.82 (1.13 - 2.8)
Escherichia coli	Carbapenems	Zambia	173.9 (87.62 - 293.85)	1.96 (1.0 - 3.31)
Staphylococcus aureus	Methicillin	Zambia	147.06 (57.93 - 268.88)	1.77 (0.7 - 3.31)
Streptococcus pneumoniae	Carbapenems	Zambia	127.21 (55.46 - 232.12)	1.07 (0.48 - 1.86)
Escherichia coli	Trimethoprim-Sulfamethoxazole	Zambia	125.84 (84.19 - 179.55)	1.42 (0.96 - 2.02)
Klebsiella pneumoniae	Trimethoprim-Sulfamethoxazole	Zambia	111.59 (54.68 - 185.18)	1.15 (0.55 - 1.95)
Klebsiella pneumoniae	Fluoroquinolones	Zambia	110.52 (64.65 - 173.98)	1.14 (0.67 - 1.78)
Klebsiella pneumoniae	Third-generation cephalosporins	Zimbabwe	386.99 (133.64 - 737.07)	4.35 (1.46 - 8.37)
Streptococcus pneumoniae	Trimethoprim-Sulfamethoxazole	Zimbabwe	235.36 (33.73 - 472.22)	2.25 (0.33 - 4.44)
Staphylococcus aureus	Trimethoprim-Sulfamethoxazole	Zimbabwe	180.05 (94.49 - 278.58)	2.26 (1.16 - 3.48)
Streptococcus pneumoniae	Carbapenems	Zimbabwe	176.39 (78.56 - 312.26)	1.68 (0.74 - 2.96)
Escherichia coli	Third-generation cephalosporins	Zimbabwe	149.76 (59.5 - 284.13)	1.81 (0.71 - 3.51)
Klebsiella pneumoniae	Trimethoprim-Sulfamethoxazole	Zimbabwe	131.45 (63.92 - 220.11)	1.48 (0.73 - 2.48)
Staphylococcus aureus	Methicillin	Zimbabwe	121.76 (45.7 - 219.8)	1.52 (0.57 - 2.76)
Escherichia coli	Trimethoprim-Sulfamethoxazole	Zimbabwe	118.41 (81.64 - 166.77)	1.43 (0.98 - 2.01)
Acinetobacter baumannii	Anti-pseudomonal penicillin/Beta-Lactamase inhibitors	Zimbabwe	104.45 (48.77 - 187.82)	1.63 (0.74 - 2.95)
Klebsiella pneumoniae	Fluoroquinolones	Zimbabwe	94.67 (55.7 - 146.38)	1.06 (0.63 - 1.67)

Supplementary Table 4. Mortality associated with antimicrobial resistance (expressed as counts and age-standardised rates (ASMR) per 100 000 with 95% uncertainty intervals) per country in the WHO African region and top 10 pathogen–drug combination.

Pathogen	Antibiotic class	Country	Deaths associated with AMR (counts)	Deaths associated with AMR (age-standardised rate
Staphylococcus	Macrolide	Algeria	2395.64 (1612.3 - 3534.11)	8.87 (6.12 - 12.93)
Escherichia coli	Aminopenicillin	Algeria	2226.08 (1387.91 - 3398.88)	8.17 (5.19 - 12.28)
Klebsiella	Beta Lactam/Beta-lactamase inhibitors	Algeria	1977.59 (1252.27 - 2974.9)	7.03 (4.47 - 10.54)
Klebsiella	Third-generation cephalosporins	Algeria	1900.1 (978.0 - 3126.45)	6.76 (3.48 - 11.13)
Staphylococcus	Methicillin	Algeria	1797.95 (1195.83 - 2678.08)	6.66 (4.54 - 9.71)
Escherichia coli	Trimethoprim-Sulfamethoxazole	Algeria	1669.8 (1035.53 - 2557.25)	6.13 (3.83 - 9.43)
Klebsiella	Trimethoprim-Sulfamethoxazole	Algeria	1486.95 (921.83 - 2342.9)	5.29 (3.35 - 8.29)
Escherichia coli	Beta Lactam/Beta-lactamase inhibitors	Algeria	1413.47 (886.76 - 2197.68)	5.19 (3.28 - 8.03)
Streptococcus	Trimethoprim-Sulfamethoxazole	Algeria	1190.81 (835.37 - 1681.43)	4.06 (2.9 - 5.67)
Streptococcus	Macrolide	Algeria	1130.6 (786.16 - 1584.75)	3.85 (2.73 - 5.33)
Streptococcus	Trimethoprim-Sulfamethoxazole	Angola	4561.21 (3252.18 - 6313.36)	20.18 (15.46 - 25.71)
Klebsiella	Trimethoprim-Sulfamethoxazole	Angola	3509.52 (2554.91 - 4728.52)	20.2 (14.88 - 27.53)
Klebsiella	Beta Lactam/Beta-lactamase inhibitors	Angola	3465.26 (2519.79 - 4610.99)	19.95 (14.5 - 26.99)
Klebsiella	Third-generation cephalosporins	Angola	3000.97 (1424.98 - 4445.88)	17.28 (8.17 - 25.95)
Escherichia coli	Trimethoprim-Sulfamethoxazole	Angola	2728.3 (2006.04 - 3673.57)	17.33 (12.74 - 23.41)
Escherichia coli	Aminopenicillin	Angola	2682.52 (1979.59 - 3626.52)	17.04 (12.51 - 22.96)
Klebsiella	Aminoglycosides	Angola	2667.36 (1830.45 - 3736.27)	15.35 (10.53 - 21.8)
Klebsiella	Fluoroquinolones	Angola	2657.97 (1874.17 - 3639.84)	15.3 (10.99 - 21.37)
Escherichia coli	Beta Lactam/Beta-lactamase inhibitors	Angola	2217.67 (1599.43 - 3010.08)	14.09 (10.34 - 19.08)
Streptococcus	Beta Lactam/Beta-lactamase inhibitors	Angola	1860.27 (1248.74 - 2689.17)	8.23 (5.85 - 11.37)
Klebsiella	Trimethoprim-Sulfamethoxazole	Benin	2554.23 (1837.14 - 3480.46)	26.96 (19.77 - 36.19)
Streptococcus	Trimethoprim-Sulfamethoxazole	Benin	2527.44 (1774.26 - 3472.09)	22.1 (16.4 - 29.05)
Klebsiella	Beta Lactam/Beta-lactamase inhibitors	Benin	2502.62 (1801.65 - 3390.05)	26.41 (19.26 - 35.34)
Klebsiella	Third-generation cephalosporins	Benin	2176.28 (1198.66 - 3179.5)	22.97 (12.8 - 33.35)
Klebsiella	Fluoroquinolones	Benin	2001.2 (1417.05 - 2751.71)	21.12 (15.04 - 28.88)
Klebsiella	Aminoglycosides	Benin	1916.62 (1299.68 - 2727.47)	20.23 (13.73 - 28.11)
Escherichia coli	Aminopenicillin	Benin	1817.49 (1294.07 - 2478.64)	21.39 (15.52 - 28.98)
Escherichia coli	Trimethoprim-Sulfamethoxazole	Benin	1803.27 (1278.27 - 2472.49)	21.22 (15.26 - 28.75)
Escherichia coli	Beta Lactam/Beta-lactamase inhibitors	Benin	1560.9 (1109.42 - 2141.52)	18.37 (13.09 - 25.01)
Staphylococcus	Trimethoprim-Sulfamethoxazole	Benin	1374.34 (988.53 - 1857.09)	17.5 (13.22 - 22.94)
Klebsiella	Beta Lactam/Beta-lactamase inhibitors	Botswana	270.06 (191.98 - 379.8)	19.41 (13.89 - 27.4)
Escherichia coli	Aminopenicillin	Botswana	265.08 (183.21 - 381.31)	20.18 (14.0 - 28.87)
Klebsiella	Trimethoprim-Sulfamethoxazole	Botswana	264.79 (185.91 - 370.7)	19.04 (13.55 - 26.86)
Streptococcus	Trimethoprim-Sulfamethoxazole	Botswana	257.87 (181.99 - 354.48)	16.15 (11.66 - 21.89)
Klebsiella	Third-generation cephalosporins	Botswana	238.06 (118.44 - 371.8)	17.11 (8.4 - 26.84)
Escherichia coli	Trimethoprim-Sulfamethoxazole	Botswana	232.36 (161.74 - 334.61)	17.69 (12.26 - 25.34)
Escherichia coli	Beta Lactam/Beta-lactamase inhibitors	Botswana	151.5 (102.5 - 214.91)	11.53 (7.88 - 16.51)
Streptococcus	Penicillin	Botswana	145.65 (86.12 - 229.68)	9.12 (5.5 - 14.36)
Staphylococcus	Trimethoprim-Sulfamethoxazole	Botswana	142.39 (102.95 - 194.24)	10.73 (7.86 - 14.46)
Staphylococcus	Fluoroquinolones	Botswana	125.91 (80.84 - 184.62)	9.49 (6.16 - 13.82)
Klebsiella	Trimethoprim-Sulfamethoxazole	Burkina Faso	5934.75 (4520.09 - 7733.81)	31.53 (24.08 - 40.79)
Klebsiella	Beta Lactam/Beta-lactamase inhibitors	Burkina Faso	5605.51 (4198.22 - 7396.45)	29.77 (22.82 - 38.84)
Streptococcus	Trimethoprim-Sulfamethoxazole	Burkina Faso	5516.89 (3974.66 - 7359.08)	23.84 (18.45 - 30.0)
Klebsiella	Third-generation cephalosporins	Burkina Faso	5325.7 (3282.19 - 7496.89)	28.3 (17.4 - 39.24)
Klebsiella	Fluoroquinolones	Burkina Faso	4568.11 (3348.64 - 6118.71)	24.26 (18.34 - 32.0)
Escherichia coli	Aminopenicillin	Burkina Faso	4139.89 (3091.2 - 5464.97)	25.4 (19.11 - 32.87)
Klebsiella	Aminoglycosides	Burkina Faso	4030.78 (2810.72 - 5553.21)	21.42 (15.25 - 29.7)
Escherichia coli	Trimethoprim-Sulfamethoxazole	Burkina Faso	3844.15 (2848.14 - 5071.11)	23.59 (17.73 - 30.54)
Escherichia coli	Beta Lactam/Beta-lactamase inhibitors	Burkina Faso	3395.6 (2541.65 - 4527.71)	20.84 (15.63 - 27.49)
Staphylococcus	Macrolide	Burkina Faso	3293.63 (2481.66 - 4318.61)	21.16 (16.35 - 26.98)

Klebsiella	Beta Lactam/Beta-lactamase inhibitors	Burundi	1854.26 (1316.03 - 2513.49)	26.53 (19.22 - 35.47)
Klebsiella	Trimethoprim-Sulfamethoxazole	Burundi	1772.9 (1253.42 - 2384.42)	25.36 (18.44 - 33.83)
Streptococcus	Trimethoprim-Sulfamethoxazole	Burundi	1698.85 (1224.43 - 2360.75)	20.78 (16.09 - 26.63)
Escherichia coli	Aminopenicillin	Burundi	1595.02 (1123.64 - 2259.05)	23.17 (16.92 - 31.37)
Klebsiella	Aminoglycosides	Burundi	1557.32 (1085.96 - 2148.26)	22.28 (15.87 - 30.67)
Klebsiella	Third-generation cephalosporins	Burundi	1413.83 (591.61 - 2274.58)	20.22 (8.8 - 32.7)
Escherichia coli	Trimethoprim-Sulfamethoxazole	Burundi	1400.7 (986.59 - 1992.63)	20.35 (14.77 - 27.82)
Escherichia coli	Beta Lactam/Beta-lactamase inhibitors	Burundi	1190.06 (832.36 - 1699.58)	17.29 (12.34 - 23.76)
Streptococcus	Fluoroquinolones	Burundi	988.82 (690.04 - 1430.83)	12.1 (9.01 - 16.2)
Staphylococcus	Trimethoprim-Sulfamethoxazole	Burundi	978.13 (733.06 - 1293.96)	16.91 (12.8 - 22.08)
Streptococcus	Trimethoprim-Sulfamethoxazole	Cameroon	4182.35 (2979.27 - 5741.69)	20.14 (15.27 - 26.46)
Klebsiella	Trimethoprim-Sulfamethoxazole	Cameroon	4170.78 (3070.29 - 5520.39)	23.9 (17.5 - 32.0)
Klebsiella	Beta Lactam/Beta-lactamase inhibitors	Cameroon	4078.46 (3006.47 - 5391.62)	23.37 (16.99 - 31.36)
Klebsiella	Fluoroquinolones	Cameroon	3393.37 (2438.43 - 4580.79)	19.45 (13.9 - 26.51)
Escherichia coli	Aminopenicillin	Cameroon	3286.84 (2377.53 - 4413.77)	19.85 (14.27 - 26.79)
Escherichia coli	Trimethoprim-Sulfamethoxazole	Cameroon	3086.8 (2198.99 - 4144.74)	18.64 (13.27 - 25.45)
Klebsiella	Aminoglycosides	Cameroon	2950.61 (1993.33 - 4035.19)	16.91 (11.26 - 23.5)
Escherichia coli	Beta Lactam/Beta-lactamase inhibitors	Cameroon	2715.54 (1942.88 - 3705.45)	16.4 (11.43 - 22.43)
Staphylococcus	Trimethoprim-Sulfamethoxazole	Cameroon	2394.14 (1798.37 - 3155.57)	15.95 (11.92 - 20.86)
Klebsiella	Third-generation cephalosporins	Cameroon	2256.84 (766.33 - 4067.5)	12.94 (4.26 - 23.91)
Acinetobacter	Anti-pseudomonal penicillin/Beta-	Cape Verde	46.47 (28.37 - 71.01)	10.99 (6.71 - 16.86)
Streptococcus	Trimethoprim-Sulfamethoxazole	Cape Verde	43.03 (34.36 - 53.05)	9.68 (7.78 - 11.89)
Acinetobacter	Third-generation cephalosporins	Cape Verde	41.4 (24.79 - 63.5)	9.79 (5.85 - 14.98)
Acinetobacter	Fluoroquinolones	Cape Verde	40.78 (24.26 - 63.07)	9.64 (5.75 - 14.95)
Escherichia coli	Aminopenicillin	Cape Verde	40.39 (28.68 - 55.91)	9.42 (6.68 - 13.03)
Klebsiella	Beta Lactam/Beta-lactamase inhibitors	Cape Verde	39.74 (28.89 - 54.58)	9.22 (6.67 - 12.64)
Acinetobacter	Fourth-generation cephalosporins	Cape Verde	39.68 (23.27 - 60.98)	9.38 (5.49 - 14.49)
Klebsiella	Trimethoprim-Sulfamethoxazole	Cape Verde	38.67 (27.94 - 52.4)	8.97 (6.47 - 12.25)
Escherichia coli	Trimethoprim-Sulfamethoxazole	Cape Verde	37.72 (26.17 - 52.29)	8.8 (6.13 - 12.24)
Acinetobacter	Carbapenems	Cape Verde	36.6 (22.06 - 56.85)	8.65 (5.2 - 13.49)
Streptococcus	Fluoroquinolones	Central African	1891.85 (1347.81 - 2637.04)	38.86 (28.99 - 52.05)
Streptococcus	Trimethoprim-Sulfamethoxazole	Central African	1809.58 (1291.49 - 2542.33)	37.17 (27.89 - 49.51)
Streptococcus	Macrolide	Central African	1522.02 (1073.57 - 2146.16)	31.26 (23.09 - 42.07)
Streptococcus	Penicillin	Central African	1488.67 (1036.32 - 2167.57)	30.58 (21.8 - 42.37)
Streptococcus	Beta Lactam/Beta-lactamase inhibitors	Central African	1487.58 (1055.01 - 2093.54)	30.55 (22.69 - 41.06)
Klebsiella	Aminoglycosides	Central African	1193.82 (847.93 - 1637.77)	31.27 (21.72 - 43.28)
Klebsiella	Trimethoprim-Sulfamethoxazole	Central African	1169.22 (848.59 - 1607.39)	30.63 (21.7 - 42.3)
Klebsiella	Fluoroquinolones	Central African	1160.12 (846.47 - 1569.73)	30.39 (21.63 - 41.5)
Klebsiella	Beta Lactam/Beta-lactamase inhibitors	Central African	1066.18 (761.55 - 1459.41)	27.93 (19.66 - 38.01)
Klebsiella	Third-generation cephalosporins	Central African	1023.62 (435.94 - 1613.38)	26.81 (11.48 - 43.09)
Streptococcus	Trimethoprim-Sulfamethoxazole	Chad	6661.67 (4833.4 - 8764.67)	32.83 (25.4 - 41.59)
Klebsiella	Beta Lactam/Beta-lactamase inhibitors	Chad	3806.06 (2836.32 - 5007.41)	25.89 (19.72 - 34.12)
Klebsiella	Third-generation cephalosporins	Chad	3788.12 (2611.09 - 5056.36)	25.77 (18.12 - 34.74)
Klebsiella	Trimethoprim-Sulfamethoxazole	Chad	3618.96 (2711.15 - 4706.38)	24.62 (18.74 - 32.38)
Escherichia coli	Aminopenicillin	Chad	3300.98 (2466.86 - 4376.74)	23.38 (17.85 - 30.27)
Klebsiella	Aminoglycosides	Chad	2978.92 (2171.22 - 4052.45)	20.27 (14.52 - 27.57)
Escherichia coli	Trimethoprim-Sulfamethoxazole	Chad	2885.53 (2170.68 - 3825.89)	20.44 (15.54 - 26.58)
Escherichia coli	Beta Lactam/Beta-lactamase inhibitors	Chad	2645.29 (1982.47 - 3512.63)	18.74 (14.14 - 24.4)
Klebsiella	Fluoroquinolones	Chad	2642.7 (1924.8 - 3546.85)	17.98 (13.05 - 24.07)
Streptococcus	Fluoroquinolones	Chad	2390.65 (1626.48 - 3350.49)	11.78 (8.48 - 15.88)
Streptococcus	Trimethoprim-Sulfamethoxazole	Comoros	109.16 (83.16 - 137.57)	18.9 (14.81 - 23.4)
Klebsiella	Beta Lactam/Beta-lactamase inhibitors	Comoros	88.93 (65.84 - 118.13)	17.15 (12.67 - 23.12)
Klebsiella	Trimethoprim-Sulfamethoxazole	Comoros	83.44 (61.54 - 110.73)	16.09 (11.86 - 21.46)
Escherichia coli	Aminopenicillin	Comoros	80.28 (60.25 - 104.15)	15.97 (11.98 - 20.96)
Escherichia coli	Trimethoprim-Sulfamethoxazole	Comoros	69.26 (51.61 - 91.31)	13.78 (10.34 - 18.18)
Klebsiella	Aminoglycosides	Comoros	68.38 (48.24 - 94.83)	13.19 (9.32 - 18.37)
Klebsiella	Fluoroquinolones	Comoros	68.06 (50.07 - 92.45)	13.13 (9.7 - 18.08)

Acinetobacter	Anti-pseudomonal penicillin/Beta-	Comoros	66.6 (41.14 - 102.04)	14.74 (9.07 - 22.72)
Klebsiella	Third-generation cephalosporins	Comoros	65.81 (28.96 - 101.53)	12.69 (5.57 - 19.74)
Escherichia coli	Beta Lactam/Beta-lactamase inhibitors	Comoros	61.45 (44.92 - 81.68)	12.22 (8.95 - 16.19)
Klebsiella	Beta Lactam/Beta-lactamase inhibitors	Congo	574.05 (406.29 - 792.45)	21.22 (15.24 - 29.39)
Klebsiella	Trimethoprim-Sulfamethoxazole	Congo	570.64 (407.38 - 783.21)	21.09 (15.12 - 29.03)
Streptococcus	Trimethoprim-Sulfamethoxazole	Congo	529.26 (392.86 - 705.16)	17.38 (13.27 - 22.42)
Klebsiella	Third-generation cephalosporins	Congo	510.15 (260.41 - 773.34)	18.86 (9.75 - 28.85)
Klebsiella	Fluoroquinolones	Congo	495.55 (355.61 - 690.37)	18.32 (13.19 - 25.66)
Escherichia coli	Aminopenicillin	Congo	468.74 (337.1 - 644.09)	17.97 (12.99 - 24.68)
Escherichia coli	Trimethoprim-Sulfamethoxazole	Congo	451.5 (323.15 - 616.12)	17.31 (12.58 - 23.57)
Klebsiella	Aminoglycosides	Congo	447.77 (305.97 - 636.18)	16.55 (11.37 - 23.52)
Escherichia coli	Beta Lactam/Beta-lactamase inhibitors	Congo	377.66 (265.94 - 522.73)	14.48 (10.25 - 19.91)
Staphylococcus	Trimethoprim-Sulfamethoxazole	Congo	323.15 (234.13 - 442.94)	13.1 (9.74 - 17.53)
Klebsiella	Trimethoprim-Sulfamethoxazole	Cote d'Ivoire	4421.02 (3250.48 - 5824.08)	25.65 (19.17 - 34.07)
Klebsiella	Beta Lactam/Beta-lactamase inhibitors	Cote d'Ivoire	4322.95 (3198.27 - 5689.84)	25.08 (18.86 - 33.7)
Streptococcus	Trimethoprim-Sulfamethoxazole	Cote d'Ivoire	4308.28 (3099.55 - 5688.17)	21.57 (16.56 - 27.55)
Klebsiella	Third-generation cephalosporins	Cote d'Ivoire	3923.94 (2238.24 - 5576.23)	22.76 (13.03 - 32.16)
Klebsiella	Fluoroquinolones	Cote d'Ivoire	3455.19 (2529.36 - 4584.78)	20.04 (14.92 - 26.61)
Klebsiella	Aminoglycosides	Cote d'Ivoire	3342.04 (2329.3 - 4530.25)	19.39 (13.73 - 26.39)
Escherichia coli	Aminopenicillin	Cote d'Ivoire	3256.77 (2336.32 - 4421.06)	20.54 (15.09 - 27.32)
Escherichia coli	Trimethoprim-Sulfamethoxazole	Cote d'Ivoire	3118.27 (2246.28 - 4213.59)	19.67 (14.46 - 26.15)
Escherichia coli	Beta Lactam/Beta-lactamase inhibitors	Cote d'Ivoire	2785.56 (1998.82 - 3775.51)	17.57 (12.88 - 23.46)
Staphylococcus	Trimethoprim-Sulfamethoxazole	Cote d'Ivoire	2501.03 (1859.55 - 3281.49)	17.31 (13.32 - 22.34)
Klebsiella	Aminoglycosides	Democratic Republic	8850.86 (6204.75 - 12146.98)	18.28 (12.5 - 25.38)
Escherichia coli	Aminopenicillin	Democratic Republic	8595.44 (6328.76 - 11455.85)	18.67 (13.55 - 25.06)
Klebsiella	Fluoroquinolones	Democratic Republic	8231.97 (6003.79 - 10896.99)	17.0 (12.24 - 23.08)
Escherichia coli	Trimethoprim-Sulfamethoxazole	Democratic Republic	8151.08 (6063.26 - 10932.4)	17.7 (12.91 - 24.16)
Streptococcus	Fluoroquinolones	Democratic Republic	7952.96 (5640.62 - 10603.91)	14.32 (10.43 - 19.12)
Escherichia coli	Beta Lactam/Beta-lactamase inhibitors	Democratic Republic	7043.06 (5097.57 - 9505.12)	15.29 (10.94 - 20.97)
Staphylococcus	Trimethoprim-Sulfamethoxazole	Democratic Republic	5993.25 (4479.02 - 7879.37)	14.42 (10.61 - 19.17)
Escherichia coli	Fluoroquinolones	Democratic Republic	5684.3 (4094.79 - 7758.75)	12.35 (8.79 - 17.14)
Escherichia coli	Third-generation cephalosporins	Democratic Republic	4971.15 (3399.86 - 6950.15)	10.8 (7.35 - 15.33)
Streptococcus	Beta Lactam/Beta-lactamase inhibitors	Democratic Republic	4553.43 (3002.04 - 6461.78)	8.2 (5.48 - 11.53)
Streptococcus	Trimethoprim-Sulfamethoxazole	Equatorial Guinea	104.54 (66.81 - 155.89)	14.16 (10.02 - 19.66)
Klebsiella	Beta Lactam/Beta-lactamase inhibitors	Equatorial Guinea	83.57 (56.02 - 122.01)	13.15 (8.89 - 18.81)
Klebsiella	Trimethoprim-Sulfamethoxazole	Equatorial Guinea	77.85 (51.74 - 114.04)	12.25 (8.28 - 17.63)
Klebsiella	Fluoroquinolones	Equatorial Guinea	68.29 (45.31 - 102.05)	10.75 (7.29 - 15.58)
Escherichia coli	Aminopenicillin	Equatorial Guinea	67.97 (44.25 - 101.24)	12.26 (8.35 - 17.34)
Escherichia coli	Trimethoprim-Sulfamethoxazole	Equatorial Guinea	62.26 (40.82 - 92.24)	11.23 (7.61 - 15.96)
Klebsiella	Third-generation cephalosporins	Equatorial Guinea	60.82 (22.84 - 109.28)	9.56 (3.62 - 16.72)
Acinetobacter	Anti-pseudomonal penicillin/Beta-	Equatorial Guinea	60.06 (37.29 - 93.05)	10.72 (6.53 - 16.7)
Klebsiella	Aminoglycosides	Equatorial Guinea	57.33 (35.9 - 87.18)	9.02 (5.73 - 13.58)
Acinetobacter	Third-generation cephalosporins	Equatorial Guinea	56.84 (35.34 - 89.31)	10.14 (6.16 - 15.94)
Klebsiella	Beta Lactam/Beta-lactamase inhibitors	Eritrea	1042.52 (722.5 - 1470.89)	32.0 (22.31 - 45.45)
Streptococcus	Trimethoprim-Sulfamethoxazole	Eritrea	1014.03 (688.22 - 1476.42)	25.54 (17.51 - 37.8)
Klebsiella	Trimethoprim-Sulfamethoxazole	Eritrea	986.1 (683.93 - 1383.97)	30.27 (21.07 - 43.02)
Escherichia coli	Aminopenicillin	Eritrea	855.73 (606.25 - 1206.1)	27.15 (19.65 - 37.51)
Klebsiella	Aminoglycosides	Eritrea	834.96 (550.66 - 1191.1)	25.62 (17.24 - 36.28)
Klebsiella	Third-generation cephalosporins	Eritrea	771.89 (329.11 - 1264.5)	23.7 (10.04 - 38.31)
Escherichia coli	Trimethoprim-Sulfamethoxazole	Eritrea	719.75 (515.04 - 1028.56)	22.83 (16.47 - 31.45)
Klebsiella	Fluoroquinolones	Eritrea	718.18 (479.96 - 1037.38)	22.04 (15.14 - 31.61)
Staphylococcus	Trimethoprim-Sulfamethoxazole	Eritrea	715.51 (487.64 - 1033.28)	25.03 (17.29 - 36.71)
Escherichia coli	Beta Lactam/Beta-lactamase inhibitors	Eritrea	663.17 (464.23 - 942.34)	21.04 (15.0 - 29.12)
Klebsiella	Fluoroquinolones	Ethiopia	8985.85 (6716.41 - 11853.79)	14.21 (10.48 - 18.52)
Escherichia coli	Third-generation cephalosporins	Ethiopia	8544.0 (6277.52 - 11415.55)	14.94 (11.16 - 19.92)
Escherichia coli	Fluoroquinolones	Ethiopia	8276.81 (6238.09 - 10890.57)	14.48 (10.79 - 18.81)
Escherichia coli	Beta Lactam/Beta-lactamase inhibitors	Ethiopia	8237.02 (6176.09 - 10912.6)	14.41 (10.79 - 18.73)

Staphylococcus	Macrolide	Ethiopia	6558.46 (4974.36 - 8637.86)	12.16 (9.26 - 16.02)
Staphylococcus	Trimethoprim-Sulfamethoxazole	Ethiopia	6197.23 (4741.64 - 7853.0)	11.49 (8.92 - 14.61)
Klebsiella	Carbapenems	Ethiopia	5806.74 (3820.26 - 8254.77)	9.18 (5.97 - 13.2)
Staphylococcus	Methicillin	Ethiopia	5375.92 (4054.82 - 7041.54)	9.96 (7.52 - 13.06)
Group B	Macrolide	Ethiopia	4087.91 (2953.8 - 5566.85)	4.91 (3.61 - 6.54)
Acinetobacter	Anti-pseudomonal penicillin/Beta-	Ethiopia	4079.78 (2624.02 - 6045.64)	8.9 (5.34 - 13.67)
Streptococcus	Trimethoprim-Sulfamethoxazole	Gabon	147.38 (109.12 - 198.0)	12.93 (9.82 - 16.88)
Klebsiella	Trimethoprim-Sulfamethoxazole	Gabon	134.43 (96.62 - 186.3)	13.0 (9.22 - 18.1)
Klebsiella	Beta Lactam/Beta-lactamase inhibitors	Gabon	133.25 (95.28 - 184.48)	12.88 (9.26 - 17.89)
Escherichia coli	Aminopenicillin	Gabon	131.72 (93.11 - 183.27)	13.23 (9.43 - 18.17)
Acinetobacter	Anti-pseudomonal penicillin/Beta-	Gabon	124.08 (75.24 - 194.37)	13.54 (8.13 - 21.43)
Klebsiella	Fluoroquinolones	Gabon	116.96 (82.54 - 163.6)	11.31 (7.89 - 15.96)
Acinetobacter	Third-generation cephalosporins	Gabon	116.98 (69.87 - 182.59)	12.76 (7.56 - 19.9)
Escherichia coli	Trimethoprim-Sulfamethoxazole	Gabon	115.38 (81.78 - 160.2)	11.59 (8.25 - 16.03)
Klebsiella	Third-generation cephalosporins	Gabon	112.3 (49.57 - 183.56)	10.86 (4.87 - 17.83)
Acinetobacter	Fluoroquinolones	Gabon	101.68 (60.83 - 160.03)	11.1 (6.54 - 17.53)
Klebsiella	Trimethoprim-Sulfamethoxazole	Ghana	4323.35 (3249.54 - 5681.82)	23.13 (17.78 - 30.35)
Klebsiella	Beta Lactam/Beta-lactamase inhibitors	Ghana	4228.69 (3162.29 - 5621.43)	22.63 (17.37 - 29.77)
Streptococcus	Trimethoprim-Sulfamethoxazole	Ghana	4075.06 (3150.7 - 5151.94)	20.14 (16.61 - 24.49)
Klebsiella	Third-generation cephalosporins	Ghana	3784.48 (2636.35 - 5271.56)	20.25 (14.26 - 27.72)
Klebsiella	Aminoglycosides	Ghana	3595.83 (2691.08 - 4811.68)	19.24 (14.55 - 26.04)
Escherichia coli	Aminopenicillin	Ghana	3410.33 (2514.36 - 4599.12)	19.92 (14.8 - 26.95)
Klebsiella	Fluoroquinolones	Ghana	3250.67 (2428.05 - 4366.86)	17.39 (13.07 - 23.16)
Escherichia coli	Trimethoprim-Sulfamethoxazole	Ghana	3213.36 (2372.59 - 4354.7)	18.77 (13.98 - 25.3)
Escherichia coli	Beta Lactam/Beta-lactamase inhibitors	Ghana	3059.83 (2240.83 - 4123.56)	17.87 (13.33 - 24.04)
Escherichia coli	Fluoroquinolones	Ghana	2576.29 (1904.07 - 3479.39)	15.05 (11.21 - 20.54)
Streptococcus	Trimethoprim-Sulfamethoxazole	Guinea	4352.75 (3052.44 - 5853.2)	33.42 (24.92 - 44.21)
Klebsiella	Beta Lactam/Beta-lactamase inhibitors	Guinea	2565.52 (1851.0 - 3416.89)	25.38 (18.32 - 34.01)
Klebsiella	Trimethoprim-Sulfamethoxazole	Guinea	2514.41 (1815.17 - 3364.64)	24.88 (18.19 - 33.59)
Klebsiella	Third-generation cephalosporins	Guinea	2260.98 (1250.27 - 3283.31)	22.37 (12.41 - 32.17)
Klebsiella	Aminoglycosides	Guinea	1955.93 (1337.68 - 2663.06)	19.35 (13.42 - 26.73)
Escherichia coli	Aminopenicillin	Guinea	1900.54 (1373.03 - 2592.17)	20.63 (15.1 - 27.3)
Klebsiella	Fluoroquinolones	Guinea	1879.67 (1345.03 - 2541.73)	18.6 (13.27 - 25.39)
Escherichia coli	Trimethoprim-Sulfamethoxazole	Guinea	1759.72 (1277.08 - 2369.81)	19.1 (13.91 - 25.21)
Streptococcus	Fluoroquinolones	Guinea	1595.94 (1071.12 - 2276.48)	12.26 (8.35 - 17.0)
Escherichia coli	Beta Lactam/Beta-lactamase inhibitors	Guinea	1532.24 (1101.17 - 2085.13)	16.63 (12.04 - 22.32)
Klebsiella	Trimethoprim-Sulfamethoxazole	Guinea-Bissau	326.52 (240.47 - 433.41)	30.85 (22.83 - 41.44)
Streptococcus	Trimethoprim-Sulfamethoxazole	Guinea-Bissau	295.84 (219.94 - 387.63)	24.98 (19.41 - 31.83)
Klebsiella	Beta Lactam/Beta-lactamase inhibitors	Guinea-Bissau	295.49 (218.46 - 395.46)	27.92 (20.69 - 38.09)
Klebsiella	Third-generation cephalosporins	Guinea-Bissau	266.21 (122.8 - 405.2)	25.15 (11.64 - 38.66)
Escherichia coli	Aminopenicillin	Guinea-Bissau	256.17 (187.78 - 341.35)	25.95 (19.27 - 34.74)
Klebsiella	Fluoroquinolones	Guinea-Bissau	247.53 (178.99 - 334.85)	23.39 (16.87 - 31.79)
Klebsiella	Aminoglycosides	Guinea-Bissau	247.83 (172.88 - 334.17)	23.41 (16.44 - 32.22)
Escherichia coli	Trimethoprim-Sulfamethoxazole	Guinea-Bissau	210.43 (152.84 - 282.94)	21.31 (15.75 - 28.52)
Escherichia coli	Beta Lactam/Beta-lactamase inhibitors	Guinea-Bissau	197.14 (142.05 - 265.72)	19.97 (14.67 - 26.69)
Staphylococcus	Macrolide	Guinea-Bissau	186.86 (137.65 - 245.34)	20.94 (15.63 - 27.55)
Klebsiella	Trimethoprim-Sulfamethoxazole	Kenya	5864.58 (4529.84 - 7559.87)	23.74 (18.28 - 31.1)
Escherichia coli	Aminopenicillin	Kenya	5730.81 (4357.62 - 7481.94)	24.42 (18.31 - 31.96)
Streptococcus	Trimethoprim-Sulfamethoxazole	Kenya	5317.61 (4296.37 - 6565.18)	18.6 (15.25 - 22.47)
Escherichia coli	Trimethoprim-Sulfamethoxazole	Kenya	5177.21 (3962.58 - 6733.64)	22.06 (16.51 - 29.07)
Klebsiella	Beta Lactam/Beta-lactamase inhibitors	Kenya	5092.29 (3896.99 - 6682.11)	20.62 (15.65 - 27.68)
Klebsiella	Third-generation cephalosporins	Kenya	4520.57 (2777.08 - 6513.73)	18.31 (11.18 - 26.77)
Klebsiella	Fluoroquinolones	Kenya	4372.06 (3310.51 - 5728.77)	17.7 (13.33 - 23.49)
Klebsiella	Aminoglycosides	Kenya	4184.89 (3058.32 - 5575.19)	16.94 (12.25 - 23.08)
Staphylococcus	Trimethoprim-Sulfamethoxazole	Kenya	3361.92 (2643.52 - 4245.47)	15.14 (11.99 - 18.97)
Escherichia coli	Beta Lactam/Beta-lactamase inhibitors	Kenya	3085.13 (2245.46 - 4154.43)	13.15 (9.49 - 18.01)
Klebsiella	Third-generation cephalosporins	Lesotho	439.18 (270.51 - 629.72)	33.02 (20.82 - 47.92)

Klebsiella	Trimethoprim-Sulfamethoxazole	Lesotho	387.15 (278.35 - 536.92)	29.11 (20.77 - 41.05)
Klebsiella	Beta Lactam/Beta-lactamase inhibitors	Lesotho	381.7 (268.62 - 532.74)	28.7 (20.06 - 40.47)
Streptococcus	Trimethoprim-Sulfamethoxazole	Lesotho	359.88 (262.22 - 463.14)	23.68 (17.34 - 30.66)
Escherichia coli	Aminopenicillin	Lesotho	322.88 (226.33 - 445.76)	25.52 (17.91 - 35.3)
Staphylococcus	Macrolide	Lesotho	304.71 (217.81 - 411.45)	23.96 (17.13 - 32.38)
Escherichia coli	Trimethoprim-Sulfamethoxazole	Lesotho	274.04 (193.54 - 379.76)	21.66 (15.42 - 29.96)
Klebsiella	Fluoroquinolones	Lesotho	265.97 (184.01 - 378.5)	20.0 (13.7 - 28.57)
Escherichia coli	Beta Lactam/Beta-lactamase inhibitors	Lesotho	230.17 (157.86 - 327.64)	18.19 (12.42 - 26.24)
Acinetobacter	Anti-pseudomonal penicillin/Beta-	Lesotho	209.43 (119.39 - 340.53)	19.45 (10.97 - 31.94)
Klebsiella	Trimethoprim-Sulfamethoxazole	Liberia	621.1 (455.08 - 836.67)	22.62 (16.52 - 30.45)
Klebsiella	Beta Lactam/Beta-lactamase inhibitors	Liberia	620.9 (457.51 - 830.8)	22.61 (16.64 - 30.44)
Klebsiella	Third-generation cephalosporins	Liberia	534.79 (284.66 - 792.44)	19.47 (10.58 - 28.53)
Escherichia coli	Aminopenicillin	Liberia	518.11 (370.68 - 706.79)	20.3 (14.67 - 27.85)
Klebsiella	Fluoroquinolones	Liberia	497.35 (358.26 - 674.48)	18.11 (12.97 - 24.64)
Klebsiella	Aminoglycosides	Liberia	489.59 (333.29 - 668.44)	17.83 (12.35 - 24.81)
Streptococcus	Trimethoprim-Sulfamethoxazole	Liberia	488.83 (356.04 - 656.82)	15.98 (12.27 - 20.55)
Escherichia coli	Trimethoprim-Sulfamethoxazole	Liberia	455.67 (326.98 - 621.66)	17.85 (12.84 - 24.57)
Escherichia coli	Beta Lactam/Beta-lactamase inhibitors	Liberia	431.98 (302.76 - 585.55)	16.92 (12.18 - 23.04)
Staphylococcus	Trimethoprim-Sulfamethoxazole	Liberia	331.52 (246.95 - 443.49)	14.06 (10.64 - 18.55)
Klebsiella	Beta Lactam/Beta-lactamase inhibitors	Madagascar	3728.54 (2731.65 - 4892.73)	24.52 (17.54 - 32.84)
Streptococcus	Trimethoprim-Sulfamethoxazole	Madagascar	3696.84 (2840.22 - 4796.88)	20.36 (15.76 - 25.87)
Escherichia coli	Aminopenicillin	Madagascar	3201.5 (2346.36 - 4261.93)	21.24 (15.36 - 28.39)
Streptococcus	Fluoroquinolones	Madagascar	2938.12 (2177.88 - 3779.55)	16.18 (12.13 - 20.88)
Klebsiella	Third-generation cephalosporins	Madagascar	2830.4 (1869.71 - 3966.19)	18.62 (11.82 - 26.99)
Escherichia coli	Trimethoprim-Sulfamethoxazole	Madagascar	2731.54 (2019.53 - 3627.39)	18.12 (13.14 - 24.22)
Klebsiella	Trimethoprim-Sulfamethoxazole	Madagascar	2725.3 (2020.04 - 3571.99)	17.93 (12.88 - 24.11)
Escherichia coli	Beta Lactam/Beta-lactamase inhibitors	Madagascar	2444.46 (1772.76 - 3295.56)	16.22 (11.71 - 22.02)
Klebsiella	Aminoglycosides	Madagascar	2410.81 (1659.7 - 3338.41)	15.86 (10.79 - 22.68)
Staphylococcus	Trimethoprim-Sulfamethoxazole	Madagascar	2225.93 (1664.05 - 2907.01)	17.06 (12.62 - 22.72)
Streptococcus	Trimethoprim-Sulfamethoxazole	Malawi	2798.41 (2221.29 - 3557.68)	21.97 (18.16 - 26.31)
Klebsiella	Trimethoprim-Sulfamethoxazole	Malawi	2634.78 (1992.6 - 3422.41)	24.75 (18.85 - 32.15)
Klebsiella	Beta Lactam/Beta-lactamase inhibitors	Malawi	2467.43 (1854.96 - 3227.49)	23.18 (17.52 - 30.12)
Klebsiella	Third-generation cephalosporins	Malawi	2462.05 (1516.68 - 3438.29)	23.13 (14.74 - 32.2)
Escherichia coli	Aminopenicillin	Malawi	2233.19 (1695.76 - 2909.28)	23.17 (17.72 - 30.05)
Escherichia coli	Trimethoprim-Sulfamethoxazole	Malawi	2180.72 (1662.86 - 2851.27)	22.63 (17.26 - 29.26)
Klebsiella	Aminoglycosides	Malawi	2071.46 (1506.67 - 2760.74)	19.46 (14.21 - 26.04)
Escherichia coli	Beta Lactam/Beta-lactamase inhibitors	Malawi	2067.92 (1546.34 - 2721.91)	21.46 (16.24 - 27.87)
Staphylococcus	Trimethoprim-Sulfamethoxazole	Malawi	1402.66 (1080.29 - 1798.04)	15.29 (12.06 - 19.26)
Klebsiella	Fluoroquinolones	Malawi	1323.53 (980.58 - 1780.68)	12.44 (9.15 - 16.51)
Klebsiella	Trimethoprim-Sulfamethoxazole	Mali	6202.86 (4486.15 - 8409.36)	29.21 (20.85 - 40.08)
Klebsiella	Beta Lactam/Beta-lactamase inhibitors	Mali	6072.35 (4424.36 - 8285.57)	28.59 (20.34 - 39.28)
Klebsiella	Third-generation cephalosporins	Mali	5836.35 (4047.58 - 7919.46)	27.48 (18.68 - 38.54)
Klebsiella	Fluoroquinolones	Mali	4875.85 (3561.23 - 6682.64)	22.96 (16.44 - 31.47)
Klebsiella	Aminoglycosides	Mali	4693.98 (3203.77 - 6521.61)	22.1 (14.95 - 31.28)
Escherichia coli	Trimethoprim-Sulfamethoxazole	Mali	4187.02 (3012.29 - 5743.15)	23.37 (16.77 - 31.63)
Escherichia coli	Aminopenicillin	Mali	4176.64 (2993.2 - 5725.43)	23.31 (16.78 - 31.62)
Streptococcus	Trimethoprim-Sulfamethoxazole	Mali	3419.29 (2517.99 - 4699.3)	12.93 (9.52 - 17.16)
Escherichia coli	Beta Lactam/Beta-lactamase inhibitors	Mali	3312.14 (2364.07 - 4570.92)	18.49 (13.24 - 25.66)
Escherichia coli	Fluoroquinolones	Mali	2778.52 (1930.88 - 3897.25)	15.51 (10.9 - 21.5)
Klebsiella	Trimethoprim-Sulfamethoxazole	Mauritania	442.58 (310.72 - 606.43)	17.71 (12.6 - 24.02)
Klebsiella	Beta Lactam/Beta-lactamase inhibitors	Mauritania	439.82 (309.16 - 607.31)	17.6 (12.54 - 23.85)
Escherichia coli	Aminopenicillin	Mauritania	403.08 (281.06 - 563.93)	16.85 (12.02 - 23.01)
Klebsiella	Third-generation cephalosporins	Mauritania	380.7 (194.03 - 569.42)	15.23 (7.97 - 22.4)
Escherichia coli	Trimethoprim-Sulfamethoxazole	Mauritania	370.53 (257.74 - 515.28)	15.49 (10.93 - 21.24)
Streptococcus	Trimethoprim-Sulfamethoxazole	Mauritania	368.5 (254.99 - 525.24)	13.95 (10.26 - 18.53)
Klebsiella	Fluoroquinolones	Mauritania	327.82 (228.16 - 457.49)	13.12 (9.24 - 18.19)
Escherichia coli	Beta Lactam/Beta-lactamase inhibitors	Mauritania	318.39 (217.84 - 451.38)	13.31 (9.17 - 18.62)

Klebsiella	Aminoglycosides	Mauritania	271.26 (172.18 - 395.47)	10.85 (7.05 - 15.72)
Staphylococcus	Methicillin	Mauritania	219.09 (151.16 - 305.76)	9.78 (6.97 - 13.19)
Staphylococcus	Methicillin	Mauritius	152.15 (98.55 - 225.75)	9.83 (6.44 - 14.52)
Escherichia coli	Aminopenicillin	Mauritius	134.25 (81.21 - 203.51)	8.58 (5.21 - 12.93)
Escherichia coli	Beta Lactam/Beta-lactamase inhibitors	Mauritius	106.08 (64.15 - 162.05)	6.78 (4.12 - 10.35)
Escherichia coli	Trimethoprim-Sulfamethoxazole	Mauritius	95.81 (58.07 - 148.53)	6.12 (3.72 - 9.44)
Staphylococcus	Macrolide	Mauritius	87.43 (55.35 - 133.43)	5.65 (3.61 - 8.57)
Escherichia coli	Fluoroquinolones	Mauritius	82.83 (49.69 - 127.28)	5.29 (3.19 - 8.11)
Klebsiella	Third-generation cephalosporins	Mauritius	77.49 (28.24 - 143.42)	5.07 (1.86 - 9.35)
Klebsiella	Trimethoprim-Sulfamethoxazole	Mauritius	75.5 (44.97 - 116.74)	4.94 (2.96 - 7.6)
Acinetobacter	Anti-pseudomonal penicillin/Beta-	Mauritius	74.46 (39.77 - 123.6)	4.71 (2.56 - 7.79)
Acinetobacter	Fourth-generation cephalosporins	Mauritius	73.71 (39.39 - 122.84)	4.67 (2.52 - 7.69)
Klebsiella	Trimethoprim-Sulfamethoxazole	Mozambique	5454.95 (4174.4 - 7138.07)	29.2 (21.8 - 38.71)
Klebsiella	Third-generation cephalosporins	Mozambique	5173.75 (3726.73 - 6900.59)	27.69 (19.98 - 38.0)
Klebsiella	Beta Lactam/Beta-lactamase inhibitors	Mozambique	5059.53 (3842.82 - 6609.37)	27.08 (20.05 - 36.2)
Streptococcus	Trimethoprim-Sulfamethoxazole	Mozambique	4532.33 (3432.19 - 5915.9)	20.61 (16.26 - 25.94)
Klebsiella	Aminoglycosides	Mozambique	4085.92 (2983.45 - 5507.59)	21.87 (15.94 - 30.33)
Klebsiella	Fluoroquinolones	Mozambique	3827.92 (2812.03 - 5085.52)	20.49 (15.01 - 27.83)
Staphylococcus	Trimethoprim-Sulfamethoxazole	Mozambique	3534.31 (2698.4 - 4622.32)	23.27 (17.72 - 30.39)
Escherichia coli	Aminopenicillin	Mozambique	3265.66 (2426.71 - 4320.3)	19.87 (14.75 - 26.55)
Escherichia coli	Trimethoprim-Sulfamethoxazole	Mozambique	2788.13 (2084.84 - 3721.98)	16.97 (12.58 - 22.86)
Escherichia coli	Beta Lactam/Beta-lactamase inhibitors	Mozambique	2407.27 (1739.64 - 3246.05)	14.65 (10.6 - 20.06)
Klebsiella	Beta Lactam/Beta-lactamase inhibitors	Namibia	289.19 (203.23 - 397.63)	19.07 (13.66 - 26.17)
Klebsiella	Trimethoprim-Sulfamethoxazole	Namibia	282.58 (197.34 - 391.99)	18.64 (13.21 - 25.6)
Escherichia coli	Aminopenicillin	Namibia	252.68 (175.41 - 353.56)	17.69 (12.43 - 24.47)
Streptococcus	Trimethoprim-Sulfamethoxazole	Namibia	233.38 (165.1 - 319.79)	13.94 (10.07 - 18.72)
Escherichia coli	Trimethoprim-Sulfamethoxazole	Namibia	229.33 (159.53 - 320.96)	16.05 (11.18 - 22.28)
Klebsiella	Third-generation cephalosporins	Namibia	221.89 (94.58 - 368.66)	14.63 (6.25 - 24.01)
Staphylococcus	Trimethoprim-Sulfamethoxazole	Namibia	190.72 (138.96 - 260.24)	13.4 (10.03 - 18.09)
Escherichia coli	Beta Lactam/Beta-lactamase inhibitors	Namibia	154.02 (104.73 - 226.72)	10.78 (7.32 - 15.5)
Staphylococcus	Macrolide	Namibia	147.16 (101.68 - 204.63)	10.34 (7.28 - 14.09)
Klebsiella	Fluoroquinolones	Namibia	138.92 (93.46 - 202.32)	9.16 (6.16 - 13.38)
Streptococcus	Trimethoprim-Sulfamethoxazole	Niger	7244.8 (5126.51 - 9765.15)	27.42 (20.78 - 35.91)
Klebsiella	Beta Lactam/Beta-lactamase inhibitors	Niger	5734.73 (4258.78 - 7624.51)	28.23 (20.73 - 37.24)
Klebsiella	Trimethoprim-Sulfamethoxazole	Niger	5669.29 (4181.5 - 7527.1)	27.91 (20.65 - 36.77)
Klebsiella	Third-generation cephalosporins	Niger	4801.85 (2600.12 - 7105.06)	23.63 (12.68 - 34.92)
Klebsiella	Aminoglycosides	Niger	4469.21 (3205.59 - 6038.45)	22.0 (15.38 - 29.85)
Escherichia coli	Aminopenicillin	Niger	4375.34 (3255.99 - 5943.96)	24.01 (17.74 - 31.78)
Klebsiella	Fluoroquinolones	Niger	4347.23 (3186.99 - 5871.29)	21.4 (15.76 - 28.73)
Escherichia coli	Trimethoprim-Sulfamethoxazole	Niger	3974.09 (2949.39 - 5391.24)	21.81 (16.11 - 28.77)
Escherichia coli	Beta Lactam/Beta-lactamase inhibitors	Niger	3454.25 (2491.16 - 4756.99)	18.96 (13.77 - 25.31)
Streptococcus	Fluoroquinolones	Niger	2396.37 (1546.48 - 3461.41)	9.07 (6.29 - 12.69)
Pseudomonas	Aminoglycosides	Nigeria	9788.55 (7002.21 - 13038.71)	6.24 (4.37 - 8.73)
Streptococcus	Third-generation cephalosporins	Nigeria	9278.91 (6000.47 - 13619.61)	4.17 (2.75 - 5.98)
Streptococcus	Beta Lactam/Beta-lactamase inhibitors	Nigeria	8934.22 (5905.54 - 12903.13)	4.02 (2.69 - 5.68)
Pseudomonas	Fluoroquinolones	Nigeria	8825.29 (6423.05 - 11912.14)	5.63 (3.93 - 7.91)
Shigella spp.	Fluoroquinolones	Nigeria	8182.85 (3273.43 - 15638.96)	3.78 (1.53 - 7.35)
Pseudomonas	Fourth-generation cephalosporins	Nigeria	7986.06 (5697.91 - 10890.21)	5.09 (3.52 - 7.09)
Enterobacter spp.	Trimethoprim-Sulfamethoxazole	Nigeria	7853.66 (5510.19 - 11101.69)	5.16 (3.47 - 7.42)
Pseudomonas	Carbapenems	Nigeria	7674.34 (5551.96 - 10607.83)	4.89 (3.41 - 6.83)
Enterobacter spp.	Fourth-generation cephalosporins	Nigeria	7630.49 (5263.76 - 10741.56)	5.02 (3.34 - 7.22)
Pseudomonas	Anti-pseudomonal penicillin/Beta-	Nigeria	7201.89 (5062.98 - 9783.98)	4.59 (3.12 - 6.46)
Klebsiella	Beta Lactam/Beta-lactamase inhibitors	Rwanda	1745.0 (1307.43 - 2312.26)	24.6 (18.44 - 32.47)
Klebsiella	Third-generation cephalosporins	Rwanda	1679.36 (1208.25 - 2256.87)	23.68 (16.73 - 31.75)
Klebsiella	Trimethoprim-Sulfamethoxazole	Rwanda	1578.88 (1179.17 - 2088.53)	22.26 (16.69 - 29.63)
Streptococcus	Trimethoprim-Sulfamethoxazole	Rwanda	1379.12 (1062.83 - 1776.32)	16.54 (13.2 - 20.59)
Escherichia coli	Aminopenicillin	Rwanda	1332.11 (992.76 - 1784.21)	20.35 (15.08 - 27.15)

Klebsiella	Aminoglycosides	Rwanda	1312.94 (958.18 - 1772.93)	18.51 (13.63 - 24.87)
Escherichia coli	Trimethoprim-Sulfamethoxazole	Rwanda	1187.47 (879.46 - 1565.12)	18.14 (13.42 - 24.24)
Staphylococcus	Trimethoprim-Sulfamethoxazole	Rwanda	1151.31 (890.92 - 1472.77)	18.3 (14.28 - 23.36)
Klebsiella	Fluoroquinolones	Rwanda	1124.25 (829.45 - 1510.41)	15.85 (11.77 - 21.34)
Escherichia coli	Beta Lactam/Beta-lactamase inhibitors	Rwanda	1055.77 (765.08 - 1426.33)	16.13 (11.74 - 21.76)
Klebsiella	Beta Lactam/Beta-lactamase inhibitors	Sao Tome and Principe	23.09 (16.25 - 31.02)	22.16 (16.09 - 29.24)
Klebsiella	Trimethoprim-Sulfamethoxazole	Sao Tome and Principe	22.55 (16.11 - 30.24)	21.64 (15.81 - 28.64)
Klebsiella	Third-generation cephalosporins	Sao Tome and Principe	20.27 (11.04 - 30.01)	19.45 (10.83 - 28.57)
Escherichia coli	Aminopenicillin	Sao Tome and Principe	19.8 (13.91 - 27.46)	19.92 (14.28 - 27.31)
Streptococcus	Trimethoprim-Sulfamethoxazole	Sao Tome and Principe	18.56 (13.85 - 23.72)	16.17 (12.46 - 20.39)
Escherichia coli	Trimethoprim-Sulfamethoxazole	Sao Tome and Principe	18.07 (12.64 - 25.14)	18.19 (12.99 - 24.95)
Klebsiella	Fluoroquinolones	Sao Tome and Principe	17.43 (12.4 - 23.85)	16.72 (12.0 - 22.62)
Staphylococcus	Macrolide	Sao Tome and Principe	16.93 (12.5 - 22.24)	17.38 (13.14 - 22.38)
Staphylococcus	Trimethoprim-Sulfamethoxazole	Sao Tome and Principe	16.82 (12.69 - 21.98)	17.27 (13.33 - 21.88)
Escherichia coli	Beta Lactam/Beta-lactamase inhibitors	Sao Tome and Principe	16.35 (11.16 - 22.78)	16.45 (11.5 - 22.59)
Klebsiella	Trimethoprim-Sulfamethoxazole	Senegal	2229.77 (1620.79 - 2954.99)	22.48 (16.48 - 30.34)
Klebsiella	Beta Lactam/Beta-lactamase inhibitors	Senegal	2227.51 (1636.41 - 2956.41)	22.46 (16.37 - 30.35)
Escherichia coli	Aminopenicillin	Senegal	1797.32 (1305.92 - 2426.55)	19.43 (14.26 - 26.21)
Klebsiella	Fluoroquinolones	Senegal	1780.96 (1296.01 - 2403.76)	17.95 (13.12 - 24.3)
Escherichia coli	Trimethoprim-Sulfamethoxazole	Senegal	1759.17 (1282.55 - 2380.89)	19.02 (13.95 - 25.89)
Klebsiella	Aminoglycosides	Senegal	1695.55 (1167.14 - 2356.34)	17.09 (11.83 - 24.12)
Streptococcus	Trimethoprim-Sulfamethoxazole	Senegal	1660.84 (1215.26 - 2194.95)	15.15 (11.56 - 19.17)
Escherichia coli	Beta Lactam/Beta-lactamase inhibitors	Senegal	1540.0 (1102.65 - 2113.94)	16.65 (11.95 - 22.63)
Staphylococcus	Trimethoprim-Sulfamethoxazole	Senegal	1307.92 (970.11 - 1700.07)	14.97 (11.23 - 19.4)
Staphylococcus	Macrolide	Senegal	1099.95 (796.0 - 1468.98)	12.59 (9.27 - 16.66)
Escherichia coli	Aminopenicillin	Seychelles	13.21 (9.79 - 17.41)	13.57 (10.1 - 17.77)
Staphylococcus	Methicillin	Seychelles	10.67 (8.17 - 13.93)	10.97 (8.45 - 14.18)
Escherichia coli	Beta Lactam/Beta-lactamase inhibitors	Seychelles	9.62 (7.01 - 12.97)	9.87 (7.23 - 13.23)
Streptococcus	Trimethoprim-Sulfamethoxazole	Seychelles	9.27 (7.18 - 11.8)	9.6 (7.45 - 12.23)
Escherichia coli	Trimethoprim-Sulfamethoxazole	Seychelles	8.96 (6.62 - 11.81)	9.2 (6.81 - 12.07)
Acinetobacter	Fourth-generation cephalosporins	Seychelles	7.86 (4.74 - 12.12)	8.08 (4.9 - 12.38)
Staphylococcus	Macrolide	Seychelles	7.85 (5.82 - 10.61)	8.08 (6.02 - 10.8)
Acinetobacter	Anti-pseudomonal penicillin/Beta-	Seychelles	7.5 (4.39 - 11.6)	7.71 (4.54 - 11.98)
Streptococcus	Macrolide	Seychelles	6.93 (5.2 - 8.92)	7.18 (5.41 - 9.27)
Klebsiella	Beta Lactam/Beta-lactamase inhibitors	Seychelles	6.84 (4.94 - 9.42)	7.05 (5.12 - 9.66)
Streptococcus	Trimethoprim-Sulfamethoxazole	Sierra Leone	1946.17 (1383.07 - 2670.55)	26.11 (19.43 - 34.16)
Klebsiella	Beta Lactam/Beta-lactamase inhibitors	Sierra Leone	1779.92 (1276.45 - 2420.65)	28.59 (20.84 - 38.54)
Klebsiella	Trimethoprim-Sulfamethoxazole	Sierra Leone	1760.08 (1267.34 - 2405.1)	28.27 (20.51 - 38.46)
Klebsiella	Fluoroquinolones	Sierra Leone	1389.15 (989.77 - 1925.13)	22.31 (16.1 - 30.66)
Klebsiella	Aminoglycosides	Sierra Leone	1356.15 (951.31 - 1906.55)	21.78 (15.52 - 30.75)
Escherichia coli	Aminopenicillin	Sierra Leone	1283.46 (928.17 - 1756.86)	23.2 (17.07 - 31.41)
Klebsiella	Third-generation cephalosporins	Sierra Leone	1248.55 (535.02 - 2072.7)	20.06 (8.79 - 33.14)
Escherichia coli	Trimethoprim-Sulfamethoxazole	Sierra Leone	1138.87 (822.16 - 1550.46)	20.58 (14.94 - 28.01)
Escherichia coli	Beta Lactam/Beta-lactamase inhibitors	Sierra Leone	1033.42 (734.88 - 1422.17)	18.68 (13.6 - 25.69)
Staphylococcus	Trimethoprim-Sulfamethoxazole	Sierra Leone	937.3 (694.67 - 1260.38)	17.69 (13.49 - 23.33)
Klebsiella	Trimethoprim-Sulfamethoxazole	South Africa	5937.04 (4407.88 - 7810.03)	14.1 (10.4 - 18.61)
Klebsiella	Third-generation cephalosporins	South Africa	5921.64 (4263.13 - 8017.33)	14.06 (10.09 - 19.15)
Klebsiella	Beta Lactam/Beta-lactamase inhibitors	South Africa	5395.86 (3978.9 - 7205.28)	12.81 (9.45 - 17.21)
Escherichia coli	Aminopenicillin	South Africa	4746.33 (3401.87 - 6312.39)	11.57 (8.31 - 15.49)
Escherichia coli	Trimethoprim-Sulfamethoxazole	South Africa	4650.77 (3389.35 - 6187.84)	11.33 (8.24 - 15.16)
Streptococcus	Trimethoprim-Sulfamethoxazole	South Africa	4498.65 (3766.88 - 5372.54)	10.03 (8.41 - 12.02)
Staphylococcus	Macrolide	South Africa	4400.13 (3448.48 - 5563.87)	10.64 (8.36 - 13.55)
Staphylococcus	Methicillin	South Africa	3660.58 (2781.04 - 4773.05)	8.85 (6.74 - 11.57)
Acinetobacter	Third-generation cephalosporins	South Africa	3130.53 (1943.44 - 4713.28)	7.73 (4.7 - 11.69)
Acinetobacter	Anti-pseudomonal penicillin/Beta-	South Africa	3084.44 (1901.93 - 4654.89)	7.62 (4.63 - 11.63)
Streptococcus	Trimethoprim-Sulfamethoxazole	South Sudan	2854.76 (2090.39 - 3857.1)	26.87 (20.74 - 35.18)
Klebsiella	Beta Lactam/Beta-lactamase inhibitors	South Sudan	1720.9 (1277.9 - 2266.95)	22.14 (16.38 - 29.56)

Streptococcus	Fluoroquinolones	South Sudan	1607.29 (1121.53 - 2242.4)	15.13 (11.01 - 20.57)
Klebsiella	Trimethoprim-Sulfamethoxazole	South Sudan	1547.53 (1153.51 - 2058.09)	19.91 (14.48 - 26.79)
Escherichia coli	Aminopenicillin	South Sudan	1383.85 (1020.24 - 1855.12)	20.05 (14.7 - 26.47)
Klebsiella	Aminoglycosides	South Sudan	1354.2 (980.26 - 1842.1)	17.42 (12.51 - 23.85)
Klebsiella	Third-generation cephalosporins	South Sudan	1198.62 (495.16 - 1906.59)	15.42 (6.31 - 24.13)
Escherichia coli	Trimethoprim-Sulfamethoxazole	South Sudan	1178.53 (861.19 - 1582.58)	17.08 (12.53 - 22.81)
Klebsiella	Fluoroquinolones	South Sudan	1068.83 (789.48 - 1463.55)	13.75 (9.94 - 19.22)
Escherichia coli	Beta Lactam/Beta-lactamase inhibitors	South Sudan	1042.46 (759.23 - 1414.11)	15.11 (10.76 - 20.18)
Klebsiella	Trimethoprim-Sulfamethoxazole	Eswatini	170.24 (121.04 - 236.13)	26.03 (18.06 - 36.82)
Klebsiella	Beta Lactam/Beta-lactamase inhibitors	Eswatini	162.89 (116.98 - 222.6)	24.9 (17.48 - 34.83)
Klebsiella	Third-generation cephalosporins	Eswatini	159.84 (89.85 - 234.61)	24.44 (13.38 - 36.27)
Streptococcus	Trimethoprim-Sulfamethoxazole	Eswatini	152.55 (110.96 - 206.05)	19.0 (13.88 - 25.59)
Escherichia coli	Aminopenicillin	Eswatini	133.08 (92.82 - 188.36)	22.08 (15.02 - 31.58)
Staphylococcus	Trimethoprim-Sulfamethoxazole	Eswatini	125.67 (91.47 - 169.58)	20.42 (14.8 - 27.7)
Escherichia coli	Trimethoprim-Sulfamethoxazole	Eswatini	114.02 (79.12 - 161.47)	18.91 (12.82 - 27.05)
Klebsiella	Fluoroquinolones	Eswatini	92.43 (63.77 - 133.08)	14.13 (9.36 - 20.8)
Acinetobacter	Anti-pseudomonal penicillin/Beta-	Eswatini	83.26 (49.05 - 132.46)	16.13 (9.34 - 25.97)
Escherichia coli	Beta Lactam/Beta-lactamase inhibitors	Eswatini	79.15 (53.74 - 115.95)	13.13 (8.71 - 19.17)
Klebsiella	Beta Lactam/Beta-lactamase inhibitors	Tanzania	8899.09 (6715.37 - 11855.11)	22.79 (17.17 - 29.87)
Klebsiella	Trimethoprim-Sulfamethoxazole	Tanzania	8876.06 (6610.29 - 11661.6)	22.74 (16.93 - 29.91)
Streptococcus	Trimethoprim-Sulfamethoxazole	Tanzania	8689.25 (6695.32 - 11175.2)	18.35 (15.04 - 22.41)
Klebsiella	Fluoroquinolones	Tanzania	7030.78 (5185.2 - 9378.85)	18.01 (13.11 - 24.12)
Klebsiella	Aminoglycosides	Tanzania	6880.59 (4893.31 - 9413.43)	17.63 (12.58 - 23.83)
Staphylococcus	Fluoroquinolones	Tanzania	6696.68 (4986.99 - 8752.92)	20.08 (15.32 - 25.66)
Klebsiella	Third-generation cephalosporins	Tanzania	6549.07 (2801.8 - 10216.84)	16.77 (7.31 - 26.16)
Staphylococcus	Trimethoprim-Sulfamethoxazole	Tanzania	6548.11 (5140.48 - 8389.69)	19.64 (15.59 - 24.6)
Escherichia coli	Aminopenicillin	Tanzania	6453.74 (4779.52 - 8527.12)	19.51 (14.31 - 25.55)
Escherichia coli	Trimethoprim-Sulfamethoxazole	Tanzania	5835.53 (4352.79 - 7675.63)	17.64 (12.91 - 23.21)
Klebsiella	Beta Lactam/Beta-lactamase inhibitors	The Gambia	348.98 (263.14 - 456.44)	28.07 (21.0 - 36.74)
Streptococcus	Trimethoprim-Sulfamethoxazole	The Gambia	339.3 (261.84 - 424.14)	25.26 (20.12 - 30.88)
Escherichia coli	Aminopenicillin	The Gambia	270.6 (197.57 - 358.66)	23.58 (17.32 - 31.16)
Klebsiella	Trimethoprim-Sulfamethoxazole	The Gambia	265.28 (200.33 - 346.59)	21.34 (16.12 - 27.77)
Escherichia coli	Trimethoprim-Sulfamethoxazole	The Gambia	240.22 (174.8 - 318.85)	20.93 (15.36 - 27.8)
Klebsiella	Aminoglycosides	The Gambia	231.99 (163.31 - 316.84)	18.66 (13.09 - 25.61)
Escherichia coli	Beta Lactam/Beta-lactamase inhibitors	The Gambia	214.84 (155.53 - 289.05)	18.72 (13.56 - 25.01)
Klebsiella	Fluoroquinolones	The Gambia	196.55 (141.42 - 267.0)	15.8 (11.5 - 21.26)
Staphylococcus	Trimethoprim-Sulfamethoxazole	The Gambia	174.68 (134.94 - 221.34)	16.25 (12.52 - 20.34)
Klebsiella	Third-generation cephalosporins	The Gambia	170.15 (52.35 - 315.45)	13.69 (4.26 - 25.56)
Klebsiella	Trimethoprim-Sulfamethoxazole	Togo	1109.88 (814.52 - 1484.51)	24.4 (18.21 - 32.81)
Klebsiella	Beta Lactam/Beta-lactamase inhibitors	Togo	1079.81 (788.71 - 1440.1)	23.74 (17.72 - 31.5)
Klebsiella	Third-generation cephalosporins	Togo	983.8 (588.68 - 1393.38)	21.63 (13.07 - 30.46)
Streptococcus	Trimethoprim-Sulfamethoxazole	Togo	937.71 (696.1 - 1242.35)	18.4 (14.13 - 23.22)
Escherichia coli	Aminopenicillin	Togo	922.86 (687.4 - 1248.08)	20.61 (15.25 - 27.92)
Klebsiella	Fluoroquinolones	Togo	899.43 (655.67 - 1223.58)	19.78 (14.69 - 26.93)
Escherichia coli	Trimethoprim-Sulfamethoxazole	Togo	859.05 (639.95 - 1155.56)	19.18 (14.15 - 25.98)
Klebsiella	Aminoglycosides	Togo	772.89 (518.43 - 1080.73)	16.99 (11.67 - 23.53)
Escherichia coli	Beta Lactam/Beta-lactamase inhibitors	Togo	735.93 (535.24 - 996.74)	16.44 (11.91 - 22.62)
Staphylococcus	Trimethoprim-Sulfamethoxazole	Togo	713.69 (540.35 - 927.38)	18.07 (13.92 - 23.34)
Klebsiella	Trimethoprim-Sulfamethoxazole	Uganda	4959.79 (3726.89 - 6584.48)	19.67 (14.8 - 25.72)
Klebsiella	Third-generation cephalosporins	Uganda	4850.94 (3022.45 - 7030.73)	19.24 (12.02 - 27.64)
Klebsiella	Beta Lactam/Beta-lactamase inhibitors	Uganda	4849.48 (3543.15 - 6423.05)	19.23 (14.24 - 25.17)
Streptococcus	Trimethoprim-Sulfamethoxazole	Uganda	4758.84 (3521.38 - 6349.85)	16.39 (13.07 - 19.89)
Klebsiella	Fluoroquinolones	Uganda	4092.55 (3011.81 - 5548.09)	16.23 (11.96 - 21.5)
Klebsiella	Aminoglycosides	Uganda	4078.25 (2840.78 - 5569.84)	16.17 (11.21 - 22.45)
Escherichia coli	Trimethoprim-Sulfamethoxazole	Uganda	3871.96 (2882.95 - 5123.17)	18.09 (13.57 - 24.05)
Staphylococcus	Trimethoprim-Sulfamethoxazole	Uganda	2893.07 (2222.12 - 3791.49)	14.49 (11.15 - 18.56)
Escherichia coli	Beta Lactam/Beta-lactamase inhibitors	Uganda	2799.95 (2017.3 - 3820.09)	13.08 (9.43 - 17.68)

Escherichia coli	Aminopenicillin	Uganda	2647.64 (1929.54 - 3506.04)	12.37 (9.18 - 16.52)
Klebsiella	Trimethoprim-Sulfamethoxazole	Zambia	2699.41 (2023.89 - 3593.58)	27.72 (20.54 - 37.26)
Klebsiella	Beta Lactam/Beta-lactamase inhibitors	Zambia	2658.27 (1994.24 - 3539.88)	27.3 (20.3 - 36.64)
Klebsiella	Aminoglycosides	Zambia	2487.65 (1863.97 - 3335.99)	25.55 (18.71 - 34.18)
Staphylococcus	Trimethoprim-Sulfamethoxazole	Zambia	2369.46 (1825.01 - 3085.25)	28.55 (21.96 - 37.64)
Streptococcus	Trimethoprim-Sulfamethoxazole	Zambia	2280.09 (1783.6 - 2904.68)	19.15 (15.35 - 23.68)
Klebsiella	Third-generation cephalosporins	Zambia	2214.61 (1226.69 - 3240.43)	22.75 (12.11 - 33.56)
Escherichia coli	Trimethoprim-Sulfamethoxazole	Zambia	1944.59 (1453.28 - 2614.25)	21.98 (16.05 - 29.97)
Klebsiella	Fluoroquinolones	Zambia	1910.48 (1414.46 - 2614.23)	19.62 (14.3 - 27.03)
Escherichia coli	Aminopenicillin	Zambia	1814.24 (1335.08 - 2454.65)	20.51 (14.88 - 28.05)
Escherichia coli	Beta Lactam/Beta-lactamase inhibitors	Zambia	1517.33 (1108.93 - 2062.18)	17.15 (12.31 - 23.64)
Streptococcus	Trimethoprim-Sulfamethoxazole	Zimbabwe	2583.98 (2025.09 - 3283.41)	24.67 (19.5 - 31.06)
Klebsiella	Trimethoprim-Sulfamethoxazole	Zimbabwe	2552.96 (1945.1 - 3314.95)	28.72 (21.64 - 38.0)
Klebsiella	Beta Lactam/Beta-lactamase inhibitors	Zimbabwe	2526.36 (1926.53 - 3272.73)	28.42 (21.46 - 37.69)
Klebsiella	Third-generation cephalosporins	Zimbabwe	2463.64 (1416.1 - 3477.55)	27.71 (16.15 - 39.67)
Escherichia coli	Aminopenicillin	Zimbabwe	1907.63 (1417.38 - 2530.03)	23.06 (16.95 - 31.34)
Escherichia coli	Trimethoprim-Sulfamethoxazole	Zimbabwe	1788.86 (1334.25 - 2376.39)	21.62 (15.77 - 29.34)
Staphylococcus	Trimethoprim-Sulfamethoxazole	Zimbabwe	1527.22 (1198.36 - 1939.07)	19.13 (14.7 - 24.84)
Klebsiella	Fluoroquinolones	Zimbabwe	1451.13 (1041.61 - 1974.18)	16.32 (11.61 - 22.63)
Klebsiella	Aminoglycosides	Zimbabwe	1219.14 (784.24 - 1785.54)	13.71 (8.58 - 20.48)
Escherichia coli	Beta Lactam/Beta-lactamase inhibitors	Zimbabwe	1204.32 (869.91 - 1652.25)	14.56 (10.28 - 20.1)

Supplementary Table 5. DALYS attributable to antimicrobial resistance (expressed as counts and age-standardised rates (ASDR) per 100 000 with 95% uncertainty intervals) per country in the WHO African region and top 10 pathogen–drug combination.

Pathogen	Antibiotic Class	Country	DALYs attributable to AMR (counts)	DALYs attributable to AMR (age-standardised rate per 100 thousand)
Staphylococcus aureus	Methicillin	Algeria	14790.5 (6341.5 - 26834.5)	41.3 (18.0 - 74.2)
Klebsiella pneumoniae	Third-generation cephalosporins	Algeria	10622.8 (2887.5 - 22703.1)	28.9 (7.9 - 62.0)
Streptococcus pneumoniae	Carbapenems	Algeria	8186.6 (3643.5 - 15127.3)	21.2 (9.5 - 38.9)
Acinetobacter baumannii	Carbapenems	Algeria	4497.6 (2030.4 - 8477.3)	12.8 (5.8 - 24.5)
Streptococcus pneumoniae	Trimethoprim-Sulfamethoxazole	Algeria	3968.9 (578.1 - 8068.3)	10.3 (1.5 - 20.7)
Acinetobacter baumannii	Fluoroquinolones	Algeria	3465.5 (1582.7 - 6251.4)	9.9 (4.5 - 17.7)
Escherichia coli	Fluoroquinolones	Algeria	3451.9 (1981.8 - 5726.2)	9.7 (5.6 - 16.2)
Pseudomonas aeruginosa	Carbapenems	Algeria	3320.2 (1725.9 - 5537.2)	9.1 (4.7 - 15.2)
Enterococcus faecium	Fluoroquinolones	Algeria	3278.5 (915.4 - 6423.4)	9.2 (2.6 - 17.9)
Staphylococcus aureus	Macrolide	Algeria	3105.4 (1132.9 - 5923.1)	8.7 (3.2 - 16.2)
Streptococcus pneumoniae	Trimethoprim-Sulfamethoxazole	Angola	28347.9 (4004.5 - 58866.3)	75.1 (10.8 - 150.0)
Klebsiella pneumoniae	Third-generation cephalosporins	Angola	24401.7 (6466.0 - 49336.0)	74.1 (19.6 - 151.4)
Streptococcus pneumoniae	Carbapenems	Angola	15031.4 (6338.6 - 29057.1)	39.9 (17.3 - 75.6)
Staphylococcus aureus	Methicillin	Angola	12137.6 (5039.1 - 21681.3)	42.4 (17.7 - 75.4)
Klebsiella pneumoniae	Aminoglycosides	Angola	11895.3 (6879.9 - 18626.7)	36.1 (20.7 - 58.6)
Escherichia coli	Trimethoprim-Sulfamethoxazole	Angola	10750.8 (7045.0 - 15432.0)	35.3 (23.4 - 50.0)
Klebsiella pneumoniae	Fluoroquinolones	Angola	10214.6 (5845.4 - 15755.1)	31.1 (17.7 - 48.7)
Klebsiella pneumoniae	Trimethoprim-Sulfamethoxazole	Angola	10173.2 (4917.3 - 16828.7)	30.9 (15.0 - 51.6)
Group B Streptococcus	Fluoroquinolones	Angola	10167.4 (1623.7 - 22986.6)	24.6 (4.0 - 55.2)
Staphylococcus aureus	Trimethoprim-Sulfamethoxazole	Angola	9777.1 (4976.6 - 15521.7)	34.2 (17.7 - 55.2)
Streptococcus pneumoniae	Trimethoprim-Sulfamethoxazole	Benin	18508.5 (2276.8 - 38869.1)	103.1 (13.1 - 213.4)
Klebsiella pneumoniae	Third-generation cephalosporins	Benin	18390.2 (5292.2 - 36553.2)	110.5 (31.2 - 219.3)
Staphylococcus aureus	Methicillin	Benin	11582.6 (4667.8 - 21253.6)	79.2 (31.5 - 142.8)
Streptococcus pneumoniae	Carbapenems	Benin	10239.8 (4089.1 - 20114.0)	57.1 (23.4 - 110.6)
Staphylococcus aureus	Trimethoprim-Sulfamethoxazole	Benin	9078.5 (4591.0 - 14643.6)	62.1 (31.7 - 97.6)
Klebsiella pneumoniae	Aminoglycosides	Benin	9043.1 (5305.8 - 13971.1)	54.3 (31.4 - 84.0)
Klebsiella pneumoniae	Fluoroquinolones	Benin	8215.4 (4665.6 - 13062.1)	49.4 (28.3 - 78.0)
Klebsiella pneumoniae	Trimethoprim-Sulfamethoxazole	Benin	7649.4 (3639.0 - 12776.9)	46.0 (22.1 - 77.2)
Escherichia coli	Trimethoprim-Sulfamethoxazole	Benin	7198.1 (4645.3 - 10420.6)	46.5 (30.2 - 66.7)
Group B Streptococcus	Fluoroquinolones	Benin	6774.6 (1209.6 - 14832.2)	35.1 (6.3 - 77.0)
Klebsiella pneumoniae	Third-generation cephalosporins	Botswana	1779.0 (498.5 - 3843.9)	93.1 (26.3 - 200.0)
Streptococcus pneumoniae	Trimethoprim-Sulfamethoxazole	Botswana	1215.5 (165.1 - 2531.4)	57.9 (7.9 - 119.5)
Streptococcus pneumoniae	Carbapenems	Botswana	1084.1 (479.2 - 2010.1)	51.7 (22.9 - 94.3)
Staphylococcus aureus	Trimethoprim-Sulfamethoxazole	Botswana	673.7 (331.7 - 1102.5)	36.5 (18.1 - 59.5)
Pseudomonas aeruginosa	Carbapenems	Botswana	664.6 (422.7 - 1023.2)	35.6 (22.6 - 54.3)
Klebsiella pneumoniae	Trimethoprim-Sulfamethoxazole	Botswana	656.0 (320.5 - 1108.2)	34.3 (17.0 - 57.6)
Escherichia coli	Trimethoprim-Sulfamethoxazole	Botswana	637.6 (410.2 - 944.9)	35.0 (22.7 - 51.2)
Acinetobacter baumannii	Carbapenems	Botswana	516.3 (258.2 - 910.3)	30.0 (15.0 - 53.4)
Escherichia coli	Third-generation cephalosporins	Botswana	505.2 (190.5 - 1021.5)	27.7 (10.4 - 56.2)
Streptococcus pneumoniae	Penicillin	Botswana	476.4 (161.8 - 1000.8)	22.7 (7.7 - 47.4)
Klebsiella pneumoniae	Third-generation cephalosporins	Burkina Faso	49149.4 (16143.2 - 98159.1)	154.1 (50.6 - 308.6)
Streptococcus pneumoniae	Trimethoprim-Sulfamethoxazole	Burkina Faso	40425.0 (5346.7 - 85248.7)	117.8 (15.8 - 248.4)
Staphylococcus aureus	Methicillin	Burkina Faso	27425.3 (10977.0 - 49938.4)	96.2 (40.3 - 172.2)
Streptococcus pneumoniae	Carbapenems	Burkina Faso	20608.7 (8511.1 - 38152.2)	60.1 (25.3 - 109.2)
Klebsiella pneumoniae	Fluoroquinolones	Burkina Faso	19877.5 (11712.2 - 31123.9)	62.4 (37.1 - 97.0)
Klebsiella pneumoniae	Aminoglycosides	Burkina Faso	19797.9 (11627.0 - 30866.1)	62.1 (37.6 - 94.2)
Staphylococcus aureus	Trimethoprim-Sulfamethoxazole	Burkina Faso	19425.0 (9512.6 - 29748.4)	68.2 (34.3 - 104.0)
Klebsiella pneumoniae	Trimethoprim-Sulfamethoxazole	Burkina Faso	19291.6 (9188.7 - 31982.4)	60.5 (29.5 - 100.5)
Escherichia coli	Trimethoprim-Sulfamethoxazole	Burkina Faso	14484.1 (9563.3 - 20598.8)	49.4 (33.4 - 69.8)

Escherichia coli	Third-generation cephalosporins	Burkina Faso	14189.7 (5564.8 - 28871.6)	48.4 (19.1 - 98.9)
Klebsiella pneumoniae	Third-generation cephalosporins	Burundi	11427.8 (2811.1 - 26149.8)	87.0 (22.2 - 199.1)
Streptococcus pneumoniae	Trimethoprim-Sulfamethoxazole	Burundi	9656.1 (1392.1 - 20565.0)	68.6 (10.2 - 141.7)
Streptococcus pneumoniae	Fluoroquinolones	Burundi	8245.9 (1815.2 - 17740.8)	58.7 (12.8 - 124.6)
Klebsiella pneumoniae	Aminoglycosides	Burundi	7124.6 (4205.6 - 11167.3)	54.2 (32.7 - 85.2)
Staphylococcus aureus	Methicillin	Burundi	6991.9 (2807.6 - 12769.1)	62.8 (25.5 - 113.5)
Staphylococcus aureus	Trimethoprim-Sulfamethoxazole	Burundi	5965.4 (3019.9 - 9534.8)	53.6 (27.4 - 83.4)
Escherichia coli	Trimethoprim-Sulfamethoxazole	Burundi	5604.2 (3521.1 - 8358.7)	43.1 (27.9 - 62.2)
Streptococcus pneumoniae	Carbapenems	Burundi	5152.8 (2063.7 - 10138.0)	36.8 (15.1 - 69.0)
Klebsiella pneumoniae	Trimethoprim-Sulfamethoxazole	Burundi	4940.2 (2254.7 - 8349.6)	37.5 (17.4 - 63.3)
Escherichia coli	Third-generation cephalosporins	Burundi	4802.8 (1728.1 - 10085.2)	36.9 (13.7 - 73.6)
Streptococcus pneumoniae	Trimethoprim-Sulfamethoxazole	Cameroon	27644.8 (3799.8 - 57328.9)	85.0 (12.2 - 175.8)
Staphylococcus aureus	Methicillin	Cameroon	23386.8 (9787.3 - 40713.8)	88.4 (37.6 - 151.8)
Klebsiella pneumoniae	Third-generation cephalosporins	Cameroon	16230.5 (3957.0 - 40437.5)	54.5 (13.1 - 136.1)
Staphylococcus aureus	Trimethoprim-Sulfamethoxazole	Cameroon	13704.4 (7174.4 - 21399.5)	51.8 (27.2 - 80.2)
Streptococcus pneumoniae	Carbapenems	Cameroon	13095.6 (5566.0 - 24591.5)	40.3 (17.0 - 74.7)
Escherichia coli	Third-generation cephalosporins	Cameroon	12760.7 (5225.8 - 24490.0)	44.5 (18.0 - 85.8)
Klebsiella pneumoniae	Fluoroquinolones	Cameroon	12163.2 (7161.0 - 19028.4)	40.9 (24.2 - 63.3)
Klebsiella pneumoniae	Aminoglycosides	Cameroon	12036.2 (6795.4 - 18320.4)	40.4 (22.6 - 62.0)
Klebsiella pneumoniae	Trimethoprim-Sulfamethoxazole	Cameroon	11083.4 (5278.2 - 18222.0)	37.2 (18.3 - 61.7)
Escherichia coli	Trimethoprim-Sulfamethoxazole	Cameroon	10999.3 (7261.9 - 15804.9)	38.3 (24.7 - 56.2)
Staphylococcus aureus	Methicillin	Cape Verde	181.8 (76.1 - 313.9)	38.8 (16.3 - 66.8)
Acinetobacter baumannii	Carbapenems	Cape Verde	147.2 (75.7 - 260.1)	32.0 (16.4 - 57.1)
Klebsiella pneumoniae	Third-generation cephalosporins	Cape Verde	137.4 (41.6 - 290.4)	29.2 (8.9 - 61.2)
Streptococcus pneumoniae	Trimethoprim-Sulfamethoxazole	Cape Verde	136.3 (19.0 - 266.4)	27.8 (3.9 - 54.0)
Acinetobacter baumannii	Fluoroquinolones	Cape Verde	127.1 (63.3 - 221.7)	27.7 (13.7 - 48.1)
Streptococcus pneumoniae	Carbapenems	Cape Verde	126.6 (56.7 - 217.6)	25.8 (11.5 - 44.2)
Escherichia coli	Third-generation cephalosporins	Cape Verde	76.5 (31.2 - 145.8)	16.4 (6.7 - 31.1)
Escherichia coli	Trimethoprim-Sulfamethoxazole	Cape Verde	63.1 (39.3 - 93.6)	13.5 (8.5 - 19.9)
Pseudomonas aeruginosa	Carbapenems	Cape Verde	59.4 (36.2 - 89.9)	12.7 (7.9 - 19.2)
Acinetobacter baumannii	Anti-pseudomonal penicillin/Beta-	Cape Verde	54.7 (23.0 - 99.6)	11.9 (5.0 - 21.5)
Streptococcus pneumoniae	Fluoroquinolones	Central	15056.0 (3984.2 - 30346.1)	213.4 (56.6 - 433.3)
Klebsiella pneumoniae	Third-generation cephalosporins	Central	8291.2 (2024.9 - 17536.7)	128.4 (31.5 - 269.9)
Streptococcus pneumoniae	Trimethoprim-Sulfamethoxazole	Central	7562.2 (1236.7 - 16043.9)	107.1 (17.3 - 223.3)
Group B Streptococcus	Fluoroquinolones	Central	7292.9 (1206.7 - 16369.6)	93.5 (15.6 - 205.7)
Mycobacterium tuberculosis	Isoniazid mono-resistance	Central	6127.3 (0.0 - 21913.7)	144.1 (0.0 - 525.8)
Klebsiella pneumoniae	Aminoglycosides	Central	6027.6 (3542.6 - 9292.5)	93.3 (54.8 - 144.6)
Klebsiella pneumoniae	Fluoroquinolones	Central	4936.0 (2793.3 - 7626.3)	76.5 (43.5 - 116.3)
Streptococcus pneumoniae	Beta Lactam/Beta-lactamase inhibitors	Central	4798.3 (61.8 - 11374.3)	68.0 (0.8 - 159.7)
Streptococcus pneumoniae	Carbapenems	Central	4629.6 (1881.1 - 9182.4)	65.7 (26.9 - 129.0)
Salmonella Typhi	Fluoroquinolones	Central	4529.8 (355.2 - 12690.5)	60.0 (4.6 - 166.9)
Streptococcus pneumoniae	Trimethoprim-Sulfamethoxazole	Chad	48928.8 (7108.1 - 101304.2)	169.5 (25.3 - 342.5)
Klebsiella pneumoniae	Third-generation cephalosporins	Chad	39349.4 (14071.6 - 74135.5)	155.2 (55.1 - 287.0)
Streptococcus pneumoniae	Carbapenems	Chad	24388.6 (9169.8 - 45149.8)	84.6 (32.5 - 154.4)
Streptococcus pneumoniae	Fluoroquinolones	Chad	24065.5 (5464.5 - 49907.1)	83.4 (19.2 - 174.0)
Group B Streptococcus	Fluoroquinolones	Chad	19859.3 (3350.8 - 43221.3)	64.4 (11.1 - 138.7)
Escherichia coli	Third-generation cephalosporins	Chad	16100.5 (6687.6 - 29838.6)	64.9 (27.0 - 119.9)
Escherichia coli	Fluoroquinolones	Chad	15937.8 (10932.6 - 22813.4)	64.2 (45.3 - 90.3)
Klebsiella pneumoniae	Aminoglycosides	Chad	15574.3 (9366.1 - 23346.4)	61.5 (37.4 - 91.5)
Escherichia coli	Trimethoprim-Sulfamethoxazole	Chad	11808.1 (7846.4 - 16733.1)	47.5 (32.2 - 66.5)
Staphylococcus aureus	Methicillin	Chad	11802.7 (4601.5 - 20903.6)	53.9 (21.4 - 94.3)
Streptococcus pneumoniae	Trimethoprim-Sulfamethoxazole	Comoros	590.4 (79.6 - 1134.3)	82.7 (11.2 - 159.5)
Streptococcus pneumoniae	Carbapenems	Comoros	411.0 (177.3 - 762.2)	57.6 (25.3 - 106.4)
Klebsiella pneumoniae	Third-generation cephalosporins	Comoros	383.4 (101.2 - 855.7)	57.3 (15.4 - 128.2)
Acinetobacter baumannii	Anti-pseudomonal penicillin/Beta-	Comoros	337.7 (173.9 - 564.6)	57.5 (29.5 - 95.7)
Escherichia coli	Third-generation cephalosporins	Comoros	236.2 (99.3 - 446.8)	36.3 (15.3 - 68.6)
Klebsiella pneumoniae	Aminoglycosides	Comoros	218.7 (129.7 - 335.9)	32.7 (19.4 - 50.1)

Staphylococcus aureus	Trimethoprim-Sulfamethoxazole	Comoros	203.5 (103.4 - 317.6)	31.7 (16.3 - 49.3)
Staphylococcus aureus	Methicillin	Comoros	195.4 (77.3 - 354.7)	30.5 (11.9 - 54.8)
Klebsiella pneumoniae	Fluoroquinolones	Comoros	187.7 (111.0 - 291.5)	28.1 (16.8 - 43.8)
Escherichia coli	Trimethoprim-Sulfamethoxazole	Comoros	185.4 (124.7 - 264.6)	28.5 (19.3 - 40.1)
Klebsiella pneumoniae	Third-generation cephalosporins	Congo	3054.1 (917.8 - 6220.0)	68.3 (20.3 - 140.1)
Staphylococcus aureus	Methicillin	Congo	2654.4 (1087.3 - 4805.9)	65.8 (27.4 - 117.7)
Streptococcus pneumoniae	Trimethoprim-Sulfamethoxazole	Congo	2418.9 (347.6 - 5242.3)	50.0 (7.4 - 105.8)
Klebsiella pneumoniae	Aminoglycosides	Congo	1474.2 (880.6 - 2332.7)	33.0 (19.6 - 52.7)
Klebsiella pneumoniae	Fluoroquinolones	Congo	1450.1 (851.8 - 2239.2)	32.4 (18.9 - 51.0)
Escherichia coli	Third-generation cephalosporins	Congo	1438.8 (571.6 - 2897.9)	33.3 (13.2 - 65.6)
Staphylococcus aureus	Trimethoprim-Sulfamethoxazole	Congo	1354.9 (693.6 - 2142.5)	33.6 (17.3 - 53.1)
Streptococcus pneumoniae	Carbapenems	Congo	1315.1 (553.9 - 2429.0)	27.2 (11.8 - 50.0)
Escherichia coli	Trimethoprim-Sulfamethoxazole	Congo	1265.7 (826.1 - 1836.0)	29.3 (19.5 - 42.4)
Escherichia coli	Fluoroquinolones	Congo	1204.6 (758.2 - 1805.1)	27.9 (17.8 - 41.9)
Klebsiella pneumoniae	Third-generation cephalosporins	Cote d'Ivoire	32505.8 (9521.4 - 65500.2)	108.3 (31.4 - 216.8)
Streptococcus pneumoniae	Trimethoprim-Sulfamethoxazole	Cote d'Ivoire	29449.9 (3761.2 - 61286.0)	92.8 (11.9 - 189.1)
Staphylococcus aureus	Methicillin	Cote d'Ivoire	18221.4 (7565.2 - 33268.0)	68.7 (28.5 - 124.4)
Staphylococcus aureus	Trimethoprim-Sulfamethoxazole	Cote d'Ivoire	16441.6 (8314.7 - 25716.1)	62.0 (32.2 - 95.9)
Streptococcus pneumoniae	Carbapenems	Cote d'Ivoire	16430.0 (6749.2 - 30571.8)	51.8 (21.7 - 95.3)
Klebsiella pneumoniae	Aminoglycosides	Cote d'Ivoire	15291.8 (9056.1 - 22846.0)	51.0 (29.8 - 76.8)
Escherichia coli	Third-generation cephalosporins	Cote d'Ivoire	13788.0 (5572.8 - 26596.5)	48.5 (19.6 - 93.1)
Klebsiella pneumoniae	Fluoroquinolones	Cote d'Ivoire	13743.1 (7891.3 - 20971.4)	45.8 (26.5 - 69.6)
Group B Streptococcus	Fluoroquinolones	Cote d'Ivoire	13234.6 (2246.6 - 29240.4)	37.8 (6.4 - 82.6)
Klebsiella pneumoniae	Trimethoprim-Sulfamethoxazole	Cote d'Ivoire	12875.0 (6107.1 - 21151.0)	42.9 (20.7 - 70.0)
Klebsiella pneumoniae	Third-generation cephalosporins	Democratic	77963.5 (23547.7 - 157257.6)	89.8 (27.8 - 184.1)
Streptococcus pneumoniae	Fluoroquinolones	Democratic	60289.3 (14457.9 - 124448.8)	64.9 (16.4 - 133.4)
Streptococcus pneumoniae	Trimethoprim-Sulfamethoxazole	Democratic	54577.9 (7945.3 - 116328.4)	58.7 (8.8 - 118.7)
Staphylococcus aureus	Methicillin	Democratic	46828.2 (19663.4 - 84599.8)	62.3 (26.5 - 112.8)
Klebsiella pneumoniae	Aminoglycosides	Democratic	36229.6 (20890.7 - 54832.9)	41.7 (24.4 - 64.2)
Escherichia coli	Third-generation cephalosporins	Democratic	33530.0 (13940.8 - 63448.3)	40.3 (16.9 - 78.0)
Staphylococcus aureus	Trimethoprim-Sulfamethoxazole	Democratic	32690.7 (16815.8 - 51578.2)	43.5 (22.4 - 68.6)
Escherichia coli	Fluoroquinolones	Democratic	30922.3 (19998.1 - 45245.5)	37.2 (24.9 - 53.9)
Salmonella Typhi	Fluoroquinolones	Democratic	30740.3 (5237.5 - 72130.3)	26.9 (4.7 - 61.8)
Klebsiella pneumoniae	Trimethoprim-Sulfamethoxazole	Democratic	29107.4 (13626.0 - 48075.4)	33.5 (16.1 - 54.8)
Streptococcus pneumoniae	Trimethoprim-Sulfamethoxazole	Equatorial	543.9 (79.6 - 1184.6)	42.7 (6.6 - 89.2)
Klebsiella pneumoniae	Third-generation cephalosporins	Equatorial	420.6 (98.6 - 1009.8)	36.5 (8.5 - 88.1)
Staphylococcus aureus	Methicillin	Equatorial	411.6 (155.9 - 758.7)	40.7 (15.9 - 74.3)
Acinetobacter baumannii	Carbapenems	Equatorial	330.9 (169.5 - 603.8)	32.3 (16.6 - 57.8)
Acinetobacter baumannii	Fluoroquinolones	Equatorial	329.7 (161.4 - 593.6)	32.2 (15.6 - 57.5)
Streptococcus pneumoniae	Carbapenems	Equatorial	322.8 (129.9 - 667.1)	25.3 (10.5 - 50.3)
Klebsiella pneumoniae	Fluoroquinolones	Equatorial	232.8 (126.6 - 388.5)	20.2 (11.0 - 33.6)
Escherichia coli	Third-generation cephalosporins	Equatorial	229.1 (87.2 - 475.2)	22.6 (8.6 - 46.2)
Klebsiella pneumoniae	Aminoglycosides	Equatorial	216.5 (113.9 - 376.2)	18.8 (9.9 - 32.4)
Streptococcus pneumoniae	Penicillin	Equatorial	208.6 (67.5 - 469.9)	16.4 (5.5 - 36.3)
Klebsiella pneumoniae	Third-generation cephalosporins	Eritrea	5395.0 (1328.0 - 12086.0)	93.0 (22.9 - 205.8)
Streptococcus pneumoniae	Trimethoprim-Sulfamethoxazole	Eritrea	5329.6 (795.0 - 11243.2)	82.2 (12.0 - 176.4)
Streptococcus pneumoniae	Fluoroquinolones	Eritrea	4190.4 (962.6 - 8829.2)	64.6 (14.5 - 138.1)
Staphylococcus aureus	Trimethoprim-Sulfamethoxazole	Eritrea	3980.8 (1873.4 - 6730.8)	77.8 (36.3 - 132.8)
Klebsiella pneumoniae	Aminoglycosides	Eritrea	3241.0 (1863.7 - 5293.7)	55.8 (32.3 - 90.1)
Escherichia coli	Third-generation cephalosporins	Eritrea	3156.0 (1284.8 - 6359.4)	55.6 (23.2 - 112.8)
Streptococcus pneumoniae	Carbapenems	Eritrea	2873.7 (1158.0 - 5612.4)	44.4 (17.6 - 84.5)
Staphylococcus aureus	Methicillin	Eritrea	2738.4 (1081.0 - 5151.7)	53.5 (20.3 - 102.2)
Escherichia coli	Fluoroquinolones	Eritrea	2356.8 (1486.3 - 3652.6)	41.5 (26.5 - 62.3)
Escherichia coli	Trimethoprim-Sulfamethoxazole	Eritrea	2349.8 (1506.1 - 3541.3)	41.4 (26.8 - 61.9)
Klebsiella pneumoniae	Third-generation cephalosporins	Ethiopia	101373.5 (41006.3 - 188467.4)	89.6 (36.7 - 166.4)
Streptococcus pneumoniae	Trimethoprim-Sulfamethoxazole	Ethiopia	94727.9 (13507.6 - 194110.8)	75.6 (10.9 - 151.2)
Klebsiella pneumoniae	Carbapenems	Ethiopia	68328.0 (40448.5 - 107728.7)	60.4 (35.9 - 92.9)

Staphylococcus aureus	Methicillin	Ethiopia	66271.9 (29489.7 - 114469.5)	67.6 (29.4 - 114.4)
Escherichia coli	Third-generation cephalosporins	Ethiopia	65356.8 (28886.7 - 119240.8)	62.9 (28.1 - 115.2)
Streptococcus pneumoniae	Carbapenems	Ethiopia	53353.3 (23012.7 - 98564.9)	42.6 (18.7 - 76.8)
Klebsiella pneumoniae	Aminoglycosides	Ethiopia	51800.4 (31646.4 - 77553.1)	45.8 (29.0 - 67.6)
Escherichia coli	Fluoroquinolones	Ethiopia	45311.0 (31155.5 - 63354.7)	43.6 (31.1 - 59.8)
Klebsiella pneumoniae	Trimethoprim-Sulfamethoxazole	Ethiopia	37287.6 (17590.7 - 61549.7)	33.0 (15.7 - 53.8)
Escherichia coli	Trimethoprim-Sulfamethoxazole	Ethiopia	36278.0 (24408.5 - 51371.0)	34.9 (24.0 - 47.2)
Staphylococcus aureus	Methicillin	Gabon	700.7 (298.7 - 1213.4)	50.6 (22.5 - 86.9)
Streptococcus pneumoniae	Trimethoprim-Sulfamethoxazole	Gabon	616.2 (84.3 - 1297.9)	38.7 (5.6 - 81.7)
Klebsiella pneumoniae	Third-generation cephalosporins	Gabon	583.2 (157.5 - 1342.0)	39.8 (10.8 - 91.0)
Acinetobacter baumannii	Fluoroquinolones	Gabon	417.2 (204.0 - 731.6)	32.7 (15.8 - 56.8)
Acinetobacter baumannii	Carbapenems	Gabon	374.5 (186.8 - 661.1)	29.4 (14.7 - 52.1)
Acinetobacter baumannii	Anti-pseudomonal penicillin/Beta-	Gabon	332.3 (150.4 - 581.6)	26.0 (11.4 - 45.4)
Streptococcus pneumoniae	Carbapenems	Gabon	322.1 (135.1 - 621.5)	20.3 (8.4 - 38.1)
Klebsiella pneumoniae	Fluoroquinolones	Gabon	303.6 (170.3 - 491.9)	20.7 (11.8 - 33.4)
Escherichia coli	Third-generation cephalosporins	Gabon	275.5 (101.7 - 545.7)	19.7 (7.4 - 39.1)
Klebsiella pneumoniae	Aminoglycosides	Gabon	253.4 (142.6 - 415.7)	17.3 (9.7 - 28.2)
Klebsiella pneumoniae	Third-generation cephalosporins	Ghana	23671.9 (8383.0 - 45271.9)	82.3 (29.1 - 156.9)
Streptococcus pneumoniae	Trimethoprim-Sulfamethoxazole	Ghana	21794.9 (2723.9 - 44259.5)	73.1 (9.2 - 145.5)
Staphylococcus aureus	Methicillin	Ghana	16592.5 (7226.0 - 29618.0)	65.7 (28.4 - 114.9)
Escherichia coli	Third-generation cephalosporins	Ghana	13262.8 (5472.7 - 25597.9)	50.2 (21.0 - 93.6)
Klebsiella pneumoniae	Aminoglycosides	Ghana	12737.9 (7620.1 - 19677.2)	44.3 (27.0 - 67.8)
Staphylococcus aureus	Trimethoprim-Sulfamethoxazole	Ghana	10464.7 (5194.7 - 16419.9)	41.4 (21.0 - 63.8)
Escherichia coli	Fluoroquinolones	Ghana	10392.8 (6912.2 - 14686.4)	39.3 (27.3 - 55.3)
Klebsiella pneumoniae	Fluoroquinolones	Ghana	9696.7 (5630.1 - 14945.0)	33.7 (19.9 - 52.0)
Klebsiella pneumoniae	Trimethoprim-Sulfamethoxazole	Ghana	9637.6 (4734.6 - 16220.8)	33.5 (16.2 - 55.2)
Streptococcus pneumoniae	Carbapenems	Ghana	8840.1 (3669.3 - 16494.3)	29.7 (13.1 - 55.4)
Streptococcus pneumoniae	Trimethoprim-Sulfamethoxazole	Guinea	29894.3 (4134.9 - 64245.2)	164.8 (22.7 - 350.9)
Klebsiella pneumoniae	Third-generation cephalosporins	Guinea	19170.0 (5841.4 - 40082.7)	118.7 (35.9 - 249.9)
Streptococcus pneumoniae	Carbapenems	Guinea	16140.7 (6712.2 - 31246.9)	89.1 (36.9 - 170.3)
Streptococcus pneumoniae	Fluoroquinolones	Guinea	14970.1 (3369.7 - 31821.9)	82.6 (18.8 - 175.1)
Group B Streptococcus	Fluoroquinolones	Guinea	13154.3 (2294.5 - 29144.3)	69.8 (12.5 - 154.1)
Klebsiella pneumoniae	Aminoglycosides	Guinea	9016.9 (5240.2 - 13899.6)	55.8 (32.7 - 84.4)
Staphylococcus aureus	Methicillin	Guinea	8202.9 (3343.0 - 15008.8)	57.0 (23.3 - 103.7)
Escherichia coli	Third-generation cephalosporins	Guinea	7619.6 (2987.2 - 14950.9)	50.1 (19.8 - 98.5)
Klebsiella pneumoniae	Fluoroquinolones	Guinea	7444.8 (4264.8 - 11775.0)	46.1 (26.8 - 73.2)
Klebsiella pneumoniae	Trimethoprim-Sulfamethoxazole	Guinea	7266.4 (3310.2 - 11998.4)	45.0 (20.9 - 75.7)
Klebsiella pneumoniae	Third-generation cephalosporins	Guinea-	2022.1 (560.9 - 4300.6)	107.8 (30.1 - 229.3)
Streptococcus pneumoniae	Trimethoprim-Sulfamethoxazole	Guinea-	1606.5 (221.2 - 3400.7)	82.7 (11.6 - 171.0)
Klebsiella pneumoniae	Aminoglycosides	Guinea-	1053.2 (617.8 - 1633.1)	56.2 (33.5 - 86.7)
Staphylococcus aureus	Trimethoprim-Sulfamethoxazole	Guinea-	1033.8 (516.8 - 1638.8)	64.9 (33.0 - 102.8)
Klebsiella pneumoniae	Trimethoprim-Sulfamethoxazole	Guinea-	914.1 (441.5 - 1502.4)	48.7 (23.4 - 80.2)
Klebsiella pneumoniae	Fluoroquinolones	Guinea-	905.1 (512.5 - 1393.7)	48.3 (28.0 - 74.0)
Streptococcus pneumoniae	Fluoroquinolones	Guinea-	856.1 (193.8 - 1851.3)	44.1 (10.3 - 93.7)
Staphylococcus aureus	Methicillin	Guinea-	848.1 (345.6 - 1491.3)	53.2 (21.9 - 94.0)
Escherichia coli	Third-generation cephalosporins	Guinea-	761.4 (281.1 - 1500.0)	43.0 (16.1 - 84.6)
Streptococcus pneumoniae	Carbapenems	Guinea-	743.1 (298.0 - 1464.5)	38.3 (15.6 - 73.2)
Klebsiella pneumoniae	Third-generation cephalosporins	Kenya	26793.0 (9071.1 - 56006.2)	65.3 (22.0 - 134.8)
Streptococcus pneumoniae	Trimethoprim-Sulfamethoxazole	Kenya	26722.6 (4004.8 - 51081.3)	58.2 (8.7 - 112.8)
Streptococcus pneumoniae	Carbapenems	Kenya	22776.4 (10227.7 - 40403.9)	49.6 (22.6 - 87.4)
Escherichia coli	Third-generation cephalosporins	Kenya	18784.4 (7573.1 - 34601.0)	48.2 (19.6 - 89.8)
Salmonella Typhi	Fluoroquinolones	Kenya	17881.7 (2414.2 - 43420.2)	30.1 (4.2 - 71.8)
Staphylococcus aureus	Trimethoprim-Sulfamethoxazole	Kenya	16347.1 (8405.2 - 25301.6)	44.3 (22.7 - 68.8)
Klebsiella pneumoniae	Aminoglycosides	Kenya	14502.9 (8897.3 - 22132.7)	35.4 (21.5 - 54.3)
Escherichia coli	Trimethoprim-Sulfamethoxazole	Kenya	14059.2 (9771.8 - 18995.3)	36.1 (25.1 - 49.3)
Klebsiella pneumoniae	Trimethoprim-Sulfamethoxazole	Kenya	13845.5 (6790.6 - 22421.6)	33.7 (16.6 - 54.9)
Staphylococcus aureus	Methicillin	Kenya	13733.8 (5537.4 - 25167.1)	37.2 (15.0 - 67.3)

Klebsiella pneumoniae	Third-generation cephalosporins	Lesotho	3451.4 (1257.2 - 6475.9)	192.1 (70.9 - 361.6)
Streptococcus pneumoniae	Trimethoprim-Sulfamethoxazole	Lesotho	1735.8 (257.1 - 3505.5)	89.7 (13.4 - 180.3)
Staphylococcus aureus	Methicillin	Lesotho	1181.1 (473.0 - 2156.7)	68.0 (27.9 - 124.0)
Streptococcus pneumoniae	Carbapenems	Lesotho	1142.7 (487.0 - 2004.2)	59.1 (25.1 - 104.0)
Staphylococcus aureus	Trimethoprim-Sulfamethoxazole	Lesotho	972.8 (480.3 - 1536.8)	56.0 (27.9 - 88.7)
Klebsiella pneumoniae	Trimethoprim-Sulfamethoxazole	Lesotho	918.4 (456.5 - 1552.0)	51.1 (25.2 - 85.6)
Escherichia coli	Third-generation cephalosporins	Lesotho	855.3 (334.7 - 1689.0)	49.3 (19.1 - 97.4)
Klebsiella pneumoniae	Fluoroquinolones	Lesotho	842.3 (476.6 - 1299.4)	46.9 (26.5 - 72.4)
Escherichia coli	Trimethoprim-Sulfamethoxazole	Lesotho	834.6 (558.0 - 1208.7)	48.1 (32.1 - 69.0)
Mycobacterium tuberculosis	Rifampicin mono-resistance	Lesotho	739.7 (116.7 - 1790.2)	40.3 (6.3 - 96.3)
Klebsiella pneumoniae	Third-generation cephalosporins	Liberia	3857.2 (1149.8 - 8163.9)	84.7 (25.6 - 175.9)
Streptococcus pneumoniae	Trimethoprim-Sulfamethoxazole	Liberia	2997.6 (396.4 - 6488.8)	62.4 (8.2 - 132.0)
Klebsiella pneumoniae	Aminoglycosides	Liberia	1931.2 (1074.6 - 2960.4)	42.5 (24.2 - 64.9)
Staphylococcus aureus	Trimethoprim-Sulfamethoxazole	Liberia	1879.9 (997.3 - 2963.0)	47.0 (24.5 - 75.4)
Klebsiella pneumoniae	Fluoroquinolones	Liberia	1695.6 (972.8 - 2715.6)	37.3 (21.8 - 58.4)
Staphylococcus aureus	Methicillin	Liberia	1606.2 (641.9 - 3019.2)	40.1 (16.5 - 74.1)
Streptococcus pneumoniae	Carbapenems	Liberia	1557.2 (628.5 - 3097.5)	32.4 (13.4 - 62.7)
Klebsiella pneumoniae	Trimethoprim-Sulfamethoxazole	Liberia	1544.6 (737.2 - 2609.5)	34.0 (16.4 - 56.8)
Escherichia coli	Third-generation cephalosporins	Liberia	1506.1 (567.4 - 3091.1)	35.2 (13.2 - 71.8)
Escherichia coli	Trimethoprim-Sulfamethoxazole	Liberia	1441.3 (909.6 - 2142.5)	33.6 (21.8 - 48.5)
Streptococcus pneumoniae	Fluoroquinolones	Madagascar	24729.1 (6258.0 - 50396.6)	84.1 (21.8 - 171.5)
Klebsiella pneumoniae	Third-generation cephalosporins	Madagascar	24082.2 (8368.1 - 45878.9)	88.4 (29.9 - 169.2)
Streptococcus pneumoniae	Trimethoprim-Sulfamethoxazole	Madagascar	18889.1 (2861.2 - 37525.3)	64.1 (9.9 - 129.3)
Staphylococcus aureus	Trimethoprim-Sulfamethoxazole	Madagascar	13396.5 (6733.0 - 20962.0)	55.7 (28.7 - 88.3)
Staphylococcus aureus	Methicillin	Madagascar	11479.2 (4351.6 - 21162.8)	47.7 (18.5 - 86.8)
Escherichia coli	Fluoroquinolones	Madagascar	11382.3 (7787.8 - 16146.8)	42.0 (28.7 - 58.4)
Klebsiella pneumoniae	Aminoglycosides	Madagascar	10829.0 (6506.4 - 16330.1)	39.8 (23.7 - 60.0)
Pseudomonas aeruginosa	Third-generation cephalosporins	Madagascar	10452.6 (4687.3 - 17600.7)	40.3 (17.8 - 68.8)
Streptococcus pneumoniae	Carbapenems	Madagascar	10334.3 (4367.6 - 19290.1)	35.1 (15.2 - 64.6)
Escherichia coli	Trimethoprim-Sulfamethoxazole	Madagascar	10042.7 (6767.4 - 14134.3)	37.1 (24.7 - 51.5)
Klebsiella pneumoniae	Third-generation cephalosporins	Malawi	20830.8 (6617.6 - 40023.1)	115.3 (38.3 - 220.1)
Streptococcus pneumoniae	Trimethoprim-Sulfamethoxazole	Malawi	17825.6 (2576.4 - 36275.1)	89.6 (12.9 - 178.0)
Klebsiella pneumoniae	Aminoglycosides	Malawi	9129.2 (5574.7 - 14145.1)	50.6 (31.0 - 77.0)
Streptococcus pneumoniae	Carbapenems	Malawi	8886.8 (3728.5 - 16545.4)	44.7 (19.2 - 82.5)
Staphylococcus aureus	Trimethoprim-Sulfamethoxazole	Malawi	8348.2 (4345.1 - 12917.1)	52.0 (27.2 - 80.0)
Escherichia coli	Trimethoprim-Sulfamethoxazole	Malawi	8084.2 (5373.9 - 11498.2)	48.4 (33.3 - 66.5)
Staphylococcus aureus	Methicillin	Malawi	7920.7 (3108.4 - 14357.5)	49.3 (19.9 - 88.2)
Klebsiella pneumoniae	Trimethoprim-Sulfamethoxazole	Malawi	7500.5 (3688.3 - 12185.6)	41.5 (20.5 - 68.1)
Escherichia coli	Beta Lactam/Beta-lactamase inhibitors	Malawi	5624.9 (3822.3 - 7985.1)	33.7 (23.1 - 47.5)
Escherichia coli	Third-generation cephalosporins	Malawi	5539.2 (2054.3 - 10759.7)	33.2 (12.7 - 63.1)
Klebsiella pneumoniae	Third-generation cephalosporins	Mali	55888.0 (19784.7 - 109494.0)	164.6 (58.5 - 318.2)
Staphylococcus aureus	Methicillin	Mali	29177.2 (11707.0 - 52062.7)	94.3 (38.7 - 170.1)
Streptococcus pneumoniae	Carbapenems	Mali	26518.3 (11611.9 - 50233.5)	72.8 (31.7 - 137.3)
Streptococcus pneumoniae	Trimethoprim-Sulfamethoxazole	Mali	26146.5 (3596.0 - 53405.5)	71.7 (10.1 - 149.2)
Klebsiella pneumoniae	Aminoglycosides	Mali	24231.1 (13906.4 - 37946.5)	71.4 (40.5 - 111.8)
Klebsiella pneumoniae	Fluoroquinolones	Mali	21928.3 (12834.2 - 33964.9)	64.6 (37.8 - 101.0)
Klebsiella pneumoniae	Trimethoprim-Sulfamethoxazole	Mali	20317.9 (9672.0 - 34050.0)	59.8 (28.8 - 101.8)
Group B Streptococcus	Fluoroquinolones	Mali	19163.2 (3006.2 - 41611.9)	50.1 (8.0 - 106.8)
Escherichia coli	Fluoroquinolones	Mali	18410.5 (11965.0 - 26687.4)	59.4 (39.2 - 86.2)
Staphylococcus aureus	Trimethoprim-Sulfamethoxazole	Mali	17450.2 (9028.3 - 27180.2)	56.4 (29.2 - 88.9)
Klebsiella pneumoniae	Third-generation cephalosporins	Mauritania	2495.9 (790.7 - 5034.0)	65.4 (20.4 - 130.2)
Staphylococcus aureus	Methicillin	Mauritania	2251.4 (958.9 - 3987.2)	65.9 (28.7 - 115.5)
Streptococcus pneumoniae	Trimethoprim-Sulfamethoxazole	Mauritania	1951.5 (251.5 - 4257.0)	49.2 (6.4 - 104.5)
Streptococcus pneumoniae	Carbapenems	Mauritania	1304.6 (510.6 - 2593.3)	32.9 (13.4 - 65.0)
Escherichia coli	Trimethoprim-Sulfamethoxazole	Mauritania	1036.3 (633.5 - 1555.9)	28.4 (17.5 - 42.1)
Klebsiella pneumoniae	Trimethoprim-Sulfamethoxazole	Mauritania	1035.0 (484.2 - 1774.4)	27.1 (12.8 - 46.0)
Klebsiella pneumoniae	Fluoroquinolones	Mauritania	1024.6 (578.5 - 1633.3)	26.9 (15.1 - 42.2)

Escherichia coli	Third-generation cephalosporins	Mauritania	988.6 (373.0 - 1893.1)	27.1 (10.1 - 51.7)
Klebsiella pneumoniae	Aminoglycosides	Mauritania	953.1 (516.2 - 1530.1)	25.0 (13.7 - 40.1)
Staphylococcus aureus	Trimethoprim-Sulfamethoxazole	Mauritania	858.8 (415.4 - 1372.4)	25.2 (12.4 - 39.7)
Staphylococcus aureus	Methicillin	Mauritius	991.5 (456.5 - 1753.3)	68.8 (31.5 - 121.3)
Klebsiella pneumoniae	Third-generation cephalosporins	Mauritius	283.5 (45.3 - 746.7)	20.9 (3.4 - 54.9)
Acinetobacter baumannii	Carbapenems	Mauritius	238.5 (110.1 - 450.8)	15.9 (7.5 - 29.6)
Streptococcus pneumoniae	Carbapenems	Mauritius	226.1 (98.9 - 411.9)	18.1 (7.9 - 32.7)
Enterococcus faecium	Fluoroquinolones	Mauritius	216.0 (59.3 - 436.2)	13.7 (3.8 - 27.3)
Escherichia coli	Fluoroquinolones	Mauritius	211.0 (118.4 - 335.8)	14.3 (8.1 - 22.7)
Escherichia coli	Third-generation cephalosporins	Mauritius	205.9 (77.1 - 423.2)	14.0 (5.2 - 28.9)
Acinetobacter baumannii	Fluoroquinolones	Mauritius	193.6 (87.0 - 351.9)	12.9 (5.9 - 23.6)
Pseudomonas aeruginosa	Carbapenems	Mauritius	187.9 (100.8 - 312.9)	13.3 (7.2 - 21.8)
Escherichia coli	Trimethoprim-Sulfamethoxazole	Mauritius	135.3 (75.6 - 222.4)	9.2 (5.2 - 15.0)
Klebsiella pneumoniae	Third-generation cephalosporins	Mozambique	46230.5 (16661.1 - 87268.6)	134.5 (49.1 - 250.1)
Streptococcus pneumoniae	Trimethoprim-Sulfamethoxazole	Mozambique	27513.1 (3571.1 - 56328.6)	74.5 (10.0 - 151.7)
Staphylococcus aureus	Trimethoprim-Sulfamethoxazole	Mozambique	23091.9 (12694.4 - 35552.6)	79.2 (43.6 - 120.6)
Staphylococcus aureus	Methicillin	Mozambique	22970.3 (9276.9 - 41136.6)	78.6 (32.9 - 138.7)
Streptococcus pneumoniae	Fluoroquinolones	Mozambique	20109.6 (4656.6 - 42655.4)	54.5 (12.7 - 115.1)
Klebsiella pneumoniae	Aminoglycosides	Mozambique	18468.1 (11142.4 - 28214.4)	53.8 (32.7 - 80.4)
Klebsiella pneumoniae	Trimethoprim-Sulfamethoxazole	Mozambique	15924.1 (7788.7 - 26521.2)	46.3 (22.8 - 76.7)
Klebsiella pneumoniae	Fluoroquinolones	Mozambique	14771.1 (8659.9 - 22895.5)	43.0 (25.0 - 66.9)
Streptococcus pneumoniae	Carbapenems	Mozambique	14232.3 (6078.7 - 27232.6)	38.6 (16.6 - 72.4)
Escherichia coli	Trimethoprim-Sulfamethoxazole	Mozambique	10982.1 (7193.5 - 15837.6)	35.3 (23.6 - 49.8)
Klebsiella pneumoniae	Third-generation cephalosporins	Namibia	1492.6 (379.0 - 3390.1)	70.4 (18.4 - 157.0)
Streptococcus pneumoniae	Trimethoprim-Sulfamethoxazole	Namibia	1046.7 (144.5 - 2201.1)	46.0 (6.4 - 96.8)
Staphylococcus aureus	Methicillin	Namibia	893.8 (341.2 - 1665.8)	45.7 (17.6 - 84.7)
Streptococcus pneumoniae	Carbapenems	Namibia	865.9 (362.6 - 1595.0)	38.0 (16.0 - 70.7)
Staphylococcus aureus	Trimethoprim-Sulfamethoxazole	Namibia	775.7 (378.5 - 1257.1)	39.6 (19.4 - 63.3)
Klebsiella pneumoniae	Trimethoprim-Sulfamethoxazole	Namibia	657.3 (311.3 - 1091.7)	31.0 (14.9 - 51.9)
Pseudomonas aeruginosa	Carbapenems	Namibia	593.2 (372.6 - 901.8)	29.0 (18.3 - 44.2)
Escherichia coli	Trimethoprim-Sulfamethoxazole	Namibia	589.3 (370.3 - 877.8)	29.9 (19.1 - 44.5)
Escherichia coli	Third-generation cephalosporins	Namibia	579.6 (229.0 - 1118.9)	29.4 (11.6 - 57.2)
Acinetobacter baumannii	Fluoroquinolones	Namibia	454.8 (221.3 - 796.3)	25.7 (12.7 - 44.6)
Streptococcus pneumoniae	Trimethoprim-Sulfamethoxazole	Niger	54029.0 (7286.9 - 113652.4)	133.6 (18.3 - 279.2)
Klebsiella pneumoniae	Third-generation cephalosporins	Niger	44922.3 (13643.3 - 95697.6)	122.6 (36.8 - 255.3)
Staphylococcus aureus	Methicillin	Niger	27808.3 (11936.9 - 49310.7)	87.1 (37.8 - 154.3)
Streptococcus pneumoniae	Carbapenems	Niger	26880.6 (11171.1 - 50322.9)	66.6 (27.1 - 122.9)
Streptococcus pneumoniae	Fluoroquinolones	Niger	24417.3 (5297.6 - 51911.4)	60.4 (13.1 - 127.7)
Klebsiella pneumoniae	Aminoglycosides	Niger	23238.7 (13844.0 - 35714.4)	63.5 (37.8 - 97.1)
Group B Streptococcus	Fluoroquinolones	Niger	19939.2 (3424.1 - 43342.5)	46.3 (8.0 - 100.7)
Klebsiella pneumoniae	Fluoroquinolones	Niger	19414.5 (11299.7 - 30210.1)	53.1 (30.7 - 82.9)
Klebsiella pneumoniae	Trimethoprim-Sulfamethoxazole	Niger	18571.9 (8633.0 - 30755.4)	50.7 (23.8 - 83.0)
Escherichia coli	Trimethoprim-Sulfamethoxazole	Niger	18268.7 (12434.1 - 25575.2)	53.0 (35.9 - 74.1)
Streptococcus pneumoniae	Trimethoprim-Sulfamethoxazole	Nigeria	353351.8 (52797.3 - 707507.4)	116.0 (17.1 - 230.7)
Staphylococcus aureus	Methicillin	Nigeria	326303.1 (138382.4 -	126.7 (53.5 - 211.9)
Klebsiella pneumoniae	Third-generation cephalosporins	Nigeria	294802.8 (96377.1 - 565069.3)	106.0 (34.7 - 204.1)
Escherichia coli	Third-generation cephalosporins	Nigeria	232612.2 (99032.4 - 424960.9)	87.2 (36.4 - 156.0)
Streptococcus pneumoniae	Carbapenems	Nigeria	186466.5 (84740.9 - 337068.5)	61.3 (28.1 - 110.1)
Escherichia coli	Fluoroquinolones	Nigeria	171878.2 (117609.6 -	64.4 (44.1 - 88.1)
Klebsiella pneumoniae	Aminoglycosides	Nigeria	165061.7 (102849.8 -	59.4 (37.8 - 87.9)
Streptococcus pneumoniae	Penicillin	Nigeria	163923.2 (71628.7 - 301165.4)	53.9 (23.8 - 99.0)
Group B Streptococcus	Fluoroquinolones	Nigeria	156569.3 (25371.9 - 342257.7)	49.0 (8.2 - 104.1)
Klebsiella pneumoniae	Fluoroquinolones	Nigeria	155695.6 (90987.5 - 235784.9)	56.0 (33.4 - 85.2)
Klebsiella pneumoniae	Third-generation cephalosporins	Rwanda	12449.2 (4745.5 - 22640.0)	106.3 (41.1 - 191.4)
Streptococcus pneumoniae	Trimethoprim-Sulfamethoxazole	Rwanda	8212.3 (1196.3 - 16223.7)	63.4 (9.4 - 125.1)
Staphylococcus aureus	Methicillin	Rwanda	7774.0 (3164.0 - 13804.0)	73.4 (30.1 - 128.5)
Staphylococcus aureus	Trimethoprim-Sulfamethoxazole	Rwanda	5643.2 (2932.5 - 8565.6)	53.3 (27.8 - 81.0)

Escherichia coli	Third-generation cephalosporins	Rwanda	5532.3 (2314.5 - 10168.0)	50.9 (21.3 - 93.0)
Klebsiella pneumoniae	Aminoglycosides	Rwanda	5007.7 (2971.3 - 7712.8)	42.8 (26.1 - 63.9)
Streptococcus pneumoniae	Carbapenems	Rwanda	4926.4 (2093.9 - 9596.2)	38.1 (16.3 - 72.1)
Klebsiella pneumoniae	Fluoroquinolones	Rwanda	3587.8 (2059.7 - 5545.0)	30.6 (17.9 - 46.7)
Klebsiella pneumoniae	Trimethoprim-Sulfamethoxazole	Rwanda	3579.6 (1749.2 - 6063.4)	30.6 (14.9 - 51.0)
Escherichia coli	Trimethoprim-Sulfamethoxazole	Rwanda	3558.5 (2313.2 - 5073.3)	32.7 (21.9 - 45.8)
Staphylococcus aureus	Methicillin	Sao Tome	125.3 (54.9 - 217.1)	88.4 (38.4 - 154.4)
Klebsiella pneumoniae	Third-generation cephalosporins	Sao Tome	106.9 (28.7 - 217.5)	69.3 (18.7 - 142.0)
Streptococcus pneumoniae	Trimethoprim-Sulfamethoxazole	Sao Tome	81.5 (11.0 - 165.9)	48.3 (6.4 - 95.8)
Streptococcus pneumoniae	Carbapenems	Sao Tome	61.3 (25.6 - 112.0)	36.3 (15.5 - 65.3)
Staphylococcus aureus	Trimethoprim-Sulfamethoxazole	Sao Tome	58.5 (29.4 - 94.3)	41.3 (20.9 - 64.3)
Klebsiella pneumoniae	Aminoglycosides	Sao Tome	43.7 (24.9 - 69.2)	28.3 (16.4 - 43.9)
Klebsiella pneumoniae	Fluoroquinolones	Sao Tome	42.8 (23.5 - 66.9)	27.7 (15.8 - 43.4)
Klebsiella pneumoniae	Trimethoprim-Sulfamethoxazole	Sao Tome	40.4 (19.6 - 68.9)	26.2 (12.9 - 44.3)
Escherichia coli	Trimethoprim-Sulfamethoxazole	Sao Tome	40.1 (25.4 - 58.4)	27.8 (18.0 - 40.1)
Acinetobacter baumannii	Carbapenems	Sao Tome	34.4 (17.2 - 62.2)	26.8 (13.3 - 48.7)
Streptococcus pneumoniae	Trimethoprim-Sulfamethoxazole	Senegal	10035.4 (1349.7 - 20679.1)	60.5 (8.3 - 122.5)
Staphylococcus aureus	Methicillin	Senegal	7364.6 (2932.0 - 13139.7)	52.0 (21.4 - 91.2)
Staphylococcus aureus	Trimethoprim-Sulfamethoxazole	Senegal	6866.8 (3585.8 - 10647.6)	48.5 (25.0 - 75.2)
Klebsiella pneumoniae	Third-generation cephalosporins	Senegal	6617.4 (1472.8 - 16929.6)	41.9 (9.2 - 107.0)
Klebsiella pneumoniae	Aminoglycosides	Senegal	6317.5 (3644.6 - 9840.5)	40.0 (23.1 - 62.2)
Klebsiella pneumoniae	Fluoroquinolones	Senegal	5729.2 (3431.2 - 8954.6)	36.3 (21.7 - 57.0)
Escherichia coli	Trimethoprim-Sulfamethoxazole	Senegal	5441.1 (3626.1 - 7864.1)	36.6 (24.4 - 52.7)
Streptococcus pneumoniae	Carbapenems	Senegal	5240.1 (2129.6 - 9753.6)	31.6 (13.0 - 57.3)
Klebsiella pneumoniae	Trimethoprim-Sulfamethoxazole	Senegal	5220.4 (2541.7 - 8653.9)	33.0 (16.2 - 54.3)
Escherichia coli	Third-generation cephalosporins	Senegal	4896.8 (1960.3 - 9131.4)	33.0 (13.3 - 62.3)
Staphylococcus aureus	Methicillin	Seychelles	71.5 (32.7 - 119.5)	68.5 (31.5 - 113.4)
Streptococcus pneumoniae	Carbapenems	Seychelles	27.2 (12.6 - 46.1)	27.1 (12.5 - 45.6)
Acinetobacter baumannii	Carbapenems	Seychelles	26.3 (13.2 - 46.0)	25.5 (12.9 - 44.3)
Klebsiella pneumoniae	Third-generation cephalosporins	Seychelles	24.6 (4.1 - 61.1)	24.1 (3.9 - 59.9)
Streptococcus pneumoniae	Trimethoprim-Sulfamethoxazole	Seychelles	24.4 (3.4 - 48.7)	24.3 (3.4 - 48.4)
Acinetobacter baumannii	Fluoroquinolones	Seychelles	20.8 (10.3 - 36.7)	20.2 (10.1 - 35.3)
Enterococcus faecium	Fluoroquinolones	Seychelles	16.8 (5.0 - 33.1)	15.1 (4.4 - 29.4)
Escherichia coli	Third-generation cephalosporins	Seychelles	16.7 (6.7 - 32.5)	15.8 (6.4 - 30.6)
Escherichia coli	Fluoroquinolones	Seychelles	16.6 (10.8 - 24.3)	15.7 (10.3 - 22.9)
Pseudomonas aeruginosa	Carbapenems	Seychelles	16.0 (10.1 - 23.9)	15.6 (9.8 - 23.3)
Streptococcus pneumoniae	Trimethoprim-Sulfamethoxazole	Sierra Leone	14382.2 (1647.8 - 30521.1)	134.9 (16.5 - 285.3)
Staphylococcus aureus	Methicillin	Sierra Leone	10088.8 (4169.1 - 17862.5)	112.5 (47.2 - 196.9)
Klebsiella pneumoniae	Third-generation cephalosporins	Sierra Leone	9856.2 (2568.6 - 22244.4)	99.2 (26.3 - 220.7)
Streptococcus pneumoniae	Carbapenems	Sierra Leone	7878.9 (3357.8 - 14264.3)	74.0 (31.8 - 132.4)
Staphylococcus aureus	Trimethoprim-Sulfamethoxazole	Sierra Leone	6267.0 (3214.1 - 10006.1)	69.9 (35.7 - 110.9)
Klebsiella pneumoniae	Aminoglycosides	Sierra Leone	6218.1 (3585.2 - 9836.7)	62.6 (36.5 - 98.6)
Klebsiella pneumoniae	Fluoroquinolones	Sierra Leone	5495.9 (3148.6 - 8843.5)	55.4 (31.9 - 89.2)
Klebsiella pneumoniae	Trimethoprim-Sulfamethoxazole	Sierra Leone	5099.8 (2375.1 - 8621.3)	51.3 (23.8 - 86.6)
Escherichia coli	Trimethoprim-Sulfamethoxazole	Sierra Leone	4215.0 (2791.2 - 6080.5)	45.7 (30.5 - 66.7)
Escherichia coli	Fluoroquinolones	Sierra Leone	3897.6 (2414.5 - 5825.9)	42.3 (26.4 - 62.7)
Klebsiella pneumoniae	Third-generation cephalosporins	South Africa	36131.3 (14307.6 - 65293.7)	74.0 (29.1 - 133.1)
Staphylococcus aureus	Methicillin	South Africa	32264.3 (14231.3 - 53651.8)	66.1 (29.1 - 109.0)
Streptococcus pneumoniae	Carbapenems	South Africa	24014.9 (11491.9 - 40789.3)	46.9 (22.4 - 79.2)
Streptococcus pneumoniae	Trimethoprim-Sulfamethoxazole	South Africa	18468.3 (2687.8 - 37003.6)	36.1 (5.2 - 72.1)
Klebsiella pneumoniae	Trimethoprim-Sulfamethoxazole	South Africa	14187.8 (7130.2 - 23145.3)	29.1 (14.6 - 47.2)
Pseudomonas aeruginosa	Carbapenems	South Africa	13108.4 (8624.5 - 18728.7)	27.0 (17.8 - 38.6)
Klebsiella pneumoniae	Carbapenems	South Africa	12979.2 (8635.0 - 18339.0)	26.6 (17.6 - 37.7)
Acinetobacter baumannii	Carbapenems	South Africa	12180.2 (6425.1 - 20180.2)	25.5 (13.5 - 42.4)
Acinetobacter baumannii	Fluoroquinolones	South Africa	12001.9 (6270.7 - 20052.7)	25.1 (13.2 - 41.5)
Escherichia coli	Trimethoprim-Sulfamethoxazole	South Africa	11606.2 (7821.2 - 16074.7)	24.1 (16.2 - 33.3)
Streptococcus pneumoniae	Trimethoprim-Sulfamethoxazole	South Sudan	19521.1 (2700.9 - 40985.1)	136.9 (18.9 - 288.6)

Streptococcus pneumoniae	Fluoroquinolones	South Sudan	16152.5 (3468.3 - 34020.8)	113.4 (24.6 - 234.1)
Streptococcus pneumoniae	Carbapenems	South Sudan	12745.8 (5583.7 - 22682.5)	89.6 (40.4 - 160.2)
Klebsiella pneumoniae	Third-generation cephalosporins	South Sudan	10968.8 (2710.1 - 23965.7)	85.9 (21.3 - 192.5)
Klebsiella pneumoniae	Aminoglycosides	South Sudan	6948.9 (4156.1 - 10747.2)	54.4 (32.2 - 84.3)
Group B Streptococcus	Fluoroquinolones	South Sudan	6058.9 (964.8 - 13544.5)	40.6 (6.7 - 90.1)
Acinetobacter baumannii	Anti-pseudomonal penicillin/Beta-	South Sudan	5320.7 (2817.3 - 8982.6)	55.2 (28.2 - 93.3)
Staphylococcus aureus	Trimethoprim-Sulfamethoxazole	South Sudan	5226.9 (2609.6 - 8402.7)	45.7 (23.1 - 72.1)
Escherichia coli	Trimethoprim-Sulfamethoxazole	South Sudan	4998.9 (3280.2 - 7112.2)	41.9 (28.0 - 59.1)
Klebsiella pneumoniae	Trimethoprim-Sulfamethoxazole	South Sudan	4709.7 (2114.1 - 7701.3)	36.9 (17.0 - 60.0)
Klebsiella pneumoniae	Third-generation cephalosporins	Eswatini	1252.8 (443.5 - 2468.5)	126.9 (44.4 - 250.2)
Streptococcus pneumoniae	Trimethoprim-Sulfamethoxazole	Eswatini	827.8 (119.9 - 1663.8)	73.9 (10.7 - 149.3)
Staphylococcus aureus	Trimethoprim-Sulfamethoxazole	Eswatini	685.2 (360.4 - 1076.1)	73.4 (38.0 - 115.7)
Streptococcus pneumoniae	Carbapenems	Eswatini	601.7 (256.9 - 1144.4)	53.7 (23.5 - 100.1)
Klebsiella pneumoniae	Trimethoprim-Sulfamethoxazole	Eswatini	440.7 (217.5 - 729.3)	44.6 (22.1 - 73.8)
Escherichia coli	Third-generation cephalosporins	Eswatini	386.1 (144.4 - 764.3)	42.0 (15.7 - 82.6)
Staphylococcus aureus	Methicillin	Eswatini	361.1 (133.0 - 677.2)	38.7 (14.5 - 72.7)
Escherichia coli	Trimethoprim-Sulfamethoxazole	Eswatini	326.3 (212.9 - 478.1)	35.5 (23.0 - 52.2)
Klebsiella pneumoniae	Fluoroquinolones	Eswatini	299.8 (166.5 - 469.2)	30.4 (16.8 - 48.2)
Acinetobacter baumannii	Anti-pseudomonal penicillin/Beta-	Eswatini	277.7 (130.7 - 501.3)	37.1 (17.4 - 67.5)
Streptococcus pneumoniae	Trimethoprim-Sulfamethoxazole	Tanzania	57135.9 (7677.6 - 115787.5)	77.0 (10.7 - 152.2)
Klebsiella pneumoniae	Third-generation cephalosporins	Tanzania	50643.0 (13603.6 - 111367.1)	75.0 (19.8 - 164.2)
Staphylococcus aureus	Methicillin	Tanzania	48993.7 (18805.1 - 85147.9)	81.8 (33.7 - 143.0)
Streptococcus pneumoniae	Carbapenems	Tanzania	34368.8 (14287.6 - 62165.0)	46.4 (19.4 - 81.8)
Staphylococcus aureus	Trimethoprim-Sulfamethoxazole	Tanzania	33516.9 (17029.6 - 52274.5)	56.0 (29.1 - 86.8)
Klebsiella pneumoniae	Aminoglycosides	Tanzania	29274.7 (17106.7 - 45390.4)	43.4 (26.0 - 67.5)
Klebsiella pneumoniae	Fluoroquinolones	Tanzania	25871.4 (15115.8 - 41535.5)	38.3 (22.7 - 60.7)
Escherichia coli	Third-generation cephalosporins	Tanzania	24756.5 (10224.0 - 48924.1)	42.0 (17.3 - 81.1)
Klebsiella pneumoniae	Trimethoprim-Sulfamethoxazole	Tanzania	23918.3 (11607.0 - 39985.3)	35.4 (17.1 - 58.1)
Salmonella Typhi	Fluoroquinolones	Tanzania	21668.5 (2375.6 - 55748.2)	26.2 (2.9 - 67.2)
Streptococcus pneumoniae	Trimethoprim-Sulfamethoxazole	The Gambia	1976.3 (284.3 - 4015.3)	91.3 (12.3 - 179.5)
Klebsiella pneumoniae	Third-generation cephalosporins	The Gambia	1081.0 (241.0 - 2738.6)	52.6 (11.5 - 134.5)
Staphylococcus aureus	Trimethoprim-Sulfamethoxazole	The Gambia	936.2 (504.0 - 1403.2)	53.0 (28.5 - 80.1)
Klebsiella pneumoniae	Beta Lactam/Beta-lactamase inhibitors	The Gambia	877.2 (353.7 - 1549.4)	42.6 (17.5 - 74.0)
Klebsiella pneumoniae	Aminoglycosides	The Gambia	856.8 (475.4 - 1381.4)	41.7 (23.9 - 65.4)
Streptococcus pneumoniae	Carbapenems	The Gambia	801.7 (329.7 - 1533.7)	37.0 (15.7 - 68.7)
Escherichia coli	Trimethoprim-Sulfamethoxazole	The Gambia	720.7 (476.6 - 1047.1)	38.1 (25.7 - 54.1)
Escherichia coli	Beta Lactam/Beta-lactamase inhibitors	The Gambia	638.0 (420.1 - 953.6)	33.8 (22.6 - 49.5)
Acinetobacter baumannii	Anti-pseudomonal penicillin/Beta-	The Gambia	611.6 (308.6 - 1053.5)	41.9 (20.7 - 71.8)
Klebsiella pneumoniae	Fluoroquinolones	The Gambia	608.8 (349.9 - 984.6)	29.6 (17.3 - 47.2)
Klebsiella pneumoniae	Third-generation cephalosporins	Togo	7122.0 (2108.2 - 14556.2)	93.2 (27.9 - 185.9)
Staphylococcus aureus	Methicillin	Togo	5987.8 (2495.5 - 10581.1)	88.7 (37.9 - 155.1)
Streptococcus pneumoniae	Trimethoprim-Sulfamethoxazole	Togo	5525.0 (734.8 - 11471.5)	68.2 (9.2 - 140.3)
Staphylococcus aureus	Trimethoprim-Sulfamethoxazole	Togo	3590.4 (1804.2 - 5523.8)	53.2 (27.3 - 81.7)
Klebsiella pneumoniae	Fluoroquinolones	Togo	3071.5 (1826.5 - 4895.9)	40.2 (24.1 - 63.4)
Klebsiella pneumoniae	Aminoglycosides	Togo	2962.3 (1725.7 - 4715.2)	38.8 (22.8 - 60.2)
Streptococcus pneumoniae	Carbapenems	Togo	2873.8 (1247.4 - 5535.2)	35.6 (15.6 - 67.3)
Escherichia coli	Third-generation cephalosporins	Togo	2791.0 (1080.7 - 5512.5)	36.9 (14.9 - 72.5)
Klebsiella pneumoniae	Trimethoprim-Sulfamethoxazole	Togo	2759.3 (1303.1 - 4649.5)	36.1 (17.0 - 61.0)
Escherichia coli	Trimethoprim-Sulfamethoxazole	Togo	2704.9 (1778.3 - 3919.2)	35.7 (23.7 - 52.1)
Klebsiella pneumoniae	Third-generation cephalosporins	Uganda	41789.8 (13250.3 - 83884.3)	87.4 (27.8 - 175.0)
Streptococcus pneumoniae	Trimethoprim-Sulfamethoxazole	Uganda	31687.9 (4383.6 - 67126.0)	62.4 (9.0 - 127.8)
Streptococcus pneumoniae	Carbapenems	Uganda	19897.9 (8467.1 - 35447.9)	39.3 (17.2 - 69.0)
Klebsiella pneumoniae	Aminoglycosides	Uganda	18931.5 (10659.7 - 29137.2)	39.6 (23.3 - 60.6)
Staphylococcus aureus	Trimethoprim-Sulfamethoxazole	Uganda	18330.6 (9241.5 - 29344.6)	46.4 (23.8 - 72.7)
Escherichia coli	Third-generation cephalosporins	Uganda	16466.9 (6848.3 - 32089.7)	39.3 (16.3 - 74.1)
Klebsiella pneumoniae	Fluoroquinolones	Uganda	16397.1 (9448.9 - 25684.6)	34.3 (20.3 - 52.7)
Escherichia coli	Trimethoprim-Sulfamethoxazole	Uganda	15878.7 (10255.6 - 23119.7)	37.9 (25.4 - 53.3)

Klebsiella pneumoniae	Trimethoprim-Sulfamethoxazole	Uganda	14682.5 (6961.4 - 24606.1)	30.7 (14.6 - 51.1)
Escherichia coli	Fluoroquinolones	Uganda	13176.1 (8814.5 - 18850.8)	31.5 (21.3 - 44.4)
Klebsiella pneumoniae	Third-generation cephalosporins	Zambia	16154.6 (5309.0 - 32943.8)	91.0 (29.8 - 184.3)
Streptococcus pneumoniae	Trimethoprim-Sulfamethoxazole	Zambia	14329.1 (2058.1 - 29246.8)	71.8 (10.7 - 143.4)
Staphylococcus aureus	Trimethoprim-Sulfamethoxazole	Zambia	13977.4 (7344.6 - 21683.7)	90.6 (48.0 - 140.1)
Klebsiella pneumoniae	Aminoglycosides	Zambia	10441.3 (6297.7 - 16062.1)	58.8 (36.3 - 88.2)
Escherichia coli	Carbapenems	Zambia	9485.9 (4738.5 - 16142.3)	58.5 (29.5 - 98.8)
Streptococcus pneumoniae	Carbapenems	Zambia	8282.6 (3640.9 - 15403.5)	41.6 (18.3 - 75.5)
Staphylococcus aureus	Methicillin	Zambia	7647.9 (3014.2 - 14171.8)	49.5 (19.7 - 90.3)
Escherichia coli	Trimethoprim-Sulfamethoxazole	Zambia	6849.5 (4517.0 - 10128.2)	42.3 (28.2 - 60.2)
Klebsiella pneumoniae	Trimethoprim-Sulfamethoxazole	Zambia	6559.3 (3203.2 - 10990.4)	36.9 (18.0 - 61.5)
Klebsiella pneumoniae	Fluoroquinolones	Zambia	6519.1 (3842.3 - 10374.5)	36.7 (21.6 - 57.7)
Klebsiella pneumoniae	Third-generation cephalosporins	Zimbabwe	21201.6 (7043.7 - 40918.0)	147.9 (51.2 - 281.5)
Streptococcus pneumoniae	Trimethoprim-Sulfamethoxazole	Zimbabwe	14313.5 (2099.0 - 28859.8)	91.7 (13.1 - 184.2)
Streptococcus pneumoniae	Carbapenems	Zimbabwe	10752.3 (4788.6 - 19079.0)	68.9 (30.8 - 121.8)
Staphylococcus aureus	Trimethoprim-Sulfamethoxazole	Zimbabwe	8754.2 (4624.8 - 13554.7)	67.6 (35.9 - 104.3)
Escherichia coli	Third-generation cephalosporins	Zimbabwe	7681.8 (3158.4 - 14768.8)	56.7 (23.0 - 106.7)
Klebsiella pneumoniae	Trimethoprim-Sulfamethoxazole	Zimbabwe	7194.1 (3520.0 - 12051.2)	50.2 (24.4 - 84.2)
Escherichia coli	Trimethoprim-Sulfamethoxazole	Zimbabwe	6066.4 (4145.9 - 8611.6)	44.8 (31.0 - 63.1)
Staphylococcus aureus	Methicillin	Zimbabwe	5936.8 (2223.6 - 10800.4)	45.8 (17.3 - 82.5)
Klebsiella pneumoniae	Fluoroquinolones	Zimbabwe	5199.6 (2994.5 - 8119.2)	36.3 (21.5 - 56.2)
Klebsiella pneumoniae	Aminoglycosides	Zimbabwe	4930.6 (2789.2 - 7954.2)	34.4 (19.5 - 55.2)

Supplementary Table 6. Relative risk estimates for sterile sources of specimen across 88 pathogen-drug combinations.

Pathogen	Drug	Sample size	Mean relative risk	Lower bound	Upper bound
Acinetobacter baumannii	Anti-pseudomonal penicillin/Beta-lactamase inhibitors	948	1.31	1.12	1.52
Acinetobacter baumannii	Beta-lactam/Beta-lactamase inhibitors	1555	1.27	1.11	1.44
Acinetobacter baumannii	Carbapenem	3232	1.42	1.27	1.58
Acinetobacter baumannii	Fourth-generation cephalosporins	1439	1.31	1.14	1.51
Acinetobacter baumannii	Third-generation cephalosporins	2055	1.35	1.13	1.62
Acinetobacter baumannii	Aminoglycosides	2066	1.1	0.97	1.25
Acinetobacter baumannii	Fluoroquinolones	3020	1.38	1.21	1.56
Citrobacter spp.	Aminoglycosides	4069	1.09	0.94	1.28
Citrobacter spp.	Anti-pseudomonal penicillin/Beta-lactamase inhibitors	3127	1.32	1.14	1.53
Citrobacter spp.	Carbapenem	3097	1.48	1.25	1.76
Citrobacter spp.	Fluoroquinolones	4387	1.36	1.18	1.57
Citrobacter spp.	Fourth-generation cephalosporins	2718	1.31	1.1	1.56
Citrobacter spp.	Third-generation cephalosporins	3984	1.38	1.16	1.64
Enterobacter spp.	Aminoglycosides	15211	1.19	1.06	1.34
Enterobacter spp.	Anti-pseudomonal penicillin/Beta-lactamase inhibitors	11857	1.23	1.13	1.34
Enterobacter spp.	Carbapenem	13299	1.53	1.4	1.67
Enterobacter spp.	Fluoroquinolones	17552	1.28	1.17	1.4
Enterobacter spp.	Fourth-generation cephalosporins	11482	1.31	1.18	1.45
Enterobacter spp.	Trimethoprim-Sulfamethoxazole	14798	1.09	0.98	1.21
Enterococcus faecalis	Fluoroquinolones	1126	1.43	1.24	1.64
Enterococcus faecalis	Vancomycin	36	1.7	1.39	2.07
Enterococcus faecium	Fluoroquinolones	4082	1.37	1.14	1.64
Enterococcus faecium	Vancomycin	9242	1.54	1.39	1.7
Other *Enterococci*	Fluoroquinolones	107	1.28	1.07	1.55
Other *Enterococci*	Vancomycin	7730	1.37	1.29	1.46
Escherichia coli	Aminoglycosides	164196	1.2	1.16	1.25
Escherichia coli	Aminopenicillin	157276	1.21	1.17	1.25
Escherichia coli	Beta-lactam/Beta-lactamase inhibitors	143458	1.15	1.11	1.18
Escherichia coli	Carbapenem	131382	1.7	1.5	1.93
Escherichia coli	Trimethoprim-Sulfamethoxazole	164240	1.14	1.11	1.18
Group A *Streptococcus*	Macrolide	130	1.07	0.89	1.29
Group B *Streptococcus*	Fluoroquinolones	44	1.26	1.04	1.53
Group B *Streptococcus*	Macrolide	465	1.18	0.99	1.41
Group B *Streptococcus*	Penicillin	15	1.29	1.06	1.57
Haemophilus influenzae	Aminopenicillin	1438	1.27	1.06	1.51
Haemophilus influenzae	Third-generation cephalosporins	308	1.48	1.23	1.79
Klebsiella pneumoniae	Aminoglycosides	51811	1.24	1.17	1.32
Klebsiella pneumoniae	Beta-lactam/Beta-lactamase inhibitors	46753	1.19	1.13	1.25
Klebsiella pneumoniae	Fluoroquinolones	53414	1.19	1.12	1.26
Klebsiella pneumoniae	Trimethoprim-Sulfamethoxazole	51737	1.12	1.06	1.19
Morganella spp.	Fluoroquinolones	3290	1.26	1.1	1.44
Morganella spp.	Fourth-generation cephalosporins	2352	1.23	1.02	1.49
Morganella spp.	Third-generation cephalosporins	3407	1.33	1.12	1.58
Proteus spp.	Aminoglycosides	21844	1.1	1.01	1.2
Proteus spp.	Aminopenicillin	20638	1.01	0.94	1.09
Proteus spp.	Fluoroquinolones	22141	1.13	1.05	1.21
Proteus spp.	Trimethoprim-Sulfamethoxazole	21838	1.06	0.98	1.14
Proteus spp.	Third-generation cephalosporins	18775	1.27	1.08	1.5
Pseudomonas aeruginosa	Aminoglycosides	39341	1.03	0.98	1.09
Pseudomonas aeruginosa	Anti-pseudomonal penicillin/Beta-lactamase inhibitors	36016	1.3	1.22	1.37

Pseudomonas aeruginosa	Carbapenem	41177	1.27	1.22	1.32
Pseudomonas aeruginosa	Fluoroquinolones	47417	1.19	1.15	1.23
Pseudomonas aeruginosa	Fourth-generation cephalosporins	34020	1.24	1.17	1.31
Pseudomonas aeruginosa	Third-generation cephalosporins	31041	1.35	1.15	1.59
Serratia spp.	Aminoglycosides	5250	1.05	0.93	1.19
Serratia spp.	Anti-pseudomonal penicillin/Beta-lactamase inhibitors	3003	1.17	1.01	1.35
Serratia spp.	Carbapenem	3639	1.39	1.2	1.63
Serratia spp.	Fluoroquinolones	5252	1.09	0.94	1.26
Serratia spp.	Fourth-generation cephalosporins	3928	1.17	0.99	1.38
Serratia spp.	Third-generation cephalosporins	5960	1.29	1.09	1.52
Staphylococcus aureus	Fluoroquinolones	37963	1.07	1.02	1.11
Staphylococcus aureus	Macrolide	53005	1.06	1.02	1.09
Staphylococcus aureus	Trimethoprim-Sulfamethoxazole	59632	1.17	1.09	1.25
Streptococcus pneumoniae	Beta-lactam/Beta-lactamase inhibitors	1419	1.14	0.95	1.37
Streptococcus pneumoniae	Carbapenem	1947	1.37	1.16	1.61
Streptococcus pneumoniae	Fluoroquinolones	6499	1.23	1.05	1.45
Streptococcus pneumoniae	Macrolide	7348	1.05	0.94	1.17
Streptococcus pneumoniae	Trimethoprim-Sulfamethoxazole	5413	1.14	1.01	1.28
Streptococcus pneumoniae	Third-generation cephalosporins	10457	1.33	1.13	1.57
Escherichia coli	Fluoroquinolones	171311	1.31	1.27	1.35
Escherichia coli	Third-generation cephalosporins	163801	1.37	1.17	1.61
Klebsiella pneumoniae	Carbapenem	41943	1.68	1.56	1.82
Klebsiella pneumoniae	Third-generation cephalosporins	52090	1.36	1.16	1.6
Mycobacterium tuberculosis	Extensive drug resistance	428524	2.59	2.46	2.72
Mycobacterium tuberculosis	Isoniazid mono-resistance	14537	1.19	0.84	1.67
Mycobacterium tuberculosis	Multidrug resistance	427342	2.5	1.17	4.74
Mycobacterium tuberculosis	Rifampicin mono-resistance	7161	1.39	1.06	1.77
Non-typhoidal *Salmonella*	Fluoroquinolones	42	1.23	1.01	1.5
Salmonella Paratyphi*	Fluoroquinolones	24	1.24	1.02	1.52
Salmonella Paratyphi*	Multidrug resistance	25	1.24	1.03	1.5
Salmonella Typhi	Fluoroquinolones	24	1.24	1.02	1.52
Salmonella Typhi	Multidrug resistance**	25	1.24	1.03	1.5
Shigella spp.	Fluoroquinolones***	24	1.24	1.02	1.52
Staphylococcus aureus	Methicillin	95696	1.43	1.2	1.7
Streptococcus pneumoniae	Penicillin	30849	1.27	1.18	1.36
Staphylococcus aureus	Vancomycin	53623	1.52	1.28	1.81

Sample size are the admission reported with known discharge disposition and antimicrobial susceptibility test.

* *Salmonella* Typhi estimates are used as proxy for *Salmonella* Paratyphi

** Trimethoprim-Sulfamethoxazole resistant *Salmonella* Typhi estimates are used as proxy for *Salmonella* Typhi Multidrug resistant

*** Fluoroquinolone resistant *Salmonella* Typhi estimates are used as proxy for Fluoroquinolone resistant *Shigella* spp.

Supplementary Table 7. Data points included in each primary modelling step by GBD region and the fraction of countries represented in each GBD region. Source: "*Global burden of bacterial antimicrobial resistance in 2019: a systematic analysis*"[1]

	Component 1: sepsis and infectious syndrome models*	Fraction of countries represented in component 1	Component 2: case-fatality ratio	Fraction of countries represented in component 2	Component 3: pathogen distribution	Fraction of countries represented in component 3	Component 4: fraction of resistance†	Fraction of countries represented in component 4	Component 5: relative risk	Fraction of countries represented in component 5
Andean Latin America	0	0/3	1784	2/3	12 010	2/3	538 644	3/3	4338	2/3
Australasia	320 909	1/2	94 818	1/2	6 294 677	2/2	4 653 832	2/2	5211	2/2
Caribbean	0	0/19	2858	5/19	6225	5/19	68 078	10/19	529	1/19
Central Asia	0	0/9	43 852	2/9	2785	1/9	304 341	9/9	6065	1/9
Central Europe	0	0/13	371 112	10/13	627 844	11/13	3 148 864	13/13	397 885	10/13
Central Latin America	8 130 066	2/9	3 932 601	9/9	11 641 626	8/9	829 686	9/9	20 210	5/9
Central sub-Saharan Africa	0	0/6	0	0/6	770	2/6	40 243	6/6	0	0/6
East Asia	1 189 309	1/3	385 443	2/3	257 522	2/3	2 501 536	3/3	185 980	2/3
Eastern Europe	0	0/7	118 754	4/7	64 212	5/7	968 565	7/7	102 904	4/7
Eastern sub-Saharan Africa	292	3/15	6388	4/15	68 791	9/15	474 280	14/15	3436	2/15
High-income Asia Pacific	0	0/4	135 907	3/4	99 042	3/4	18 909 332	4/4	7577	3/4
High-income North America	84 520 574	2/3	7 184 424	3/3	7 255 147	2/3	32 205 001	3/3	14 071 025	2/3
North Africa and Middle East	0	0/21	209 479	13/21	53 833	16/21	531 120	21/21	90 079	10/21
Oceania	0	0/18	0	0/18	20	1/18	4297	12/18	0	0/18
South Asia	54	1/5	77 811	4/5	51 810	4/5	1 413 840	5/5	97 131	4/5
Southeast Asia	0	0/13	195 087	9/13	91 259	8/13	3 128 014	12/13	172 947	8/13
Southern Latin America	0	0/3	200 665	3/3	73 512	2/3	740 385	3/3	5000	1/3
Southern sub-Saharan Africa	4 696 789	1/6	80 717	2/6	4 699 304	2/6	910 509	6/6	1051	1/6
Tropical Latin America	17 224 511	1/2	3 988 611	1/2	20 956 932	2/2	286 450	2/2	6443	1/2
Western Europe	10 599 906	2/24	94 506 554	20/24	105 183 184	21/24	18 909 732	21/24	932 016	21/24
Western sub-Saharan Africa	83	2/19	26 985	9/19	21 896	10/19	369 482	18/19	14 880	2/19

Total sample size and fraction of countries covered for each modelling component by GBD region. The units for sample size are deaths for sepsis and infectious syndrome models; cases for case-fatality ratios; cases, deaths, or isolates for pathogen distribution; pathogen–drug tests for fraction of resistance; and pathogen–drug tests for relative risk. Sample sizes reflect model-specific selection criteria, resulting in lower totals for the sepsis, infectious syndrome, case-fatality ratio, and pathogen distribution models in this table than those in figure 1. Totals for fraction of resistance and relative risk are higher in this table than in figure 1 because of the difference in units for certain source types, such as microbial data (isolates in figure 1, pathogen–drug tests here). Several data sources inform multiple components; therefore, data points should not be summed across a row as that will lead to duplication. More information on the data types used and the components that they inform is presented in the appendix (pp 8–15). GBD=Global Burden of Diseases, Injuries, and Risk Factors Study. *The data points listed in the sepsis and infectious syndrome models include only sources used to determine the fraction of sepsis in non-communicable diseases; maternal, neonatal, and nutritional diseases; and injuries, as well as the distribution of infectious syndromes; final estimates of the number of deaths in each infectious syndrome were generated by multiplying the fractions of sepsis and infection syndromes on GBD 2019 death estimates; GBD 2019 death estimates include 7417 sources with 28106 location-years of data for under-5 mortality and 7355 sources with over 7322 location-years of data. †For sources in the fraction of resistance modelling component, de-duplication across antibiotic resistance tests was not possible, leading to potential double counting, as seen in the high-income Asia Pacific region

1158 **Supplementary Table 8.** Overall antimicrobial resistance burden by steps in the estimation. ASMR: age-standardised mortality rate per 100 000.

Country	All-cause death counts (95% UI)	ASMR (95% UI)	Fraction of all deaths that involve infection (95% UI)	Death counts involving infection (95% UI)	ASMR involving infection (95% UI)	Fraction of deaths involving infection that are associated with resistance (95% UI)	Death counts associated with resistance (95% UI)	ASMR associated with resistance (95% UI)	Fraction of deaths involving infection that are attributable to resistance (95% UI)	Death counts attributable to resistance (95% UI)	ASMR attributable to resistance (95% UI)
Algeria	201,000 (172,000-234,000)	733.8 (639.1-839.5)	14.1% (9.7%-19.8%)	28,300 (19,000-41,500)	99.6 (67.5-143.6)	47.9% (44.7%-50.3%)	13,600 (8,790-20,300)	48.9 (31.9-72.4)	12.0% (10.5%-13.6%)	3,410 (2,180-5,180)	12.2 (7.9-18.5)
Angola	185,000 (154,000-219,000)	1204.7 (1059.6-1400.2)	48.7% (41.0%-56.8%)	90,100 (70,400-114,000)	497.6 (402.8-621.5)	25.1% (22.2%-28.7%)	22,700 (16,500-30,200)	131.6 (97.9-177.5)	5.9% (4.8%-7.7%)	5,350 (3,770-7,550)	31.8 (22.5-45.1)
Benin	93,100 (75,800-115,000)	1184.8 (1003.3-1432.6)	49.8% (41.1%-59.0%)	46,400 (35,100-61,100)	491.0 (384.6-631.9)	30.0% (26.0%-34.0%)	13,900 (10,100-18,800)	154.5 (114.8-206.3)	6.7% (5.4%-8.0%)	3,120 (2,200-4,320)	35.5 (25.5-48.2)
Botswana	21,200 (17,500-25,900)	1404.5 (1174.4-1701.7)	45.3% (37.4%-53.6%)	9,580 (7,680-12,000)	554.3 (445.9-694.3)	18.6% (15.4%-22.3%)	1,790 (1,280-2,490)	126.7 (91.3-176.6)	4.3% (3.3%-5.9%)	417 (276-615)	29.2 (19.7-41.9)
Burkina Faso	202,000 (176,000-236,000)	1323.8 (1190.0-1489.4)	52.4% (44.1%-60.9%)	106,000 (84,400-133,000)	581.3 (477.9-708.3)	28.8% (24.9%-32.4%)	30,500 (23,300-38,900)	172.5 (134.8-219.4)	6.6% (5.4%-8.0%)	6,960 (5,100-9,160)	40.5 (30.5-53.5)
Burundi	83,500 (67,500-103,000)	1287.4 (1074.9-1560.9)	53.1% (45.0%-62.0%)	44,300 (33,900-57,300)	599.6 (471.6-756.2)	25.6% (21.6%-30.4%)	11,300 (8,340-15,100)	167.0 (124.1-221.4)	6.2% (4.7%-8.9%)	2,750 (1,910-4,150)	41.9 (28.5-65.1)
Cameroon	207,000 (173,000-247,000)	1262.3 (1083.9-1514.6)	50.0% (41.7%-58.2%)	104,000 (81,500-131,000)	534.4 (421.5-670.7)	23.7% (20.2%-27.1%)	24,600 (18,300-32,500)	143.7 (107.3-191.8)	5.4% (4.4%-6.5%)	5,590 (4,010-7,520)	33.5 (24.2-45.8)
Cape Verde	3,500 (3,180-3,850)	805.4 (736.3-878.1)	25.6% (20.9%-31.6%)	895 (713-1,120)	203.5 (162.2-253.2)	35.7% (31.3%-39.6%)	321 (233-433)	74.0 (53.8-99.9)	8.8% (7.4%-10.4%)	79 (56-108)	18.2 (13.0-24.9)
Central African Republic	67,800 (54,900-84,400)	2091.2 (1712.4-2545.7)	59.8% (52.9%-66.3%)	40,500 (32,100-51,200)	1106.0 (878.8-1379.3)	23.4% (20.1%-27.3%)	9,480 (6,920-12,700)	251.3 (181.2-339.8)	5.5% (4.3%-7.2%)	2,240 (1,520-3,210)	60.2 (40.2-88.8)
Chad	157,000 (136,000-182,000)	1399.1 (1230.4-1608.1)	63.6% (56.3%-70.6%)	99,700 (82,300-121,000)	696.4 (584.7-840.4)	25.2% (21.2%-29.1%)	25,100 (19,400-31,900)	170.6 (131.6-219.3)	5.8% (4.7%-7.2%)	5,820 (4,330-7,680)	41.5 (29.7-55.6)

Comoros	5,010 (4,420-5,820)	1006.1 (904.0-1175.7)	40.6% (34.3%-47.7%)	2,030 (1,660-2,530)	389.9 (320.8-485.9)	31.6% (26.1%-37.6%)	644 (478-878)	124.8 (92.4-169.3)	8.1% (5.9%-11.7%)	165 (110-251)	32.3 (21.5-50.0)
Congo	35,700 (30,000-43,000)	1256.6 (1102.2-1465.5)	42.6% (35.1%-50.5%)	15,200 (11,900-19,400)	475.1 (377.0-599.0)	23.8% (20.8%-27.2%)	3,620 (2,630-4,880)	134.4 (97.8-180.7)	5.8% (4.8%-7.3%)	890 (620-1,250)	33.3 (23.9-46.7)
Cote d'Ivoire	182,000 (159,000-210,000)	1217.1 (1079.1-1394.5)	48.7% (39.5%-58.0%)	88,900 (70,000-111,000)	512.8 (413.4-639.1)	27.9% (23.4%-31.9%)	24,800 (18,400-32,500)	149.8 (114.7-195.4)	6.5% (5.1%-7.9%)	5,790 (4,160-7,810)	35.9 (26.2-48.3)
Democratic Republic of the Congo	564,000 (480,000-659,000)	1219.8 (1041.5-1427.9)	46.8% (38.6%-55.2%)	264,000 (208,000-332,000)	498.2 (389.9-625.2)	28.6% (25.2%-32.5%)	75,600 (57,200-97,800)	158.5 (117.5-208.4)	6.9% (5.5%-9.0%)	18,300 (13,000-25,800)	39.1 (27.5-56.0)
Equatorial Guinea	7,620 (5,830-9,810)	1156.7 (931.7-1429.8)	48.4% (37.9%-59.5%)	3,690 (2,610-5,280)	472.0 (351.9-638.2)	17.0% (12.5%-21.4%)	624 (416-913)	97.9 (66.9-139.7)	4.1% (3.0%-5.2%)	150 (99-224)	23.9 (15.9-34.9)
Eritrea	43,400 (34,100-56,600)	1364.4 (1131.2-1691.2)	50.0% (43.4%-57.4%)	21,700 (16,400-28,700)	620.8 (478.1-793.3)	30.4% (25.1%-36.1%)	6,610 (4,690-9,350)	203.8 (145.5-281.8)	7.4% (5.5%-10.5%)	1,610 (1,060-2,490)	50.2 (33.6-77.0)
Ethiopia	560,000 (506,000-622,000)	993.5 (915.0-1070.6)	49.5% (42.2%-57.3%)	277,000 (231,000-336,000)	432.0 (366.9-514.2)	30.7% (27.7%-33.9%)	85,300 (66,300-109,000)	138.1 (107.9-175.9)	7.6% (6.5%-9.2%)	21,200 (16,200-27,800)	35.3 (26.7-46.5)
Gabon	11,800 (9,890-13,600)	1110.5 (946.3-1261.7)	36.4% (29.0%-44.6%)	4,280 (3,320-5,540)	372.2 (289.9-475.8)	23.9% (20.1%-28.3%)	1,020 (739-1,410)	99.2 (71.9-136.4)	6.0% (4.8%-7.7%)	257 (179-376)	25.0 (17.5-35.8)
Ghana	208,000 (181,000-239,000)	1139.0 (1028.9-1289.2)	42.6% (35.2%-50.7%)	88,600 (70,800-109,000)	438.3 (358.1-541.9)	28.5% (24.5%-32.0%)	25,300 (19,200-33,400)	137.8 (106.5-179.7)	6.7% (5.4%-8.3%)	5,920 (4,280-8,090)	32.8 (24.2-44.3)
Guinea	114,000 (93,700-138,000)	1372.7 (1154.3-1622.2)	51.7% (43.7%-59.9%)	59,100 (45,500-75,700)	618.6 (484.0-779.8)	29.4% (25.8%-32.8%)	17,400 (12,800-23,100)	171.6 (126.3-225.9)	6.8% (5.5%-8.0%)	4,000 (2,840-5,560)	40.8 (29.1-56.3)
Guinea-Bissau	14,800 (12,400-17,900)	1484.7 (1256.2-1763.8)	50.1% (42.7%-57.6%)	7,420 (5,900-9,400)	643.8 (521.7-808.5)	24.5% (21.2%-27.9%)	1,820 (1,370-2,370)	180.2 (135.5-235.2)	5.6% (4.6%-6.7%)	413 (300-556)	41.5 (29.9-56.5)
Kenya	294,000 (268,000-322,000)	1135.2 (1047.4-1229.0)	51.1% (43.4%-58.5%)	150,000 (125,000-178,000)	522.6 (440.3-618.2)	24.8% (22.3%-27.3%)	37,300 (29,500-47,200)	148.8 (116.9-188.8)	5.7% (4.8%-6.6%)	8,540 (6,570-11,100)	34.5 (26.4-45.0)
Lesotho	32,500 (27,300-38,400)	2238.3 (1878.8-2623.6)	53.0% (44.7%-61.1%)	17,200 (13,800-21,200)	1102.5 (888.4-1350.7)	16.5% (13.5%-19.9%)	2,850 (2,050-3,880)	212.5 (152.8-292.4)	4.2% (3.1%-6.1%)	725 (469-1,110)	53.5 (35.2-80.6)

Liberia	29,700 (25,400-35,200)	1122.5 (985.0-1322.1)	48.4% (39.7%-57.8%)	14,400 (11,300-18,400)	477.1 (378.8-606.6)	23.7% (20.1%-27.5%)	3,420 (2,540-4,550)	128.3 (95.8-171.9)	5.4% (4.3%-6.5%)	772 (552-1,060)	29.5 (21.5-40.5)
Madagascar	164,000 (135,000-197,000)	1206.0 (994.7-1450.3)	49.2% (42.7%-56.3%)	80,700 (63,700-100,000)	486.7 (374.5-618.1)	28.4% (25.0%-31.9%)	22,900 (17,000-29,800)	155.4 (112.6-207.3)	6.7% (5.5%-8.3%)	5,390 (3,800-7,400)	37.1 (25.7-52.7)
Malawi	117,000 (101,000-135,000)	1203.8 (1062.6-1364.2)	51.3% (43.3%-59.9%)	59,800 (48,900-73,500)	553.6 (460.0-667.2)	26.3% (23.3%-29.1%)	15,700 (12,100-20,100)	152.7 (117.6-196.3)	6.0% (5.0%-7.2%)	3,610 (2,680-4,770)	36.0 (26.6-47.3)
Mali	201,000 (165,000-249,000)	1273.9 (1086.2-1531.2)	51.8% (42.9%-60.9%)	104,000 (78,900-137,000)	542.7 (421.9-698.1)	28.4% (25.1%-31.4%)	29,700 (21,800-39,800)	150.4 (109.0-203.1)	6.8% (5.6%-8.0%)	7,070 (5,070-9,610)	37.0 (26.3-50.9)
Mauritania	21,000 (17,600-25,800)	897.7 (792.2-1064.3)	40.3% (33.4%-48.2%)	8,470 (6,460-11,100)	327.7 (259.3-420.0)	30.7% (24.5%-35.9%)	2,600 (1,840-3,580)	106.1 (77.1-143.4)	7.2% (5.6%-8.8%)	609 (421-851)	25.2 (17.7-34.1)
Mauritius	10,700 (8,940-12,800)	678.9 (568.3-809.5)	15.4% (10.5%-21.6%)	1,650 (1,070-2,400)	108.4 (71.6-157.4)	46.7% (44.1%-49.2%)	772 (481-1,150)	49.9 (31.3-74.3)	12.3% (10.6%-14.0%)	203 (124-312)	13.1 (8.0-20.0)
Mozambique	265,000 (228,000-308,000)	1589.4 (1386.6-1847.0)	54.2% (44.3%-63.8%)	144,000 (113,000-182,000)	762.7 (616.0-959.2)	21.6% (18.5%-25.4%)	31,100 (23,700-40,700)	177.9 (134.1-235.8)	5.5% (4.2%-7.5%)	7,830 (5,430-11,200)	47.1 (31.8-70.3)
Namibia	18,900 (15,700-23,000)	1224.0 (1039.5-1457.9)	43.5% (36.6%-50.7%)	8,210 (6,470-10,400)	486.7 (386.1-610.2)	22.8% (19.7%-25.8%)	1,880 (1,370-2,550)	124.9 (92.4-168.7)	5.5% (4.3%-7.1%)	451 (306-644)	29.9 (20.6-42.0)
Niger	203,000 (166,000-249,000)	1263.8 (1077.7-1515.2)	61.3% (52.8%-69.0%)	124,000 (96,700-158,000)	596.3 (477.2-750.6)	25.2% (21.5%-29.0%)	31,300 (23,500-41,100)	161.4 (122.3-210.8)	5.7% (4.6%-6.9%)	7,090 (5,050-9,530)	38.1 (27.5-51.4)
Nigeria	1,590,000 (1,390,000-1,830,000)	1142.6 (1006.8-1285.7)	55.9% (47.6%-64.7%)	891,000 (728,000-1,100,000)	523.5 (425.5-642.3)	29.5% (26.3%-32.5%)	263,000 (203,000-336,000)	155.1 (118.2-199.9)	7.2% (6.1%-8.5%)	64,500 (48,300-83,900)	39.2 (29.0-51.7)
Rwanda	68,600 (59,700-79,500)	1017.0 (917.4-1161.5)	43.0% (35.7%-51.2%)	29,500 (23,700-36,600)	392.7 (321.8-483.6)	33.0% (29.5%-36.3%)	9,760 (7,430-12,800)	140.7 (108.1-182.7)	8.0% (6.6%-9.6%)	2,370 (1,750-3,200)	34.7 (25.5-47.2)
Sao Tome and Principe	1,010 (848-1,170)	984.7 (854.8-1081.1)	31.7% (25.7%-38.6%)	320 (241-416)	294.5 (228.4-373.4)	44.2% (41.2%-47.0%)	142 (103-189)	136.6 (101.7-178.2)	10.5% (9.0%-12.1%)	34 (24-46)	32.6 (23.8-43.7)
Senegal	89,900 (79,000-104,000)	1021.0 (923.4-1179.9)	44.1% (36.8%-52.4%)	39,700 (31,900-49,600)	396.6 (320.6-491.5)	29.8% (25.0%-34.3%)	11,900 (8,790-15,600)	126.6 (93.7-167.9)	6.8% (5.5%-8.1%)	2,700 (1,950-3,630)	29.2 (21.3-39.8)

Seychelles	797 (731-871)	806.0 (742.1-875.1)	24.2% (19.7%-30.2%)	193 (154-241)	199.8 (160.4-249.3)	38.8% (36.1%-41.5%)	75 (56-99)	77.0 (58.4-101.0)	9.8% (8.4%-11.2%)	19 (14-25)	19.3 (14.1-25.9)
Sierra Leone	70,100 (58,200-84,600)	1313.3 (1136.3-1556.8)	49.7% (40.4%-59.3%)	34,900 (26,700-45,300)	581.0 (458.4-740.4)	27.9% (23.4%-32.5%)	9,730 (7,080-13,200)	161.8 (121.2-218.0)	6.3% (5.0%-7.7%)	2,210 (1,560-3,100)	37.8 (27.5-52.1)
South Africa	522,000 (495,000-555,000)	1134.9 (1084.0-1194.8)	44.2% (36.8%-51.6%)	231,000 (188,000-278,000)	469.2 (384.4-560.3)	16.9% (14.7%-19.3%)	39,000 (29,900-50,200)	91.4 (70.0-117.7)	4.1% (3.4%-5.1%)	9,490 (7,130-12,600)	22.2 (16.6-29.6)
South Sudan	72,700 (61,000-87,500)	1206.9 (998.1-1435.9)	57.2% (48.9%-65.6%)	41,600 (33,000-52,600)	596.1 (468.7-741.8)	27.7% (24.0%-31.3%)	11,500 (8,840-15,100)	151.2 (114.5-198.6)	6.5% (5.2%-8.2%)	2,710 (1,960-3,650)	37.2 (26.1-51.9)
Eswatini	11,600 (9,450-14,100)	1676.4 (1366.6-2063.8)	48.6% (40.4%-56.8%)	5,600 (4,450-7,050)	720.4 (571.3-905.7)	20.0% (16.5%-24.0%)	1,120 (787-1,540)	170.4 (118.8-235.5)	5.3% (3.7%-7.8%)	296 (182-467)	44.3 (27.6-67.5)
Tanzania	354,000 (312,000-408,000)	1065.8 (956.2-1188.6)	47.0% (39.3%-55.4%)	167,000 (135,000-208,000)	439.6 (363.5-531.7)	32.3% (29.2%-35.6%)	54,000 (41,500-69,900)	144.2 (111.6-184.1)	7.5% (6.3%-9.1%)	12,500 (9,370-16,800)	34.5 (25.3-46.5)
The Gambia	13,500 (11,800-15,600)	1172.0 (1028.7-1340.1)	46.8% (39.3%-54.9%)	6,320 (5,070-7,880)	496.6 (404.1-615.9)	29.0% (24.5%-32.5%)	1,840 (1,390-2,380)	153.2 (116.3-198.0)	6.3% (5.1%-7.7%)	402 (295-536)	34.1 (24.7-45.9)
Togo	53,600 (45,600-63,000)	1185.7 (1042.5-1379.3)	48.6% (40.7%-56.7%)	26,000 (20,900-32,500)	503.1 (409.0-626.2)	24.1% (20.6%-27.9%)	6,280 (4,650-8,270)	141.1 (106.8-186.3)	5.7% (4.6%-7.0%)	1,480 (1,080-2,020)	33.9 (24.8-46.2)
Uganda	243,000 (213,000-280,000)	1121.6 (991.2-1254.3)	49.0% (40.4%-58.0%)	119,000 (94,700-149,000)	468.0 (383.7-574.1)	25.8% (22.8%-28.6%)	30,700 (23,300-40,100)	129.5 (98.4-166.6)	6.0% (4.8%-7.5%)	7,110 (5,070-9,660)	30.8 (21.9-42.7)
Zambia	123,000 (107,000-144,000)	1340.2 (1175.9-1552.3)	49.5% (41.2%-58.1%)	61,000 (49,200-75,600)	567.2 (462.1-702.3)	25.5% (22.8%-28.9%)	15,600 (11,800-20,600)	164.7 (121.7-218.8)	6.0% (4.9%-7.6%)	3,670 (2,600-5,080)	39.9 (28.1-55.4)
Zimbabwe	127,000 (110,000-147,000)	1505.9 (1295.0-1744.3)	49.8% (42.5%-57.2%)	62,900 (52,100-76,500)	673.0 (556.3-820.9)	25.0% (22.0%-29.4%)	15,800 (12,100-20,500)	181.5 (137.8-239.1)	6.2% (4.7%-8.8%)	3,880 (2,700-5,840)	45.2 (31.3-68.6)

1159

Supplementary Table 9. Input data by source type

Source type	Number of study-GBD location-years	Sample size	Sample size units	Estimation step used
Multiple cause of death (MCoD)	2980	120,871,372	Deaths	1. Sepsis<br>2. Infectious syndrome<br>4. Pathogen distribution
Hospital discharge	391	192,533,415	Discharges	1. Sepsis<br>2. Infectious syndrome<br>3. Case fatality rate<br>4. Pathogen distribution
Microbial or laboratory data with outcome	1448	3,038,363	Isolates	3. Case fatality rate<br>4. Pathogen distribution<br>6. Fraction of resistance<br>7. Resistance profiles<br>8. Relative risk of death<br>9. Relative length of stay
Microbial or laboratory data without outcome	3767	16,251,075	Isolates	4. Pathogen distribution<br>6. Fraction of resistance<br>7. Resistance profiles
Literature studies	811	803,175	Cases, isolates, or pathogen-drug susceptibility tests	3. Case fatality rate<br>4. Pathogen distribution<br>6. Fraction of resistance<br>8. Relative risk of death<br>9. Relative length of stay
Single drug resistance profiles	158	8,648,390	Isolates	6. Fraction of resistance
Pharmaceutical sales	1536	1,536	Study-country-years	5. Antibiotic use
Antibiotic use among children under 5 years old who reported illness	203	151,455	Households surveyed	5. Antibiotic use
Mortality surveillance (Minimally invasive tissue sampling [MITS] from Child Health and Mortality Prevention Surveillance [CHAMPS])	29	2,163	Deaths	1. Sepsis<br>2. Infectious syndrome<br>4. Pathogen distribution
Linkage (mortality only)	38	264,010	Deaths	1. Sepsis<br>2. Infectious syndrome<br>4. Pathogen distribution
**Grand Total**	**11,361**	**342,564,954**		

1163 **Supplementary Table 10.** Comparison of AMR burden by WHO region in 2019

WHO region	All-cause death counts	All-cause death rate per 100k	Fraction of all deaths that involve infection	Death counts involving infection	Death rate per 100k involving infection	Fraction of deaths involving infection that are associated with resistance	Death counts associated with resistance	Death rate per 100k associated with resistance	Fraction of deaths involving infection that are attributable to resistance	Death counts attributable to resistance	Death rate per 100k attributable to resistance
Eastern Mediterranean Region	4,120,000 (3,700,000 - 4,560,000)	567.7 (510.5 - 627.9)	26.9% (21.2% - 33.8%)	1,110,000 (855,000 - 1,430,000)	153.0 (117.8 - 196.9)	41.6% (38.4% - 44.6%)	464,000 (337,000 - 619,000)	63.9 (46.5 - 85.4)	11.1% (9.7% - 12.7%)	123,000 (87,400 - 171,000)	17.0 (12.0 - 23.5)
European Region	9,440,000 (9,100,000 - 9,790,000)	1013.6 (977.3 - 1050.5)	13.2% (9.6% - 18.0%)	1,250,000 (900,000 - 1,700,000)	133.8 (96.6 - 182.1)	43.2% (40.8% - 45.6%)	541,000 (370,000 - 763,000)	58.1 (39.7 - 81.9)	10.6% (9.4% - 11.9%)	133,000 (90,100 - 188,000)	14.3 (9.7 - 20.2)
Western Pacific Region	14,300,000 (12,900,000 - 15,700,000)	746.6 (674.6 - 819.4)	14.4% (10.4% - 19.8%)	2,070,000 (1,500,000 - 2,870,000)	107.7 (78.1 - 149.8)	43.1% (40.2% - 45.5%)	895,000 (600,000 - 1,300,000)	46.7 (31.3 - 67.8)	10.5% (9.2% - 12.1%)	219,000 (146,000 - 321,000)	11.4 (7.6 - 16.7)
Region of the Americas	7,260,000 (6,990,000 - 7,570,000)	718.7 (691.6 - 749.2)	18.3% (13.8% - 24.0%)	1,330,000 (993,000 - 1,750,000)	131.3 (98.3 - 173.3)	42.7% (40.8% - 44.5%)	569,000 (406,000 - 771,000)	56.3 (40.2 - 76.3)	10.6% (9.3% - 12.0%)	141,000 (99,900 - 196,000)	13.9 (9.9 - 19.4)
South-East Asia Region	13,400,000 (12,300,000 - 14,600,000)	667.1 (609.5 - 724.4)	30.0% (24.5% - 37.0%)	4,030,000 (3,220,000 - 5,010,000)	200.0 (159.7 - 248.8)	34.9% (29.3% - 39.7%)	1,410,000 (1,030,000 - 1,860,000)	69.9 (51.3 - 92.3)	9.7% (7.8% - 12.0%)	393,000 (273,000 - 542,000)	19.5 (13.6 - 26.9)
African Region	7,670,000 (6,930,000 - 8,570,000)	697.4 (630.0 - 779.5)	49.9% (42.3% - 58.0%)	3,830,000 (3,170,000 - 4,670,000)	348.3 (288.2 - 425.1)	27.3% (25.0% - 29.4%)	1,050,000 (829,000 - 1,320,000)	95.2 (75.4 - 119.7)	6.5% (5.6% - 7.5%)	250,000 (192,000 - 325,000)	22.7 (17.5 - 29.5)

1164

Supplementary Figure 1. Heatmap representing DALYs attributable to antimicrobial resistance (AMR) by pathogen–drug combination in the WHO African region in 2019. Abbreviations: 3GC=third-generation cephalosporins. 4GC=fourth-generation cephalosporins. Anti-pseudomonal=anti-pseudomonal penicillin or beta-lactamase inhibitors. BL-BLI=β-lactam or β-lactamase inhibitors. MDR=multidrug resistance. Mono INH=isoniazid mono-resistance. Mono RIF=rifampicin mono-resistance. NA=not applicable. Resistance to 1+=resistance to one or more drug. S Paratyphi=*Salmonella enterica* serotype Paratyphi. S Typhi=*Salmonella enterica* serotype Typhi. TMP-SMX=trimethoprim-sulfamethoxazole. XDR=extensive drug resistance.

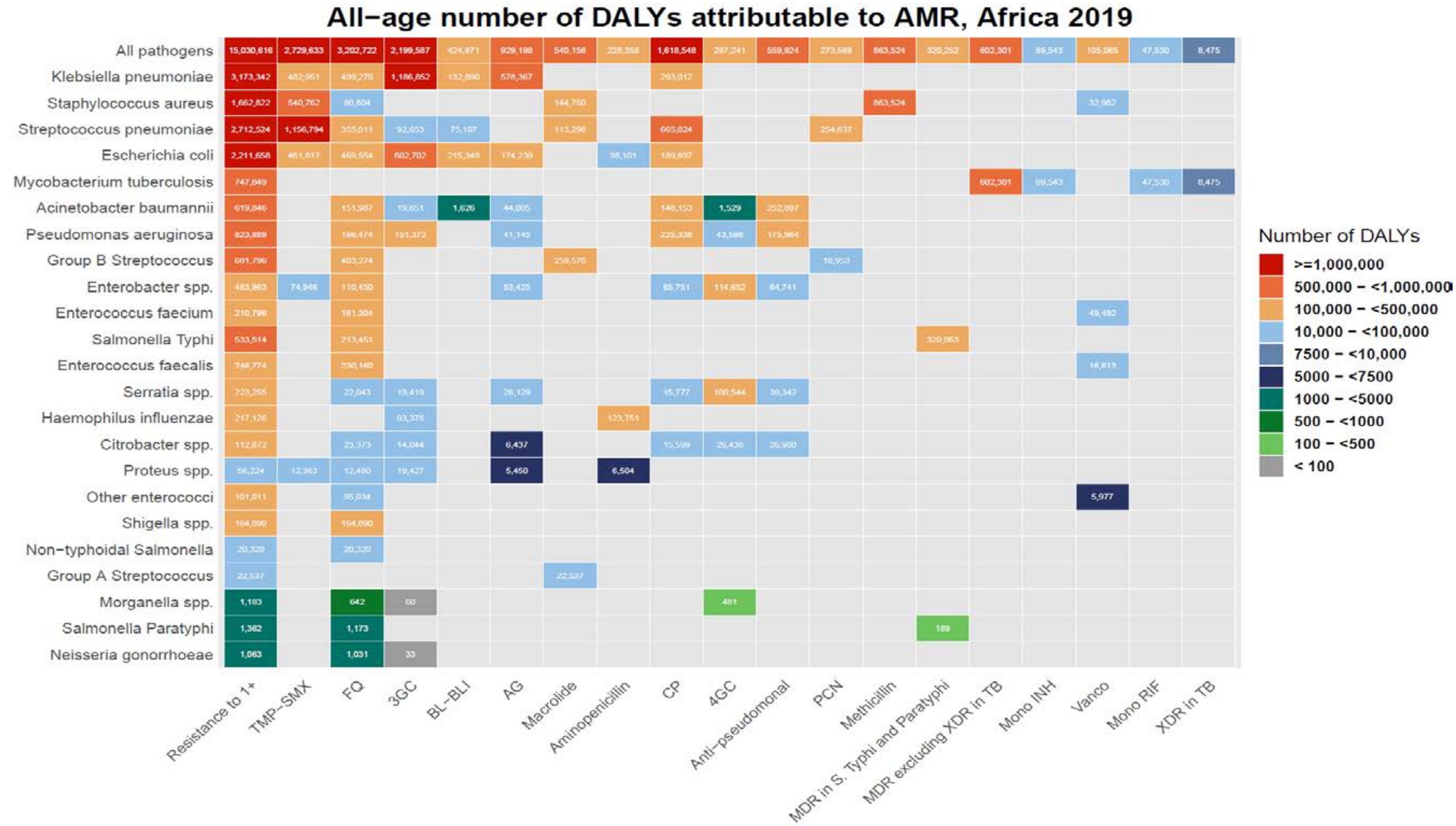

Supplementary Figure 2. Heatmap representing DALYs associated with antimicrobial resistance (AMR) by pathogen–drug combination in the WHO African region in 2019. Abbreviations: 3GC=third-generation cephalosporins. 4GC=fourth-generation cephalosporins. Anti-pseudomonal=anti-pseudomonal penicillin or beta-lactamase inhibitors. BL-BLI=β-lactam or β-lactamase inhibitors. MDR=multidrug resistance. Mono INH=isoniazid mono-resistance. Mono RIF=rifampicin mono-resistance. NA=not applicable. Resistance to 1+=resistance to one or more drug. S Paratyphi=*Salmonella enterica* serotype Paratyphi. S Typhi=*Salmonella enterica* serotype Typhi. TMP-SMX=trimethoprim-sulfamethoxazole. XDR=extensive drug resistance.

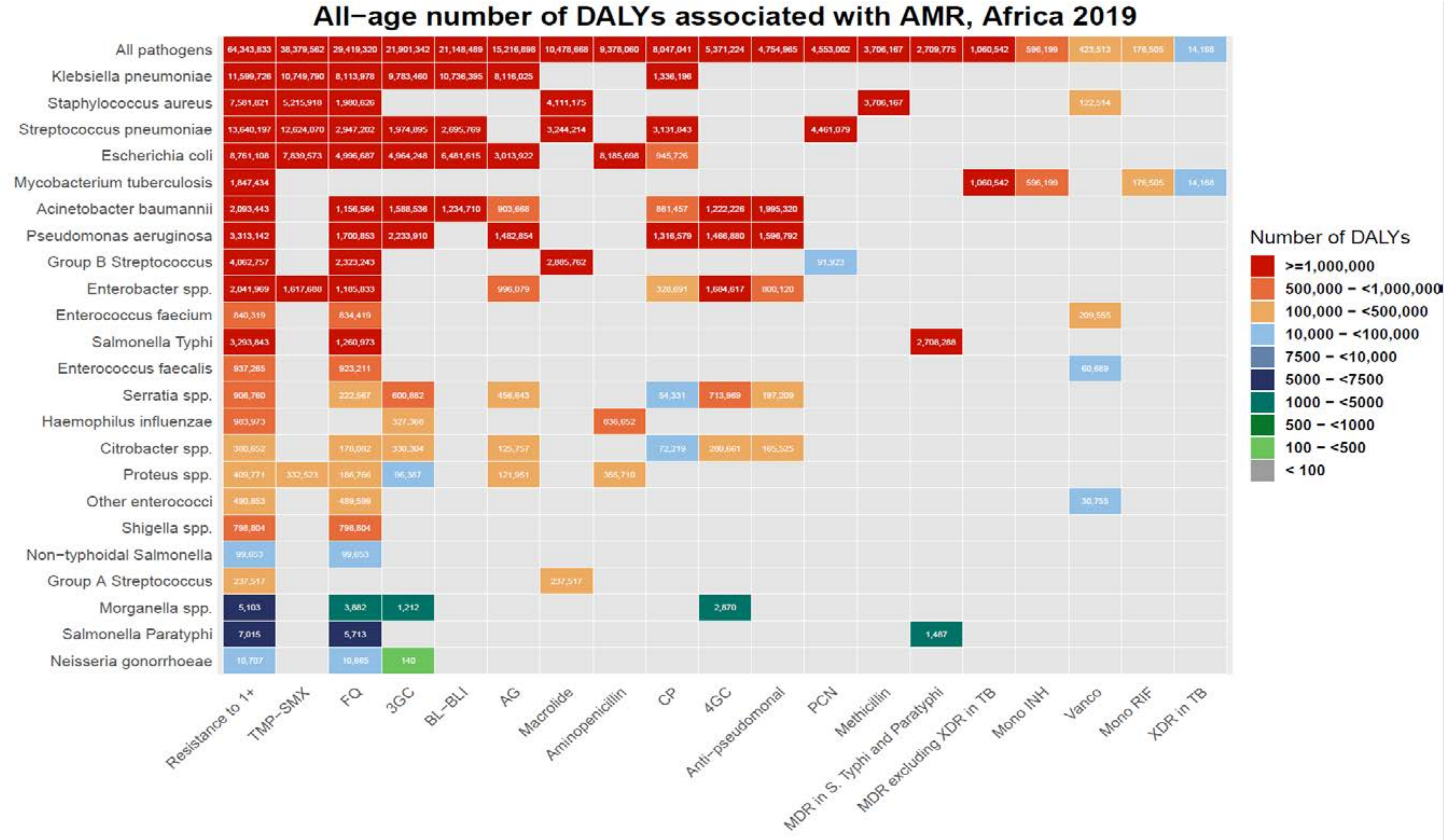

Supplementary Figure 3. Heatmap representing age-standardised DALYs per 100,000 person years associated with antimicrobial resistance (AMR) by pathogen and country for the WHO African region in 2019.

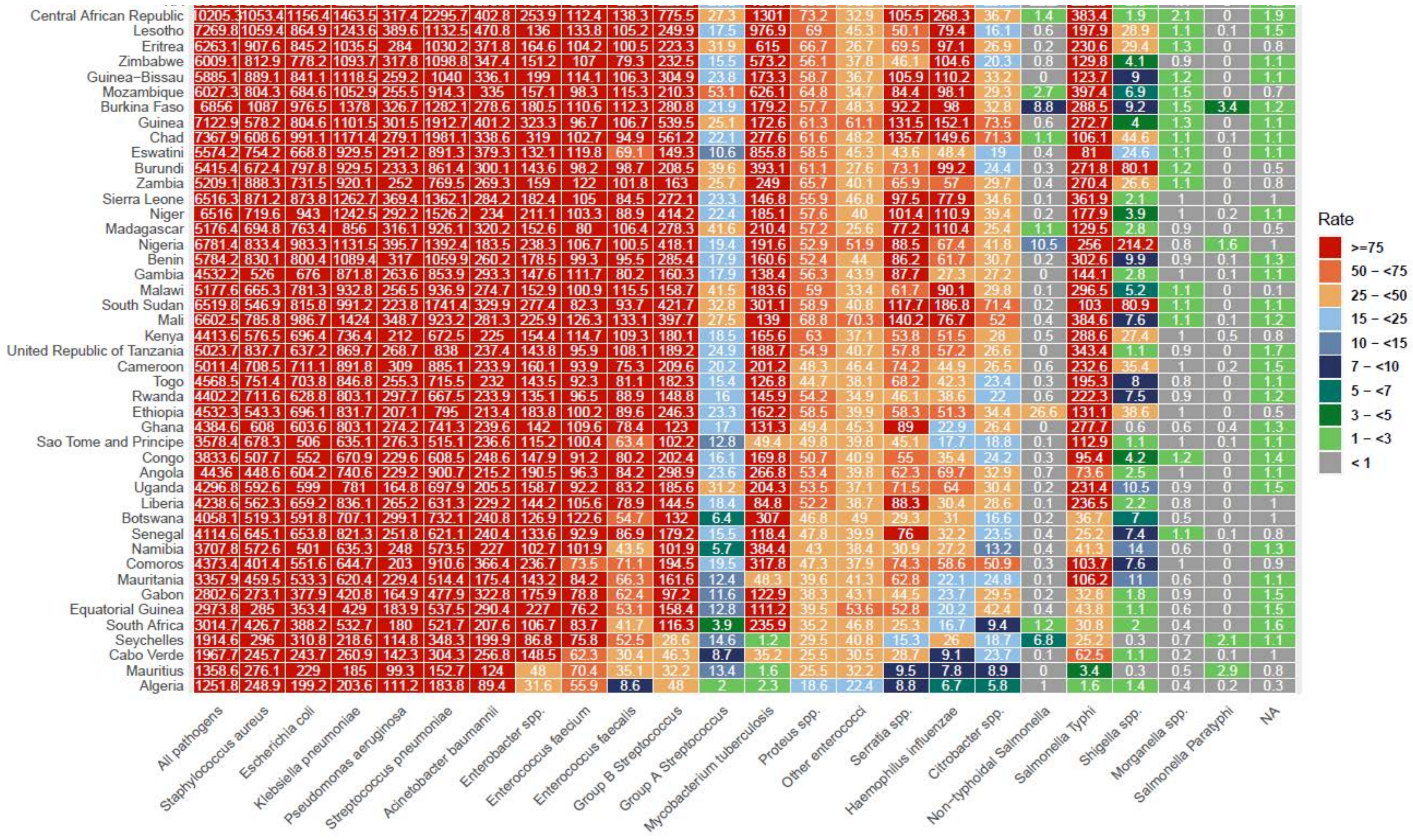

Supplementary Figure 4. Heatmap representing age-standardised DALYs per 100 000 person years attributable to antimicrobial resistance (AMR) by pathogen and country for the WHO Africa region in 2019.

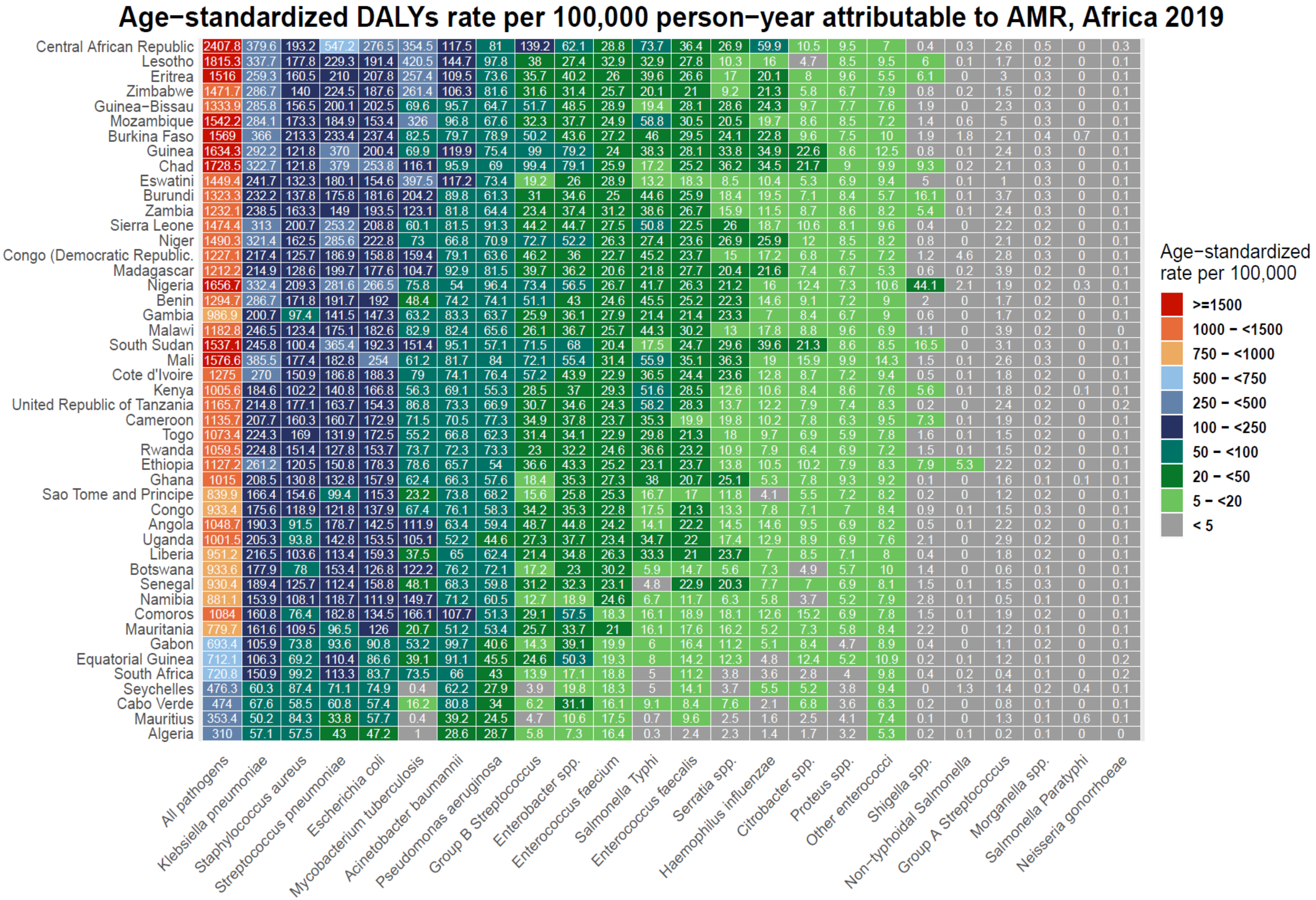

Supplementary Figure 5. Correlation between the number of deaths directly attributed to trimethoprim-sulfamethoxazole resistant *S. pneumoniae* in 2019 and number of deaths due to HIV/AIDS in 2019

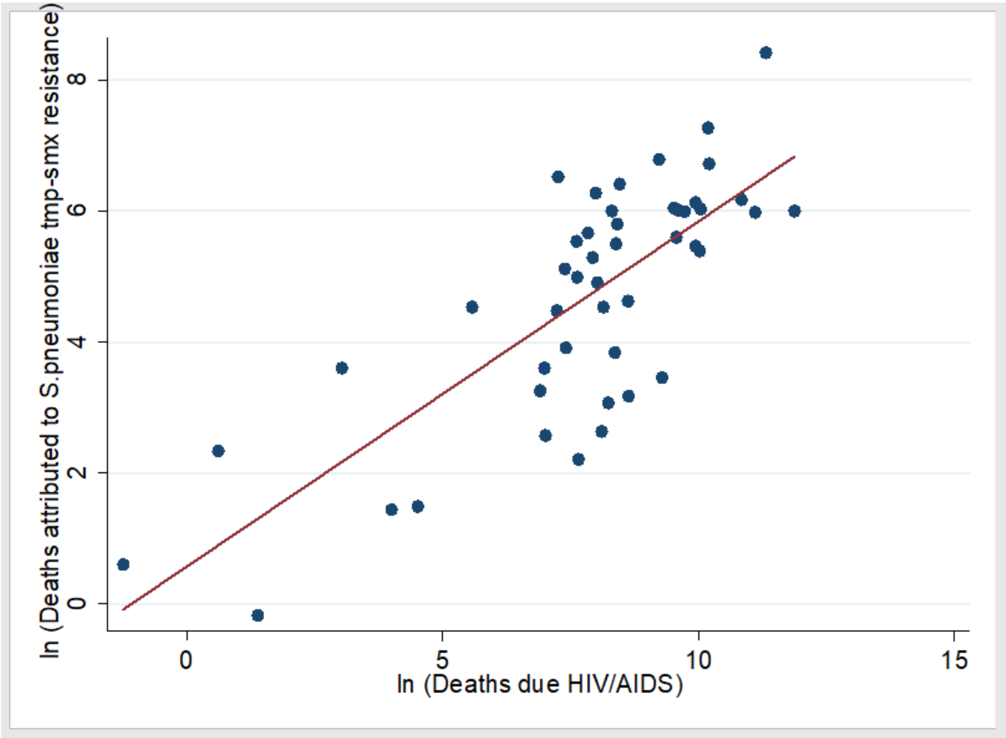

Supplementary Figure 6. Crude mortality rates associated to AMR by Socio-demographic Index (SDI) for countries in the WHO African region in 2019. Note: highlighted subregions are in accordance with GBD regions.

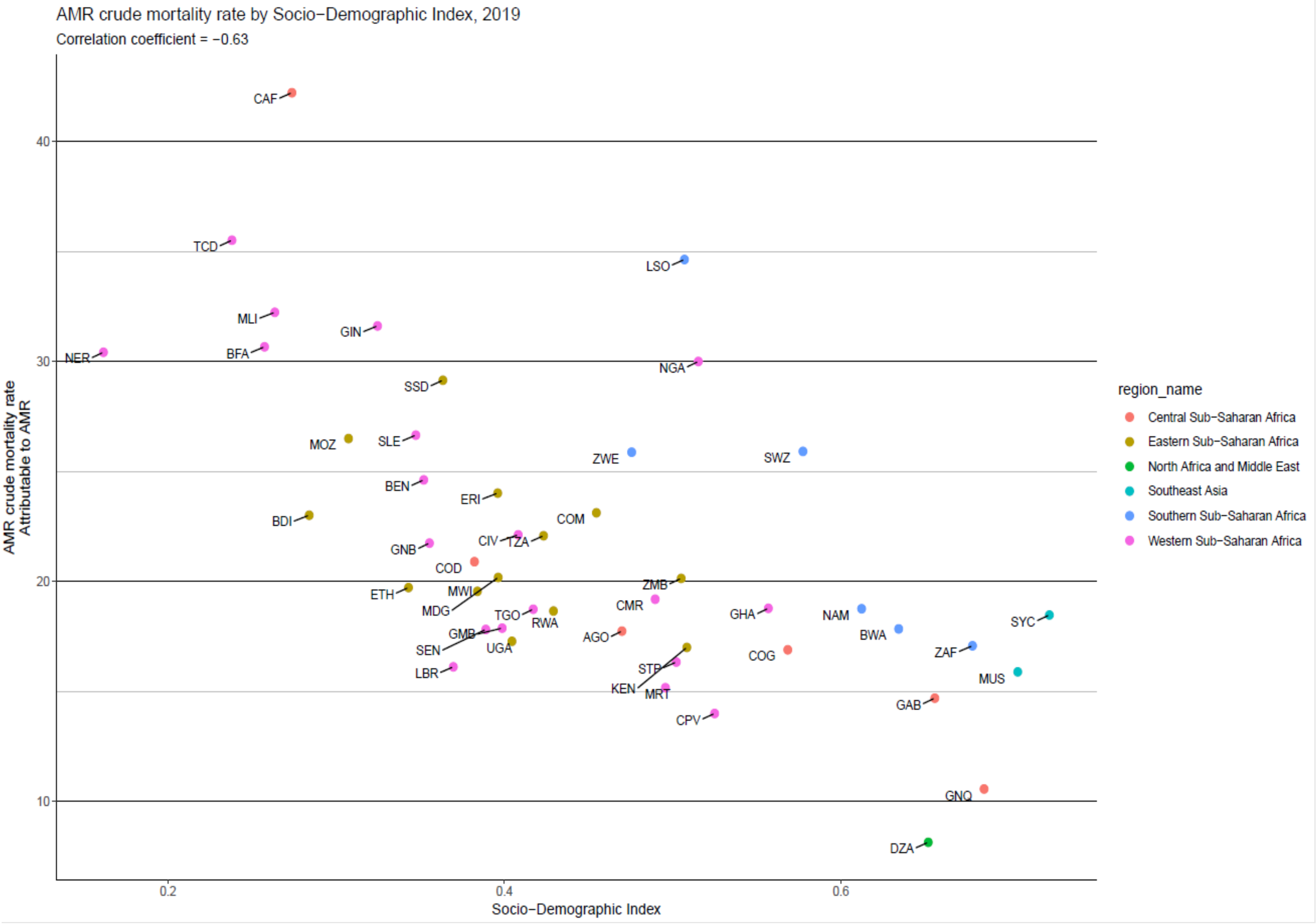

Supplementary Figure 7. Correlation between neonatal mortality associated with and attributable to AMR versus SDI [DZA: Algeria; AGO: Angola; BEN: Benin; BWA: Botswana; BFA: Burkina Faso; BDI: Burundi; CMR: Cameroon; CPV: Cape Verde; CAF: Central African Republic; TCD: Chad; COM: Comoros; COG: Congo' CIV: Cote d'Ivoire; COD: Democratic Republic of the Congo; GNQ: Equatorial Guinea; ERI: Eritrea; ETH: Ethiopia; GAB: Gabon; GHA: Ghana; GIN: Guinea; GNB: Guinea-Bissau; KEN: Kenya; LSO: Lesotho; LBR: Liberia; MDG: Madagascar; MWI: Malawi; MLI: Mali; MRT: Mauritania; MUS: Mauritius; MOZ: Mozambique; NAM: Namibia; NER: Niger; NGA: Nigeria; RWA: Rwanda; STP: Sao Tome and Principe; SEN: Senegal; SYC: Seychelles; SLE: Sierra Leone; ZAF: South Africa; SSD: South Sudan; SWZ: Swaziland; TZA: Tanzania; GMB: The Gambia; TGO: Togo; UGA: Uganda; ZMB: Zambia; ZWE: Zimbabwe]

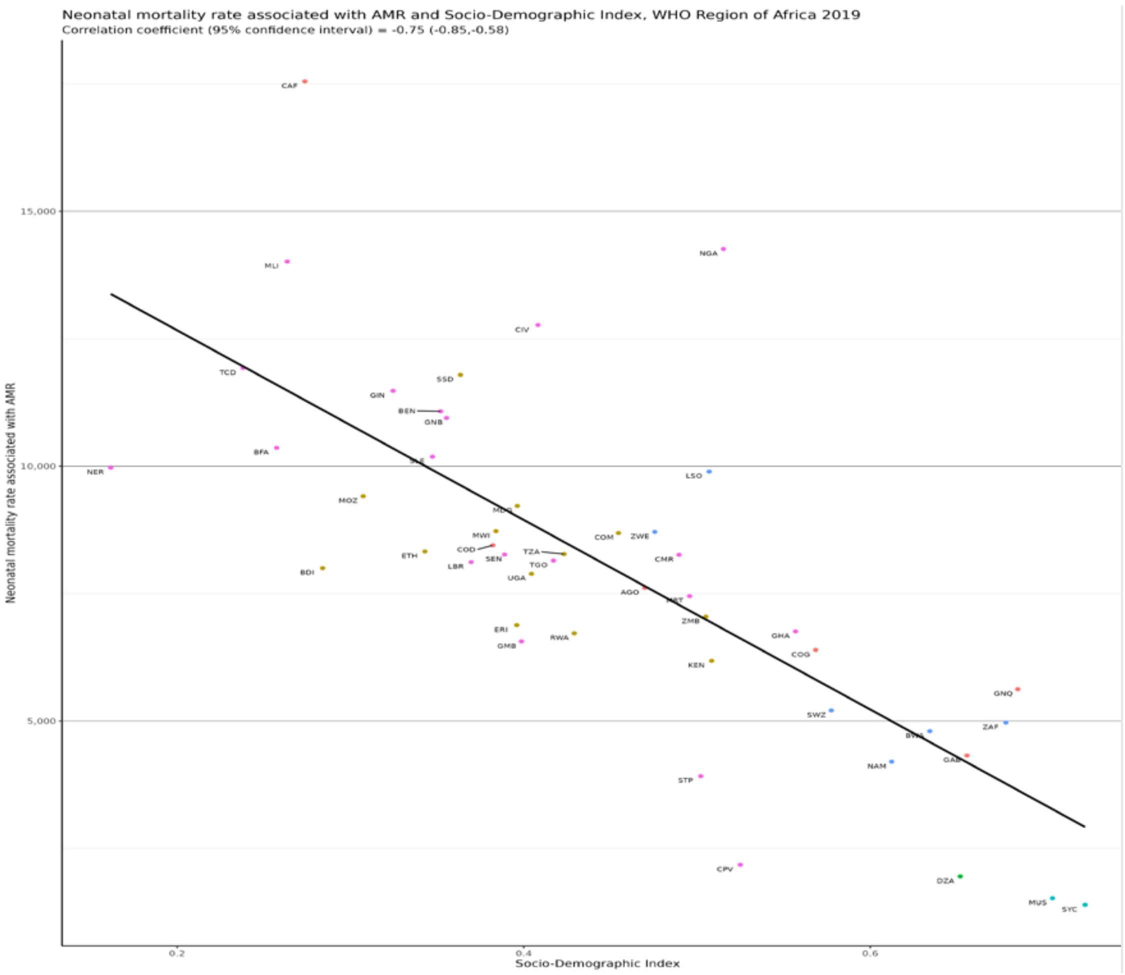

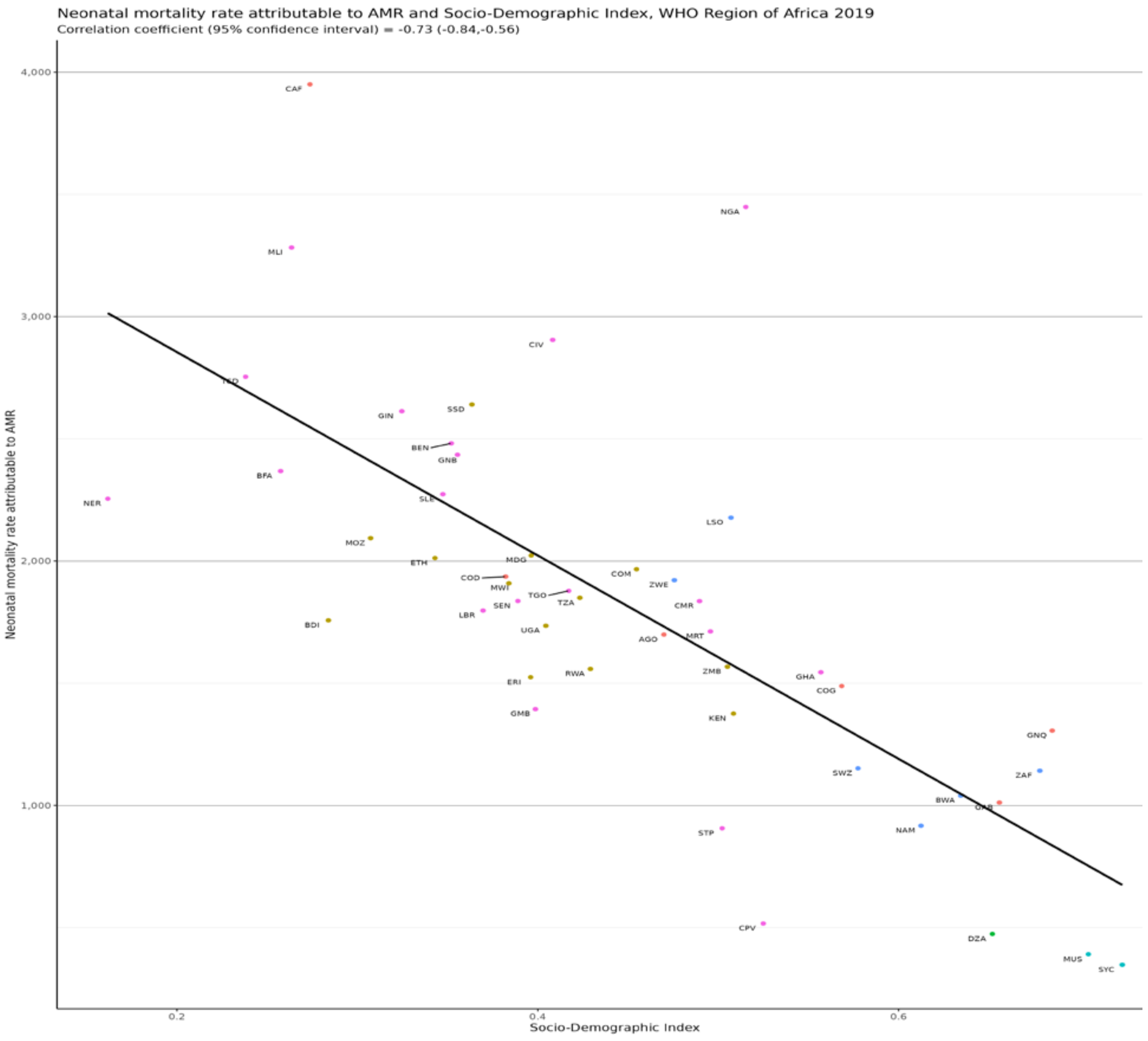

Neonatal mortality rate attributable to AMR and Socio-Demographic Index, WHO Region of Africa 2019
Correlation coefficient (95% confidence interval) = -0.73 (-0.84,-0.56)
Neonatal mortality rate attributable to AMR
Socio-Demographic Index
4,000
3,000
2,000
1,000
0.2
0.4
0.6
CAF
NGA
MLI
CIV
SSD
GIN
BEN
GNB
BFA
NER
LSO
MOZ
ETH
MDG
COD
MWI
COM
ZWE
TGO
TZA
SEN
CMR
LBR
BDI
UGA
AGO
MRT
RWA
ZMB
GHA
ERI
COG
GMB
KEN
GNQ
SWZ
ZAF
BWA
NAM
STP
CPV
DZA
MUS
SYC

Supplementary Figure 8: Correlation between AMR attributable mortality rates, DDD per 1000 people, and other key health/infrastructural indicators: SDI and water and sanitation, by country, in 2019. [DZA: Algeria; AGO: Angola; BEN: Benin; BWA: Botswana; BFA: Burkina Faso; BDI: Burundi; CMR: Cameroon; CPV: Cape Verde; CAF: Central African Republic; TCD: Chad; COM: Comoros; COG: Congo' CIV: Cote d'Ivoire; COD: Democratic Republic of the Congo; GNQ: Equatorial Guinea; ERI: Eritrea; ETH: Ethiopia; GAB: Gabon; GHA: Ghana; GIN: Guinea; GNB: Guinea-Bissau; KEN: Kenya; LSO: Lesotho; LBR: Liberia; MDG: Madagascar; MWI: Malawi; MLI: Mali; MRT: Mauritania; MUS: Mauritius; MOZ: Mozambique; NAM: Namibia; NER: Niger; NGA: Nigeria; RWA: Rwanda; STP: Sao Tome and Principe; SEN: Senegal; SYC: Seychelles; SLE: Sierra Leone; ZAF: South Africa; SSD: South Sudan; SWZ: Swaziland; TZA: Tanzania; GMB: The Gambia; TGO: Togo; UGA: Uganda; ZMB: Zambia; ZWE: Zimbabwe]

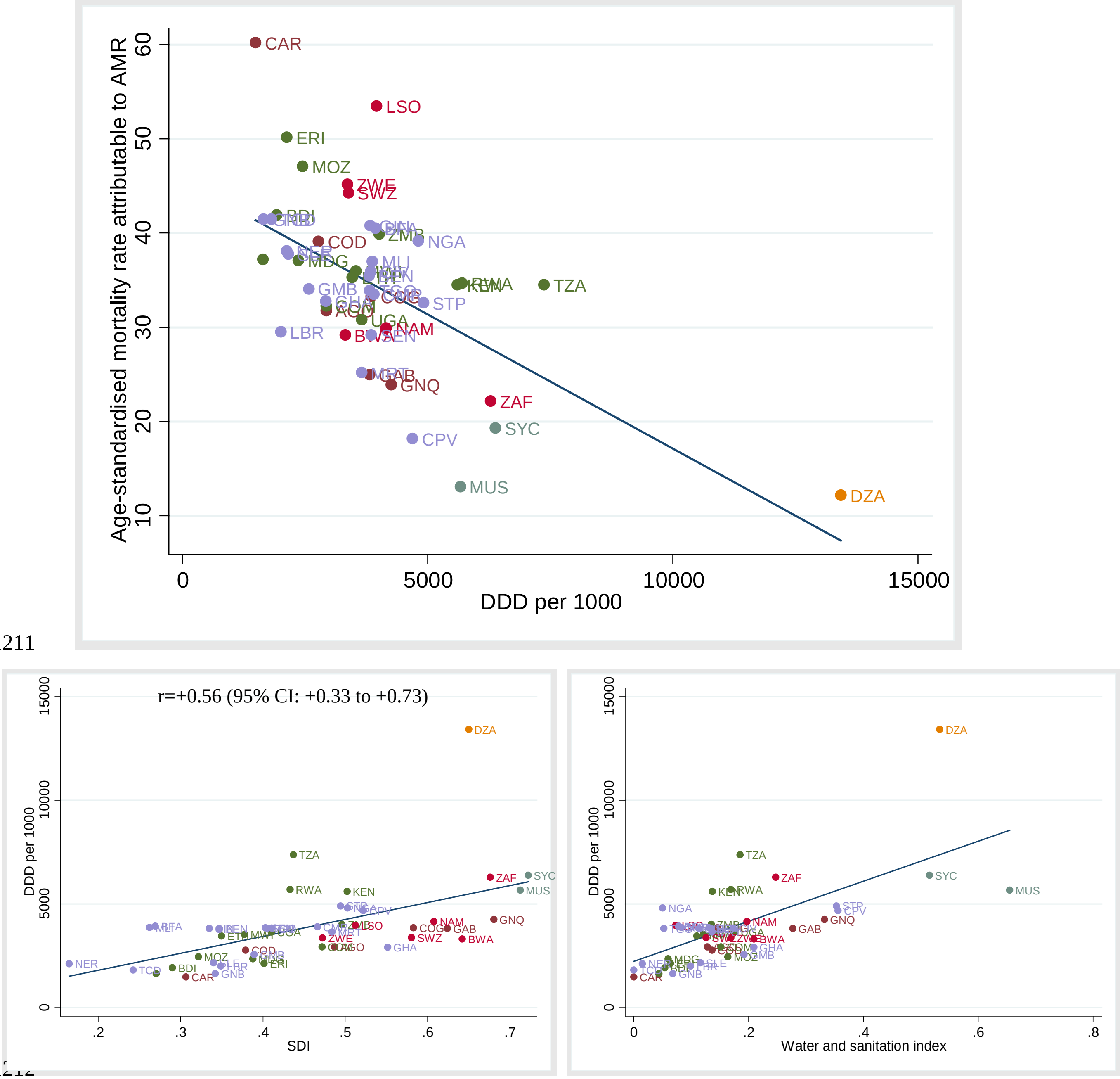

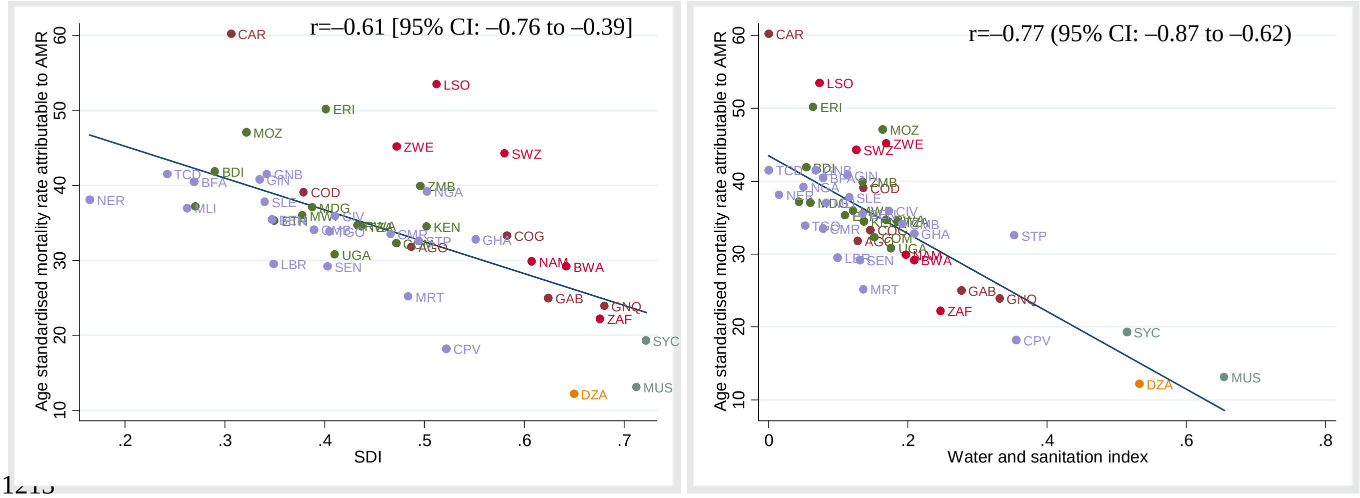
r=–0.61 [95% CI: –0.76 to –0.39]
Age standardised mortality rate attributable to AMR
SDI
10
20
30
40
50
60
.2
.3
.4
.5
.6
.7
CAR
LSO
ERI
MOZ
ZWE
SWZ
BDI
TCD
BFA
GNB
GIN
NER
MLI
SLE
COD
MDG
ZMB
NGA
KEN
COG
GHA
UGA
LBR
SEN
NAM
BWA
MRT
GAB
GNQ
ZAF
SYC
CPV
DZA
MUS
r=–0.77 (95% CI: –0.87 to –0.62)
Age standardised mortality rate attributable to AMR
Water and sanitation index
0
.2
.4
.6
.8
CAR
LSO
ERI
MOZ
SWZ
ZWE
COD
STP
GHA
LBR
SEN
MRT
GAB
GNQ
ZAF
SYC
CPV
DZA
MUS

Supplementary Figure 9: Age−specific mortality rates per 100 000 person-years for deaths attributable to and deaths associated with AMR per country in the WHO Region of Africa, 2019. Rates for males are shown on the left-hand side of each plot, while rates for females are shown on the right.

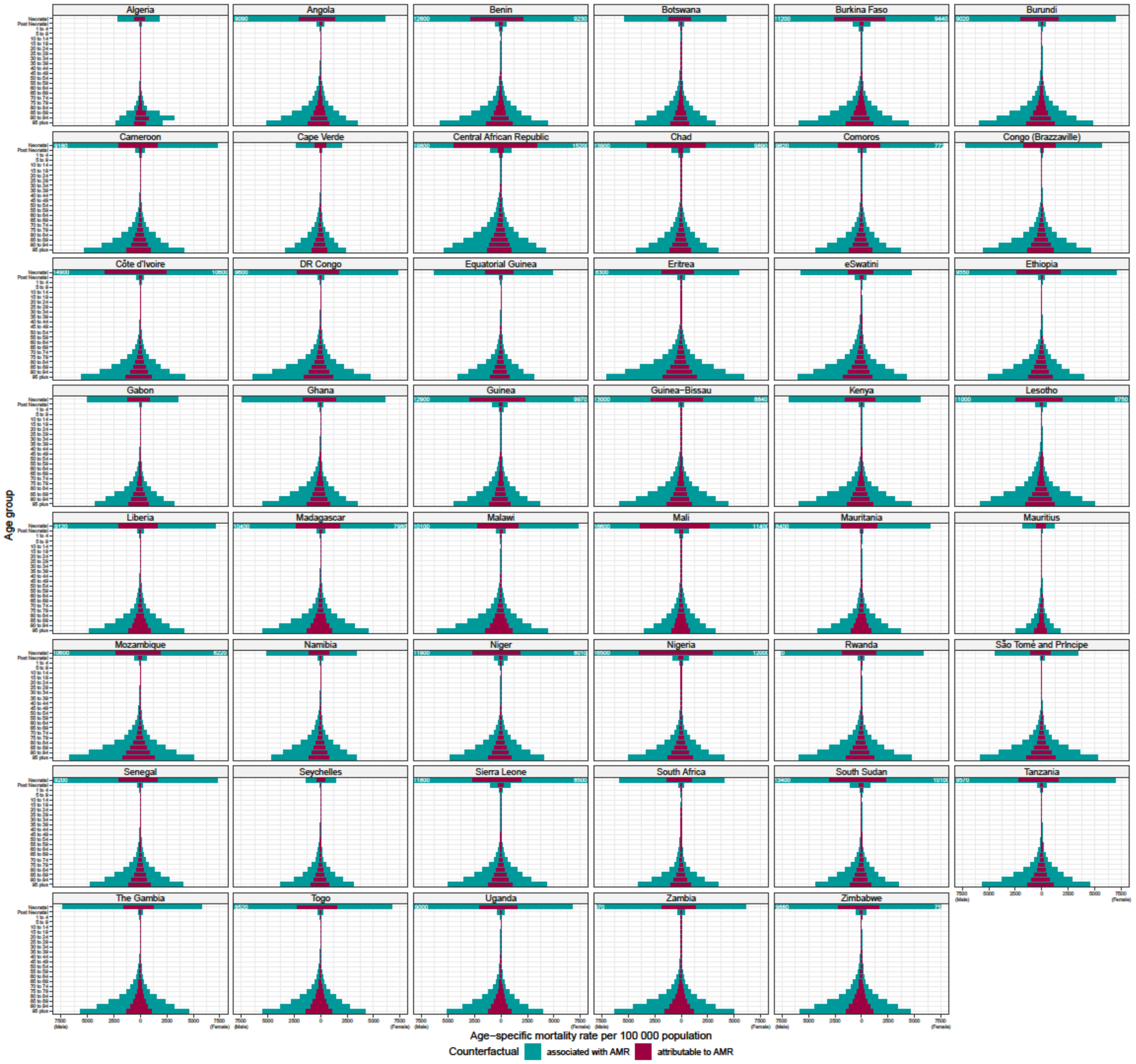

Section 5: GATHER Compliance: Guidelines for Accurate and Transparent Health Estimates Reporting

This study complies with GATHER recommendations. We have documented the steps in our analytical procedures and detailed the data sources used. The GATHER recommendations can be found on the GATHER website.

Checklist of information that should be included in new reports of global health estimates

Item #	Checklist item	Reported on page #
**Objectives and funding**		
**1**	Define the indicator(s), populations (including age, sex, and geographic entities), and time period(s) for which estimates were made.	Main text methods section (overview and input data)
**2**	List the funding sources for the work.	Main text abstract section (funding statement) and acknowledgements section
**Data Inputs**		
For all data inputs from multiple sources that are synthesized as part of the study:		
**3**	Describe how the data were identified and how the data were accessed.	Main text methods section and supplementary appendix (sections 2, 3.2.1, 3.3.1, 3.4.1, 3.5.1, and 3.6.1)
**4**	Specify the inclusion and exclusion criteria. Identify all ad-hoc exclusions.	Supplementary appendix (section 2)
**5**	Provide information on all included data sources and their main characteristics. For each data source used, report reference information or contact name/institution, population represented, data collection method, year(s) of data collection, sex and age range, diagnostic criteria or measurement method, and sample size, as relevant.	Supplementary appendix (section 2) and https://ghdx.healthdata.org/gbd-2019/data-input-sources

**6**	Identify and describe any categories of input data that have potentially important biases (e.g., based on characteristics listed in item 5).	Main text limitations section and supplementary appendix (biases for input data in each modelling step identified in each section)
For data inputs that contribute to the analysis but were not synthesized as part of the study:		
**7**	Describe and give sources for any other data inputs.	GBD 2019 estimates<br>(https://ghdx.healthdata.org/gbd-results-tool)
For all data inputs:		
**8**	Provide all data inputs in a file format from which data can be efficiently extracted (e.g., a spreadsheet rather than a PDF), including all relevant meta-data listed in item 5. For any data inputs that cannot be shared because of ethical or legal reasons, such as third-party ownership, provide a contact name or the name of the institution that retains the right to the data.	Data inputs and/or contact information available at<br>(https://ghdx.healthdata.org/gbd-2019/data-input-sources)
**Data analysis**		
**9**	Provide a conceptual overview of the data analysis method. A diagram may be helpful.	Main text methods section
**10**	Provide a detailed description of all steps of the analysis, including mathematical formulae. This description should cover, as relevant, data cleaning, data pre-processing, data adjustments and weighting of data sources, and mathematical or statistical model(s).	Supplementary appendix (section 3)
**11**	Describe how candidate models were evaluated and how the final model(s) were selected.	Supplementary appendix (section 3)
**12**	Provide the results of an evaluation of model performance, if done, as well as the results of any relevant sensitivity analysis.	Supplementary appendix (section 3.5.3)
**13**	Describe methods for calculating uncertainty of the estimates. State which sources of uncertainty were, and were not, accounted for in the uncertainty analysis.	Main text methods section (modelling tools and framework and uncertainty analysis), main text limitations section, and supplementary

		appendix (section 3)
**14**	State how analytic or statistical source code used to generate estimates can be accessed.	Main text methods section (link to GitHub code will be available at the time of publication)
**Results and Discussion**		
**15**	Provide published estimates in a file format from which data can be efficiently extracted.	Main text results section. CSV files are available upon request to the corresponding author
**16**	Report a quantitative measure of the uncertainty of the estimates (e.g. uncertainty intervals).	Uncertainty intervals are provided for all estimates throughout the main text (summary, results, and discussion sections)
**17**	Interpret results in light of existing evidence. If updating a previous set of estimates, describe the reasons for changes in estimates.	Main text (research in context, introduction, and discussion sections)
**18**	Discuss limitations of the estimates. Include a discussion of any modelling assumptions or data limitations that affect interpretation of the estimates.	Main text limitations section and supplementary appendix (section 3)

This checklist should be used in conjunction with the GATHER statement and Explanation and Elaboration document, found on gather-statement.org

Section 6: References

1 Antimicrobial Resistance Collaborators. Global burden of bacterial antimicrobial resistance in 2019: a systematic analysis. *The Lancet* 2022; **399**: 629–55.

2 GBD 2019 Diseases and Injuries Collaborators. Global burden of 369 diseases and injuries in 204 countries and territories, 1990–2019: a systematic analysis for the Global Burden of Disease Study 2019. The Lancet 2020; 396(10258): 1204-22.

3 GBD 2019 Demographics Collaborators. Global age-sex-specific fertility, mortality, healthy life expectancy (HALE), and population estimates in 204 countries and territories, 1950–2019: a comprehensive demographic analysis for the Global Burden of Disease Study 2019. *The Lancet* 2020; published online Oct 17. DOI:doi:10.1016/S0140-6736(20)30977-6.

4 Rudd KE, Johnson SC, Agesa KM, *et al.* Global, regional, and national sepsis incidence and mortality, 1990–2017: analysis for the Global Burden of Disease Study. *The Lancet* 2020; **395**: 200–11.

5 GBD 2019 Diseases and Injuries Collaborators. Global burden of 369 diseases and injuries in 204 countries and territories, 1990–2019: a systematic analysis for the Global Burden of Disease Study 2019. *The Lancet* 2020; **396**: 1204–22.

6 Zheng P, Barber R, Sorensen RJD, Murray CJL, Aravkin AY. Trimmed constrained mixed effects models: formulations and algorithms. *J Comput Graph Stat* 2021; **30**: 544–56.

7 Ewig S, Schlochtermeier M, Göke N, Niederman MS. Applying sputum as a diagnostic tool in pneumonia: limited yield, minimal impact on treatment decisions. *Chest* 2002; **121**: 1486–92.

8 Ogawa H, Kitsios GD, Iwata M, Terasawa T. Sputum gram stain for bacterial pathogen diagnosis in community-acquired pneumonia: a systematic review and bayesian meta-analysis of diagnostic accuracy and yield. *Clin Infect Dis Off Publ Infect Dis Soc Am* 2020; **71**: 499–513.

9 Feikin DR, Scott JAG, Gessner BD. Use of vaccines as probes to define disease burden. *Lancet Lond Engl* 2014; **383**: 1762–70.

10 GBD 2016 Lower Respiratory Infections Collaborators. Estimates of the global, regional, and national morbidity, mortality, and aetiologies of lower respiratory infections in 195 countries, 1990-2016: a systematic analysis for the Global Burden of Disease Study 2016. *Lancet Infect Dis* 2018; **18**: 1191–210.

11 Johnson HL, Deloria-Knoll M, Levine OS, *et al.* Systematic evaluation of serotypes causing invasive pneumococcal disease among children under five: The Pneumococcal Global Serotype Project. *PLOS Med* 2010; **7**: e1000348.

12 Grijalva CG, Nuorti JP, Arbogast PG, Martin SW, Edwards KM, Griffin MR. Decline in pneumonia admissions after routine childhood immunisation with pneumococcal conjugate vaccine in the USA: a time-series analysis. *Lancet Lond Engl* 2007; **369**: 1179–86.

13 Nocedal J, Wright S. Numerical Optimization, 2nd edn. New York: Springer-Verlag, 2006 https://link.springer.com/book/10.1007/978-0-387-40065-5 (accessed Aug 8, 2022).

14 GBD Diarrhoeal Diseases Collaborators. Estimates of global, regional, and national morbidity, mortality, and aetiologies of diarrhoeal diseases: a systematic analysis for the Global Burden of Disease Study 2015. *Lancet Infect Dis* 2017; **17**: 909–48.

15 Moher D, Liberati A, Tetzlaff J, Altman DG, PRISMA Group. Preferred reporting items for systematic reviews and meta-analyses: the PRISMA statement. *PLoS Med* 2009; **6**: e1000097.

16 Browne AJ, Kashef Hamadani BH, Kumaran EAP, et al. Drug-resistant enteric fever worldwide, 1990 to 2018: a systematic review and meta-analysis. *BMC Med* 2020; **18**.

17 Jamison DT, Breman JG, Measham AR, *et al.*, editors. Disease control priorities in developing countries, 2nd edn. Washington (DC): World Bank, 2006 http://www.ncbi.nlm.nih.gov/books/NBK11728/ (accessed Aug 8, 2022).

18 Cassini A, Högberg LD, Plachouras D, *et al.* Attributable deaths and disability-adjusted life-years caused by infections with antibiotic-resistant bacteria in the EU and the European Economic Area in 2015: a population-level modelling analysis. *Lancet Infect Dis* 2019; **19**: 56–66.

19 Rajendran NB, Mutters NT, Marasca G, et al. Mandatory surveillance and outbreaks reporting of the WHO priority pathogens for research & discovery of new antibiotics in European countries. *Clin Microbiol Infect Off Publ Eur Soc Clin Microbiol Infect Dis* 2020; **26**: 943.e1-943.e6.

20 Global Burden of Disease Collaborative Network. Socio-Demographic Index (SDI) 1950–2019. Global Burden of Disease Study 2019 (GBD 2019). Seattle, United States of America: Institute for Health Metrics and Evaluation (IHME); 2020. .

21 Browne AJ, Chipeta MG, Haines-Woodhouse G, et al. Global antibiotic consumption and usage in humans, 2000–18: a spatial modelling study. The Lancet Planetary Health 2021; 5(12): e893-e904. .

22 WHO. Global Database for Tracking Antimicrobial Resistance (AMR) Country Self- Assessment Survey (TrACSS). https://amrcountryprogress.org/#/visualization-view (accessed Feb 21, 2023).

Authors' Contributions

Managing the overall research enterprise

Benn Sartorius, Eve E Wool, Simon I Hay, Christiane Dolecek, Christopher J L Murray, Mohsen Naghavi

Writing the first draft of the manuscript

Benn Sartorius, Authia P Gray, Nicole Davis Weaver

Primary responsibility for applying analytical methods to produce estimates

Authia P Gray, Gisela Robles Aguilar, Lucien R Swetschinski, Kevin S Ikuta, Tomislav Mestrovic, Erin Chung, Chieh Han, Mohsen Naghavi

Primary responsibility for seeking, cataloguing, extracting, or cleaning data; designing or coding figures and tables

Authia P Gray, Gisela Robles Aguilar, Lucien R Swetschinski, Chieh Han, Anna Gershberg Hayoon, Mohsen Naghavi

Providing data or critical feedback on data sources

Richard Gyan Aboagye, Ayman Ahmed, Wuraola Akande-Sholabi, Ganiyu Adeniyi Amusa, Daniel T Araki, Ayele Mamo Argaw, Ahmed Y Azzam, Francois-Xavier Babin, Indrajit Banerjee, Nebiyou Simegnew Bayileyegn, Melaku Ashagrie Belete, James A Berkley, Julia A Bielicki, Denise Dekker, Christiane Dolecek, Susanna J Dunachie, Michael Ekholuenetale, Temitope Cyrus Ekundayo, Iman El Sayed, Nicholas A Feasey, Frederick Fell, Karen M Forrest, Georgina Haines-Woodhouse, Chieh Han, Ahmed I. Hasaballah, Andrea Haekyung Haselbeck, Yingfen Hsia, Kevin S Ikuta, Arnaud Iradukunda, Kenneth Chukwuemeka Iregbu, Chidozie C D Iwu, Assefa N Iyasu, Fatoumatta Jaiteh, Hyon Jin Jeon, Charity Ehimwenma Joshua, Gebrehiwot G Kassa, Ralf Krumkamp, Emmanuelle A P Kumaran, Hmwe Hmwe Kyu, Florian Marks, Barney McManigal, Tomislav Mestrovic, Mustapha Mohammed, Shafiu Mohammed, Ali H Mokdad, Catrin E Moore, Neema Mturi, Christopher J L Murray, Patrick Musicha, Lillian A Musila, Mohsen Naghavi, Dooshanveer C. Nuckchady, Ismail A. Odetokun, Oluwaseun Adeolu Ogundijo, Osaretin Christabel Okonji, Andrew T Olagunju, Gi Deok Pak, Olga Perovic, Andrew Pollard, Mathieu Raad, Clotaire Donatien Rafaï, Elrashdy Moustafa Mohamed Redwan, Gisela Robles Aguilar, Anna Roca, Victor Daniel Rosenthal, Abdallah M Samy, M Sharland, Aminu Shittu, Andy Stergachis, Lucien R Swetschinski, Caroline Tigoi, Timothy Walsh, and Judd L Walson.

Developing methods or computational machinery

Ahmed Y Azzam, Indrajit Banerjee, Julia A Bielicki, Adeniyi Francis Fagbamigbe, Frederick Fell, Authia P Gray, Georgina Haines-Woodhouse, Simon I Hay, Yingfen Hsia, Kevin S Ikuta, Emmanuelle A P Kumaran, Christopher J L Murray, Mohsen Naghavi, Andrew T Olagunju, Gisela Robles Aguilar, Abdallah M Samy, Benn Sartorius, M Sharland, and Lucien R Swetschinski.

Providing critical feedback on methods or results

Sherief Abd-Elsalam, Richard Gyan Aboagye, Lawan Hassan Adamu, Abiola Victor Adepoju, Ayman Ahmed, Gizachew Taddesse Akalu, Wuraola Akande-Sholabi, John H Amuasi, Ganiyu Adeniyi Amusa, Daniel T Araki, Raphael Taiwo Aruleba, Tewachew Awoke, Melese Kitu Ayalew, Ahmed Y Azzam, Indrajit Banerjee, Nebiyou Simegnew Bayileyegn, Melaku Ashagrie Belete, Erin Chung, Nicole Davis Weaver, Dessalegn Demeke, Desalegn Getnet Demsie, Anteneh Mengist Dessie, Christiane Dolecek, Susanna J Dunachie, Abdelaziz Ed-Dra, Michael Ekholuenetale, Temitope Cyrus Ekundayo, Iman El Sayed, Muhammed Elhadi, Ibrahim Elsohaby, Adeniyi Francis Fagbamigbe, Nicholas A Feasey, Ginenus Fekadu, Frederick Fell, Mesfin Gebrehiwot, Anna Gershberg Hayoon, Kebede Embaye Gezae, Ramy Mohamed Ghazy, Authia P Gray, Tewodros Tesfa Hailegiyorgis, Georgina Haines-Woodhouse, Chieh Han, Ahmed I. Hasaballah, Andrea Haekyung Haselbeck, Simon I Hay, Kevin S Ikuta, Arnaud Iradukunda, Kenneth Chukwuemeka Iregbu, Chidozie C D Iwu, Chinwe Juliana Iwu-Jaja, Charity Ehimwenma Joshua, Gebrehiwot G Kassa, Patrick D.M.C. Katoto, Emmanuelle A P Kumaran, Hmwe Hmwe Kyu, Aseer Manilal, Barney McManigal, Addisu Melese, Tomislav Mestrovic, Kebede Haile Misgina, Nouh Saad Mohamed, Mustapha Mohammed, Shafiu Mohammed, Shikur Mohammed, Ali H Mokdad, Catrin E Moore, Neema Mturi, Temesgen Mulugeta, Christopher J L Murray, Fungai Musaigwa, Patrick Musicha, Lillian A Musila, Saravanan Muthupandian, Mohsen Naghavi, Pirouz Naghavi, Hadush Negash, Christina W. Obiero, Ismail A. Odetokun, Oluwaseun Adeolu Ogundijo, Lawrence Okidi, Osaretin Christabel Okonji, Andrew T Olagunju, Isaac Iyinoluwa

Olufadewa, Gi Deok Pak, Andrew Pollard, Mathieu Raad, Elrashdy Moustafa Mohamed Redwan, Gisela Robles Aguilar, Victor Daniel Rosenthal, Mohamed A. Saleh, Abdallah M Samy, Benn Sartorius, Aminu Shittu, Emmanuel Edwar Siddig, Andy Stergachis, Lucien R Swetschinski, Marius Belmondo Tincho, Tenaw Yimer Tiruye, Chukwuma David Umeokonkwo, Timothy Walsh, Judd L Walson, and Naod Gebrekrstos Zeru.

Drafting the work or revising it critically for important intellectual content

Sherief Abd-Elsalam, Lawan Hassan Adamu, Abiola Victor Adepoju, Ayman Ahmed, John H Amuasi, Ganiyu Adeniyi Amusa, Raphael Taiwo Aruleba, Melese Kitu Ayalew, Ahmed Y Azzam, Indrajit Banerjee, Afisu Basiru, James A Berkley, Erin Chung, Nicole Davis Weaver, Desalegn Getnet Demsie, Anteneh Mengist Dessie, Christiane Dolecek, Susanna J Dunachie, Iman El Sayed, Muhammed Elhadi, Ibrahim Elsohaby, Adeniyi Francis Fagbamigbe, Nicholas A Feasey, Mesfin Gebrehiwot, Anna Gershberg Hayoon, Authia P Gray, Tewodros Tesfa Hailegiyorgis, Georgina Haines-Woodhouse, Ahmed I. Hasaballah, Simon I Hay, Kevin S Ikuta, Kenneth Chukwuemeka Iregbu, Chidozie C D Iwu, Chinwe Juliana Iwu-Jaja, Assefa N Iyasu, Patrick D.M.C. Katoto, Emmanuelle A P Kumaran, Aseer Manilal, Jürgen May, Susan A McLaughlin, Barney McManigal, Tomislav Mestrovic, Kebede Haile Misgina, Nouh Saad Mohamed, Mustapha Mohammed, Shafiu Mohammed, Ali H Mokdad, Catrin E Moore, Vincent Mougin, Christopher J L Murray, Fungai Musaigwa, Saravanan Muthupandian, Mohsen Naghavi, Hadush Negash, Dooshanveer C. Nuckchady, Ismail A. Odetokun, Oluwaseun Adeolu Ogundijo, Lawrence Okidi, Osaretin Christabel Okonji, Andrew T Olagunju, Mathieu Raad, Hazem Ramadan, Elrashdy Moustafa Mohamed Redwan, Gisela Robles Aguilar, Victor Daniel Rosenthal, Abdallah M Samy, Benn Sartorius, Aminu Shittu, Emmanuel Edwar Siddig, Eskinder Ayalew Sisay, Lucien R Swetschinski, Wegen Beyene Tesfamariam, Marius Belmondo Tincho, Tenaw Yimer Tiruye, Chukwuma David Umeokonkwo, Judd L Walson, and Hadiza Yusuf.

Managing the estimation or publications process

Ahmed Y Azzam, Indrajit Banerjee, Nicole Davis Weaver, Christiane Dolecek, Simon I Hay, Mustapha Mohammed, Ali H Mokdad, Catrin E Moore, Christopher J L Murray, Mohsen Naghavi, Clotaire Donatien Rafaï, Abdallah M Samy, Benn Sartorius, Lucien R Swetschinski, and Eve E Wool.

African Region

Deaths (count) *associated with* bacterial antimicrobial resistance by pathogen−drug combinations, 2019

	Resistance to 1+	AG	Aminopenicillin	Anti−pseudomonal	BL−BLI	CP	3GC	4GC	FQ	Mono INH	Macrolide	MDR in S. Typhi and Paratyphi	MDR excluding XDR in TB	Methicillin	PCN	Mono RIF	TMP−SMX	Vanco	XDR in TB
Acinetobacter baumannii	48,200	20,800		45,800	28,900	20,500	37,300	28,400	27,400										
Citrobacter spp.	6,230	2,020		2,700		1,160	5,380	4,520	2,940										
Enterobacter spp.	35,100	16,700		13,700		5,670		28,600	20,100								27,700		
Enterococcus faecalis	17,600								17,300									1,150	
Enterococcus faecium	19,500								19,400									4,880	
Other enterococci	8,710								8,680									607	
Escherichia coli	147,000	48,800	137,000		107,000	15,500	81,700		82,400								131,000		
Group A Streptococcus	4,330										4,330								
Group B Streptococcus	54,800								30,700		39,400				1,250				
Haemophilus influenzae	13,300		11,400				4,290												
Klebsiella pneumoniae	184,000	126,000			170,000	20,800	155,000		127,000								170,000		
Morganella spp.	186						44	102	142										
Mycobacterium tuberculosis	41,900									13,100			24,500			3,960			334
Proteus spp.	10,700	3,190	9,300				2,530		4,830								8,680		
Pseudomonas aeruginosa	55,700	24,000		26,800		22,400	37,300	24,300	28,300										
Salmonella Paratyphi	89.6								72.8			19.2							
Salmonella Typhi	42,400								16,300			34,800							
Non−typhoidal Salmonella	1,300								1,300										
Serratia spp.	14,300	7,180		3,110		877	9,370	11,100	3,510										
Shigella spp.	10,400								10,400										
Staphylococcus aureus	136,000								36,700		73,700			65,500			91,500	2,140	
Streptococcus pneumoniae	195,000				38,500	45,500	28,100		42,300		46,500				63,700		180,000		
All pathogens	1,050,000	249,000	158,000	92,100	345,000	132,000	361,000	97,100	479,000	13,100	164,000	34,800	24,500	65,500	64,900	3,960	609,000	8,790	334

Counts
- < 5k
- 5k − <10k
- 10k − <25k
- 25k − <50k
- 50k − <75k
- 75k − <100k
- >=100k

African Region

Deaths (count) *attributable to* bacterial antimicrobial resistance by pathogen−drug combinations, 2019

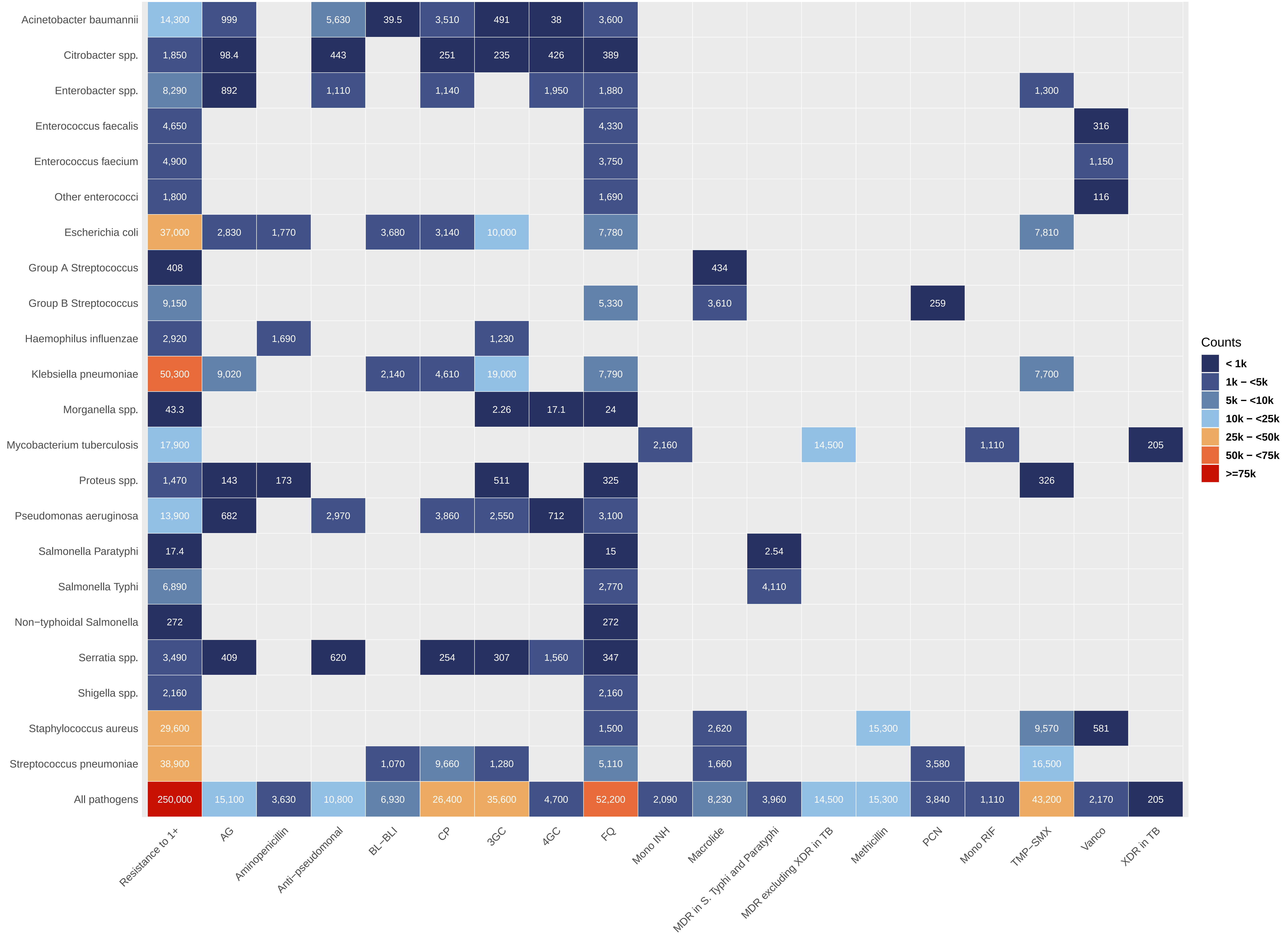

Central sub−Saharan Africa

Deaths (count) *associated with* bacterial antimicrobial resistance by pathogen−drug combinations, 2019

	Resistance to 1+	AG	Aminopenicillin	Anti−pseudomonal	BL−BLI	CP	3GC	4GC	FQ	Mono INH	Macrolide	MDR in S. Typhi and Paratyphi	MDR excluding XDR in TB	Methicillin	PCN	Mono RIF	TMP−SMX	Vanco	XDR in TB
Acinetobacter baumannii	5,800	2,030		5,640	3,550	2,280	4,660	2,880	3,320										
Citrobacter spp.	599	161		306		73.8	455	427	261										
Enterobacter spp.	3,680	1,560		1,450		712		3,050	1,950								2,730		
Enterococcus faecalis	1,960								1,930									160	
Enterococcus faecium	2,020								2,000									504	
Other enterococci	820								817									57.1	
Escherichia coli	14,400	4,730	12,900		10,400	1,590	6,910		8,050								12,400		
Group A Streptococcus	590										590								
Group B Streptococcus	6,720								3,860		4,910				345				
Haemophilus influenzae	1,930		1,840				475												
Klebsiella pneumoniae	18,600	13,300			16,800	490	15,000		12,700								16,700		
Morganella spp.	23.8						5.76	14.9	17.5										
Mycobacterium tuberculosis	9,220									4,600			3,720			852			50.6
Proteus spp.	1,180	314	1,050				284		514								922		
Pseudomonas aeruginosa	5,440	1,700		3,330		2,140	3,930	2,730	2,400										
Salmonella Paratyphi	0.189								0.127			0.064							
Salmonella Typhi	4,600								3,140			2,950							
Non−typhoidal Salmonella	333								333										
Serratia spp.	1,450	641		286		132	746	1,220	308										
Shigella spp.	106								106										
Staphylococcus aureus	12,600								5,250		5,440			5,600			8,660	145	
Streptococcus pneumoniae	21,000				8,200	4,020	2,250		11,200		6,910				7,400		18,800		
All pathogens	113,000	24,400	15,800	11,000	39,000	11,400	34,700	10,300	58,200	4,600	17,800	2,950	3,720	5,600	7,740	852	60,100	865	50.6

Counts
- < 2.5k
- 2.5k − <5k
- 5k − <10k
- 10k − <25k
- 25k − <50k
- 50k − <75k
- >=75k

Central sub−Saharan Africa

Deaths (count) *attributable to* bacterial antimicrobial resistance by pathogen−drug combinations, 2019

	Resistance to 1+	AG	Aminopenicillin	Anti−pseudomonal	BL−BLI	CP	3GC	4GC	FQ	Mono INH	Macrolide	MDR in S. Typhi and Paratyphi	MDR excluding XDR in TB	Methicillin	PCN	Mono RIF	TMP−SMX	Vanco	XDR in TB
Acinetobacter baumannii	1,730	96.8		766	0.925	387	38	0.294	441										
Citrobacter spp.	172	8.55		56		16.8	18.3	37.8	35.2										
Enterobacter spp.	882	84.3		122		146		214	185								132		
Enterococcus faecalis	522								479									42.6	
Enterococcus faecium	506								387									118	
Other enterococci	170								159									11	
Escherichia coli	3,540	279	151		417	332	822		780								761		
Group A Streptococcus	59.1										57.7								
Group B Streptococcus	1,150								659		431				59				
Haemophilus influenzae	407		275				131												
Klebsiella pneumoniae	4,850	946			270	164	1,950		772								750		
Morganella spp.	5.58						0.224	2.49	2.87										
Mycobacterium tuberculosis	3,210									740			2,210			238			31
Proteus spp.	161	14.1	20				58.1		34.5								34.1		
Pseudomonas aeruginosa	1,420	47.1		433		388	219	68.2	261										
Salmonella Paratyphi	0.035								0.026			0.008							
Salmonella Typhi	860								549			311							
Non−typhoidal Salmonella	70								70										
Serratia spp.	343	35.5		54.8		38	19.4	165	30.2										
Shigella spp.	21.9								21.9										
Staphylococcus aureus	2,670								215		189			1,300			929	43.5	
Streptococcus pneumoniae	4,460				313	766	107		1,340		204				270		1,460		
All pathogens	27,200	1,510	447	1,430	1,000	2,240	3,360	488	6,420	754	888	316	2,210	1,300	329	238	4,060	216	31

Counts
- < 2.5k
- 2.5k − <5k
- 5k − <10k
- 25k − <50k

Eastern sub−Saharan Africa

Deaths (count) *associated with* bacterial antimicrobial resistance by pathogen−drug combinations, 2019

	Resistance to 1+	AG	Aminopenicillin	Anti−pseudomonal	BL−BLI	CP	3GC	4GC	FQ	Mono INH	Macrolide	MDR in S. Typhi and Paratyphi	MDR excluding XDR in TB	Methicillin	PCN	Mono RIF	TMP−SMX	Vanco	XDR in TB
Acinetobacter baumannii	17,100	5,960		15,300	12,100	6,130	14,600	9,710	9,770										
Citrobacter spp.	2,090	645		914		292	1,810	1,310	1,290										
Enterobacter spp.	11,200	5,040		4,370		1,410		9,880	6,490								8,390		
Enterococcus faecalis	6,720								6,630									414	
Enterococcus faecium	6,600								6,530									1,740	
Other enterococci	2,580								2,570									154	
Escherichia coli	47,000	10,900	42,900		31,900	4,630	23,900		23,000								40,300		
Group A Streptococcus	1,960										1,960								
Group B Streptococcus	14,800								6,280		12,400				329				
Haemophilus influenzae	4,700		4,500				889												
Klebsiella pneumoniae	58,900	42,800			54,500	6,400	49,100		37,700								53,200		
Morganella spp.	67.1						15.8	39.7	50.2										
Mycobacterium tuberculosis	14,100									2,220			10,300			1,370			140
Proteus spp.	3,990	1,470	3,390				745		2,090								3,470		
Pseudomonas aeruginosa	16,000	4,760		6,490		4,380	12,600	5,280	7,120										
Salmonella Paratyphi	4.58								0.771			3.78							
Salmonella Typhi	16,400								6,840			12,600							
Non−typhoidal Salmonella	503								503										
Serratia spp.	4,410	2,140		1,620		243	2,720	3,000	1,200										
Shigella spp.	1,510								1,510										
Staphylococcus aureus	45,300								16,600		23,000			17,500			32,100	525	
Streptococcus pneumoniae	56,500				8,630	14,400	8,120		14,400		7,740				15,200		53,700		
All pathogens	332,000	73,700	50,800	28,700	107,000	37,900	115,000	29,200	151,000	2,220	45,100	12,600	10,300	17,500	15,500	1,370	191,000	2,830	140

Counts
- < 2.5k
- 2.5k − <5k
- 5k − <10k
- 10k − <25k
- 25k − <50k
- 50k − <75k
- >=75k

Eastern sub−Saharan Africa

Deaths (count) *attributable to* bacterial antimicrobial resistance by pathogen−drug combinations, 2019

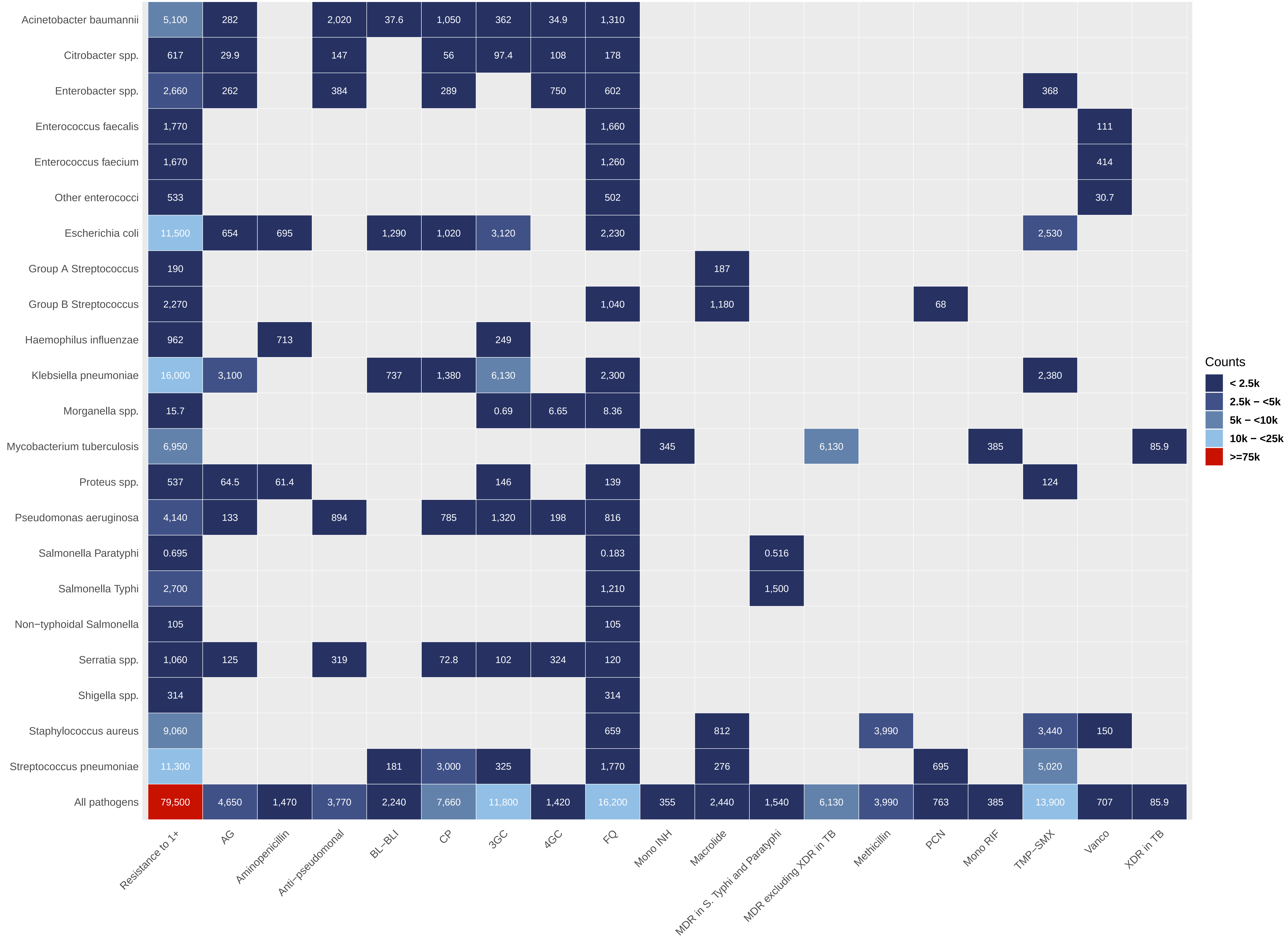

North Africa and Middle East

Deaths (count) *associated with* bacterial antimicrobial resistance by pathogen−drug combinations, 2019

	Resistance to 1+	AG	Aminopenicillin	Anti−pseudomonal	BL−BLI	CP	3GC	4GC	FQ	Mono INH	Macrolide	MDR in S. Typhi and Paratyphi	MDR excluding XDR in TB	Methicillin	PCN	Mono RIF	TMP−SMX	Vanco	XDR in TB
Acinetobacter baumannii	1,110	968		1,100	1,070	980	1,080	1,060	1,000										
Citrobacter spp.	59.4	6.65		19.8		11.3	40.5	19.1	30										
Enterobacter spp.	332	51.9		198		77		168	80								143		
Enterococcus faecalis	99.5								86.2									19	
Enterococcus faecium	695								690									389	
Other enterococci	259								258									111	
Escherichia coli	2,310	401	2,230		1,410	304	777		1,120								1,670		
Group A Streptococcus	19.5										19.5								
Group B Streptococcus	411								55.1		398				0.431				
Haemophilus influenzae	53.4		49.4				9.26												
Klebsiella pneumoniae	2,150	636			1,980	287	1,900		917								1,490		
Morganella spp.	5.91						2.13	1.18	5.13										
Mycobacterium tuberculosis	25.6									3.93			20			0.132			1.48
Proteus spp.	258	145	234				113		121								178		
Pseudomonas aeruginosa	1,220	444		499		571	909	609	632										
Salmonella Paratyphi	1.41								1.4			0.02							
Salmonella Typhi	10.3								9.25			0.841							
Non−typhoidal Salmonella	3.9								3.9										
Serratia spp.	91	22		20.4		19.4	65	45.5	13.3										
Shigella spp.	4.27								4.27										
Staphylococcus aureus	2,830								594		2,400			1,800			552	27.6	
Streptococcus pneumoniae	1,650				339	878	182		101		1,130				1,020		1,190		
All pathogens	13,600	2,670	2,510	1,840	4,800	3,130	5,080	1,900	5,720	3.93	3,940	0.86	20	1,800	1,020	0.132	5,220	547	1.48

Counts
- **< 2.5k**
- **2.5k − <5k**
- **5k − <10k**
- **10k − <25k**

North Africa and Middle East

Deaths (count) *attributable to* bacterial antimicrobial resistance by pathogen−drug combinations, 2019

	Resistance to 1+	AG	Aminopenicillin	Anti−pseudomonal	BL−BLI	CP	3GC	4GC	FQ	Mono INH	Macrolide	MDR in S. Typhi and Paratyphi	MDR excluding XDR in TB	Methicillin	PCN	Mono RIF	TMP−SMX	Vanco	XDR in TB
Acinetobacter baumannii	357	41		29.5	0.595	160	2.36	0.427	123										
Citrobacter spp.	17.1	0.344		3.65		2.7	3.83	1.53	5.02										
Enterobacter spp.	76.3	3.35		26.1		20.2		9.23	8.64								8.97		
Enterococcus faecalis	28.4								21.5									6.93	
Enterococcus faecium	203								114									89.5	
Other enterococci	61.3								42.7									18.6	
Escherichia coli	552	24.4	67.2		81.3	69.7	93.6		114								102		
Group A Streptococcus	1.95										1.82								
Group B Streptococcus	49.5								8.61		40				0.407				
Haemophilus influenzae	10.9		8.22				2.72												
Klebsiella pneumoniae	603	52.1			31.1	71.6	305		66.6								76.1		
Morganella spp.	1.4						0.281	0.209	0.91										
Mycobacterium tuberculosis	13.6									0.668			12			0.037			0.905
Proteus spp.	44.2	6.84	3.14				21.1		7.28								5.64		
Pseudomonas aeruginosa	314	12.3		43.3		100	66.1	23.9	68.6										
Salmonella Paratyphi	0.286								0.284			0.002							
Salmonella Typhi	2.07								1.92			0.148							
Non−typhoidal Salmonella	0.827								0.827										
Serratia spp.	24	1.34		4.46		5.41	6.1	5.25	1.44										
Shigella spp.	0.889								0.889										
Staphylococcus aureus	656								27.8		98.5			471			50.9	8.1	
Streptococcus pneumoniae	388				1.35	191	0.513		10.1		43.1				50.1		93.1		
All pathogens	3,410	142	78.6	107	114	620	502	40.6	624	0.666	184	0.154	12	471	50.5	0.037	337	123	0.905

Counts
- < 2.5k
- 2.5k − <5k

Southeast Asia

Deaths (count) *associated with* bacterial antimicrobial resistance by pathogen−drug combinations, 2019

	Resistance to 1+	AG	Aminopenicillin	Anti−pseudomonal	BL−BLI	CP	3GC	4GC	FQ	Mono INH	Macrolide	MDR in S. Typhi and Paratyphi	MDR excluding XDR in TB	Methicillin	PCN	Mono RIF	TMP−SMX	Vanco	XDR in TB
Acinetobacter baumannii	85.6	65.6		82	65.2	67	76.1	81.6	71.7										
Citrobacter spp.	5.16	0.647		0.675		0.939	3.2	3.17	2.11										
Enterobacter spp.	27.6	4.54		13.2		7.6		11.4	7.33								17.4		
Enterococcus faecalis	20.8								19.8									2.61	
Enterococcus faecium	45.8								45.6									10.5	
Other enterococci	21.5								21.3									7.53	
Escherichia coli	154	20	147		116	20.6	72.7		89.4								105		
Group A Streptococcus	7.63										7.63								
Group B Streptococcus	14.9								4		12.5				1.3				
Haemophilus influenzae	4.09		3.74				0.939												
Klebsiella pneumoniae	109	14			78.7	20.6	84.1		54.9								80.8		
Morganella spp.	0.432						0.255	0.094	0.259										
Mycobacterium tuberculosis	0.706									0.474			0.178			0.02			0.034
Proteus spp.	19.5	13.4	15.7				7.25		7.61								11		
Pseudomonas aeruginosa	62.2	18.4		23.5		44.1	31.7	23.9	27.5										
Salmonella Paratyphi	0.415								0.407			0.009							
Salmonella Typhi	1.16								1.05			0.119							
Non−typhoidal Salmonella	0.157								0.157										
Serratia spp.	5.23	0.784		1.64		1.15	3.62	1.73	1.65										
Shigella spp.	0.056								0.056										
Staphylococcus aureus	178								51		95.3			163			44.9	3.49	
Streptococcus pneumoniae	81.9				13.8	40.4	17.9		3.56		49.2				27.2		60.2		
All pathogens	847	137	167	121	273	202	298	122	410	0.474	165	0.128	0.178	163	28.5	0.02	319	24.2	0.034

Counts
< 2.5k

Southeast Asia

Deaths (count) *attributable to* bacterial antimicrobial resistance by pathogen−drug combinations, 2019

	Resistance to 1+	AG	Aminopenicillin	Anti−pseudomonal	BL−BLI	CP	3GC	4GC	FQ	Mono INH	Macrolide	MDR in S. Typhi and Paratyphi	MDR excluding XDR in TB	Methicillin	PCN	Mono RIF	TMP−SMX	Vanco	XDR in TB
Acinetobacter baumannii	27	2.98		3.38	0.014	11	0.438	0.416	8.92										
Citrobacter spp.	1.44	0.034		0.13		0.224	0.307	0.423	0.325										
Enterobacter spp.	6.12	0.301		1.47		1.96		0.473	0.785								1.13		
Enterococcus faecalis	5.68								4.89									0.786	
Enterococcus faecium	11.4								8.9									2.48	
Other enterococci	4.93								3.67									1.25	
Escherichia coli	38.8	1.21	2.32		5.7	4.6	9.27		9.49								6.21		
Group A Streptococcus	0.757										0.795								
Group B Streptococcus	2.18								0.692		1.21				0.241				
Haemophilus influenzae	0.861		0.592				0.269												
Klebsiella pneumoniae	29.7	1.42			1.55	5.36	12.3		4.26								4.79		
Morganella spp.	0.11						0.046	0.021	0.044										
Mycobacterium tuberculosis	0.207									0.073			0.106			0.006			0.021
Proteus spp.	3.1	0.725	0.221				1.34		0.454								0.356		
Pseudomonas aeruginosa	15.3	0.498		1.25		8.36	1.74	0.566	2.89										
Salmonella Paratyphi	0.085								0.084			0.001							
Salmonella Typhi	0.233								0.218			0.016							
Non−typhoidal Salmonella	0.033								0.033										
Serratia spp.	1.38	0.045		0.343		0.318	0.318	0.161	0.19										
Shigella spp.	0.012								0.012										
Staphylococcus aureus	54.5								2.29		3.3			44.3			3.76	0.786	
Streptococcus pneumoniae	18				0.049	9.2	0.345		0.396		2.01				0.739		5.26		
All pathogens	222	7.11	3.14	6.57	7.3	41	26.4	2.06	48.6	0.072	7.28	0.017	0.106	44.3	0.98	0.006	21.5	5.31	0.021

Counts

< 2.5k

Southern sub−Saharan Africa

Deaths (count) *associated with* bacterial antimicrobial resistance by pathogen−drug combinations, 2019

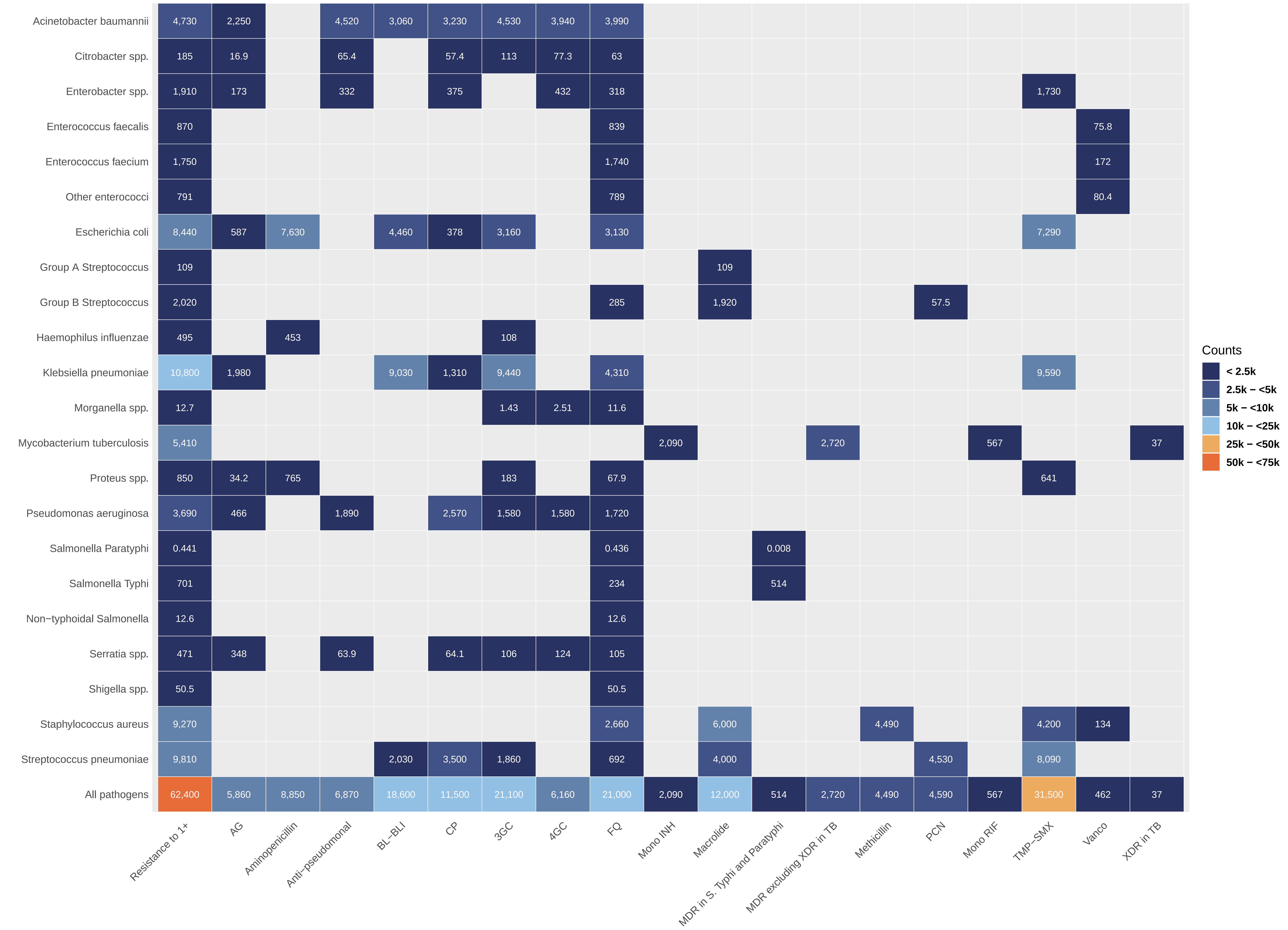

Southern sub−Saharan Africa

Deaths (count) *attributable to* bacterial antimicrobial resistance by pathogen−drug combinations, 2019

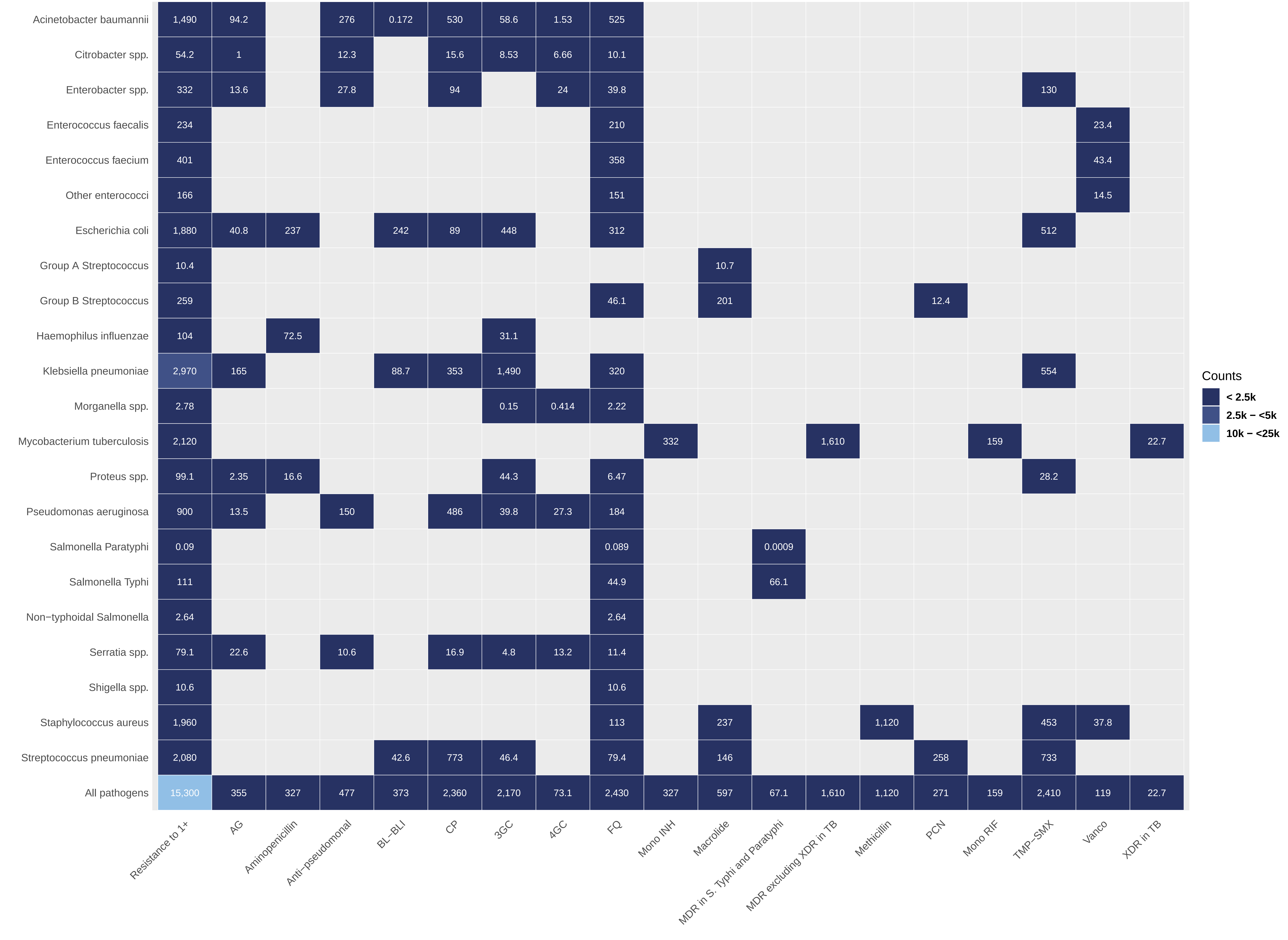

Western sub−Saharan Africa

Deaths (count) *associated with* bacterial antimicrobial resistance by pathogen−drug combinations, 2019

	Resistance to 1+	AG	Aminopenicillin	Anti−pseudomonal	BL−BLI	CP	3GC	4GC	FQ	Mono INH	Macrolide	MDR in S. Typhi and Paratyphi	MDR excluding XDR in TB	Methicillin	PCN	Mono RIF	TMP−SMX	Vanco	XDR in TB
Acinetobacter baumannii	19,300	9,490		19,100	9,080	7,830	12,400	10,800	9,270										
Citrobacter spp.	3,290	1,190		1,390		725	2,960	2,690	1,300										
Enterobacter spp.	18,000	9,840		7,280		3,090		15,000	11,200								14,700		
Enterococcus faecalis	7,920								7,810									479	
Enterococcus faecium	8,420								8,370									2,070	
Other enterococci	4,240								4,230									197	
Escherichia coli	74,800	32,200	71,500		59,200	8,530	46,900		47,000								69,200		
Group A Streptococcus	1,650										1,650								
Group B Streptococcus	30,900								20,200		19,800				521				
Haemophilus influenzae	6,060		4,500				2,810												
Klebsiella pneumoniae	93,500	67,600			87,500	12,300	79,200		70,900								89,000		
Morganella spp.	75.8						18.6	43.8	57.4										
Mycobacterium tuberculosis	13,200									4,200			7,710			1,170			105
Proteus spp.	4,420	1,210	3,850				1,200		2,030								3,450		
Pseudomonas aeruginosa	29,200	16,600		14,600		12,700	18,300	14,100	16,400										
Salmonella Paratyphi	82.6								69.7			15.3							
Salmonella Typhi	20,600								6,110			18,700							
Non−typhoidal Salmonella	449								449										
Serratia spp.	7,820	4,020		1,120		417	5,730	6,710	1,880										
Shigella spp.	8,690								8,690										
Staphylococcus aureus	65,500								11,600		36,800			35,900			45,900	1,310	
Streptococcus pneumoniae	106,000				19,300	22,700	15,600		15,900		26,700				35,600		98,200		
All pathogens	524,000	142,000	79,900	43,500	175,000	68,200	185,000	49,300	244,000	4,200	84,900	18,700	7,710	35,900	36,100	1,170	321,000	4,050	105

Counts
- < 2.5k
- 2.5k − <5k
- 5k − <10k
- 10k − <25k
- 25k − <50k
- 50k − <75k
- >=75k

Western sub−Saharan Africa

Deaths (count) *attributable to* bacterial antimicrobial resistance by pathogen−drug combinations, 2019

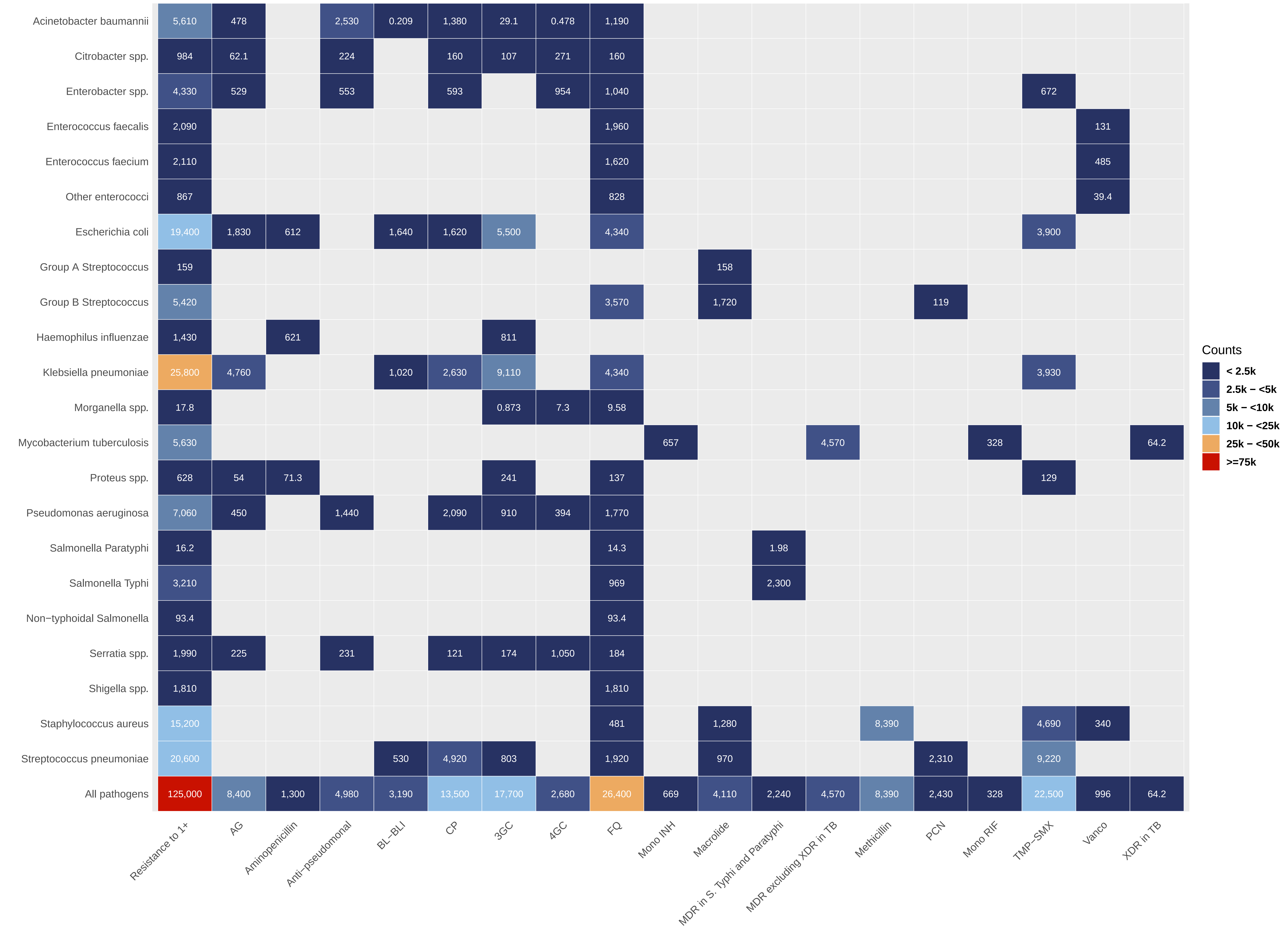

Algeria

Deaths (count) *associated with* bacterial antimicrobial resistance by pathogen−drug combinations, 2019

	Resistance to 1+	AG	Aminopenicillin	Anti−pseudomonal	BL−BLI	CP	3GC	4GC	FQ	Mono INH	Macrolide	MDR in S. Typhi and Paratyphi	MDR excluding XDR in TB	Methicillin	PCN	Mono RIF	TMP−SMX	Vanco	XDR in TB
Acinetobacter baumannii	1,110	968		1,100	1,070	980	1,080	1,060	1,000										
Citrobacter spp.	59.4	6.65		19.8		11.3	40.5	19.1	30										
Enterobacter spp.	332	51.9		198		77		168	80								143		
Enterococcus faecalis	99.5								86.2									19	
Enterococcus faecium	695								690									389	
Other enterococci	259								258									111	
Escherichia coli	2,310	401	2,230		1,410	304	777		1,120								1,670		
Group A Streptococcus	19.5										19.5								
Group B Streptococcus	411								55.1		398				0.431				
Haemophilus influenzae	53.4		49.4				9.26												
Klebsiella pneumoniae	2,150	636			1,980	287	1,900		917								1,490		
Morganella spp.	5.91						2.13	1.18	5.13										
Mycobacterium tuberculosis	25.6									3.93			20			0.132			1.48
Proteus spp.	258	145	234				113		121								178		
Pseudomonas aeruginosa	1,220	444		499		571	909	609	632										
Salmonella Paratyphi	1.41								1.4			0.02							
Salmonella Typhi	10.3								9.25			0.841							
Non−typhoidal Salmonella	3.9								3.9										
Serratia spp.	91	22		20.4		19.4	65	45.5	13.3										
Shigella spp.	4.27								4.27										
Staphylococcus aureus	2,830								594		2,400			1,800			552	27.6	
Streptococcus pneumoniae	1,650				339	878	182		101		1,130				1,020		1,190		
All pathogens	13,600	2,670	2,510	1,840	4,800	3,130	5,080	1,900	5,720	3.93	3,940	0.86	20	1,800	1,020	0.132	5,220	547	1.48

Counts
- < 500
- 500 − <1k
- 1k − <2.5k
- 2.5k − <7.5k
- 7.5k − <15k

Algeria

Deaths (count) *attributable to* bacterial antimicrobial resistance by pathogen−drug combinations, 2019

	Resistance to 1+	AG	Aminopenicillin	Anti−pseudomonal	BL−BLI	CP	3GC	4GC	FQ	Mono INH	Macrolide	MDR in S. Typhi and Paratyphi	MDR excluding XDR in TB	Methicillin	PCN	Mono RIF	TMP−SMX	Vanco	XDR in TB
Acinetobacter baumannii	357	40.1		29.5	0.595	160	2.36	0.427	123										
Citrobacter spp.	17.1	0.344		3.65		2.7	3.83	1.53	5.02										
Enterobacter spp.	76.3	3.35		26.1		20.2		9.23	8.64								8.53		
Enterococcus faecalis	28.4								21.5									6.93	
Enterococcus faecium	203								114									89.5	
Other enterococci	61.3								42.7									18.6	
Escherichia coli	552	24.4	67.2		81.3	69.7	93.6		114								102		
Group A Streptococcus	1.9										1.96								
Group B Streptococcus	49.9								8.61		41.2				0.407				
Haemophilus influenzae	10.9		8.22				2.72												
Klebsiella pneumoniae	603	52.1			31.1	71.6	305		66.6								76.1		
Morganella spp.	1.4						0.281	0.209	0.91										
Mycobacterium tuberculosis	13.6									0.649			12			0.037			0.905
Proteus spp.	44.2	6.84	3.36				21.1		7.28								5.81		
Pseudomonas aeruginosa	314	11.8		43.3		100	66.1	23.9	68.6										
Salmonella Paratyphi	0.286								0.284			0.002							
Salmonella Typhi	2.07								1.92			0.139							
Non−typhoidal Salmonella	0.827								0.827										
Serratia spp.	24	1.28		4.46		5.41	6.1	5.25	1.44										
Shigella spp.	0.889								0.889										
Staphylococcus aureus	656								27.8		98.5			471			50.9	8.1	
Streptococcus pneumoniae	388				1.35	191	0.513		10.1		41.5				50.1		93.1		
All pathogens	3,410	142	78.6	107	114	620	502	40.6	624	0.683	184	0.15	12	471	50.5	0.037	337	123	0.905

Counts
- < 500
- 500 − <1k
- 2.5k − <7.5k

Angola

Deaths (count) *associated with* bacterial antimicrobial resistance by pathogen−drug combinations, 2019

	Resistance to 1+	AG	Aminopenicillin	Anti−pseudomonal	BL−BLI	CP	3GC	4GC	FQ	Mono INH	Macrolide	MDR in S. Typhi and Paratyphi	MDR excluding XDR in TB	Methicillin	PCN	Mono RIF	TMP−SMX	Vanco	XDR in TB
Acinetobacter baumannii	1,040	371		1,020	564	356	786	469	614										
Citrobacter spp.	170	37.8		85.2		20.1	132	118	72.4										
Enterobacter spp.	971	429		384		156		791	505								735		
Enterococcus faecalis	412								405									29.3	
Enterococcus faecium	454								452									113	
Other enterococci	202								201									13.5	
Escherichia coli	3,050	979	2,680		2,220	375	1,190		1,410								2,730		
Group A Streptococcus	114										114								
Group B Streptococcus	1,640								798		1,280				86.4				
Haemophilus influenzae	380		355				87.4												
Klebsiella pneumoniae	3,840	2,670			3,470	101	3,000		2,660								3,510		
Morganella spp.	4.48						1.03	2.74	3.27										
Mycobacterium tuberculosis	1,190									401			750			31.7			10.2
Proteus spp.	247	65.1	221				55.9		73.6								195		
Pseudomonas aeruginosa	1,170	391		720		480	836	513	540										
Salmonella Paratyphi	0.023								0.011			0.012							
Salmonella Typhi	409								324			98.7							
Non−typhoidal Salmonella	3.51								3.51										
Serratia spp.	323	146		67.9		25	163	253	68.8										
Shigella spp.	13.2								13.2										
Staphylococcus aureus	2,240								708		1,040			927			1,550	24.4	
Streptococcus pneumoniae	4,810				1,860	1,030	543		1,160		1,330				1,500		4,560		
All pathogens	22,700	5,090	3,260	2,270	8,110	2,540	6,800	2,150	10,000	401	3,760	98.8	750	927	1,580	31.7	13,300	180	10.2

Counts
- < 500
- 500 − <1k
- 1k − <2.5k
- 2.5k − <7.5k
- 7.5k − <15k
- 15k − <25k

Angola

Deaths (count) *attributable to* bacterial antimicrobial resistance by pathogen−drug combinations, 2019

	Resistance to 1+	AG	Aminopenicillin	Anti−pseudomonal	BL−BLI	CP	3GC	4GC	FQ	Mono INH	Macrolide	MDR in S. Typhi and Paratyphi	MDR excluding XDR in TB	Methicillin	PCN	Mono RIF	TMP−SMX	Vanco	XDR in TB
Acinetobacter baumannii	306	17.3		142	0.018	59.9	4.04	0.021	82.2										
Citrobacter spp.	49.2	1.99		15.9		4.63	5.9	10.9	9.88										
Enterobacter spp.	229	23.5		33.4		32.2		56.1	47.7								35.1		
Enterococcus faecalis	109								101									8.01	
Enterococcus faecium	114								87.4									26.6	
Other enterococci	41.8								39.2									2.62	
Escherichia coli	722	58.9	33.2		101	81.5	136		132								179		
Group A Streptococcus	10.7										11								
Group B Streptococcus	268								135		112				15.2				
Haemophilus influenzae	79.7		55.1				24.6												
Klebsiella pneumoniae	987	187			60	33.8	385		161								160		
Morganella spp.	1.05						0.042	0.464	0.542										
Mycobacterium tuberculosis	523									63.5			444			8.9			6.24
Proteus spp.	32.1	3.06	4.29				11.9		5.14								7.72		
Pseudomonas aeruginosa	303	10.6		90.1		86.3	48.5	8.77	58.8										
Salmonella Paratyphi	0.004								0.002			0.002							
Salmonella Typhi	78.6								66.1			12.8							
Non−typhoidal Salmonella	0.74								0.74										
Serratia spp.	74.9	8.41		13.3		7.43	5.42	33.6	6.81										
Shigella spp.	2.78								2.78										
Staphylococcus aureus	459								28.9		38.1			212			172	7.62	
Streptococcus pneumoniae	957				75.3	213	24.4		135		45.3				61.6		403		
All pathogens	5,350	311	92.7	295	236	519	645	110	1,100	59.7	211	12.7	444	212	76.8	8.9	957	44.8	6.24

Counts
- < 500
- 500 − <1k
- 1k − <2.5k
- 2.5k − <7.5k

Benin

Deaths (count) *associated with* bacterial antimicrobial resistance by pathogen−drug combinations, 2019

	Resistance to 1+	AG	Aminopenicillin	Anti−pseudomonal	BL−BLI	CP	3GC	4GC	FQ	Mono INH	Macrolide	MDR in S. Typhi and Paratyphi	MDR excluding XDR in TB	Methicillin	PCN	Mono RIF	TMP−SMX	Vanco	XDR in TB
Acinetobacter baumannii	609	261		605	229	196	366	362	251										
Citrobacter spp.	72.6	24.9		32.4		11.5	65.4	61.8	30.9										
Enterobacter spp.	425	206		163		71.7		363	273								349		
Enterococcus faecalis	220								217									14.3	
Enterococcus faecium	219								218									50	
Other enterococci	108								108									4.99	
Escherichia coli	1,920	752	1,820		1,560	221	818		916								1,800		
Group A Streptococcus	41.9										41.9								
Group B Streptococcus	720								499		436				13.6				
Haemophilus influenzae	155		113				72.3												
Klebsiella pneumoniae	2,670	1,920			2,500	107	2,180		2,000								2,550		
Morganella spp.	2.07						0.512	1.26	1.52										
Mycobacterium tuberculosis	313									196			115			0.998			1.57
Proteus spp.	119	31.3	104				31.3		42.1								93.1		
Pseudomonas aeruginosa	772	440		414		295	428	339	419										
Salmonella Paratyphi	0.143								0.121			0.024							
Salmonella Typhi	748								194			679							
Non−typhoidal Salmonella	0.564								0.564										
Serratia spp.	207	113		28.8		9.37	164	173	55.4										
Shigella spp.	23.1								23.1										
Staphylococcus aureus	1,980								383		1,130			833			1,370	63.3	
Streptococcus pneumoniae	2,600				510	644	304		242		637				212		2,530		
All pathogens	13,900	3,740	2,040	1,240	4,800	1,560	4,430	1,300	5,880	196	2,250	679	115	833	225	0.998	8,700	133	1.57

Counts
- < 500
- 500 − <1k
- 1k − <2.5k
- 2.5k − <7.5k
- 7.5k − <15k

Benin

Deaths (count) *attributable to* bacterial antimicrobial resistance by pathogen−drug combinations, 2019

	Resistance to 1+	AG	Aminopenicillin	Anti−pseudomonal	BL−BLI	CP	3GC	4GC	FQ	Mono INH	Macrolide	MDR in S. Typhi and Paratyphi	MDR excluding XDR in TB	Methicillin	PCN	Mono RIF	TMP−SMX	Vanco	XDR in TB
Acinetobacter baumannii	174	13.8		92.1	0.003	35.1	0.066	0.012	32.8										
Citrobacter spp.	21.6	1.31		5.53		2.5	1.91	6.5	3.87										
Enterobacter spp.	102	10.6		12.2		13.8		24.4	25.8								15.6		
Enterococcus faecalis	58.1								54.3									3.84	
Enterococcus faecium	54.2								42.5									11.8	
Other enterococci	22								21									1.01	
Escherichia coli	463	45	18.8		64.2	45.2	92.2		84.8								112		
Group A Streptococcus	3.91										3.67								
Group B Streptococcus	129								89		36				2.9				
Haemophilus influenzae	36.6		15.7				20.9												
Klebsiella pneumoniae	702	133			35.2	29.7	271		121								113		
Morganella spp.	0.486						0.022	0.212	0.252										
Mycobacterium tuberculosis	100									31.7			68.4			0.281			0.96
Proteus spp.	16.3	1.44	1.99				6.48		2.88								3.61		
Pseudomonas aeruginosa	180	12.9		47.4		48.7	18	7.9	45.8										
Salmonella Paratyphi	0.028								0.025			0.003							
Salmonella Typhi	115								30.7			83.5							
Non−typhoidal Salmonella	0.117								0.117										
Serratia spp.	53.7	6.18		6.01		2.77	5.91	27.2	5.34										
Shigella spp.	4.9								4.9										
Staphylococcus aureus	411								15		41.9			189			149	16.1	
Streptococcus pneumoniae	471				14.6	140	8.19		28.9		23.5				2.16		254		
All pathogens	3,120	224	36.5	163	114	318	425	66.3	608	30.5	107	81.2	68.4	189	5.06	0.281	647	32.7	0.96

Counts
- < 500
- 500 − <1k
- 2.5k − <7.5k

Botswana

Deaths (count) *associated with* bacterial antimicrobial resistance by pathogen−drug combinations, 2019

	Resistance to 1+	AG	Aminopenicillin	Anti−pseudomonal	BL−BLI	CP	3GC	4GC	FQ	Mono INH	Macrolide	MDR in S. Typhi and Paratyphi	MDR excluding XDR in TB	Methicillin	PCN	Mono RIF	TMP−SMX	Vanco	XDR in TB
Acinetobacter baumannii	117	73.3		113	84.8	90.1	111	93.7	102										
Citrobacter spp.	7.1	0.448		1.57		2.37	4.8	3.28	2.53										
Enterobacter spp.	55.7	5.25		15		10.3		16.6	10.9								49.7		
Enterococcus faecalis	25.1								23.9									2.41	
Enterococcus faecium	59.7								59.4									13.3	
Other enterococci	21.4								21.3									1	
Escherichia coli	270	29.1	265		151	8.79	84.3		56.2								232		
Group A Streptococcus	2.81										2.81								
Group B Streptococcus	52.5								7.27		49.8				3.58				
Haemophilus influenzae	11.9		8.6				5.37												
Klebsiella pneumoniae	304	77.1			270	1.32	238		126								265		
Morganella spp.	0.265						0.038	0.043	0.248										
Mycobacterium tuberculosis	140									42.3			79.7			17			1.08
Proteus spp.	23.6	1.64	19.8				5.03		4.92								19.4		
Pseudomonas aeruginosa	133	32.2		67.5		89.7	58.5	49.5	80.6										
Salmonella Paratyphi	0.012								0.012			0.0001							
Salmonella Typhi	13.4								4.94			9.48							
Non−typhoidal Salmonella	0.067								0.067										
Serratia spp.	12.4	7.91		3.33		1.93	3.84	4.33	2.56										
Shigella spp.	2.25								2.25										
Staphylococcus aureus	239								126		77.9			28.3			142	4.32	
Streptococcus pneumoniae	300				70	97.7	47.6		21.3		92.3				146		258		
All pathogens	1,790	227	293	201	576	302	559	167	652	42.3	223	9.48	79.7	28.3	149	17	967	21.1	1.08

Counts
- < 500
- 500 − <1k
- 1k − <2.5k

Botswana

Deaths (count) *attributable to* bacterial antimicrobial resistance by pathogen−drug combinations, 2019

	Resistance to 1+	AG	Aminopenicillin	Anti−pseudomonal	BL−BLI	CP	3GC	4GC	FQ	Mono INH	Macrolide	MDR in S. Typhi and Paratyphi	MDR excluding XDR in TB	Methicillin	PCN	Mono RIF	TMP−SMX	Vanco	XDR in TB
Acinetobacter baumannii	37	3.03		5.27	0.007	14.6	1.07	0.011	13										
Citrobacter spp.	2.09	0.023		0.268		0.614	0.485	0.308	0.391										
Enterobacter spp.	10.1	0.422		1.45		2.5		0.761	1.31								3.71		
Enterococcus faecalis	6.76								5.99									0.772	
Enterococcus faecium	14.7								11.6									3.13	
Other enterococci	4.37								4.17									0.2	
Escherichia coli	57.9	1.97	10.6		8.92	2.15	12.6		5.65								16		
Group A Streptococcus	0.261										0.265								
Group B Streptococcus	6.57								1.15		4.97				0.639				
Haemophilus influenzae	2.8		1.24				1.57												
Klebsiella pneumoniae	76.3	6.2			5.77	0.852	40		8.81								14.8		
Morganella spp.	0.058						0.004	0.007	0.047										
Mycobacterium tuberculosis	59.2									6.75			46.9			4.81			0.663
Proteus spp.	2.89	0.101	0.399				1.14		0.392								0.848		
Pseudomonas aeruginosa	31.9	0.754		4.42		15.8	1.51	0.631	8.83										
Salmonella Paratyphi	0.002								0.002			0.00002							
Salmonella Typhi	2.16								0.946			1.22							
Non−typhoidal Salmonella	0.014								0.014										
Serratia spp.	2.38	0.506		0.594		0.511	0.133	0.374	0.271										
Shigella spp.	0.474								0.474										
Staphylococcus aureus	35.9								6.13		2.81			8.81			16.8	1.34	
Streptococcus pneumoniae	63				1.75	21.2	1.11		2.45		3.16				9.34		23.8		
All pathogens	417	13.1	12.2	12	16.4	58.2	59.6	2.09	71.6	7.01	11.5	1.18	46.9	8.81	9.98	4.81	75.9	5.45	0.663

Counts

< 500

Burkina Faso

Deaths (count) *associated with* bacterial antimicrobial resistance by pathogen−drug combinations, 2019

	Resistance to 1+	AG	Aminopenicillin	Anti−pseudomonal	BL−BLI	CP	3GC	4GC	FQ	Mono INH	Macrolide	MDR in S. Typhi and Paratyphi	MDR excluding XDR in TB	Methicillin	PCN	Mono RIF	TMP−SMX	Vanco	XDR in TB
Acinetobacter baumannii	1,200	461		1,180	531	302	733	800	509										
Citrobacter spp.	144	37		60.8		19.1	126	119	51.4										
Enterobacter spp.	794	299		281		140		657	538								620		
Enterococcus faecalis	484								478									31.2	
Enterococcus faecium	458								456									97.7	
Other enterococci	216								216									12.6	
Escherichia coli	4,320	2,310	4,140		3,400	471	1,930		2,230								3,840		
Group A Streptococcus	95										95								
Group B Streptococcus	1,300								907		760				13.5				
Haemophilus influenzae	454		359				199												
Klebsiella pneumoniae	6,230	4,030			5,610	220	5,330		4,570								5,930		
Morganella spp.	6.15						1.45	3.81	4.51										
Mycobacterium tuberculosis	659									165			481			6.35			6.51
Proteus spp.	241	28	213				57.5		89.7								175		
Pseudomonas aeruginosa	1,470	610		893		500	791	671	831										
Salmonella Paratyphi	15.1								12.5			2.8							
Salmonella Typhi	1,300								456			900							
Non−typhoidal Salmonella	38								38										
Serratia spp.	413	189		47.1		69.3	304	359	107										
Shigella spp.	38.9								38.9										
Staphylococcus aureus	4,800								820		3,290			1,840			2,830	196	
Streptococcus pneumoniae	5,810				2,000	1,250	674		576		1,750				497		5,520		
All pathogens	30,500	7,960	4,710	2,460	11,500	2,970	10,100	2,610	12,900	165	5,900	902	481	1,840	510	6.35	18,900	337	6.51

Counts
- < 500
- 500 − <1k
- 1k − <2.5k
- 2.5k − <7.5k
- 7.5k − <15k
- 15k − <25k
- >=25k

Burkina Faso

Deaths (count) *attributable to* bacterial antimicrobial resistance by pathogen−drug combinations, 2019

	Resistance to 1+	AG	Aminopenicillin	Anti−pseudomonal	BL−BLI	CP	3GC	4GC	FQ	Mono INH	Macrolide	MDR in S. Typhi and Paratyphi	MDR excluding XDR in TB	Methicillin	PCN	Mono RIF	TMP−SMX	Vanco	XDR in TB
Acinetobacter baumannii	343	24.5		190	0.012	56.9	1.44	0.068	69.5										
Citrobacter spp.	42.4	1.93		11.3		4.45	4.61	13.6	6.51										
Enterobacter spp.	192	14.9		20.8		27.3		46	54.6								28.3		
Enterococcus faecalis	127								119									8.25	
Enterococcus faecium	113								89.5									23.4	
Other enterococci	44.7								42.2									2.5	
Escherichia coli	1,050	141	49.7		136	93	215		200								220		
Group A Streptococcus	9.42										8.4								
Group B Streptococcus	232								163		64.8				3.59				
Haemophilus influenzae	106		48.9				56.8												
Klebsiella pneumoniae	1,660	281			57.6	64.1	698		281								274		
Morganella spp.	1.44						0.058	0.64	0.744										
Mycobacterium tuberculosis	317									24.7			285			1.79			3.99
Proteus spp.	31.6	1.46	4.49				12.6		6.28								6.8		
Pseudomonas aeruginosa	355	17.5		119		82.8	27.3	14.8	94.1										
Salmonella Paratyphi	2.97								2.6			0.359							
Salmonella Typhi	207								90			118							
Non−typhoidal Salmonella	8.02								8.02										
Serratia spp.	108	10.1		8.72		17.4	9.14	52.2	10.2										
Shigella spp.	8.17								8.17										
Staphylococcus aureus	943								34.6		129			426			302	52.5	
Streptococcus pneumoniae	1,060				83.9	272	31		69.6		65.5				7.36		535		
All pathogens	6,960	492	103	350	273	618	1,050	127	1,350	29.1	269	116	285	426	10.9	1.79	1,370	86.6	3.99

Counts
- < 500
- 500 − <1k
- 1k − <2.5k
- 2.5k − <7.5k

Burundi

Deaths (count) *associated with* bacterial antimicrobial resistance by pathogen−drug combinations, 2019

	Resistance to 1+	AG	Aminopenicillin	Anti−pseudomonal	BL−BLI	CP	3GC	4GC	FQ	Mono INH	Macrolide	MDR in S. Typhi and Paratyphi	MDR excluding XDR in TB	Methicillin	PCN	Mono RIF	TMP−SMX	Vanco	XDR in TB
Acinetobacter baumannii	598	159		537	400	195	536	373	217										
Citrobacter spp.	50.7	11.2		23.2		3.12	42.6	33.8	30.1										
Enterobacter spp.	296	148		118		38.2		271	166								212		
Enterococcus faecalis	202								200									11.8	
Enterococcus faecium	193								191									53.2	
Other enterococci	58.6								58.3									3.96	
Escherichia coli	1,690	382	1,600		1,190	130	604		648								1,400		
Group A Streptococcus	80.9										80.9								
Group B Streptococcus	463								172		407				15.6				
Haemophilus influenzae	219		217				29												
Klebsiella pneumoniae	1,990	1,560			1,850	24.1	1,410		973								1,770		
Morganella spp.	2.2						0.481	1.44	1.55										
Mycobacterium tuberculosis	707									76.6			597			24.7			8.13
Proteus spp.	119	41.9	99.4				22.2		70.7								104		
Pseudomonas aeruginosa	495	151		246		128	405	192	173										
Salmonella Paratyphi	0.017								0.003			0.014							
Salmonella Typhi	615								253			443							
Non−typhoidal Salmonella	0.702								0.702										
Serratia spp.	157	71		50.6		7	109	132	37.4										
Shigella spp.	176								176										
Staphylococcus aureus	1,390								461		533			561			978	18	
Streptococcus pneumoniae	1,840				338	411	220		989		157				234		1,700		
All pathogens	11,300	2,520	1,910	975	3,780	936	3,380	1,000	4,820	76.6	1,180	443	597	561	250	24.7	6,170	86.9	8.13

Counts
- < 500
- 500 – <1k
- 1k – <2.5k
- 2.5k – <7.5k
- 7.5k – <15k

Burundi

Deaths (count) *attributable to* bacterial antimicrobial resistance by pathogen−drug combinations, 2019

	Resistance to 1+	AG	Aminopenicillin	Anti−pseudomonal	BL−BLI	CP	3GC	4GC	FQ	Mono INH	Macrolide	MDR in S. Typhi and Paratyphi	MDR excluding XDR in TB	Methicillin	PCN	Mono RIF	TMP−SMX	Vanco	XDR in TB
Acinetobacter baumannii	179	8.54		84.8	0.964	38.1	16.6	0.075	29.8										
Citrobacter spp.	14.8	0.519		4.15		0.714	2.06	3.09	4.23										
Enterobacter spp.	71.2	7.85		10.4		7.77		21.1	14.9								9.37		
Enterococcus faecalis	53.1								50									3.13	
Enterococcus faecium	49.2								36.7									12.6	
Other enterococci	12.2								11.4									0.789	
Escherichia coli	385	23.5	33.6		66.3	29.2	78.2		62.6								91.6		
Group A Streptococcus	8.15										7.5								
Group B Streptococcus	69.1								27.9		38.7				2.96				
Haemophilus influenzae	43.1		35.1				8												
Klebsiella pneumoniae	497	116			45.4	10.5	186		58.5								80.5		
Morganella spp.	0.514						0.015	0.244	0.254										
Mycobacterium tuberculosis	379									10.9			355			6.91			4.99
Proteus spp.	16.3	1.8	1.66				4.29		4.71								3.71		
Pseudomonas aeruginosa	130	4.39		37.7		23.6	38.6	6.23	19.3										
Salmonella Paratyphi	0.003								0.0008			0.002							
Salmonella Typhi	101								46.9			53.5							
Non−typhoidal Salmonella	0.146								0.146										
Serratia spp.	39.5	4.08		10.2		2.21	3	16.5	3.68										
Shigella spp.	35.9								35.9										
Staphylococcus aureus	285								19.4		19.4			130			111	5.17	
Streptococcus pneumoniae	378				8.47	78.6	6.6		126		5.71				4.45		147		
All pathogens	2,750	167	70.4	147	121	191	344	47.2	553	12.8	71	51.3	355	130	7.41	6.91	443	21.7	4.99

Counts
- < 500
- 500 − <1k
- 2.5k − <7.5k

Cameroon

Deaths (count) *associated with* bacterial antimicrobial resistance by pathogen−drug combinations, 2019

	Resistance to 1+	AG	Aminopenicillin	Anti−pseudomonal	BL−BLI	CP	3GC	4GC	FQ	Mono INH	Macrolide	MDR in S. Typhi and Paratyphi	MDR excluding XDR in TB	Methicillin	PCN	Mono RIF	TMP−SMX	Vanco	XDR in TB
Acinetobacter baumannii	1,180	579		1,140	519	659	907	695	651										
Citrobacter spp.	129	39.8		52.8		14.6	114	86.7	53.6										
Enterobacter spp.	786	394		321		117		624	484								611		
Enterococcus faecalis	368								361									25	
Enterococcus faecium	458								455									118	
Other enterococci	231								230									12.9	
Escherichia coli	3,490	1,350	3,290		2,720	321	1,770		1,650								3,090		
Group A Streptococcus	98.8										98.8								
Group B Streptococcus	1,020								578		715				16.8				
Haemophilus influenzae	218		173				82.8												
Klebsiella pneumoniae	4,390	2,950			4,080	108	2,260		3,390								4,170		
Morganella spp.	5.06						1.28	2.85	3.84										
Mycobacterium tuberculosis	921									177			341			398			4.68
Proteus spp.	241	44.9	217				54.9		86.3								178		
Pseudomonas aeruginosa	1,530	686		773		688	1,040	572	854										
Salmonella Paratyphi	1.09								1.07			0.026							
Salmonella Typhi	1,150								310			1,020							
Non−typhoidal Salmonella	2.95								2.95										
Serratia spp.	361	133		61.9		25.5	307	270	95										
Shigella spp.	168								168										
Staphylococcus aureus	3,500								744		1,680			1,840			2,390	28.4	
Streptococcus pneumoniae	4,330				915	892	528		449		952				838		4,180		
All pathogens	24,600	6,180	3,680	2,350	8,230	2,830	7,060	2,250	10,600	177	3,440	1,020	341	1,840	855	398	14,600	185	4.68

Counts
- < 500
- 500 − <1k
- 1k − <2.5k
- 2.5k − <7.5k
- 7.5k − <15k
- 15k − <25k

Cameroon

Deaths (count) *attributable to* bacterial antimicrobial resistance by pathogen−drug combinations, 2019

	Resistance to 1+	AG	Aminopenicillin	Anti−pseudomonal	BL−BLI	CP	3GC	4GC	FQ	Mono INH	Macrolide	MDR in S. Typhi and Paratyphi	MDR excluding XDR in TB	Methicillin	PCN	Mono RIF	TMP−SMX	Vanco	XDR in TB
Acinetobacter baumannii	355	27.9		118	0.012	119	7.75	0.03	82.9										
Citrobacter spp.	37.8	2.05		9.81		3.23	6.77	9	6.9										
Enterobacter spp.	186	21.2		26.3		22.9		40.1	46.9								28.5		
Enterococcus faecalis	97.6								90.4									7.13	
Enterococcus faecium	116								87.8									27.9	
Other enterococci	47.4								44.9									2.51	
Escherichia coli	849	80.8	36.7		108	66.2	218		150								189		
Group A Streptococcus	10										9.89								
Group B Streptococcus	171								101		68.7				4.19				
Haemophilus influenzae	49.5		25.5				23.9												
Klebsiella pneumoniae	1,020	199			134	36	269		200								183		
Morganella spp.	1.19						0.066	0.478	0.644										
Mycobacterium tuberculosis	344									27.4			202			112			2.87
Proteus spp.	31.3	2.16	4.33				11.9		5.95								6.77		
Pseudomonas aeruginosa	381	18.4		76		117	66.4	10.4	93.3										
Salmonella Paratyphi	0.226								0.222			0.003							
Salmonella Typhi	175								51.4			125							
Non−typhoidal Salmonella	0.61								0.61										
Serratia spp.	96.3	7.69		13.2		7.1	18.5	40.5	9.47										
Shigella spp.	35.1								35.1										
Staphylococcus aureus	793								30		60.4			437			257	8.54	
Streptococcus pneumoniae	787				29.8	197	23.1		54.5		34.9				31.6		417		
All pathogens	5,590	359	66.6	243	272	567	645	100	1,090	26.6	170	125	202	437	35.7	112	1,080	46.1	2.87

Counts
- **< 500**
- **500 − <1k**
- **1k − <2.5k**
- **2.5k − <7.5k**

Cape Verde

Deaths (count) *associated with* bacterial antimicrobial resistance by pathogen−drug combinations, 2019

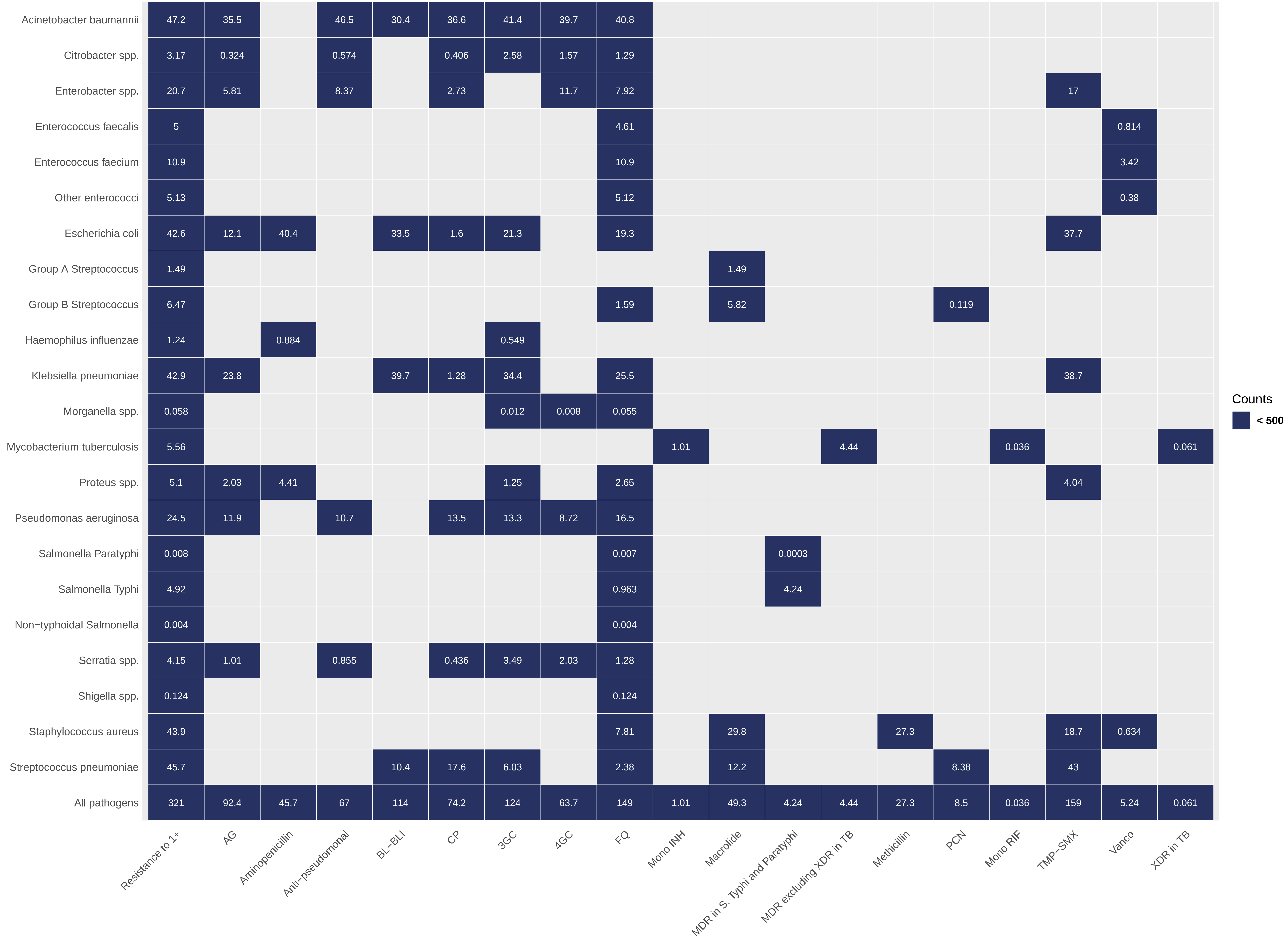

Cape Verde

Deaths (count) *attributable to* bacterial antimicrobial resistance by pathogen−drug combinations, 2019

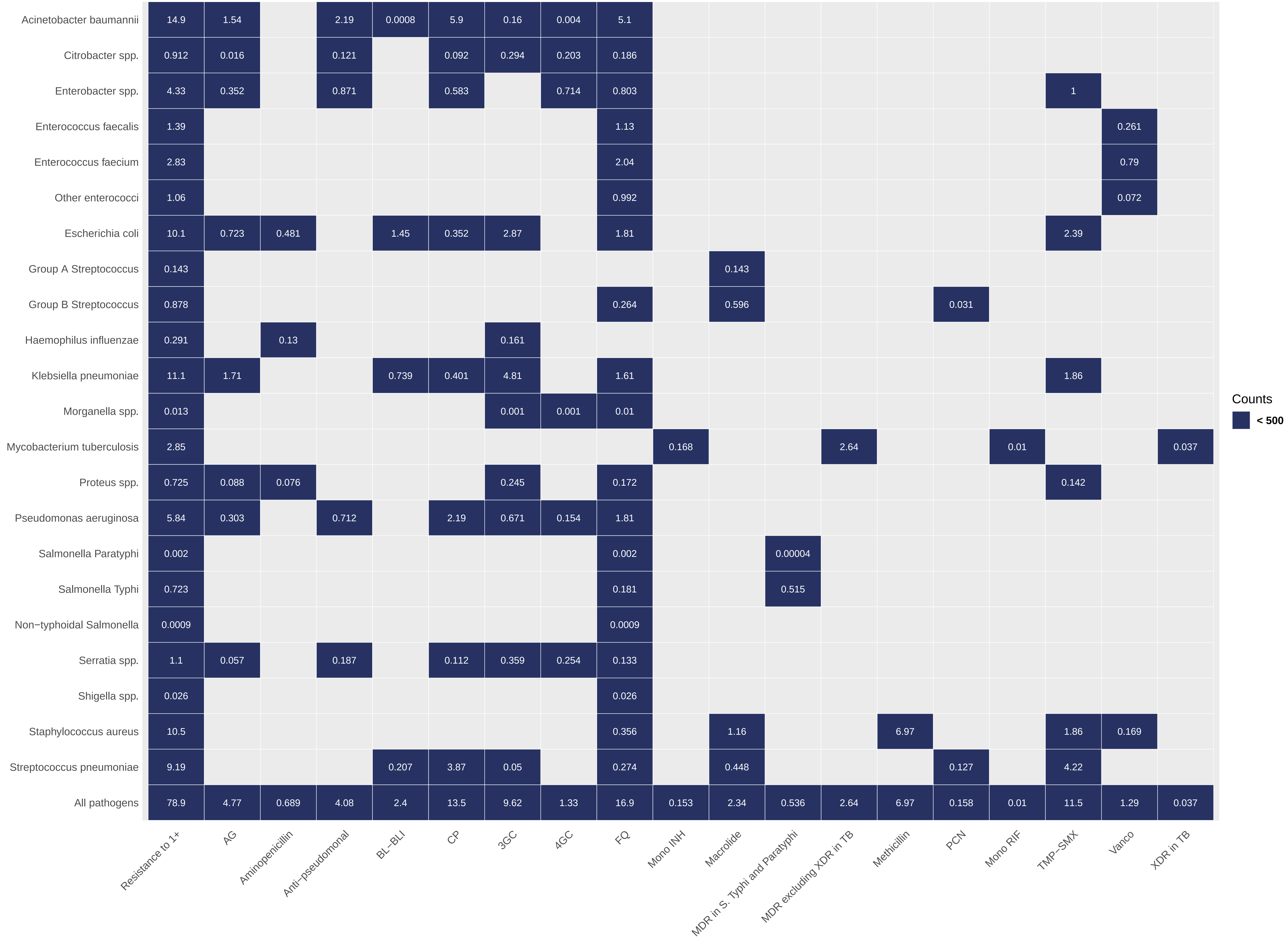

Central African Republic

Deaths (count) *associated with* bacterial antimicrobial resistance by pathogen−drug combinations, 2019

	Resistance to 1+	AG	Aminopenicillin	Anti−pseudomonal	BL−BLI	CP	3GC	4GC	FQ	Mono INH	Macrolide	MDR in S. Typhi and Paratyphi	MDR excluding XDR in TB	Methicillin	PCN	Mono RIF	TMP−SMX	Vanco	XDR in TB
Acinetobacter baumannii	346	89		326	225	88	249	237	150										
Citrobacter spp.	33.8	8.05		18.7		4.41	25.2	23.1	11.4										
Enterobacter spp.	234	83.8		75		53.4		205	115								161		
Enterococcus faecalis	124								123									8.43	
Enterococcus faecium	97.3								96.5									28.1	
Other enterococci	29.5								29.3									3.49	
Escherichia coli	1,090	576	918		628	146	375		567								853		
Group A Streptococcus	23.3										23.3								
Group B Streptococcus	769								531		488				22.1				
Haemophilus influenzae	259		248				98.5												
Klebsiella pneumoniae	1,380	1,190			1,070	48.9	1,020		1,160								1,170		
Morganella spp.	1.72						0.411	1.1	1.28										
Mycobacterium tuberculosis	1,110									782			287			36.7			3.86
Proteus spp.	61.6	11.2	57.5				16		16.7								40.1		
Pseudomonas aeruginosa	296	99		193		114	182	206	99.9										
Salmonella Paratyphi	0.012								0.01			0.002							
Salmonella Typhi	376								295			117							
Non−typhoidal Salmonella	1.34								1.34										
Serratia spp.	98.1	34		14.3		26.3	55.2	87.4	20.8										
Shigella spp.	1.84								1.84										
Staphylococcus aureus	964								721		621			255			719	13.1	
Streptococcus pneumoniae	2,190				1,490	331	216		1,890		1,520				1,490		1,810		
All pathogens	9,480	2,090	1,220	626	3,410	812	2,240	759	5,830	782	2,650	117	287	255	1,510	36.7	4,750	53.1	3.86

Counts
- < 500
- 500 − <1k
- 1k − <2.5k
- 2.5k − <7.5k
- 7.5k − <15k

Central African Republic

Deaths (count) *attributable to* bacterial antimicrobial resistance by pathogen−drug combinations, 2019

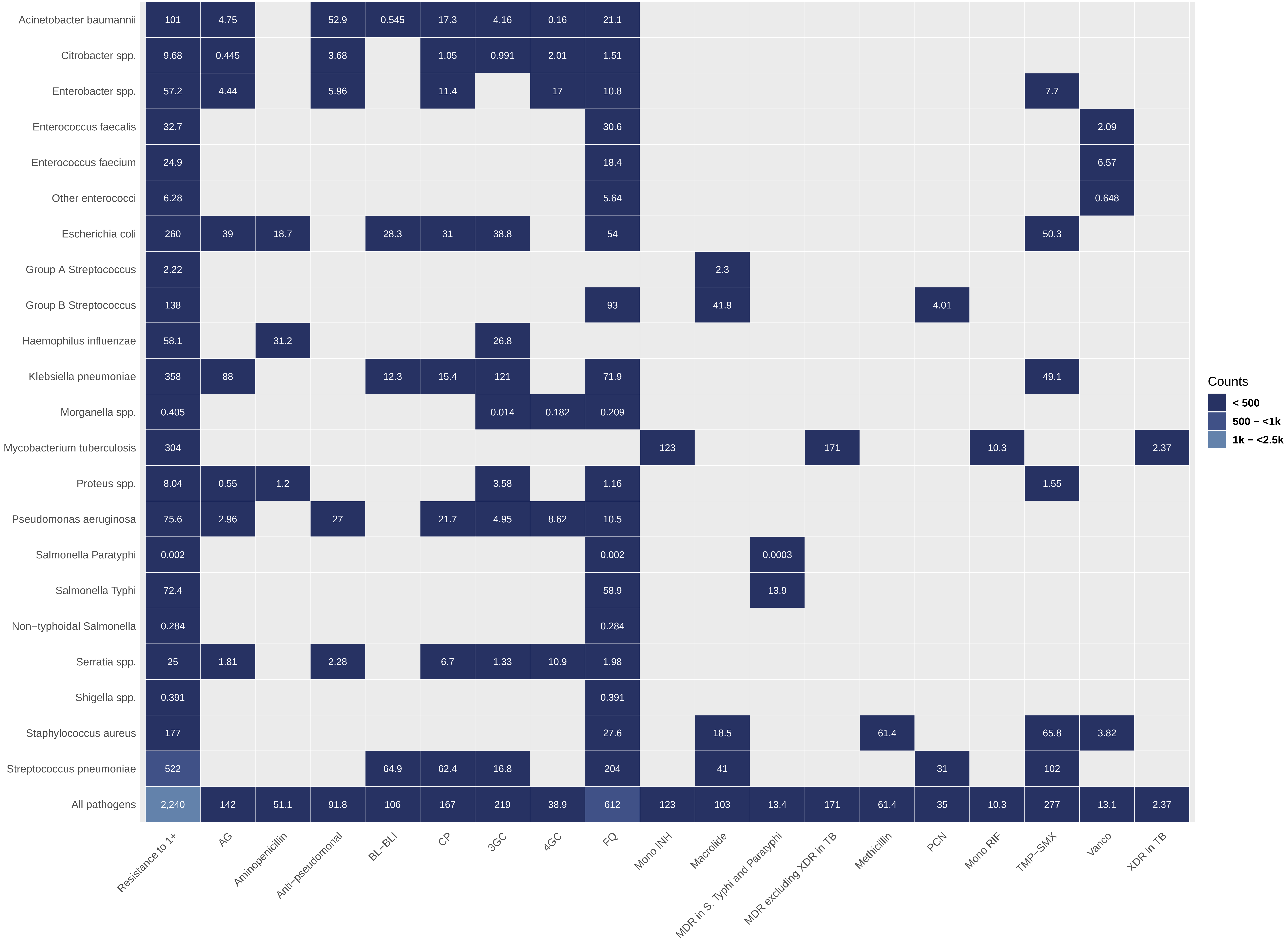

Chad

Deaths (count) *associated with* bacterial antimicrobial resistance by pathogen−drug combinations, 2019

	Resistance to 1+	AG	Aminopenicillin	Anti−pseudomonal	BL−BLI	CP	3GC	4GC	FQ	Mono INH	Macrolide	MDR in S. Typhi and Paratyphi	MDR excluding XDR in TB	Methicillin	PCN	Mono RIF	TMP−SMX	Vanco	XDR in TB
Acinetobacter baumannii	1,050	427		1,040	554	361	623	446	347										
Citrobacter spp.	242	97.8		103		94.4	214	212	88.8										
Enterobacter spp.	1,070	455		393		232		953	665								815		
Enterococcus faecalis	278								272									21.3	
Enterococcus faecium	272								270									70.5	
Other enterococci	162								161									9.28	
Escherichia coli	3,370	978	3,300		2,650	378	1,870		2,230								2,890		
Group A Streptococcus	66.1										66.1								
Group B Streptococcus	2,120								1,400		1,400				69.3				
Haemophilus influenzae	556		461				237												
Klebsiella pneumoniae	4,030	2,980			3,810	75.5	3,790		2,640								3,620		
Morganella spp.	2.95						0.731	1.88	2.13										
Mycobacterium tuberculosis	679									247			421			5.76			5.71
Proteus spp.	167	60.6	144				43.9		97.5								132		
Pseudomonas aeruginosa	942	440		534		292	607	540	437										
Salmonella Paratyphi	0.296								0.272			0.026							
Salmonella Typhi	372								135			248							
Non−typhoidal Salmonella	3.71								3.71										
Serratia spp.	450	244		62.7		21	392	378	122										
Shigella spp.	162								162										
Staphylococcus aureus	1,950								238		1,200			763			1,360	61.5	
Streptococcus pneumoniae	7,140				1,320	1,510	715		2,390		1,980				915		6,660		
All pathogens	25,100	5,680	3,910	2,130	8,330	2,960	8,490	2,530	11,700	247	4,640	248	421	763	985	5.76	15,500	163	5.71

Counts
- < 500
- 500 − <1k
- 1k − <2.5k
- 2.5k − <7.5k
- 7.5k − <15k
- 15k − <25k
- >=25k

Chad

Deaths (count) *attributable to* bacterial antimicrobial resistance by pathogen−drug combinations, 2019

	Resistance to 1+	AG	Aminopenicillin	Anti−pseudomonal	BL−BLI	CP	3GC	4GC	FQ	Mono INH	Macrolide	MDR in S. Typhi and Paratyphi	MDR excluding XDR in TB	Methicillin	PCN	Mono RIF	TMP−SMX	Vanco	XDR in TB
Acinetobacter baumannii	297	22.6		162	0.007	67.1	0.051	0.02	44.6										
Citrobacter spp.	73.5	5.35		13.2		21.1	5.35	18.2	10.5										
Enterobacter spp.	264	23.1		28.9		45.5		68.4	62.4								35.8		
Enterococcus faecalis	73.8								67.9									5.89	
Enterococcus faecium	68.5								52									16.5	
Other enterococci	33.4								31.5									1.87	
Escherichia coli	864	54.8	40.2		93.6	73.5	221		219								162		
Group A Streptococcus	6.01										6.34								
Group B Streptococcus	376								244		120				12.4				
Haemophilus influenzae	129		61.4				67.3												
Klebsiella pneumoniae	1,110	211			23.6	26.1	534		158								156		
Morganella spp.	0.693						0.028	0.317	0.348										
Mycobacterium tuberculosis	295									37.8			252			1.61			3.5
Proteus spp.	24.5	2.57	2.42				8.4		6.48								4.56		
Pseudomonas aeruginosa	233	12.2		73.8		49.9	28.4	21	47.1										
Salmonella Paratyphi	0.06								0.057			0.003							
Salmonella Typhi	60.4								27.8			32.8							
Non−typhoidal Salmonella	0.784								0.784										
Serratia spp.	120	14.3		12.9		5.97	14.6	60.7	11.9										
Shigella spp.	34.1								34.1										
Staphylococcus aureus	392								10.3		45			173			147	15.8	
Streptococcus pneumoniae	1,370				38.6	305	20.4		302		68.4				22.4		613		
All pathogens	5,820	346	104	291	156	594	900	169	1,330	33.9	240	32.3	252	173	34.8	1.61	1,120	40.1	3.5

Counts
- < 500
- 500 − <1k
- 1k − <2.5k
- 2.5k − <7.5k

Comoros

Deaths (count) *associated with* bacterial antimicrobial resistance by pathogen−drug combinations, 2019

	Resistance to 1+	AG	Aminopenicillin	Anti−pseudomonal	BL−BLI	CP	3GC	4GC	FQ	Mono INH	Macrolide	MDR in S. Typhi and Paratyphi	MDR excluding XDR in TB	Methicillin	PCN	Mono RIF	TMP−SMX	Vanco	XDR in TB
Acinetobacter baumannii	68.2	24.3		66.6	47.3	21.3	54.8	25.2	30.5										
Citrobacter spp.	7	2.64		3.14		1.32	6.39	5.2	4.16										
Enterobacter spp.	33.8	15.7		14		5.28		30.4	21.4								25.7		
Enterococcus faecalis	11.9								11.7									0.934	
Enterococcus faecium	13.8								13.6									3.15	
Other enterococci	6.04								6.02									0.389	
Escherichia coli	83.7	18.6	80.3		61.4	9.2	41.8		36.2								69.3		
Group A Streptococcus	3.43										3.43								
Group B Streptococcus	22.9								8.42		19.6				0.875				
Haemophilus influenzae	6.75		5.99				1.95												
Klebsiella pneumoniae	93.3	68.4			88.9	0.855	65.8		68.1								83.4		
Morganella spp.	0.233						0.068	0.138	0.172										
Mycobacterium tuberculosis	53.5									3.56			48.4			0.968			0.662
Proteus spp.	9.74	5.74	7.81				2.08		6.28								8.74		
Pseudomonas aeruginosa	30.5	12.3		10.2		7.47	24.5	10.9	12.4										
Salmonella Paratyphi	0.0006								0.0003			0.0003							
Salmonella Typhi	11.2								3.67			8.55							
Non−typhoidal Salmonella	0.038								0.038										
Serratia spp.	10.5	5.02		3.4		0.542	6.47	8.35	2.83										
Shigella spp.	0.942								0.942										
Staphylococcus aureus	62.9								17.2		33.2			21.7			44.9	1.26	
Streptococcus pneumoniae	114				16.5	33.8	16.2		19.7		14.3				40.3		109		
All pathogens	644	153	94.1	97.4	214	79.7	220	80.1	263	3.56	70.6	8.56	48.4	21.7	41.2	0.968	341	5.73	0.662

Counts
< 500
500 − <1k

Comoros

Deaths (count) *attributable to* bacterial antimicrobial resistance by pathogen−drug combinations, 2019

	Resistance to 1+	AG	Aminopenicillin	Anti−pseudomonal	BL−BLI	CP	3GC	4GC	FQ	Mono INH	Macrolide	MDR in S. Typhi and Paratyphi	MDR excluding XDR in TB	Methicillin	PCN	Mono RIF	TMP−SMX	Vanco	XDR in TB
Acinetobacter baumannii	20.1	1.28		10.7	0.014	3.7	0.351	0.002	4.03										
Citrobacter spp.	2.1	0.128		0.476		0.255	0.265	0.435	0.543										
Enterobacter spp.	8.23	0.798		1.15		1.04		2.14	1.99								1.13		
Enterococcus faecalis	3.17								2.91									0.257	
Enterococcus faecium	3.43								2.67									0.759	
Other enterococci	1.25								1.18									0.077	
Escherichia coli	20.5	1.12	1.29		2.73	2.01	5.52		3.46								4.36		
Group A Streptococcus	0.317										0.316								
Group B Streptococcus	3.42								1.39		1.83				0.163				
Haemophilus influenzae	1.46		0.904				0.554												
Klebsiella pneumoniae	23.3	4.73			2.21	0.412	8.31		4.04								3.56		
Morganella spp.	0.055						0.004	0.024	0.028										
Mycobacterium tuberculosis	29.4									0.618			28.1			0.272			0.406
Proteus spp.	1.42	0.25	0.118				0.37		0.395								0.287		
Pseudomonas aeruginosa	7.71	0.349		1.38		1.3	2.73	0.537	1.4										
Salmonella Paratyphi	0.0001								0.00006			0.00004							
Salmonella Typhi	1.75								0.687			1.05							
Non−typhoidal Salmonella	0.008								0.008										
Serratia spp.	2.55	0.277		0.662		0.167	0.183	0.983	0.278										
Shigella spp.	0.195								0.195										
Staphylococcus aureus	12								0.679		1.23			4.79			5	0.331	
Streptococcus pneumoniae	22.9				0.288	7.18	0.339		2.28		0.527				1.89		10.4		
All pathogens	165	8.92	2.31	14.4	5.25	16.1	18.6	4.12	28.2	0.56	3.96	1.04	28.1	4.79	2.05	0.272	24.7	1.42	0.406

Counts

< 500

Congo (Brazzaville)

Deaths (count) *associated with* bacterial antimicrobial resistance by pathogen−drug combinations, 2019

	Resistance to 1+	AG	Aminopenicillin	Anti−pseudomonal	BL−BLI	CP	3GC	4GC	FQ	Mono INH	Macrolide	MDR in S. Typhi and Paratyphi	MDR excluding XDR in TB	Methicillin	PCN	Mono RIF	TMP−SMX	Vanco	XDR in TB
Acinetobacter baumannii	251	104		243	147	136	223	153	157										
Citrobacter spp.	22.8	6.27		11		2.72	19	15.3	10.7										
Enterobacter spp.	141	55.9		60.6		29.1		114	72.7								108		
Enterococcus faecalis	77.6								76.1									6.52	
Enterococcus faecium	91								90.5									22.5	
Other enterococci	38.7								38.7									2.09	
Escherichia coli	521	173	469		378	71.5	266		279								451		
Group A Streptococcus	15.3										15.3								
Group B Streptococcus	177								99.6		129				8.02				
Haemophilus influenzae	31.5		26.6				10.7												
Klebsiella pneumoniae	629	448			574	18.8	510		496								571		
Morganella spp.	1.19						0.304	0.727	0.895										
Mycobacterium tuberculosis	164									65			91.9			5.96			1.27
Proteus spp.	51.5	14.2	46				12.8		23.9								40.5		
Pseudomonas aeruginosa	218	90.5		120		98.2	150	96.4	110										
Salmonella Paratyphi	0.014								0.014			0.0004							
Salmonella Typhi	82								56.8			35							
Non−typhoidal Salmonella	0.278								0.278										
Serratia spp.	50.9	21.6		9.16		4.3	29.3	40.7	11.8										
Shigella spp.	3.42								3.42										
Staphylococcus aureus	491								256		209			276			323	5.3	
Streptococcus pneumoniae	563				202	122	67.9		131		178				200		529		
All pathogens	3,620	914	541	444	1,300	482	1,290	420	1,910	65	531	35	91.9	276	208	5.96	2,020	36.4	1.27

Counts
- < 500
- 500 − <1k
- 1k − <2.5k
- 2.5k − <7.5k

Congo (Brazzaville)

Deaths (count) *attributable to* bacterial antimicrobial resistance by pathogen−drug combinations, 2019

	Resistance to 1+	AG	Aminopenicillin	Anti−pseudomonal	BL−BLI	CP	3GC	4GC	FQ	Mono INH	Macrolide	MDR in S. Typhi and Paratyphi	MDR excluding XDR in TB	Methicillin	PCN	Mono RIF	TMP−SMX	Vanco	XDR in TB
Acinetobacter baumannii	76.8	4.65		26.8	0.003	23.1	1.97	0.006	20.1										
Citrobacter spp.	6.65	0.316		1.99		0.592	0.938	1.37	1.44										
Enterobacter spp.	33.6	3.02		5.16		5.96		7.31	6.91								5.21		
Enterococcus faecalis	20.7								18.9									1.76	
Enterococcus faecium	22.8								17.5									5.3	
Other enterococci	7.95								7.55									0.402	
Escherichia coli	130	10.1	5.31		14.3	15	31.5		26.4								27.8		
Group A Streptococcus	1.47										1.47								
Group B Streptococcus	30.1								17.1		11.8				1.4				
Haemophilus influenzae	6.99		3.93				3.06												
Klebsiella pneumoniae	165	30.9			8.49	5.94	64.1		30.3								24.9		
Morganella spp.	0.281						0.013	0.121	0.147										
Mycobacterium tuberculosis	68.4									10.6			55.3			1.68			0.776
Proteus spp.	7.15	0.631	0.851				2.59		1.59								1.48		
Pseudomonas aeruginosa	55.4	2.46		12.9		17.3	8.75	2.03	11.9										
Salmonella Paratyphi	0.003								0.003			0.00006							
Salmonella Typhi	15.1								11			3.96							
Non−typhoidal Salmonella	0.058								0.058										
Serratia spp.	12.3	1.19		1.83		1.24	1.15	5.67	1.17										
Shigella spp.	0.716								0.716										
Staphylococcus aureus	115								10.4		6.89			63.7			32.6	1.52	
Streptococcus pneumoniae	113				7.79	25.2	2.97		15.3		5.78				9.37		46.4		
All pathogens	890	53.5	10.1	48.7	30.6	94.2	117	16.5	199	11.1	25.9	4.05	55.3	63.7	10.8	1.68	139	8.99	0.776

Counts
- **< 500**
- **500 − <1k**

Cote d'Ivoire

Deaths (count) *associated with* bacterial antimicrobial resistance by pathogen−drug combinations, 2019

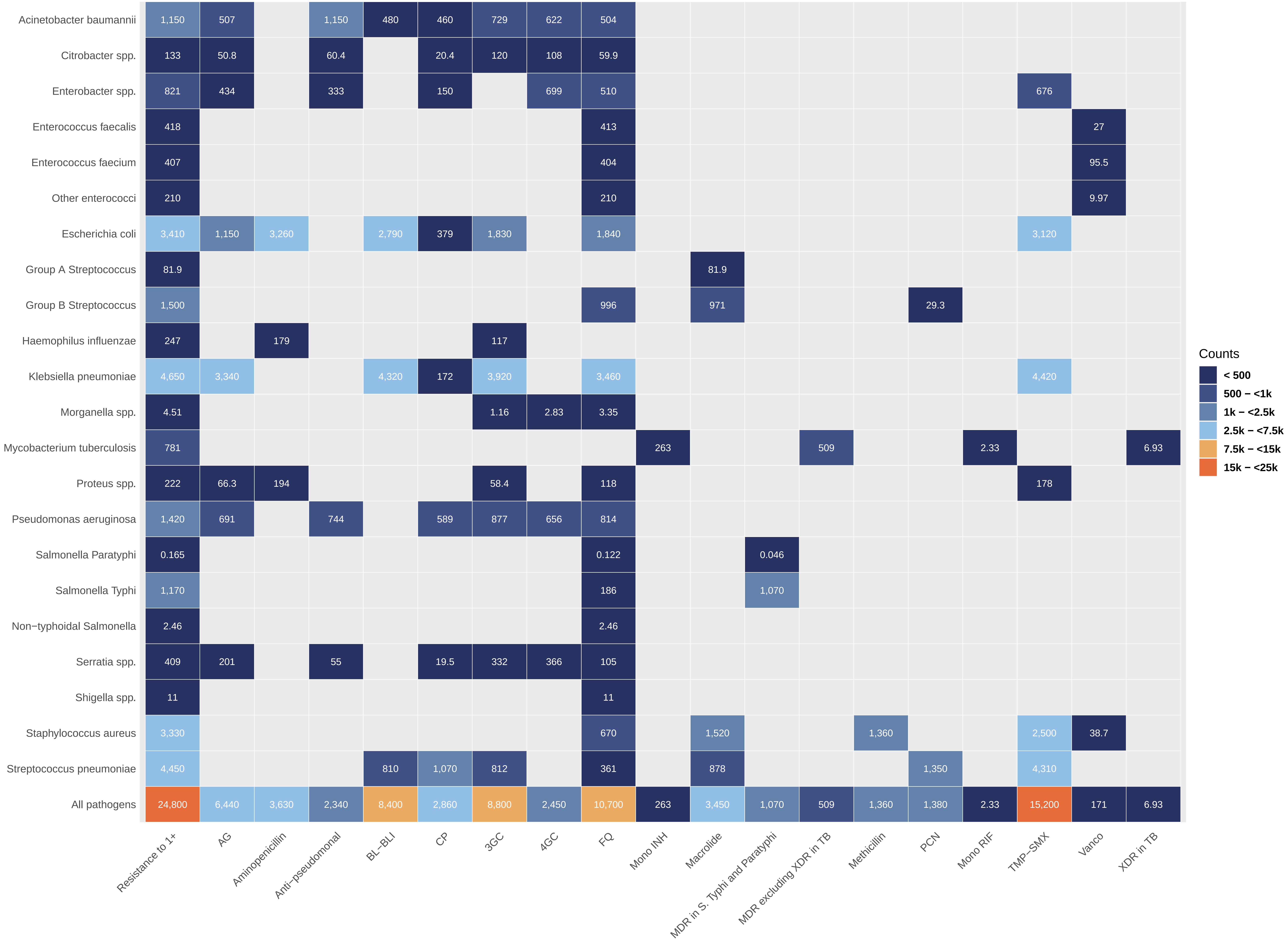

Cote d'Ivoire

Deaths (count) *attributable to* bacterial antimicrobial resistance by pathogen−drug combinations, 2019

	Resistance to 1+	AG	Aminopenicillin	Anti−pseudomonal	BL−BLI	CP	3GC	4GC	FQ	Mono INH	Macrolide	MDR in S. Typhi and Paratyphi	MDR excluding XDR in TB	Methicillin	PCN	Mono RIF	TMP−SMX	Vanco	XDR in TB
Acinetobacter baumannii	332	25.1		160	0.007	81.4	0.101	0.023	64.4										
Citrobacter spp.	39.5	2.62		10.2		4.34	4.14	10.6	7.54										
Enterobacter spp.	199	22.9		25		28.7		44.6	47.2								30.6		
Enterococcus faecalis	110								103									7.21	
Enterococcus faecium	101								78.7									22.4	
Other enterococci	43								41									1.99	
Escherichia coli	854	67.1	29.7		100	76.3	220		172								188		
Group A Streptococcus	7.98										7.89								
Group B Streptococcus	265								175		85.8				6.04				
Haemophilus influenzae	58.6		24.8				33.8												
Klebsiella pneumoniae	1,230	233			52.1	48.9	495		209								196		
Morganella spp.	1.06						0.046	0.471	0.547										
Mycobacterium tuberculosis	349									44.6			303			0.656			4.24
Proteus spp.	32	2.88	3.44				11.5		7.86								6.47		
Pseudomonas aeruginosa	348	19.3		80.5		98.1	43.7	16.7	89.7										
Salmonella Paratyphi	0.032								0.026			0.006							
Salmonella Typhi	169								32.7			133							
Non−typhoidal Salmonella	0.521								0.521										
Serratia spp.	108	11.5		11.6		5.63	8.35	60.3	10.4										
Shigella spp.	2.34								2.34										
Staphylococcus aureus	683								26.1		55.1			311			281	10.5	
Streptococcus pneumoniae	855				18.6	236	40.9		41.3		32.4				60.3		425		
All pathogens	5,790	384	57.8	288	171	580	857	133	1,110	40.4	179	144	303	311	66.3	0.656	1,130	42.1	4.24

Counts
- **< 500**
- **500 − <1k**
- **1k − <2.5k**
- **2.5k − <7.5k**

DR Congo

Deaths (count) *associated with* bacterial antimicrobial resistance by pathogen−drug combinations, 2019

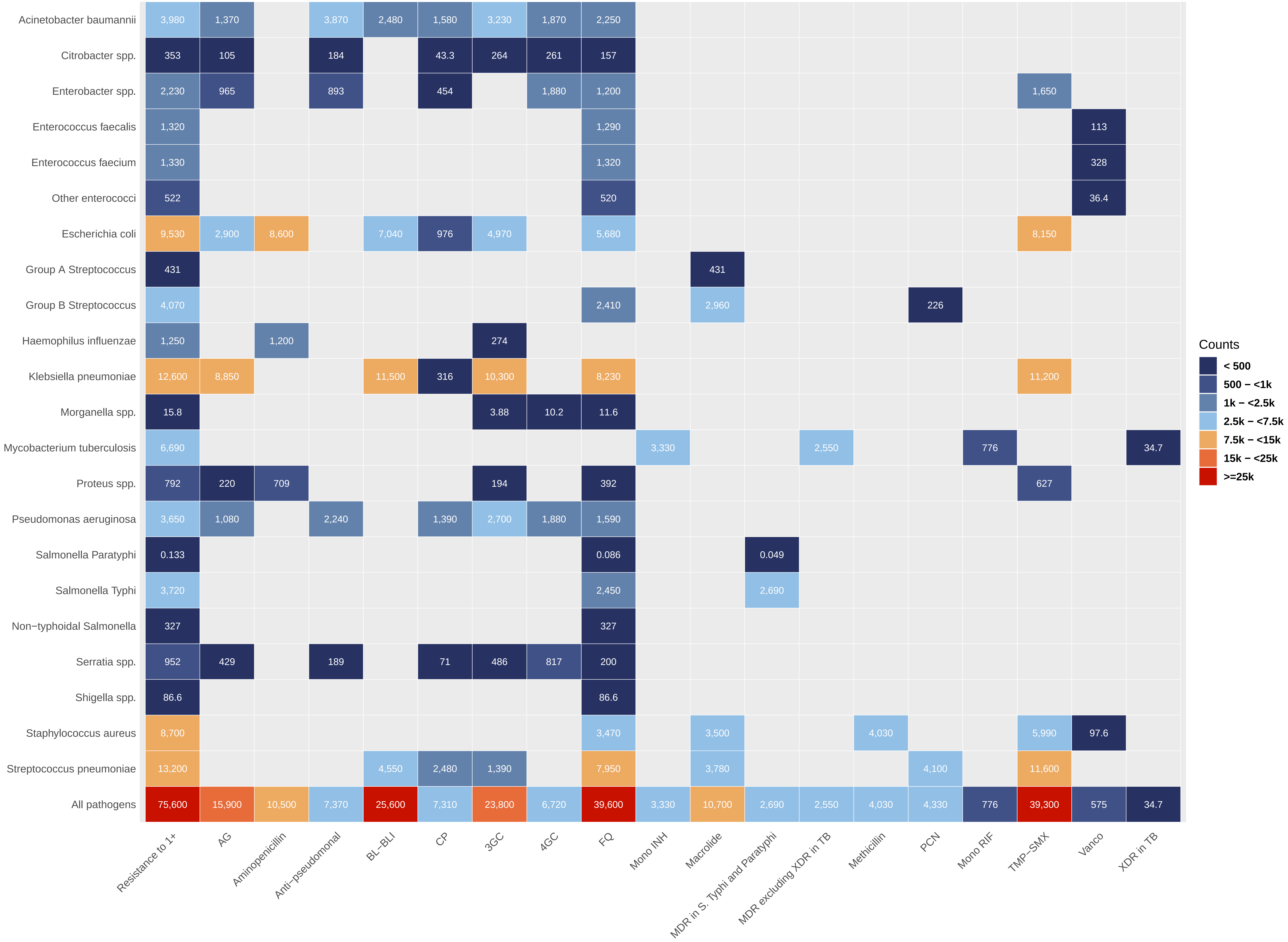

DR Congo

Deaths (count) *attributable to* bacterial antimicrobial resistance by pathogen−drug combinations, 2019

	Resistance to 1+	AG	Aminopenicillin	Anti−pseudomonal	BL−BLI	CP	3GC	4GC	FQ	Mono INH	Macrolide	MDR in S. Typhi and Paratyphi	MDR excluding XDR in TB	Methicillin	PCN	Mono RIF	TMP−SMX	Vanco	XDR in TB
Acinetobacter baumannii	1,190	66		530	0.314	268	25.5	0.087	298										
Citrobacter spp.	101	5.57		33.1		9.82	9.33	22.7	21										
Enterobacter spp.	538	51.7		73		92.4		130	114								76.6		
Enterococcus faecalis	350								320									30	
Enterococcus faecium	332								255									77	
Other enterococci	108								101									7.05	
Escherichia coli	2,380	165	91.1		268	200	604		557								493		
Group A Streptococcus	39.5										41.6								
Group B Streptococcus	706								409		259				38				
Haemophilus influenzae	259		184				75.7												
Klebsiella pneumoniae	3,280	629			184	106	1,360		497								506		
Morganella spp.	3.72						0.141	1.7	1.88										
Mycobacterium tuberculosis	2,280									538			1,510			217			21.3
Proteus spp.	110	9.72	13				38.9		26								22.5		
Pseudomonas aeruginosa	958	30.5		299		254	154	48.1	173										
Salmonella Paratyphi	0.024								0.018			0.006							
Salmonella Typhi	690								411			279							
Non−typhoidal Salmonella	68.9								68.9										
Serratia spp.	224	23.8		36.5		21.1	10.9	112	19.6										
Shigella spp.	17.9								17.9										
Staphylococcus aureus	1,880								144		123			931			652	29.4	
Streptococcus pneumoniae	2,810				161	453	61.2		979		114				161		884		
All pathogens	18,300	980	288	971	614	1,400	2,340	314	4,410	528	537	278	1,510	931	199	217	2,640	144	21.3

Counts
- < 500
- 500 − <1k
- 1k − <2.5k
- 2.5k − <7.5k
- 15k − <25k

Equatorial Guinea

Deaths (count) *associated with* bacterial antimicrobial resistance by pathogen−drug combinations, 2019

	Resistance to 1+	AG	Aminopenicillin	Anti−pseudomonal	BL−BLI	CP	3GC	4GC	FQ	Mono INH	Macrolide	MDR in S. Typhi and Paratyphi	MDR excluding XDR in TB	Methicillin	PCN	Mono RIF	TMP−SMX	Vanco	XDR in TB
Acinetobacter baumannii	61.6	34.6		60.1	45.4	42.6	56.8	45.5	52.7										
Citrobacter spp.	8.79	0.706		3.33		2.11	6.69	4.99	4.34										
Enterobacter spp.	47.2	10.5		20.9		9.22		28.6	20.9								37.6		
Enterococcus faecalis	11.1								10.7									1.01	
Enterococcus faecium	15.8								15.7									4.22	
Other enterococci	11.3								11.2									0.563	
Escherichia coli	73.8	26.8	68		51.4	5.83	38.1		41.6								62.3		
Group A Streptococcus	2.64										2.64								
Group B Streptococcus	33.2								14.1		27				1.42				
Haemophilus influenzae	4.24		3.02				1.96												
Klebsiella pneumoniae	90.2	57.3			83.6	2.22	60.8		68.3								77.8		
Morganella spp.	0.136						0.034	0.028	0.124										
Mycobacterium tuberculosis	21.6									9.92			10.9			0.7			0.148
Proteus spp.	8.3	2.69	7.37				1.73		3.69								6.53		
Pseudomonas aeruginosa	38.7	15.5		20.3		21	22.7	15.1	23.4										
Salmonella Paratyphi	0.003								0.003			0.0003							
Salmonella Typhi	9.6								6.56			4.42							
Non−typhoidal Salmonella	0.088								0.088										
Serratia spp.	10.9	5.05		2.49		1.36	5.6	7.22	3.12										
Shigella spp.	0.192								0.192										
Staphylococcus aureus	59.8								38.2		17.4			36.2			36.2	0.685	
Streptococcus pneumoniae	114				38.3	25.7	14.6		19.4		37.9				56.6		105		
All pathogens	624	153	78.4	107	219	110	209	101	334	9.92	85	4.42	10.9	36.2	58	0.7	325	6.47	0.148

Counts
- < 500
- 500 − <1k

Equatorial Guinea

Deaths (count) *attributable to* bacterial antimicrobial resistance by pathogen−drug combinations, 2019

	Resistance to 1+	AG	Aminopenicillin	Anti−pseudomonal	BL−BLI	CP	3GC	4GC	FQ	Mono INH	Macrolide	MDR in S. Typhi and Paratyphi	MDR excluding XDR in TB	Methicillin	PCN	Mono RIF	TMP−SMX	Vanco	XDR in TB
Acinetobacter baumannii	19.3	1.45		3.75	0.004	6.86	0.419	0.002	6.84										
Citrobacter spp.	2.57	0.034		0.539		0.474	0.45	0.437	0.636										
Enterobacter spp.	10.5	0.617		2.03		1.94		1.58	2.16								2.15		
Enterococcus faecalis	2.98								2.68									0.299	
Enterococcus faecium	3.99								3.01									0.986	
Other enterococci	2.31								2.2									0.108	
Escherichia coli	18.1	1.56	0.973		2.02	1.2	4.74		3.94								3.71		
Group A Streptococcus	0.249										0.26								
Group B Streptococcus	5.18								2.39		2.55				0.257				
Haemophilus influenzae	1		0.432				0.57												
Klebsiella pneumoniae	22.3	3.95			2.25	0.773	7.68		4.23								3.45		
Morganella spp.	0.031						0.004	0.005	0.023										
Mycobacterium tuberculosis	8.29									1.64			6.43			0.196			0.091
Proteus spp.	1.1	0.118	0.147				0.355		0.244								0.236		
Pseudomonas aeruginosa	9.55	0.389		1.71		3.58	1.06	0.242	2.56										
Salmonella Paratyphi	0.0006								0.0006			0.00004							
Salmonella Typhi	1.75								1.26			0.493							
Non−typhoidal Salmonella	0.019								0.019										
Serratia spp.	2.54	0.287		0.466		0.358	0.262	0.851	0.318										
Shigella spp.	0.04								0.04										
Staphylococcus aureus	14.6								1.65		0.555			8.58			3.6	0.202	
Streptococcus pneumoniae	23.5				1.44	5.37	0.659		2.18		1.25				3.49		9.1		
All pathogens	150	8.44	1.55	8.49	5.72	20.6	16.2	3.11	36.4	1.57	4.62	0.503	6.43	8.58	3.74	0.196	22.3	1.59	0.091

Counts
< 500

Eritrea

Deaths (count) *associated with* bacterial antimicrobial resistance by pathogen−drug combinations, 2019

	Resistance to 1+	AG	Aminopenicillin	Anti−pseudomonal	BL−BLI	CP	3GC	4GC	FQ	Mono INH	Macrolide	MDR in S. Typhi and Paratyphi	MDR excluding XDR in TB	Methicillin	PCN	Mono RIF	TMP−SMX	Vanco	XDR in TB
Acinetobacter baumannii	386	123		361	274	103	322	153	156										
Citrobacter spp.	28.4	12		13.7		5.47	25.1	21.5	16.9										
Enterobacter spp.	173	80.2		69.9		29.4		160	92.7								130		
Enterococcus faecalis	107								105									8.19	
Enterococcus faecium	110								109									25.3	
Other enterococci	27.8								27.7									1.58	
Escherichia coli	885	209	856		663	85.9	450		449								720		
Group A Streptococcus	32.9										32.9								
Group B Streptococcus	237								113		193				9.14				
Haemophilus influenzae	103		98.9				23.2												
Klebsiella pneumoniae	1,090	835			1,040	12.9	772		718								986		
Morganella spp.	1.27						0.368	0.795	0.927										
Mycobacterium tuberculosis	642									179			391			66.3			5.32
Proteus spp.	69.2	29.3	56.9				14.8		45.3								62.1		
Pseudomonas aeruginosa	299	108		101		62.4	257	127	88.7										
Salmonella Paratyphi	0.009								0.002			0.007							
Salmonella Typhi	255								133			154							
Non−typhoidal Salmonella	0.183								0.183										
Serratia spp.	73.8	42.7		25.1		3.59	51.1	53.2	19.3										
Shigella spp.	30.6								30.6										
Staphylococcus aureus	954								242		478			259			716	11.6	
Streptococcus pneumoniae	1,100				176	244	144		548		124				219		1,010		
All pathogens	6,610	1,440	1,010	571	2,160	547	2,060	516	2,900	179	827	154	391	259	228	66.3	3,630	46.6	5.32

Counts
- **< 500**
- **500 − <1k**
- **1k − <2.5k**
- **2.5k − <7.5k**

Eritrea

Deaths (count) *attributable to* bacterial antimicrobial resistance by pathogen−drug combinations, 2019

	Resistance to 1+	AG	Aminopenicillin	Anti−pseudomonal	BL−BLI	CP	3GC	4GC	FQ	Mono INH	Macrolide	MDR in S. Typhi and Paratyphi	MDR excluding XDR in TB	Methicillin	PCN	Mono RIF	TMP−SMX	Vanco	XDR in TB
Acinetobacter baumannii	114	6.41		60.5	0.578	18.7	6.15	0.008	21.1										
Citrobacter spp.	8.49	0.605		2.05		1.06	0.901	1.69	2.2										
Enterobacter spp.	42.3	4.23		5.98		5.93		12.2	8.15								5.72		
Enterococcus faecalis	28.3								26.1									2.19	
Enterococcus faecium	27.4								21.3									6.11	
Other enterococci	5.77								5.45									0.326	
Escherichia coli	218	12.3	13.1		28.9	18.2	58.3		43.6								43.6		
Group A Streptococcus	3.11										3.09								
Group B Streptococcus	38								18.8		16.5				1.69				
Haemophilus influenzae	21.4		15				6.39												
Klebsiella pneumoniae	274	59			26.1	5.61	98.3		42.4								42.5		
Morganella spp.	0.303						0.017	0.135	0.151										
Mycobacterium tuberculosis	281									28.7			230			18.6			3.26
Proteus spp.	9.99	1.24	0.918				2.72		2.97								2.13		
Pseudomonas aeruginosa	77.5	3.34		15.3		11.5	29.4	8.12	9.79										
Salmonella Paratyphi	0.001								0.0004			0.001							
Salmonella Typhi	43.9								25.6			17.9							
Non−typhoidal Salmonella	0.038								0.038										
Serratia spp.	18.1	2.5		4.84		1.08	1.92	5.93	1.87										
Shigella spp.	6.42								6.42										
Staphylococcus aureus	169								9.64		17.8			56.4			82.1	3.23	
Streptococcus pneumoniae	226				3.95	47.5	4.98		69.5		4.5				6.96		88.4		
All pathogens	1,610	89.7	29	88.7	59.5	110	209	28.1	315	29.8	42.8	17.5	230	56.4	8.65	18.6	265	11.9	3.26

Counts
- < 500
- 1k − <2.5k

Ethiopia

Deaths (count) *associated with* bacterial antimicrobial resistance by pathogen−drug combinations, 2019

	Resistance to 1+	AG	Aminopenicillin	Anti−pseudomonal	BL−BLI	CP	3GC	4GC	FQ	Mono INH	Macrolide	MDR in S. Typhi and Paratyphi	MDR excluding XDR in TB	Methicillin	PCN	Mono RIF	TMP−SMX	Vanco	XDR in TB
Acinetobacter baumannii	4,120	1,490		4,080	3,910	1,400	3,940	3,870	2,770										
Citrobacter spp.	643	200		284		111	544	384	395										
Enterobacter spp.	3,460	1,520		1,310		393		3,060	1,850								2,550		
Enterococcus faecalis	1,660								1,630									115	
Enterococcus faecium	1,820								1,800									448	
Other enterococci	773								770									60.1	
Escherichia coli	13,100	1,950	12,300		8,240	753	8,540		8,280								11,200		
Group A Streptococcus	443										443								
Group B Streptococcus	4,710								1,750		4,090				135				
Haemophilus influenzae	973		919				182												
Klebsiella pneumoniae	15,800	11,500			15,300	5,810	15,400		8,990								14,000		
Morganella spp.	18.3						4.38	10.8	13.5										
Mycobacterium tuberculosis	2,690									329			2,050			279			27.7
Proteus spp.	1,100	475	936				194		618								962		
Pseudomonas aeruginosa	3,950	1,180		1,600		1,080	3,220	1,370	1,670										
Salmonella Paratyphi	0.108								0.032			0.065							
Salmonella Typhi	2,520								1,490			1,220							
Non−typhoidal Salmonella	475								475										
Serratia spp.	1,100	463		407		57.9	623	691	301										
Shigella spp.	714								714										
Staphylococcus aureus	10,200								2,210		6,560			5,380			6,200	113	
Streptococcus pneumoniae	15,000				2,520	3,710	1,960		2,380		1,840				3,650		14,600		
All pathogens	85,300	18,700	14,100	7,690	30,000	13,300	34,600	9,390	38,100	329	12,900	1,220	2,050	5,380	3,780	279	49,500	737	27.7

Counts
- < 500
- 500 − <1k
- 1k − <2.5k
- 2.5k − <7.5k
- 7.5k − <15k
- 15k − <25k
- >=25k

Ethiopia

Deaths (count) *attributable to* bacterial antimicrobial resistance by pathogen−drug combinations, 2019

	Resistance to 1+	AG	Aminopenicillin	Anti−pseudomonal	BL−BLI	CP	3GC	4GC	FQ	Mono INH	Macrolide	MDR in S. Typhi and Paratyphi	MDR excluding XDR in TB	Methicillin	PCN	Mono RIF	TMP−SMX	Vanco	XDR in TB
Acinetobacter baumannii	1,270	67.1		591	1.58	225	7.36	0.853	376										
Citrobacter spp.	190	9.27		44.5		20.9	30.5	29.6	54.7										
Enterobacter spp.	816	79.9		120		83.4		246	169								119		
Enterococcus faecalis	440								407									32.2	
Enterococcus faecium	456								349									107	
Other enterococci	161								149									11.5	
Escherichia coli	3,360	111	186		241	155	1,180		821								660		
Group A Streptococcus	44.1										40.5								
Group B Streptococcus	701								287		390				26.2				
Haemophilus influenzae	199		148				51.8												
Klebsiella pneumoniae	4,960	870			46.1	1,150	1,700		565								627		
Morganella spp.	4.29						0.201	1.83	2.26										
Mycobacterium tuberculosis	1,360									54.8			1,210			78			17
Proteus spp.	149	20.4	16.3				37.6		40.3								34.5		
Pseudomonas aeruginosa	1,030	33.8		221		193	342	49.7	191										
Salmonella Paratyphi	0.018								0.009			0.009							
Salmonella Typhi	446								295			145							
Non−typhoidal Salmonella	99.4								99.4										
Serratia spp.	259	26.5		83.1		18.2	24.8	75.5	30.8										
Shigella spp.	148								148										
Staphylococcus aureus	2,270								88.6		243			1,270			637	30.3	
Streptococcus pneumoniae	2,860				63.2	806	59.4		281		71.2				145		1,440		
All pathogens	21,200	1,220	350	1,060	352	2,650	3,440	403	4,360	52.8	742	154	1,210	1,270	171	78	3,510	181	17

Counts
- < 500
- 500 − <1k
- 1k − <2.5k
- 2.5k − <7.5k
- 15k − <25k

Gabon

Deaths (count) *associated with* bacterial antimicrobial resistance by pathogen−drug combinations, 2019

	Resistance to 1+	AG	Aminopenicillin	Anti−pseudomonal	BL−BLI	CP	3GC	4GC	FQ	Mono INH	Macrolide	MDR in S. Typhi and Paratyphi	MDR excluding XDR in TB	Methicillin	PCN	Mono RIF	TMP−SMX	Vanco	XDR in TB
Acinetobacter baumannii	131	62.9		124	86.9	73.4	117	99	102										
Citrobacter spp.	10.1	2.78		3.92		1.13	7.91	4.25	4.54										
Enterobacter spp.	61.2	17.5		21.5		10.5		38.2	30.1								47.2		
Enterococcus faecalis	23.9								23.5									1.58	
Enterococcus faecium	32.1								31.9									8.23	
Other enterococci	16.2								16.2									1.07	
Escherichia coli	142	70.3	132		98.6	14	63.3		69.1								115		
Group A Streptococcus	4.46										4.46								
Group B Streptococcus	30.2								10.4		25.4				0.982				
Haemophilus influenzae	7.37		6.44				2.02												
Klebsiella pneumoniae	152	90.5			133	3.6	112		117								134		
Morganella spp.	0.399						0.1	0.103	0.355										
Mycobacterium tuberculosis	45.1									13.1			30.5			1.19			0.417
Proteus spp.	16.4	0.718	14.4				3.49		5.04								12		
Pseudomonas aeruginosa	61.3	22.5		32.6		30.8	34.8	24.7	36.9										
Salmonella Paratyphi	0.003								0.003			0.00009							
Salmonella Typhi	9.3								6.3			4.06							
Non−typhoidal Salmonella	0.069								0.069										
Serratia spp.	15.6	5.64		2.69		4.46	8.11	11.8	3.7										
Shigella spp.	0.485								0.485										
Staphylococcus aureus	104								60.5		48.9			76.5			38.9	3.54	
Streptococcus pneumoniae	161				57.8	32.5	19.8		28.1		59				56.5		147		
All pathogens	1,020	273	153	185	377	170	369	178	546	13.1	138	4.06	30.5	76.5	57.5	1.19	495	14.4	0.417

Counts
- < 500
- 500 − <1k
- 1k − <2.5k

Gabon

Deaths (count) *attributable to* bacterial antimicrobial resistance by pathogen−drug combinations, 2019

	Resistance to 1+	AG	Aminopenicillin	Anti−pseudomonal	BL−BLI	CP	3GC	4GC	FQ	Mono INH	Macrolide	MDR in S. Typhi and Paratyphi	MDR excluding XDR in TB	Methicillin	PCN	Mono RIF	TMP−SMX	Vanco	XDR in TB
Acinetobacter baumannii	40.5	2.77		10.6	0.041	11.9	1.89	0.018	13.3										
Citrobacter spp.	2.9	0.15		0.755		0.242	0.691	0.388	0.664										
Enterobacter spp.	13.6	1		1.98		2.19		2.56	3.21								2.62		
Enterococcus faecalis	6.32								5.88									0.439	
Enterococcus faecium	8.09								6.15									1.94	
Other enterococci	3.35								3.14									0.205	
Escherichia coli	34.1	4.46	2.12		4.28	2.91	7.41		6.26								6.71		
Group A Streptococcus	0.4										0.425								
Group B Streptococcus	4.45								1.78		2.28				0.192				
Haemophilus influenzae	1.59		1.01				0.584												
Klebsiella pneumoniae	38.3	6.26			2.67	1.29	14.4		7.46								6.22		
Morganella spp.	0.092						0.01	0.017	0.064										
Mycobacterium tuberculosis	20.8									2.04			18.1			0.335			0.255
Proteus spp.	2.02	0.048	0.299				0.797		0.371								0.502		
Pseudomonas aeruginosa	15.1	0.632		3.12		5.28	1.55	0.441	4.1										
Salmonella Paratyphi	0.0007								0.0007			0.00001							
Salmonella Typhi	1.7								1.23			0.492							
Non−typhoidal Salmonella	0.014								0.014										
Serratia spp.	3.93	0.288		0.44		1.15	0.343	1.31	0.368										
Shigella spp.	0.101								0.101										
Staphylococcus aureus	28.1								2.64		1.72			19.3			3.64	0.874	
Streptococcus pneumoniae	31.7				2.4	6.83	1.04		3.33		2.14				2.89		13.1		
All pathogens	257	15.6	3.44	16.9	9.39	31.8	28.7	4.74	60	2.03	6.7	0.495	18.1	19.3	3.08	0.335	32.8	3.46	0.255

Counts

< 500

Ghana

Deaths (count) *associated with* bacterial antimicrobial resistance by pathogen−drug combinations, 2019

	Resistance to 1+	AG	Aminopenicillin	Anti−pseudomonal	BL−BLI	CP	3GC	4GC	FQ	Mono INH	Macrolide	MDR in S. Typhi and Paratyphi	MDR excluding XDR in TB	Methicillin	PCN	Mono RIF	TMP−SMX	Vanco	XDR in TB
Acinetobacter baumannii	1,520	700		1,510	575	632	592	469	690										
Citrobacter spp.	148	38.9		42		23.3	136	118	51.2										
Enterobacter spp.	831	412		293		210		679	577								637		
Enterococcus faecalis	449								440									31	
Enterococcus faecium	658								655									159	
Other enterococci	255								255									10.9	
Escherichia coli	3,560	2,190	3,410		3,060	162	2,470		2,580								3,210		
Group A Streptococcus	96.6										96.6								
Group B Streptococcus	674								260		549				10.5				
Haemophilus influenzae	125		91.1				55												
Klebsiella pneumoniae	4,580	3,600			4,230	61.6	3,780		3,250								4,320		
Morganella spp.	3.79						0.876	1.62	3.07										
Mycobacterium tuberculosis	786									180			598			0.223			8.21
Proteus spp.	310	119	236				162		215								243		
Pseudomonas aeruginosa	1,600	1,040		785		474	663	379	974										
Salmonella Paratyphi	2.03								1.81			0.242							
Salmonella Typhi	1,380								97.8			1,320							
Non−typhoidal Salmonella	0.019								0.019										
Serratia spp.	483	234		62.9		26.5	479	438	203										
Shigella spp.	3.05								3.05										
Staphylococcus aureus	3,670								1,340		1,670			1,690			2,290	95.8	
Streptococcus pneumoniae	4,160				311	755	376		666		369				1,010		4,080		
All pathogens	25,300	8,320	3,740	2,690	8,180	2,350	8,720	2,090	12,300	180	2,680	1,320	598	1,690	1,020	0.223	14,800	297	8.21

Counts
- < 500
- 500 − <1k
- 1k − <2.5k
- 2.5k − <7.5k
- 7.5k − <15k
- >=25k

Ghana

Deaths (count) *attributable to* bacterial antimicrobial resistance by pathogen−drug combinations, 2019

	Resistance to 1+	AG	Aminopenicillin	Anti−pseudomonal	BL−BLI	CP	3GC	4GC	FQ	Mono INH	Macrolide	MDR in S. Typhi and Paratyphi	MDR excluding XDR in TB	Methicillin	PCN	Mono RIF	TMP−SMX	Vanco	XDR in TB
Acinetobacter baumannii	420	34.7		187	0.009	111	0.034	0.029	86.9										
Citrobacter spp.	43.9	2.06		7.47		5.29	6.76	15.9	6.36										
Enterobacter spp.	207	21.4		18.5		38.7		42.3	57.6								28.1		
Enterococcus faecalis	119								110									9.07	
Enterococcus faecium	164								127									37.3	
Other enterococci	52.2								50									2.2	
Escherichia coli	934	125	15.2		69.3	31.4	297		233								164		
Group A Streptococcus	9.34										9.45								
Group B Streptococcus	101								44.6		53.7				2.91				
Haemophilus influenzae	29.3		13.3				16												
Klebsiella pneumoniae	1,190	253			59	25.4	470		192								191		
Morganella spp.	0.876						0.063	0.272	0.541										
Mycobacterium tuberculosis	391									27.3			358			0.063			5.03
Proteus spp.	58.9	4.98	2.43				28.9		14.5								8.09		
Pseudomonas aeruginosa	336	28.4		92.4		71	29.4	2.58	112										
Salmonella Paratyphi	0.411								0.378			0.033							
Salmonella Typhi	197								17.6			174							
Non−typhoidal Salmonella	0.004								0.004										
Serratia spp.	136	12.8		10.4		6.3	12.9	73.5	20.1										
Shigella spp.	0.634								0.634										
Staphylococcus aureus	792								56.8		61.6			397			251	25.2	
Streptococcus pneumoniae	748				5.94	167	14		78.9		14.8				56.2		412		
All pathogens	5,920	483	30.8	316	134	456	875	135	1,210	27.7	138	174	358	397	59.1	0.063	1,050	73.8	5.03

Counts
- < 500
- 500 − <1k
- 1k − <2.5k
- 2.5k − <7.5k

Guinea

Deaths (count) *associated with* bacterial antimicrobial resistance by pathogen−drug combinations, 2019

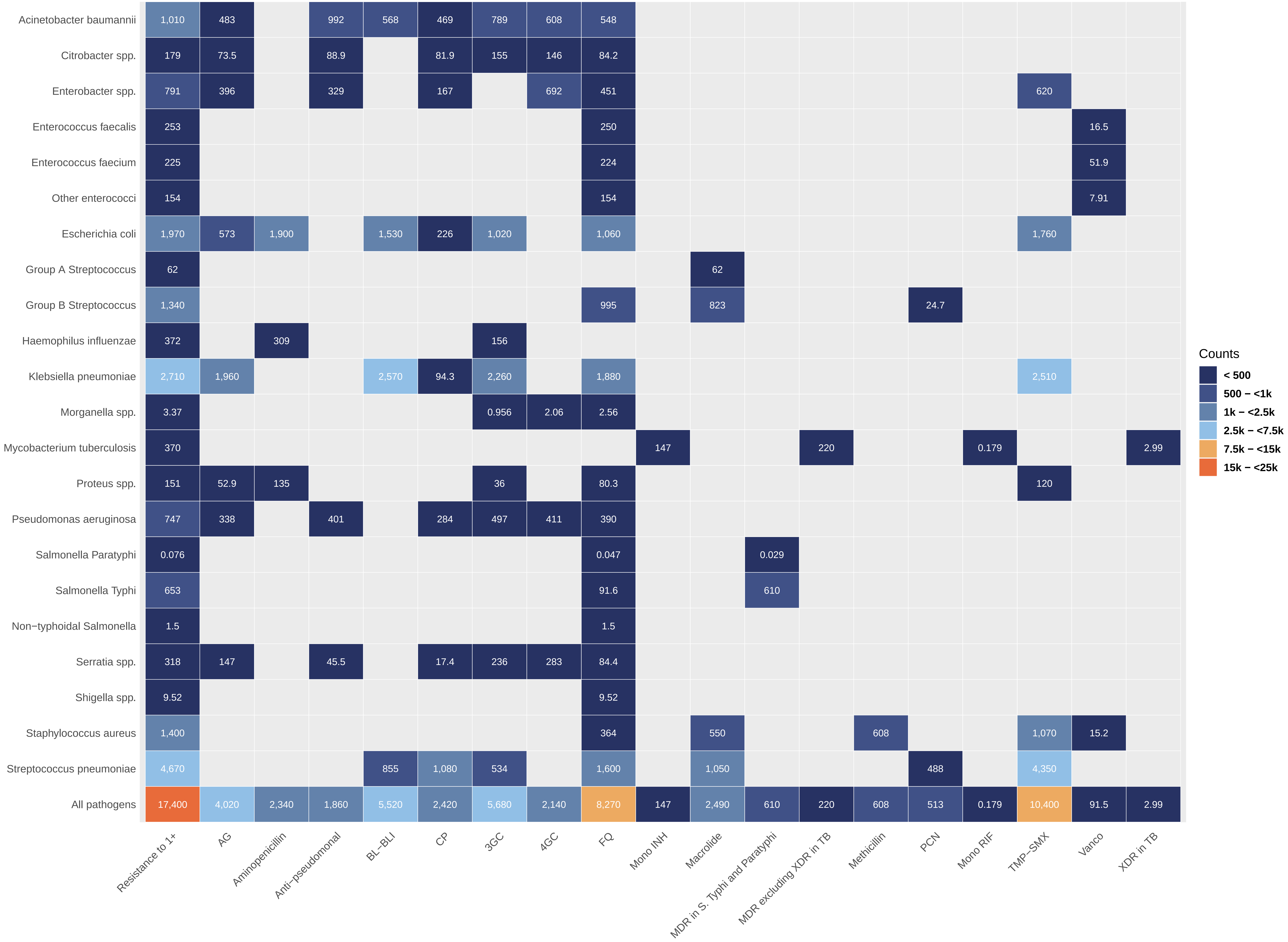

Guinea

Deaths (count) *attributable to* bacterial antimicrobial resistance by pathogen−drug combinations, 2019

	Resistance to 1+	AG	Aminopenicillin	Anti−pseudomonal	BL−BLI	CP	3GC	4GC	FQ	Mono INH	Macrolide	MDR in S. Typhi and Paratyphi	MDR excluding XDR in TB	Methicillin	PCN	Mono RIF	TMP−SMX	Vanco	XDR in TB
Acinetobacter baumannii	302	23.8		124	0.019	80.9	3.02	0.023	70.1										
Citrobacter spp.	55.1	3.6		10		17.8	4.37	8.91	10.3										
Enterobacter spp.	194	21.1		25.3		32.9		45.4	41.1								27.7		
Enterococcus faecalis	66.8								62.5									4.3	
Enterococcus faecium	55.9								43.7									12.3	
Other enterococci	31.7								30.1									1.58	
Escherichia coli	492	33.4	23.8		58.8	45.8	123		101								106		
Group A Streptococcus	5.91										6.01								
Group B Streptococcus	247								175		65.4				4.91				
Haemophilus influenzae	85.8		41.5				44.3												
Klebsiella pneumoniae	720	138			37.9	27.6	293		113								111		
Morganella spp.	0.801						0.044	0.34	0.418										
Mycobacterium tuberculosis	157									23.7			131			0.051			1.83
Proteus spp.	21.3	2.26	2.37				7.1		5.25								4.25		
Pseudomonas aeruginosa	187	9.45		47.9		48.2	24.8	14.1	42.3										
Salmonella Paratyphi	0.014								0.01			0.004							
Salmonella Typhi	92.9								15.3			75.8							
Non−typhoidal Salmonella	0.318								0.318										
Serratia spp.	81.9	8.02		9.5		5.05	5.95	45	8.24										
Shigella spp.	1.97								1.97										
Staphylococcus aureus	296								14.3		19.2			138			120	4.34	
Streptococcus pneumoniae	905				22.7	217	14.5		202		36.7				7.56		403		
All pathogens	4,000	239	67.7	217	119	476	520	114	937	23.1	130	77.5	131	138	12.5	0.051	772	22.5	1.83

Counts
- < 500
- 500 − <1k
- 2.5k − <7.5k

Guinea−Bissau

Deaths (count) *associated with* bacterial antimicrobial resistance by pathogen−drug combinations, 2019

	Resistance to 1+	AG	Aminopenicillin	Anti−pseudomonal	BL−BLI	CP	3GC	4GC	FQ	Mono INH	Macrolide	MDR in S. Typhi and Paratyphi	MDR excluding XDR in TB	Methicillin	PCN	Mono RIF	TMP−SMX	Vanco	XDR in TB
Acinetobacter baumannii	102	38		97.9	50.9	27.4	62.7	54.4	27.7										
Citrobacter spp.	10.2	2.3		3.91		1.4	8.72	8.11	2.86										
Enterobacter spp.	61.2	23.6		18.9		11.2		53.9	38.2								44.6		
Enterococcus faecalis	32.6								32.1									2.35	
Enterococcus faecium	34								33.8									9.14	
Other enterococci	11.5								11.5									0.82	
Escherichia coli	260	102	256		197	26.7	111		123								210		
Group A Streptococcus	7.26										7.26								
Group B Streptococcus	97								58		65.6				1.47				
Haemophilus influenzae	34.6		31.4				12												
Klebsiella pneumoniae	350	248			295	9.68	266		248								327		
Morganella spp.	0.359						0.087	0.233	0.254										
Mycobacterium tuberculosis	50									20.1			29.2			0.34			0.396
Proteus spp.	17.6	1.81	15.5				4.61		5.36								12.3		
Pseudomonas aeruginosa	80.6	33.4		50		27.5	50.6	44.4	28.1										
Salmonella Paratyphi	0.0005								0.0002			0.0004							
Salmonella Typhi	40.5								11.1			30.5							
Non−typhoidal Salmonella	0.004								0.004										
Serratia spp.	33.2	13.6		3.77		5.9	27	30.1	8.4										
Shigella spp.	2.81								2.81										
Staphylococcus aureus	272								41.6		187			69.7			184	10.4	
Streptococcus pneumoniae	325				115	58.4	39.4		110		109				30.5		296		
All pathogens	1,820	462	303	175	658	168	582	191	782	20.1	369	30.5	29.2	69.7	31.9	0.34	1,070	22.7	0.396

Counts
- < 500
- 500 − <1k
- 1k − <2.5k

Guinea−Bissau

Deaths (count) *attributable to* bacterial antimicrobial resistance by pathogen−drug combinations, 2019

	Resistance to 1+	AG	Aminopenicillin	Anti−pseudomonal	BL−BLI	CP	3GC	4GC	FQ	Mono INH	Macrolide	MDR in S. Typhi and Paratyphi	MDR excluding XDR in TB	Methicillin	PCN	Mono RIF	TMP−SMX	Vanco	XDR in TB
Acinetobacter baumannii	29	2.17		16.2	0.034	5.82	0.728	0.004	4										
Citrobacter spp.	2.98	0.131		0.758		0.34	0.405	0.995	0.359										
Enterobacter spp.	14.9	1.18		1.44		2.24		4.38	3.7								2.01		
Enterococcus faecalis	8.65								8.01									0.639	
Enterococcus faecium	8.63								6.48									2.15	
Other enterococci	2.4								2.23									0.161	
Escherichia coli	62.8	6.21	4.68		9.3	5.55	13.3		11.6								12.2		
Group A Streptococcus	0.711										0.712								
Group B Streptococcus	16.5								10.2		5.95				0.373				
Haemophilus influenzae	7.66		4.31				3.35												
Klebsiella pneumoniae	89.3	17.6			4.51	3.24	33.7		15								15.3		
Morganella spp.	0.084						0.003	0.04	0.041										
Mycobacterium tuberculosis	20.7									3.05			17.2			0.096			0.243
Proteus spp.	2.32	0.099	0.323				1.02		0.383								0.511		
Pseudomonas aeruginosa	20.1	1.01		7.3		5.15	2.19	1.49	2.99										
Salmonella Paratyphi	0.00008								0.00003			0.00005							
Salmonella Typhi	6.35								2.3			4.26							
Non−typhoidal Salmonella	0.0008								0.0008										
Serratia spp.	8.95	0.773		0.689		1.46	0.653	4.58	0.815										
Shigella spp.	0.591								0.591										
Staphylococcus aureus	48								1.72		7.21			16.3			20	2.81	
Streptococcus pneumoniae	62.7				4.68	11.9	2.22		13.8		3.84				0.577		25.9		
All pathogens	413	29.1	9.3	26.4	18.3	35.7	57.6	11.5	84.3	3.11	17.6	3.91	17.2	16.3	0.95	0.096	75.8	5.76	0.243

Counts

< 500

Kenya

Deaths (count) *associated with* bacterial antimicrobial resistance by pathogen−drug combinations, 2019

	Resistance to 1+	AG	Aminopenicillin	Anti−pseudomonal	BL−BLI	CP	3GC	4GC	FQ	Mono INH	Macrolide	MDR in S. Typhi and Paratyphi	MDR excluding XDR in TB	Methicillin	PCN	Mono RIF	TMP−SMX	Vanco	XDR in TB
Acinetobacter baumannii	2,010	773		1,920	1,410	1,060	1,850	1,120	1,340										
Citrobacter spp.	237	68		101		49.5	206	137	167										
Enterobacter spp.	1,320	566		583		194		1,140	786								1,010		
Enterococcus faecalis	939								930									46.8	
Enterococcus faecium	1,010								996									284	
Other enterococci	313								312									12.3	
Escherichia coli	5,890	1,840	5,730		3,090	230	3,040		2,800								5,180		
Group A Streptococcus	158										158								
Group B Streptococcus	1,450								710		1,170				12.6				
Haemophilus influenzae	412		391				82.3												
Klebsiella pneumoniae	6,190	4,180			5,090	205	4,520		4,370								5,860		
Morganella spp.	8.94						1.98	5.04	7										
Mycobacterium tuberculosis	1,390									631			584			164			7.95
Proteus spp.	565	211	486				103		331								496		
Pseudomonas aeruginosa	1,800	448		1,320		764	1,190	713	537										
Salmonella Paratyphi	3.94								0.673			3.25							
Salmonella Typhi	2,390								1,440			1,660							
Non−typhoidal Salmonella	3.87								3.87										
Serratia spp.	444	229		149		31.3	266	255	142										
Shigella spp.	213								213										
Staphylococcus aureus	4,970								1,370		2,700			1,440			3,360	67.6	
Streptococcus pneumoniae	5,550				436	1,950	523		433		1,330				2,890		5,320		
All pathogens	37,300	8,320	6,610	4,080	10,000	4,480	11,800	3,370	16,900	631	5,360	1,660	584	1,440	2,910	164	21,200	411	7.95

Counts
- < 500
- 500 − <1k
- 1k − <2.5k
- 2.5k − <7.5k
- 7.5k − <15k
- 15k − <25k
- >=25k

Kenya

Deaths (count) *attributable to* bacterial antimicrobial resistance by pathogen−drug combinations, 2019

	Resistance to 1+	AG	Aminopenicillin	Anti−pseudomonal	BL−BLI	CP	3GC	4GC	FQ	Mono INH	Macrolide	MDR in S. Typhi and Paratyphi	MDR excluding XDR in TB	Methicillin	PCN	Mono RIF	TMP−SMX	Vanco	XDR in TB
Acinetobacter baumannii	617	33.9		206	0.48	177	24.3	0.053	175										
Citrobacter spp.	70.7	3.02		14.4		8.92	11.6	9.41	23.3										
Enterobacter spp.	317	29.2		50.8		39.4		76.7	74.5								46.3		
Enterococcus faecalis	245								233									12.5	
Enterococcus faecium	258								190									67	
Other enterococci	64								61.4									2.6	
Escherichia coli	1,410	108	168		114	49.5	409		260								307		
Group A Streptococcus	14.3										15.1								
Group B Streptococcus	229								119		107				3.98				
Haemophilus influenzae	84.9		61.8				23.1												
Klebsiella pneumoniae	1,550	297			85.6	65.6	550		270								284		
Morganella spp.	2.08						0.086	0.82	1.18										
Mycobacterium tuberculosis	496									101			347			46.1			4.87
Proteus spp.	77	9.02	8.66				19.9		21.8								18.3		
Pseudomonas aeruginosa	469	12.7		187		150	56.4	5.05	57.2										
Salmonella Paratyphi	0.598								0.158			0.445							
Salmonella Typhi	430								250			180							
Non−typhoidal Salmonella	0.809								0.809										
Serratia spp.	104	13.2		28.8		8.59	12.4	26.5	14.5										
Shigella spp.	44.8								44.8										
Staphylococcus aureus	883								56.2		104			321			383	19.1	
Streptococcus pneumoniae	1,170				1.91	410	3.63		45		45.7				177		483		
All pathogens	8,540	507	238	487	202	909	1,110	119	1,900	101	272	183	347	321	181	46.1	1,520	101	4.87

Counts
- < 500
- 500 − <1k
- 1k − <2.5k
- 7.5k − <15k

Lesotho

Deaths (count) *associated with* bacterial antimicrobial resistance by pathogen−drug combinations, 2019

	Resistance to 1+	AG	Aminopenicillin	Anti−pseudomonal	BL−BLI	CP	3GC	4GC	FQ	Mono INH	Macrolide	MDR in S. Typhi and Paratyphi	MDR excluding XDR in TB	Methicillin	PCN	Mono RIF	TMP−SMX	Vanco	XDR in TB
Acinetobacter baumannii	223	65.4		209	128	120	197	179	142										
Citrobacter spp.	6.59	1.3		3.39		1.47	4.51	2.84	2.37										
Enterobacter spp.	55.6	10.9		15.1		10.2		24.3	20.5								47		
Enterococcus faecalis	43.9								43.1									3.13	
Enterococcus faecium	59.9								59.7									13	
Other enterococci	19.1								19									2.13	
Escherichia coli	342	60.3	323		230	17.9	130		74.1								274		
Group A Streptococcus	7.61										7.61								
Group B Streptococcus	85.3								34.9		70.6				1.92				
Haemophilus influenzae	26.2		25.1				3.96												
Klebsiella pneumoniae	473	177			382	6.67	439		266								387		
Morganella spp.	0.529						0.082	0.287	0.402										
Mycobacterium tuberculosis	403									99.4			240			60.5			3.27
Proteus spp.	32.7	1.96	29.8				8.01		4.05								23.1		
Pseudomonas aeruginosa	153	45.4		100		84.3	83.3	68.1	77.3										
Salmonella Paratyphi	0.016								0.016			0.0002							
Salmonella Typhi	65								28.6			43.1							
Non−typhoidal Salmonella	0.192								0.192										
Serratia spp.	19.8	12.7		4.29		4.51	7.03	7.79	4.14										
Shigella spp.	9.74								9.74										
Staphylococcus aureus	424								128		305			112			208	13.4	
Streptococcus pneumoniae	399				146	101	51.9		63.6		148				158		360		
All pathogens	2,850	375	378	332	885	346	925	283	977	99.4	531	43.1	240	112	160	60.5	1,300	31.6	3.27

Counts
- < 500
- 500 − <1k
- 1k − <2.5k
- 2.5k − <7.5k

Lesotho

Deaths (count) *attributable to* bacterial antimicrobial resistance by pathogen−drug combinations, 2019

	Resistance to 1+	AG	Aminopenicillin	Anti−pseudomonal	BL−BLI	CP	3GC	4GC	FQ	Mono INH	Macrolide	MDR in S. Typhi and Paratyphi	MDR excluding XDR in TB	Methicillin	PCN	Mono RIF	TMP−SMX	Vanco	XDR in TB
Acinetobacter baumannii	68.4	3		21.5	0.022	21.3	3.53	0.197	18.8										
Citrobacter spp.	1.92	0.077		0.665		0.368	0.244	0.215	0.353										
Enterobacter spp.	11.2	0.718		1.38		2.29		1.47	2.27								3.04		
Enterococcus faecalis	11.6								10.8									0.871	
Enterococcus faecium	14.7								11.7									3.05	
Other enterococci	4.04								3.64									0.392	
Escherichia coli	75.8	3.97	7.86		13.8	4.32	19.5		7.27								19.1		
Group A Streptococcus	0.689										0.758								
Group B Streptococcus	13								5.87		6.67				0.399				
Haemophilus influenzae	5.27		4.14				1.13												
Klebsiella pneumoniae	128	13			2.39	2.73	73.1		17.8								19.5		
Morganella spp.	0.12						0.003	0.048	0.069										
Mycobacterium tuberculosis	178									15.8			143			16.9			2
Proteus spp.	4.02	0.129	0.648				1.89		0.347								0.995		
Pseudomonas aeruginosa	38.3	1.19		10.4		15.3	2.19	0.85	8.3										
Salmonella Paratyphi	0.003								0.003			0.00003							
Salmonella Typhi	10.8								5.43			5.34							
Non−typhoidal Salmonella	0.04								0.04										
Serratia spp.	4.05	0.772		0.715		1.18	0.288	0.658	0.426										
Shigella spp.	2.04								2.04										
Staphylococcus aureus	71.3								5.29		12.4			27.3			22.5	3.87	
Streptococcus pneumoniae	80.9				5.67	20.8	1.9		7.43		5.29				8.21		31.7		
All pathogens	725	23	12.7	34.7	21.9	68.3	104	3.43	108	15.7	25	5.46	143	27.3	8.61	16.9	96.8	8.19	2

Counts
< 500
500 − <1k

Liberia

Deaths (count) *associated with* bacterial antimicrobial resistance by pathogen−drug combinations, 2019

	Resistance to 1+	AG	Aminopenicillin	Anti−pseudomonal	BL−BLI	CP	3GC	4GC	FQ	Mono INH	Macrolide	MDR in S. Typhi and Paratyphi	MDR excluding XDR in TB	Methicillin	PCN	Mono RIF	TMP−SMX	Vanco	XDR in TB
Acinetobacter baumannii	198	83.4		197	100	56.1	129	69.4	72.1										
Citrobacter spp.	22.6	5.93		9.2		3.25	20.5	18	7.81										
Enterobacter spp.	117	57.2		41.1		16.6		102	76.1								84.9		
Enterococcus faecalis	63.8								62.1									5.32	
Enterococcus faecium	86.6								86.1									20.2	
Other enterococci	31.4								31.3									1.9	
Escherichia coli	533	182	518		432	54.3	236		262								456		
Group A Streptococcus	14.9										14.9								
Group B Streptococcus	112								40.6		93.5				3.8				
Haemophilus influenzae	23.6		17.9				9.89												
Klebsiella pneumoniae	669	490			621	12.4	535		497								621		
Morganella spp.	0.744						0.209	0.451	0.537										
Mycobacterium tuberculosis	68.3									19.6			47.5			0.547			0.657
Proteus spp.	44.4	11.7	37.5				11.5		14.2								35.8		
Pseudomonas aeruginosa	215	127		111		76.4	134	86.3	99										
Salmonella Paratyphi	0.015								0.008			0.007							
Salmonella Typhi	184								20.9			170							
Non−typhoidal Salmonella	0.058								0.058										
Serratia spp.	69.1	29		9.76		3.26	59.5	60.1	15.8										
Shigella spp.	1.63								1.63										
Staphylococcus aureus	464								90.8		215			145			332	5.02	
Streptococcus pneumoniae	503				92.5	117	61.3		44.1		98.5				58.1		489		
All pathogens	3,420	986	574	368	1,250	339	1,200	336	1,420	19.6	422	170	47.5	145	61.9	0.547	2,020	32.5	0.657

Counts
- **< 500**
- **500 − <1k**
- **1k − <2.5k**
- **2.5k − <7.5k**

Liberia

Deaths (count) *attributable to* bacterial antimicrobial resistance by pathogen−drug combinations, 2019

	Resistance to 1+	AG	Aminopenicillin	Anti−pseudomonal	BL−BLI	CP	3GC	4GC	FQ	Mono INH	Macrolide	MDR in S. Typhi and Paratyphi	MDR excluding XDR in TB	Methicillin	PCN	Mono RIF	TMP−SMX	Vanco	XDR in TB
Acinetobacter baumannii	56.2	4.41		32.5	0.001	9.87	0.039	0.004	9.32										
Citrobacter spp.	6.69	0.316		1.7		0.745	0.876	2.08	0.973										
Enterobacter spp.	28.2	2.94		3.25		3.26		7.79	7.3								3.57		
Enterococcus faecalis	17.1								15.5									1.56	
Enterococcus faecium	21.5								16.8									4.79	
Other enterococci	6.48								6.1									0.381	
Escherichia coli	129	10.9	6.69		19.7	11.2	28.5		24.8								27.3		
Group A Streptococcus	1.3										1.47								
Group B Streptococcus	16.7								6.9		9.17				0.727				
Haemophilus influenzae	5.46		2.59				2.87												
Klebsiella pneumoniae	173	34			9.94	4.58	67.9		29.7								27.2		
Morganella spp.	0.176						0.011	0.077	0.088										
Mycobacterium tuberculosis	32.1									2.98			28.4			0.152			0.402
Proteus spp.	6.05	0.55	0.676				2.39		0.998								1.39		
Pseudomonas aeruginosa	50.5	3.7		13.4		13	7.86	1.97	10.7										
Salmonella Paratyphi	0.003								0.002			0.001							
Salmonella Typhi	26.4								3.83			21.7							
Non−typhoidal Salmonella	0.012								0.012										
Serratia spp.	18.5	1.67		2.11		0.956	2	10.2	1.59										
Shigella spp.	0.339								0.339										
Staphylococcus aureus	85.7								3.69		8.35			33.2			38.9	1.53	
Streptococcus pneumoniae	90.6				2.64	25.8	2.1		5.28		3.85				1.11		49.9		
All pathogens	772	58.4	9.96	53	32.3	69.4	114	22.1	144	3.35	22.6	22.7	28.4	33.2	1.84	0.152	148	8.26	0.402

Counts

< 500

500 − <1k

Madagascar

Deaths (count) *associated with* bacterial antimicrobial resistance by pathogen−drug combinations, 2019

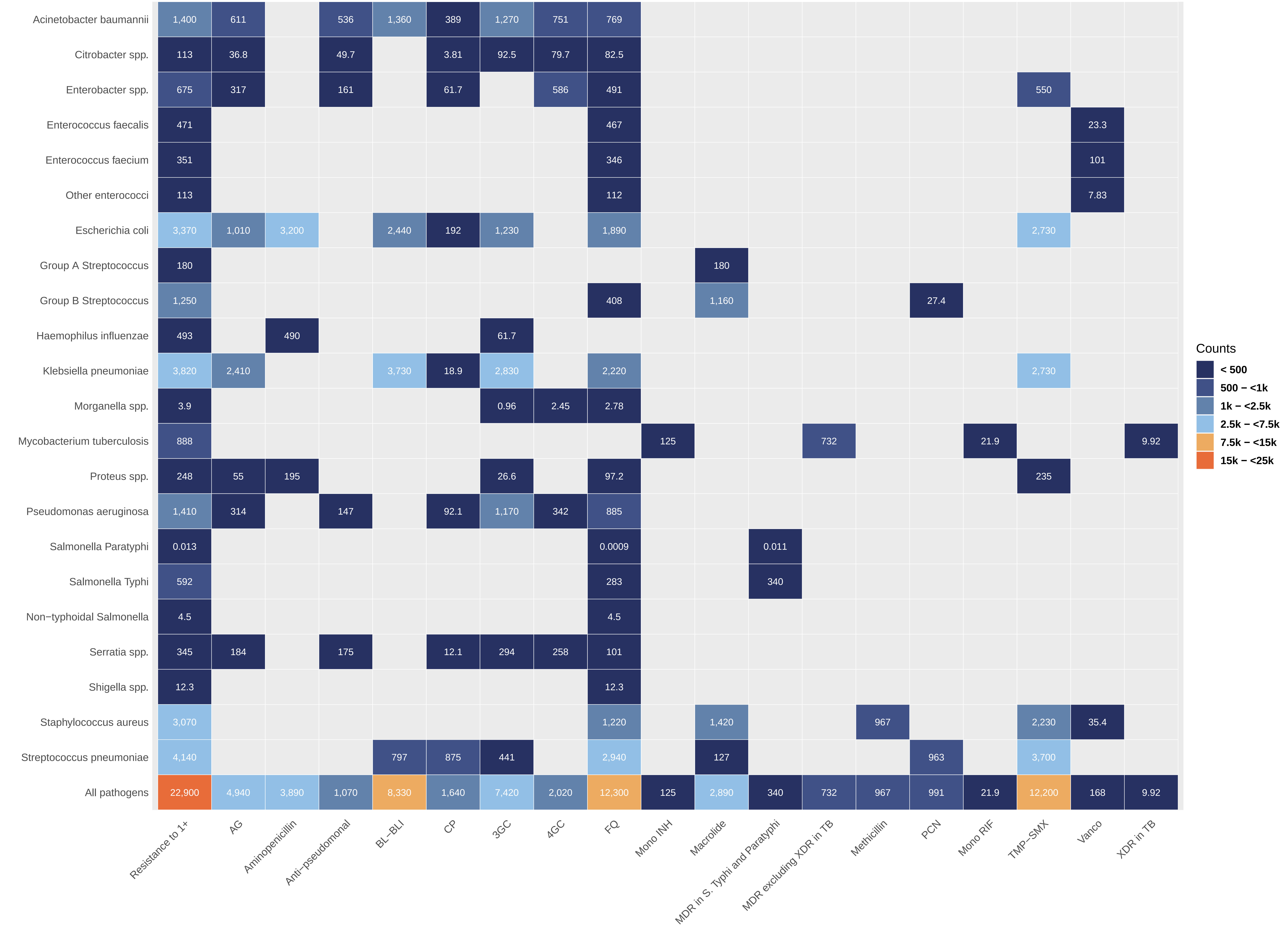

Madagascar

Deaths (count) *attributable to* bacterial antimicrobial resistance by pathogen−drug combinations, 2019

	Resistance to 1+	AG	Aminopenicillin	Anti−pseudomonal	BL−BLI	CP	3GC	4GC	FQ	Mono INH	Macrolide	MDR in S. Typhi and Paratyphi	MDR excluding XDR in TB	Methicillin	PCN	Mono RIF	TMP−SMX	Vanco	XDR in TB
Acinetobacter baumannii	405	28.8		31.1	21.5	61	133	33.6	96.4										
Citrobacter spp.	32.8	1.64		8.05		0.921	3.5	6.84	11.8										
Enterobacter spp.	160	15.3		12.1		12.2		50.3	47.2								23		
Enterococcus faecalis	123								117									6.07	
Enterococcus faecium	90.4								66.4									24	
Other enterococci	23.5								22									1.56	
Escherichia coli	787	59.3	59.3		129	40.8	147		186								165		
Group A Streptococcus	16.6										17.5								
Group B Streptococcus	179								63.4		110				5.49				
Haemophilus influenzae	96.7		79.6				17.1												
Klebsiella pneumoniae	958	177			117	12.1	394		140								118		
Morganella spp.	0.916						0.04	0.419	0.458										
Mycobacterium tuberculosis	463									19.2			431			6.14			6.08
Proteus spp.	29	2.62	3.97				5.76		7.02								9.92		
Pseudomonas aeruginosa	362	7.95		24.3		17.3	179	31.4	102										
Salmonella Paratyphi	0.002								0.0004			0.002							
Salmonella Typhi	99.9								56.3			42.9							
Non−typhoidal Salmonella	0.952								0.952										
Serratia spp.	91.2	10.3		34.6		3.79	9.08	23.1	9.96										
Shigella spp.	2.56								2.56										
Staphylococcus aureus	570								50.6		50.5			211			247	10.3	
Streptococcus pneumoniae	896				20.9	157	12.3		378		5.25				34.9		288		
All pathogens	5,390	303	143	110	289	305	902	146	1,360	19.6	183	44.4	431	211	40.3	6.14	850	41.9	6.08

Counts
- < 500
- 500 − <1k
- 1k − <2.5k
- 2.5k − <7.5k

Malawi

Deaths (count) *associated with* bacterial antimicrobial resistance by pathogen−drug combinations, 2019

	Resistance to 1+	AG	Aminopenicillin	Anti−pseudomonal	BL−BLI	CP	3GC	4GC	FQ	Mono INH	Macrolide	MDR in S. Typhi and Paratyphi	MDR excluding XDR in TB	Methicillin	PCN	Mono RIF	TMP−SMX	Vanco	XDR in TB
Acinetobacter baumannii	874	293		723	293	201	854	306	442										
Citrobacter spp.	89.5	23.7		37.6		4.74	89.2	54.3	18.2										
Enterobacter spp.	466	263		184		54.4		414	299								337		
Enterococcus faecalis	349								346									18.3	
Enterococcus faecium	305								302									83.1	
Other enterococci	103								103									5.64	
Escherichia coli	2,380	608	2,230		2,070	331	856		803								2,180		
Group A Streptococcus	127										127								
Group B Streptococcus	477								255		334				10.5				
Haemophilus influenzae	270		266				36.8												
Klebsiella pneumoniae	2,840	2,070			2,470	31.6	2,460		1,320								2,630		
Morganella spp.	3.3						0.804	2.01	2.4										
Mycobacterium tuberculosis	523									111			345			63.4			4.68
Proteus spp.	185	97.2	163				65		116								128		
Pseudomonas aeruginosa	786	277		293		222	620	268	372										
Salmonella Paratyphi	0.008								0.002			0.006							
Salmonella Typhi	893								202			865							
Non−typhoidal Salmonella	0.338								0.338										
Serratia spp.	184	80.3		70.3		11	37.2	147	37.5										
Shigella spp.	15.4								15.4										
Staphylococcus aureus	2,050								484		1,050			669			1,400	25.9	
Streptococcus pneumoniae	2,830				542	629	185		415		285				880		2,800		
All pathogens	15,700	3,710	2,660	1,310	5,370	1,480	5,210	1,190	5,540	111	1,800	865	345	669	891	63.4	9,480	133	4.68

Counts
- < 500
- 500 − <1k
- 1k − <2.5k
- 2.5k − <7.5k
- 7.5k − <15k
- 15k − <25k

Malawi

Deaths (count) *attributable to* bacterial antimicrobial resistance by pathogen−drug combinations, 2019

	Resistance to 1+	AG	Aminopenicillin	Anti−pseudomonal	BL−BLI	CP	3GC	4GC	FQ	Mono INH	Macrolide	MDR in S. Typhi and Paratyphi	MDR excluding XDR in TB	Methicillin	PCN	Mono RIF	TMP−SMX	Vanco	XDR in TB
Acinetobacter baumannii	262	14.5		108	0.013	34.3	47.3	0.032	58.7										
Citrobacter spp.	26.6	1.39		7.98		1.17	7.5	6.37	2.19										
Enterobacter spp.	112	14		15.3		10.7		30.1	27.7								14.3		
Enterococcus faecalis	91.4								86.6									4.8	
Enterococcus faecium	77.7								57.9									19.8	
Other enterococci	21.3								20.2									1.15	
Escherichia coli	557	38.4	16.1		102	73.7	101		76.8								148		
Group A Streptococcus	12.2										11.3								
Group B Streptococcus	78.7								45.2		30.9				2.42				
Haemophilus influenzae	53.5		43.4				10.1												
Klebsiella pneumoniae	751	154			24.5	14.1	351		80.3								127		
Morganella spp.	0.774						0.035	0.342	0.397										
Mycobacterium tuberculosis	245									18			206			17.7			2.87
Proteus spp.	30.1	4.34	2.41				11.8		7.55								3.99		
Pseudomonas aeruginosa	201	7.94		37.2		38.1	64.8	10.4	42.6										
Salmonella Paratyphi	0.001								0.0004			0.0009							
Salmonella Typhi	132								29.7			105							
Non−typhoidal Salmonella	0.071								0.071										
Serratia spp.	38.9	4.38		12.3		3.46	0.043	15.2	3.54										
Shigella spp.	3.23								3.23										
Staphylococcus aureus	380								19.7		40.7			152			161	7.37	
Streptococcus pneumoniae	530				18.7	135	2.45		45.6		10.4				46.5		272		
All pathogens	3,610	239	61.8	180	146	311	597	62.5	608	17.4	94.5	104	206	152	48.9	17.7	725	33.1	2.87

Counts
- < 500
- 500 − <1k
- 2.5k − <7.5k

Mali

Deaths (count) *associated with* bacterial antimicrobial resistance by pathogen−drug combinations, 2019

	Resistance to 1+	AG	Aminopenicillin	Anti−pseudomonal	BL−BLI	CP	3GC	4GC	FQ	Mono INH	Macrolide	MDR in S. Typhi and Paratyphi	MDR excluding XDR in TB	Methicillin	PCN	Mono RIF	TMP−SMX	Vanco	XDR in TB
Acinetobacter baumannii	1,220	541		1,210	672	467	782	765	602										
Citrobacter spp.	233	106		108		97.2	209	207	98.7										
Enterobacter spp.	1,010	465		399		213		862	647								823		
Enterococcus faecalis	574								566									38.8	
Enterococcus faecium	512								509									121	
Other enterococci	321								320									14.5	
Escherichia coli	4,380	1,480	4,180		3,310	820	2,140		2,780								4,190		
Group A Streptococcus	120										120								
Group B Streptococcus	1,890								1,350		1,150				38.9				
Haemophilus influenzae	362		235				207												
Klebsiella pneumoniae	6,510	4,690			6,070	219	5,840		4,880								6,200		
Morganella spp.	4.4						1.12	2.67	3.31										
Mycobacterium tuberculosis	491									145			336			4.63			4.59
Proteus spp.	282	99.1	246				71.3		154								224		
Pseudomonas aeruginosa	1,590	771		892		605	893	794	916										
Salmonella Paratyphi	0.283								0.144			0.143							
Salmonella Typhi	1,770								323			1,590							
Non−typhoidal Salmonella	1.86								1.86										
Serratia spp.	634	356		89.1		31	502	547	184										
Shigella spp.	31.8								31.8										
Staphylococcus aureus	3,490								630		1,630			1,750			2,310	127	
Streptococcus pneumoniae	4,250				1,060	1,480	660		492		1,560				513		3,420		
All pathogens	29,700	8,520	4,660	2,700	11,100	3,930	11,300	3,180	14,500	145	4,460	1,590	336	1,750	551	4.63	17,200	301	4.59

Counts
- < 500
- 500 − <1k
- 1k − <2.5k
- 2.5k − <7.5k
- 7.5k − <15k
- 15k − <25k
- >=25k

Mali

Deaths (count) *attributable to* bacterial antimicrobial resistance by pathogen−drug combinations, 2019

	Resistance to 1+	AG	Aminopenicillin	Anti−pseudomonal	BL−BLI	CP	3GC	4GC	FQ	Mono INH	Macrolide	MDR in S. Typhi and Paratyphi	MDR excluding XDR in TB	Methicillin	PCN	Mono RIF	TMP−SMX	Vanco	XDR in TB
Acinetobacter baumannii	355	26.6		168	0.019	81.5	0.373	0.027	78.4										
Citrobacter spp.	71.4	5.48		12.9		21.1	4.41	15.8	11.7										
Enterobacter spp.	247	23.8		28.7		40.7		55.2	61.4								36.9		
Enterococcus faecalis	152								141									10.3	
Enterococcus faecium	127								98.9									28.4	
Other enterococci	65.5								62.6									2.91	
Escherichia coli	1,130	84.8	49.6		109	160	217		264								247		
Group A Streptococcus	11.4										10.4								
Group B Streptococcus	343								239		98				7.68				
Haemophilus influenzae	89.8		30				59.8												
Klebsiella pneumoniae	1,760	326			50.8	63.6	753		295								274		
Morganella spp.	1.04						0.048	0.444	0.544										
Mycobacterium tuberculosis	227									23.2			200			1.3			2.81
Proteus spp.	40.4	4.21	4.28				13.9		10.2								7.83		
Pseudomonas aeruginosa	382	21.8		106		99.9	32.2	21.3	101										
Salmonella Paratyphi	0.049								0.03			0.019							
Salmonella Typhi	264								56.6			203							
Non−typhoidal Salmonella	0.398								0.398										
Serratia spp.	164	19.9		18.2		8.88	14.5	85.2	17.7										
Shigella spp.	6.62								6.62										
Staphylococcus aureus	789								26.2		60.3			419			251	32.2	
Streptococcus pneumoniae	842				26.1	335	14.3		66.6		65.6				4.52		331		
All pathogens	7,070	513	83.9	334	187	810	1,110	178	1,540	22.9	232	202	200	419	12.2	1.3	1,150	73.8	2.81

Counts
- **< 500**
- **500 − <1k**
- **1k − <2.5k**
- **2.5k − <7.5k**

Mauritania

Deaths (count) *associated with* bacterial antimicrobial resistance by pathogen−drug combinations, 2019

	Resistance to 1+	AG	Aminopenicillin	Anti−pseudomonal	BL−BLI	CP	3GC	4GC	FQ	Mono INH	Macrolide	MDR in S. Typhi and Paratyphi	MDR excluding XDR in TB	Methicillin	PCN	Mono RIF	TMP−SMX	Vanco	XDR in TB
Acinetobacter baumannii	153	83.7		153	86.1	72.2	95.1	83.4	87.4										
Citrobacter spp.	18.5	5.8		6.44		2.97	16.3	15.2	7.11										
Enterobacter spp.	110	40.8		42.2		20.4		83.4	67.3								89.6		
Enterococcus faecalis	51								49.9									3.81	
Enterococcus faecium	68.6								68.3									16.5	
Other enterococci	32.5								32.4									2.01	
Escherichia coli	416	191	403		318	39.8	177		164								371		
Group A Streptococcus	9.81										9.81								
Group B Streptococcus	113								53.2		86.4				3.26				
Haemophilus influenzae	15.7		10.9				7.51												
Klebsiella pneumoniae	476	271			440	20.4	381		328								443		
Morganella spp.	0.623						0.152	0.173	0.554										
Mycobacterium tuberculosis	37.9									11.6			25.5			0.324			0.35
Proteus spp.	35.2	20	28.8				9		18.7								28.2		
Pseudomonas aeruginosa	180	96.8		88		74.6	96.1	62.3	114										
Salmonella Paratyphi	0.03								0.025			0.005							
Salmonella Typhi	70.7								17.2			58.2							
Non−typhoidal Salmonella	0.052								0.052										
Serratia spp.	45.3	22.2		7.02		3.41	35.2	35.8	13.6										
Shigella spp.	7.11								7.11										
Staphylococcus aureus	376								77		217			219			201	14.8	
Streptococcus pneumoniae	384				66.9	114	50		27.2		114				52.2		369		
All pathogens	2,600	732	443	296	911	348	867	280	1,130	11.6	427	58.3	25.5	219	55.5	0.324	1,500	37.1	0.35

Counts
- < 500
- 500 − <1k
- 1k − <2.5k
- 2.5k − <7.5k

Mauritania

Deaths (count) *attributable to* bacterial antimicrobial resistance by pathogen−drug combinations, 2019

	Resistance to 1+	AG	Aminopenicillin	Anti−pseudomonal	BL−BLI	CP	3GC	4GC	FQ	Mono INH	Macrolide	MDR in S. Typhi and Paratyphi	MDR excluding XDR in TB	Methicillin	PCN	Mono RIF	TMP−SMX	Vanco	XDR in TB
Acinetobacter baumannii	44.8	3.96		17.8	0.001	12	0.008	0.003	11										
Citrobacter spp.	5.44	0.309		1.11		0.664	0.635	1.83	0.891										
Enterobacter spp.	25.9	2.15		3.3		3.99		5.29	6.77								4.33		
Enterococcus faecalis	13.6								12.5									1.08	
Enterococcus faecium	17.1								13.2									3.89	
Other enterococci	6.67								6.29									0.38	
Escherichia coli	98.5	11.8	6.34		13.9	8.23	21.2		14.8								22.3		
Group A Streptococcus	0.934										1.03								
Group B Streptococcus	17.9								9.21		8.01				0.633				
Haemophilus influenzae	3.74		1.55				2.19												
Klebsiella pneumoniae	124	19.2			7.44	5.7	50.2		20.5								20.8		
Morganella spp.	0.143						0.015	0.028	0.1										
Mycobacterium tuberculosis	17.5									1.96			15.3			0.092			0.214
Proteus spp.	5.18	0.931	0.479				1.66		1.17								0.932		
Pseudomonas aeruginosa	41.8	2.59		8.65		11.9	4.83	1.06	12.8										
Salmonella Paratyphi	0.006								0.005			0.0007							
Salmonella Typhi	10.7								3.25			7.74							
Non−typhoidal Salmonella	0.011								0.011										
Serratia spp.	11.7	1.24		1.41		0.923	1.6	5.23	1.33										
Shigella spp.	1.49								1.49										
Staphylococcus aureus	89.8								3.27		8.15			54			20.7	3.71	
Streptococcus pneumoniae	72.2				1.48	24.6	0.966		3.24		4.27				0.859		36.9		
All pathogens	609	42.2	8.35	32.3	22.8	67.9	83.3	13.4	122	1.9	21.4	7.46	15.3	54	1.49	0.092	106	9.05	0.214

Counts
- < 500
- 500 − <1k

Mauritius

Deaths (count) *associated with* bacterial antimicrobial resistance by pathogen−drug combinations, 2019

	Resistance to 1+	AG	Aminopenicillin	Anti−pseudomonal	BL−BLI	CP	3GC	4GC	FQ	Mono INH	Macrolide	MDR in S. Typhi and Paratyphi	MDR excluding XDR in TB	Methicillin	PCN	Mono RIF	TMP−SMX	Vanco	XDR in TB
Acinetobacter baumannii	77.4	59.8		74.5	60.2	60.7	70.1	73.7	65.1										
Citrobacter spp.	4.5	0.469		0.579		0.805	2.78	2.68	1.91										
Enterobacter spp.	24.7	3.78		12.2		6.62		10.2	6.32								15.4		
Enterococcus faecalis	18.8								17.8									2.39	
Enterococcus faecium	42.7								42.4									9.98	
Other enterococci	19.7								19.6									6.9	
Escherichia coli	141	18.2	134		106	19	67.4		82.8								95.8		
Group A Streptococcus	7.12										7.12								
Group B Streptococcus	13.9								3.79		11.7				1.25				
Haemophilus influenzae	3.23		2.94				0.715												
Klebsiella pneumoniae	100	12.7			71.8	18.8	77.5		50.4								75.5		
Morganella spp.	0.391						0.233	0.086	0.233										
Mycobacterium tuberculosis	0.66									0.454			0.159			0.016			0.03
Proteus spp.	18.1	13.2	14.4				6.88		7.17								10.2		
Pseudomonas aeruginosa	57.6	16.9		21.8		40.7	29.6	22	25.5										
Salmonella Paratyphi	0.389								0.382			0.008							
Salmonella Typhi	0.827								0.76			0.072							
Non−typhoidal Salmonella	0.011								0.011										
Serratia spp.	4.7	0.691		1.55		1.08	3.36	1.4	1.43										
Shigella spp.	0.05								0.05										
Staphylococcus aureus	166								45.9		87.4			152			43.9	2.67	
Streptococcus pneumoniae	69.9				11.8	36.2	13.8		3.09		42.3				24.4		50.9		
All pathogens	772	126	152	111	250	184	272	110	375	0.454	149	0.081	0.159	152	25.6	0.016	292	21.9	0.03

Counts
- **< 500**
- **500 − <1k**

Mauritius

Deaths (count) *attributable to* bacterial antimicrobial resistance by pathogen−drug combinations, 2019

	Resistance to 1+	AG	Aminopenicillin	Anti−pseudomonal	BL−BLI	CP	3GC	4GC	FQ	Mono INH	Macrolide	MDR in S. Typhi and Paratyphi	MDR excluding XDR in TB	Methicillin	PCN	Mono RIF	TMP−SMX	Vanco	XDR in TB
Acinetobacter baumannii	24.5	2.58		3.08	0.013	9.95	0.417	0.316	8.09										
Citrobacter spp.	1.26	0.024		0.113		0.193	0.277	0.357	0.298										
Enterobacter spp.	5.45	0.251		1.39		1.71		0.419	0.674								1.04		
Enterococcus faecalis	5.13								4.4									0.723	
Enterococcus faecium	10.6								8.27									2.35	
Other enterococci	4.52								3.37									1.15	
Escherichia coli	35.5	1.1	2.01		5.14	4.23	8.58		8.81								5.67		
Group A Streptococcus	0.667										0.637								
Group B Streptococcus	2.05								0.657		1.15				0.23				
Haemophilus influenzae	0.678		0.472				0.206												
Klebsiella pneumoniae	27.3	1.29			1.37	4.88	11.3		3.91								4.49		
Morganella spp.	0.1						0.042	0.019	0.039										
Mycobacterium tuberculosis	0.189									0.07			0.095			0.005			0.019
Proteus spp.	2.92	0.713	0.193				1.26		0.424								0.321		
Pseudomonas aeruginosa	14.2	0.454		1.16		7.73	1.64	0.51	2.68										
Salmonella Paratyphi	0.08								0.079			0.001							
Salmonella Typhi	0.168								0.158			0.01							
Non−typhoidal Salmonella	0.002								0.002										
Serratia spp.	1.25	0.04		0.326		0.297	0.302	0.119	0.165										
Shigella spp.	0.01								0.01										
Staphylococcus aureus	50.8								2.06		3.01			41.4			3.68	0.59	
Streptococcus pneumoniae	15.5				0.036	8.26	0.072		0.342		1.71				0.681		4.42		
All pathogens	203	6.5	2.68	6.07	6.56	37.3	24.1	1.74	44.4	0.078	6.57	0.01	0.095	41.4	0.911	0.005	19.6	4.81	0.019

Counts
< 500

Mozambique

Deaths (count) *associated with* bacterial antimicrobial resistance by pathogen−drug combinations, 2019

	Resistance to 1+	AG	Aminopenicillin	Anti−pseudomonal	BL−BLI	CP	3GC	4GC	FQ	Mono INH	Macrolide	MDR in S. Typhi and Paratyphi	MDR excluding XDR in TB	Methicillin	PCN	Mono RIF	TMP−SMX	Vanco	XDR in TB
Acinetobacter baumannii	1,650	488		1,420	1,060	271	1,340	652	589										
Citrobacter spp.	153	49.9		72.1		9.95	134	109	87.5										
Enterobacter spp.	805	389		293		96.7		732	492								610		
Enterococcus faecalis	591								584									44.5	
Enterococcus faecium	482								476									123	
Other enterococci	183								182									11.4	
Escherichia coli	3,540	1,000	3,270		2,410	255	1,200		1,260								2,790		
Group A Streptococcus	273										273								
Group B Streptococcus	1,150								485		968				32.4				
Haemophilus influenzae	536		528				90												
Klebsiella pneumoniae	5,600	4,090			5,060	18.1	5,170		3,830								5,450		
Morganella spp.	6.71						1.56	4.21	4.88										
Mycobacterium tuberculosis	2,720									218			2,250			224			30.7
Proteus spp.	317	88.4	264				62.9		125								282		
Pseudomonas aeruginosa	1,340	368		526		332	1,150	530	480										
Salmonella Paratyphi	0.039								0.002			0.034							
Salmonella Typhi	2,220								482			2,000							
Non−typhoidal Salmonella	14.5								14.5										
Serratia spp.	447	222		176		23.2	267	365	111										
Shigella spp.	35.2								35.2										
Staphylococcus aureus	4,130								1,130		688			1,780			3,530	50.1	
Streptococcus pneumoniae	4,880				913	1,050	598		2,320		496				940		4,530		
All pathogens	31,100	6,690	4,060	2,480	9,440	2,060	10,000	2,390	12,700	218	2,420	2,000	2,250	1,780	972	224	17,200	229	30.7

Counts
- < 500
- 500 − <1k
- 1k − <2.5k
- 2.5k − <7.5k
- 7.5k − <15k
- 15k − <25k
- >=25k

Mozambique

Deaths (count) *attributable to* bacterial antimicrobial resistance by pathogen−drug combinations, 2019

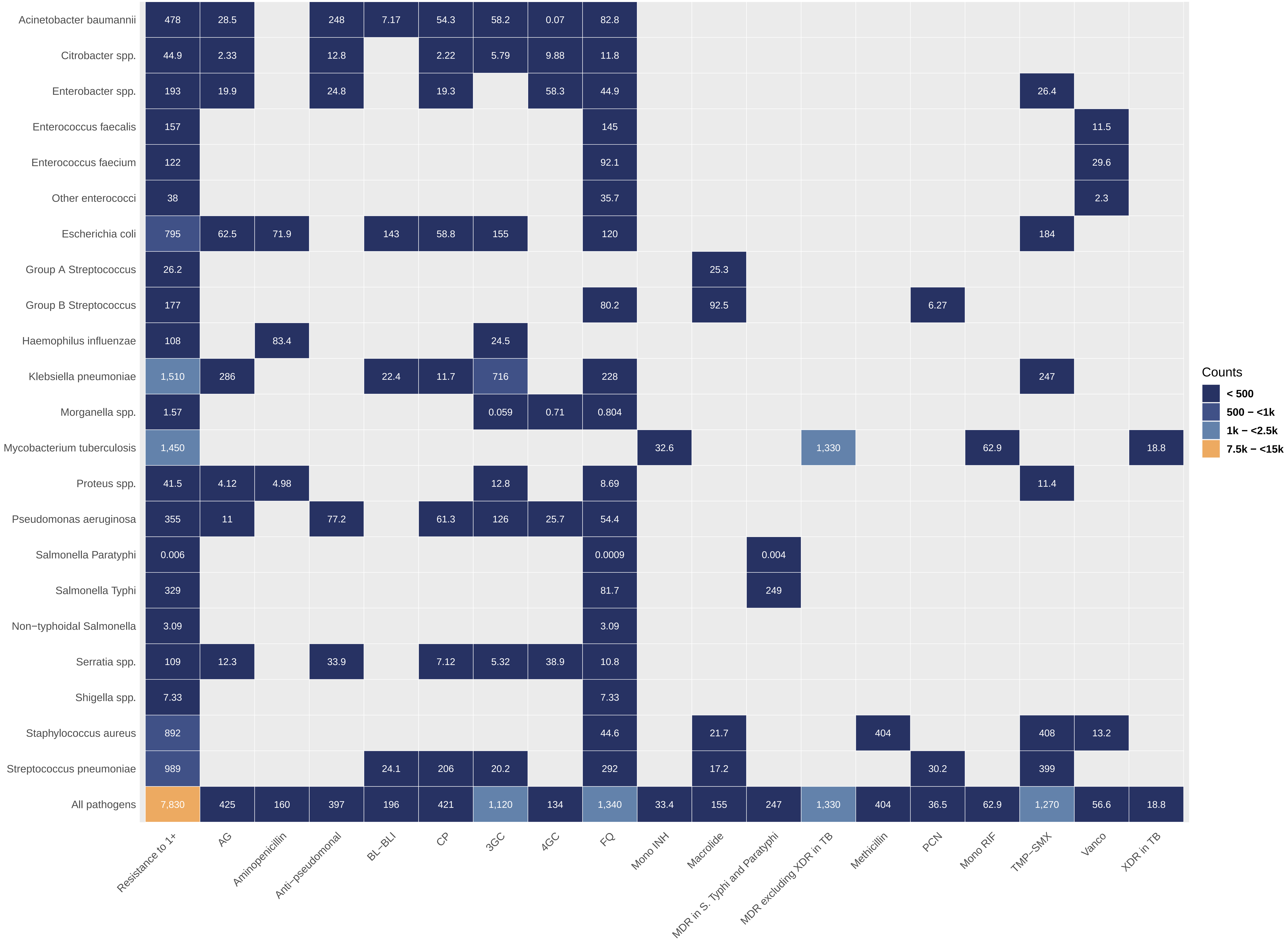

Namibia

Deaths (count) *associated with* bacterial antimicrobial resistance by pathogen−drug combinations, 2019

	Resistance to 1+	AG	Aminopenicillin	Anti−pseudomonal	BL−BLI	CP	3GC	4GC	FQ	Mono INH	Macrolide	MDR in S. Typhi and Paratyphi	MDR excluding XDR in TB	Methicillin	PCN	Mono RIF	TMP−SMX	Vanco	XDR in TB
Acinetobacter baumannii	132	63.9		128	89.2	90	121	95.5	114										
Citrobacter spp.	6.43	0.397		2.19		0.947	4.2	3.3	2.5										
Enterobacter spp.	52.6	5.65		13.7		10.7		14.8	11.9								47.4		
Enterococcus faecalis	22.2								21.2									2.17	
Enterococcus faecium	54.7								54.5									9.99	
Other enterococci	20.1								20									1.15	
Escherichia coli	266	24.2	253		154	12.5	106		73.7								229		
Group A Streptococcus	2.86										2.86								
Group B Streptococcus	47.2								5.05		45.6				2.14				
Haemophilus influenzae	12.4		11				3.02												
Klebsiella pneumoniae	319	82.8			289	2.99	222		139								283		
Morganella spp.	0.412						0.055	0.08	0.38										
Mycobacterium tuberculosis	177									61.4			101			13.2			1.38
Proteus spp.	25.3	1.36	22.3				5.16		5.72								19.7		
Pseudomonas aeruginosa	128	33.4		73.8		84.2	59.2	45.9	72.4										
Salmonella Paratyphi	0.016								0.016			0.0002							
Salmonella Typhi	16.4								6.37			11.2							
Non−typhoidal Salmonella	0.122								0.122										
Serratia spp.	14.6	8.99		2.82		1.86	5.56	7.34	2.73										
Shigella spp.	5.74								5.74										
Staphylococcus aureus	306								124		147			107			191	4.14	
Streptococcus pneumoniae	269				65.8	82.5	44.1		19.2		87.6				125		233		
All pathogens	1,880	221	286	221	598	286	570	167	679	61.4	283	11.2	101	107	128	13.2	1,000	17.4	1.38

Counts
- < 500
- 500 − <1k
- 1k − <2.5k

Namibia

Deaths (count) *attributable to* bacterial antimicrobial resistance by pathogen−drug combinations, 2019

	Resistance to 1+	AG	Aminopenicillin	Anti−pseudomonal	BL−BLI	CP	3GC	4GC	FQ	Mono INH	Macrolide	MDR in S. Typhi and Paratyphi	MDR excluding XDR in TB	Methicillin	PCN	Mono RIF	TMP−SMX	Vanco	XDR in TB
Acinetobacter baumannii	41.4	2.72		8.03	0.004	14.6	1.02	0.005	14.9										
Citrobacter spp.	1.81	0.022		0.437		0.234	0.348	0.367	0.406										
Enterobacter spp.	9.69	0.439		1.23		2.56		0.605	1.42								3.45		
Enterococcus faecalis	6.01								5.3									0.707	
Enterococcus faecium	13.2								10.8									2.38	
Other enterococci	4.14								3.91									0.227	
Escherichia coli	59.5	1.64	7.74		8.27	2.94	15.6		7.32								16		
Group A Streptococcus	0.294										0.298								
Group B Streptococcus	5.86								0.791		4.57				0.396				
Haemophilus influenzae	2.63		1.76				0.874												
Klebsiella pneumoniae	77.3	6.61			8.58	1.46	35.4		9.65								15.6		
Morganella spp.	0.091						0.006	0.013	0.072										
Mycobacterium tuberculosis	74.2									9.65			59.9			3.7			0.847
Proteus spp.	3.05	0.088	0.467				1.19		0.448								0.855		
Pseudomonas aeruginosa	31.3	0.847		5.66		15	1.46	0.439	7.85										
Salmonella Paratyphi	0.003								0.003			0.00002							
Salmonella Typhi	2.67								1.25			1.47							
Non−typhoidal Salmonella	0.026								0.026										
Serratia spp.	2.96	0.543		0.529		0.497	0.275	0.831	0.277										
Shigella spp.	1.21								1.21										
Staphylococcus aureus	57.9								5.42		5.55			24.5			21.3	1.21	
Streptococcus pneumoniae	55.7				1.77	17.8	1.27		2.2		3.24				7.88		21.6		
All pathogens	451	12.9	9.98	15.9	18.6	55.1	57.5	2.26	73.3	10	13.7	1.45	59.9	24.5	8.28	3.7	78.7	4.52	0.847

Counts

< 500

Niger

Deaths (count) *associated with* bacterial antimicrobial resistance by pathogen−drug combinations, 2019

	Resistance to 1+	AG	Aminopenicillin	Anti−pseudomonal	BL−BLI	CP	3GC	4GC	FQ	Mono INH	Macrolide	MDR in S. Typhi and Paratyphi	MDR excluding XDR in TB	Methicillin	PCN	Mono RIF	TMP−SMX	Vanco	XDR in TB
Acinetobacter baumannii	971	397		961	540	334	598	431	325										
Citrobacter spp.	182	79.6		86.4		69.6	165	163	67.6										
Enterobacter spp.	968	411		338		203		853	655								746		
Enterococcus faecalis	370								362									29.5	
Enterococcus faecium	391								389									109	
Other enterococci	186								185									10.2	
Escherichia coli	4,490	1,370	4,380		3,450	497	1,890		1,540								3,970		
Group A Streptococcus	94.9										94.9								
Group B Streptococcus	2,210								1,420		1,470				73.4				
Haemophilus influenzae	590		464				267												
Klebsiella pneumoniae	6,110	4,470			5,730	163	4,800		4,350								5,670		
Morganella spp.	3.43						0.83	2.19	2.48										
Mycobacterium tuberculosis	614									162			317			131			4.3
Proteus spp.	216	77.2	183				56.6		133								173		
Pseudomonas aeruginosa	1,390	671		773		416	847	756	702										
Salmonella Paratyphi	1.15								1.12			0.034							
Salmonella Typhi	898								260			685							
Non−typhoidal Salmonella	0.825								0.825										
Serratia spp.	475	269		72.3		22	407	400	126										
Shigella spp.	19.7								19.7										
Staphylococcus aureus	3,310								375		1,480			1,650			2,210	121	
Streptococcus pneumoniae	7,780				1,370	1,660	770		2,400		1,880				175		7,240		
All pathogens	31,300	7,740	5,020	2,230	11,100	3,370	9,810	2,600	13,300	162	4,930	685	317	1,650	249	131	20,000	269	4.3

Counts
- < 500
- 500 − <1k
- 1k − <2.5k
- 2.5k − <7.5k
- 7.5k − <15k
- 15k − <25k
- >=25k

Niger

Deaths (count) *attributable to* bacterial antimicrobial resistance by pathogen−drug combinations, 2019

	Resistance to 1+	AG	Aminopenicillin	Anti−pseudomonal	BL−BLI	CP	3GC	4GC	FQ	Mono INH	Macrolide	MDR in S. Typhi and Paratyphi	MDR excluding XDR in TB	Methicillin	PCN	Mono RIF	TMP−SMX	Vanco	XDR in TB
Acinetobacter baumannii	277	20.9		148	0.018	63.7	0.392	0.021	42.9										
Citrobacter spp.	55.6	4.35		11.2		15.4	3.29	13.4	7.96										
Enterobacter spp.	240	20.5		23.8		39		60.9	63.3								31.8		
Enterococcus faecalis	98.6								90.3									8.26	
Enterococcus faecium	99.7								74.3									25.4	
Other enterococci	38.3								36.2									2.08	
Escherichia coli	1,060	84.6	74.5		161	108	237		143								253		
Group A Streptococcus	8.77										9.08								
Group B Streptococcus	388								247		129				13.2				
Haemophilus influenzae	138		61.9				76.3												
Klebsiella pneumoniae	1,580	312			102	52.8	603		260								249		
Morganella spp.	0.807						0.032	0.369	0.406										
Mycobacterium tuberculosis	253									26.1			188			36.8			2.64
Proteus spp.	31.9	3.24	3.05				10.7		8.92								6.17		
Pseudomonas aeruginosa	337	19.2		106		69.2	37.8	28.1	76.8										
Salmonella Paratyphi	0.233								0.229			0.004							
Salmonella Typhi	139								51			86.3							
Non−typhoidal Salmonella	0.174								0.174										
Serratia spp.	126	15.5		14.7		6.29	14.4	63	12.3										
Shigella spp.	4.08								4.08										
Staphylococcus aureus	749								17.2		55.2			401			244	31.4	
Streptococcus pneumoniae	1,460				38.7	339	21.6		309		69				1.13		684		
All pathogens	7,090	480	139	304	302	694	1,000	166	1,450	26.7	259	86.5	188	401	14.3	36.8	1,470	67.1	2.64

Counts
- < 500
- 500 − <1k
- 1k − <2.5k
- 2.5k − <7.5k

Nigeria

Deaths (count) *associated with* bacterial antimicrobial resistance by pathogen−drug combinations, 2019

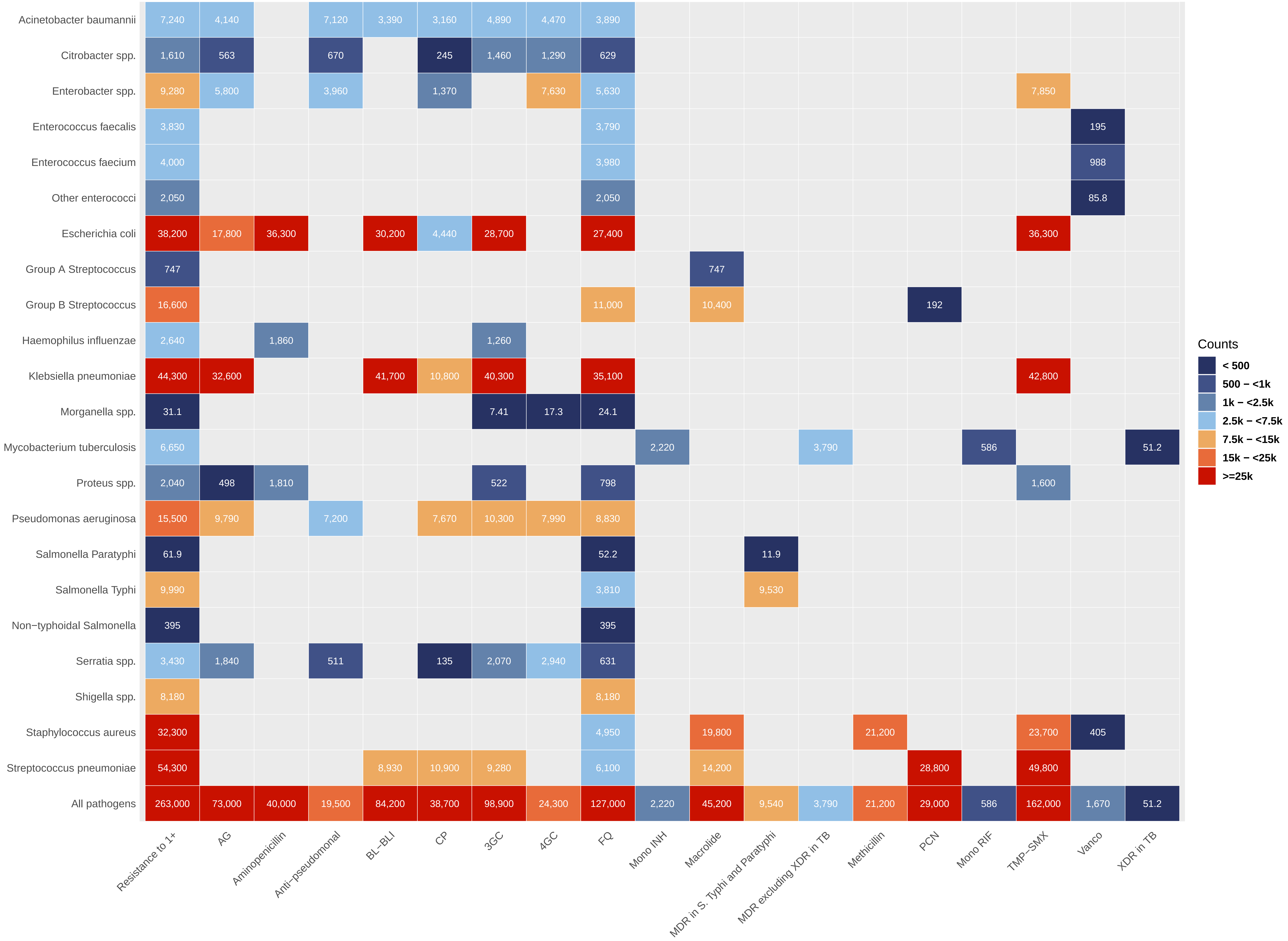

Nigeria

Deaths (count) *attributable to* bacterial antimicrobial resistance by pathogen−drug combinations, 2019

	Resistance to 1+	AG	Aminopenicillin	Anti−pseudomonal	BL−BLI	CP	3GC	4GC	FQ	Mono INH	Macrolide	MDR in S. Typhi and Paratyphi	MDR excluding XDR in TB	Methicillin	PCN	Mono RIF	TMP−SMX	Vanco	XDR in TB
Acinetobacter baumannii	2,130	206		868	0.054	542	14.4	0.171	498										
Citrobacter spp.	479	30.1		117		54	58.1	141	78.3										
Enterobacter spp.	2,200	320		307		261		456	506								347		
Enterococcus faecalis	1,000								951									52.8	
Enterococcus faecium	1,000								771									231	
Other enterococci	419								402									17.2	
Escherichia coli	10,400	985	198		546	794	3,390		2,500								1,950		
Group A Streptococcus	67.9										72.1								
Group B Streptococcus	2,910								1,950		910				53.3				
Haemophilus influenzae	629		263				366												
Klebsiella pneumoniae	13,000	2,320			282	2,180	4,150		2,190								1,890		
Morganella spp.	7.25						0.357	2.86	4.04										
Mycobacterium tuberculosis	2,790									328			2,240			165			31.4
Proteus spp.	280	23	33.1				108		54.3								61.5		
Pseudomonas aeruginosa	3,780	268		557		1,260	531	235	928										
Salmonella Paratyphi	12.1								10.7			1.51							
Salmonella Typhi	1,630								550			1,070							
Non−typhoidal Salmonella	82.2								82.2										
Serratia spp.	823	105		108		44.3	51.7	453	61.7										
Shigella spp.	1,700								1,700										
Staphylococcus aureus	8,140								208		647			4,930			2,260	102	
Streptococcus pneumoniae	11,000				224	2,390	571		687		476				2,100		4,530		
All pathogens	64,500	4,260	495	1,960	1,050	7,530	9,240	1,290	14,100	338	2,120	1,050	2,240	4,930	2,150	165	11,000	403	31.4

Counts
- < 500
- 500 − <1k
- 1k − <2.5k
- 2.5k − <7.5k
- 7.5k − <15k
- >=25k

Rwanda

Deaths (count) *associated with* bacterial antimicrobial resistance by pathogen−drug combinations, 2019

	Resistance to 1+	AG	Aminopenicillin	Anti−pseudomonal	BL−BLI	CP	3GC	4GC	FQ	Mono INH	Macrolide	MDR in S. Typhi and Paratyphi	MDR excluding XDR in TB	Methicillin	PCN	Mono RIF	TMP−SMX	Vanco	XDR in TB
Acinetobacter baumannii	563	184		540	345	347	516	366	359										
Citrobacter spp.	49.2	10.1		18.6		2.77	41.8	22.9	31.4										
Enterobacter spp.	308	174		138		22		291	176								215		
Enterococcus faecalis	201								199									10.2	
Enterococcus faecium	226								224									62.6	
Other enterococci	78.2								77.9									4.39	
Escherichia coli	1,400	371	1,330		1,060	71.4	806		655								1,190		
Group A Streptococcus	35.9										35.9								
Group B Streptococcus	313								141		256				2.52				
Haemophilus influenzae	80.1		75.4				14.2												
Klebsiella pneumoniae	1,770	1,310			1,750	80.9	1,680		1,120								1,580		
Morganella spp.	2.3						0.485	1.26	1.8										
Mycobacterium tuberculosis	328									38.3			266			19.4			3.61
Proteus spp.	130	33.4	114				21.9		59.9								110		
Pseudomonas aeruginosa	666	396		169		260	528	161	249										
Salmonella Paratyphi	0.028								0.008			0.02							
Salmonella Typhi	456								196			364							
Non−typhoidal Salmonella	1.04								1.04										
Serratia spp.	100	51.4		27.5		5.47	67.4	45.1	31.9										
Shigella spp.	14.9								14.9										
Staphylococcus aureus	1,610								550		848			744			1,150	17.2	
Streptococcus pneumoniae	1,420				136	376	194		207		214				389		1,380		
All pathogens	9,760	2,530	1,520	893	3,280	1,160	3,870	887	4,300	38.3	1,350	364	266	744	391	19.4	5,620	94.4	3.61

Counts
- < 500
- 500 − <1k
- 1k − <2.5k
- 2.5k − <7.5k
- 7.5k − <15k

Rwanda

Deaths (count) *attributable to* bacterial antimicrobial resistance by pathogen−drug combinations, 2019

	Resistance to 1+	AG	Aminopenicillin	Anti−pseudomonal	BL−BLI	CP	3GC	4GC	FQ	Mono INH	Macrolide	MDR in S. Typhi and Paratyphi	MDR excluding XDR in TB	Methicillin	PCN	Mono RIF	TMP−SMX	Vanco	XDR in TB
Acinetobacter baumannii	174	8.22		51.2	0.019	61.7	6.61	0.015	46.4										
Citrobacter spp.	14.3	0.46		3.37		0.583	3.28	2.05	4.57										
Enterobacter spp.	73.4	9.3		13.3		4.56		22.1	15.2								9.04		
Enterococcus faecalis	52.7								49.9									2.8	
Enterococcus faecium	57.6								42.8									14.8	
Other enterococci	16.1								15.2									0.884	
Escherichia coli	344	21.9	16.7		41	15.3	114		61.4								73.5		
Group A Streptococcus	3.38										3.22								
Group B Streptococcus	48.5								23.7		24.6				0.861				
Haemophilus influenzae	16.4		12.3				4.07												
Klebsiella pneumoniae	495	94.2			11.6	20.6	234		67.2								67.4		
Morganella spp.	0.535						0.022	0.206	0.306										
Mycobacterium tuberculosis	172									6.24			158			5.4			2.21
Proteus spp.	16.6	1.51	2.22				4.56		4.09								4.13		
Pseudomonas aeruginosa	164	11.4		14.4		46.3	60.2	5.52	26										
Salmonella Paratyphi	0.004								0.002			0.002							
Salmonella Typhi	75.3								32.9			41.3							
Non−typhoidal Salmonella	0.22								0.22										
Serratia spp.	23.7	3.01		5.63		1.54	5.17	5.05	3.28										
Shigella spp.	3.1								3.1										
Staphylococcus aureus	344								21.3		29.5			167			121	5.16	
Streptococcus pneumoniae	273				1.64	81.1	5.38		24.2		8.21				17		136		
All pathogens	2,370	150	31.3	87.9	54.3	232	437	34.9	442	5.81	64.9	41.3	158	167	17.9	5.4	411	23.7	2.21

Counts
- < 500
- 1k − <2.5k

Sao Tome and Principe

Deaths (count) *associated with* bacterial antimicrobial resistance by pathogen−drug combinations, 2019

	Resistance to 1+	AG	Aminopenicillin	Anti−pseudomonal	BL−BLI	CP	3GC	4GC	FQ	Mono INH	Macrolide	MDR in S. Typhi and Paratyphi	MDR excluding XDR in TB	Methicillin	PCN	Mono RIF	TMP−SMX	Vanco	XDR in TB
Acinetobacter baumannii	10.4	6.34		10.1	6.82	7.28	9.11	6.75	8.59										
Citrobacter spp.	0.702	0.073		0.243		0.081	0.578	0.413	0.344										
Enterobacter spp.	4.55	1.45		2.02		0.736		3	2.21								3.69		
Enterococcus faecalis	2.45								2.37									0.236	
Enterococcus faecium	4.06								4.04									1.06	
Other enterococci	1.56								1.56									0.118	
Escherichia coli	20.5	7.45	19.8		16.3	1.21	7.65		7.98								18.1		
Group A Streptococcus	0.482										0.482								
Group B Streptococcus	3.7								1.59		2.98				0.051				
Haemophilus influenzae	0.644		0.464				0.283												
Klebsiella pneumoniae	24.7	15.7			23.1	0.676	20.3		17.4								22.6		
Morganella spp.	0.044						0.01	0.009	0.041										
Mycobacterium tuberculosis	1.92									0.379			1.52			0.005			0.021
Proteus spp.	2.15	1.54	1.82				0.522		0.999								1.61		
Pseudomonas aeruginosa	11	5.09		5.4		5.45	7.07	3.58	7.14										
Salmonella Paratyphi	0.003								0.003			0.0002							
Salmonella Typhi	3.6								0.744			3.09							
Non−typhoidal Salmonella	0.002								0.002										
Serratia spp.	1.63	0.541		0.299		0.119	1.35	0.97	0.499										
Shigella spp.	0.028								0.028										
Staphylococcus aureus	28.2								8.06		16.9			15.5			16.8	0.354	
Streptococcus pneumoniae	19.3				3.53	6.35	2.58		1.24		5.95				3.77		18.6		
All pathogens	142	38.2	22.1	18.1	49.8	21.9	49.4	14.7	64.9	0.379	26.3	3.09	1.52	15.5	3.82	0.005	81.3	1.77	0.021

Counts

< 500

Sao Tome and Principe

Deaths (count) *attributable to* bacterial antimicrobial resistance by pathogen−drug combinations, 2019

	Resistance to 1+	AG	Aminopenicillin	Anti−pseudomonal	BL−BLI	CP	3GC	4GC	FQ	Mono INH	Macrolide	MDR in S. Typhi and Paratyphi	MDR excluding XDR in TB	Methicillin	PCN	Mono RIF	TMP−SMX	Vanco	XDR in TB
Acinetobacter baumannii	3.24	0.276		0.63	0.0004	1.18	0.069	0.0003	1.09										
Citrobacter spp.	0.204	0.003		0.046		0.018	0.043	0.045	0.049										
Enterobacter spp.	1.02	0.082		0.191		0.15		0.172	0.222								0.199		
Enterococcus faecalis	0.659								0.589									0.07	
Enterococcus faecium	1.03								0.779									0.247	
Other enterococci	0.323								0.301									0.022	
Escherichia coli	4.68	0.456	0.297		0.833	0.258	0.961		0.741								1.13		
Group A Streptococcus	0.046										0.048								
Group B Streptococcus	0.568								0.27		0.29				0.014				
Haemophilus influenzae	0.151		0.068				0.083												
Klebsiella pneumoniae	6.46	1.1			0.364	0.216	2.69		1.07								1.02		
Morganella spp.	0.01						0.001	0.001	0.008										
Mycobacterium tuberculosis	0.981									0.057			0.908			0.001			0.013
Proteus spp.	0.311	0.076	0.031				0.096		0.059								0.049		
Pseudomonas aeruginosa	2.72	0.135		0.447		0.885	0.426	0.048	0.786										
Salmonella Paratyphi	0.0007								0.0006			0.00002							
Salmonella Typhi	0.538								0.141			0.379							
Non−typhoidal Salmonella	0.0005								0.0005										
Serratia spp.	0.428	0.031		0.065		0.033	0.117	0.134	0.051										
Shigella spp.	0.006								0.006										
Staphylococcus aureus	6.43								0.318		0.618			3.68			1.72	0.097	
Streptococcus pneumoniae	3.75				0.073	1.36	0.036		0.144		0.221				0.088		1.82		
All pathogens	33.6	2.15	0.395	1.38	1.27	4.1	4.53	0.4	6.63	0.059	1.17	0.397	0.908	3.68	0.102	0.001	5.95	0.434	0.013

Counts
< 500

Senegal

Deaths (count) *associated with* bacterial antimicrobial resistance by pathogen−drug combinations, 2019

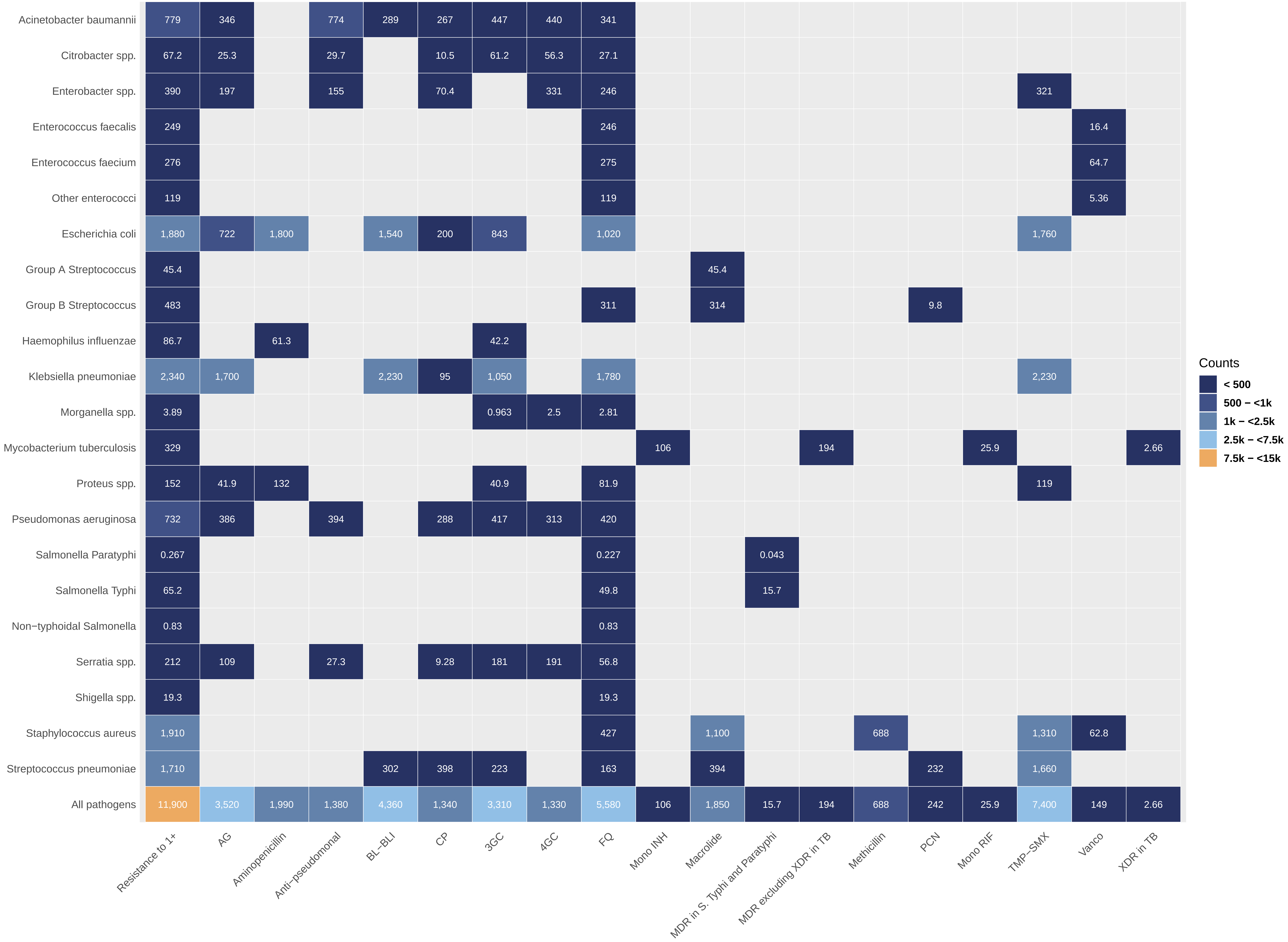

Senegal

Deaths (count) *attributable to* bacterial antimicrobial resistance by pathogen−drug combinations, 2019

	Resistance to 1+	AG	Aminopenicillin	Anti−pseudomonal	BL−BLI	CP	3GC	4GC	FQ	Mono INH	Macrolide	MDR in S. Typhi and Paratyphi	MDR excluding XDR in TB	Methicillin	PCN	Mono RIF	TMP−SMX	Vanco	XDR in TB
Acinetobacter baumannii	221	17.5		113	0.004	46.5	0.032	0.015	44										
Citrobacter spp.	20	1.33		5.11		2.28	1.96	5.95	3.35										
Enterobacter spp.	94.3	10.3		11.6		13.5		21.4	23.1								14.3		
Enterococcus faecalis	65.7								61.3									4.38	
Enterococcus faecium	68.7								53.4									15.2	
Other enterococci	24.3								23.3									1.07	
Escherichia coli	459	42.5	18.5		61.4	40.1	95.2		94.9								106		
Group A Streptococcus	4.39										4.59								
Group B Streptococcus	84.5								54.9		27.5				2.04				
Haemophilus influenzae	20.7		8.48				12.2												
Klebsiella pneumoniae	540	114			82.1	26.9	120		103								94.4		
Morganella spp.	0.916						0.036	0.422	0.458										
Mycobacterium tuberculosis	141									17.6			116			7.28			1.63
Proteus spp.	22	1.82	2.27				8.04		5.48								4.34		
Pseudomonas aeruginosa	174	10.4		44.1		47.2	18.6	6.75	46.4										
Salmonella Paratyphi	0.053								0.047			0.006							
Salmonella Typhi	12.6								10.4			2.11							
Non−typhoidal Salmonella	0.175								0.175										
Serratia spp.	56.5	6.16		5.68		2.64	4.24	32.1	5.61										
Shigella spp.	4.06								4.06										
Staphylococcus aureus	372								16.7		41.3			154			144	16.5	
Streptococcus pneumoniae	311				8.2	87	8.18		19.6		14.6				5.29		168		
All pathogens	2,700	205	29.3	180	152	266	268	66.7	570	16.8	87.9	2.17	116	154	7.33	7.28	531	37.2	1.63

Counts
- < 500
- 500 − <1k
- 2.5k − <7.5k

Seychelles

Deaths (count) *associated with* bacterial antimicrobial resistance by pathogen−drug combinations, 2019

	Resistance to 1+	AG	Aminopenicillin	Anti−pseudomonal	BL−BLI	CP	3GC	4GC	FQ	Mono INH	Macrolide	MDR in S. Typhi and Paratyphi	MDR excluding XDR in TB	Methicillin	PCN	Mono RIF	TMP−SMX	Vanco	XDR in TB
Acinetobacter baumannii	8.2	5.78		7.5	5.02	6.26	6	7.86	6.63										
Citrobacter spp.	0.658	0.178		0.096		0.134	0.415	0.487	0.202										
Enterobacter spp.	2.96	0.769		0.954		0.98		1.24	1.01								2.02		
Enterococcus faecalis	2.05								1.98									0.219	
Enterococcus faecium	3.19								3.17									0.559	
Other enterococci	1.77								1.76									0.63	
Escherichia coli	13.5	1.81	13.2		9.62	1.63	5.3		6.58								8.96		
Group A Streptococcus	0.511										0.511								
Group B Streptococcus	0.952								0.202		0.835				0.055				
Haemophilus influenzae	0.863		0.798				0.224												
Klebsiella pneumoniae	8.69	1.3			6.84	1.79	6.62		4.55								5.32		
Morganella spp.	0.04						0.022	0.007	0.026										
Mycobacterium tuberculosis	0.046									0.02			0.019			0.003			0.004
Proteus spp.	1.43	0.236	1.37				0.361		0.441								0.725		
Pseudomonas aeruginosa	4.65	1.47		1.69		3.31	2.16	1.92	2										
Salmonella Paratyphi	0.026								0.025			0.0009							
Salmonella Typhi	0.332								0.29			0.047							
Non−typhoidal Salmonella	0.146								0.146										
Serratia spp.	0.531	0.093		0.088		0.078	0.26	0.333	0.217										
Shigella spp.	0.006								0.006										
Staphylococcus aureus	12.4								5.11		7.85			10.7			0.979	0.814	
Streptococcus pneumoniae	12				2.02	4.24	4.1		0.473		6.93				2.78		9.27		
All pathogens	75	11.6	15.4	10.3	23.5	18.4	25.5	11.9	34.8	0.02	16.1	0.048	0.019	10.7	2.83	0.003	27.3	2.22	0.004

Counts

< 500

Seychelles

Deaths (count) *attributable to* bacterial antimicrobial resistance by pathogen−drug combinations, 2019

	Resistance to 1+	AG	Aminopenicillin	Anti−pseudomonal	BL−BLI	CP	3GC	4GC	FQ	Mono INH	Macrolide	MDR in S. Typhi and Paratyphi	MDR excluding XDR in TB	Methicillin	PCN	Mono RIF	TMP−SMX	Vanco	XDR in TB
Acinetobacter baumannii	2.55	0.264		0.298	0.0009	1.05	0.02	0.1	0.829										
Citrobacter spp.	0.183	0.01		0.017		0.032	0.03	0.067	0.027										
Enterobacter spp.	0.676	0.05		0.084		0.248		0.054	0.111								0.127		
Enterococcus faecalis	0.553								0.49									0.063	
Enterococcus faecium	0.768								0.632									0.136	
Other enterococci	0.406								0.301									0.105	
Escherichia coli	3.27	0.113	0.308		0.556	0.375	0.687		0.685								0.543		
Group A Streptococcus	0.052										0.048								
Group B Streptococcus	0.131								0.035		0.087				0.01				
Haemophilus influenzae	0.183		0.121				0.063												
Klebsiella pneumoniae	2.4	0.128			0.174	0.478	0.96		0.359								0.298		
Morganella spp.	0.01						0.004	0.002	0.005										
Mycobacterium tuberculosis	0.018									0.003			0.011			0.0009			0.002
Proteus spp.	0.183	0.012	0.031				0.083		0.03								0.026		
Pseudomonas aeruginosa	1.13	0.041		0.091		0.63	0.101	0.056	0.21										
Salmonella Paratyphi	0.005								0.005			0.0001							
Salmonella Typhi	0.066								0.06			0.006							
Non−typhoidal Salmonella	0.03								0.03										
Serratia spp.	0.128	0.005		0.018		0.021	0.017	0.042	0.025										
Shigella spp.	0.001								0.001										
Staphylococcus aureus	3.68								0.224		0.294			2.88			0.08	0.196	
Streptococcus pneumoniae	2.46				0.013	0.935	0.273		0.054		0.3				0.059		0.843		
All pathogens	18.9	0.614	0.459	0.507	0.745	3.77	2.24	0.321	4.11	0.003	0.712	0.006	0.011	2.88	0.069	0.0009	1.92	0.499	0.002

Counts

< 500

Sierra Leone

Deaths (count) *associated with* bacterial antimicrobial resistance by pathogen−drug combinations, 2019

	Resistance to 1+	AG	Aminopenicillin	Anti−pseudomonal	BL−BLI	CP	3GC	4GC	FQ	Mono INH	Macrolide	MDR in S. Typhi and Paratyphi	MDR excluding XDR in TB	Methicillin	PCN	Mono RIF	TMP−SMX	Vanco	XDR in TB
Acinetobacter baumannii	446	195		444	255	171	291	148	176										
Citrobacter spp.	51.1	20.4		22.1		20.1	46.6	41.9	19.3										
Enterobacter spp.	274	134		105		51.7		239	177								202		
Enterococcus faecalis	126								123									10.7	
Enterococcus faecium	156								155									52.4	
Other enterococci	72.1								71.9									4.09	
Escherichia coli	1,310	380	1,280		1,030	150	490		660								1,140		
Group A Streptococcus	35										35								
Group B Streptococcus	405								203		307				14.4				
Haemophilus influenzae	114		80				57.9												
Klebsiella pneumoniae	1,890	1,360			1,780	67.6	1,250		1,390								1,760		
Morganella spp.	1.61						0.464	0.995	1.16										
Mycobacterium tuberculosis	203									74.4			126			0.806			1.7
Proteus spp.	86.6	32.9	72.7				21.8		50.6								70.7		
Pseudomonas aeruginosa	558	260		276		187	380	248	307										
Salmonella Paratyphi	0.023								0.013			0.009							
Salmonella Typhi	528								67.9			496							
Non−typhoidal Salmonella	0.118								0.118										
Serratia spp.	145	65.8		21.6		7.35	123	122	37.3										
Shigella spp.	2.95								2.95										
Staphylococcus aureus	1,320								180		423			688			937	16.6	
Streptococcus pneumoniae	2,000				355	491	415		148		393				244		1,950		
All pathogens	9,730	2,440	1,440	868	3,420	1,150	3,070	799	3,770	74.4	1,160	496	126	688	259	0.806	6,050	83.8	1.7

Counts
- < 500
- 500 − <1k
- 1k − <2.5k
- 2.5k − <7.5k
- 7.5k − <15k

Sierra Leone

Deaths (count) *attributable to* bacterial antimicrobial resistance by pathogen−drug combinations, 2019

	Resistance to 1+	AG	Aminopenicillin	Anti−pseudomonal	BL−BLI	CP	3GC	4GC	FQ	Mono INH	Macrolide	MDR in S. Typhi and Paratyphi	MDR excluding XDR in TB	Methicillin	PCN	Mono RIF	TMP−SMX	Vanco	XDR in TB
Acinetobacter baumannii	128	9.95		64.9	0.004	30.7	0.042	0.01	22.3										
Citrobacter spp.	15.6	1.08		2.84		4.43	1.61	3.36	2.3										
Enterobacter spp.	67.1	6.93		7.9		9.99		16.6	17								8.81		
Enterococcus faecalis	33.7								30.6									3.08	
Enterococcus faecium	40.9								28.8									12.1	
Other enterococci	14.8								14									0.816	
Escherichia coli	314	22.5	21.1		50.4	31.2	56.3		63.6								68.9		
Group A Streptococcus	3.17										3.51								
Group B Streptococcus	65.9								35		28.1				2.61				
Haemophilus influenzae	27.6		10.8				16.8												
Klebsiella pneumoniae	469	93.8			46.7	20	149		82.8								77		
Morganella spp.	0.383						0.023	0.171	0.189										
Mycobacterium tuberculosis	87.8									10.5			74.9			0.226			1.04
Proteus spp.	12.6	1.4	1.23				4.15		3.34								2.55		
Pseudomonas aeruginosa	138	7.35		33.3		30.6	25	7.48	34.2										
Salmonella Paratyphi	0.004								0.003			0.001							
Salmonella Typhi	75								11.4			59.5							
Non−typhoidal Salmonella	0.025								0.025										
Serratia spp.	38.6	3.68		4.57		2.12	4.83	19.6	3.69										
Shigella spp.	0.62								0.62										
Staphylococcus aureus	304								8.14		15.3			170			106	4.36	
Streptococcus pneumoniae	373				6.7	109	23		17.4		15.2				3.27		199		
All pathogens	2,210	147	33.1	113	104	238	280	47.2	375	11.4	61.9	60.6	74.9	170	5.88	0.226	462	20.4	1.04

Counts
- < 500
- 1k − <2.5k

South Africa

Deaths (count) *associated with* bacterial antimicrobial resistance by pathogen−drug combinations, 2019

	Resistance to 1+	AG	Aminopenicillin	Anti−pseudomonal	BL−BLI	CP	3GC	4GC	FQ	Mono INH	Macrolide	MDR in S. Typhi and Paratyphi	MDR excluding XDR in TB	Methicillin	PCN	Mono RIF	TMP−SMX	Vanco	XDR in TB
Acinetobacter baumannii	3,210	1,700		3,080	2,160	2,420	3,130	2,850	2,960										
Citrobacter spp.	108	6.86		32.4		43.9	58.5	32.7	36										
Enterobacter spp.	1,320	46.6		164		265		143	132								1,230		
Enterococcus faecalis	553								530									50.7	
Enterococcus faecium	1,250								1,250									79.2	
Other enterococci	620								618									66.9	
Escherichia coli	5,370	139	4,750		2,640	153	1,710		2,080								4,650		
Group A Streptococcus	52.9										52.9								
Group B Streptococcus	1,220								109		1,180				18.5				
Haemophilus influenzae	172		147				44.7												
Klebsiella pneumoniae	6,650	348			5,400	1,260	5,920		2,240								5,940		
Morganella spp.	8.69						0.845	0.554	8.5										
Mycobacterium tuberculosis	3,020									1,430			1,120			454			15.3
Proteus spp.	599	7.57	541				128		33.5								448		
Pseudomonas aeruginosa	2,380	156		1,020		1,870	835	1,030	1,110										
Salmonella Paratyphi	0.28								0.279			0.002							
Salmonella Typhi	267								102			179							
Non−typhoidal Salmonella	10.3								10.3										
Serratia spp.	295	239		32.3		42.8	48.6	29.8	67.6										
Shigella spp.	18.4								18.4										
Staphylococcus aureus	5,990								1,710		4,400			3,660			2,010	82.3	
Streptococcus pneumoniae	5,860				870	2,340	1,260		248		2,680				2,910		4,500		
All pathogens	39,000	2,640	5,430	4,340	11,100	8,390	13,100	4,090	13,300	1,430	8,320	179	1,120	3,660	2,930	454	18,800	279	15.3

Counts
- < 500
- 500 − <1k
- 1k − <2.5k
- 2.5k − <7.5k
- 7.5k − <15k
- 15k − <25k
- >=25k

South Africa

Deaths (count) *attributable to* bacterial antimicrobial resistance by pathogen−drug combinations, 2019

	Resistance to 1+	AG	Aminopenicillin	Anti−pseudomonal	BL−BLI	CP	3GC	4GC	FQ	Mono INH	Macrolide	MDR in S. Typhi and Paratyphi	MDR excluding XDR in TB	Methicillin	PCN	Mono RIF	TMP−SMX	Vanco	XDR in TB
Acinetobacter baumannii	1,020	69.4		129	0.075	394	34	1.18	389										
Citrobacter spp.	32	0.437		5.8		12.3	5.06	2.24	6.17										
Enterobacter spp.	213	5.34		12.6		69.4		5.26	20								102		
Enterococcus faecalis	149								133									16	
Enterococcus faecium	282								261									21.4	
Other enterococci	130								118									11.9	
Escherichia coli	1,160	12.5	165		157	38.2	242		211								335		
Group A Streptococcus	5.05										5.35								
Group B Streptococcus	151								17.3		128				5.14				
Haemophilus influenzae	37		23.8				13.2												
Klebsiella pneumoniae	1,890	43.7			38	332	924		183								363		
Morganella spp.	1.88						0.122	0.088	1.67										
Mycobacterium tuberculosis	1,030									215			669			127			9.35
Proteus spp.	68.7	0.812	12.2				31.6		3.6								20.3		
Pseudomonas aeruginosa	568	4.87		52.8		357	14.5	21.2	117										
Salmonella Paratyphi	0.057								0.057			0.0002							
Salmonella Typhi	43.1								19.7			22.6							
Non−typhoidal Salmonella	2.16								2.16										
Serratia spp.	44.1	15.2		4.86		11.1	2.65	2.22	7.59										
Shigella spp.	3.87								3.87										
Staphylococcus aureus	1,400								72.5		175			929			197	22.5	
Streptococcus pneumoniae	1,270				6.46	526	30.5		28.3		105				172		406		
All pathogens	9,490	155	201	205	202	1,740	1,300	32.2	1,600	222	409	24.9	669	929	177	127	1,420	71.9	9.35

Counts
- < 500
- 500 − <1k
- 1k − <2.5k
- 7.5k − <15k

South Sudan

Deaths (count) *associated with* bacterial antimicrobial resistance by pathogen−drug combinations, 2019

	Resistance to 1+	AG	Aminopenicillin	Anti−pseudomonal	BL−BLI	CP	3GC	4GC	FQ	Mono INH	Macrolide	MDR in S. Typhi and Paratyphi	MDR excluding XDR in TB	Methicillin	PCN	Mono RIF	TMP−SMX	Vanco	XDR in TB
Acinetobacter baumannii	582	189		559	395	146	426	248	190										
Citrobacter spp.	128	54.8		53.1		25.2	114	107	69.9										
Enterobacter spp.	495	204		184		84.8		461	276								373		
Enterococcus faecalis	161								159									10.9	
Enterococcus faecium	136								135									29.6	
Other enterococci	73.5								73.2									5.74	
Escherichia coli	1,430	368	1,380		1,040	169	540		583								1,180		
Group A Streptococcus	56.5										56.5								
Group B Streptococcus	779								447		589				31.5				
Haemophilus influenzae	335		320				91												
Klebsiella pneumoniae	1,770	1,350			1,720	20.1	1,200		1,070								1,550		
Morganella spp.	1.82						0.459	1.17	1.31										
Mycobacterium tuberculosis	456									65.4			372			14.4			5.05
Proteus spp.	99.1	49.9	80.8				22.1		63								89.2		
Pseudomonas aeruginosa	401	149		144		87.2	322	205	105										
Salmonella Paratyphi	0.024								0.002			0.022							
Salmonella Typhi	186								92.1			101							
Non−typhoidal Salmonella	0.267								0.267										
Serratia spp.	210	116		61.7		9.36	150	171	57										
Shigella spp.	136								136										
Staphylococcus aureus	953								221		558			287			725	19.1	
Streptococcus pneumoniae	3,120				510	846	519		1,610		441				459		2,850		
All pathogens	11,500	2,480	1,780	1,000	3,670	1,390	3,380	1,190	5,280	65.4	1,640	101	372	287	491	14.4	6,770	65.3	5.05

Counts
- < 500
- 500 − <1k
- 1k − <2.5k
- 2.5k − <7.5k
- 7.5k − <15k

South Sudan

Deaths (count) *attributable to* bacterial antimicrobial resistance by pathogen−drug combinations, 2019

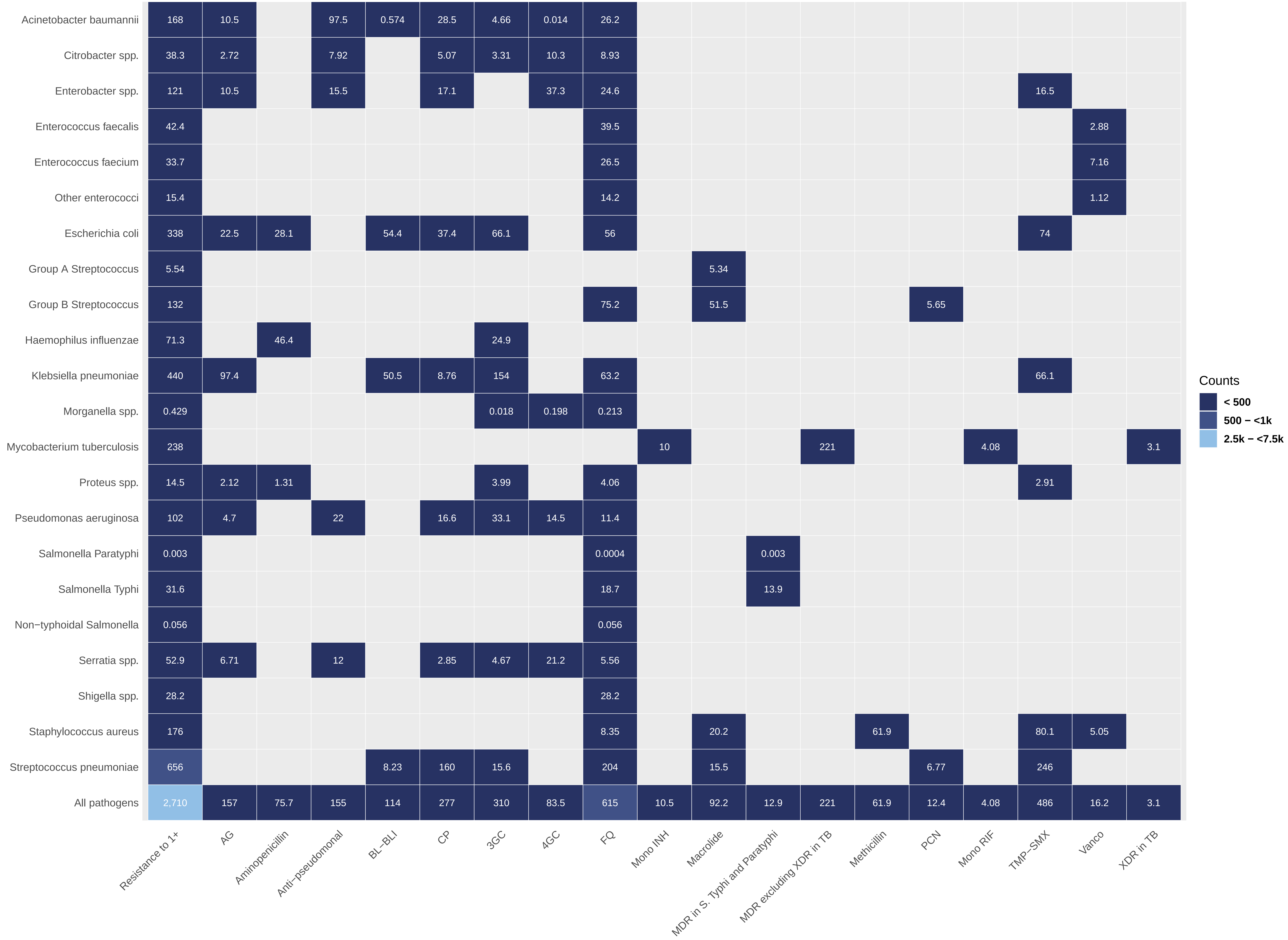

Swaziland

Deaths (count) *associated with* bacterial antimicrobial resistance by pathogen−drug combinations, 2019

	Resistance to 1+	AG	Aminopenicillin	Anti−pseudomonal	BL−BLI	CP	3GC	4GC	FQ	Mono INH	Macrolide	MDR in S. Typhi and Paratyphi	MDR excluding XDR in TB	Methicillin	PCN	Mono RIF	TMP−SMX	Vanco	XDR in TB
Acinetobacter baumannii	84.9	31.7		83.3	50.4	48.7	77.3	60.6	61.7										
Citrobacter spp.	3.83	0.446		1.38		0.609	2.48	2.42	1.32										
Enterobacter spp.	27.2	5.17		8.22		4.99		11.7	7.83								23.7		
Enterococcus faecalis	14.4								13.9									1.07	
Enterococcus faecium	25.7								25.6									4.59	
Other enterococci	9.47								9.44									0.731	
Escherichia coli	138	36.9	133		79.1	9.5	61		38								114		
Group A Streptococcus	2.2										2.2								
Group B Streptococcus	28.4								3.83		27				1.55				
Haemophilus influenzae	8.91		7.83				2.43												
Klebsiella pneumoniae	186	75.3			163	0.764	160		92.4								170		
Morganella spp.	0.262						0.039	0.124	0.206										
Mycobacterium tuberculosis	171									28.3			124			17.2			1.7
Proteus spp.	13.1	0.987	11.6				2.79		1.54								10		
Pseudomonas aeruginosa	59.4	18.6		36.6		32.1	34.4	24	28.3										
Salmonella Paratyphi	0.008								0.007			0.0004							
Salmonella Typhi	14.8								5.78			9.87							
Non−typhoidal Salmonella	0.062								0.062										
Serratia spp.	8.73	5.39		1.46		0.764	2.78	4.63	2.04										
Shigella spp.	4.3								4.3										
Staphylococcus aureus	155								44		47.6			36.6			126	1.98	
Streptococcus pneumoniae	169				37.7	48.1	25.6		17.3		58.6				71.2		153		
All pathogens	1,120	174	153	131	330	146	369	104	358	28.3	135	9.87	124	36.6	72.7	17.2	596	8.37	1.7

Counts

< 500

500 − <1k

1k − <2.5k

Swaziland

Deaths (count) *attributable to* bacterial antimicrobial resistance by pathogen−drug combinations, 2019

	Resistance to 1+	AG	Aminopenicillin	Anti−pseudomonal	BL−BLI	CP	3GC	4GC	FQ	Mono INH	Macrolide	MDR in S. Typhi and Paratyphi	MDR excluding XDR in TB	Methicillin	PCN	Mono RIF	TMP−SMX	Vanco	XDR in TB
Acinetobacter baumannii	26.2	1.41		8.3	0.0007	8.02	0.423	0.003	8.11										
Citrobacter spp.	1.08	0.025		0.267		0.15	0.165	0.273	0.198										
Enterobacter spp.	5.35	0.351		0.774		1.13		0.659	0.851								1.59		
Enterococcus faecalis	3.82								3.5									0.328	
Enterococcus faecium	6.2								5.1									1.1	
Other enterococci	1.97								1.83									0.14	
Escherichia coli	31.9	2.33	4.02		3.83	2.14	8.66		3.54								7.34		
Group A Streptococcus	0.219										0.207								
Group B Streptococcus	3.73								0.608		2.92				0.283				
Haemophilus influenzae	1.92		1.22				0.697												
Klebsiella pneumoniae	48.3	5.57			1.9	0.476	25.4		6.05								8.93		
Morganella spp.	0.059						0.002	0.021	0.037										
Mycobacterium tuberculosis	82.5									4.43			72.1			4.81			1.04
Proteus spp.	1.55	0.062	0.273				0.658		0.133								0.441		
Pseudomonas aeruginosa	15	0.513		3.84		5.91	1.35	0.313	3.04										
Salmonella Paratyphi	0.002								0.002			0.00006							
Salmonella Typhi	2.41								1.13			1.24							
Non−typhoidal Salmonella	0.013								0.013										
Serratia spp.	1.7	0.324		0.272		0.215	0.111	0.56	0.211										
Shigella spp.	0.899								0.899										
Staphylococcus aureus	27.2								1.85		1.73			7.94			15.1	0.57	
Streptococcus pneumoniae	34.1				0.998	10.2	0.738		2		2.12				4.13		14		
All pathogens	296	10.6	5.49	13.5	6.73	28.2	38.2	1.83	39.1	4.44	6.8	1.3	72.1	7.94	4.41	4.81	47.4	2.14	1.04

Counts

< 500

Tanzania

Deaths (count) *associated with* bacterial antimicrobial resistance by pathogen−drug combinations, 2019

	Resistance to 1+	AG	Aminopenicillin	Anti−pseudomonal	BL−BLI	CP	3GC	4GC	FQ	Mono INH	Macrolide	MDR in S. Typhi and Paratyphi	MDR excluding XDR in TB	Methicillin	PCN	Mono RIF	TMP−SMX	Vanco	XDR in TB
Acinetobacter baumannii	2,640	1,080		2,460	2,080	1,490	2,400	1,310	2,230										
Citrobacter spp.	284	101		126		57.8	242	161	201										
Enterobacter spp.	1,550	615		680		257		1,360	852								1,170		
Enterococcus faecalis	1,150								1,140									68.3	
Enterococcus faecium	1,010								997									269	
Other enterococci	450								449									19.2	
Escherichia coli	6,850	1,560	6,450		5,330	781	3,400		2,600								5,840		
Group A Streptococcus	270										270								
Group B Streptococcus	2,050								1,100		1,580				9.6				
Haemophilus influenzae	618		557				167												
Klebsiella pneumoniae	9,460	6,880			8,900	74.5	6,550		7,030								8,880		
Morganella spp.	9.72						2.2	5.55	7.65										
Mycobacterium tuberculosis	1,780									166			1,160			442			15.7
Proteus spp.	598	218	529				111		329								517		
Pseudomonas aeruginosa	2,930	903		1,030		842	2,080	919	1,900										
Salmonella Paratyphi	0.324								0.013			0.309							
Salmonella Typhi	3,610								1,720			3,030							
Non−typhoidal Salmonella	0.171								0.171										
Serratia spp.	617	317		202		40.5	382	377	176										
Shigella spp.	11.4								11.4										
Staphylococcus aureus	9,100								6,700		4,580			3,900			6,550	89.9	
Streptococcus pneumoniae	8,990				801	2,320	1,170		1,240		1,670				3,390		8,690		
All pathogens	54,000	11,700	7,540	4,490	17,100	5,860	16,500	4,130	28,700	166	8,110	3,030	1,160	3,900	3,400	442	31,600	446	15.7

Counts
- < 500
- 500 − <1k
- 1k − <2.5k
- 2.5k − <7.5k
- 7.5k − <15k
- 15k − <25k
- >=25k

Tanzania

Deaths (count) *attributable to* bacterial antimicrobial resistance by pathogen−drug combinations, 2019

	Resistance to 1+	AG	Aminopenicillin	Anti−pseudomonal	BL−BLI	CP	3GC	4GC	FQ	Mono INH	Macrolide	MDR in S. Typhi and Paratyphi	MDR excluding XDR in TB	Methicillin	PCN	Mono RIF	TMP−SMX	Vanco	XDR in TB
Acinetobacter baumannii	816	46.5		180	4.61	242	43.6	0.072	298										
Citrobacter spp.	84.3	4.6		17.7		10.2	13.1	10.6	28										
Enterobacter spp.	373	31.9		59.7		52.8		94.3	79.3								54.6		
Enterococcus faecalis	301								284									17.5	
Enterococcus faecium	255								191									63.5	
Other enterococci	92.2								88.2									4.03	
Escherichia coli	1,660	95.9	78.4		237	174	452		245								381		
Group A Streptococcus	24.9										25								
Group B Streptococcus	334								187		140				3.55				
Haemophilus influenzae	132		85				47.4												
Klebsiella pneumoniae	2,340	472			209	37.6	816		416								386		
Morganella spp.	2.27						0.093	0.899	1.28										
Mycobacterium tuberculosis	853									26			692			124			9.62
Proteus spp.	80.7	9.41	9.52				21.8		21.5								18.5		
Pseudomonas aeruginosa	730	23.7		115		132	202	31.1	226										
Salmonella Paratyphi	0.046								0.004			0.041							
Salmonella Typhi	613								276			334							
Non−typhoidal Salmonella	0.036								0.036										
Serratia spp.	146	18.6		39.5		11.5	18	41.3	17.8										
Shigella spp.	2.39								2.39										
Staphylococcus aureus	1,930								264		136			890			610	27.9	
Streptococcus pneumoniae	1,760				9.7	498	32.3		141		60				190		829		
All pathogens	12,500	703	173	412	460	1,160	1,650	178	2,770	25.7	366	328	692	890	193	124	2,280	113	9.62

Counts
- < 500
- 500 − <1k
- 1k − <2.5k
- 2.5k − <7.5k
- 7.5k − <15k

The Gambia

Deaths (count) *associated with* bacterial antimicrobial resistance by pathogen−drug combinations, 2019

	Resistance to 1+	AG	Aminopenicillin	Anti−pseudomonal	BL−BLI	CP	3GC	4GC	FQ	Mono INH	Macrolide	MDR in S. Typhi and Paratyphi	MDR excluding XDR in TB	Methicillin	PCN	Mono RIF	TMP−SMX	Vanco	XDR in TB
Acinetobacter baumannii	126	56.9		126	68.9	47.4	74	44.5	52.1										
Citrobacter spp.	10.7	4.33		4.72		4.36	9.91	8.85	4.08										
Enterobacter spp.	59.7	28.1		23.1		11.9		51.4	38								46.4		
Enterococcus faecalis	31.8								30.9									2.75	
Enterococcus faecium	44								43.8									10.6	
Other enterococci	18.2								18.1									0.904	
Escherichia coli	276	73	271		215	27.8	48.2		92.8								240		
Group A Streptococcus	7.35										7.35								
Group B Streptococcus	63.7								31.8		48				2.21				
Haemophilus influenzae	10.9		5.97				6.95												
Klebsiella pneumoniae	353	232			349	7.42	170		197								265		
Morganella spp.	0.427						0.121	0.255	0.314										
Mycobacterium tuberculosis	51.3									11			37.6			2.19			0.515
Proteus spp.	23.7	9.17	19.9				1.78		14.1								19.3		
Pseudomonas aeruginosa	108	51		61.6		40.8	62.1	49.2	59.5										
Salmonella Paratyphi	0.03								0.027			0.004							
Salmonella Typhi	55.4								10.8			46.4							
Non−typhoidal Salmonella	0.003								0.003										
Serratia spp.	33.9	17.5		5.04		1.54	28.9	28.4	8.94										
Shigella spp.	1.05								1.05										
Staphylococcus aureus	219								22.7		50.5			52.1			175	10.8	
Streptococcus pneumoniae	341				46.5	65.1	37.2		16.4		15.5				4.26		339		
All pathogens	1,840	472	296	220	679	206	439	183	642	11	121	46.4	37.6	52.1	6.47	2.19	1,090	25	0.515

Counts
- < 500
- 500 − <1k
- 1k − <2.5k

The Gambia

Deaths (count) *attributable to* bacterial antimicrobial resistance by pathogen−drug combinations, 2019

	Resistance to 1+	AG	Aminopenicillin	Anti−pseudomonal	BL−BLI	CP	3GC	4GC	FQ	Mono INH	Macrolide	MDR in S. Typhi and Paratyphi	MDR excluding XDR in TB	Methicillin	PCN	Mono RIF	TMP−SMX	Vanco	XDR in TB
Acinetobacter baumannii	35.9	2.94		18.1	0.0009	8.32	0.006	0.003	6.59										
Citrobacter spp.	3.28	0.234		0.592		0.959	0.33	0.691	0.483										
Enterobacter spp.	14.6	1.45		1.72		2.29		3.46	3.62								2.07		
Enterococcus faecalis	8.51								7.71									0.797	
Enterococcus faecium	11								8.5									2.49	
Other enterococci	3.73								3.55									0.183	
Escherichia coli	60.2	4.52	5.69		13.8	6.23	5.39		9								15.6		
Group A Streptococcus	0.744										0.679								
Group B Streptococcus	10.3								5.5		4.24				0.405				
Haemophilus influenzae	2.79		0.755				2.03												
Klebsiella pneumoniae	81.3	16.9			17.3	2.7	21.3		12								11.2		
Morganella spp.	0.101						0.006	0.043	0.052										
Mycobacterium tuberculosis	25.1									1.67			22.4			0.613			0.315
Proteus spp.	2.82	0.389	0.427				0.398		0.943								0.675		
Pseudomonas aeruginosa	26.1	1.4		7.52		6.84	2.58	1.13	6.61										
Salmonella Paratyphi	0.006								0.006			0.0005							
Salmonella Typhi	8.49								2.14			5.98							
Non−typhoidal Salmonella	0.0007								0.0007										
Serratia spp.	9.01	0.955		1.05		0.449	1.11	4.53	0.874										
Shigella spp.	0.219								0.219										
Staphylococcus aureus	40.7								0.973		2.02			12.3			22.2	3.21	
Streptococcus pneumoniae	56.8				1.2	14.8	1.51		1.91		0.668				0.041		36.7		
All pathogens	402	28.8	6.86	28.9	32.2	42.6	34.7	9.86	70.6	1.63	7.81	6	22.4	12.3	0.445	0.613	88.4	6.68	0.315

Counts
< 500

Togo

Deaths (count) *associated with* bacterial antimicrobial resistance by pathogen−drug combinations, 2019

	Resistance to 1+	AG	Aminopenicillin	Anti−pseudomonal	BL−BLI	CP	3GC	4GC	FQ	Mono INH	Macrolide	MDR in S. Typhi and Paratyphi	MDR excluding XDR in TB	Methicillin	PCN	Mono RIF	TMP−SMX	Vanco	XDR in TB
Acinetobacter baumannii	333	150		329	133	108	206	217	154										
Citrobacter spp.	32.3	11.1		13.9		4.89	28.5	26.3	12.8										
Enterobacter spp.	199	77.9		72.3		32		160	131								159		
Enterococcus faecalis	113								111									7.33	
Enterococcus faecium	131								130									29.8	
Other enterococci	52.5								52.4									2.48	
Escherichia coli	963	514	923		736	113	452		480								859		
Group A Streptococcus	21.1										21.1								
Group B Streptococcus	243								152		156				3.9				
Haemophilus influenzae	56.5		44.3				23.1												
Klebsiella pneumoniae	1,160	773			1,080	28.8	984		899								1,110		
Morganella spp.	1.19						0.269	0.705	0.882										
Mycobacterium tuberculosis	173									53.5			117			0.052			1.6
Proteus spp.	64.2	8.84	56.7				15.7		23								47.9		
Pseudomonas aeruginosa	353	148		197		147	203	147	210										
Salmonella Paratyphi	0.03								0.02			0.01							
Salmonella Typhi	259								68.4			220							
Non−typhoidal Salmonella	0.387								0.387										
Serratia spp.	93	39.3		10.3		8.58	74.7	78.2	23.5										
Shigella spp.	10.2								10.2										
Staphylococcus aureus	1,050								194		589			534			714	36.5	
Streptococcus pneumoniae	974				210	222	140		113		235				152		938		
All pathogens	6,280	1,720	1,020	622	2,160	665	2,130	629	2,770	53.5	1,000	220	117	534	156	0.052	3,830	76.1	1.6

Counts
- **< 500**
- **500 − <1k**
- **1k − <2.5k**
- **2.5k − <7.5k**

Togo

Deaths (count) *attributable to* bacterial antimicrobial resistance by pathogen−drug combinations, 2019

	Resistance to 1+	AG	Aminopenicillin	Anti−pseudomonal	BL−BLI	CP	3GC	4GC	FQ	Mono INH	Macrolide	MDR in S. Typhi and Paratyphi	MDR excluding XDR in TB	Methicillin	PCN	Mono RIF	TMP−SMX	Vanco	XDR in TB
Acinetobacter baumannii	96	7.97		48	0.002	19.4	0.399	0.01	20.5										
Citrobacter spp.	9.53	0.602		2.43		1.08	1.03	2.8	1.61										
Enterobacter spp.	47.4	3.98		5.56		6.25		10.9	13.2								7.29		
Enterococcus faecalis	29.7								27.8									1.99	
Enterococcus faecium	32.5								25.4									7.07	
Other enterococci	10.7								10.2									0.496	
Escherichia coli	236	31.4	11.9		28.2	22.4	50.5		42.8								49.1		
Group A Streptococcus	2.04										2.07								
Group B Streptococcus	42								27		13.5				0.978				
Haemophilus influenzae	13		6.33				6.65												
Klebsiella pneumoniae	308	53.3			12.6	9.24	128		55								49.7		
Morganella spp.	0.277						0.011	0.118	0.147										
Mycobacterium tuberculosis	79.3									9.09			69.7			0.015			0.98
Proteus spp.	8.49	0.449	1.11				3.39		1.62								1.9		
Pseudomonas aeruginosa	86	4.01		22.1		24.6	9.01	2.8	23.6										
Salmonella Paratyphi	0.006								0.004			0.001							
Salmonella Typhi	39.8								12.3			26.7							
Non−typhoidal Salmonella	0.08								0.08										
Serratia spp.	24.6	2.17		2.16		2.29	3.01	12.6	2.31										
Shigella spp.	2.14								2.14										
Staphylococcus aureus	236								7.79		21.1			124			74.6	8.94	
Streptococcus pneumoniae	180				6.11	48.4	5.98		13.7		8.39				3.99		93.2		
All pathogens	1,480	104	19.4	80.2	47	134	208	29.3	287	8.85	45.8	27.2	69.7	124	4.97	0.015	276	18.5	0.98

Counts
- **< 500**
- **1k − <2.5k**

Uganda

Deaths (count) *associated with* bacterial antimicrobial resistance by pathogen−drug combinations, 2019

	Resistance to 1+	AG	Aminopenicillin	Anti−pseudomonal	BL−BLI	CP	3GC	4GC	FQ	Mono INH	Macrolide	MDR in S. Typhi and Paratyphi	MDR excluding XDR in TB	Methicillin	PCN	Mono RIF	TMP−SMX	Vanco	XDR in TB
Acinetobacter baumannii	1,400	300		1,380	94.8	152	351	103	280										
Citrobacter spp.	221	58.2		96.8		12.6	190	139	130										
Enterobacter spp.	1,130	552		435		120		978	718								846		
Enterococcus faecalis	584								573									39	
Enterococcus faecium	608								601									161	
Other enterococci	276								275									15.2	
Escherichia coli	4,220	668	2,650		2,800	873	2,340		2,210								3,870		
Group A Streptococcus	223										223								
Group B Streptococcus	1,410								516		1,210				30.7				
Haemophilus influenzae	483		467				80.5												
Klebsiella pneumoniae	5,730	4,080			4,850	91.1	4,850		4,090								4,960		
Morganella spp.	5.32						1.28	3.18	3.87										
Mycobacterium tuberculosis	1,200									181			995			7.1			13.5
Proteus spp.	355	90.2	299				64		152								312		
Pseudomonas aeruginosa	1,190	189		608		237	1,020	208	296										
Salmonella Paratyphi	0.054								0.03			0.024							
Salmonella Typhi	1,820								411			1,620							
Non−typhoidal Salmonella	1.39								1.39										
Serratia spp.	529	258		203		31.6	335	376	132										
Shigella spp.	71.3								71.3										
Staphylococcus aureus	4,150								1,270		2,420			737			2,890	50.1	
Streptococcus pneumoniae	5,140				1,070	1,340	1,820		927		715				375		4,760		
All pathogens	30,700	6,190	3,410	2,730	8,810	2,860	11,100	1,810	12,700	181	4,570	1,620	995	737	406	7.1	17,600	265	13.5

Counts
- < 500
- 500 − <1k
- 1k − <2.5k
- 2.5k − <7.5k
- 7.5k − <15k
- 15k − <25k
- >=25k

Uganda

Deaths (count) *attributable to* bacterial antimicrobial resistance by pathogen−drug combinations, 2019

	Resistance to 1+	AG	Aminopenicillin	Anti−pseudomonal	BL−BLI	CP	3GC	4GC	FQ	Mono INH	Macrolide	MDR in S. Typhi and Paratyphi	MDR excluding XDR in TB	Methicillin	PCN	Mono RIF	TMP−SMX	Vanco	XDR in TB
Acinetobacter baumannii	355	18.1		255	0.017	37.9	0.088	0.057	44										
Citrobacter spp.	64.7	2.79		17.5		2.85	10.6	12.8	18.2										
Enterobacter spp.	267	28.5		36.9		24.2		72.4	68.2								37.1		
Enterococcus faecalis	154								143									11	
Enterococcus faecium	154								116									38.4	
Other enterococci	57								53.9									3.11	
Escherichia coli	1,080	39.7	5.65		77.3	193	277		222								269		
Group A Streptococcus	22.4										20.6								
Group B Streptococcus	208								85.5		117				6.41				
Haemophilus influenzae	97.5		75				22.5												
Klebsiella pneumoniae	1,510	291			58.5	39.2	642		251								226		
Morganella spp.	1.25						0.059	0.542	0.645										
Mycobacterium tuberculosis	635									27.5			596			1.98			8.29
Proteus spp.	45.7	4.18	5.69				13.2		10.6								12.1		
Pseudomonas aeruginosa	322	5.83		108		47.8	125	2.29	33.9										
Salmonella Paratyphi	0.01								0.006			0.003							
Salmonella Typhi	274								71			210							
Non−typhoidal Salmonella	0.288								0.288										
Serratia spp.	129	14.8		39.8		9.4	11.5	39.9	13.1										
Shigella spp.	14.9								14.9										
Staphylococcus aureus	659								50.8		91.5			175			326	15.6	
Streptococcus pneumoniae	1,050				11.1	289	151		112		27.8				1.33		462		
All pathogens	7,110	404	86.3	457	147	644	1,250	128	1,310	30.4	257	207	596	175	7.75	1.98	1,330	68.1	8.29

Counts
- < 500
- 500 − <1k
- 1k − <2.5k
- 2.5k − <7.5k

Zambia

Deaths (count) *associated with* bacterial antimicrobial resistance by pathogen−drug combinations, 2019

	Resistance to 1+	AG	Aminopenicillin	Anti−pseudomonal	BL−BLI	CP	3GC	4GC	FQ	Mono INH	Macrolide	MDR in S. Typhi and Paratyphi	MDR excluding XDR in TB	Methicillin	PCN	Mono RIF	TMP−SMX	Vanco	XDR in TB
Acinetobacter baumannii	788	252		742	450	357	721	438	395										
Citrobacter spp.	87.5	17.3		34		4.79	77.8	53.5	52.9										
Enterobacter spp.	467	200		200		52.3		397	274								361		
Enterococcus faecalis	299								294									16.2	
Enterococcus faecium	346								343									97.7	
Other enterococci	122								122									6.1	
Escherichia coli	2,170	908	1,810		1,520	751	881		798								1,940		
Group A Streptococcus	76.9										76.9								
Group B Streptococcus	501								166		442				11				
Haemophilus influenzae	176		169				28.6												
Klebsiella pneumoniae	2,780	2,490			2,660	14.3	2,210		1,910								2,700		
Morganella spp.	3.04						0.75	1.68	2.31										
Mycobacterium tuberculosis	679									93.3			534			44.8			7.27
Proteus spp.	189	77.4	157				35		79.5								168		
Pseudomonas aeruginosa	756	266		296		266	576	241	356										
Salmonella Paratyphi	0.019								0.004			0.015							
Salmonella Typhi	866								137			823							
Non−typhoidal Salmonella	1.09								1.09										
Serratia spp.	197	102		68		9.73	131	123	49.7										
Shigella spp.	79.5								79.5										
Staphylococcus aureus	2,640								683		1,100			734			2,370	25.7	
Streptococcus pneumoniae	2,360				372	592	328		385		319				725		2,280		
All pathogens	15,600	4,310	2,140	1,340	5,000	2,050	4,990	1,250	6,130	93.3	1,940	823	534	734	736	44.8	9,820	146	7.27

Counts
- < 500
- 500 − <1k
- 1k − <2.5k
- 2.5k − <7.5k
- 7.5k − <15k
- 15k − <25k

Zambia

Deaths (count) *attributable to* bacterial antimicrobial resistance by pathogen−drug combinations, 2019

	Resistance to 1+	AG	Aminopenicillin	Anti−pseudomonal	BL−BLI	CP	3GC	4GC	FQ	Mono INH	Macrolide	MDR in S. Typhi and Paratyphi	MDR excluding XDR in TB	Methicillin	PCN	Mono RIF	TMP−SMX	Vanco	XDR in TB
Acinetobacter baumannii	239	11.9		98	0.063	64	13.4	0.019	51.7										
Citrobacter spp.	25.7	0.781		6.21		1.08	4.96	5.31	7.38										
Enterobacter spp.	110	10.3		18.1		10.9		27.7	26								17		
Enterococcus faecalis	78.4								73.8									4.56	
Enterococcus faecium	88.5								65.4									23.1	
Other enterococci	25.1								23.8									1.26	
Escherichia coli	574	57.9	17		50.7	174	77.7		71.4								126		
Group A Streptococcus	7.54										7.39								
Group B Streptococcus	72.2								27.2		43.7				2.29				
Haemophilus influenzae	35.6		27.5				8.07												
Klebsiella pneumoniae	720	178			37.8	7.61	275		111								112		
Morganella spp.	0.712						0.04	0.283	0.39										
Mycobacterium tuberculosis	348									15.1			316			12.6			4.46
Proteus spp.	24.9	3.49	2.93				6.95		5.34								6.16		
Pseudomonas aeruginosa	193	7.33		33.8		47	57.3	7.11	40.1										
Salmonella Paratyphi	0.003								0.001			0.002							
Salmonella Typhi	121								21.6			102							
Non−typhoidal Salmonella	0.232								0.232										
Serratia spp.	47.6	5.93		13.7		2.96	6.31	13.7	4.98										
Shigella spp.	16.5								16.5										
Staphylococcus aureus	485								25.1		36			147			270	7.76	
Streptococcus pneumoniae	457				8.49	127	10.6		44.7		11.6				33.7		221		
All pathogens	3,670	276	47.4	170	97.1	435	460	54.1	616	15.9	97.8	99.7	316	147	36	12.6	751	36.7	4.46

Counts
- < 500
- 500 − <1k
- 2.5k − <7.5k

Zimbabwe

Deaths (count) *associated with* bacterial antimicrobial resistance by pathogen−drug combinations, 2019

	Resistance to 1+	AG	Aminopenicillin	Anti−pseudomonal	BL−BLI	CP	3GC	4GC	FQ	Mono INH	Macrolide	MDR in S. Typhi and Paratyphi	MDR excluding XDR in TB	Methicillin	PCN	Mono RIF	TMP−SMX	Vanco	XDR in TB
Acinetobacter baumannii	970	322		906	541	457	892	666	618										
Citrobacter spp.	53.4	7.46		24.5		8.08	38.3	32.7	18.3										
Enterobacter spp.	401	99.9		116		74.3		222	135								340		
Enterococcus faecalis	212								207									16.3	
Enterococcus faecium	291								290									51.5	
Other enterococci	101								101									8.47	
Escherichia coli	2,050	297	1,910		1,200	176	1,060		809								1,790		
Group A Streptococcus	41.1										41.1								
Group B Streptococcus	588								125		539				29.8				
Haemophilus influenzae	264		253				48.7												
Klebsiella pneumoniae	2,860	1,220			2,530	33.5	2,460		1,450								2,550		
Morganella spp.	2.54						0.37	1.42	1.9										
Mycobacterium tuberculosis	1,500									428			1,050			5.74			14.3
Proteus spp.	157	20.7	140				33.9		18.2								121		
Pseudomonas aeruginosa	838	180		585		407	511	359	356										
Salmonella Paratyphi	0.109								0.106			0.004							
Salmonella Typhi	324								86.7			261							
Non−typhoidal Salmonella	1.84								1.84										
Serratia spp.	120	73.2		19.6		12.1	37.9	70.2	26										
Shigella spp.	10								10										
Staphylococcus aureus	2,160								531		1,020			545			1,530	28.4	
Streptococcus pneumoniae	2,810				837	839	426		323		935				1,120		2,580		
All pathogens	15,800	2,220	2,300	1,650	5,110	2,010	5,520	1,350	5,110	428	2,540	261	1,050	545	1,150	5.74	8,910	105	14.3

Counts
- < 500
- 500 − <1k
- 1k − <2.5k
- 2.5k − <7.5k
- 7.5k − <15k
- 15k − <25k

Zimbabwe

Deaths (count) *attributable to* bacterial antimicrobial resistance by pathogen−drug combinations, 2019

	Resistance to 1+	AG	Aminopenicillin	Anti−pseudomonal	BL−BLI	CP	3GC	4GC	FQ	Mono INH	Macrolide	MDR in S. Typhi and Paratyphi	MDR excluding XDR in TB	Methicillin	PCN	Mono RIF	TMP−SMX	Vanco	XDR in TB
Acinetobacter baumannii	297	14.7		104	0.064	77.1	18.6	0.139	81.8										
Citrobacter spp.	15.3	0.393		4.81		1.95	2.23	3.25	2.62										
Enterobacter spp.	83.3	6.33		10.4		16.2		15.2	13.9								21		
Enterococcus faecalis	56.3								51.6									4.7	
Enterococcus faecium	70								57.7									12.3	
Other enterococci	21.2								19.6									1.61	
Escherichia coli	495	18.4	42.6		49.6	39.2	150		77.3								118		
Group A Streptococcus	3.84										3.83								
Group B Streptococcus	80								20.4		52.1				5.56				
Haemophilus influenzae	53.9		40.3				13.6												
Klebsiella pneumoniae	750	90			32	14.7	387		94.7								131		
Morganella spp.	0.578						0.013	0.237	0.328										
Mycobacterium tuberculosis	700									66.9			621			1.62			8.8
Proteus spp.	18.8	1.16	2.91				7.87		1.55								5.33		
Pseudomonas aeruginosa	215	4.9		72.3		76.8	18.7	3.87	38.6										
Salmonella Paratyphi	0.022								0.022			0.0005							
Salmonella Typhi	50.2								16.4			35.3							
Non−typhoidal Salmonella	0.386								0.386										
Serratia spp.	23.9	4.4		3.59		3.34	1.35	8.54	2.63										
Shigella spp.	2.09								2.09										
Staphylococcus aureus	372								22.1		39.9			122			180	8.35	
Streptococcus pneumoniae	576				25.9	176	10.8		37.1		33				57.2		235		
All pathogens	3,880	140	86	196	108	406	610	31.3	541	68.8	131	33.3	621	122	62.7	1.62	692	27	8.8

Counts
- **< 500**
- **500 − <1k**
- **2.5k − <7.5k**

African Region

DALYs (count) *associated with* bacterial antimicrobial resistance by pathogen−drug combinations, 2019

	Resistance to 1+	AG	Aminopenicillin	Anti−pseudomonal	BL−BLI	CP	3GC	4GC	FQ	Mono INH	Macrolide	MDR in S. Typhi and Paratyphi	MDR excluding XDR in TB	Methicillin	PCN	Mono RIF	TMP−SMX	Vanco	XDR in TB
Acinetobacter baumannii	2,090,000	904,000		2,000,000	1,230,000	861,000	1,590,000	1,220,000	1,160,000										
Citrobacter spp.	381,000	126,000		166,000		72,200	330,000	281,000	178,000										
Enterobacter spp.	2,040,000	996,000		800,000		329,000		1,680,000	1,190,000								1,620,000		
Enterococcus faecalis	937,000								923,000									60,700	
Enterococcus faecium	840,000								834,000									210,000	
Other enterococci	491,000								490,000									30,800	
Escherichia coli	8,760,000	3,010,000	8,190,000		6,480,000	946,000	4,960,000		5,000,000								7,840,000		
Group A Streptococcus	238,000										238,000								
Group B Streptococcus	4,060,000								2,320,000		2,890,000				91,900				
Haemophilus influenzae	984,000		837,000				327,000												
Klebsiella pneumoniae	11,600,000	8,120,000			10,700,000	1,340,000	9,780,000		8,110,000								10,700,000		
Morganella spp.	5,100						1,210	2,870	3,880										
Mycobacterium tuberculosis	1,850,000									596,000			1,060,000			177,000			14,200
Neisseria gonorrhoeae	10,700						140		10,700										
Proteus spp.	410,000	122,000	356,000				96,400		187,000								333,000		
Pseudomonas aeruginosa	3,310,000	1,480,000		1,600,000		1,320,000	2,230,000	1,470,000	1,700,000										
Salmonella Paratyphi	7,020								5,710			1,490							
Salmonella Typhi	3,290,000								1,260,000			2,710,000							
Non−typhoidal Salmonella	99,700								99,700										
Serratia spp.	907,000	457,000		197,000		54,300	601,000	714,000	223,000										
Shigella spp.	799,000								799,000										
Staphylococcus aureus	7,580,000								1,980,000		4,110,000			3,710,000			5,220,000	123,000	
Streptococcus pneumoniae	13,600,000				2,700,000	3,130,000	1,970,000		2,950,000		3,240,000				4,460,000		12,600,000		
All pathogens	64,300,000	15,200,000	9,380,000	4,750,000	21,100,000	8,050,000	21,900,000	5,370,000	29,400,000	596,000	10,500,000	2,710,000	1,060,000	3,710,000	4,550,000	177,000	38,400,000	424,000	14,200

Counts
- <50k
- 50k − <100k
- 100k − <500k
- 500k − <1M
- 1M − <1.5M
- 1.5M − <2M
- >= 2M

African Region

DALYs (count) *attributable to* bacterial antimicrobial resistance by pathogen−drug combinations, 2019

	Resistance to 1+	AG	Aminopenicillin	Anti−pseudomonal	BL−BLI	CP	3GC	4GC	FQ	Mono INH	Macrolide	MDR in S. Typhi and Paratyphi	MDR excluding XDR in TB	Methicillin	PCN	Mono RIF	TMP−SMX	Vanco	XDR in TB
Acinetobacter baumannii	620,000	44,000		253,000	1,630	148,000	19,700	1,530	152,000										
Citrobacter spp.	113,000	6,560		27,000		15,600	14,000	26,400	23,400										
Enterobacter spp.	484,000	53,400		64,700		65,800		115,000	110,000								74,800		
Enterococcus faecalis	247,000								230,000									16,600	
Enterococcus faecium	211,000								161,000									49,500	
Other enterococci	101,000								95,000									5,980	
Escherichia coli	2,210,000	174,000	98,100		215,000	190,000	603,000		470,000								462,000		
Group A Streptococcus	21,400										23,000								
Group B Streptococcus	682,000								403,000		256,000				19,000				
Haemophilus influenzae	217,000		124,000				93,400												
Klebsiella pneumoniae	3,170,000	578,000			133,000	293,000	1,190,000		499,000								483,000		
Morganella spp.	1,180						59.9	481	642										
Mycobacterium tuberculosis	748,000									87,000			602,000			47,500			8,470
Neisseria gonorrhoeae	1,060						32.9		1,030										
Proteus spp.	56,200	5,450	6,420				19,400		12,500								12,700		
Pseudomonas aeruginosa	824,000	41,500		176,000		225,000	151,000	43,600	186,000										
Salmonella Paratyphi	1,360								1,170			192							
Salmonella Typhi	534,000								213,000			324,000							
Non−typhoidal Salmonella	20,300								20,300										
Serratia spp.	223,000	26,400		39,300		15,800	19,400	101,000	22,000										
Shigella spp.	165,000								165,000										
Staphylococcus aureus	1,660,000								80,800		145,000			864,000			541,000	33,000	
Streptococcus pneumoniae	2,710,000				75,100	665,000	92,700		355,000		112,000				255,000		1,160,000		
All pathogens	15,000,000	929,000	228,000	560,000	425,000	1,620,000	2,200,000	287,000	3,200,000	90,600	540,000	322,000	602,000	864,000	274,000	47,500	2,730,000	105,000	8,470

Counts
- <50k
- 50k − <100k
- 100k − <500k
- 500k − <1M
- 1M − <1.5M
- 1.5M − <2M
- >= 2M

Central sub−Saharan Africa

DALYs (count) *associated with* bacterial antimicrobial resistance by pathogen−drug combinations, 2019

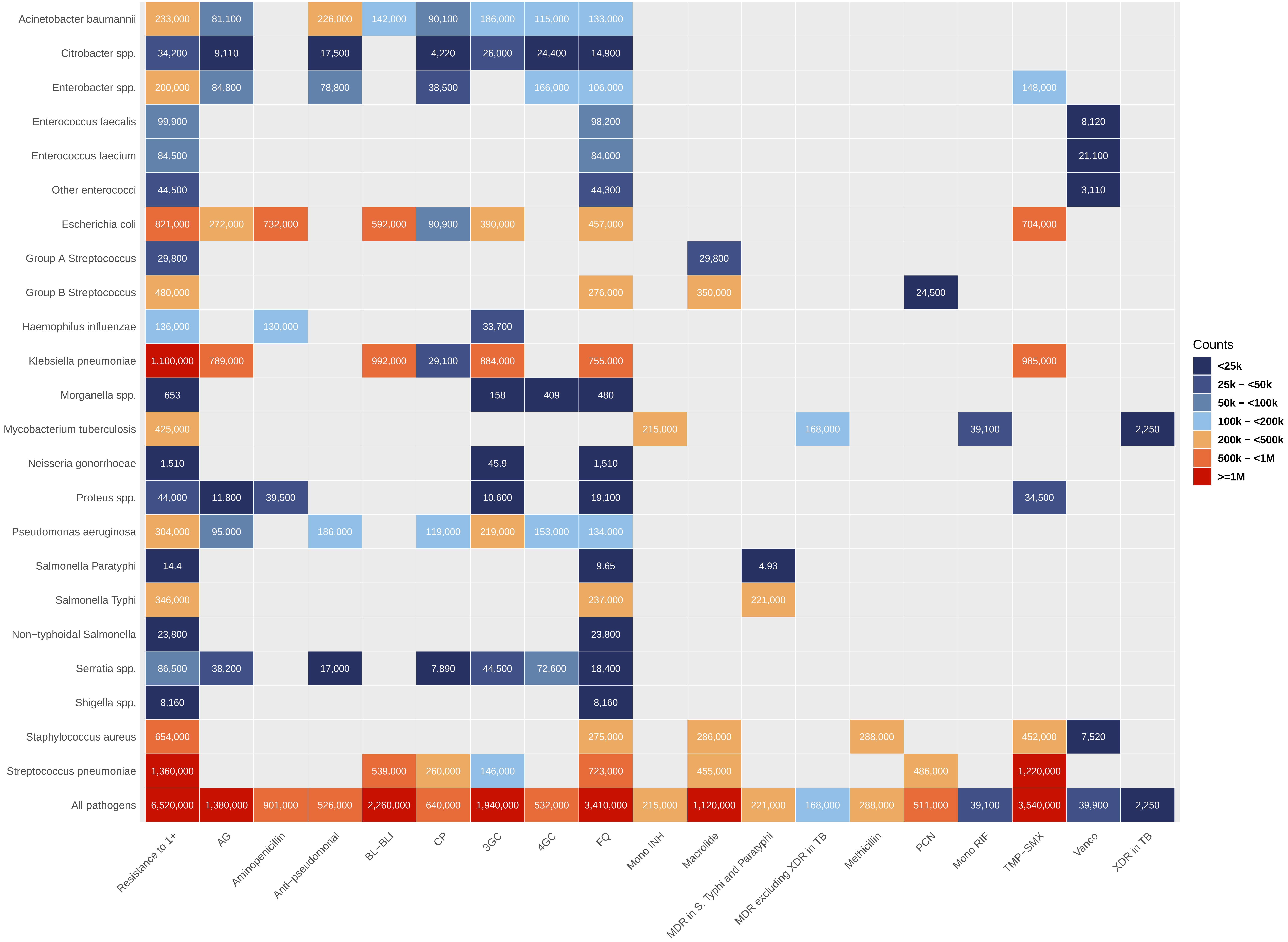

Central sub−Saharan Africa

DALYs (count) *attributable to* bacterial antimicrobial resistance by pathogen−drug combinations, 2019

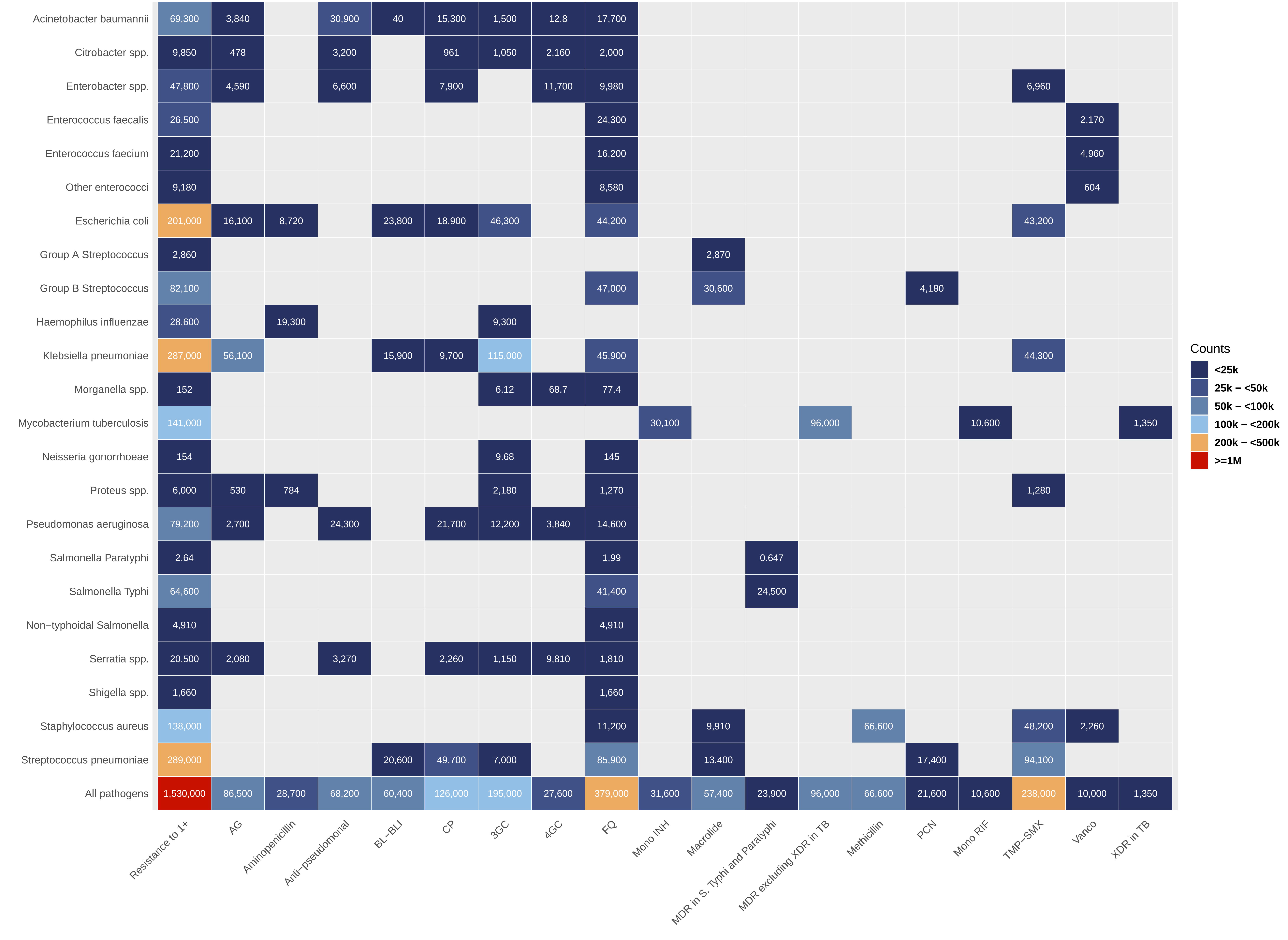

Eastern sub−Saharan Africa

DALYs (count) *associated with* bacterial antimicrobial resistance by pathogen−drug combinations, 2019

	Resistance to 1+	AG	Aminopenicillin	Anti−pseudomonal	BL−BLI	CP	3GC	4GC	FQ	Mono INH	Macrolide	MDR in S. Typhi and Paratyphi	MDR excluding XDR in TB	Methicillin	PCN	Mono RIF	TMP−SMX	Vanco	XDR in TB
Acinetobacter baumannii	716,000	249,000		645,000	508,000	252,000	606,000	409,000	406,000										
Citrobacter spp.	123,000	38,100		53,600		16,900	106,000	77,100	75,100										
Enterobacter spp.	604,000	272,000		235,000		76,100		534,000	351,000								453,000		
Enterococcus faecalis	352,000								348,000									21,900	
Enterococcus faecium	283,000								280,000									74,600	
Other enterococci	149,000								148,000									8,980	
Escherichia coli	2,610,000	601,000	2,380,000		1,780,000	261,000	1,320,000		1,280,000								2,240,000		
Group A Streptococcus	108,000										108,000								
Group B Streptococcus	1,040,000								439,000		870,000				23,400				
Haemophilus influenzae	335,000		321,000				63,600												
Klebsiella pneumoniae	3,540,000	2,570,000			3,280,000	379,000	2,950,000		2,260,000								3,190,000		
Morganella spp.	1,800						424	1,070	1,340										
Mycobacterium tuberculosis	601,000									96,400			439,000			59,300			5,860
Neisseria gonorrhoeae	3,250						78.8		3,230										
Proteus spp.	151,000	55,400	129,000				28,300		78,700								132,000		
Pseudomonas aeruginosa	910,000	269,000		362,000		244,000	715,000	299,000	406,000										
Salmonella Paratyphi	331								55.6			273							
Salmonella Typhi	1,270,000								526,000			980,000							
Non−typhoidal Salmonella	38,800								38,800										
Serratia spp.	279,000	135,000		102,000		15,300	172,000	190,000	75,400										
Shigella spp.	107,000								107,000										
Staphylococcus aureus	2,390,000								884,000		1,200,000			923,000			1,700,000	27,700	
Streptococcus pneumoniae	3,760,000				577,000	950,000	546,000		976,000		509,000				987,000		3,570,000		
All pathogens	19,400,000	4,190,000	2,830,000	1,400,000	6,150,000	2,190,000	6,510,000	1,510,000	8,690,000	96,400	2,690,000	980,000	439,000	923,000	1,010,000	59,300	11,300,000	133,000	5,860

Counts
- <25k
- 25k − <50k
- 50k − <100k
- 100k − <200k
- 200k − <500k
- 500k − <1M
- >=1M

Eastern sub−Saharan Africa

DALYs (count) *attributable to* bacterial antimicrobial resistance by pathogen−drug combinations, 2019

	Resistance to 1+	AG	Aminopenicillin	Anti−pseudomonal	BL−BLI	CP	3GC	4GC	FQ	Mono INH	Macrolide	MDR in S. Typhi and Paratyphi	MDR excluding XDR in TB	Methicillin	PCN	Mono RIF	TMP−SMX	Vanco	XDR in TB
Acinetobacter baumannii	213,000	11,900		86,200	1,550	43,000	14,700	1,420	54,500										
Citrobacter spp.	36,100	1,820		8,640		3,260	5,640	6,440	10,400										
Enterobacter spp.	144,000	14,200		20,700		15,600		40,800	32,400								20,700		
Enterococcus faecalis	92,600								86,700									5,900	
Enterococcus faecium	71,700								53,900									17,700	
Other enterococci	30,700								28,900									1,800	
Escherichia coli	639,000	36,100	37,900		72,600	57,500	172,000		124,000								140,000		
Group A Streptococcus	9,640										10,000								
Group B Streptococcus	159,000								72,600		83,200				4,790				
Haemophilus influenzae	68,400		50,600				17,800												
Klebsiella pneumoniae	962,000	186,000			44,600	81,900	369,000		138,000								143,000		
Morganella spp.	418						18.5	179	220										
Mycobacterium tuberculosis	284,000									13,700			250,000			16,000			3,510
Neisseria gonorrhoeae	329						17.3		312										
Proteus spp.	20,300	2,430	2,480				5,540		5,210								4,740		
Pseudomonas aeruginosa	235,000	7,630		50,000		43,600	75,600	11,500	46,700										
Salmonella Paratyphi	50								13.2			35.7							
Salmonella Typhi	208,000								92,600			118,000							
Non−typhoidal Salmonella	7,810								7,810										
Serratia spp.	67,100	7,960		20,200		4,580	6,400	20,600	7,570										
Shigella spp.	21,900								21,900										
Staphylococcus aureus	477,000								35,100		42,400			211,000			181,000	7,930	
Streptococcus pneumoniae	747,000				12,100	198,000	22,100		119,000		19,200				44,600		332,000		
All pathogens	4,500,000	268,000	90,900	186,000	131,000	448,000	688,000	80,800	938,000	14,400	152,000	115,000	250,000	211,000	49,400	16,000	821,000	33,400	3,510

Counts
- <25k
- 25k − <50k
- 50k − <100k
- 100k − <200k
- 200k − <500k
- 500k − <1M
- >=1M

North Africa and Middle East

DALYs (count) *associated with* bacterial antimicrobial resistance by pathogen−drug combinations, 2019

Counts: <25k; 25k − <50k; 50k − <100k; 100k − <200k; 200k − <500k

	Resistance to 1+	AG	Aminopenicillin	Anti−pseudomonal	BL−BLI	CP	3GC	4GC	FQ	Mono INH	Macrolide	MDR in S. Typhi and Paratyphi	MDR excluding XDR in TB	Methicillin	PCN	Mono RIF	TMP−SMX	Vanco	XDR in TB
Acinetobacter baumannii	31,400	27,200		30,900	30,200	27,600	30,300	29,900	28,300										
Citrobacter spp.	2,180	244		728		414	1,480	701	1,100										
Enterobacter spp.	11,700	1,830		6,960		2,710		5,920	2,820								5,020		
Enterococcus faecalis	3,140								2,720									599	
Enterococcus faecium	20,000								19,800									11,200	
Other enterococci	7,970								7,930									3,430	
Escherichia coli	70,700	12,200	68,000		43,200	9,250	23,700		34,200								51,000		
Group A Streptococcus	770										770								
Group B Streptococcus	18,700								2,500		18,100				19.5				
Haemophilus influenzae	2,610		2,410				452												
Klebsiella pneumoniae	74,800	22,200			68,900	9,970	66,200		31,900								51,800		
Morganella spp.	114						41	22.9	98.8										
Mycobacterium tuberculosis	848									141			657			4.67			45.3
Neisseria gonorrhoeae	145						5.59		143										
Proteus spp.	6,200	3,500	5,620				2,720		2,900								4,270		
Pseudomonas aeruginosa	40,400	14,700		16,500		18,900	30,100	20,200	21,000										
Salmonella Paratyphi	98.6								98.3			1.37							
Salmonella Typhi	669								602			54.9							
Non−typhoidal Salmonella	395								395										
Serratia spp.	3,310	798		742		705	2,360	1,650	483										
Shigella spp.	575								575										
Staphylococcus aureus	89,300								18,700		75,500			56,700			17,400	869	
Streptococcus pneumoniae	71,000				14,500	37,700	7,800		4,340		48,500				43,700		51,100		
All pathogens	457,000	82,700	76,100	55,900	157,000	107,000	165,000	58,400	181,000	141	143,000	56.2	657	56,700	43,700	4.67	181,000	16,100	45.3

North Africa and Middle East

DALYs (count) *attributable to* bacterial antimicrobial resistance by pathogen−drug combinations, 2019

	Resistance to 1+	AG	Aminopenicillin	Anti−pseudomonal	BL−BLI	CP	3GC	4GC	FQ	Mono INH	Macrolide	MDR in S. Typhi and Paratyphi	MDR excluding XDR in TB	Methicillin	PCN	Mono RIF	TMP−SMX	Vanco	XDR in TB
Acinetobacter baumannii	10,000	1,160		829	16.7	4,500	66.3	12	3,470										
Citrobacter spp.	627	12.4		134		98.9	141	56.2	184										
Enterobacter spp.	2,690	119		918		713		325	303								314		
Enterococcus faecalis	894								675									219	
Enterococcus faecium	5,850								3,280									2,570	
Other enterococci	1,880								1,300									578	
Escherichia coli	16,800	742	2,050		2,480	2,110	2,850		3,450								3,080		
Group A Streptococcus	69.7										71.9								
Group B Streptococcus	2,170								389		1,780				18.4				
Haemophilus influenzae	532		399				132												
Klebsiella pneumoniae	21,000	1,820			1,080	2,490	10,600		2,330								2,640		
Morganella spp.	26.7						5.43	4.05	17.2										
Mycobacterium tuberculosis	380									18.3			335			1.16			25.3
Neisseria gonorrhoeae	15						1.24		13.8										
Proteus spp.	1,060	165	76.6				508		173								139		
Pseudomonas aeruginosa	10,400	390		1,430		3,320	2,190	792	2,280										
Salmonella Paratyphi	19.9								19.8			0.125							
Salmonella Typhi	134								125			9.63							
Non−typhoidal Salmonella	55.4								55.4										
Serratia spp.	871	47		162		196	221	191	52.7										
Shigella spp.	89.5								89.5										
Staphylococcus aureus	20,600								874		3,110			14,800			1,600	255	
Streptococcus pneumoniae	16,600				57.6	8,190	22.1		429		1,770				2,140		3,970		
All pathogens	113,000	4,470	2,520	3,480	3,640	21,600	16,800	1,380	19,500	18.7	6,840	9.71	335	14,800	2,150	1.16	11,700	3,630	25.3

Counts
- <25k
- 100k − <200k

Southeast Asia

DALYs (count) *associated with* bacterial antimicrobial resistance by pathogen−drug combinations, 2019

	Resistance to 1+	AG	Aminopenicillin	Anti−pseudomonal	BL−BLI	CP	3GC	4GC	FQ	Mono INH	Macrolide	MDR in S. Typhi and Paratyphi	MDR excluding XDR in TB	Methicillin	PCN	Mono RIF	TMP−SMX	Vanco	XDR in TB
Acinetobacter baumannii	2,060	1,580		1,970	1,570	1,610	1,830	1,960	1,730										
Citrobacter spp.	142	18		18.6		25.9	88.1	87.3	58										
Enterobacter spp.	747	124		355		206		310	199								472		
Enterococcus faecalis	572								545									71.9	
Enterococcus faecium	1,200								1,190									275	
Other enterococci	491								488									172	
Escherichia coli	3,710	480	3,550		2,780	494	1,750		2,150								2,520		
Group A Streptococcus	202										202								
Group B Streptococcus	426								114		358				37.2				
Haemophilus influenzae	114		104				26.2												
Klebsiella pneumoniae	2,740	351			1,970	516	2,110		1,380								2,030		
Morganella spp.	8.52						5.03	1.85	5.11										
Mycobacterium tuberculosis	27.2									19			6.32			0.746			1.1
Neisseria gonorrhoeae	11.8						0.09		11.8										
Proteus spp.	433	299	350				161		169								244		
Pseudomonas aeruginosa	1,520	449		573		1,070	773	582	671										
Salmonella Paratyphi	28.1								27.5			0.611							
Salmonella Typhi	57.9								52.2			6.23							
Non−typhoidal Salmonella	6.83								6.83										
Serratia spp.	148	22.2		46.2		32.5	102	49.1	46.7										
Shigella spp.	3.34								3.34										
Staphylococcus aureus	4,290								1,230		2,290			3,910			1,080	84.3	
Streptococcus pneumoniae	2,260				382	1,110	497		98		1,360				747		1,660		
All pathogens	21,200	3,320	4,000	2,970	6,710	5,070	7,340	3,000	10,200	19	4,210	6.84	6.32	3,910	785	0.746	8,000	604	1.1

Counts
<25k

Southeast Asia

DALYs (count) *attributable to* bacterial antimicrobial resistance by pathogen−drug combinations, 2019

	Resistance to 1+	AG	Aminopenicillin	Anti−pseudomonal	BL−BLI	CP	3GC	4GC	FQ	Mono INH	Macrolide	MDR in S. Typhi and Paratyphi	MDR excluding XDR in TB	Methicillin	PCN	Mono RIF	TMP−SMX	Vanco	XDR in TB
Acinetobacter baumannii	651	69.4		81.3	0.329	265	10.5	10.1	214										
Citrobacter spp.	39.7	0.951		3.59		6.18	8.44	11.7	8.9										
Enterobacter spp.	166	8.24		39.7		52.9		12.8	21.2								31.2		
Enterococcus faecalis	156								134									21.6	
Enterococcus faecium	298								233									64.9	
Other enterococci	113								83.7									28.9	
Escherichia coli	931	29.1	55.8		137	110	223		228								148		
Group A Streptococcus	19.4										18.2								
Group B Streptococcus	62.1								19.6		32.3				6.82				
Haemophilus influenzae	23.9		16.4				7.48												
Klebsiella pneumoniae	744	35.7			38.8	134	308		108								120		
Morganella spp.	2.17						0.898	0.411	0.866										
Mycobacterium tuberculosis	6.2									2.43			3			0.181			0.593
Neisseria gonorrhoeae	1.16						0.024		1.14										
Proteus spp.	68.8	16.1	5.01				29.8		10								7.73		
Pseudomonas aeruginosa	374	12.7		30.4		204	42.4	13.8	70.8										
Salmonella Paratyphi	5.75								5.66			0.094							
Salmonella Typhi	11.6								10.8			0.81							
Non−typhoidal Salmonella	1.27								1.27										
Serratia spp.	38.9	1.31		9.65		8.94	8.95	4.59	5.44										
Shigella spp.	0.551								0.551										
Staphylococcus aureus	1,310								55		79.5			1,060			89.9	19	
Streptococcus pneumoniae	495				1.36	253	9.9		10.8		55.7				20.2		144		
All pathogens	5,510	173	77.3	165	177	1,030	649	53.4	1,220	2.26	189	0.895	3	1,060	27.1	0.181	541	134	0.593

Counts

<25k

Southern sub−Saharan Africa

DALYs (count) *associated with* bacterial antimicrobial resistance by pathogen−drug combinations, 2019

	Resistance to 1+	AG	Aminopenicillin	Anti−pseudomonal	BL−BLI	CP	3GC	4GC	FQ	Mono INH	Macrolide	MDR in S. Typhi and Paratyphi	MDR excluding XDR in TB	Methicillin	PCN	Mono RIF	TMP−SMX	Vanco	XDR in TB
Acinetobacter baumannii	153,000	71,800		146,000	98,000	103,000	146,000	126,000	128,000										
Citrobacter spp.	8,340	779		2,980		2,520	5,120	3,560	2,830										
Enterobacter spp.	79,900	7,910		14,400		15,600		19,400	14,000								72,100		
Enterococcus faecalis	34,600								33,400									3,000	
Enterococcus faecium	59,400								59,200									6,040	
Other enterococci	30,000								29,900									3,020	
Escherichia coli	335,000	26,500	304,000		179,000	16,400	130,000		124,000								289,000		
Group A Streptococcus	4,380										4,380								
Group B Streptococcus	108,000								16,100		102,000				3,300				
Haemophilus influenzae	29,200		26,900				6,220												
Klebsiella pneumoniae	475,000	99,400			400,000	51,700	415,000		196,000								423,000		
Morganella spp.	283						32.5	62.4	256										
Mycobacterium tuberculosis	236,000									92,200			118,000			24,900			1,560
Neisseria gonorrhoeae	1,260						1.01		1,250										
Proteus spp.	24,700	1,100	22,200				5,300		2,030								18,600		
Pseudomonas aeruginosa	151,000	20,700		79,900		102,000	67,400	64,600	70,200										
Salmonella Paratyphi	33.6								33.1			0.592							
Salmonella Typhi	46,000								15,200			34,000							
Non−typhoidal Salmonella	787								787										
Serratia spp.	21,400	15,700		2,960		2,870	4,950	6,090	4,780										
Shigella spp.	2,980								2,980										
Staphylococcus aureus	360,000								103,000		227,000			166,000			172,000	5,240	
Streptococcus pneumoniae	499,000				108,000	175,000	92,500		37,500		200,000				227,000		416,000		
All pathogens	2,660,000	244,000	353,000	246,000	785,000	469,000	873,000	220,000	840,000	92,200	533,000	34,000	118,000	166,000	231,000	24,900	1,390,000	17,300	1,560

Counts

- <25k
- 25k – <50k
- 50k – <100k
- 100k – <200k
- 200k – <500k
- 500k – <1M
- >=1M

Southern sub−Saharan Africa

DALYs (count) *attributable to* bacterial antimicrobial resistance by pathogen−drug combinations, 2019

	Resistance to 1+	AG	Aminopenicillin	Anti−pseudomonal	BL−BLI	CP	3GC	4GC	FQ	Mono INH	Macrolide	MDR in S. Typhi and Paratyphi	MDR excluding XDR in TB	Methicillin	PCN	Mono RIF	TMP−SMX	Vanco	XDR in TB
Acinetobacter baumannii	47,900	3,100		9,190	5.73	16,900	1,920	48.2	16,800										
Citrobacter spp.	2,430	45.2		559		682	382	310	453										
Enterobacter spp.	14,000	606		1,210		3,890		1,110	1,720								5,340		
Enterococcus faecalis	9,260								8,340									923	
Enterococcus faecium	13,700								12,100									1,520	
Other enterococci	6,250								5,700									549	
Escherichia coli	75,000	1,800	9,160		9,470	3,820	18,400		12,300								20,100		
Group A Streptococcus	418										410								
Group B Streptococcus	13,900								2,610		10,700				694				
Haemophilus influenzae	6,070		4,300				1,780												
Klebsiella pneumoniae	130,000	8,090			4,070	14,000	65,300		14,300								24,100		
Morganella spp.	61.2						3.24	10.4	47.6										
Mycobacterium tuberculosis	87,000									13,200			66,000			6,570			930
Neisseria gonorrhoeae	123						0.739		122										
Proteus spp.	2,870	73.5	488				1,280		190								831		
Pseudomonas aeruginosa	37,100	578		6,760		19,300	1,790	1,070	7,540										
Salmonella Paratyphi	6.83								6.76			0.069							
Salmonella Typhi	7,300								2,900			4,280							
Non−typhoidal Salmonella	150								150										
Serratia spp.	3,660	1,060		492		757	221	658	521										
Shigella spp.	610								610										
Staphylococcus aureus	74,500								4,350		8,970			41,000			18,700	1,480	
Streptococcus pneumoniae	105,000				2,410	38,500	2,320		4,290		7,310				12,800		37,600		
All pathogens	637,000	15,300	13,900	18,200	16,000	97,900	93,400	3,210	95,100	13,400	27,300	4,480	66,000	41,000	13,500	6,570	107,000	4,480	930

Counts
- <25k
- 25k − <50k
- 50k − <100k
- 100k − <200k
- 500k − <1M

Western sub−Saharan Africa

DALYs (count) *associated with* bacterial antimicrobial resistance by pathogen−drug combinations, 2019

	Resistance to 1+	AG	Aminopenicillin	Anti−pseudomonal	BL−BLI	CP	3GC	4GC	FQ	Mono INH	Macrolide	MDR in S. Typhi and Paratyphi	MDR excluding XDR in TB	Methicillin	PCN	Mono RIF	TMP−SMX	Vanco	XDR in TB
Acinetobacter baumannii	959,000	473,000		946,000	455,000	387,000	618,000	540,000	460,000										
Citrobacter spp.	213,000	77,500		90,800		48,100	192,000	175,000	84,200										
Enterobacter spp.	1,150,000	629,000		464,000		196,000		959,000	713,000								938,000		
Enterococcus faecalis	446,000								440,000									27,000	
Enterococcus faecium	392,000								390,000									96,400	
Other enterococci	259,000								259,000									12,000	
Escherichia coli	4,920,000	2,100,000	4,700,000		3,880,000	568,000	3,100,000		3,100,000								4,560,000		
Group A Streptococcus	94,600										94,600								
Group B Streptococcus	2,420,000								1,590,000		1,540,000				40,700				
Haemophilus influenzae	481,000		357,000				223,000												
Klebsiella pneumoniae	6,410,000	4,630,000			6,000,000	866,000	5,460,000		4,870,000								6,100,000		
Morganella spp.	2,250						551	1,310	1,700										
Mycobacterium tuberculosis	585,000									192,000			335,000			53,100			4,460
Neisseria gonorrhoeae	4,530						8.27		4,520										
Proteus spp.	183,000	49,900	160,000				49,300		83,800								143,000		
Pseudomonas aeruginosa	1,910,000	1,080,000		951,000		832,000	1,200,000	930,000	1,070,000										
Salmonella Paratyphi	6,510								5,490			1,210							
Salmonella Typhi	1,630,000								483,000			1,470,000							
Non−typhoidal Salmonella	35,900								35,900										
Serratia spp.	517,000	267,000		74,200		27,500	377,000	443,000	124,000										
Shigella spp.	680,000								680,000										
Staphylococcus aureus	4,080,000								699,000		2,320,000			2,270,000			2,870,000	81,100	
Streptococcus pneumoniae	7,950,000				1,460,000	1,710,000	1,180,000		1,210,000		2,030,000				2,720,000		7,370,000		
All pathogens	35,300,000	9,310,000	5,220,000	2,530,000	11,800,000	4,630,000	12,400,000	3,050,000	16,300,000	192,000	5,990,000	1,470,000	335,000	2,270,000	2,760,000	53,100	22,000,000	217,000	4,460

Counts
- **<25k**
- **25k − <50k**
- **50k − <100k**
- **100k − <200k**
- **200k − <500k**
- **500k − <1M**
- **>=1M**

Western sub−Saharan Africa

DALYs (count) *attributable to* bacterial antimicrobial resistance by pathogen−drug combinations, 2019

	Resistance to 1+	AG	Aminopenicillin	Anti−pseudomonal	BL−BLI	CP	3GC	4GC	FQ	Mono INH	Macrolide	MDR in S. Typhi and Paratyphi	MDR excluding XDR in TB	Methicillin	PCN	Mono RIF	TMP−SMX	Vanco	XDR in TB
Acinetobacter baumannii	279,000	23,900		126,000	10.2	68,200	1,450	23.7	59,300										
Citrobacter spp.	63,800	4,190		14,400		10,600	6,830	17,500	10,400										
Enterobacter spp.	275,000	33,900		35,300		37,600		60,800	66,000								41,300		
Enterococcus faecalis	117,000								110,000									7,380	
Enterococcus faecium	98,100								75,500									22,600	
Other enterococci	52,900								50,500									2,420	
Escherichia coli	1,280,000	119,000	40,200		107,000	107,000	363,000		286,000								256,000		
Group A Streptococcus	8,950										8,680								
Group B Streptococcus	425,000								281,000		138,000				9,260				
Haemophilus influenzae	114,000		49,100				64,400												
Klebsiella pneumoniae	1,770,000	326,000			67,100	185,000	627,000		298,000								269,000		
Morganella spp.	522						25.7	218	278										
Mycobacterium tuberculosis	236,000									28,200			190,000			14,300			2,660
Neisseria gonorrhoeae	441						3.91		437										
Proteus spp.	25,900	2,230	2,900				9,890		5,630								5,320		
Pseudomonas aeruginosa	462,000	29,300		93,500		137,000	59,500	26,400	115,000										
Salmonella Paratyphi	1,280								1,130			142							
Salmonella Typhi	253,000								76,500			179,000							
Non−typhoidal Salmonella	7,390								7,390										
Serratia spp.	131,000	14,900		15,200		7,970	11,400	69,300	12,100										
Shigella spp.	141,000								141,000										
Staphylococcus aureus	951,000								29,100		80,200			529,000			291,000	21,000	
Streptococcus pneumoniae	1,550,000				40,000	370,000	61,100		145,000		72,900				178,000		689,000		
All pathogens	8,250,000	555,000	92,200	284,000	214,000	924,000	1,210,000	174,000	1,770,000	29,200	296,000	174,000	190,000	529,000	187,000	14,300	1,550,000	53,400	2,660

Counts
- **<25k**
- **25k − <50k**
- **50k − <100k**
- **100k − <200k**
- **200k − <500k**
- **500k − <1M**
- **>=1M**

Algeria

DALYs (count) *associated with* bacterial antimicrobial resistance by pathogen−drug combinations, 2019

	Resistance to 1+	AG	Aminopenicillin	Anti−pseudomonal	BL−BLI	CP	3GC	4GC	FQ	Mono INH	Macrolide	MDR in S. Typhi and Paratyphi	MDR excluding XDR in TB	Methicillin	PCN	Mono RIF	TMP−SMX	Vanco	XDR in TB
Acinetobacter baumannii	31,400	27,200		30,900	30,200	27,600	30,300	29,900	28,300										
Citrobacter spp.	2,180	244		728		414	1,480	701	1,100										
Enterobacter spp.	11,700	1,830		6,960		2,710		5,920	2,820								5,020		
Enterococcus faecalis	3,140								2,720									599	
Enterococcus faecium	20,000								19,800									11,200	
Other enterococci	7,970								7,930									3,430	
Escherichia coli	70,700	12,200	68,000		43,200	9,250	23,700		34,200								51,000		
Group A Streptococcus	770										770								
Group B Streptococcus	18,700								2,500		18,100				19.5				
Haemophilus influenzae	2,610		2,410				452												
Klebsiella pneumoniae	74,800	22,200			68,900	9,970	66,200		31,900								51,800		
Morganella spp.	114						41	22.9	98.8										
Mycobacterium tuberculosis	848									141			657			4.67			45.3
Neisseria gonorrhoeae	145						5.59		143										
Proteus spp.	6,200	3,500	5,620				2,720		2,900								4,270		
Pseudomonas aeruginosa	40,400	14,700		16,500		18,900	30,100	20,200	21,000										
Salmonella Paratyphi	98.6								98.3			1.37							
Salmonella Typhi	669								602			54.9							
Non−typhoidal Salmonella	395								395										
Serratia spp.	3,310	798		742		705	2,360	1,650	483										
Shigella spp.	575								575										
Staphylococcus aureus	89,300								18,700		75,500			56,700			17,400	869	
Streptococcus pneumoniae	71,000				14,500	37,700	7,800		4,340		48,500				43,700		51,100		
All pathogens	457,000	82,700	76,100	55,900	157,000	107,000	165,000	58,400	181,000	141	143,000	56.2	657	56,700	43,700	4.67	181,000	16,100	45.3

Counts
- <2.5k
- 2.5k − <5k
- 5k − <10k
- 10k − <25k
- 25k − <75k
- 75k − <150k
- >=150k

Algeria

DALYs (count) *attributable to* bacterial antimicrobial resistance by pathogen−drug combinations, 2019

	Resistance to 1+	AG	Aminopenicillin	Anti−pseudomonal	BL−BLI	CP	3GC	4GC	FQ	Mono INH	Macrolide	MDR in S. Typhi and Paratyphi	MDR excluding XDR in TB	Methicillin	PCN	Mono RIF	TMP−SMX	Vanco	XDR in TB
Acinetobacter baumannii	10,000	1,160		829	16.7	4,500	66.3	12	3,470										
Citrobacter spp.	627	12.6		134		98.9	141	56.2	184										
Enterobacter spp.	2,690	119		918		713		325	303								309		
Enterococcus faecalis	894								675									219	
Enterococcus faecium	5,850								3,280									2,570	
Other enterococci	1,880								1,300									578	
Escherichia coli	16,800	742	2,050		2,480	2,110	2,850		3,450								3,080		
Group A Streptococcus	74.5										71.2								
Group B Streptococcus	2,200								389		1,820				18.4				
Haemophilus influenzae	532		399				132												
Klebsiella pneumoniae	21,000	1,820			1,080	2,490	10,600		2,330								2,640		
Morganella spp.	26.7						5.43	4.05	17.2										
Mycobacterium tuberculosis	380									19.4			335			1.16			25.3
Neisseria gonorrhoeae	15						1.24		13.8										
Proteus spp.	1,060	165	79.1				508		173								139		
Pseudomonas aeruginosa	10,400	406		1,430		3,320	2,190	792	2,280										
Salmonella Paratyphi	19.9								19.8			0.123							
Salmonella Typhi	134								125			9.45							
Non−typhoidal Salmonella	55.4								55.4										
Serratia spp.	871	48.8		162		196	221	191	52.7										
Shigella spp.	89.5								89.5										
Staphylococcus aureus	20,600								874		3,110			14,800			1,600	255	
Streptococcus pneumoniae	16,600				57.6	8,190	22.1		429		1,810				2,140		3,970		
All pathogens	113,000	4,470	2,520	3,480	3,640	21,600	16,800	1,380	19,500	20.2	6,840	9.25	335	14,800	2,150	1.16	11,700	3,630	25.3

Counts
- <2.5k
- 2.5k − <5k
- 5k − <10k
- 10k − <25k
- 75k − <150k

Angola

DALYs (count) *associated with* bacterial antimicrobial resistance by pathogen−drug combinations, 2019

	Resistance to 1+	AG	Aminopenicillin	Anti−pseudomonal	BL−BLI	CP	3GC	4GC	FQ	Mono INH	Macrolide	MDR in S. Typhi and Paratyphi	MDR excluding XDR in TB	Methicillin	PCN	Mono RIF	TMP−SMX	Vanco	XDR in TB
Acinetobacter baumannii	49,800	17,800		48,800	27,100	17,100	37,700	22,500	29,400										
Citrobacter spp.	10,700	2,370		5,360		1,260	8,320	7,450	4,560										
Enterobacter spp.	58,100	25,700		23,000		9,320		47,300	30,200								44,000		
Enterococcus faecalis	21,800								21,500									1,550	
Enterococcus faecium	19,700								19,600									4,900	
Other enterococci	12,100								12,100									810	
Escherichia coli	184,000	58,900	161,000		133,000	22,600	71,700		84,600								164,000		
Group A Streptococcus	6,370										6,370								
Group B Streptococcus	123,000								60,000		95,900				6,490				
Haemophilus influenzae	28,500		26,600				6,560												
Klebsiella pneumoniae	244,000	169,000			220,000	6,420	190,000		169,000								223,000		
Morganella spp.	130						29.8	79.4	94.6										
Mycobacterium tuberculosis	57,400									19,700			35,700			1,550			477
Neisseria gonorrhoeae	308						3.08		307										
Proteus spp.	9,830	2,590	8,780				2,220		2,930								7,760		
Pseudomonas aeruginosa	70,900	23,600		43,600		29,000	50,600	31,100	32,700										
Salmonella Paratyphi	1.82								0.86			0.976							
Salmonella Typhi	31,900								25,300			7,720							
Non−typhoidal Salmonella	270								270										
Serratia spp.	20,500	9,250		4,300		1,590	10,300	16,100	4,370										
Shigella spp.	1,020								1,020										
Staphylococcus aureus	128,000								40,600		59,600			53,100			88,700	1,400	
Streptococcus pneumoniae	340,000				131,000	72,600	38,300		81,900		94,000				106,000		322,000		
All pathogens	1,420,000	310,000	197,000	125,000	512,000	160,000	416,000	125,000	620,000	19,700	256,000	7,720	35,700	53,100	112,000	1,550	849,000	8,660	477

Counts
- <2.5k
- 2.5k − <5k
- 5k − <10k
- 10k − <25k
- 25k − <75k
- 75k − <150k
- >=150k

Angola

DALYs (count) *attributable to* bacterial antimicrobial resistance by pathogen−drug combinations, 2019

	Resistance to 1+	AG	Aminopenicillin	Anti−pseudomonal	BL−BLI	CP	3GC	4GC	FQ	Mono INH	Macrolide	MDR in S. Typhi and Paratyphi	MDR excluding XDR in TB	Methicillin	PCN	Mono RIF	TMP−SMX	Vanco	XDR in TB
Acinetobacter baumannii	14,700	846		6,820	0.845	2,870	194	1	3,940										
Citrobacter spp.	3,090	121		1,000		291	371	686	621										
Enterobacter spp.	13,700	1,410		2,000		1,930		3,360	2,850								2,090		
Enterococcus faecalis	5,770								5,340									425	
Enterococcus faecium	4,950								3,800									1,150	
Other enterococci	2,500								2,340									158	
Escherichia coli	43,400	3,540	2,000		6,060	4,890	8,170		7,950								10,800		
Group A Streptococcus	568										610								
Group B Streptococcus	20,100								10,200		8,840				1,140				
Haemophilus influenzae	5,980		4,130				1,840												
Klebsiella pneumoniae	62,600	11,900			3,810	2,140	24,400		10,200								10,200		
Morganella spp.	30.1						1.22	13.5	15.4										
Mycobacterium tuberculosis	24,000									3,000			20,300			419			285
Neisseria gonorrhoeae	30.8						0.874		29.9										
Proteus spp.	1,280	122	179				474		203								300		
Pseudomonas aeruginosa	18,400	643		5,450		5,230	2,940	530	3,560										
Salmonella Paratyphi	0.311								0.18			0.137							
Salmonella Typhi	6,130								5,160			956							
Non−typhoidal Salmonella	55.1								55.1										
Serratia spp.	4,750	527		845		471	343	2,130	434										
Shigella spp.	213								213										
Staphylococcus aureus	26,200								1,650		2,180			12,100			9,780	436	
Streptococcus pneumoniae	67,400				5,300	15,000	1,720		9,510		3,240				4,340		28,300		
All pathogens	326,000	19,100	6,300	16,100	15,200	32,900	40,500	6,720	68,100	2,950	14,800	981	20,300	12,100	5,480	419	61,500	2,170	285

Counts
- <2.5k
- 2.5k − <5k
- 5k − <10k
- 10k − <25k
- 25k − <75k
- >=150k

Benin

DALYs (count) *associated with* bacterial antimicrobial resistance by pathogen−drug combinations, 2019

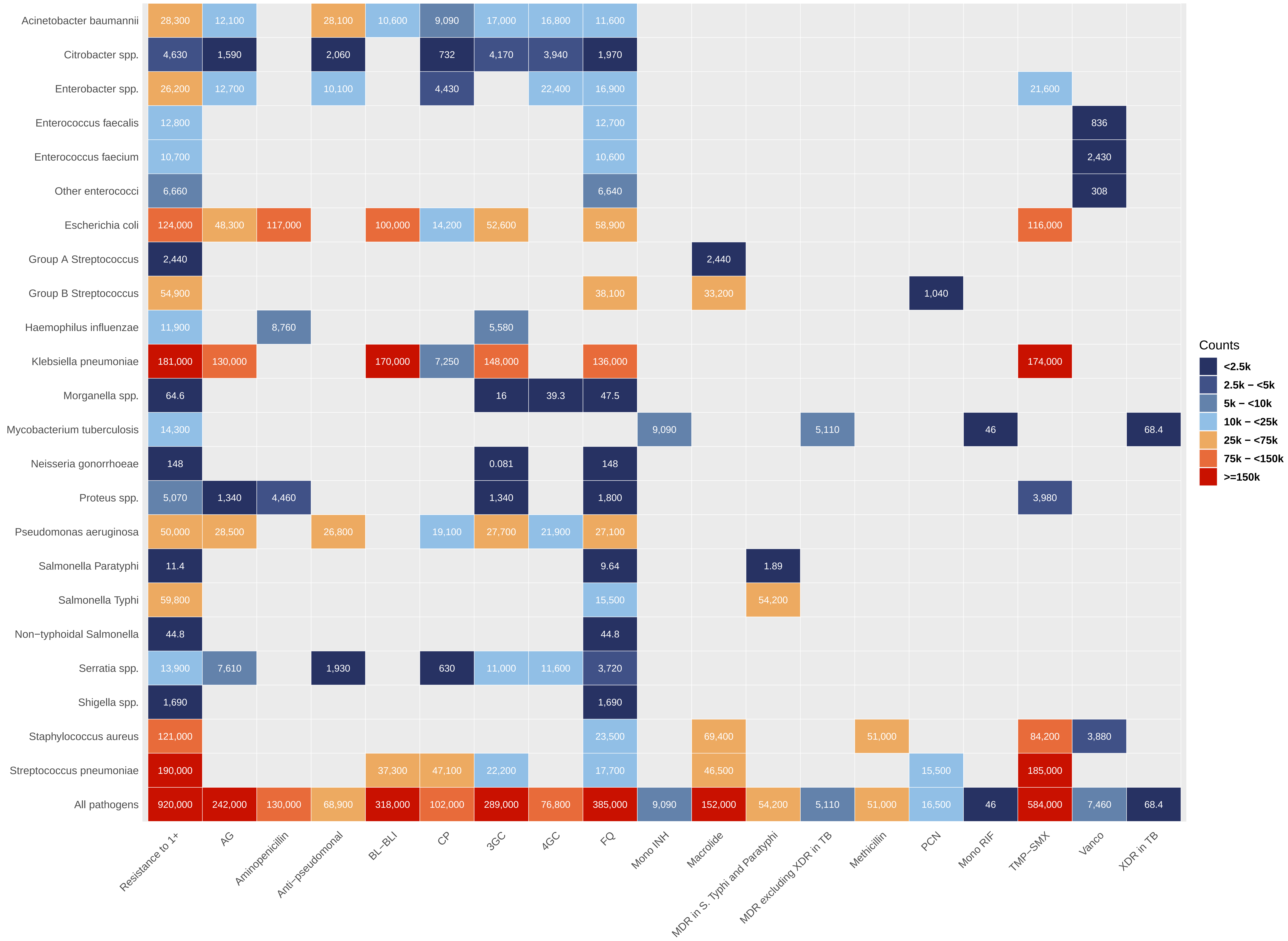

Benin

DALYs (count) *attributable to* bacterial antimicrobial resistance by pathogen−drug combinations, 2019

	Resistance to 1+	AG	Aminopenicillin	Anti−pseudomonal	BL−BLI	CP	3GC	4GC	FQ	Mono INH	Macrolide	MDR in S. Typhi and Paratyphi	MDR excluding XDR in TB	Methicillin	PCN	Mono RIF	TMP−SMX	Vanco	XDR in TB
Acinetobacter baumannii	8,060	633		4,280	0.156	1,630	3.1	0.573	1,520										
Citrobacter spp.	1,380	84.4		353		159	122	415	247										
Enterobacter spp.	6,320	655		757		853		1,510	1,590								990		
Enterococcus faecalis	3,380								3,150									224	
Enterococcus faecium	2,640								2,070									574	
Other enterococci	1,360								1,290									62.8	
Escherichia coli	29,700	2,890	1,210		4,130	2,900	5,930		5,450								7,200		
Group A Streptococcus	240										224								
Group B Streptococcus	9,850								6,770		2,870				220				
Haemophilus influenzae	2,820		1,210				1,610												
Klebsiella pneumoniae	47,700	9,040			2,390	2,010	18,400		8,220								7,650		
Morganella spp.	15.1						0.702	6.64	7.74										
Mycobacterium tuberculosis	4,300									1,250			2,900			12.4			40.7
Neisseria gonorrhoeae	14.3						0.06		14.3										
Proteus spp.	697	61.9	78				277		123								151		
Pseudomonas aeruginosa	11,700	801		3,070		3,150	1,170	511	2,970										
Salmonella Paratyphi	2.26								2.01			0.244							
Salmonella Typhi	8,800								2,440			6,770							
Non−typhoidal Salmonella	9.1								9.1										
Serratia spp.	3,610	427		404		186	397	1,830	360										
Shigella spp.	354								354										
Staphylococcus aureus	25,100								918		2,560			11,600			9,080	985	
Streptococcus pneumoniae	34,400				1,060	10,200	599		2,100		1,680				158		18,500		
All pathogens	203,000	14,600	2,510	8,860	7,590	21,100	28,500	4,270	39,600	1,270	7,370	6,550	2,900	11,600	378	12.4	43,500	1,850	40.7

Counts
- <2.5k
- 2.5k – <5k
- 5k – <10k
- 10k – <25k
- 25k – <75k
- >=150k

Botswana

DALYs (count) *associated with* bacterial antimicrobial resistance by pathogen−drug combinations, 2019

	Resistance to 1+	AG	Aminopenicillin	Anti−pseudomonal	BL−BLI	CP	3GC	4GC	FQ	Mono INH	Macrolide	MDR in S. Typhi and Paratyphi	MDR excluding XDR in TB	Methicillin	PCN	Mono RIF	TMP−SMX	Vanco	XDR in TB
Acinetobacter baumannii	4,140	2,590		4,000	3,000	3,180	3,920	3,310	3,610										
Citrobacter spp.	320	20.2		70.6		107	216	148	114										
Enterobacter spp.	2,390	225		644		442		711	466								2,130		
Enterococcus faecalis	1,030								987									99.5	
Enterococcus faecium	2,190								2,180									490	
Other enterococci	895								893									42	
Escherichia coli	10,800	1,170	10,600		6,070	352	3,370		2,250								9,310		
Group A Streptococcus	120										120								
Group B Streptococcus	2,690								372		2,550				183				
Haemophilus influenzae	646		466				291												
Klebsiella pneumoniae	13,500	3,430			12,000	58.7	10,600		5,600								11,800		
Morganella spp.	6.87						0.986	1.12	6.42										
Mycobacterium tuberculosis	6,540									2,010			3,670			804			48.6
Neisseria gonorrhoeae	25.9						0.061		25.8										
Proteus spp.	765	53.1	642				163		159								630		
Pseudomonas aeruginosa	5,590	1,360		2,850		3,780	2,470	2,090	3,400										
Salmonella Paratyphi	0.922								0.916			0.009							
Salmonella Typhi	834								309			592							
Non−typhoidal Salmonella	5								5										
Serratia spp.	572	365		154		89.3	177	200	119										
Shigella spp.	155								155										
Staphylococcus aureus	9,590								5,060		3,130			1,140			5,720	174	
Streptococcus pneumoniae	15,400				3,570	4,990	2,430		1,090		4,720				7,440		13,200		
All pathogens	78,200	9,210	11,700	7,720	24,700	13,000	23,600	6,460	26,800	2,010	10,500	592	3,670	1,140	7,630	804	42,800	805	48.6

Counts
- <2.5k
- 2.5k − <5k
- 5k − <10k
- 10k − <25k
- 25k − <75k
- 75k − <150k

Botswana

DALYs (count) *attributable to* bacterial antimicrobial resistance by pathogen−drug combinations, 2019

	Resistance to 1+	AG	Aminopenicillin	Anti−pseudomonal	BL−BLI	CP	3GC	4GC	FQ	Mono INH	Macrolide	MDR in S. Typhi and Paratyphi	MDR excluding XDR in TB	Methicillin	PCN	Mono RIF	TMP−SMX	Vanco	XDR in TB
Acinetobacter baumannii	1,310	109		186	0.257	516	37.7	0.379	460										
Citrobacter spp.	94	1.1		12		27.6	21.8	13.9	17.6										
Enterobacter spp.	433	18.2		62.4		107		32.7	56.3								157		
Enterococcus faecalis	278								246									31.8	
Enterococcus faecium	540								426									115	
Other enterococci	182								173									8.47	
Escherichia coli	2,310	78.7	422		358	85.7	505		226								638		
Group A Streptococcus	11.2										12.1								
Group B Streptococcus	363								58.8		259				32.5				
Haemophilus influenzae	152		67				84.6												
Klebsiella pneumoniae	3,400	276			257	37.9	1,780		393								656		
Morganella spp.	1.49						0.108	0.183	1.2										
Mycobacterium tuberculosis	2,580									302			2,030			215			28.7
Neisseria gonorrhoeae	2.53						0.034		2.49										
Proteus spp.	93.5	3.29	13.5				36.8		12.6								27.3		
Pseudomonas aeruginosa	1,350	33.3		186		665	63.4	26.6	374										
Salmonella Paratyphi	0.188								0.187			0.001							
Salmonella Typhi	135								59			78.2							
Non−typhoidal Salmonella	0.908								0.908										
Serratia spp.	110	22.6		27.4		23.6	6.14	17.3	12.6										
Shigella spp.	31.5								31.5										
Staphylococcus aureus	1,440								246		113			353			674	54	
Streptococcus pneumoniae	3,220				89.1	1,080	56.9		125		169				476		1,220		
All pathogens	18,000	542	503	474	704	2,550	2,590	90.9	2,920	303	554	77.8	2,030	353	509	215	3,370	209	28.7

Counts

- <2.5k
- 2.5k − <5k
- 10k − <25k

Burkina Faso

DALYs (count) *associated with* bacterial antimicrobial resistance by pathogen−drug combinations, 2019

	Resistance to 1+	AG	Aminopenicillin	Anti−pseudomonal	BL−BLI	CP	3GC	4GC	FQ	Mono INH	Macrolide	MDR in S. Typhi and Paratyphi	MDR excluding XDR in TB	Methicillin	PCN	Mono RIF	TMP−SMX	Vanco	XDR in TB
Acinetobacter baumannii	61,400	23,600		60,600	27,200	15,500	37,600	41,000	26,100										
Citrobacter spp.	9,490	2,430		4,000		1,260	8,270	7,810	3,380										
Enterobacter spp.	50,000	18,900		17,700		8,860		41,400	33,900								39,100		
Enterococcus faecalis	29,200								28,900									1,890	
Enterococcus faecium	23,700								23,600									5,050	
Other enterococci	13,500								13,500									787	
Escherichia coli	286,000	153,000	274,000		225,000	31,200	128,000		147,000								254,000		
Group A Streptococcus	5,960										5,960								
Group B Streptococcus	98,900								69,100		58,000				1,030				
Haemophilus influenzae	35,800		28,300				15,700												
Klebsiella pneumoniae	439,000	284,000			395,000	15,500	375,000		322,000								418,000		
Morganella spp.	209						49.3	129	153										
Mycobacterium tuberculosis	31,900									8,160			23,100			311			308
Neisseria gonorrhoeae	247						0.483		246										
Proteus spp.	11,000	1,280	9,760				2,630		4,100								8,000		
Pseudomonas aeruginosa	98,900	41,100		60,100		33,600	53,200	45,200	55,900										
Salmonella Paratyphi	1,240								1,020			230							
Salmonella Typhi	105,000								36,900			72,800							
Non−typhoidal Salmonella	3,050								3,050										
Serratia spp.	28,400	13,000		3,250		4,780	21,000	24,700	7,340										
Shigella spp.	2,830								2,830										
Staphylococcus aureus	309,000								52,900		212,000			118,000			183,000	12,600	
Streptococcus pneumoniae	440,000				151,000	94,700	51,000		43,600		133,000				37,600		418,000		
All pathogens	2,090,000	537,000	312,000	146,000	798,000	205,000	693,000	160,000	876,000	8,160	409,000	73,100	23,100	118,000	38,600	311	1,320,000	20,400	308

Counts
- <2.5k
- 2.5k − <5k
- 5k − <10k
- 10k − <25k
- 25k − <75k
- 75k − <150k
- >=150k

Burkina Faso

DALYs (count) *attributable to* bacterial antimicrobial resistance by pathogen−drug combinations, 2019

	Resistance to 1+	AG	Aminopenicillin	Anti−pseudomonal	BL−BLI	CP	3GC	4GC	FQ	Mono INH	Macrolide	MDR in S. Typhi and Paratyphi	MDR excluding XDR in TB	Methicillin	PCN	Mono RIF	TMP−SMX	Vanco	XDR in TB
Acinetobacter baumannii	17,600	1,270		9,760	0.617	2,920	73.2	3.51	3,570										
Citrobacter spp.	2,790	134		741		292	303	896	428										
Enterobacter spp.	12,100	943		1,310		1,720		2,900	3,440								1,850		
Enterococcus faecalis	7,690								7,190									499	
Enterococcus faecium	5,840								4,630									1,210	
Other enterococci	2,790								2,640									157	
Escherichia coli	69,600	9,320	3,290		8,980	6,140	14,200		13,200								14,500		
Group A Streptococcus	582										563								
Group B Streptococcus	17,700								12,400		4,950				273				
Haemophilus influenzae	8,320		3,850				4,470												
Klebsiella pneumoniae	117,000	19,800			4,070	4,510	49,100		19,900								19,300		
Morganella spp.	48.8						1.96	21.8	25										
Mycobacterium tuberculosis	14,700									1,240			13,200			85.1			185
Neisseria gonorrhoeae	24						0.229		23.7										
Proteus spp.	1,440	67	199				575		286								317		
Pseudomonas aeruginosa	23,900	1,150		7,990		5,570	1,840	994	6,350										
Salmonella Paratyphi	244								213			31							
Salmonella Typhi	16,700								7,270			9,190							
Non−typhoidal Salmonella	633								633										
Serratia spp.	7,440	686		600		1,200	630	3,600	703										
Shigella spp.	584								584										
Staphylococcus aureus	60,800								2,230		8,300			27,400			19,400	3,380	
Streptococcus pneumoniae	80,100				5,820	20,600	2,340		5,260		4,780				556		40,400		
All pathogens	468,000	33,400	7,340	20,400	19,100	43,000	73,600	8,420	90,900	1,230	18,800	9,280	13,200	27,400	829	85.1	95,700	5,240	185

Counts
- <2.5k
- 2.5k − <5k
- 5k − <10k
- 10k − <25k
- 25k − <75k
- 75k − <150k
- >=150k

Burundi

DALYs (count) *associated with* bacterial antimicrobial resistance by pathogen−drug combinations, 2019

	Resistance to 1+	AG	Aminopenicillin	Anti−pseudomonal	BL−BLI	CP	3GC	4GC	FQ	Mono INH	Macrolide	MDR in S. Typhi and Paratyphi	MDR excluding XDR in TB	Methicillin	PCN	Mono RIF	TMP−SMX	Vanco	XDR in TB
Acinetobacter baumannii	24,900	6,640		22,400	16,700	8,130	22,400	15,600	9,070										
Citrobacter spp.	2,830	623		1,290		174	2,380	1,880	1,680										
Enterobacter spp.	15,600	7,850		6,220		2,020		14,300	8,760								11,200		
Enterococcus faecalis	11,000								10,900									642	
Enterococcus faecium	8,630								8,540									2,380	
Other enterococci	3,400								3,380									230	
Escherichia coli	104,000	23,500	98,100		73,200	7,970	37,100		39,900								86,100		
Group A Streptococcus	4,440										4,440								
Group B Streptococcus	32,700								12,200		28,700				1,100				
Haemophilus influenzae	15,400		15,300				2,040												
Klebsiella pneumoniae	122,000	95,700			114,000	1,480	86,900		59,800								109,000		
Morganella spp.	63.5						13.9	41.5	44.8										
Mycobacterium tuberculosis	30,600									3,400			25,800			1,090			346
Neisseria gonorrhoeae	57.4						1.19		56.8										
Proteus spp.	4,750	1,670	3,970				888		2,820								4,170		
Pseudomonas aeruginosa	29,000	8,870		14,400		7,500	23,800	11,300	10,100										
Salmonella Paratyphi	1.34								0.261			1.07							
Salmonella Typhi	47,700								19,700			34,400							
Non−typhoidal Salmonella	55.5								55.5										
Serratia spp.	9,830	4,450		3,170		439	6,840	8,280	2,350										
Shigella spp.	13,500								13,500										
Staphylococcus aureus	74,800								24,900		28,800			30,300			52,800	969	
Streptococcus pneumoniae	121,000				22,200	26,900	14,400		64,800		10,300				15,300		111,000		
All pathogens	676,000	149,000	117,000	47,600	226,000	54,700	197,000	51,400	293,000	3,400	72,200	34,400	25,800	30,300	16,400	1,090	375,000	4,220	346

Counts
- <2.5k
- 2.5k − <5k
- 5k − <10k
- 10k − <25k
- 25k − <75k
- 75k − <150k
- >=150k

Burundi

DALYs (count) *attributable to* bacterial antimicrobial resistance by pathogen−drug combinations, 2019

	Resistance to 1+	AG	Aminopenicillin	Anti−pseudomonal	BL−BLI	CP	3GC	4GC	FQ	Mono INH	Macrolide	MDR in S. Typhi and Paratyphi	MDR excluding XDR in TB	Methicillin	PCN	Mono RIF	TMP−SMX	Vanco	XDR in TB
Acinetobacter baumannii	7,470	360		3,540	40.3	1,590	693	3.11	1,240										
Citrobacter spp.	824	28.6		232		39.8	115	173	236										
Enterobacter spp.	3,770	416		551		411		1,120	787								476		
Enterococcus faecalis	2,890								2,720									171	
Enterococcus faecium	2,200								1,640									562	
Other enterococci	704								658									45.9	
Escherichia coli	23,600	1,450	2,060		4,080	1,790	4,800		3,840								5,600		
Group A Streptococcus	417										408								
Group B Streptococcus	4,870								1,960		2,760				208				
Haemophilus influenzae	3,030		2,470				563												
Klebsiella pneumoniae	30,500	7,120			2,790	643	11,400		3,600								4,940		
Morganella spp.	14.8						0.445	7.08	7.23										
Mycobacterium tuberculosis	15,800									502			14,800			295			207
Neisseria gonorrhoeae	5.83						0.325		5.51										
Proteus spp.	649	72	69.1				171		187								153		
Pseudomonas aeruginosa	7,620	265		2,210		1,380	2,260	365	1,130										
Salmonella Paratyphi	0.203								0.061			0.148							
Salmonella Typhi	7,840								3,640			4,040							
Non−typhoidal Salmonella	11.1								11.1										
Serratia spp.	2,480	254		637		138	187	1,030	232										
Shigella spp.	2,730								2,730										
Staphylococcus aureus	15,300								1,050		1,050			6,990			5,970	279	
Streptococcus pneumoniae	24,700				553	5,150	433		8,250		367				291		9,660		
All pathogens	157,000	9,970	4,600	7,160	7,460	11,100	20,700	2,700	33,900	491	4,540	4,170	14,800	6,990	499	295	26,800	1,060	207

Counts
- <2.5k
- 2.5k − <5k
- 5k − <10k
- 10k − <25k
- 25k − <75k
- >=150k

Cameroon

DALYs (count) *associated with* bacterial antimicrobial resistance by pathogen−drug combinations, 2019

	Resistance to 1+	AG	Aminopenicillin	Anti−pseudomonal	BL−BLI	CP	3GC	4GC	FQ	Mono INH	Macrolide	MDR in S. Typhi and Paratyphi	MDR excluding XDR in TB	Methicillin	PCN	Mono RIF	TMP−SMX	Vanco	XDR in TB
Acinetobacter baumannii	49,600	24,400		48,100	21,800	27,800	38,200	29,300	27,400										
Citrobacter spp.	7,550	2,330		3,090		857	6,660	5,080	3,140										
Enterobacter spp.	43,800	21,900		17,900		6,510		34,700	26,900								34,000		
Enterococcus faecalis	19,600								19,200									1,330	
Enterococcus faecium	20,500								20,400									5,290	
Other enterococci	12,700								12,700									711	
Escherichia coli	204,000	79,100	192,000		159,000	18,700	103,000		96,200								181,000		
Group A Streptococcus	5,180										5,180								
Group B Streptococcus	71,000								40,100		49,600				1,170				
Haemophilus influenzae	15,500		12,300				5,890												
Klebsiella pneumoniae	265,000	178,000			247,000	6,480	137,000		205,000								252,000		
Morganella spp.	148						37.6	83.6	112										
Mycobacterium tuberculosis	40,400									7,910			14,600			17,700			196
Neisseria gonorrhoeae	424						2.35		423										
Proteus spp.	9,430	1,760	8,480				2,150		3,370								6,980		
Pseudomonas aeruginosa	87,200	39,200		44,100		39,300	59,200	32,700	48,800										
Salmonella Paratyphi	85.6								83.8			2.07							
Salmonella Typhi	87,900								23,700			78,000							
Non−typhoidal Salmonella	237								237										
Serratia spp.	22,100	8,140		3,780		1,560	18,700	16,500	5,800										
Shigella spp.	13,600								13,600										
Staphylococcus aureus	187,000								39,800		89,800			98,600			128,000	1,520	
Streptococcus pneumoniae	288,000				60,800	59,400	35,100		29,900		63,300				55,800		278,000		
All pathogens	1,450,000	355,000	213,000	117,000	488,000	161,000	406,000	118,000	617,000	7,910	208,000	78,000	14,600	98,600	56,900	17,700	880,000	8,850	196

Counts
- <2.5k
- 2.5k − <5k
- 5k − <10k
- 10k − <25k
- 25k − <75k
- 75k − <150k
- >=150k

Cameroon

DALYs (count) *attributable to* bacterial antimicrobial resistance by pathogen−drug combinations, 2019

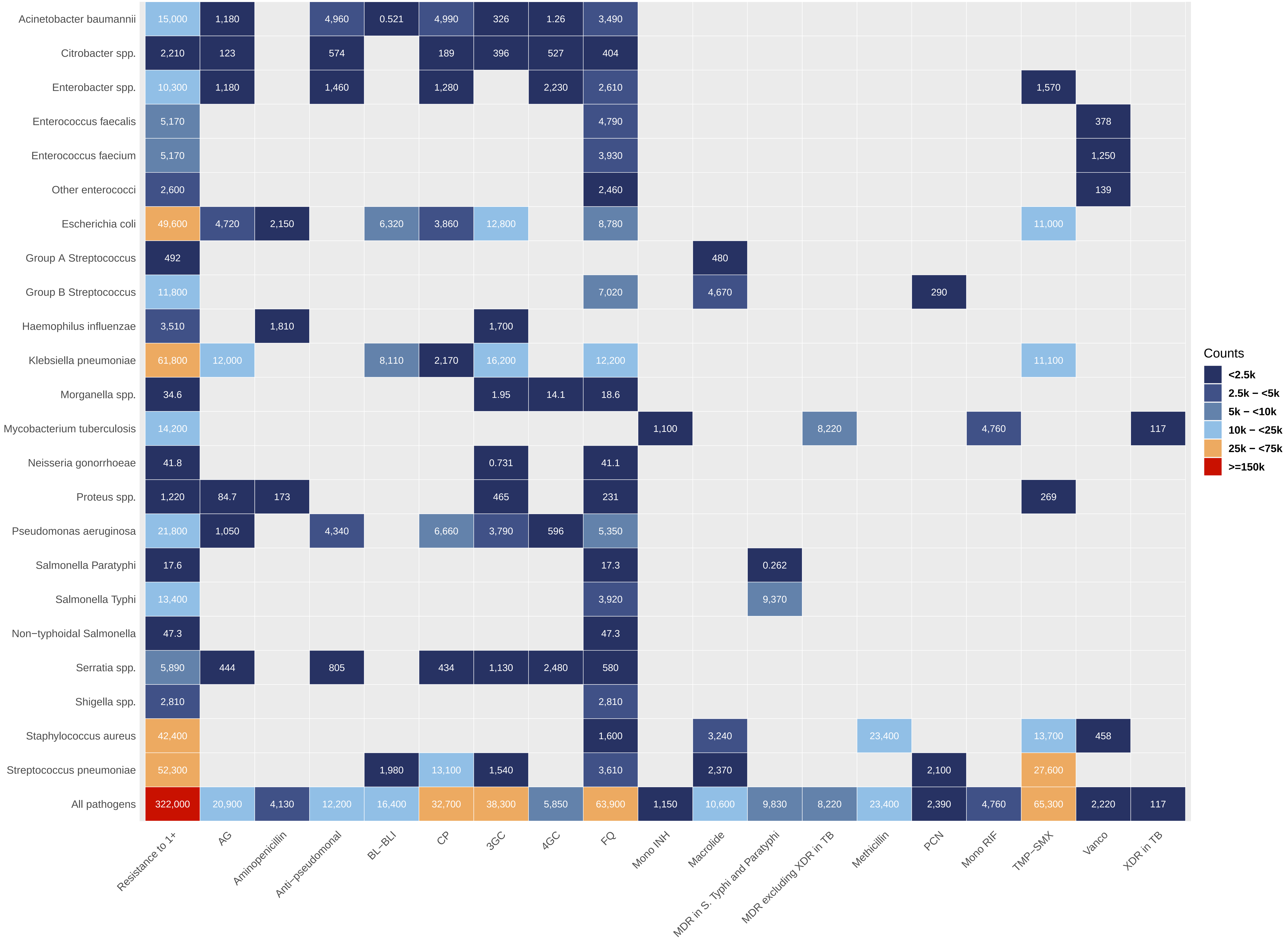

Cape Verde

DALYs (count) *associated with* bacterial antimicrobial resistance by pathogen−drug combinations, 2019

	Resistance to 1+	AG	Aminopenicillin	Anti−pseudomonal	BL−BLI	CP	3GC	4GC	FQ	Mono INH	Macrolide	MDR in S. Typhi and Paratyphi	MDR excluding XDR in TB	Methicillin	PCN	Mono RIF	TMP−SMX	Vanco	XDR in TB
Acinetobacter baumannii	1,180	887		1,160	759	914	1,030	991	1,020										
Citrobacter spp.	115	11.8		20.8		14.7	93.7	56.8	46.8										
Enterobacter spp.	715	201		289		94.2		404	273								588		
Enterococcus faecalis	145								134									23.7	
Enterococcus faecium	290								289									90.7	
Other enterococci	142								141									10.5	
Escherichia coli	1,140	325	1,080		897	42.6	568		517								1,010		
Group A Streptococcus	41.2										41.2								
Group B Streptococcus	225								55.3		203				4.12				
Haemophilus influenzae	44.9		32				19.9												
Klebsiella pneumoniae	1,230	681			1,140	36.5	986		732								1,110		
Morganella spp.	1.08						0.218	0.149	1.01										
Mycobacterium tuberculosis	176									34.1			139			1.21			1.82
Neisseria gonorrhoeae	5.98						0.012		5.97										
Proteus spp.	115	45.7	99.1				28.1		59.6								90.8		
Pseudomonas aeruginosa	665	323		291		367	360	237	449										
Salmonella Paratyphi	0.575								0.556			0.021							
Salmonella Typhi	353								68.9			303							
Non−typhoidal Salmonella	0.278								0.278										
Serratia spp.	137	33.3		28.2		14.4	116	67.1	42.4										
Shigella spp.	5.8								5.8										
Staphylococcus aureus	1,150								205		781			716			489	16.6	
Streptococcus pneumoniae	1,500				340	576	197		77.8		398				274		1,410		
All pathogens	9,370	2,510	1,210	1,790	3,130	2,060	3,400	1,760	4,120	34.1	1,420	303	139	716	278	1.21	4,690	142	1.82

Counts
- **<2.5k**
- **2.5k − <5k**
- **5k − <10k**

Cape Verde

DALYs (count) *attributable to* bacterial antimicrobial resistance by pathogen−drug combinations, 2019

	Resistance to 1+	AG	Aminopenicillin	Anti−pseudomonal	BL−BLI	CP	3GC	4GC	FQ	Mono INH	Macrolide	MDR in S. Typhi and Paratyphi	MDR excluding XDR in TB	Methicillin	PCN	Mono RIF	TMP−SMX	Vanco	XDR in TB
Acinetobacter baumannii	371	38.1		54.7	0.019	147	3.99	0.108	127										
Citrobacter spp.	33	0.589		4.39		3.33	10.6	7.36	6.74										
Enterobacter spp.	150	12.2		30.1		20.1		24.7	27.6								35.1		
Enterococcus faecalis	40.3								32.7									7.57	
Enterococcus faecium	75.2								54.2									21	
Other enterococci	29.2								27.2									2.01	
Escherichia coli	268	19.3	12.9		38.8	9.33	76.5		48.2								63.1		
Group A Streptococcus	3.92										3.9								
Group B Streptococcus	30.4								9.05		21.2				1.06				
Haemophilus influenzae	10.4		4.67				5.78												
Klebsiella pneumoniae	319	48.9			21.3	11.4	137		46.6								53		
Morganella spp.	0.237						0.026	0.025	0.186										
Mycobacterium tuberculosis	80.4									4.63			74.2			0.311			1.05
Neisseria gonorrhoeae	0.585						0.006		0.58										
Proteus spp.	16.2	1.99	1.71				5.49		3.82								3.19		
Pseudomonas aeruginosa	159	8.47		19.3		59.4	18.2	4.18	49.6										
Salmonella Paratyphi	0.118								0.115			0.003							
Salmonella Typhi	51.4								12.9			39.5							
Non−typhoidal Salmonella	0.048								0.048										
Serratia spp.	36.5	1.9		6.19		3.71	11.9	8.41	4.44										
Shigella spp.	1.06								1.06										
Staphylococcus aureus	274								9.29		30.4			182			48.3	4.42	
Streptococcus pneumoniae	299				6.7	127	1.64		8.85		14.5				4.15		136		
All pathogens	2,250	131	19.3	115	66.8	381	272	44.8	470	4.99	69.1	40.3	74.2	182	5.21	0.311	339	35	1.05

Counts
<2.5k

Central African Republic

DALYs (count) *associated with* bacterial antimicrobial resistance by pathogen−drug combinations, 2019

	Resistance to 1+	AG	Aminopenicillin	Anti−pseudomonal	BL−BLI	CP	3GC	4GC	FQ	Mono INH	Macrolide	MDR in S. Typhi and Paratyphi	MDR excluding XDR in TB	Methicillin	PCN	Mono RIF	TMP−SMX	Vanco	XDR in TB
Acinetobacter baumannii	16,300	4,190		15,300	10,600	4,150	11,700	11,100	7,070										
Citrobacter spp.	2,020	482		1,120		264	1,510	1,380	683										
Enterobacter spp.	14,300	5,120		4,580		3,260		12,500	7,020								9,810		
Enterococcus faecalis	6,650								6,600									452	
Enterococcus faecium	4,170								4,140									1,210	
Other enterococci	1,720								1,710									204	
Escherichia coli	74,500	39,500	62,900		43,000	10,000	25,700		38,900								58,500		
Group A Streptococcus	1,240										1,240								
Group B Streptococcus	60,400								41,700		38,400				1,740				
Haemophilus influenzae	20,300		19,400				7,700												
Klebsiella pneumoniae	94,500	81,700			73,000	3,350	70,100		79,400								80,100		
Morganella spp.	53.4						12.8	34.1	39.7										
Mycobacterium tuberculosis	55,400									39,100			14,200			1,830			190
Neisseria gonorrhoeae	95.8						32.4		92.7										
Proteus spp.	2,460	448	2,300				642		666								1,600		
Pseudomonas aeruginosa	19,500	6,500		12,700		7,500	11,900	13,500	6,570										
Salmonella Paratyphi	0.934								0.757			0.189							
Salmonella Typhi	29,000								22,700			9,000							
Non−typhoidal Salmonella	101								101										
Serratia spp.	6,090	2,110		889		1,630	3,430	5,430	1,290										
Shigella spp.	144								144										
Staphylococcus aureus	60,400								45,100		38,900			16,000			45,000	822	
Streptococcus pneumoniae	162,000				110,000	24,500	16,000		140,000		113,000				110,000		134,000		
All pathogens	631,000	140,000	84,600	34,600	237,000	54,700	149,000	44,000	404,000	39,100	191,000	9,000	14,200	16,000	112,000	1,830	329,000	2,680	190

Counts
- **<2.5k**
- **2.5k − <5k**
- **5k − <10k**
- **10k − <25k**
- **25k − <75k**
- **75k − <150k**
- **>=150k**

Central African Republic

DALYs (count) *attributable to* bacterial antimicrobial resistance by pathogen−drug combinations, 2019

	Resistance to 1+	AG	Aminopenicillin	Anti−pseudomonal	BL−BLI	CP	3GC	4GC	FQ	Mono INH	Macrolide	MDR in S. Typhi and Paratyphi	MDR excluding XDR in TB	Methicillin	PCN	Mono RIF	TMP−SMX	Vanco	XDR in TB
Acinetobacter baumannii	4,750	225		2,490	25.8	816	196	7.6	996										
Citrobacter spp.	580	26.5		220		62.8	59.2	120	90.6										
Enterobacter spp.	3,490	271		364		694		1,040	660								473		
Enterococcus faecalis	1,750								1,640									112	
Enterococcus faecium	1,070								788									282	
Other enterococci	366								328									37.9	
Escherichia coli	17,800	2,670	1,280		1,940	2,120	2,660		3,700								3,440		
Group A Streptococcus	121										117								
Group B Streptococcus	10,900								7,290		3,200				314				
Haemophilus influenzae	4,530		2,440				2,090												
Klebsiella pneumoniae	24,500	6,030			845	1,050	8,290		4,940								3,360		
Morganella spp.	12.5						0.45	5.66	6.41										
Mycobacterium tuberculosis	15,200									6,320			8,310			507			115
Neisseria gonorrhoeae	13.7						5.78		7.94										
Proteus spp.	321	22	48.7				143		46.1								62.4		
Pseudomonas aeruginosa	4,970	187		1,770		1,430	325	566	689										
Salmonella Paratyphi	0.181								0.157			0.024							
Salmonella Typhi	5,570								4,530			1,080							
Non−typhoidal Salmonella	21.1								21.1										
Serratia spp.	1,550	107		141		416	82.5	678	124										
Shigella spp.	30.5								30.5										
Staphylococcus aureus	11,100								1,730		1,160			3,840			4,100	239	
Streptococcus pneumoniae	38,600				4,800	4,630	1,240		15,100		3,160				2,290		7,560		
All pathogens	147,000	9,550	3,770	4,990	7,610	11,200	15,100	2,420	42,700	5,890	7,570	1,010	8,310	3,840	2,610	507	19,000	671	115

Counts
- <2.5k
- 2.5k − <5k
- 5k − <10k
- 10k − <25k
- 25k − <75k
- 75k − <150k

Chad

DALYs (count) *associated with* bacterial antimicrobial resistance by pathogen−drug combinations, 2019

	Resistance to 1+	AG	Aminopenicillin	Anti−pseudomonal	BL−BLI	CP	3GC	4GC	FQ	Mono INH	Macrolide	MDR in S. Typhi and Paratyphi	MDR excluding XDR in TB	Methicillin	PCN	Mono RIF	TMP−SMX	Vanco	XDR in TB
Acinetobacter baumannii	61,200	24,900		60,800	32,400	21,100	36,400	26,100	20,300										
Citrobacter spp.	17,500	7,090		7,460		6,840	15,500	15,300	6,440										
Enterobacter spp.	75,000	32,100		27,700		16,400		67,100	46,800								57,400		
Enterococcus faecalis	16,500								16,100									1,260	
Enterococcus faecium	13,000								12,900									3,370	
Other enterococci	10,900								10,800									624	
Escherichia coli	246,000	71,400	241,000		193,000	27,500	136,000		163,000								211,000		
Group A Streptococcus	3,930										3,930								
Group B Streptococcus	173,000								114,000		114,000				5,650				
Haemophilus influenzae	45,800		38,000				19,500												
Klebsiella pneumoniae	297,000	219,000			280,000	5,550	279,000		195,000								267,000		
Morganella spp.	95.5						23.7	61.1	69.1										
Mycobacterium tuberculosis	34,100									12,600			21,000			292			281
Neisseria gonorrhoeae	148						0.307		147										
Proteus spp.	7,400	2,690	6,380				1,950		4,320								5,850		
Pseudomonas aeruginosa	66,500	31,100		37,700		20,600	42,800	38,200	30,900										
Salmonella Paratyphi	23.6								21.7			2.08							
Salmonella Typhi	29,900								10,900			19,900							
Non−typhoidal Salmonella	301								301										
Serratia spp.	31,700	17,200		4,420		1,480	27,600	26,600	8,600										
Shigella spp.	13,300								13,300										
Staphylococcus aureus	133,000								16,200		81,700			52,000			92,600	4,190	
Streptococcus pneumoniae	571,000				106,000	121,000	57,200		191,000		158,000				73,200		533,000		
All pathogens	1,850,000	406,000	285,000	138,000	612,000	220,000	616,000	173,000	860,000	12,600	358,000	20,000	21,000	52,000	78,800	292	1,170,000	9,440	281

Counts
- <2.5k
- 2.5k − <5k
- 5k − <10k
- 10k − <25k
- 25k − <75k
- 75k − <150k
- >=150k

Chad

DALYs (count) *attributable to* bacterial antimicrobial resistance by pathogen−drug combinations, 2019

	Resistance to 1+	AG	Aminopenicillin	Anti−pseudomonal	BL−BLI	CP	3GC	4GC	FQ	Mono INH	Macrolide	MDR in S. Typhi and Paratyphi	MDR excluding XDR in TB	Methicillin	PCN	Mono RIF	TMP−SMX	Vanco	XDR in TB
Acinetobacter baumannii	17,300	1,330		9,470	0.419	3,930	2.96	1.14	2,610										
Citrobacter spp.	5,330	380		956		1,530	388	1,320	764										
Enterobacter spp.	18,600	1,630		2,040		3,200		4,820	4,390								2,540		
Enterococcus faecalis	4,370								4,020									349	
Enterococcus faecium	3,270								2,480									790	
Other enterococci	2,240								2,110									126	
Escherichia coli	63,000	4,000	2,930		6,830	5,360	16,100		15,900								11,800		
Group A Streptococcus	373										388								
Group B Streptococcus	30,600								19,900		9,730				1,010				
Haemophilus influenzae	10,600		5,060				5,540												
Klebsiella pneumoniae	81,800	15,600			1,740	1,920	39,300		11,700								11,500		
Morganella spp.	22.3						0.905	10.3	11.1										
Mycobacterium tuberculosis	14,300									2,030			12,200			79.6			169
Neisseria gonorrhoeae	14.4						0.145		14.3										
Proteus spp.	1,080	114	110				372		286								201		
Pseudomonas aeruginosa	16,500	879		5,210		3,520	2,010	1,480	3,330										
Salmonella Paratyphi	4.81								4.54			0.267							
Salmonella Typhi	4,850								2,230			2,640							
Non−typhoidal Salmonella	62.6								62.6										
Serratia spp.	8,460	962		907		420	1,030	4,280	837										
Shigella spp.	2,780								2,780										
Staphylococcus aureus	26,700								700		3,070			11,800			10,000	1,080	
Streptococcus pneumoniae	109,000				3,080	24,400	1,630		24,100		5,360				1,790		48,900		
All pathogens	422,000	24,900	8,100	18,600	11,700	44,300	66,400	11,900	98,200	1,810	18,700	2,730	12,200	11,800	2,800	79.6	85,000	2,340	169

Counts
- <2.5k
- 2.5k − <5k
- 5k − <10k
- 10k − <25k
- 25k − <75k
- 75k − <150k
- >=150k

Comoros

DALYs (count) *associated with* bacterial antimicrobial resistance by pathogen−drug combinations, 2019

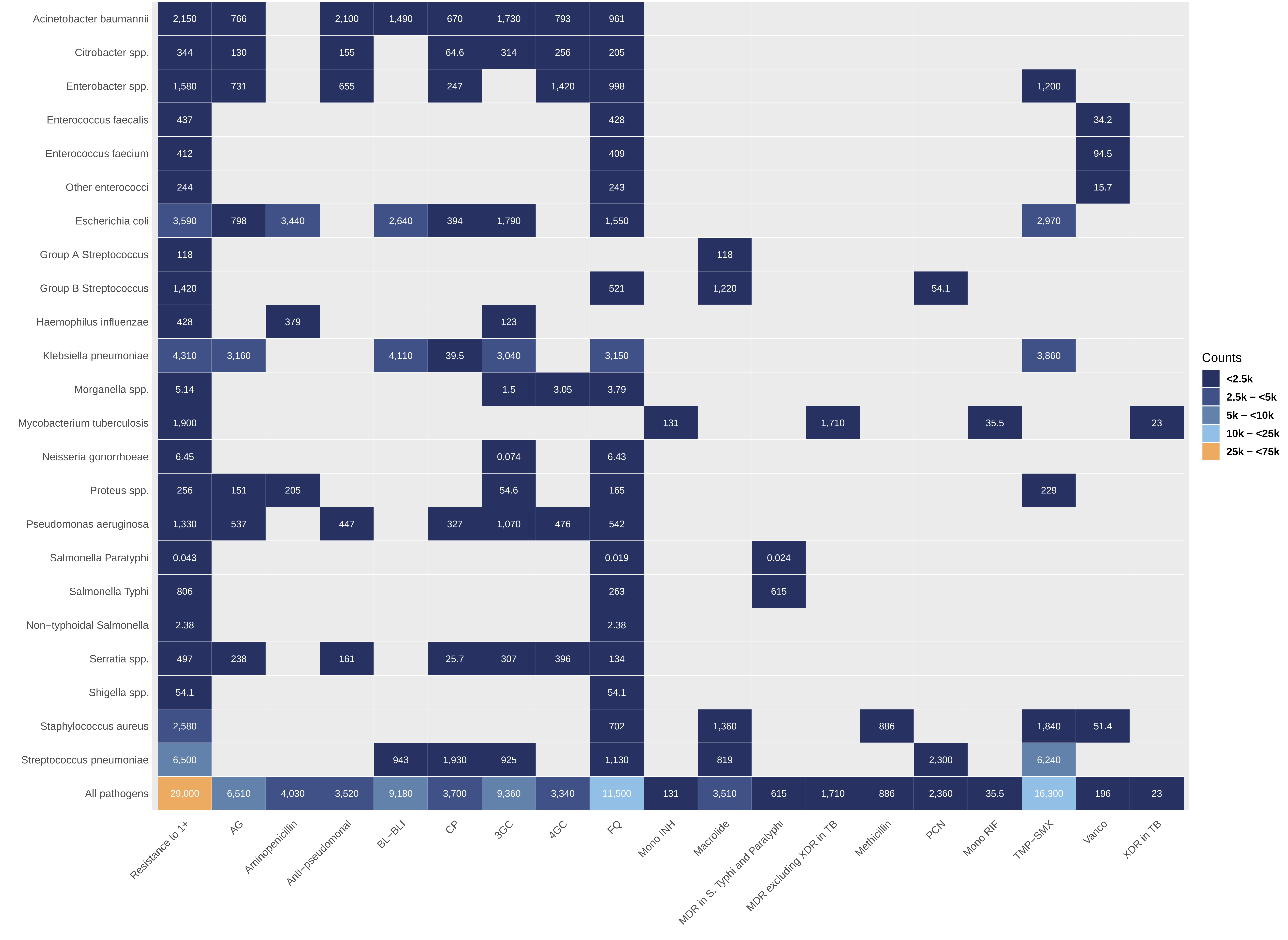

Comoros

DALYs (count) *attributable to* bacterial antimicrobial resistance by pathogen−drug combinations, 2019

	Resistance to 1+	AG	Aminopenicillin	Anti−pseudomonal	BL−BLI	CP	3GC	4GC	FQ	Mono INH	Macrolide	MDR in S. Typhi and Paratyphi	MDR excluding XDR in TB	Methicillin	PCN	Mono RIF	TMP−SMX	Vanco	XDR in TB
Acinetobacter baumannii	632	39.3		338	0.429	117	11	0.048	127										
Citrobacter spp.	103	6.02		23.4		12.5	13	21.4	26.7										
Enterobacter spp.	384	37.3		53.7		48.5		99.9	92.7								52		
Enterococcus faecalis	116								106									9.42	
Enterococcus faecium	103								80									22.7	
Other enterococci	50.6								47.4									3.14	
Escherichia coli	876	48.1	55.1		117	85.7	236		148								185		
Group A Streptococcus	10.9										11.2								
Group B Streptococcus	212								85.8		116				10.1				
Haemophilus influenzae	92.1		57.1				35												
Klebsiella pneumoniae	1,080	219			102	19	383		188								164		
Morganella spp.	1.21						0.079	0.52	0.614										
Mycobacterium tuberculosis	989									19.2			946			9.51			13.6
Neisseria gonorrhoeae	0.642						0.02		0.621										
Proteus spp.	37.4	6.57	3.26				9.7		10.3								7.7		
Pseudomonas aeruginosa	337	15.9		60.3		56.8	119	23.5	61.6										
Salmonella Paratyphi	0.007								0.004			0.003							
Salmonella Typhi	125								49.2			76.9							
Non−typhoidal Salmonella	0.477								0.477										
Serratia spp.	121	13.6		31.4		7.91	8.67	46.7	13.2										
Shigella spp.	10.9								10.9										
Staphylococcus aureus	490								27.8		50.3			195			203	13.5	
Streptococcus pneumoniae	1,310				16.4	411	19.4		130		29.5				108		590		
All pathogens	7,070	386	115	506	236	758	836	192	1,210	19.5	208	76	946	195	118	9.51	1,200	48.8	13.6

Counts
- **<2.5k**
- **5k − <10k**

Congo (Brazzaville)

DALYs (count) *associated with* bacterial antimicrobial resistance by pathogen−drug combinations, 2019

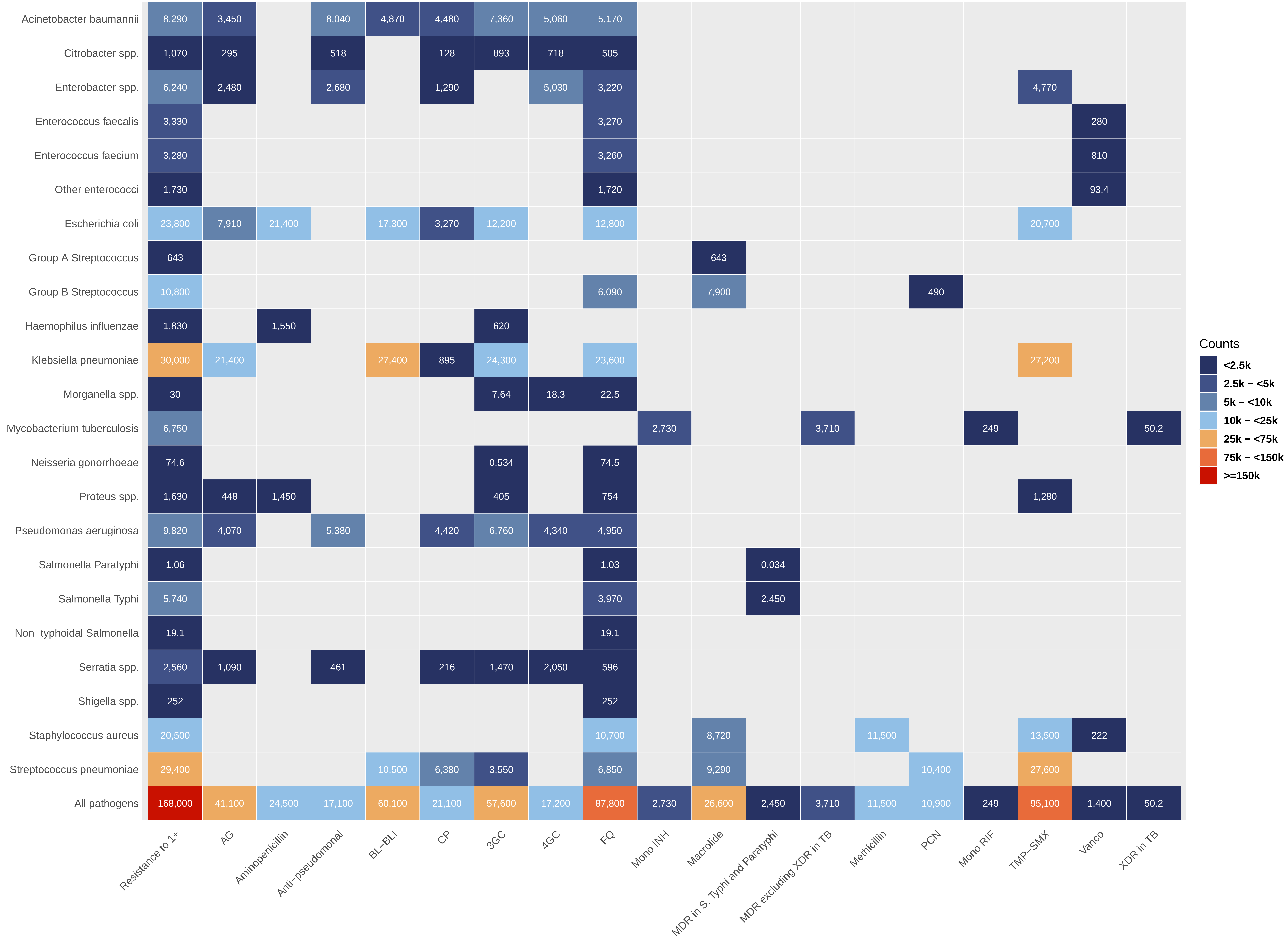

Congo (Brazzaville)

DALYs (count) *attributable to* bacterial antimicrobial resistance by pathogen−drug combinations, 2019

	Resistance to 1+	AG	Aminopenicillin	Anti−pseudomonal	BL−BLI	CP	3GC	4GC	FQ	Mono INH	Macrolide	MDR in S. Typhi and Paratyphi	MDR excluding XDR in TB	Methicillin	PCN	Mono RIF	TMP−SMX	Vanco	XDR in TB
Acinetobacter baumannii	2,540	158		887	0.107	762	65	0.207	664										
Citrobacter spp.	312	15.5		93.5		27.8	44	64.3	67.8										
Enterobacter spp.	1,490	134		229		264		324	306								237		
Enterococcus faecalis	885								810									75.8	
Enterococcus faecium	821								630									191	
Other enterococci	353								335									18.1	
Escherichia coli	5,950	462	243		654	683	1,440		1,200								1,270		
Group A Streptococcus	64.5										59.1								
Group B Streptococcus	1,830								1,040		680				84.9				
Haemophilus influenzae	406		228				178												
Klebsiella pneumoniae	7,850	1,470			406	283	3,050		1,450								1,190		
Morganella spp.	7.02						0.327	3.06	3.64										
Mycobacterium tuberculosis	2,660									433			2,130			67.5			30
Neisseria gonorrhoeae	7.41						0.151		7.26										
Proteus spp.	226	20	25.7				82.1		49.8								47		
Pseudomonas aeruginosa	2,500	116		580		779	394	91.3	537										
Salmonella Paratyphi	0.219								0.214			0.005							
Salmonella Typhi	1,050								770			269							
Non−typhoidal Salmonella	3.83								3.83										
Serratia spp.	618	62.8		92		62.5	57.7	285	59.5										
Shigella spp.	51.7								51.7										
Staphylococcus aureus	4,800								435		288			2,650			1,350	63.5	
Streptococcus pneumoniae	5,890				404	1,320	155		797		309				489		2,420		
All pathogens	40,300	2,440	498	1,880	1,460	4,180	5,470	768	9,230	485	1,370	283	2,130	2,650	574	67.5	6,510	348	30

Counts
- **<2.5k**
- **2.5k − <5k**
- **5k − <10k**
- **25k − <75k**

Cote d'Ivoire

DALYs (count) *associated with* bacterial antimicrobial resistance by pathogen−drug combinations, 2019

	Resistance to 1+	AG	Aminopenicillin	Anti−pseudomonal	BL−BLI	CP	3GC	4GC	FQ	Mono INH	Macrolide	MDR in S. Typhi and Paratyphi	MDR excluding XDR in TB	Methicillin	PCN	Mono RIF	TMP−SMX	Vanco	XDR in TB
Acinetobacter baumannii	51,800	22,800		51,500	21,600	20,700	32,800	28,000	22,700										
Citrobacter spp.	8,210	3,140		3,730		1,260	7,400	6,650	3,700										
Enterobacter spp.	50,100	26,500		20,300		9,140		42,600	31,100								41,200		
Enterococcus faecalis	24,100								23,800									1,560	
Enterococcus faecium	19,200								19,100									4,520	
Other enterococci	12,800								12,800									607	
Escherichia coli	214,000	72,200	205,000		175,000	23,800	115,000		116,000								196,000		
Group A Streptococcus	4,620										4,620								
Group B Streptococcus	113,000								75,300		73,400				2,220				
Haemophilus influenzae	18,500		13,400				8,730												
Klebsiella pneumoniae	306,000	220,000			284,000	11,300	258,000		227,000								291,000		
Morganella spp.	132						33.8	82.6	97.5										
Mycobacterium tuberculosis	34,700									12,000			22,300			106			298
Neisseria gonorrhoeae	283						0.121		282										
Proteus spp.	9,280	2,770	8,090				2,440		4,940								7,430		
Pseudomonas aeruginosa	90,200	43,800		47,100		37,300	55,600	41,500	51,600										
Salmonella Paratyphi	13								9.59			3.59							
Salmonella Typhi	91,700								14,600			84,100							
Non−typhoidal Salmonella	196								196										
Serratia spp.	26,900	13,200		3,620		1,280	21,800	24,000	6,920										
Shigella spp.	822								822										
Staphylococcus aureus	196,000								39,300		89,200			80,000			147,000	2,270	
Streptococcus pneumoniae	309,000				56,300	74,500	56,400		25,000		61,000				93,800		299,000		
All pathogens	1,580,000	404,000	226,000	126,000	537,000	179,000	558,000	143,000	675,000	12,000	228,000	84,100	22,300	80,000	96,000	106	981,000	8,950	298

Counts
- <2.5k
- 2.5k − <5k
- 5k − <10k
- 10k − <25k
- 25k − <75k
- 75k − <150k
- >=150k

Cote d'Ivoire

DALYs (count) *attributable to* bacterial antimicrobial resistance by pathogen−drug combinations, 2019

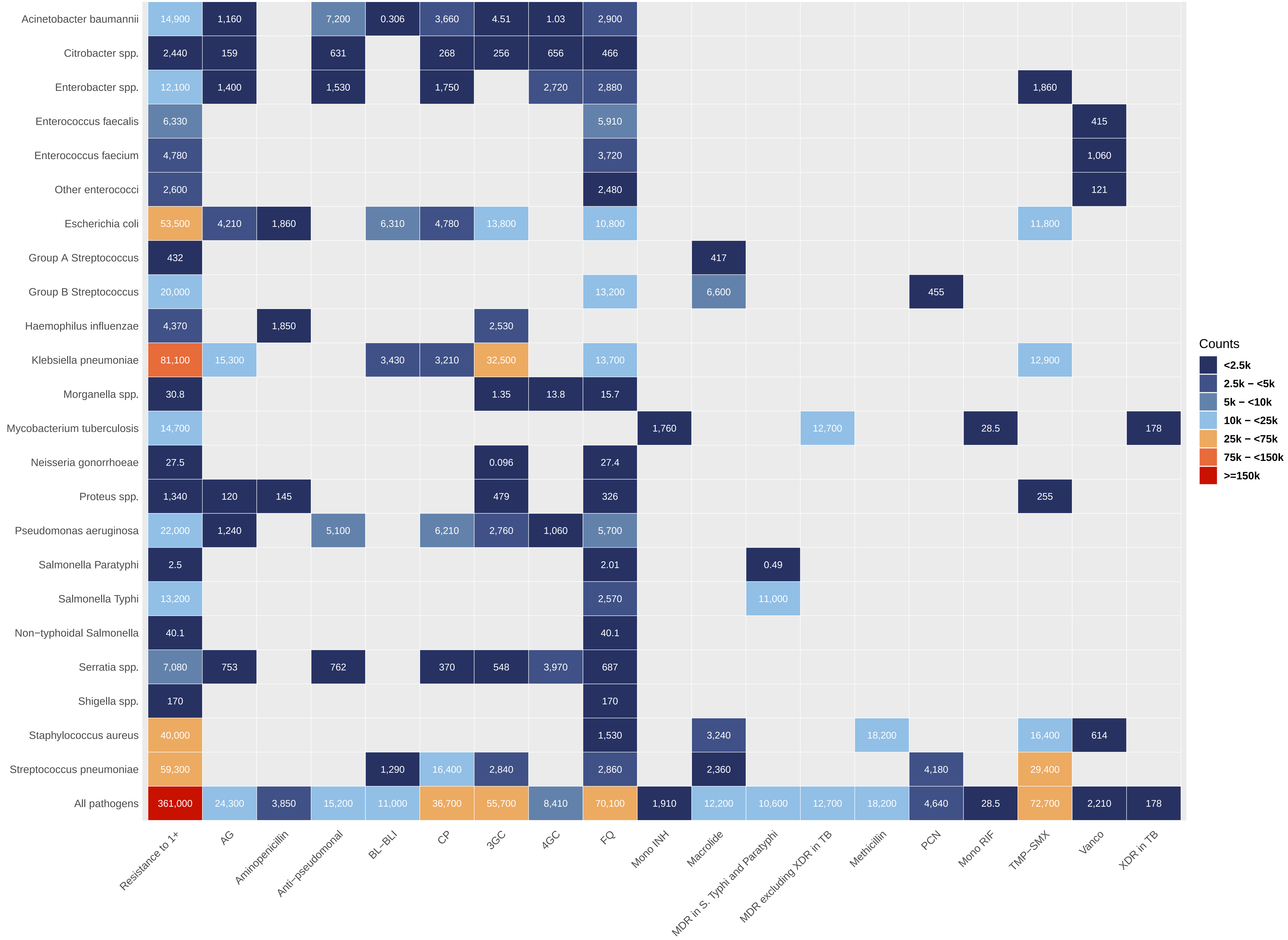

DR Congo

DALYs (count) *associated with* bacterial antimicrobial resistance by pathogen−drug combinations, 2019

	Resistance to 1+	AG	Aminopenicillin	Anti−pseudomonal	BL−BLI	CP	3GC	4GC	FQ	Mono INH	Macrolide	MDR in S. Typhi and Paratyphi	MDR excluding XDR in TB	Methicillin	PCN	Mono RIF	TMP−SMX	Vanco	XDR in TB
Acinetobacter baumannii	151,000	52,000		147,000	94,400	60,000	123,000	71,200	85,500										
Citrobacter spp.	19,400	5,790		10,100		2,380	14,500	14,300	8,640										
Enterobacter spp.	116,000	50,100		46,400		23,600		97,400	62,500								85,500		
Enterococcus faecalis	66,700								65,500									5,730	
Enterococcus faecium	55,700								55,400									13,800	
Other enterococci	27,700								27,600									1,930	
Escherichia coli	530,000	161,000	478,000		392,000	54,200	276,000		316,000								453,000		
Group A Streptococcus	21,200										21,200								
Group B Streptococcus	281,000								166,000		205,000				15,600				
Haemophilus influenzae	84,700		81,400				18,600												
Klebsiella pneumoniae	723,000	510,000			661,000	18,200	591,000		474,000								645,000		
Morganella spp.	427						105	274	312										
Mycobacterium tuberculosis	302,000									153,000			113,000			35,400			1,510
Neisseria gonorrhoeae	986						9.55		982										
Proteus spp.	29,400	8,150	26,300				7,180		14,500								23,300		
Pseudomonas aeruginosa	199,000	59,100		122,000		76,000	147,000	103,000	87,100										
Salmonella Paratyphi	10.1								6.54			3.7							
Salmonella Typhi	279,000								184,000			201,000							
Non−typhoidal Salmonella	23,400								23,400										
Serratia spp.	56,000	25,200		11,100		4,180	28,600	48,100	11,800										
Shigella spp.	6,700								6,700										
Staphylococcus aureus	439,000								175,000		177,000			203,000			302,000	4,920	
Streptococcus pneumoniae	814,000				282,000	153,000	85,900		492,000		234,000				254,000		720,000		
All pathogens	4,230,000	872,000	586,000	337,000	1,430,000	392,000	1,290,000	334,000	2,260,000	153,000	637,000	201,000	113,000	203,000	269,000	35,400	2,230,000	26,400	1,510

Counts
- <2.5k
- 2.5k − <5k
- 5k − <10k
- 10k − <25k
- 25k − <75k
- 75k − <150k
- >=150k

DR Congo

DALYs (count) *attributable to* bacterial antimicrobial resistance by pathogen−drug combinations, 2019

	Resistance to 1+	AG	Aminopenicillin	Anti−pseudomonal	BL−BLI	CP	3GC	4GC	FQ	Mono INH	Macrolide	MDR in S. Typhi and Paratyphi	MDR excluding XDR in TB	Methicillin	PCN	Mono RIF	TMP−SMX	Vanco	XDR in TB
Acinetobacter baumannii	45,100	2,450		20,100	11.9	10,200	967	3.31	11,300										
Citrobacter spp.	5,570	297		1,820		538	511	1,250	1,150										
Enterobacter spp.	27,900	2,690		3,800		4,800		6,740	5,900								4,040		
Enterococcus faecalis	17,700								16,200									1,530	
Enterococcus faecium	14,000								10,700									3,240	
Other enterococci	5,710								5,340									376	
Escherichia coli	132,000	9,180	5,070		14,900	11,100	33,500		30,900								27,300		
Group A Streptococcus	2,020										1,910								
Group B Streptococcus	48,700								28,200		18,600				2,610				
Haemophilus influenzae	17,500		12,400				5,120												
Klebsiella pneumoniae	189,000	36,200			10,600	6,120	78,000		28,700								29,100		
Morganella spp.	99.7						3.8	46	49.9										
Mycobacterium tuberculosis	98,000									22,700			64,300			9,560			907
Neisseria gonorrhoeae	97.5						2.78		94.7										
Proteus spp.	4,090	361	483				1,440		955								860		
Pseudomonas aeruginosa	52,300	1,730		16,300		13,800	8,420	2,620	9,480										
Salmonella Paratyphi	1.83								1.34			0.485							
Salmonella Typhi	51,600								30,700			20,700							
Non−typhoidal Salmonella	4,830								4,830										
Serratia spp.	13,200	1,500		2,140		1,240	638	6,610	1,150										
Shigella spp.	1,360								1,360										
Staphylococcus aureus	94,400								7,250		6,190			46,800			32,700	1,480	
Streptococcus pneumoniae	174,000				9,920	28,100	3,790		60,300		7,180				9,960		54,600		
All pathogens	999,000	54,300	18,000	44,200	35,500	75,900	132,000	17,300	255,000	24,500	33,100	21,100	64,300	46,800	12,600	9,560	149,000	6,620	907

Counts
- **<2.5k**
- **2.5k − <5k**
- **5k − <10k**
- **10k − <25k**
- **25k − <75k**
- **75k − <150k**
- **>=150k**

Equatorial Guinea

DALYs (count) *associated with* bacterial antimicrobial resistance by pathogen−drug combinations, 2019

	Resistance to 1+	AG	Aminopenicillin	Anti−pseudomonal	BL−BLI	CP	3GC	4GC	FQ	Mono INH	Macrolide	MDR in S. Typhi and Paratyphi	MDR excluding XDR in TB	Methicillin	PCN	Mono RIF	TMP−SMX	Vanco	XDR in TB
Acinetobacter baumannii	2,970	1,670		2,890	2,190	2,050	2,740	2,190	2,540										
Citrobacter spp.	561	45.1		213		134	426	318	277										
Enterobacter spp.	2,870	638		1,270		560		1,740	1,270								2,280		
Enterococcus faecalis	548								530									49.7	
Enterococcus faecium	642								638									172	
Other enterococci	617								616									30.9	
Escherichia coli	3,580	1,300	3,290		2,490	282	1,840		2,010								3,020		
Group A Streptococcus	133										133								
Group B Streptococcus	2,240								953		1,820				95.5				
Haemophilus influenzae	282		201				130												
Klebsiella pneumoniae	4,940	3,140			4,580	122	3,330		3,740								4,260		
Morganella spp.	3.36						0.834	0.707	3.07										
Mycobacterium tuberculosis	974									462			474			32.3			6.22
Neisseria gonorrhoeae	22.9						0.164		22.8										
Proteus spp.	298	96.7	265				62		132								234		
Pseudomonas aeruginosa	2,010	808		1,060		1,090	1,180	788	1,220										
Salmonella Paratyphi	0.244								0.222			0.025							
Salmonella Typhi	710								485			327							
Non−typhoidal Salmonella	6.38								6.38										
Serratia spp.	656	303		149		81.3	336	433	187										
Shigella spp.	13.9								13.9										
Staphylococcus aureus	2,880								1,840		839			1,740			1,740	33	
Streptococcus pneumoniae	6,850				2,300	1,540	879		1,160		2,270				3,400		6,270		
All pathogens	33,800	8,000	3,760	5,580	11,600	5,860	10,900	5,470	17,700	462	5,070	327	474	1,740	3,490	32.3	17,800	285	6.22

Counts
- <2.5k
- 2.5k − <5k
- 5k − <10k
- 10k − <25k
- 25k − <75k

Equatorial Guinea

DALYs (count) *attributable to* bacterial antimicrobial resistance by pathogen−drug combinations, 2019

	Resistance to 1+	AG	Aminopenicillin	Anti−pseudomonal	BL−BLI	CP	3GC	4GC	FQ	Mono INH	Macrolide	MDR in S. Typhi and Paratyphi	MDR excluding XDR in TB	Methicillin	PCN	Mono RIF	TMP−SMX	Vanco	XDR in TB
Acinetobacter baumannii	933	70.5		181	0.198	331	20.2	0.119	330										
Citrobacter spp.	164	2.2		34.3		30.2	28.7	27.9	40.5										
Enterobacter spp.	636	37.5		123		118		95.9	131								131		
Enterococcus faecalis	147								132									14.8	
Enterococcus faecium	163								122									40.2	
Other enterococci	126								120									5.96	
Escherichia coli	876	75.6	47.1		98.1	57.9	229		191								178		
Group A Streptococcus	12.4										13.2								
Group B Streptococcus	348								161		166				17.2				
Haemophilus influenzae	66.5		28.6				37.8												
Klebsiella pneumoniae	1,220	217			123	42.2	421		233								189		
Morganella spp.	0.758						0.092	0.116	0.55										
Mycobacterium tuberculosis	335									65.5			257			8.41			3.63
Neisseria gonorrhoeae	2.28						0.046		2.23										
Proteus spp.	39.3	4.24	4.95				12.7		8.67								8.45		
Pseudomonas aeruginosa	497	20.9		88.6		186	54.9	12.6	134										
Salmonella Paratyphi	0.049								0.046			0.003							
Salmonella Typhi	129								92.5			38.1							
Non−typhoidal Salmonella	1.31								1.31										
Serratia spp.	153	17.4		27.9		21.4	15.7	51	19.2										
Shigella spp.	2.68								2.68										
Staphylococcus aureus	699								79.3		26.7			412			172	9.7	
Streptococcus pneumoniae	1,410				86.1	323	39.6		130		81.9				209		544		
All pathogens	7,960	445	80.8	455	308	1,110	859	188	1,930	66.6	286	35.9	257	412	226	8.41	1,220	70.6	3.63

Counts
<2.5k
5k − <10k

Eritrea

DALYs (count) *associated with* bacterial antimicrobial resistance by pathogen−drug combinations, 2019

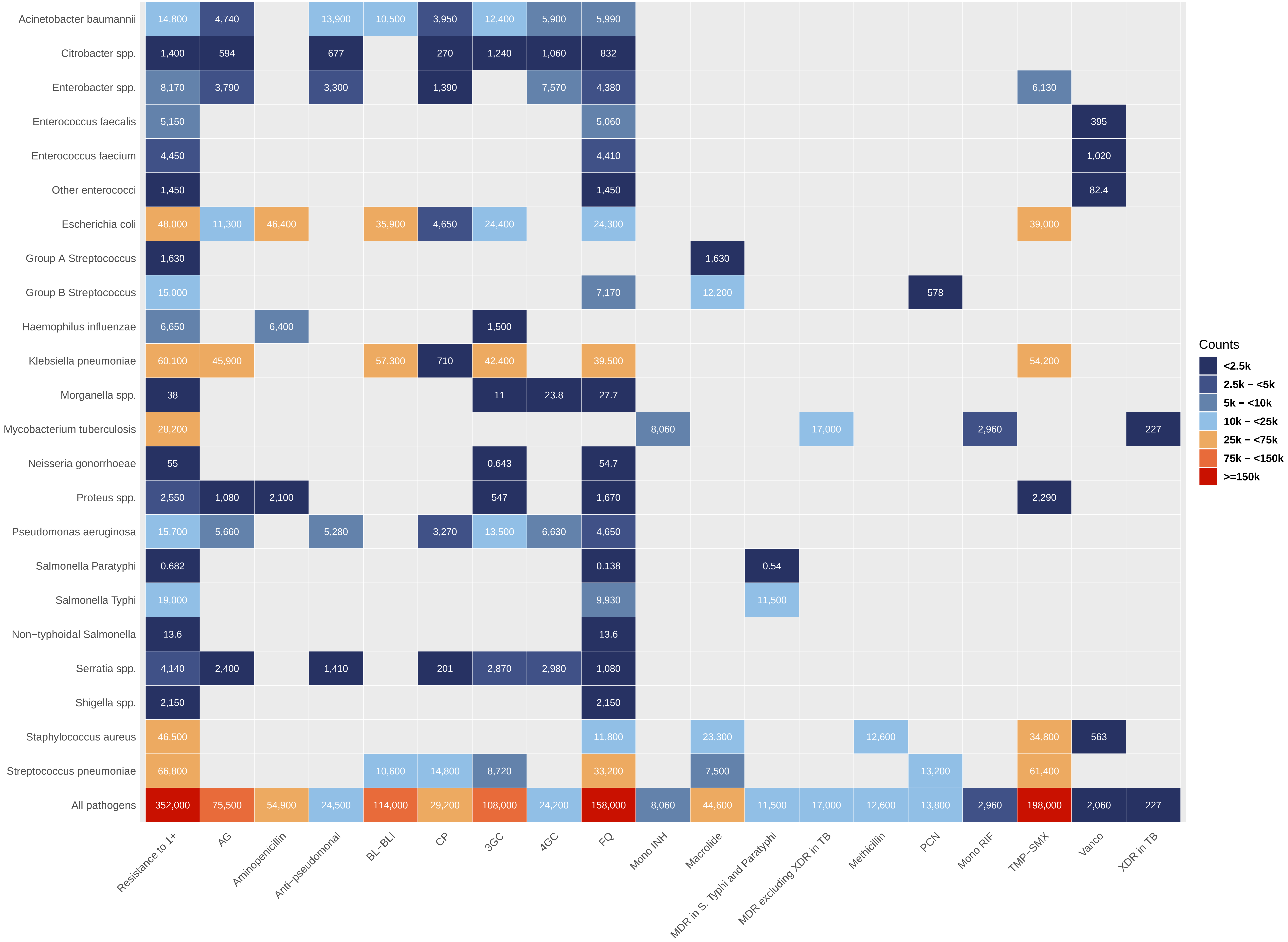

Eritrea

DALYs (count) *attributable to* bacterial antimicrobial resistance by pathogen−drug combinations, 2019

	Resistance to 1+	AG	Aminopenicillin	Anti−pseudomonal	BL−BLI	CP	3GC	4GC	FQ	Mono INH	Macrolide	MDR in S. Typhi and Paratyphi	MDR excluding XDR in TB	Methicillin	PCN	Mono RIF	TMP−SMX	Vanco	XDR in TB
Acinetobacter baumannii	4,360	251		2,320	22.2	720	236	0.296	810										
Citrobacter spp.	419	28.1		101		52.2	44.5	83.7	109										
Enterobacter spp.	1,990	200		282		280		578	384								274		
Enterococcus faecalis	1,360								1,260									106	
Enterococcus faecium	1,110								862									247	
Other enterococci	300								283									17.1	
Escherichia coli	11,800	667	707		1,570	984	3,160		2,360								2,350		
Group A Streptococcus	151										158								
Group B Streptococcus	2,400								1,180		1,150				106				
Haemophilus influenzae	1,380		968				412												
Klebsiella pneumoniae	15,000	3,240			1,440	308	5,400		2,330								2,340		
Morganella spp.	9						0.519	4.05	4.43										
Mycobacterium tuberculosis	11,700									1,230			9,570			798			135
Neisseria gonorrhoeae	5.45						0.183		5.27										
Proteus spp.	367	45.6	34.9				100		109								80.5		
Pseudomonas aeruginosa	4,060	176		802		602	1,540	425	514										
Salmonella Paratyphi	0.104								0.032			0.07							
Salmonella Typhi	3,270								1,900			1,340							
Non−typhoidal Salmonella	2.71								2.71										
Serratia spp.	1,020	142		271		60.3	108	332	105										
Shigella spp.	444								444										
Staphylococcus aureus	8,210								469		867			2,740			3,980	158	
Streptococcus pneumoniae	13,600				238	2,870	302		4,190		267				420		5,330		
All pathogens	83,000	4,750	1,710	3,780	3,260	5,880	11,300	1,420	17,300	1,250	2,390	1,340	9,570	2,740	526	798	14,300	527	135

Counts
- <2.5k
- 2.5k − <5k
- 5k − <10k
- 10k − <25k
- 75k − <150k

Ethiopia

DALYs (count) *associated with* bacterial antimicrobial resistance by pathogen−drug combinations, 2019

	Resistance to 1+	AG	Aminopenicillin	Anti−pseudomonal	BL−BLI	CP	3GC	4GC	FQ	Mono INH	Macrolide	MDR in S. Typhi and Paratyphi	MDR excluding XDR in TB	Methicillin	PCN	Mono RIF	TMP−SMX	Vanco	XDR in TB
Acinetobacter baumannii	184,000	66,500		183,000	175,000	62,600	176,000	173,000	124,000										
Citrobacter spp.	38,400	11,900		17,000		6,620	32,400	22,900	23,600										
Enterobacter spp.	192,000	84,300		72,700		21,800		170,000	103,000								142,000		
Enterococcus faecalis	86,300								84,700									6,010	
Enterococcus faecium	75,300								74,600									18,600	
Other enterococci	46,000								45,800									3,580	
Escherichia coli	723,000	108,000	680,000		456,000	41,600	473,000		458,000								619,000		
Group A Streptococcus	23,900										23,900								
Group B Streptococcus	333,000								124,000		289,000				9,560				
Haemophilus influenzae	69,000		65,200				12,900												
Klebsiella pneumoniae	940,000	684,000			913,000	346,000	918,000		535,000								833,000		
Morganella spp.	458						110	271	338										
Mycobacterium tuberculosis	111,000									14,000			84,000			11,800			1,110
Neisseria gonorrhoeae	540						19.4		535										
Proteus spp.	40,400	17,400	34,300				7,080		22,600								35,200		
Pseudomonas aeruginosa	224,000	67,000		91,000		61,200	183,000	77,500	94,700										
Salmonella Paratyphi	8.2								2.44			4.92							
Salmonella Typhi	197,000								116,000			95,300							
Non−typhoidal Salmonella	36,600								36,600										
Serratia spp.	70,700	29,900		26,300		3,740	40,200	44,600	19,400										
Shigella spp.	50,600								50,600										
Staphylococcus aureus	533,000								115,000		342,000			280,000			323,000	5,910	
Streptococcus pneumoniae	995,000				167,000	246,000	130,000		158,000		122,000				242,000		964,000		
All pathogens	4,970,000	1,070,000	780,000	389,000	1,710,000	789,000	1,970,000	488,000	2,190,000	14,000	777,000	95,300	84,000	280,000	251,000	11,800	2,920,000	34,100	1,110

Counts
- <2.5k
- 2.5k − <5k
- 5k − <10k
- 10k − <25k
- 25k − <75k
- 75k − <150k
- >=150k

Ethiopia

DALYs (count) *attributable to* bacterial antimicrobial resistance by pathogen−drug combinations, 2019

	Resistance to 1+	AG	Aminopenicillin	Anti−pseudomonal	BL−BLI	CP	3GC	4GC	FQ	Mono INH	Macrolide	MDR in S. Typhi and Paratyphi	MDR excluding XDR in TB	Methicillin	PCN	Mono RIF	TMP−SMX	Vanco	XDR in TB
Acinetobacter baumannii	56,800	3,030		26,500	70.7	10,000	329	38.2	16,800										
Citrobacter spp.	11,300	584		2,650		1,250	1,820	1,770	3,260										
Enterobacter spp.	45,200	4,440		6,670		4,620		13,600	9,370								6,550		
Enterococcus faecalis	22,800								21,100									1,680	
Enterococcus faecium	18,900								14,500									4,450	
Other enterococci	9,530								8,840									689	
Escherichia coli	185,000	6,110	10,300		13,400	8,560	65,400		45,300								36,300		
Group A Streptococcus	2,290										2,430								
Group B Streptococcus	49,500								20,200		26,400				1,850				
Haemophilus influenzae	14,100		10,400				3,670												
Klebsiella pneumoniae	295,000	51,800			2,750	68,300	101,000		33,800								37,300		
Morganella spp.	107						5.04	45.9	55.6										
Mycobacterium tuberculosis	53,100									2,130			47,200			3,160			660
Neisseria gonorrhoeae	56.1						4.19		51.9										
Proteus spp.	5,440	746	634				1,380		1,470								1,220		
Pseudomonas aeruginosa	58,500	2,020		12,500		10,900	19,400	2,820	10,900										
Salmonella Paratyphi	1.39								0.689			0.685							
Salmonella Typhi	34,700								23,000			11,200							
Non−typhoidal Salmonella	7,360								7,360										
Serratia spp.	16,700	1,720		5,360		1,170	1,600	4,870	1,990										
Shigella spp.	10,400								10,400										
Staphylococcus aureus	118,000								4,610		12,700			66,300			33,000	1,580	
Streptococcus pneumoniae	189,000				4,170	53,400	3,940		18,600		4,490				9,600		94,700		
All pathogens	1,200,000	70,400	21,300	53,700	20,300	158,000	199,000	23,200	252,000	2,070	46,900	11,500	47,200	66,300	11,400	3,160	209,000	8,390	660

Counts
- <2.5k
- 2.5k − <5k
- 5k − <10k
- 10k − <25k
- 25k − <75k
- 75k − <150k
- >=150k

Gabon

DALYs (count) *associated with* bacterial antimicrobial resistance by pathogen−drug combinations, 2019

	Resistance to 1+	AG	Aminopenicillin	Anti−pseudomonal	BL−BLI	CP	3GC	4GC	FQ	Mono INH	Macrolide	MDR in S. Typhi and Paratyphi	MDR excluding XDR in TB	Methicillin	PCN	Mono RIF	TMP−SMX	Vanco	XDR in TB
Acinetobacter baumannii	4,120	1,980		3,900	2,730	2,310	3,680	3,110	3,190										
Citrobacter spp.	455	125		176		50.6	356	191	204										
Enterobacter spp.	2,680	769		944		462		1,680	1,320								2,070		
Enterococcus faecalis	859								844									56.8	
Enterococcus faecium	996								989									255	
Other enterococci	600								599									39.7	
Escherichia coli	5,280	2,620	4,910		3,680	522	2,360		2,570								4,300		
Group A Streptococcus	154										154								
Group B Streptococcus	1,640								567		1,380				53.2				
Haemophilus influenzae	398		347				109												
Klebsiella pneumoniae	6,170	3,660			5,390	145	4,540		4,730								5,440		
Morganella spp.	9.01						2.26	2.32	8.02										
Mycobacterium tuberculosis	1,710									512			1,140			46.3			15.2
Neisseria gonorrhoeae	27						0.168		26.9										
Proteus spp.	445	19.5	392				95		137								325		
Pseudomonas aeruginosa	2,340	861		1,250		1,180	1,330	946	1,410										
Salmonella Paratyphi	0.242								0.235			0.007							
Salmonella Typhi	613								415			267							
Non−typhoidal Salmonella	4.12								4.12										
Serratia spp.	672	243		116		192	349	506	160										
Shigella spp.	32.6								32.6										
Staphylococcus aureus	3,790								2,210		1,780			2,790			1,420	129	
Streptococcus pneumoniae	7,600				2,720	1,530	935		1,320		2,780				2,660		6,950		
All pathogens	40,600	10,300	5,650	6,380	14,500	6,390	13,800	6,430	20,700	512	6,090	267	1,140	2,790	2,710	46.3	20,500	481	15.2

Counts
- <2.5k
- 2.5k − <5k
- 5k − <10k
- 10k − <25k
- 25k − <75k

Gabon

DALYs (count) *attributable to* bacterial antimicrobial resistance by pathogen−drug combinations, 2019

	Resistance to 1+	AG	Aminopenicillin	Anti−pseudomonal	BL−BLI	CP	3GC	4GC	FQ	Mono INH	Macrolide	MDR in S. Typhi and Paratyphi	MDR excluding XDR in TB	Methicillin	PCN	Mono RIF	TMP−SMX	Vanco	XDR in TB
Acinetobacter baumannii	1,270	86.6		332	1.28	375	59.3	0.57	417										
Citrobacter spp.	130	7.2		33.8		10.9	31	17.4	29.8										
Enterobacter spp.	596	44.1		87		95.9		113	140								117		
Enterococcus faecalis	226								210									15.8	
Enterococcus faecium	251								191									60.2	
Other enterococci	123								116									7.66	
Escherichia coli	1,270	166	79.1		159	108	275		233								248		
Group A Streptococcus	14.5										14.6								
Group B Streptococcus	241								96.1		135				10.3				
Haemophilus influenzae	85.5		54				31.4												
Klebsiella pneumoniae	1,550	253			108	51.9	583		304								251		
Morganella spp.	2.05						0.232	0.385	1.44										
Mycobacterium tuberculosis	736									79.5			639			12.4			9.01
Neisseria gonorrhoeae	2.66						0.048		2.61										
Proteus spp.	54.9	1.31	8.54				21.7		9.99								13.7		
Pseudomonas aeruginosa	577	23.1		119		202	59.2	16.8	158										
Salmonella Paratyphi	0.05								0.049			0.001							
Salmonella Typhi	111								80.4			31.5							
Non−typhoidal Salmonella	0.809								0.809										
Serratia spp.	169	13.5		18.9		49.5	14.8	56.5	16										
Shigella spp.	6.43								6.43										
Staphylococcus aureus	1,020								96		62.6			701			132	31.9	
Streptococcus pneumoniae	1,490				112	322	49.2		156		97.4				136		616		
All pathogens	9,930	595	142	591	381	1,210	1,130	204	2,260	75.9	309	31.5	639	701	146	12.4	1,380	116	9.01

Counts
<2.5k
5k − <10k

Ghana

DALYs (count) *associated with* bacterial antimicrobial resistance by pathogen−drug combinations, 2019

	Resistance to 1+	AG	Aminopenicillin	Anti−pseudomonal	BL−BLI	CP	3GC	4GC	FQ	Mono INH	Macrolide	MDR in S. Typhi and Paratyphi	MDR excluding XDR in TB	Methicillin	PCN	Mono RIF	TMP−SMX	Vanco	XDR in TB
Acinetobacter baumannii	54,500	25,100		54,200	20,700	22,700	21,300	16,900	24,800										
Citrobacter spp.	7,730	2,040		2,190		1,220	7,120	6,170	2,680										
Enterobacter spp.	38,300	19,000		13,500		9,670		31,300	26,600								29,400		
Enterococcus faecalis	22,300								21,800									1,540	
Enterococcus faecium	27,300								27,200									6,590	
Other enterococci	13,500								13,500									579	
Escherichia coli	160,000	97,900	153,000		137,000	7,270	111,000		115,000								144,000		
Group A Streptococcus	4,820										4,820								
Group B Streptococcus	38,300								14,700		31,200				594				
Haemophilus influenzae	7,020		5,100				3,080												
Klebsiella pneumoniae	231,000	181,000			213,000	3,110	191,000		164,000								218,000		
Morganella spp.	101						23.3	43.3	81.7										
Mycobacterium tuberculosis	30,200									7,120			22,700			8.79			306
Neisseria gonorrhoeae	429						0.856		428										
Proteus spp.	11,200	4,300	8,530				5,850		7,760								8,780		
Pseudomonas aeruginosa	75,000	48,600		36,700		22,200	31,000	17,700	45,600										
Salmonella Paratyphi	161								144			19.2							
Salmonella Typhi	105,000								7,450			101,000							
Non−typhoidal Salmonella	1.43								1.43										
Serratia spp.	27,900	13,500		3,640		1,530	27,700	25,300	11,700										
Shigella spp.	206								206										
Staphylococcus aureus	154,000								56,100		69,700			70,700			95,800	4,010	
Streptococcus pneumoniae	221,000				16,500	40,100	20,000		35,300		19,600				53,400		216,000		
All pathogens	1,230,000	392,000	166,000	110,000	387,000	108,000	418,000	97,500	575,000	7,120	125,000	101,000	22,700	70,700	54,000	8.79	712,000	12,700	306

Counts
- <2.5k
- 2.5k − <5k
- 5k − <10k
- 10k − <25k
- 25k − <75k
- 75k − <150k
- >=150k

Ghana

DALYs (count) *attributable to* bacterial antimicrobial resistance by pathogen−drug combinations, 2019

	Resistance to 1+	AG	Aminopenicillin	Anti−pseudomonal	BL−BLI	CP	3GC	4GC	FQ	Mono INH	Macrolide	MDR in S. Typhi and Paratyphi	MDR excluding XDR in TB	Methicillin	PCN	Mono RIF	TMP−SMX	Vanco	XDR in TB
Acinetobacter baumannii	15,100	1,240		6,730	0.33	3,980	1.23	1.03	3,120										
Citrobacter spp.	2,290	109		390		277	354	833	333										
Enterobacter spp.	9,520	989		852		1,790		1,950	2,650								1,320		
Enterococcus faecalis	5,890								5,440									449	
Enterococcus faecium	6,820								5,270									1,540	
Other enterococci	2,760								2,640									117	
Escherichia coli	41,700	5,610	681		3,100	1,400	13,300		10,400								7,290		
Group A Streptococcus	451										453								
Group B Streptococcus	5,720								2,520		3,080				164				
Haemophilus influenzae	1,640		743				897												
Klebsiella pneumoniae	60,000	12,700			2,980	1,280	23,700		9,700								9,640		
Morganella spp.	23						1.69	7.29	14.1										
Mycobacterium tuberculosis	14,200									1,020			13,000			2.38			182
Neisseria gonorrhoeae	42.3						0.416		41.9										
Proteus spp.	2,130	180	83.7				1,050		522								294		
Pseudomonas aeruginosa	15,700	1,380		4,320		3,320	1,370	121	5,250										
Salmonella Paratyphi	32.6								30			2.57							
Salmonella Typhi	14,600								1,330			12,400							
Non−typhoidal Salmonella	0.283								0.283										
Serratia spp.	7,870	743		599		364	745	4,250	1,160										
Shigella spp.	41.6								41.6										
Staphylococcus aureus	33,100								2,380		2,580			16,600			10,500	1,060	
Streptococcus pneumoniae	39,600				314	8,840	742		4,170		768				2,970		21,800		
All pathogens	279,000	23,000	1,510	12,900	6,390	21,300	42,100	7,170	57,000	1,040	6,830	12,800	13,000	16,600	3,130	2.38	50,800	3,170	182

Counts
- <2.5k
- 2.5k − <5k
- 5k − <10k
- 10k − <25k
- 25k − <75k
- >=150k

Guinea

DALYs (count) *associated with* bacterial antimicrobial resistance by pathogen−drug combinations, 2019

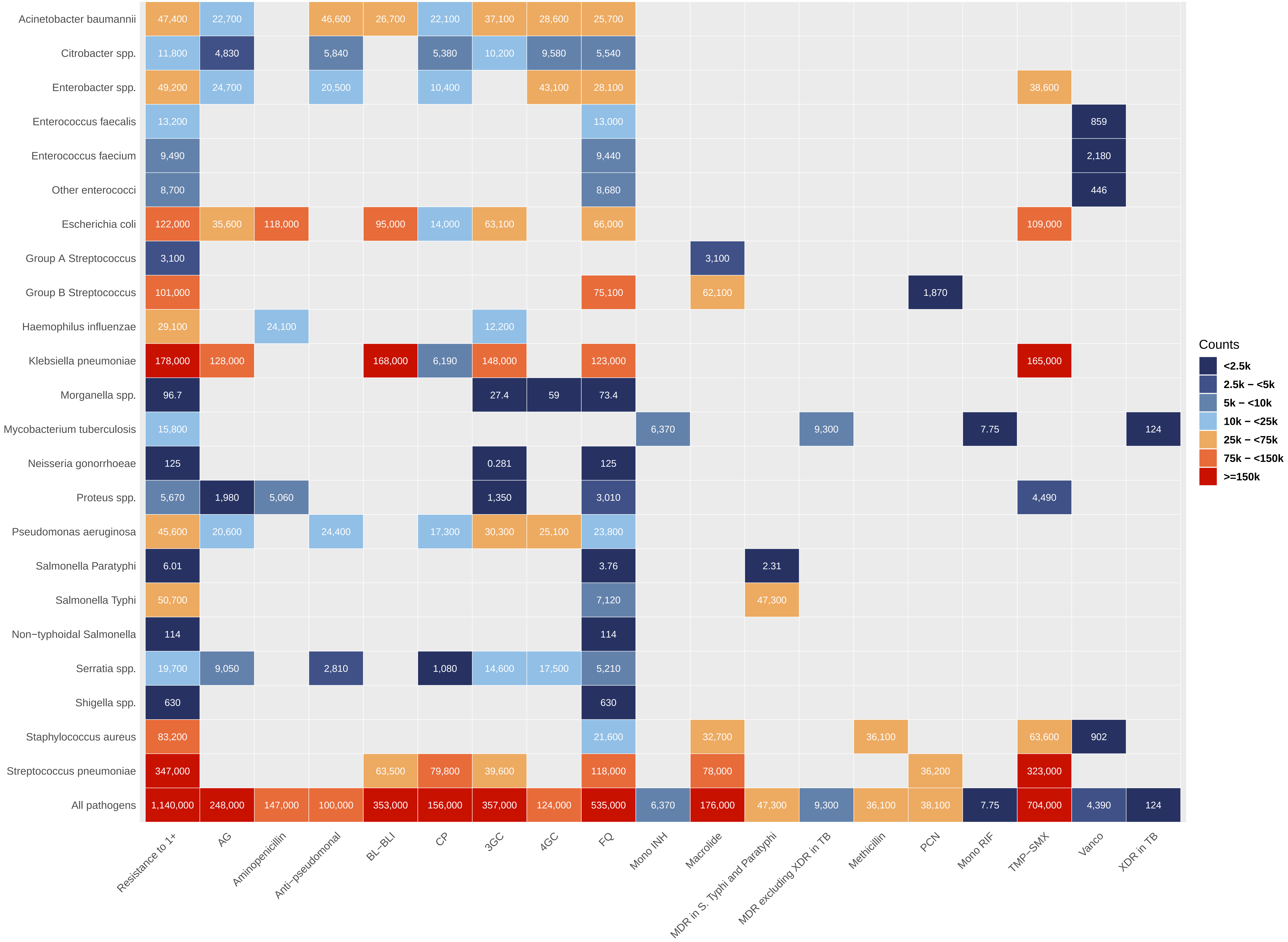

Guinea

DALYs (count) *attributable to* bacterial antimicrobial resistance by pathogen−drug combinations, 2019

	Resistance to 1+	AG	Aminopenicillin	Anti−pseudomonal	BL−BLI	CP	3GC	4GC	FQ	Mono INH	Macrolide	MDR in S. Typhi and Paratyphi	MDR excluding XDR in TB	Methicillin	PCN	Mono RIF	TMP−SMX	Vanco	XDR in TB
Acinetobacter baumannii	14,200	1,100		5,830	0.903	3,800	142	1.09	3,300										
Citrobacter spp.	3,620	231		660		1,170	287	586	676										
Enterobacter spp.	12,100	1,310		1,580		2,050		2,820	2,550								1,740		
Enterococcus faecalis	3,470								3,240									224	
Enterococcus faecium	2,350								1,840									516	
Other enterococci	1,780								1,690									89.2	
Escherichia coli	30,500	2,070	1,480		3,650	2,830	7,620		6,250								6,550		
Group A Streptococcus	298										287								
Group B Streptococcus	18,600								13,200		4,920				369				
Haemophilus influenzae	6,690		3,230				3,460												
Klebsiella pneumoniae	47,200	9,020			2,490	1,810	19,200		7,440								7,270		
Morganella spp.	22.8						1.26	9.76	11.8										
Mycobacterium tuberculosis	6,390									989			5,320			2.12			74.2
Neisseria gonorrhoeae	12.2						0.125		12.1										
Proteus spp.	796	85	91.2				266		196								155		
Pseudomonas aeruginosa	11,400	571		2,920		2,940	1,510	859	2,590										
Salmonella Paratyphi	1.11								0.794			0.309							
Salmonella Typhi	7,100								1,190			5,790							
Non−typhoidal Salmonella	23.8								23.8										
Serratia spp.	5,060	506		586		312	367	2,780	510										
Shigella spp.	128								128										
Staphylococcus aureus	17,500								845		1,140			8,200			7,080	258	
Streptococcus pneumoniae	67,100				1,680	16,100	1,080		15,000		2,780				561		29,900		
All pathogens	256,000	14,900	4,800	11,600	7,820	31,100	33,900	7,060	60,600	980	9,300	5,930	5,320	8,200	930	2.12	52,700	1,090	74.2

Counts
- <2.5k
- 2.5k – <5k
- 5k – <10k
- 10k – <25k
- 25k – <75k
- >=150k

Guinea−Bissau

DALYs (count) *associated with* bacterial antimicrobial resistance by pathogen−drug combinations, 2019

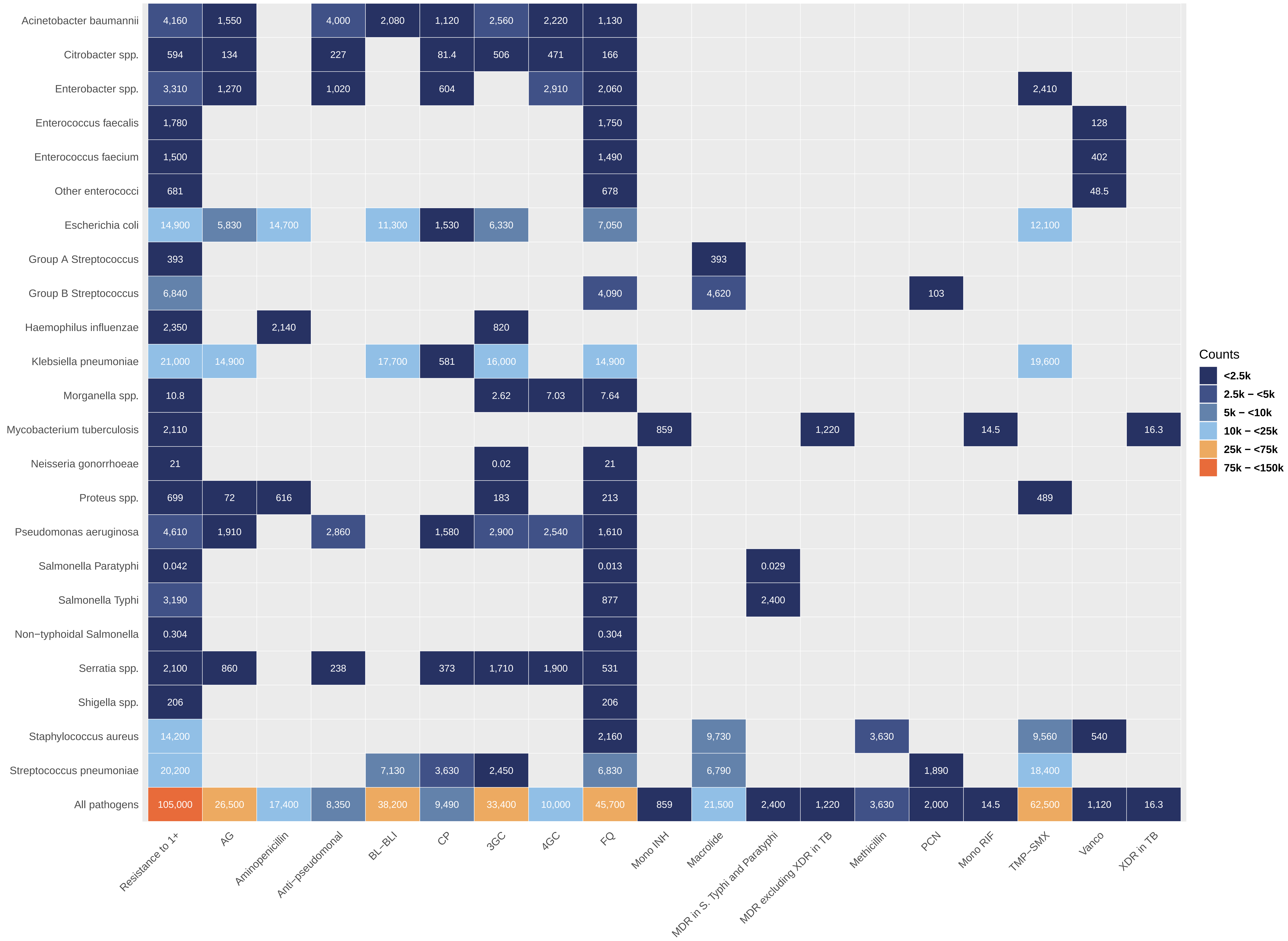

Guinea−Bissau

DALYs (count) *attributable to* bacterial antimicrobial resistance by pathogen−drug combinations, 2019

	Resistance to 1+	AG	Aminopenicillin	Anti−pseudomonal	BL−BLI	CP	3GC	4GC	FQ	Mono INH	Macrolide	MDR in S. Typhi and Paratyphi	MDR excluding XDR in TB	Methicillin	PCN	Mono RIF	TMP−SMX	Vanco	XDR in TB
Acinetobacter baumannii	1,180	91		661	1.37	238	29.8	0.16	163										
Citrobacter spp.	173	7.23		43.9		19.7	23.5	57.7	20.8										
Enterobacter spp.	806	64.2		77.7		121		237	199								107		
Enterococcus faecalis	470								435									34.8	
Enterococcus faecium	379								285									94.3	
Other enterococci	141								132									9.58	
Escherichia coli	3,590	356	268		533	317	761		661								696		
Group A Streptococcus	37.9										36.7								
Group B Streptococcus	1,160								718		440				26.2				
Haemophilus influenzae	520		292				227												
Klebsiella pneumoniae	5,360	1,050			271	194	2,020		905								914		
Morganella spp.	2.53						0.097	1.2	1.23										
Mycobacterium tuberculosis	840									126			697			3.97			9.83
Neisseria gonorrhoeae	2.04						0.013		2.03										
Proteus spp.	92.2	3.93	13				40.7		15.1								19.7		
Pseudomonas aeruginosa	1,150	56.4		418		295	126	85.1	171										
Salmonella Paratyphi	0.007								0.003			0.004							
Salmonella Typhi	500								181			317							
Non−typhoidal Salmonella	0.063								0.063										
Serratia spp.	566	47.7		43.6		92.3	41.3	290	51.6										
Shigella spp.	42.9								42.9										
Staphylococcus aureus	2,490								89.3		376			848			1,030	146	
Streptococcus pneumoniae	3,890				272	743	138		856		236				35.8		1,610		
All pathogens	23,400	1,680	573	1,240	1,080	2,020	3,410	671	4,930	128	1,060	317	697	848	62	3.97	4,380	285	9.83

Counts
- <2.5k
- 2.5k − <5k
- 5k − <10k
- 10k − <25k

Kenya

DALYs (count) *associated with* bacterial antimicrobial resistance by pathogen−drug combinations, 2019

	Resistance to 1+	AG	Aminopenicillin	Anti−pseudomonal	BL−BLI	CP	3GC	4GC	FQ	Mono INH	Macrolide	MDR in S. Typhi and Paratyphi	MDR excluding XDR in TB	Methicillin	PCN	Mono RIF	TMP−SMX	Vanco	XDR in TB
Acinetobacter baumannii	71,100	27,400		68,000	49,900	37,600	65,400	39,600	47,500										
Citrobacter spp.	10,600	3,040		4,530		2,210	9,200	6,100	7,440										
Enterobacter spp.	56,600	24,200		24,900		8,300		48,700	33,600								43,100		
Enterococcus faecalis	39,900								39,500									1,990	
Enterococcus faecium	35,800								35,500									10,100	
Other enterococci	15,000								15,000									592	
Escherichia coli	271,000	84,700	264,000		142,000	10,600	140,000		129,000								239,000		
Group A Streptococcus	7,240										7,240								
Group B Streptococcus	87,700								43,100		70,900				764				
Haemophilus influenzae	25,100		23,800				5,010												
Klebsiella pneumoniae	302,000	204,000			249,000	9,980	221,000		213,000								286,000		
Morganella spp.	231						51	130	180										
Mycobacterium tuberculosis	57,500									26,600			23,700			6,870			315
Neisseria gonorrhoeae	398						0.32		398										
Proteus spp.	18,000	6,720	15,500				3,290		10,500								15,800		
Pseudomonas aeruginosa	83,700	20,900		61,600		35,600	55,500	33,200	25,000										
Salmonella Paratyphi	281								48			232							
Salmonella Typhi	172,000								103,000			119,000							
Non−typhoidal Salmonella	280								280										
Serratia spp.	22,800	11,800		7,670		1,610	13,700	13,100	7,330										
Shigella spp.	14,100								14,100										
Staphylococcus aureus	213,000								58,800		116,000			61,900			144,000	2,900	
Streptococcus pneumoniae	309,000				24,200	108,000	29,100		24,100		74,200				161,000		295,000		
All pathogens	1,810,000	383,000	303,000	167,000	465,000	214,000	542,000	141,000	808,000	26,600	268,000	119,000	23,700	61,900	161,000	6,870	1,020,000	15,600	315

Counts
- <2.5k
- 2.5k − <5k
- 5k − <10k
- 10k − <25k
- 25k − <75k
- 75k − <150k
- >=150k

Kenya

DALYs (count) *attributable to* bacterial antimicrobial resistance by pathogen−drug combinations, 2019

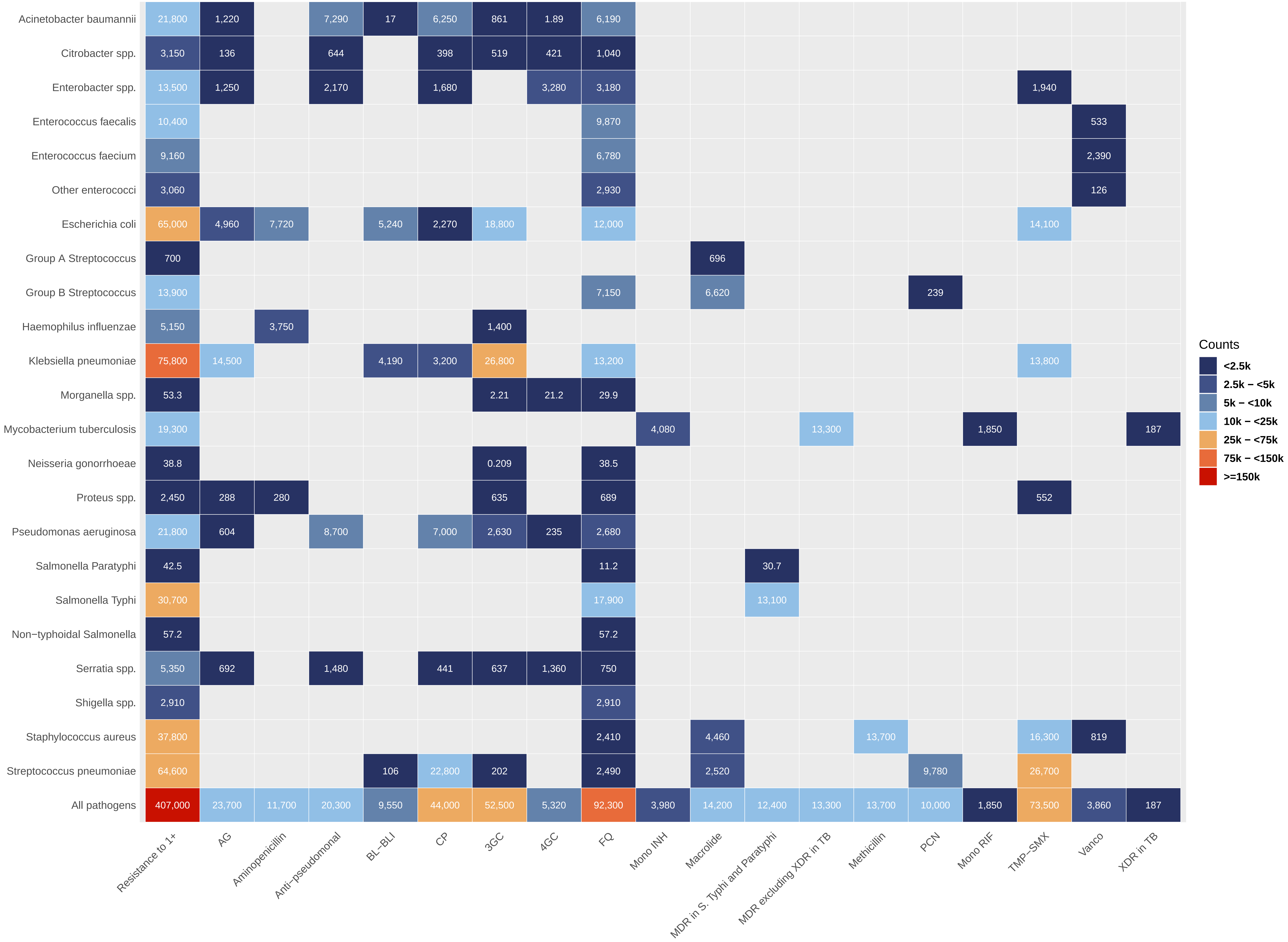

Lesotho

DALYs (count) *associated with* bacterial antimicrobial resistance by pathogen−drug combinations, 2019

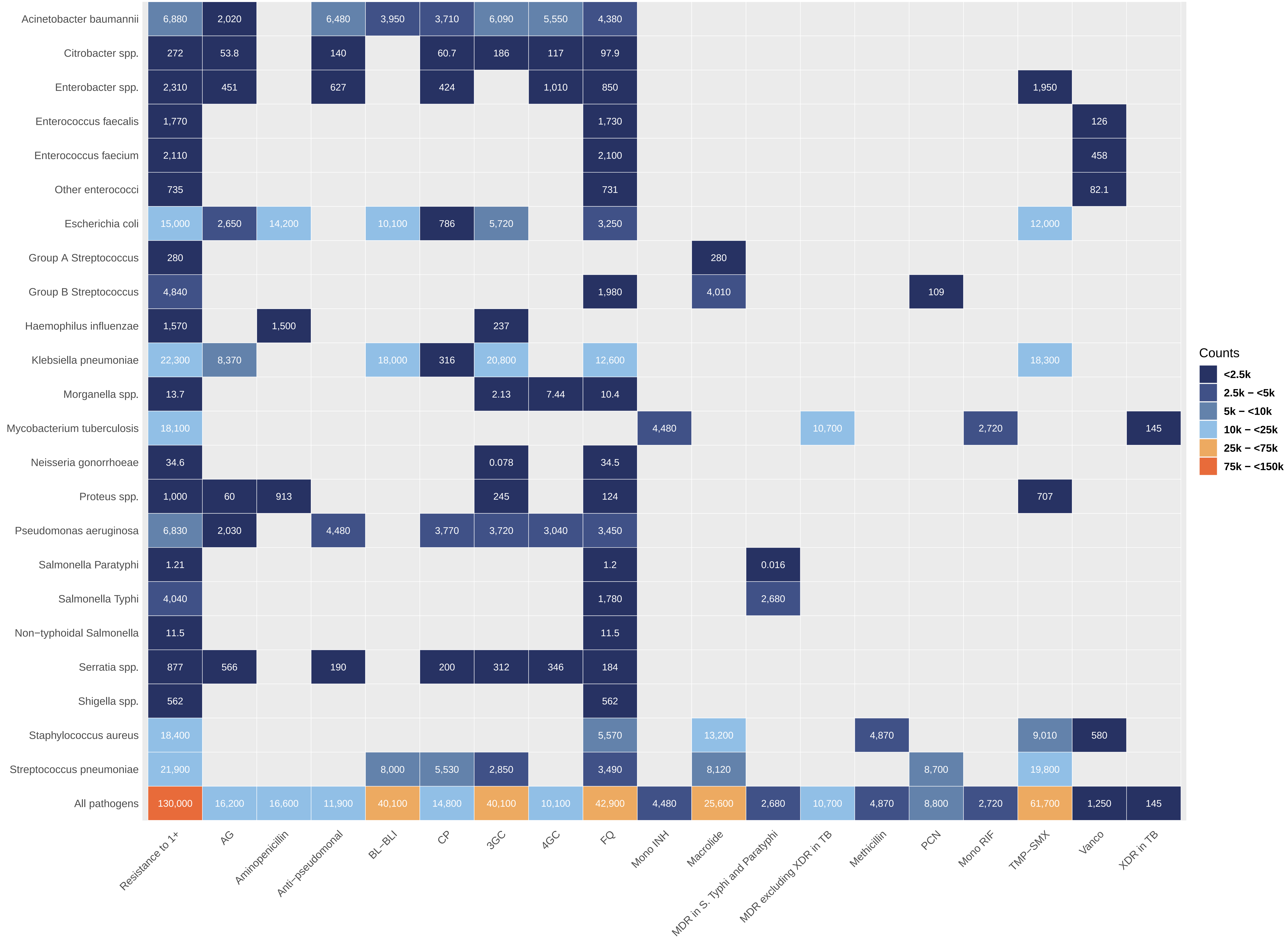

Lesotho

DALYs (count) *attributable to* bacterial antimicrobial resistance by pathogen−drug combinations, 2019

	Resistance to 1+	AG	Aminopenicillin	Anti−pseudomonal	BL−BLI	CP	3GC	4GC	FQ	Mono INH	Macrolide	MDR in S. Typhi and Paratyphi	MDR excluding XDR in TB	Methicillin	PCN	Mono RIF	TMP−SMX	Vanco	XDR in TB
Acinetobacter baumannii	2,120	94.6		665	0.673	657	109	6.09	582										
Citrobacter spp.	79.2	3.09		27.4		15.2	10.1	8.87	14.6										
Enterobacter spp.	465	29.9		57.4		94.9		60.9	94								128		
Enterococcus faecalis	467								432									35.1	
Enterococcus faecium	518								411									107	
Other enterococci	155								140									15.2	
Escherichia coli	3,320	174	345		605	189	855		319								835		
Group A Streptococcus	26.6										26.3								
Group B Streptococcus	737								333		363				22.6				
Haemophilus influenzae	315		247				67.6												
Klebsiella pneumoniae	6,070	615			112	129	3,450		842								918		
Morganella spp.	3.09						0.084	1.25	1.76										
Mycobacterium tuberculosis	7,740									689			6,220			740			87.1
Neisseria gonorrhoeae	3.39						0.042		3.35										
Proteus spp.	123	3.96	19.4				57.9		10.5								31.5		
Pseudomonas aeruginosa	1,710	54.2		466		685	97.8	38	372										
Salmonella Paratyphi	0.249								0.247			0.002							
Salmonella Typhi	672								337			338							
Non−typhoidal Salmonella	2.23								2.23										
Serratia spp.	180	34.2		31.7		52.3	12.8	29.2	19										
Shigella spp.	116								116										
Staphylococcus aureus	3,090								229		536			1,180			973	168	
Streptococcus pneumoniae	4,440				311	1,140	105		407		287				450		1,740		
All pathogens	32,300	1,010	612	1,250	1,030	2,970	4,770	144	4,660	682	1,230	323	6,220	1,180	472	740	4,620	326	87.1

Counts
- <2.5k
- 2.5k − <5k
- 5k − <10k
- 25k − <75k

Liberia

DALYs (count) *associated with* bacterial antimicrobial resistance by pathogen−drug combinations, 2019

	Resistance to 1+	AG	Aminopenicillin	Anti−pseudomonal	BL−BLI	CP	3GC	4GC	FQ	Mono INH	Macrolide	MDR in S. Typhi and Paratyphi	MDR excluding XDR in TB	Methicillin	PCN	Mono RIF	TMP−SMX	Vanco	XDR in TB
Acinetobacter baumannii	7,700	3,230		7,640	3,890	2,170	5,000	2,690	2,800										
Citrobacter spp.	1,270	334		517		183	1,150	1,010	439										
Enterobacter spp.	5,930	2,900		2,090		840		5,190	3,860								4,310		
Enterococcus faecalis	3,370								3,270									281	
Enterococcus faecium	3,830								3,810									897	
Other enterococci	1,750								1,740									106	
Escherichia coli	28,200	9,660	27,500		22,900	2,880	12,500		13,900								24,200		
Group A Streptococcus	784										784								
Group B Streptococcus	7,380								2,670		6,160				250				
Haemophilus influenzae	1,540		1,170				645												
Klebsiella pneumoniae	38,000	27,800			35,300	699	30,400		28,300								35,300		
Morganella spp.	20.2						5.65	12.2	14.5										
Mycobacterium tuberculosis	2,750									814			1,880			22.6			25.4
Neisseria gonorrhoeae	46.2						0.109		46.1										
Proteus spp.	1,710	450	1,440				444		546								1,380		
Pseudomonas aeruginosa	11,500	6,790		5,960		4,090	7,160	4,620	5,310										
Salmonella Paratyphi	1.13								0.591			0.542							
Salmonella Typhi	14,000								1,590			12,900							
Non−typhoidal Salmonella	4.39								4.39										
Serratia spp.	4,250	1,790		601		201	3,660	3,700	975										
Shigella spp.	120								120										
Staphylococcus aureus	22,500								4,400		10,400			7,030			16,100	243	
Streptococcus pneumoniae	30,300				5,570	7,060	3,700		2,660		5,940				3,500		29,500		
All pathogens	187,000	53,000	30,100	16,800	67,600	18,100	64,700	17,200	76,400	814	23,300	12,900	1,880	7,030	3,750	22.6	111,000	1,530	25.4

Counts
- <2.5k
- 2.5k − <5k
- 5k − <10k
- 10k − <25k
- 25k − <75k
- 75k − <150k
- >=150k

Liberia

DALYs (count) *attributable to* bacterial antimicrobial resistance by pathogen−drug combinations, 2019

	Resistance to 1+	AG	Aminopenicillin	Anti−pseudomonal	BL−BLI	CP	3GC	4GC	FQ	Mono INH	Macrolide	MDR in S. Typhi and Paratyphi	MDR excluding XDR in TB	Methicillin	PCN	Mono RIF	TMP−SMX	Vanco	XDR in TB
Acinetobacter baumannii	2,180	172		1,260	0.056	383	1.52	0.172	362										
Citrobacter spp.	376	18		95.2		41.8	49.2	117	54.7										
Enterobacter spp.	1,430	150		165		165		396	370								187		
Enterococcus faecalis	898								816									82.5	
Enterococcus faecium	953								741									212	
Other enterococci	360								339									21.4	
Escherichia coli	6,820	575	354		1,040	592	1,510		1,310								1,440		
Group A Streptococcus	73.4										76.3								
Group B Streptococcus	1,100								452		584				47.6				
Haemophilus influenzae	355		168				187												
Klebsiella pneumoniae	9,850	1,930			564	259	3,860		1,700								1,540		
Morganella spp.	4.74						0.288	2.1	2.35										
Mycobacterium tuberculosis	1,200									125			1,060			5.95			15
Neisseria gonorrhoeae	4.57						0.052		4.51										
Proteus spp.	233	21.2	26.3				92		38.2								54.2		
Pseudomonas aeruginosa	2,710	199		718		694	421	105	574										
Salmonella Paratyphi	0.199								0.125			0.075							
Salmonella Typhi	1,960								290			1,650							
Non−typhoidal Salmonella	0.891								0.891										
Serratia spp.	1,140	104		130		58.8	123	625	98.3										
Shigella spp.	24.6								24.6										
Staphylococcus aureus	4,140								179		405			1,610			1,880	74	
Streptococcus pneumoniae	5,450				158	1,560	127		316		224				66.8		3,000		
All pathogens	41,300	3,170	549	2,370	1,770	3,750	6,360	1,250	7,670	108	1,300	1,640	1,060	1,610	114	5.95	8,100	390	15

Counts
- <2.5k
- 2.5k − <5k
- 5k − <10k
- 25k − <75k

Madagascar

DALYs (count) *associated with* bacterial antimicrobial resistance by pathogen−drug combinations, 2019

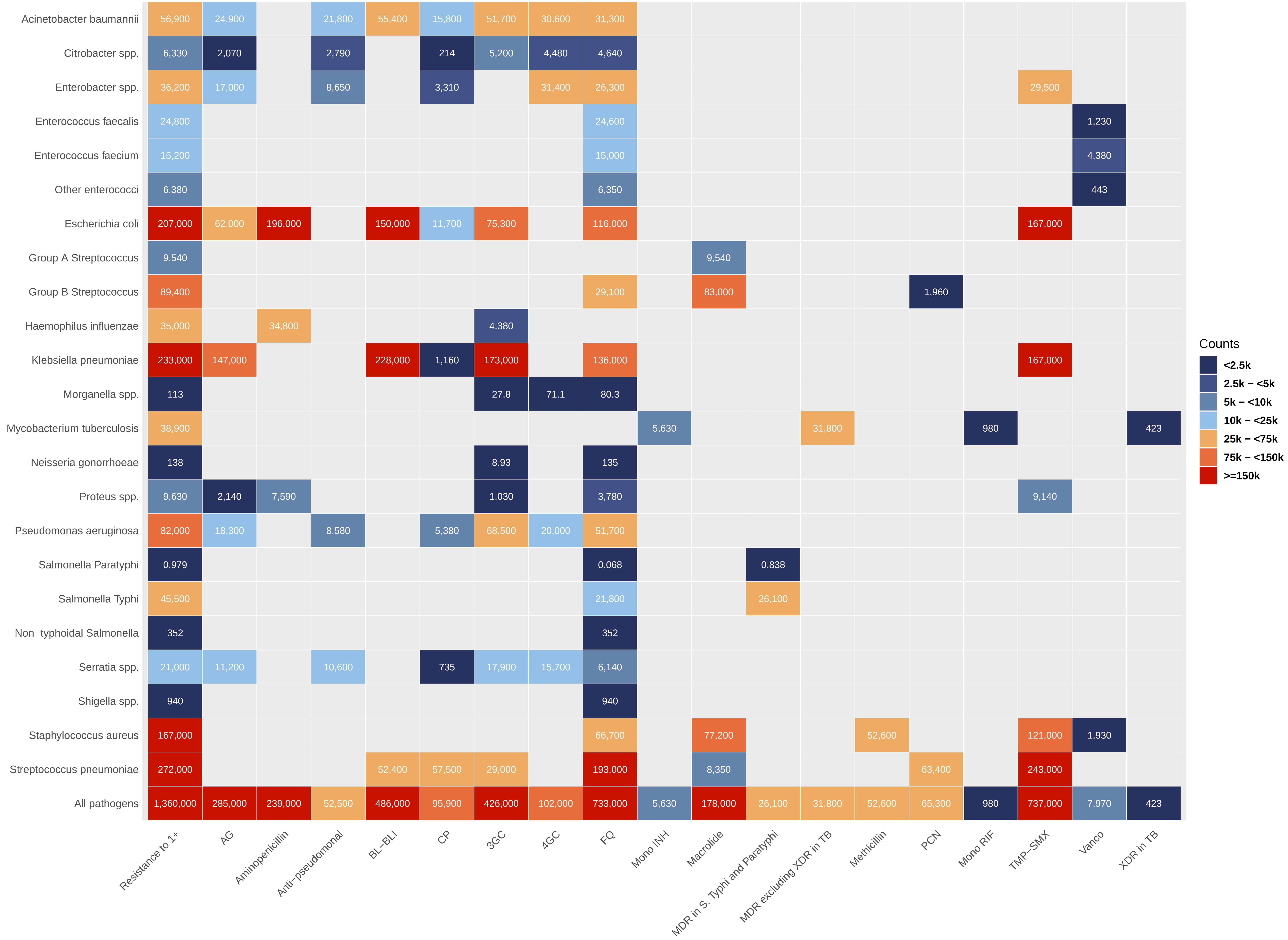

Madagascar

DALYs (count) *attributable to* bacterial antimicrobial resistance by pathogen−drug combinations, 2019

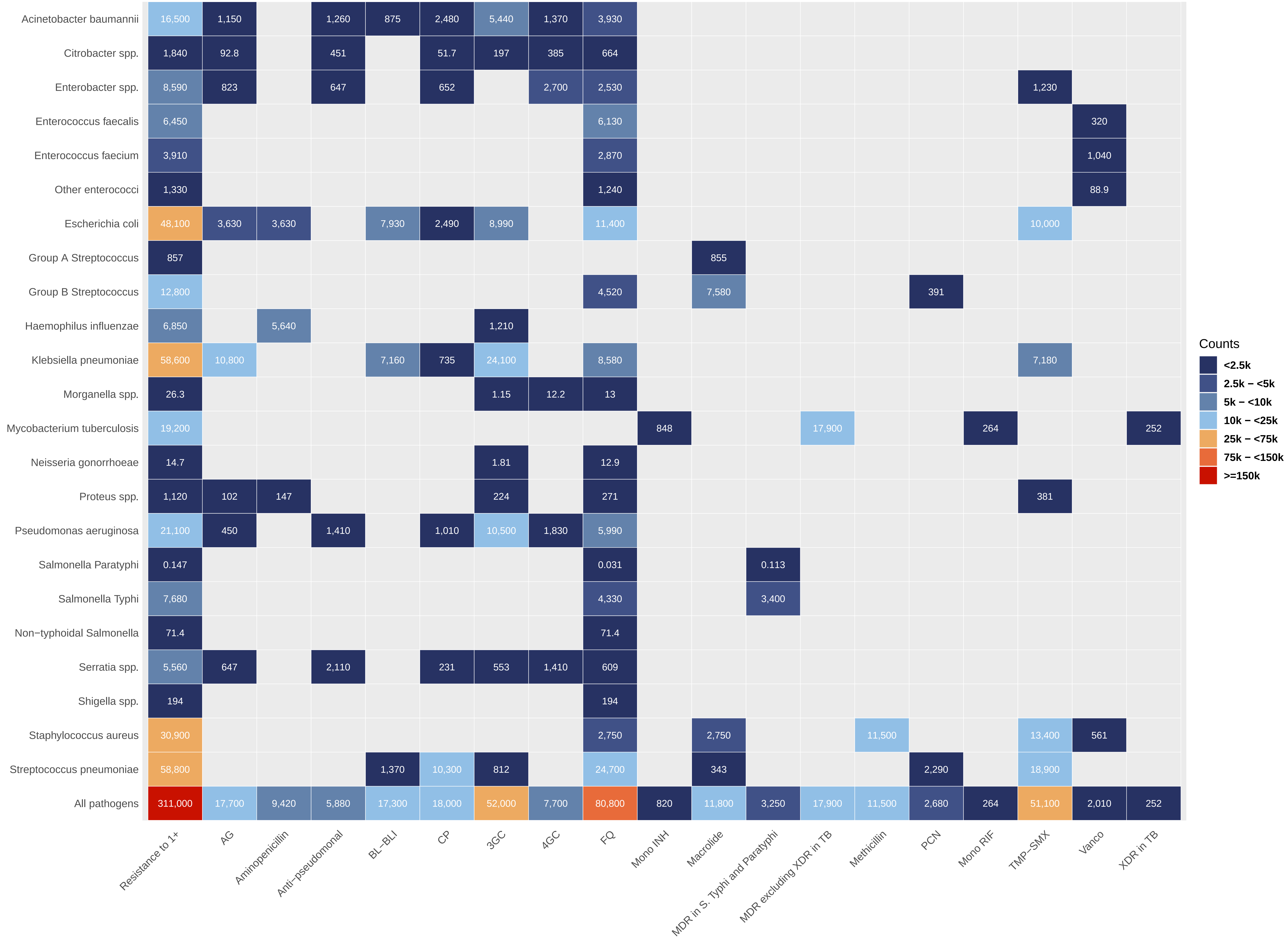

Malawi

DALYs (count) *associated with* bacterial antimicrobial resistance by pathogen−drug combinations, 2019

	Resistance to 1+	AG	Aminopenicillin	Anti−pseudomonal	BL−BLI	CP	3GC	4GC	FQ	Mono INH	Macrolide	MDR in S. Typhi and Paratyphi	MDR excluding XDR in TB	Methicillin	PCN	Mono RIF	TMP−SMX	Vanco	XDR in TB
Acinetobacter baumannii	34,000	11,400		28,200	11,400	7,810	33,200	11,900	17,200										
Citrobacter spp.	4,870	1,290		2,040		258	4,850	2,950	993										
Enterobacter spp.	23,600	13,400		9,320		2,750		21,000	15,200								17,100		
Enterococcus faecalis	18,000								17,800									940	
Enterococcus faecium	12,900								12,700									3,500	
Other enterococci	5,740								5,720									314	
Escherichia coli	130,000	33,400	123,000		114,000	18,100	47,000		44,000								120,000		
Group A Streptococcus	6,850										6,850								
Group B Streptococcus	32,800								17,500		22,900				722				
Haemophilus influenzae	19,000		18,700				2,590												
Klebsiella pneumoniae	169,000	123,000			146,000	1,880	146,000		78,500								156,000		
Morganella spp.	87.8						21.4	53.5	63.8										
Mycobacterium tuberculosis	21,900									4,750			14,200			2,700			190
Neisseria gonorrhoeae	24.9						3.14		22.8										
Proteus spp.	6,850	3,610	6,030				2,410		4,310								4,750		
Pseudomonas aeruginosa	44,000	15,500		16,400		12,400	34,700	15,000	20,800										
Salmonella Paratyphi	0.629								0.12			0.501							
Salmonella Typhi	69,500								15,700			67,300							
Non−typhoidal Salmonella	26.2								26.2										
Serratia spp.	11,300	4,900		4,290		672	2,270	8,990	2,290										
Shigella spp.	1,020								1,020										
Staphylococcus aureus	107,000								25,300		55,000			34,900			73,200	1,350	
Streptococcus pneumoniae	186,000				35,600	41,400	12,200		27,300		18,700				57,900		184,000		
All pathogens	904,000	206,000	147,000	60,200	307,000	85,300	285,000	59,900	307,000	4,750	104,000	67,300	14,200	34,900	58,600	2,700	555,000	6,110	190

Counts
- <2.5k
- 2.5k − <5k
- 5k − <10k
- 10k − <25k
- 25k − <75k
- 75k − <150k
- >=150k

Malawi

DALYs (count) *attributable to* bacterial antimicrobial resistance by pathogen−drug combinations, 2019

	Resistance to 1+	AG	Aminopenicillin	Anti−pseudomonal	BL−BLI	CP	3GC	4GC	FQ	Mono INH	Macrolide	MDR in S. Typhi and Paratyphi	MDR excluding XDR in TB	Methicillin	PCN	Mono RIF	TMP−SMX	Vanco	XDR in TB
Acinetobacter baumannii	10,200	557		4,190	0.511	1,340	1,840	1.26	2,290										
Citrobacter spp.	1,450	74.6		434		63.5	408	347	119										
Enterobacter spp.	5,670	710		774		543		1,530	1,400								730		
Enterococcus faecalis	4,690								4,450									247	
Enterococcus faecium	3,280								2,440									834	
Other enterococci	1,190								1,120									64.7	
Escherichia coli	30,500	2,100	883		5,620	4,030	5,540		4,200								8,080		
Group A Streptococcus	653										640								
Group B Streptococcus	5,400								3,090		2,080				165				
Haemophilus influenzae	3,760		3,040				712												
Klebsiella pneumoniae	44,500	9,130			1,450	838	20,800		4,770								7,500		
Morganella spp.	20.5						0.939	9.12	10.4										
Mycobacterium tuberculosis	9,740									715			8,180			730			114
Neisseria gonorrhoeae	2.89						0.717		2.17										
Proteus spp.	1,110	161	88.6				436		278								149		
Pseudomonas aeruginosa	11,200	470		2,080		2,130	3,620	582	2,390										
Salmonella Paratyphi	0.097								0.029			0.067							
Salmonella Typhi	10,400								2,310			8,340							
Non−typhoidal Salmonella	5.42								5.42										
Serratia spp.	2,370	259		749		211	2.64	926	217										
Shigella spp.	209								209										
Staphylococcus aureus	19,800								1,030		2,120			7,920			8,350	384	
Streptococcus pneumoniae	34,800				1,220	8,890	162		2,990		683				3,060		17,800		
All pathogens	201,000	13,500	4,020	8,230	8,300	18,000	33,600	3,390	33,300	694	5,600	8,000	8,180	7,920	3,220	730	42,600	1,530	114

Counts
- <2.5k
- 2.5k − <5k
- 5k − <10k
- 10k − <25k
- 25k − <75k
- >=150k

Mali

DALYs (count) *associated with* bacterial antimicrobial resistance by pathogen−drug combinations, 2019

	Resistance to 1+	AG	Aminopenicillin	Anti−pseudomonal	BL−BLI	CP	3GC	4GC	FQ	Mono INH	Macrolide	MDR in S. Typhi and Paratyphi	MDR excluding XDR in TB	Methicillin	PCN	Mono RIF	TMP−SMX	Vanco	XDR in TB
Acinetobacter baumannii	70,300	31,100		69,700	38,600	26,900	44,900	44,000	34,600										
Citrobacter spp.	16,800	7,650		7,760		6,990	15,000	14,900	7,100										
Enterobacter spp.	69,100	31,900		27,400		14,600		59,200	44,400								56,500		
Enterococcus faecalis	37,200								36,700									2,520	
Enterococcus faecium	28,200								28,000									6,640	
Other enterococci	21,900								21,900									992	
Escherichia coli	306,000	104,000	291,000		231,000	57,100	149,000		194,000								292,000		
Group A Streptococcus	7,810										7,810								
Group B Streptococcus	152,000								109,000		92,600				3,130				
Haemophilus influenzae	29,800		19,300				17,000												
Klebsiella pneumoniae	484,000	349,000			451,000	16,300	433,000		362,000								461,000		
Morganella spp.	144						36.5	87.2	108										
Mycobacterium tuberculosis	22,400									6,760			15,200			215			204
Neisseria gonorrhoeae	215						0.422		214										
Proteus spp.	13,900	4,880	12,100				3,510		7,600								11,000		
Pseudomonas aeruginosa	113,000	55,000		63,700		43,200	63,700	56,700	65,400										
Salmonella Paratyphi	22.6								11.5			11.4							
Salmonella Typhi	145,000								26,400			130,000							
Non−typhoidal Salmonella	148								148										
Serratia spp.	46,000	25,800		6,460		2,250	36,400	39,700	13,300										
Shigella spp.	2,260								2,260										
Staphylococcus aureus	243,000								43,900		114,000			122,000			161,000	8,850	
Streptococcus pneumoniae	336,000				84,200	117,000	52,300		39,000		123,000				40,600		271,000		
All pathogens	2,150,000	608,000	323,000	175,000	805,000	285,000	816,000	214,000	1,040,000	6,760	337,000	130,000	15,200	122,000	43,700	215	1,250,000	19,000	204

Counts
- <2.5k
- 2.5k − <5k
- 5k − <10k
- 10k − <25k
- 25k − <75k
- 75k − <150k
- >=150k

Mali

DALYs (count) *attributable to* bacterial antimicrobial resistance by pathogen−drug combinations, 2019

	Resistance to 1+	AG	Aminopenicillin	Anti−pseudomonal	BL−BLI	CP	3GC	4GC	FQ	Mono INH	Macrolide	MDR in S. Typhi and Paratyphi	MDR excluding XDR in TB	Methicillin	PCN	Mono RIF	TMP−SMX	Vanco	XDR in TB
Acinetobacter baumannii	20,400	1,560		9,650	1.06	4,690	21.4	1.57	4,510										
Citrobacter spp.	5,140	417		926		1,520	317	1,130	843										
Enterobacter spp.	16,900	1,640		1,980		2,800		3,790	4,210								2,570		
Enterococcus faecalis	9,820								9,150									668	
Enterococcus faecium	7,000								5,440									1,560	
Other enterococci	4,470								4,270									199	
Escherichia coli	78,700	5,910	3,460		7,580	11,100	15,100		18,400								17,100		
Group A Streptococcus	763										724								
Group B Streptococcus	27,600								19,200		7,590				616				
Haemophilus influenzae	7,380		2,470				4,920												
Klebsiella pneumoniae	131,000	24,200			3,770	4,720	55,900		21,900								20,300		
Morganella spp.	33.7						1.59	14.5	17.6										
Mycobacterium tuberculosis	9,890									1,050			8,700			58.3			122
Neisseria gonorrhoeae	21						0.203		20.7										
Proteus spp.	1,990	207	213				683		498								407		
Pseudomonas aeruginosa	27,300	1,490		7,570		7,130	2,300	1,520	7,260										
Salmonella Paratyphi	3.92								2.39			1.56							
Salmonella Typhi	20,700								4,630			16,300							
Non−typhoidal Salmonella	31.4								31.4										
Serratia spp.	11,900	1,420		1,320		644	1,050	6,180	1,290										
Shigella spp.	464								464										
Staphylococcus aureus	54,900								1,830		4,200			29,200			17,500	2,240	
Streptococcus pneumoniae	66,600				2,220	26,500	1,130		5,260		4,960				358		26,100		
All pathogens	503,000	36,900	6,130	21,400	13,500	59,100	81,400	12,600	109,000	957	17,800	16,100	8,700	29,200	974	58.3	84,000	4,680	122

Counts
- <2.5k
- 2.5k − <5k
- 5k − <10k
- 10k − <25k
- 25k − <75k
- 75k − <150k
- >=150k

Mauritania

DALYs (count) *associated with* bacterial antimicrobial resistance by pathogen−drug combinations, 2019

	Resistance to 1+	AG	Aminopenicillin	Anti−pseudomonal	BL−BLI	CP	3GC	4GC	FQ	Mono INH	Macrolide	MDR in S. Typhi and Paratyphi	MDR excluding XDR in TB	Methicillin	PCN	Mono RIF	TMP−SMX	Vanco	XDR in TB
Acinetobacter baumannii	5,470	2,980		5,440	3,070	2,580	3,390	2,970	3,110										
Citrobacter spp.	941	296		329		152	830	776	363										
Enterobacter spp.	5,310	1,970		2,040		986		4,030	3,250								4,330		
Enterococcus faecalis	2,340								2,290									175	
Enterococcus faecium	2,600								2,580									626	
Other enterococci	1,550								1,550									96.2	
Escherichia coli	19,500	8,940	18,900		14,900	1,860	8,300		7,690								17,300		
Group A Streptococcus	434										434								
Group B Streptococcus	7,030								3,310		5,390				203				
Haemophilus influenzae	934		647				447												
Klebsiella pneumoniae	23,700	13,500			21,900	1,010	18,900		16,300								22,000		
Morganella spp.	13.8						3.37	3.84	12.3										
Mycobacterium tuberculosis	1,310									417			866			11.5			11.5
Neisseria gonorrhoeae	40.6						0.078		40.5										
Proteus spp.	1,120	636	916				286		595								897		
Pseudomonas aeruginosa	8,460	4,550		4,140		3,510	4,520	2,930	5,380										
Salmonella Paratyphi	2.33								1.96			0.386							
Salmonella Typhi	5,310								1,290			4,380							
Non−typhoidal Salmonella	3.99								3.99										
Serratia spp.	2,510	1,230		388		189	1,950	1,980	755										
Shigella spp.	506								506										
Staphylococcus aureus	15,700								3,220		9,060			9,150			8,380	616	
Streptococcus pneumoniae	20,400				3,550	6,060	2,650		1,440		6,020				2,770		19,600		
All pathogens	125,000	34,100	20,400	12,300	43,400	16,300	41,300	12,700	53,700	417	20,900	4,380	866	9,150	2,970	11.5	72,500	1,510	11.5

Counts
- <2.5k
- 2.5k − <5k
- 5k − <10k
- 10k − <25k
- 25k − <75k
- 75k − <150k

Mauritania

DALYs (count) *attributable to* bacterial antimicrobial resistance by pathogen−drug combinations, 2019

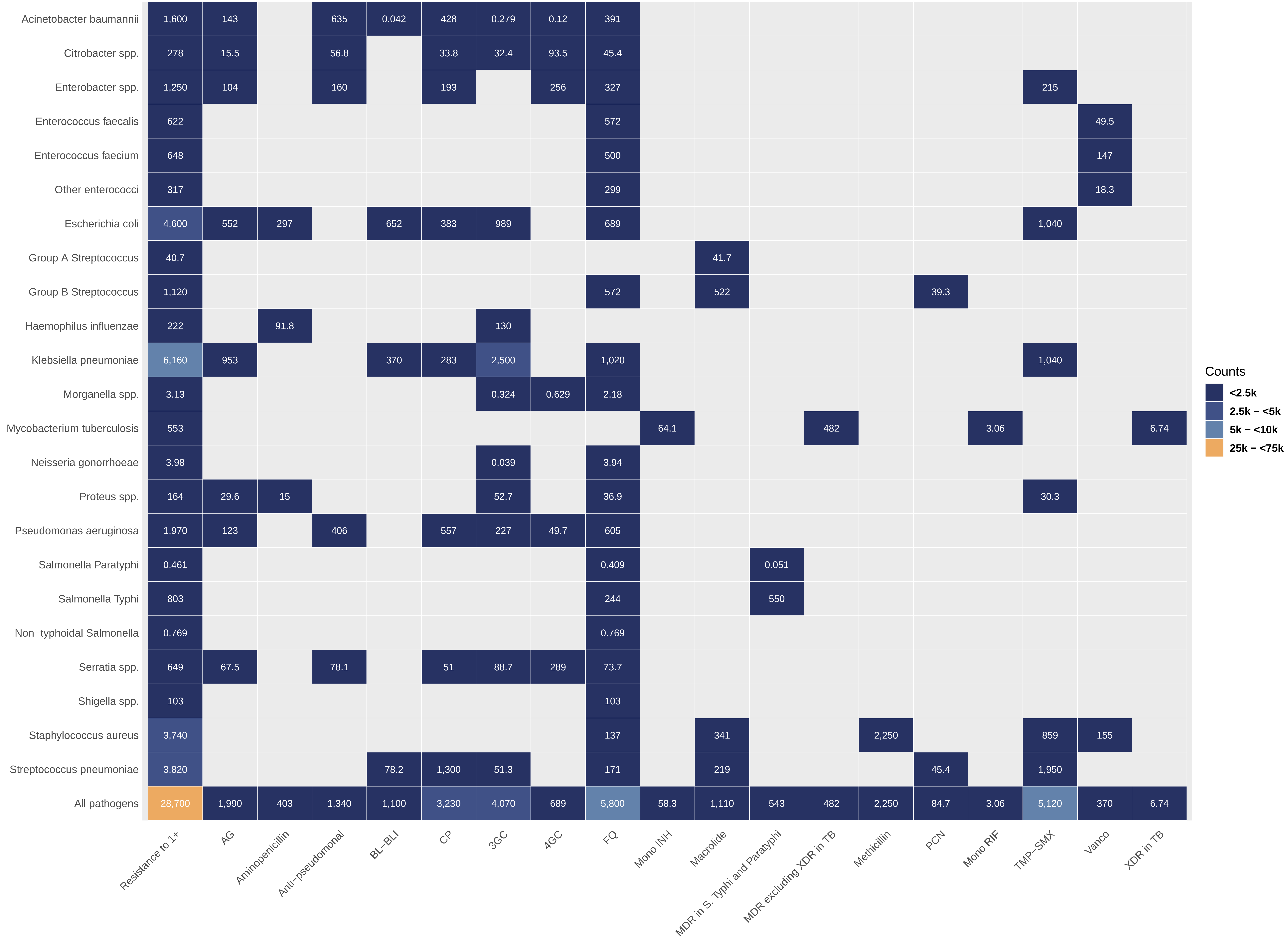

Mauritius

DALYs (count) *associated with* bacterial antimicrobial resistance by pathogen−drug combinations, 2019

	Resistance to 1+	AG	Aminopenicillin	Anti−pseudomonal	BL−BLI	CP	3GC	4GC	FQ	Mono INH	Macrolide	MDR in S. Typhi and Paratyphi	MDR excluding XDR in TB	Methicillin	PCN	Mono RIF	TMP−SMX	Vanco	XDR in TB
Acinetobacter baumannii	1,860	1,430		1,790	1,440	1,460	1,680	1,770	1,560										
Citrobacter spp.	123	12.8		15.8		22	76.1	73.3	52.2										
Enterobacter spp.	660	101		327		177		273	169								413		
Enterococcus faecalis	514								489									65.7	
Enterococcus faecium	1,110								1,110									261	
Other enterococci	451								448									158	
Escherichia coli	3,380	436	3,230		2,550	455	1,620		1,990								2,300		
Group A Streptococcus	187										187								
Group B Streptococcus	398								108		333				35.6				
Haemophilus influenzae	88.9		81				19.7												
Klebsiella pneumoniae	2,510	317			1,800	470	1,940		1,260								1,890		
Morganella spp.	7.76						4.61	1.72	4.61										
Mycobacterium tuberculosis	25.9									18.4			5.79			0.652			1.01
Neisseria gonorrhoeae	10.6						0.082		10.6										
Proteus spp.	402	294	320				153		159								228		
Pseudomonas aeruginosa	1,400	412		530		990	718	534	620										
Salmonella Paratyphi	26.3								25.8			0.548							
Salmonella Typhi	36.1								33.2			3.16							
Non−typhoidal Salmonella	0.5								0.5										
Serratia spp.	132	19.4		43.6		30.2	94.3	39.2	40.3										
Shigella spp.	3.11								3.11										
Staphylococcus aureus	3,980								1,100		2,100			3,650			1,050	64.1	
Streptococcus pneumoniae	1,910				323	990	378		84.3		1,160				667		1,390		
All pathogens	19,200	3,030	3,630	2,700	6,110	4,590	6,680	2,690	9,270	18.4	3,770	3.71	5.79	3,650	702	0.652	7,280	548	1.01

Counts
- **<2.5k**
- **2.5k − <5k**
- **5k − <10k**
- **10k − <25k**

Mauritius

DALYs (count) *attributable to* bacterial antimicrobial resistance by pathogen−drug combinations, 2019

	Resistance to 1+	AG	Aminopenicillin	Anti−pseudomonal	BL−BLI	CP	3GC	4GC	FQ	Mono INH	Macrolide	MDR in S. Typhi and Paratyphi	MDR excluding XDR in TB	Methicillin	PCN	Mono RIF	TMP−SMX	Vanco	XDR in TB
Acinetobacter baumannii	587	62.9		73.8	0.307	238	9.99	7.57	194										
Citrobacter spp.	34.4	0.669		3.09		5.26	7.57	9.75	8.12										
Enterobacter spp.	146	6.76		37.2		45.6		11.2	18								27		
Enterococcus faecalis	140								120									19.8	
Enterococcus faecium	277								216									61.3	
Other enterococci	103								76.8									26.5	
Escherichia coli	851	26.4	48.3		123	101	206		211								135		
Group A Streptococcus	18.2										17								
Group B Streptococcus	58.2								18.6		32.6				6.51				
Haemophilus influenzae	18.6		12.9				5.65												
Klebsiella pneumoniae	682	32.4			34.4	122	283		98.3								112		
Morganella spp.	1.98						0.822	0.381	0.78										
Mycobacterium tuberculosis	5.76									2.33			2.73			0.157			0.539
Neisseria gonorrhoeae	1.05						0.022		1.02										
Proteus spp.	64.8	15.9	4.25				28		9.37								7.14		
Pseudomonas aeruginosa	345	11.4		28.1		188	39.8	12.4	65.5										
Salmonella Paratyphi	5.38								5.3			0.085							
Salmonella Typhi	7.3								6.88			0.416							
Non−typhoidal Salmonella	0.089								0.089										
Serratia spp.	35.1	1.12		9.13		8.31	8.46	3.33	4.69										
Shigella spp.	0.511								0.511										
Staphylococcus aureus	1,220								49.4		72.2			992			87.9	14.1	
Streptococcus pneumoniae	423				0.983	226	1.97		9.28		44.8				18.5		120		
All pathogens	5,020	157	65.7	151	159	935	592	44.6	1,110	2.37	170	0.508	2.73	992	25.1	0.157	489	122	0.539

Counts
- <2.5k
- 5k − <10k

Mozambique

DALYs (count) *associated with* bacterial antimicrobial resistance by pathogen−drug combinations, 2019

	Resistance to 1+	AG	Aminopenicillin	Anti−pseudomonal	BL−BLI	CP	3GC	4GC	FQ	Mono INH	Macrolide	MDR in S. Typhi and Paratyphi	MDR excluding XDR in TB	Methicillin	PCN	Mono RIF	TMP−SMX	Vanco	XDR in TB
Acinetobacter baumannii	69,200	20,400		59,300	44,300	11,300	56,200	27,300	24,600										
Citrobacter spp.	9,600	3,130		4,530		625	8,380	6,830	5,490										
Enterobacter spp.	45,700	22,100		16,600		5,490		41,600	27,900								34,600		
Enterococcus faecalis	35,100								34,600									2,640	
Enterococcus faecium	24,000								23,700									6,140	
Other enterococci	11,100								11,000									689	
Escherichia coli	213,000	60,100	196,000		145,000	15,300	71,900		76,000								168,000		
Group A Streptococcus	16,000										16,000								
Group B Streptococcus	82,200								34,800		69,300				2,320				
Haemophilus influenzae	39,100		38,500				6,570												
Klebsiella pneumoniae	362,000	264,000			327,000	1,170	334,000		247,000								352,000		
Morganella spp.	191						44.5	120	139										
Mycobacterium tuberculosis	119,000									9,630			97,900			9,860			1,320
Neisseria gonorrhoeae	173						1.59		172										
Proteus spp.	13,800	3,850	11,500				2,740		5,430								12,300		
Pseudomonas aeruginosa	82,000	22,500		32,100		20,300	70,000	32,400	29,300										
Salmonella Paratyphi	3.01								0.125			2.68							
Salmonella Typhi	176,000								38,100			159,000							
Non−typhoidal Salmonella	1,160								1,160										
Serratia spp.	29,900	14,800		11,700		1,550	17,900	24,400	7,410										
Shigella spp.	2,600								2,600										
Staphylococcus aureus	235,000								64,000		39,100			101,000			201,000	2,850	
Streptococcus pneumoniae	337,000				63,000	72,500	41,300		160,000		34,300				64,900		313,000		
All pathogens	1,900,000	411,000	246,000	124,000	579,000	128,000	609,000	133,000	794,000	9,630	159,000	159,000	97,900	101,000	67,200	9,860	1,080,000	12,300	1,320

Counts
- <2.5k
- 2.5k − <5k
- 5k − <10k
- 10k − <25k
- 25k − <75k
- 75k − <150k
- >=150k

Mozambique

DALYs (count) *attributable to* bacterial antimicrobial resistance by pathogen−drug combinations, 2019

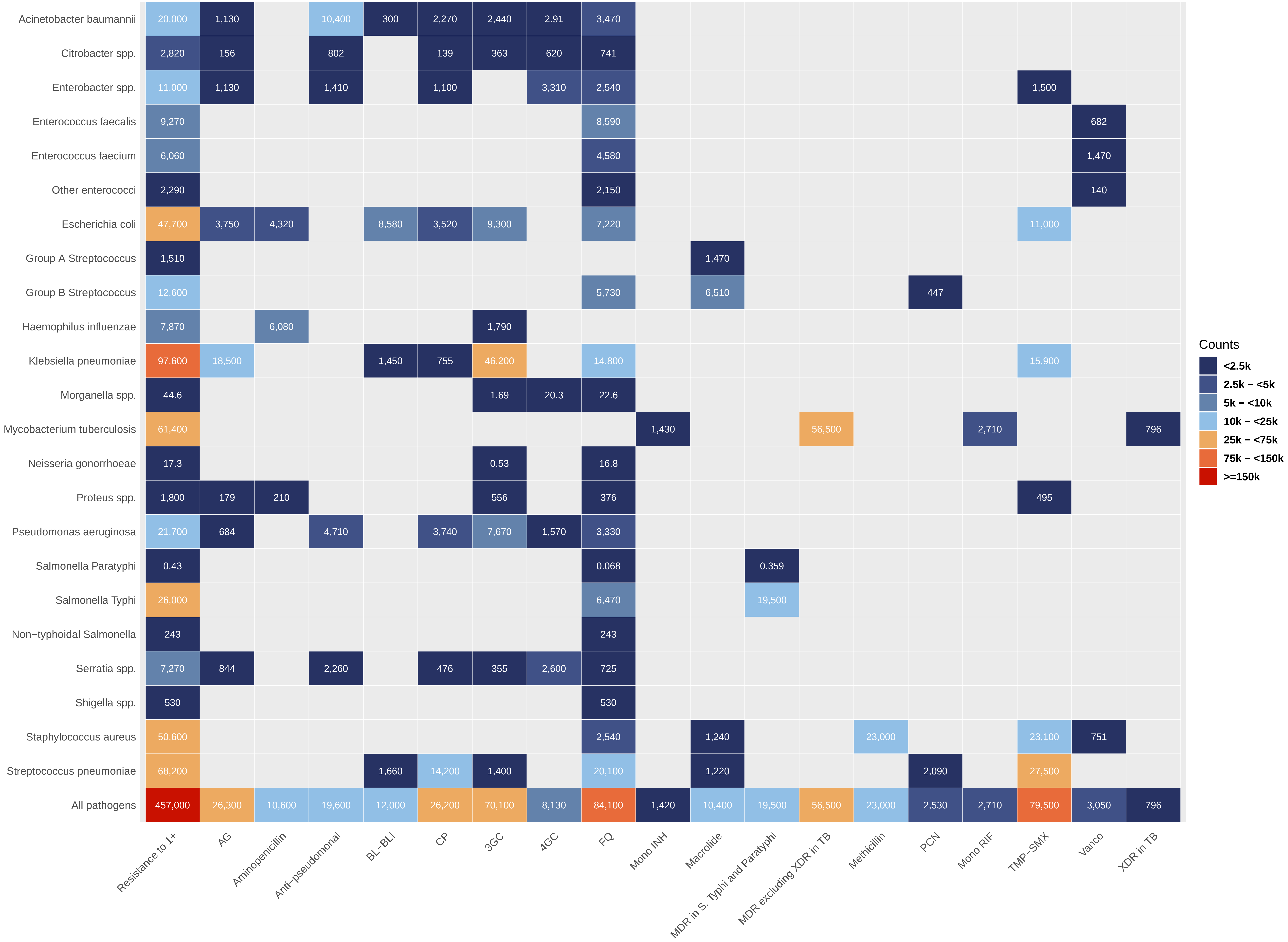

Namibia

DALYs (count) *associated with* bacterial antimicrobial resistance by pathogen−drug combinations, 2019

	Resistance to 1+	AG	Aminopenicillin	Anti−pseudomonal	BL−BLI	CP	3GC	4GC	FQ	Mono INH	Macrolide	MDR in S. Typhi and Paratyphi	MDR excluding XDR in TB	Methicillin	PCN	Mono RIF	TMP−SMX	Vanco	XDR in TB
Acinetobacter baumannii	4,020	1,950		3,900	2,720	2,740	3,690	2,910	3,460										
Citrobacter spp.	279	17.3		95		41.2	183	144	109										
Enterobacter spp.	2,090	224		543		425		587	471								1,880		
Enterococcus faecalis	879								837									85.9	
Enterococcus faecium	1,870								1,860									341	
Other enterococci	791								789									45.2	
Escherichia coli	9,870	899	9,380		5,720	464	3,920		2,730								8,510		
Group A Streptococcus	117										117								
Group B Streptococcus	2,350								252		2,280				107				
Haemophilus influenzae	638		566				156												
Klebsiella pneumoniae	13,500	3,500			12,200	126	9,370		5,860								11,900		
Morganella spp.	8.72						1.17	1.71	8.04										
Mycobacterium tuberculosis	7,770									2,770			4,360			587			57.5
Neisseria gonorrhoeae	32.2						0.043		32.1										
Proteus spp.	732	39.3	646				149		165								569		
Pseudomonas aeruginosa	5,070	1,320		2,910		3,320	2,330	1,810	2,860										
Salmonella Paratyphi	1.23								1.22			0.012							
Salmonella Typhi	1,080								420			740							
Non−typhoidal Salmonella	8.56								8.56										
Serratia spp.	675	415		130		85.8	256	339	126										
Shigella spp.	345								345										
Staphylococcus aureus	11,200								4,560		5,390			3,910			6,980	152	
Streptococcus pneumoniae	13,100				3,200	4,010	2,150		932		4,260				6,090		11,300		
All pathogens	76,400	8,350	10,600	7,580	23,800	11,200	22,200	5,790	25,800	2,770	12,000	740	4,360	3,910	6,200	587	41,200	624	57.5

Counts
- <2.5k
- 2.5k − <5k
- 5k − <10k
- 10k − <25k
- 25k − <75k
- 75k − <150k

Namibia

DALYs (count) *attributable to* bacterial antimicrobial resistance by pathogen−drug combinations, 2019

	Resistance to 1+	AG	Aminopenicillin	Anti−pseudomonal	BL−BLI	CP	3GC	4GC	FQ	Mono INH	Macrolide	MDR in S. Typhi and Paratyphi	MDR excluding XDR in TB	Methicillin	PCN	Mono RIF	TMP−SMX	Vanco	XDR in TB
Acinetobacter baumannii	1,260	83.5		244	0.118	445	31	0.147	455										
Citrobacter spp.	78.8	0.939		19		10.2	15.1	15.9	17.6										
Enterobacter spp.	384	17.5		48.8		102		24	56.1								137		
Enterococcus faecalis	237								209									28	
Enterococcus faecium	451								370									81.4	
Other enterococci	162								153									9.04	
Escherichia coli	2,200	60.7	287		307	109	580		271								589		
Group A Streptococcus	11.4										11.2								
Group B Streptococcus	288								39.3		233				19.6				
Haemophilus influenzae	136		90.6				45.1												
Klebsiella pneumoniae	3,260	279			363	61.6	1,490		409								657		
Morganella spp.	1.9						0.12	0.279	1.5										
Mycobacterium tuberculosis	2,970									374			2,380			154			33.7
Neisseria gonorrhoeae	3.15						0.028		3.12										
Proteus spp.	87.9	2.55	13.9				34.3		12.8								24.6		
Pseudomonas aeruginosa	1,240	33.1		223		593	57.6	17.3	311										
Salmonella Paratyphi	0.251								0.249			0.002							
Salmonella Typhi	176								82.4			90.7							
Non−typhoidal Salmonella	1.56								1.56										
Serratia spp.	137	25.3		24.3		22.9	12.7	38.4	12.9										
Shigella spp.	70.5								70.5										
Staphylococcus aureus	2,120								198		203			894			776	44.2	
Streptococcus pneumoniae	2,700				85.7	866	61.8		107		154				382		1,050		
All pathogens	18,000	503	392	559	756	2,210	2,330	96.1	2,780	364	602	92.8	2,380	894	402	154	3,230	163	33.7

Counts
- <2.5k
- 2.5k − <5k
- 10k − <25k

Niger

DALYs (count) *associated with* bacterial antimicrobial resistance by pathogen−drug combinations, 2019

	Resistance to 1+	AG	Aminopenicillin	Anti−pseudomonal	BL−BLI	CP	3GC	4GC	FQ	Mono INH	Macrolide	MDR in S. Typhi and Paratyphi	MDR excluding XDR in TB	Methicillin	PCN	Mono RIF	TMP−SMX	Vanco	XDR in TB
Acinetobacter baumannii	54,700	22,400		54,200	30,400	18,900	33,700	24,300	18,300										
Citrobacter spp.	12,600	5,540		6,010		4,840	11,500	11,300	4,710										
Enterobacter spp.	66,100	28,000		23,100		13,900		58,200	44,700								50,900		
Enterococcus faecalis	22,900								22,400									1,820	
Enterococcus faecium	20,300								20,100									5,630	
Other enterococci	12,700								12,600									696	
Escherichia coli	325,000	98,700	316,000		250,000	35,900	137,000		111,000								287,000		
Group A Streptococcus	6,120										6,120								
Group B Streptococcus	178,000								114,000		119,000				5,920				
Haemophilus influenzae	48,300		38,000				21,900												
Klebsiella pneumoniae	455,000	333,000			427,000	12,200	358,000		324,000								422,000		
Morganella spp.	120						29	76.4	86.4										
Mycobacterium tuberculosis	30,600									8,170			15,600			6,610			208
Neisseria gonorrhoeae	205						0.442		205										
Proteus spp.	10,300	3,680	8,730				2,700		6,340								8,240		
Pseudomonas aeruginosa	98,800	47,700		55,000		29,600	60,200	53,800	49,900										
Salmonella Paratyphi	91.8								89.7			2.69							
Salmonella Typhi	72,500								21,000			55,400							
Non−typhoidal Salmonella	66.9								66.9										
Serratia spp.	33,700	19,100		5,140		1,560	28,900	28,400	8,970										
Shigella spp.	1,600								1,600										
Staphylococcus aureus	229,000								26,000		103,000			114,000			154,000	8,400	
Streptococcus pneumoniae	616,000				108,000	131,000	61,000		190,000		149,000				13,900		574,000		
All pathogens	2,300,000	558,000	363,000	143,000	816,000	248,000	715,000	176,000	976,000	8,170	377,000	55,400	15,600	114,000	19,800	6,610	1,500,000	16,500	208

Counts

- <2.5k
- 2.5k − <5k
- 5k − <10k
- 10k − <25k
- 25k − <75k
- 75k − <150k
- >=150k

Niger

DALYs (count) *attributable to* bacterial antimicrobial resistance by pathogen−drug combinations, 2019

	Resistance to 1+	AG	Aminopenicillin	Anti−pseudomonal	BL−BLI	CP	3GC	4GC	FQ	Mono INH	Macrolide	MDR in S. Typhi and Paratyphi	MDR excluding XDR in TB	Methicillin	PCN	Mono RIF	TMP−SMX	Vanco	XDR in TB
Acinetobacter baumannii	15,600	1,210		8,360	1.01	3,590	22.2	1.18	2,410										
Citrobacter spp.	3,870	295		780		1,070	229	932	554										
Enterobacter spp.	16,400	1,400		1,630		2,660		4,160	4,320								2,160		
Enterococcus faecalis	6,080								5,570									511	
Enterococcus faecium	5,160								3,840									1,320	
Other enterococci	2,600								2,460									142	
Escherichia coli	76,700	6,120	5,390		11,600	7,830	17,100		10,300								18,300		
Group A Streptococcus	595										561								
Group B Streptococcus	31,300								19,900		10,600				1,060				
Haemophilus influenzae	11,300		5,060				6,240												
Klebsiella pneumoniae	118,000	23,200			7,620	3,930	44,900		19,400								18,600		
Morganella spp.	28						1.11	12.9	14										
Mycobacterium tuberculosis	12,100									1,220			8,890			1,790			125
Neisseria gonorrhoeae	19.9						0.209		19.7										
Proteus spp.	1,520	155	147				510		423								282		
Pseudomonas aeruginosa	24,000	1,380		7,550		4,920	2,680	2,000	5,470										
Salmonella Paratyphi	18.6								18.3			0.324							
Salmonella Typhi	11,200								4,120			7,010							
Non−typhoidal Salmonella	13.8								13.8										
Serratia spp.	8,960	1,050		1,040		447	1,020	4,470	873										
Shigella spp.	331								331										
Staphylococcus aureus	51,900								1,190		3,830			27,800			16,900	2,180	
Streptococcus pneumoniae	115,000				3,060	26,900	1,710		24,400		5,450				89.1		54,000		
All pathogens	513,000	34,900	10,600	19,400	22,300	51,300	74,500	11,600	106,000	1,230	20,000	7,010	8,890	27,800	1,150	1,790	110,000	4,150	125

Counts
- <2.5k
- 2.5k − <5k
- 5k − <10k
- 10k − <25k
- 25k − <75k
- 75k − <150k
- >=150k

Nigeria

DALYs (count) *associated with* bacterial antimicrobial resistance by pathogen−drug combinations, 2019

	Resistance to 1+	AG	Aminopenicillin	Anti−pseudomonal	BL−BLI	CP	3GC	4GC	FQ	Mono INH	Macrolide	MDR in S. Typhi and Paratyphi	MDR excluding XDR in TB	Methicillin	PCN	Mono RIF	TMP−SMX	Vanco	XDR in TB
Acinetobacter baumannii	396,000	227,000		390,000	186,000	173,000	268,000	244,000	213,000										
Citrobacter spp.	105,000	36,700		43,600		16,000	95,400	84,400	41,000										
Enterobacter spp.	615,000	384,000		262,000		90,500		505,000	373,000								520,000		
Enterococcus faecalis	214,000								212,000									10,900	
Enterococcus faecium	186,000								185,000									45,700	
Other enterococci	128,000								128,000									5,340	
Escherichia coli	2,630,000	1,220,000	2,500,000		2,070,000	305,000	1,970,000		1,880,000								2,490,000		
Group A Streptococcus	43,300										43,300								
Group B Streptococcus	1,330,000								883,000		839,000				15,400				
Haemophilus influenzae	216,000		152,000				103,000												
Klebsiella pneumoniae	3,150,000	2,310,000			2,960,000	768,000	2,870,000		2,500,000								3,040,000		
Morganella spp.	897						214	501	693										
Mycobacterium tuberculosis	294,000									102,000			163,000			26,700			2,160
Neisseria gonorrhoeae	1,880						2.06		1,880										
Proteus spp.	83,800	20,500	74,300				21,500		32,800								65,700		
Pseudomonas aeruginosa	1,060,000	667,000		491,000		523,000	703,000	544,000	602,000										
Salmonella Paratyphi	4,820								4,070			925							
Salmonella Typhi	787,000								300,000			751,000							
Non−typhoidal Salmonella	31,700								31,700										
Serratia spp.	228,000	122,000		34,000		9,010	138,000	196,000	42,000										
Shigella spp.	640,000								640,000										
Staphylococcus aureus	2,140,000								328,000		1,320,000			1,400,000			1,570,000	26,800	
Streptococcus pneumoniae	4,240,000				698,000	849,000	725,000		476,000		1,110,000				2,250,000		3,890,000		
All pathogens	18,500,000	5,000,000	2,720,000	1,220,000	5,920,000	2,730,000	6,890,000	1,570,000	8,870,000	102,000	3,310,000	752,000	163,000	1,400,000	2,260,000	26,700	11,600,000	88,800	2,160

Counts
- <2.5k
- 2.5k − <5k
- 5k − <10k
- 10k − <25k
- 25k − <75k
- 75k − <150k
- >=150k

Nigeria

DALYs (count) *attributable to* bacterial antimicrobial resistance by pathogen−drug combinations, 2019

	Resistance to 1+	AG	Aminopenicillin	Anti−pseudomonal	BL−BLI	CP	3GC	4GC	FQ	Mono INH	Macrolide	MDR in S. Typhi and Paratyphi	MDR excluding XDR in TB	Methicillin	PCN	Mono RIF	TMP−SMX	Vanco	XDR in TB
Acinetobacter baumannii	117,000	11,300		47,500	2.96	29,700	792	9.33	27,300										
Citrobacter spp.	31,200	1,940		7,640		3,520	3,790	9,190	5,100										
Enterobacter spp.	146,000	21,300		20,300		17,300		30,200	33,500								21,900		
Enterococcus faecalis	56,000								53,100									2,960	
Enterococcus faecium	46,400								35,700									10,700	
Other enterococci	26,000								24,900									1,070	
Escherichia coli	711,000	67,600	13,600		37,600	54,400	233,000		172,000								134,000		
Group A Streptococcus	4,060										4,050								
Group B Streptococcus	234,000								157,000		70,500				4,280				
Haemophilus influenzae	51,300		21,500				29,800												
Klebsiella pneumoniae	924,000	165,000			20,100	155,000	295,000		156,000								134,000		
Morganella spp.	207						10.3	82.7	114										
Mycobacterium tuberculosis	115,000									15,200			91,700			7,220			1,280
Neisseria gonorrhoeae	183						1.28		181										
Proteus spp.	11,500	947	1,330				4,430		2,220								2,480		
Pseudomonas aeruginosa	258,000	18,100		37,900		86,200	36,100	16,000	63,500										
Salmonella Paratyphi	943								830			114							
Salmonella Typhi	128,000								43,300			86,800							
Non−typhoidal Salmonella	6,510								6,510										
Serratia spp.	54,800	7,140		7,150		2,940	3,430	30,100	4,110										
Shigella spp.	132,000								132,000										
Staphylococcus aureus	539,000								13,800		42,900			326,000			149,000	6,780	
Streptococcus pneumoniae	857,000				17,400	186,000	44,600		53,600		37,500				164,000		353,000		
All pathogens	4,450,000	293,000	36,500	121,000	75,100	536,000	650,000	85,600	984,000	15,400	159,000	85,600	91,700	326,000	168,000	7,220	796,000	21,500	1,280

Counts
- <2.5k
- 2.5k − <5k
- 5k − <10k
- 10k − <25k
- 25k − <75k
- 75k − <150k
- >=150k

Rwanda

DALYs (count) *associated with* bacterial antimicrobial resistance by pathogen−drug combinations, 2019

	Resistance to 1+	AG	Aminopenicillin	Anti−pseudomonal	BL−BLI	CP	3GC	4GC	FQ	Mono INH	Macrolide	MDR in S. Typhi and Paratyphi	MDR excluding XDR in TB	Methicillin	PCN	Mono RIF	TMP−SMX	Vanco	XDR in TB
Acinetobacter baumannii	19,900	6,530		19,100	12,200	12,300	18,300	12,900	12,700										
Citrobacter spp.	2,390	494		906		135	2,040	1,120	1,530										
Enterobacter spp.	14,000	7,930		6,310		1,000		13,300	8,020								9,800		
Enterococcus faecalis	9,340								9,240									473	
Enterococcus faecium	8,810								8,710									2,440	
Other enterococci	3,860								3,850									217	
Escherichia coli	68,300	18,100	65,000		51,500	3,470	39,300		31,900								57,900		
Group A Streptococcus	1,710										1,710								
Group B Streptococcus	19,700								8,860		16,100				158				
Haemophilus influenzae	5,240		4,930				925												
Klebsiella pneumoniae	94,100	69,900			92,900	4,300	89,300		59,800								84,000		
Morganella spp.	61.3						12.9	33.4	47.8										
Mycobacterium tuberculosis	13,500									1,610			10,900			814			145
Neisseria gonorrhoeae	152						0.881		152										
Proteus spp.	4,450	1,140	3,890				750		2,050								3,760		
Pseudomonas aeruginosa	33,200	19,800		8,420		13,000	26,400	8,050	12,400										
Salmonella Paratyphi	2.11								0.597			1.54							
Salmonella Typhi	34,000								14,600			27,200							
Non−typhoidal Salmonella	77.5								77.5										
Serratia spp.	5,510	2,820		1,510		300	3,700	2,480	1,750										
Shigella spp.	1,010								1,010										
Staphylococcus aureus	75,400								25,700		39,600			34,700			53,700	805	
Streptococcus pneumoniae	86,300				8,250	22,800	11,800		12,600		13,000				23,600		83,800		
All pathogens	501,000	127,000	73,800	36,200	165,000	57,300	193,000	37,900	215,000	1,610	70,300	27,200	10,900	34,700	23,700	814	293,000	3,930	145

Counts
- <2.5k
- 2.5k − <5k
- 5k − <10k
- 10k − <25k
- 25k − <75k
- 75k − <150k
- >=150k

Rwanda

DALYs (count) *attributable to* bacterial antimicrobial resistance by pathogen−drug combinations, 2019

	Resistance to 1+	AG	Aminopenicillin	Anti−pseudomonal	BL−BLI	CP	3GC	4GC	FQ	Mono INH	Macrolide	MDR in S. Typhi and Paratyphi	MDR excluding XDR in TB	Methicillin	PCN	Mono RIF	TMP−SMX	Vanco	XDR in TB
Acinetobacter baumannii	6,160	292		1,810	0.678	2,180	233	0.528	1,640										
Citrobacter spp.	697	22.6		164		28.4	160	99.7	223										
Enterobacter spp.	3,350	426		609		208		1,010	693								400		
Enterococcus faecalis	2,440								2,310									130	
Enterococcus faecium	2,240								1,670									578	
Other enterococci	791								747									43.9	
Escherichia coli	16,700	1,070	814		2,000	741	5,530		2,990								3,560		
Group A Streptococcus	166										166								
Group B Streptococcus	3,040								1,480		1,500				53.7				
Haemophilus influenzae	1,070		803				266												
Klebsiella pneumoniae	26,300	5,010			619	1,090	12,400		3,590								3,580		
Morganella spp.	14.1						0.595	5.51	7.98										
Mycobacterium tuberculosis	6,740									249			6,190			218			86.6
Neisseria gonorrhoeae	15						0.269		14.7										
Proteus spp.	565	51.8	77				156		139								141		
Pseudomonas aeruginosa	8,180	584		719		2,310	3,000	275	1,300										
Salmonella Paratyphi	0.335								0.128			0.207							
Salmonella Typhi	5,600								2,450			3,190							
Non−typhoidal Salmonella	15.4								15.4										
Serratia spp.	1,300	166		309		84.5	284	277	181										
Shigella spp.	206								206										
Staphylococcus aureus	16,000								995		1,380			7,770			5,640	241	
Streptococcus pneumoniae	16,500				98.9	4,930	327		1,460		473				1,030		8,210		
All pathogens	118,000	7,600	1,690	3,610	2,720	11,600	22,400	1,670	22,100	232	3,530	3,070	6,190	7,770	1,090	218	21,500	992	86.6

Counts

- <2.5k
- 2.5k − <5k
- 5k − <10k
- 10k − <25k
- 25k − <75k
- 75k − <150k

Sao Tome and Principe

DALYs (count) *associated with* bacterial antimicrobial resistance by pathogen−drug combinations, 2019

	Resistance to 1+	AG	Aminopenicillin	Anti−pseudomonal	BL−BLI	CP	3GC	4GC	FQ	Mono INH	Macrolide	MDR in S. Typhi and Paratyphi	MDR excluding XDR in TB	Methicillin	PCN	Mono RIF	TMP−SMX	Vanco	XDR in TB
Acinetobacter baumannii	304	185		296	199	213	266	197	251										
Citrobacter spp.	29.8	3.11		10.3		3.42	24.5	17.5	14.6										
Enterobacter spp.	171	54.7		76		27.7		113	83.3								139		
Enterococcus faecalis	98.5								95.3									9.47	
Enterococcus faecium	142								141									36.9	
Other enterococci	61.3								61.2									4.63	
Escherichia coli	730	265	706		583	43	272		284								644		
Group A Streptococcus	20.9										20.9								
Group B Streptococcus	174								74.3		140				2.37				
Haemophilus influenzae	30		21.6				13.2												
Klebsiella pneumoniae	981	626			919	26.9	806		694								897		
Morganella spp.	1.09						0.25	0.22	1										
Mycobacterium tuberculosis	70.4									14.6			55			0.179			0.718
Neisseria gonorrhoeae	2.37						0.004		2.37										
Proteus spp.	64.3	46	54.5				15.6		29.9								48		
Pseudomonas aeruginosa	411	190		201		203	263	133	266										
Salmonella Paratyphi	0.256								0.243			0.014							
Salmonella Typhi	261								53.9			224							
Non−typhoidal Salmonella	0.172								0.172										
Serratia spp.	75	24.8		13.7		5.48	62	44.6	23										
Shigella spp.	1.99								1.99										
Staphylococcus aureus	962								275		578			530			574	12.1	
Streptococcus pneumoniae	869				158	285	116		55.5		267				169		833		
All pathogens	5,460	1,400	782	597	1,860	807	1,840	506	2,410	14.6	1,010	224	55	530	172	0.179	3,140	63	0.718

Counts
- <2.5k
- 2.5k − <5k
- 5k − <10k

Sao Tome and Principe

DALYs (count) *attributable to* bacterial antimicrobial resistance by pathogen−drug combinations, 2019

	Resistance to 1+	AG	Aminopenicillin	Anti−pseudomonal	BL−BLI	CP	3GC	4GC	FQ	Mono INH	Macrolide	MDR in S. Typhi and Paratyphi	MDR excluding XDR in TB	Methicillin	PCN	Mono RIF	TMP−SMX	Vanco	XDR in TB
Acinetobacter baumannii	94.7	7.96		18.4	0.01	34.4	2.01	0.009	31.9										
Citrobacter spp.	8.65	0.145		1.94		0.758	1.83	1.9	2.08										
Enterobacter spp.	38.4	3.1		7.22		5.66		6.49	8.35								7.51		
Enterococcus faecalis	26.4								23.6									2.79	
Enterococcus faecium	35.7								27.2									8.59	
Other enterococci	12.6								11.8									0.858	
Escherichia coli	166	16.2	10.6		29.7	9.16	34.2		26.3								40.1		
Group A Streptococcus	1.95										1.93								
Group B Streptococcus	26.5								12.6		13.3				0.654				
Haemophilus influenzae	7.02		3.17				3.85												
Klebsiella pneumoniae	257	43.7			14.5	8.55	107		42.8								40.4		
Morganella spp.	0.246						0.027	0.036	0.183										
Mycobacterium tuberculosis	32.6									2.08			30.1			0.047			0.418
Neisseria gonorrhoeae	0.231						0.002		0.229										
Proteus spp.	9.3	2.27	0.932				2.87		1.74								1.54		
Pseudomonas aeruginosa	102	5.03		16.6		33	15.9	1.79	29.4										
Salmonella Paratyphi	0.053								0.051			0.002							
Salmonella Typhi	38.7								10.2			29.5							
Non−typhoidal Salmonella	0.033								0.033										
Serratia spp.	19.7	1.36		2.98		1.49	5.34	6.13	2.35										
Shigella spp.	0.365								0.365										
Staphylococcus aureus	219								10.9		21.1			125			58.5	3.3	
Streptococcus pneumoniae	168				3.25	61.3	1.63		6.41		9.94				3.93		81.5		
All pathogens	1,260	79.8	14.6	47.2	47.5	154	175	16.4	248	2.06	46.1	27.9	30.1	125	4.58	0.047	230	15.5	0.418

Counts

<2.5k

Senegal

DALYs (count) *associated with* bacterial antimicrobial resistance by pathogen−drug combinations, 2019

	Resistance to 1+	AG	Aminopenicillin	Anti−pseudomonal	BL−BLI	CP	3GC	4GC	FQ	Mono INH	Macrolide	MDR in S. Typhi and Paratyphi	MDR excluding XDR in TB	Methicillin	PCN	Mono RIF	TMP−SMX	Vanco	XDR in TB
Acinetobacter baumannii	27,000	12,000		26,800	10,000	9,270	15,500	15,300	11,800										
Citrobacter spp.	3,410	1,290		1,510		532	3,110	2,860	1,380										
Enterobacter spp.	19,000	9,630		7,570		3,440		16,200	12,000								15,700		
Enterococcus faecalis	12,300								12,100									808	
Enterococcus faecium	11,200								11,200									2,630	
Other enterococci	5,930								5,920									268	
Escherichia coli	97,000	37,200	92,600		79,300	10,300	43,400		52,300								90,600		
Group A Streptococcus	2,190										2,190								
Group B Streptococcus	32,200								20,700		21,000				652				
Haemophilus influenzae	5,750		4,060				2,800												
Klebsiella pneumoniae	130,000	93,900			123,000	5,260	58,400		98,700								124,000		
Morganella spp.	95						23.5	60.9	68.5										
Mycobacterium tuberculosis	12,400									4,100			7,190			997			96.3
Neisseria gonorrhoeae	117						0.243		117										
Proteus spp.	5,230	1,450	4,540				1,410		2,820								4,120		
Pseudomonas aeruginosa	38,300	20,200		20,600		15,100	21,800	16,400	22,000										
Salmonella Paratyphi	20.9								17.8			3.35							
Salmonella Typhi	4,960								3,790			1,190							
Non−typhoidal Salmonella	61.5								61.5										
Serratia spp.	12,200	6,270		1,570		535	10,400	11,000	3,280										
Shigella spp.	1,250								1,250										
Staphylococcus aureus	91,300								20,500		52,700			33,000			62,700	3,010	
Streptococcus pneumoniae	103,000				18,100	23,900	13,400		9,820		23,700				14,000		99,900		
All pathogens	615,000	182,000	101,000	58,100	231,000	68,300	170,000	61,700	290,000	4,100	99,600	1,200	7,190	33,000	14,600	997	397,000	6,720	96.3

Counts
- <2.5k
- 2.5k − <5k
- 5k − <10k
- 10k − <25k
- 25k − <75k
- 75k − <150k
- >=150k

Senegal

DALYs (count) *attributable to* bacterial antimicrobial resistance by pathogen−drug combinations, 2019

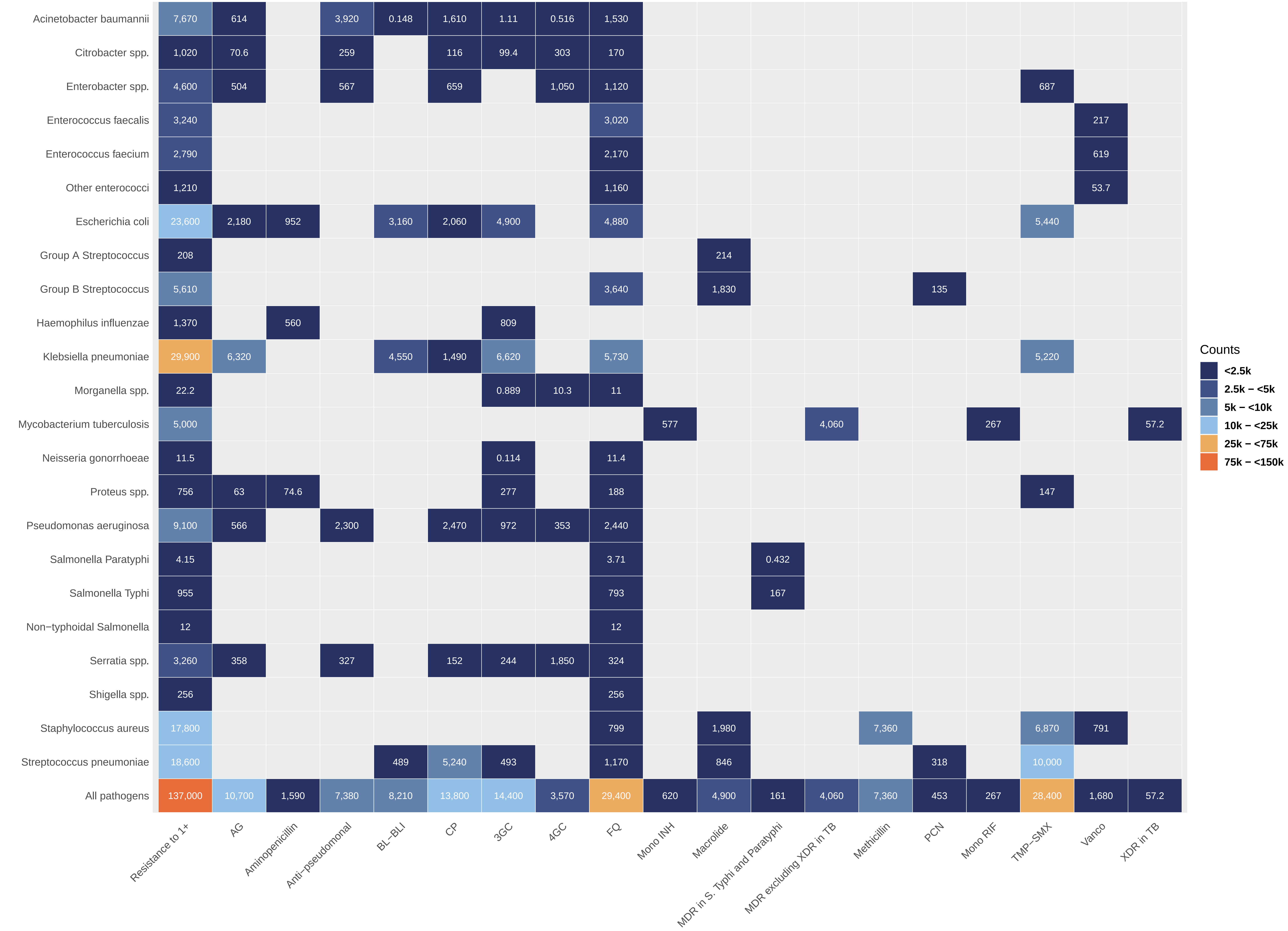

Seychelles

DALYs (count) *associated with* bacterial antimicrobial resistance by pathogen−drug combinations, 2019

	Resistance to 1+	AG	Aminopenicillin	Anti−pseudomonal	BL−BLI	CP	3GC	4GC	FQ	Mono INH	Macrolide	MDR in S. Typhi and Paratyphi	MDR excluding XDR in TB	Methicillin	PCN	Mono RIF	TMP−SMX	Vanco	XDR in TB
Acinetobacter baumannii	206	145		189	126	157	151	198	167										
Citrobacter spp.	19	5.15		2.77		3.86	12	14.1	5.84										
Enterobacter spp.	87.3	22.7		28.1		28.9		36.6	29.9								59.6		
Enterococcus faecalis	57.8								55.9									6.18	
Enterococcus faecium	84.8								84.4									14.9	
Other enterococci	40.3								40									14.4	
Escherichia coli	328	43.9	321		234	39.4	129		160								217		
Group A Streptococcus	14.8										14.8								
Group B Streptococcus	28.1								5.96		24.6				1.63				
Haemophilus influenzae	25.2		23.3				6.54												
Klebsiella pneumoniae	223	33.4			175	45.9	170		117								136		
Morganella spp.	0.765						0.419	0.137	0.497										
Mycobacterium tuberculosis	1.33									0.609			0.533			0.095			0.095
Neisseria gonorrhoeae	1.19						0.008		1.19										
Proteus spp.	31.2	5.14	29.9				7.87		9.62								15.8		
Pseudomonas aeruginosa	118	37.3		42.9		83.9	54.7	48.7	50.8										
Salmonella Paratyphi	1.79								1.73			0.063							
Salmonella Typhi	21.8								19			3.07							
Non−typhoidal Salmonella	6.33								6.33										
Serratia spp.	15.8	2.77		2.6		2.31	7.71	9.87	6.45										
Shigella spp.	0.232								0.232										
Staphylococcus aureus	309								127		195			265			24.3	20.2	
Streptococcus pneumoniae	349				58.5	123	119		13.7		201				80.5		269		
All pathogens	1,970	296	374	265	594	485	657	307	902	0.609	436	3.13	0.533	265	82.1	0.095	722	55.7	0.095

Counts

<2.5k

Seychelles

DALYs (count) *attributable to* bacterial antimicrobial resistance by pathogen−drug combinations, 2019

	Resistance to 1+	AG	Aminopenicillin	Anti−pseudomonal	BL−BLI	CP	3GC	4GC	FQ	Mono INH	Macrolide	MDR in S. Typhi and Paratyphi	MDR excluding XDR in TB	Methicillin	PCN	Mono RIF	TMP−SMX	Vanco	XDR in TB
Acinetobacter baumannii	64.1	6.47		7.47	0.022	26.3	0.516	2.51	20.8										
Citrobacter spp.	5.3	0.295		0.499		0.922	0.872	1.93	0.779										
Enterobacter spp.	19.9	1.48		2.49		7.31		1.61	3.26								3.81		
Enterococcus faecalis	15.5								13.8									1.78	
Enterococcus faecium	20.5								16.8									3.61	
Other enterococci	9.26								6.85									2.41	
Escherichia coli	79.2	2.73	7.47		13.5	9.08	16.7		16.6								13.1		
Group A Streptococcus	1.3										1.41								
Group B Streptococcus	3.86								1.03		2.52				0.309				
Haemophilus influenzae	5.33		3.5				1.83												
Klebsiella pneumoniae	61.5	3.28			4.47	12.2	24.6		9.25								7.62		
Morganella spp.	0.192						0.076	0.03	0.086										
Mycobacterium tuberculosis	0.435									0.085			0.274			0.024			0.054
Neisseria gonorrhoeae	0.118						0.002		0.115										
Proteus spp.	3.99	0.258	0.691				1.81		0.661								0.583		
Pseudomonas aeruginosa	28.6	1.04		2.29		16	2.55	1.41	5.34										
Salmonella Paratyphi	0.365								0.356			0.009							
Salmonella Typhi	4.28								3.89			0.4							
Non−typhoidal Salmonella	1.18								1.18										
Serratia spp.	3.81	0.151		0.525		0.632	0.493	1.26	0.746										
Shigella spp.	0.04								0.04										
Staphylococcus aureus	91.2								5.56		7.31			71.5			1.97	4.87	
Streptococcus pneumoniae	71.2				0.382	27.2	7.93		1.55		8.33				1.7		24.4		
All pathogens	491	15.7	11.6	13.3	18.4	99.6	57.3	8.75	109	0.087	19.4	0.4	0.274	71.5	2.01	0.024	51.5	12.7	0.054

Counts

<2.5k

Sierra Leone

DALYs (count) *associated with* bacterial antimicrobial resistance by pathogen−drug combinations, 2019

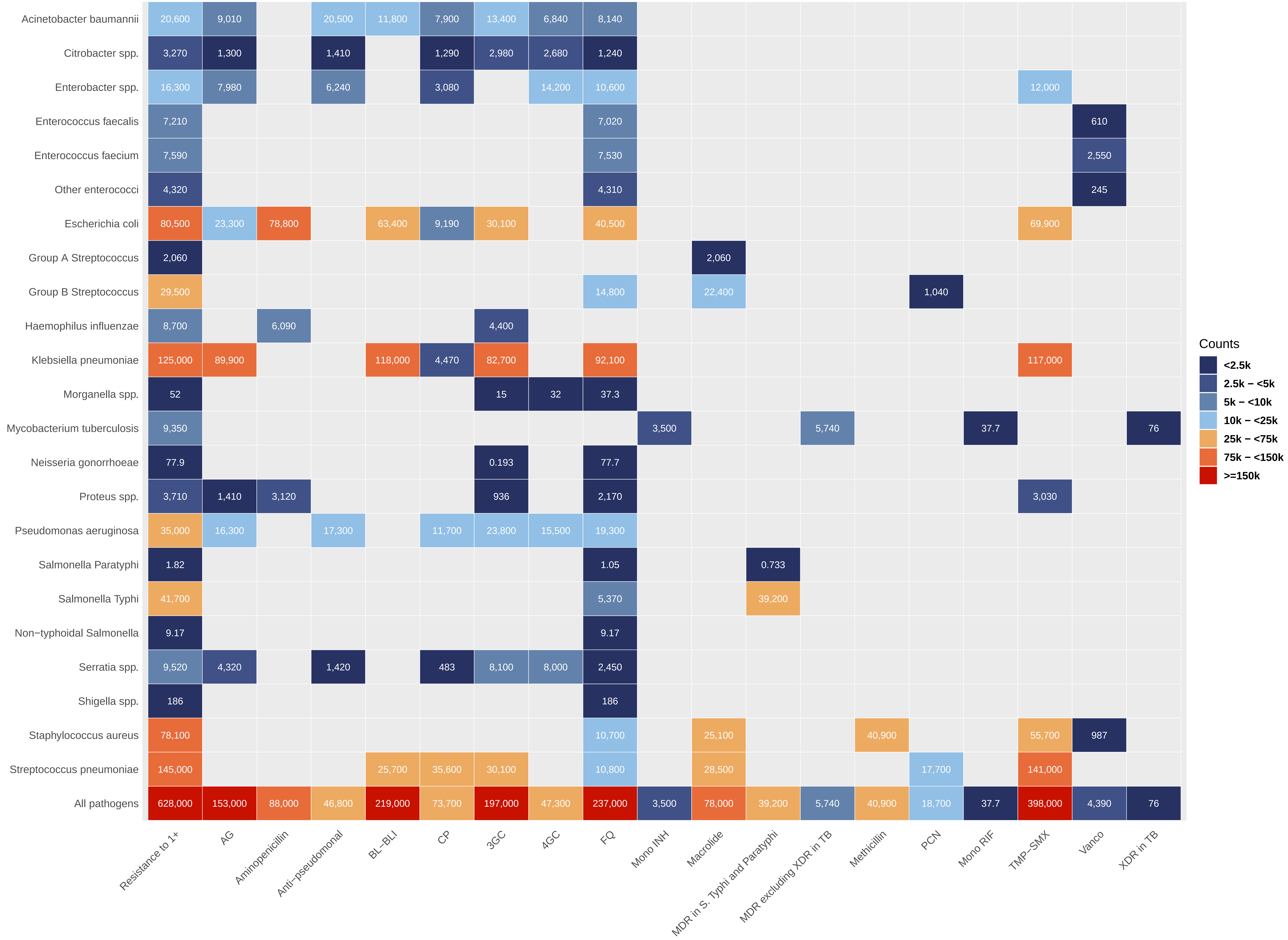

Sierra Leone

DALYs (count) *attributable to* bacterial antimicrobial resistance by pathogen−drug combinations, 2019

	Resistance to 1+	AG	Aminopenicillin	Anti−pseudomonal	BL−BLI	CP	3GC	4GC	FQ	Mono INH	Macrolide	MDR in S. Typhi and Paratyphi	MDR excluding XDR in TB	Methicillin	PCN	Mono RIF	TMP−SMX	Vanco	XDR in TB
Acinetobacter baumannii	5,910	463		3,000	0.168	1,420	1.98	0.456	1,030										
Citrobacter spp.	998	66.7		182		283	103	215	147										
Enterobacter spp.	4,000	414		471		595		988	1,010								522		
Enterococcus faecalis	1,920								1,750									176	
Enterococcus faecium	1,990								1,400									591	
Other enterococci	885								836									49.1	
Escherichia coli	19,200	1,380	1,290		3,100	1,910	3,450		3,900								4,220		
Group A Streptococcus	200										196								
Group B Streptococcus	4,790								2,540		1,990				189				
Haemophilus influenzae	2,090		819				1,270												
Klebsiella pneumoniae	31,100	6,220			3,100	1,320	9,860		5,500								5,100		
Morganella spp.	12.3						0.75	5.5	6.01										
Mycobacterium tuberculosis	3,830									527			3,260			10.1			45.4
Neisseria gonorrhoeae	7.65						0.088		7.56										
Proteus spp.	540	60.2	53.2				178		143								99.7		
Pseudomonas aeruginosa	8,640	450		2,080		1,920	1,570	469	2,150										
Salmonella Paratyphi	0.33								0.226			0.106							
Salmonella Typhi	5,950								902			5,110							
Non−typhoidal Salmonella	1.9								1.9										
Serratia spp.	2,530	246		300		140	317	1,290	243										
Shigella spp.	38.5								38.5										
Staphylococcus aureus	18,000								483		910			10,100			6,270	259	
Streptococcus pneumoniae	27,000				484	7,880	1,660		1,260		1,040				237		14,400		
All pathogens	140,000	9,300	2,160	6,030	6,680	15,500	18,400	2,970	23,300	508	4,250	4,800	3,260	10,100	426	10.1	30,600	1,080	45.4

Counts
- <2.5k
- 2.5k − <5k
- 5k − <10k
- 10k − <25k
- 25k − <75k
- 75k − <150k

South Africa

DALYs (count) *associated with* bacterial antimicrobial resistance by pathogen−drug combinations, 2019

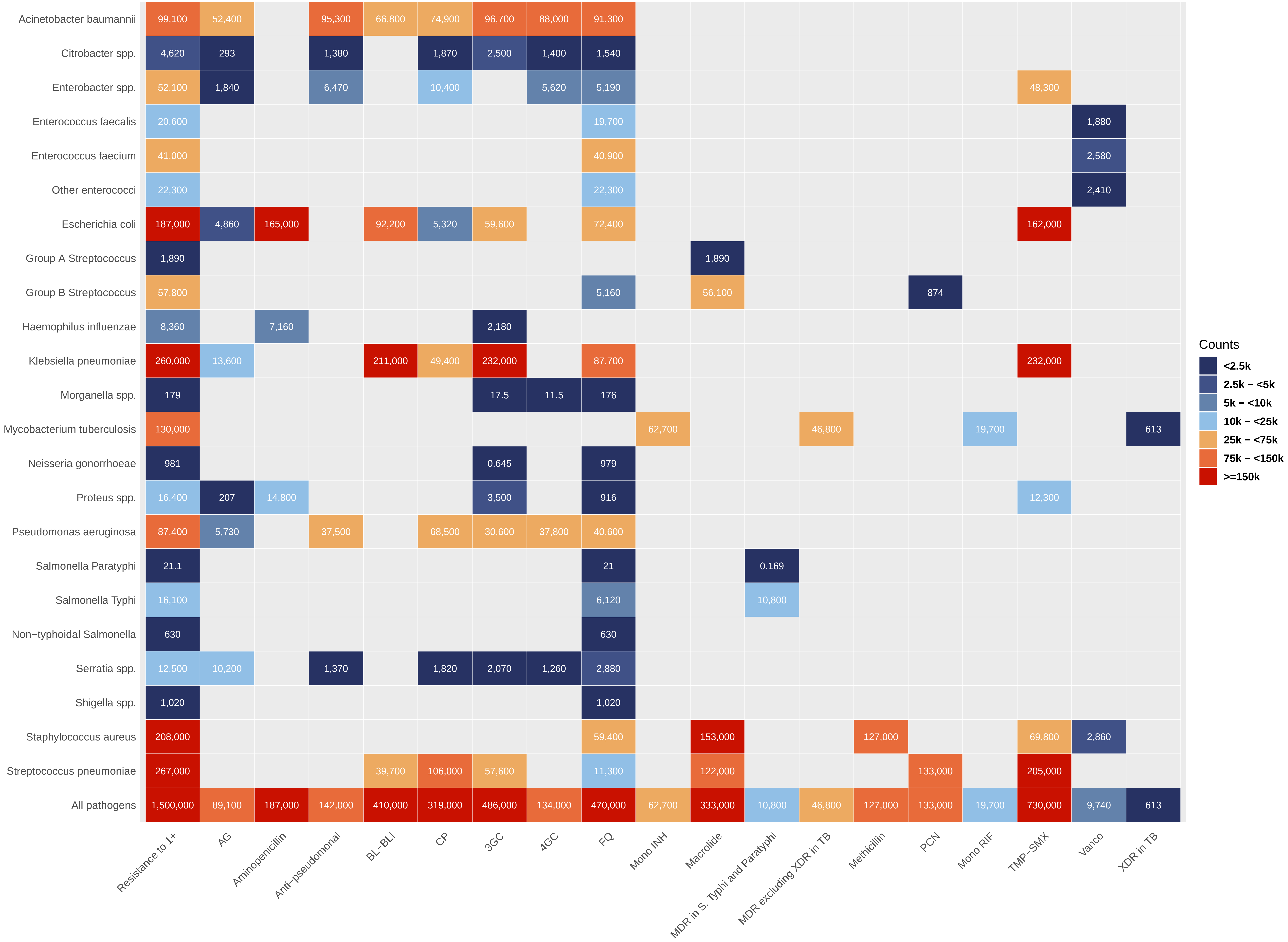

South Africa

DALYs (count) *attributable to* bacterial antimicrobial resistance by pathogen−drug combinations, 2019

	Resistance to 1+	AG	Aminopenicillin	Anti−pseudomonal	BL−BLI	CP	3GC	4GC	FQ	Mono INH	Macrolide	MDR in S. Typhi and Paratyphi	MDR excluding XDR in TB	Methicillin	PCN	Mono RIF	TMP−SMX	Vanco	XDR in TB
Acinetobacter baumannii	31,500	2,230		3,980	2.3	12,200	1,050	36.4	12,000										
Citrobacter spp.	1,370	17.9		247		525	216	95.7	263										
Enterobacter spp.	8,370	211		499		2,730		208	786								3,980		
Enterococcus faecalis	5,520								4,920									597	
Enterococcus faecium	9,210								8,510									700	
Other enterococci	4,660								4,230									433	
Escherichia coli	40,400	434	5,740		5,480	1,330	8,420		7,350								11,600		
Group A Streptococcus	184										190								
Group B Streptococcus	6,910								818		6,170				242				
Haemophilus influenzae	1,800		1,160				640												
Klebsiella pneumoniae	73,700	1,710			1,490	13,000	36,100		7,200								14,200		
Morganella spp.	38.2						2.54	1.84	33.8										
Mycobacterium tuberculosis	40,200									9,090			25,600			5,170			358
Neisseria gonorrhoeae	95.9						0.505		95.4										
Proteus spp.	1,880	22.2	336				863		97.5								553		
Pseudomonas aeruginosa	20,900	179		1,930		13,100	531	777	4,320										
Salmonella Paratyphi	4.27								4.26			0.018							
Salmonella Typhi	2,590								1,190			1,430							
Non−typhoidal Salmonella	120								120										
Serratia spp.	1,880	647		206		471	113	94.1	326										
Shigella spp.	208								208										
Staphylococcus aureus	48,500								2,520		6,090			32,300			6,820	782	
Streptococcus pneumoniae	58,000				293	24,000	1,390		1,280		4,730				7,810		18,500		
All pathogens	358,000	5,480	7,230	6,870	7,260	67,300	49,400	1,210	56,300	9,820	16,900	1,350	25,600	32,300	8,050	5,170	55,600	2,510	358

Counts
- <2.5k
- 2.5k − <5k
- 5k − <10k
- 10k − <25k
- 25k − <75k
- >=150k

South Sudan

DALYs (count) *associated with* bacterial antimicrobial resistance by pathogen−drug combinations, 2019

	Resistance to 1+	AG	Aminopenicillin	Anti−pseudomonal	BL−BLI	CP	3GC	4GC	FQ	Mono INH	Macrolide	MDR in S. Typhi and Paratyphi	MDR excluding XDR in TB	Methicillin	PCN	Mono RIF	TMP−SMX	Vanco	XDR in TB
Acinetobacter baumannii	31,800	10,300		30,500	21,600	7,950	23,300	13,500	10,400										
Citrobacter spp.	9,100	3,900		3,780		1,790	8,120	7,640	4,970										
Enterobacter spp.	33,300	13,700		12,400		5,710		31,100	18,600								25,100		
Enterococcus faecalis	8,960								8,840									606	
Enterococcus faecium	5,900								5,860									1,290	
Other enterococci	4,700								4,670									367	
Escherichia coli	97,300	25,000	94,000		70,800	11,500	36,700		39,600								80,100		
Group A Streptococcus	3,120										3,120								
Group B Streptococcus	62,900								36,000		47,500				2,540				
Haemophilus influenzae	27,600		26,300				7,490												
Klebsiella pneumoniae	127,000	96,600			123,000	1,440	85,500		76,300								110,000		
Morganella spp.	49.3						12.4	31.7	35.4										
Mycobacterium tuberculosis	20,400									3,000			16,600			660			222
Neisseria gonorrhoeae	89.9						0.919		89.6										
Proteus spp.	3,940	1,980	3,210				879		2,500								3,550		
Pseudomonas aeruginosa	27,400	10,100		9,810		5,950	22,000	14,000	7,180										
Salmonella Paratyphi	1.88								0.146			1.73							
Salmonella Typhi	14,900								7,410			8,090							
Non−typhoidal Salmonella	20.8								20.8										
Serratia spp.	14,400	7,970		4,230		641	10,300	11,700	3,910										
Shigella spp.	9,660								9,660										
Staphylococcus aureus	62,500								14,500		36,600			18,800			47,500	1,250	
Streptococcus pneumoniae	248,000				40,500	67,200	41,300		128,000		35,000				36,500		227,000		
All pathogens	812,000	170,000	124,000	60,800	256,000	102,000	235,000	77,900	378,000	3,000	122,000	8,090	16,600	18,800	39,000	660	493,000	3,510	222

Counts
- <2.5k
- 2.5k − <5k
- 5k − <10k
- 10k − <25k
- 25k − <75k
- 75k − <150k
- >=150k

South Sudan

DALYs (count) *attributable to* bacterial antimicrobial resistance by pathogen−drug combinations, 2019

	Resistance to 1+	AG	Aminopenicillin	Anti−pseudomonal	BL−BLI	CP	3GC	4GC	FQ	Mono INH	Macrolide	MDR in S. Typhi and Paratyphi	MDR excluding XDR in TB	Methicillin	PCN	Mono RIF	TMP−SMX	Vanco	XDR in TB
Acinetobacter baumannii	9,170	579		5,320	31.4	1,560	254	0.786	1,430										
Citrobacter spp.	2,720	184		563		361	235	734	636										
Enterobacter spp.	8,170	710		1,040		1,150		2,510	1,650								1,130		
Enterococcus faecalis	2,360								2,200									161	
Enterococcus faecium	1,460								1,150									311	
Other enterococci	978								907									71.6	
Escherichia coli	22,900	1,530	1,910		3,700	2,530	4,480		3,790								5,000		
Group A Streptococcus	282										288								
Group B Streptococcus	10,700								6,060		4,240				455				
Haemophilus influenzae	5,860		3,810				2,050												
Klebsiella pneumoniae	31,400	6,950			3,610	624	11,000		4,520								4,710		
Morganella spp.	11.5						0.481	5.39	5.67										
Mycobacterium tuberculosis	10,300									437			9,490			181			133
Neisseria gonorrhoeae	8.92						0.258		8.66										
Proteus spp.	574	84.5	52.9				159		160								123		
Pseudomonas aeruginosa	6,980	326		1,500		1,130	2,260	989	782										
Salmonella Paratyphi	0.262								0.034			0.224							
Salmonella Typhi	2,540								1,500			1,000							
Non−typhoidal Salmonella	4.3								4.3										
Serratia spp.	3,630	446		822		195	320	1,450	382										
Shigella spp.	1,980								1,980										
Staphylococcus aureus	11,500								546		1,320			4,050			5,230	331	
Streptococcus pneumoniae	52,100				653	12,700	1,240		16,200		1,150				537		19,500		
All pathogens	186,000	10,800	5,770	9,250	7,990	20,300	22,000	5,700	43,900	472	6,970	996	9,490	4,050	992	181	35,700	874	133

Counts

- **<2.5k**
- **2.5k − <5k**
- **5k − <10k**
- **10k − <25k**
- **25k − <75k**
- **>=150k**

Swaziland

DALYs (count) *associated with* bacterial antimicrobial resistance by pathogen−drug combinations, 2019

	Resistance to 1+	AG	Aminopenicillin	Anti−pseudomonal	BL−BLI	CP	3GC	4GC	FQ	Mono INH	Macrolide	MDR in S. Typhi and Paratyphi	MDR excluding XDR in TB	Methicillin	PCN	Mono RIF	TMP−SMX	Vanco	XDR in TB
Acinetobacter baumannii	2,840	1,060		2,790	1,690	1,630	2,590	2,030	2,070										
Citrobacter spp.	178	20.8		64.4		28.3	115	113	61.6										
Enterobacter spp.	1,200	229		363		221		518	346								1,050		
Enterococcus faecalis	615								598									45.9	
Enterococcus faecium	958								955									171	
Other enterococci	397								396									30.7	
Escherichia coli	6,140	1,650	5,940		3,530	424	2,720		1,700								5,090		
Group A Streptococcus	93.6										93.6								
Group B Streptococcus	1,630								220		1,550				88.5				
Haemophilus influenzae	561		493				153												
Klebsiella pneumoniae	9,180	3,720			8,050	37.8	7,900		4,570								8,410		
Morganella spp.	6.67						0.981	3.16	5.24										
Mycobacterium tuberculosis	8,310									1,390			6,000			840			80.6
Neisseria gonorrhoeae	14.3						0.038		14.2										
Proteus spp.	425	32.1	378				90.5		49.9								325		
Pseudomonas aeruginosa	2,740	859		1,690		1,480	1,590	1,110	1,310										
Salmonella Paratyphi	0.603								0.575			0.034							
Salmonella Typhi	967								378			645							
Non−typhoidal Salmonella	4.43								4.43										
Serratia spp.	416	257		69.4		36.4	132	221	97.1										
Shigella spp.	294								294										
Staphylococcus aureus	7,040								2,000		2,170			1,660			5,720	90.2	
Streptococcus pneumoniae	9,980				2,230	2,850	1,520		1,020		3,470				4,210		9,030		
All pathogens	54,000	7,820	6,810	4,970	15,500	6,700	16,800	3,990	16,100	1,390	7,280	645	6,000	1,660	4,300	840	29,600	338	80.6

Counts
- <2.5k
- 2.5k − <5k
- 5k − <10k
- 10k − <25k
- 25k − <75k

Swaziland

DALYs (count) *attributable to* bacterial antimicrobial resistance by pathogen−drug combinations, 2019

	Resistance to 1+	AG	Aminopenicillin	Anti−pseudomonal	BL−BLI	CP	3GC	4GC	FQ	Mono INH	Macrolide	MDR in S. Typhi and Paratyphi	MDR excluding XDR in TB	Methicillin	PCN	Mono RIF	TMP−SMX	Vanco	XDR in TB
Acinetobacter baumannii	878	46.5		278	0.024	268	14.2	0.089	272										
Citrobacter spp.	50.1	1.12		12.4		6.98	7.68	12.7	9.21										
Enterobacter spp.	236	15.6		34.2		49.8		29.2	37.5								69.3		
Enterococcus faecalis	163								149									14.1	
Enterococcus faecium	231								190									41	
Other enterococci	82.2								76.3									5.91	
Escherichia coli	1,420	104	179		171	95.1	386		158								326		
Group A Streptococcus	8.83										8.42								
Group B Streptococcus	205								34.8		161				16.2				
Haemophilus influenzae	121		76.7				43.9												
Klebsiella pneumoniae	2,390	275			94.1	23.5	1,250		300								441		
Morganella spp.	1.5						0.05	0.532	0.916										
Mycobacterium tuberculosis	3,820									209			3,330			226			48.1
Neisseria gonorrhoeae	1.39						0.019		1.37										
Proteus spp.	50.1	2.01	8.23				21.3		4.29								13.9		
Pseudomonas aeruginosa	691	23.6		177		272	62.1	14.4	141										
Salmonella Paratyphi	0.122								0.118			0.004							
Salmonella Typhi	157								73.8			85.6							
Non−typhoidal Salmonella	0.849								0.849										
Serratia spp.	81.2	15.6		12.9		10.2	5.29	26.7	10.1										
Shigella spp.	60.4								60.4										
Staphylococcus aureus	1,230								84.1		78.6			361			685	26	
Streptococcus pneumoniae	2,020				59	602	43.6		118		122				244		828		
All pathogens	13,900	484	264	514	324	1,330	1,840	83.7	1,720	201	369	82.2	3,330	361	260	226	2,360	86.9	48.1

Counts
- <2.5k
- 2.5k − <5k
- 10k − <25k

Tanzania

DALYs (count) *associated with* bacterial antimicrobial resistance by pathogen−drug combinations, 2019

	Resistance to 1+	AG	Aminopenicillin	Anti−pseudomonal	BL−BLI	CP	3GC	4GC	FQ	Mono INH	Macrolide	MDR in S. Typhi and Paratyphi	MDR excluding XDR in TB	Methicillin	PCN	Mono RIF	TMP−SMX	Vanco	XDR in TB
Acinetobacter baumannii	110,000	45,000		103,000	86,800	62,200	100,000	54,600	93,100										
Citrobacter spp.	17,100	6,090		7,570		3,480	14,600	9,670	12,100										
Enterobacter spp.	85,800	34,100		37,700		14,200		75,500	47,300								65,000		
Enterococcus faecalis	62,900								62,300									3,750	
Enterococcus faecium	46,300								45,900									12,400	
Other enterococci	26,000								25,900									1,110	
Escherichia coli	376,000	85,400	354,000		292,000	42,700	187,000		142,000								320,000		
Group A Streptococcus	15,000										15,000								
Group B Streptococcus	144,000								77,300		111,000				671				
Haemophilus influenzae	45,000		40,600				12,200												
Klebsiella pneumoniae	587,000	427,000			553,000	4,620	407,000		437,000								551,000		
Morganella spp.	269						60.9	153	211										
Mycobacterium tuberculosis	76,000									7,230			48,900			19,200			653
Neisseria gonorrhoeae	902						27.1		900										
Proteus spp.	23,800	8,690	21,100				4,440		13,100								20,600		
Pseudomonas aeruginosa	172,000	53,100		60,300		49,500	122,000	54,000	112,000										
Salmonella Paratyphi	25.5								1.02			24.3							
Salmonella Typhi	283,000								135,000			238,000							
Non−typhoidal Salmonella	13								13										
Serratia spp.	39,400	20,300		12,900		2,580	24,400	24,100	11,300										
Shigella spp.	644								644										
Staphylococcus aureus	502,000								369,000		252,000			215,000			361,000	4,950	
Streptococcus pneumoniae	621,000				55,300	160,000	80,900		85,600		116,000				234,000		600,000		
All pathogens	3,230,000	680,000	416,000	221,000	987,000	340,000	952,000	218,000	1,670,000	7,230	494,000	238,000	48,900	215,000	235,000	19,200	1,920,000	22,200	653

Counts
- <2.5k
- 2.5k − <5k
- 5k − <10k
- 10k − <25k
- 25k − <75k
- 75k − <150k
- >=150k

Tanzania

DALYs (count) *attributable to* bacterial antimicrobial resistance by pathogen−drug combinations, 2019

	Resistance to 1+	AG	Aminopenicillin	Anti−pseudomonal	BL−BLI	CP	3GC	4GC	FQ	Mono INH	Macrolide	MDR in S. Typhi and Paratyphi	MDR excluding XDR in TB	Methicillin	PCN	Mono RIF	TMP−SMX	Vanco	XDR in TB
Acinetobacter baumannii	34,100	1,980		7,530	192	10,100	1,820	3	12,500										
Citrobacter spp.	5,070	281		1,060		614	790	638	1,690										
Enterobacter spp.	20,700	1,770		3,310		2,930		5,230	4,390								3,110		
Enterococcus faecalis	16,500								15,500									963	
Enterococcus faecium	11,700								8,800									2,920	
Other enterococci	5,310								5,080									234	
Escherichia coli	91,000	5,250	4,300		13,000	9,520	24,800		13,400								20,800		
Group A Streptococcus	1,420										1,430								
Group B Streptococcus	23,300								13,000		10,200				247				
Haemophilus influenzae	9,640		6,190				3,450												
Klebsiella pneumoniae	145,000	29,300			13,000	2,330	50,600		25,900								23,900		
Morganella spp.	62.3						2.59	25	34.7										
Mycobacterium tuberculosis	34,700									1,110			28,100			5,190			390
Neisseria gonorrhoeae	91						5.28		85.7										
Proteus spp.	3,210	375	386				867		853								728		
Pseudomonas aeruginosa	42,900	1,430		6,750		7,730	11,800	1,830	13,300										
Salmonella Paratyphi	3.58								0.295			3.26							
Salmonella Typhi	48,000								21,700			26,700							
Non−typhoidal Salmonella	2.64								2.64										
Serratia spp.	9,360	1,150		2,520		731	1,150	2,640	1,140										
Shigella spp.	131								131										
Staphylococcus aureus	106,000								14,600		7,520			49,000			33,500	1,540	
Streptococcus pneumoniae	121,000				668	34,400	2,240		9,710		4,180				13,100		57,100		
All pathogens	730,000	41,500	10,900	21,200	26,900	68,300	97,600	10,400	162,000	1,100	23,100	25,900	28,100	49,000	13,300	5,190	139,000	5,650	390

Counts
- <2.5k
- 2.5k − <5k
- 5k − <10k
- 10k − <25k
- 25k − <75k
- 75k − <150k
- >=150k

The Gambia

DALYs (count) *associated with* bacterial antimicrobial resistance by pathogen−drug combinations, 2019

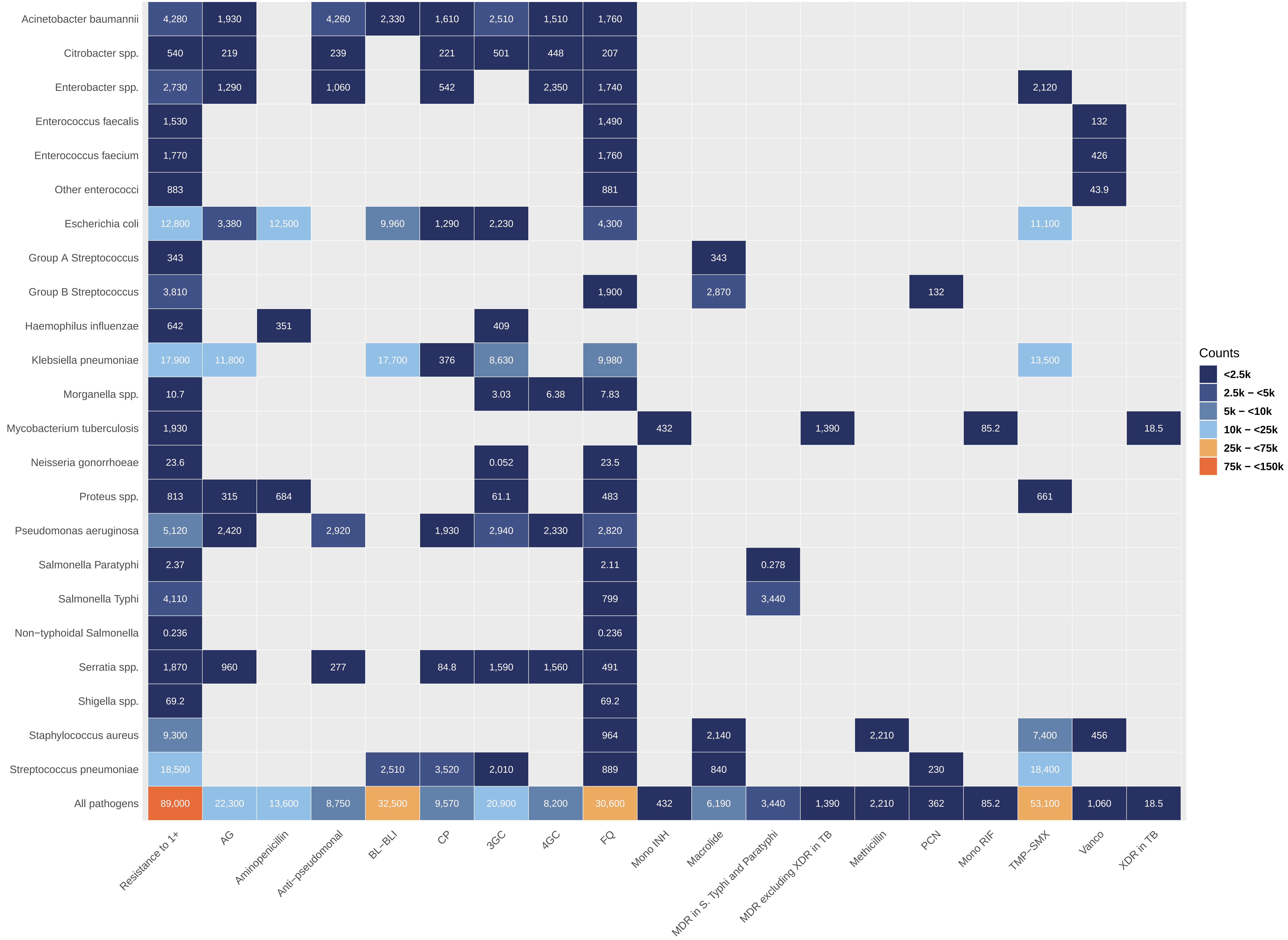

The Gambia

DALYs (count) *attributable to* bacterial antimicrobial resistance by pathogen−drug combinations, 2019

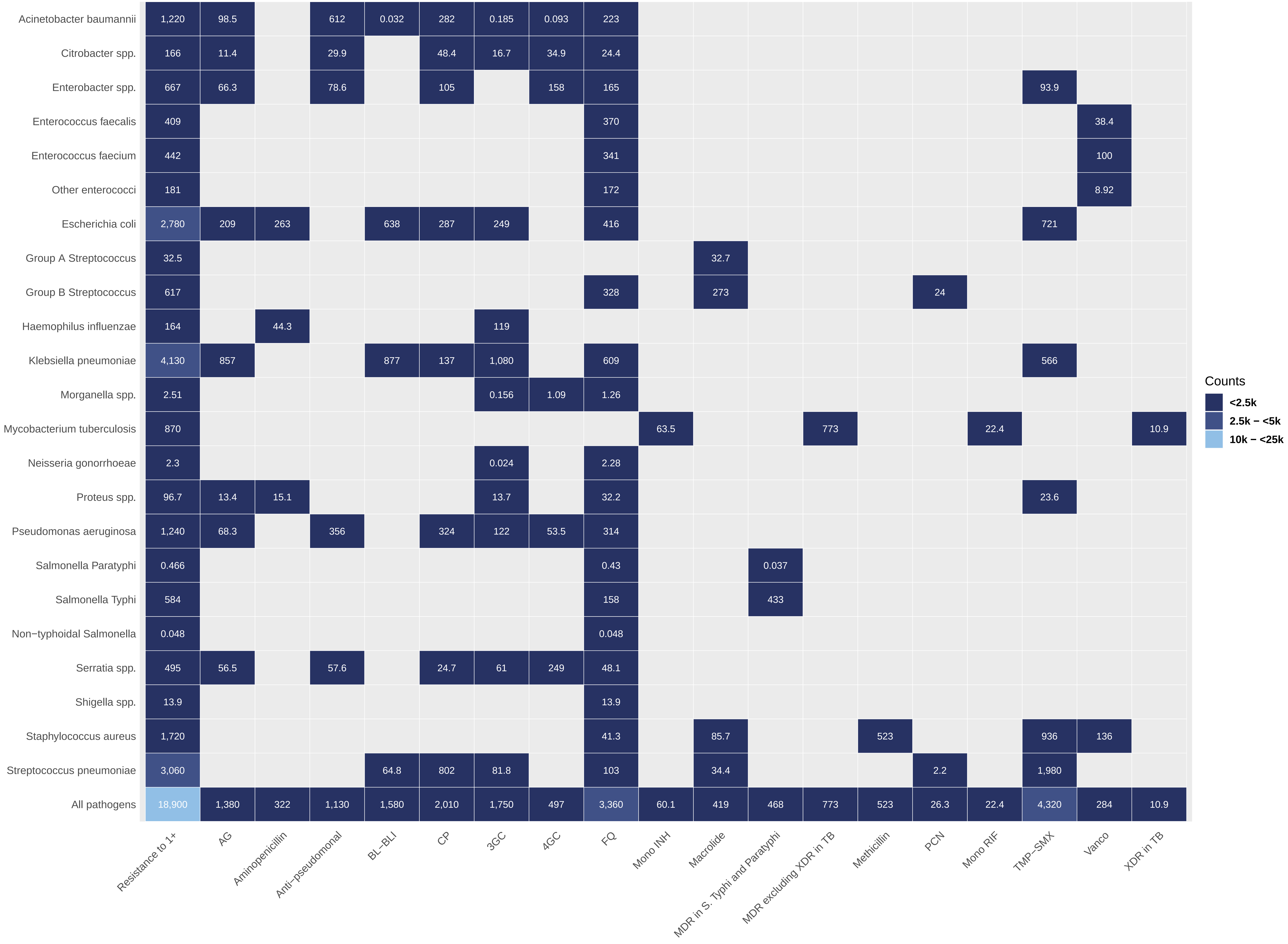

Togo

DALYs (count) *associated with* bacterial antimicrobial resistance by pathogen−drug combinations, 2019

	Resistance to 1+	AG	Aminopenicillin	Anti−pseudomonal	BL−BLI	CP	3GC	4GC	FQ	Mono INH	Macrolide	MDR in S. Typhi and Paratyphi	MDR excluding XDR in TB	Methicillin	PCN	Mono RIF	TMP−SMX	Vanco	XDR in TB
Acinetobacter baumannii	12,900	5,780		12,700	5,140	4,150	7,970	8,380	5,950										
Citrobacter spp.	1,680	577		725		255	1,490	1,370	669										
Enterobacter spp.	10,100	3,940		3,660		1,620		8,080	6,610								8,060		
Enterococcus faecalis	5,600								5,520									364	
Enterococcus faecium	5,430								5,400									1,240	
Other enterococci	2,770								2,760									131	
Escherichia coli	53,200	28,400	51,000		40,700	6,250	25,000		26,600								47,500		
Group A Streptococcus	1,050										1,050								
Group B Streptococcus	16,100								10,000		10,300				257				
Haemophilus influenzae	3,670		2,880				1,500												
Klebsiella pneumoniae	64,700	43,000			60,100	1,600	54,700		50,000								61,700		
Morganella spp.	33.7						7.64	20	25										
Mycobacterium tuberculosis	7,040									2,250			4,730			2.19			63
Neisseria gonorrhoeae	85.4						0.163		85.2										
Proteus spp.	2,360	325	2,090				576		846								1,760		
Pseudomonas aeruginosa	18,700	7,880		10,400		7,820	10,700	7,790	11,200										
Salmonella Paratyphi	2.3								1.56			0.767							
Salmonella Typhi	19,400								5,120			16,500							
Non−typhoidal Salmonella	29.7								29.7										
Serratia spp.	5,340	2,260		589		493	4,290	4,490	1,350										
Shigella spp.	803								803										
Staphylococcus aureus	50,700								9,380		28,500			25,800			34,500	1,770	
Streptococcus pneumoniae	57,900				12,400	13,200	8,330		6,700		13,900				9,040		55,700		
All pathogens	340,000	92,200	56,000	28,100	118,000	35,400	115,000	30,100	149,000	2,250	53,800	16,500	4,730	25,800	9,300	2.19	209,000	3,500	63

Counts
- <2.5k
- 2.5k − <5k
- 5k − <10k
- 10k − <25k
- 25k − <75k
- 75k − <150k
- >=150k

Togo

DALYs (count) *attributable to* bacterial antimicrobial resistance by pathogen−drug combinations, 2019

	Resistance to 1+	AG	Aminopenicillin	Anti−pseudomonal	BL−BLI	CP	3GC	4GC	FQ	Mono INH	Macrolide	MDR in S. Typhi and Paratyphi	MDR excluding XDR in TB	Methicillin	PCN	Mono RIF	TMP−SMX	Vanco	XDR in TB
Acinetobacter baumannii	3,710	301		1,850	0.083	749	15.4	0.397	789										
Citrobacter spp.	497	30.8		127		56.5	53.6	146	83.7										
Enterobacter spp.	2,400	202		282		316		554	666								374		
Enterococcus faecalis	1,470								1,370									98.8	
Enterococcus faecium	1,350								1,050									293	
Other enterococci	563								537									26.3	
Escherichia coli	13,000	1,740	659		1,560	1,230	2,790		2,360								2,700		
Group A Streptococcus	98.9										101								
Group B Streptococcus	2,780								1,780		919				64.4				
Haemophilus influenzae	843		411				432												
Klebsiella pneumoniae	17,100	2,960			704	514	7,120		3,070								2,760		
Morganella spp.	7.78						0.325	3.37	4.08										
Mycobacterium tuberculosis	3,040									345			2,660			0.59			37.4
Neisseria gonorrhoeae	8.32						0.079		8.24										
Proteus spp.	312	16.5	42.3				125		59								70.3		
Pseudomonas aeruginosa	4,570	212		1,170		1,300	478	148	1,250										
Salmonella Paratyphi	0.436								0.332			0.106							
Salmonella Typhi	2,970								917			2,180							
Non−typhoidal Salmonella	5.97								5.97										
Serratia spp.	1,410	126		124		131	173	723	133										
Shigella spp.	166								166										
Staphylococcus aureus	11,400								377		1,020			5,990			3,590	432	
Streptococcus pneumoniae	10,700				361	2,870	356		813		509				237		5,520		
All pathogens	78,500	5,590	1,110	3,550	2,620	7,180	11,500	1,580	15,500	342	2,560	2,030	2,660	5,990	301	0.59	15,000	850	37.4

Counts
- <2.5k
- 2.5k − <5k
- 5k − <10k
- 10k − <25k
- 75k − <150k

Uganda

DALYs (count) *associated with* bacterial antimicrobial resistance by pathogen−drug combinations, 2019

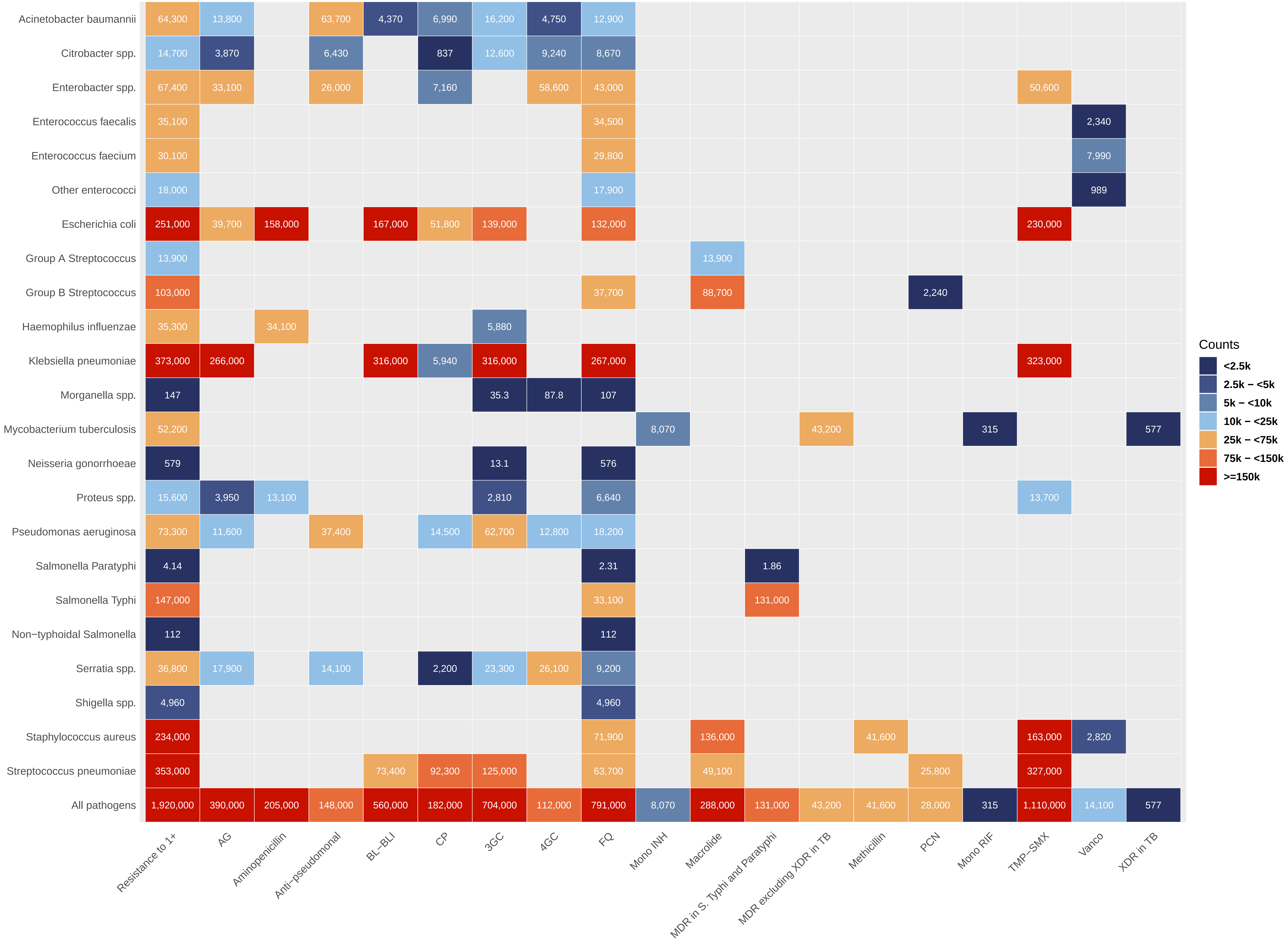

Uganda

DALYs (count) *attributable to* bacterial antimicrobial resistance by pathogen−drug combinations, 2019

	Resistance to 1+	AG	Aminopenicillin	Anti−pseudomonal	BL−BLI	CP	3GC	4GC	FQ	Mono INH	Macrolide	MDR in S. Typhi and Paratyphi	MDR excluding XDR in TB	Methicillin	PCN	Mono RIF	TMP−SMX	Vanco	XDR in TB
Acinetobacter baumannii	16,400	838		11,700	0.802	1,740	4.11	2.63	2,030										
Citrobacter spp.	4,300	175		1,160		189	703	854	1,210										
Enterobacter spp.	16,000	1,710		2,210		1,450		4,340	4,080								2,180		
Enterococcus faecalis	9,260								8,600									661	
Enterococcus faecium	7,640								5,730									1,910	
Other enterococci	3,710								3,510									203	
Escherichia coli	64,300	2,360	336		4,610	11,400	16,500		13,200								15,900		
Group A Streptococcus	1,360										1,420								
Group B Streptococcus	15,200								6,230		8,600				467				
Haemophilus influenzae	7,110		5,470				1,640												
Klebsiella pneumoniae	98,200	18,900			3,810	2,550	41,800		16,400								14,700		
Morganella spp.	34.1						1.63	15	17.4										
Mycobacterium tuberculosis	26,500									1,190			24,800			85.1			346
Neisseria gonorrhoeae	59						3.07		55.9										
Proteus spp.	2,000	183	249				577		461								531		
Pseudomonas aeruginosa	19,800	358		6,630		2,940	7,670	141	2,090										
Salmonella Paratyphi	0.74								0.49			0.251							
Salmonella Typhi	22,000								5,700			16,300							
Non−typhoidal Salmonella	22.3								22.3										
Serratia spp.	8,950	1,100		2,770		653	797	2,780	915										
Shigella spp.	1,010								1,010										
Staphylococcus aureus	37,100								2,860		5,160			9,860			18,300	879	
Streptococcus pneumoniae	72,400				759	19,900	10,400		7,680		1,830				91.6		31,700		
All pathogens	433,000	25,600	6,050	24,500	9,180	40,900	80,000	8,130	81,800	1,210	16,900	16,000	24,800	9,860	558	85.1	83,300	3,650	346

Counts
- <2.5k
- 2.5k − <5k
- 5k − <10k
- 10k − <25k
- 25k − <75k
- 75k − <150k
- >=150k

Zambia

DALYs (count) *associated with* bacterial antimicrobial resistance by pathogen−drug combinations, 2019

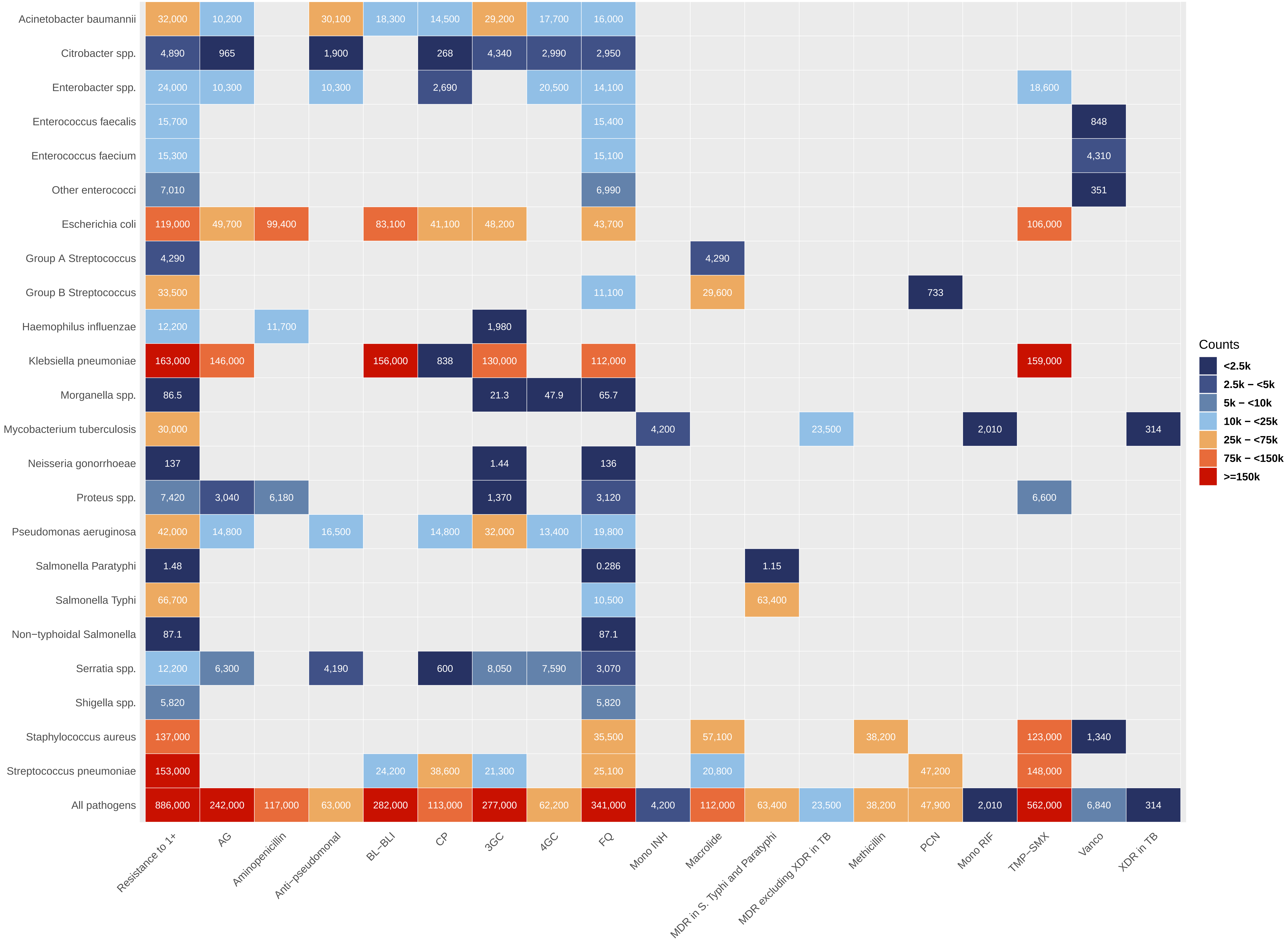

Zambia

DALYs (count) *attributable to* bacterial antimicrobial resistance by pathogen−drug combinations, 2019

	Resistance to 1+	AG	Aminopenicillin	Anti−pseudomonal	BL−BLI	CP	3GC	4GC	FQ	Mono INH	Macrolide	MDR in S. Typhi and Paratyphi	MDR excluding XDR in TB	Methicillin	PCN	Mono RIF	TMP−SMX	Vanco	XDR in TB
Acinetobacter baumannii	9,720	495		3,980	2.54	2,590	545	0.776	2,100										
Citrobacter spp.	1,440	43.3		346		60.5	277	297	412										
Enterobacter spp.	5,660	533		936		562		1,430	1,340								859		
Enterococcus faecalis	4,100								3,860									239	
Enterococcus faecium	3,900								2,880									1,020	
Other enterococci	1,440								1,370									72.8	
Escherichia coli	31,400	3,170	934		2,780	9,490	4,250		3,900								6,850		
Group A Streptococcus	402										375								
Group B Streptococcus	4,820								1,810		2,790				152				
Haemophilus influenzae	2,460		1,900				557												
Klebsiella pneumoniae	42,400	10,400			2,230	447	16,200		6,520								6,560		
Morganella spp.	20.1						1.12	8.07	10.9										
Mycobacterium tuberculosis	14,600									633			13,300			542			187
Neisseria gonorrhoeae	13.5						0.411		13.1										
Proteus spp.	975	137	104				272		208								244		
Pseudomonas aeruginosa	10,700	400		1,880		2,610	3,180	395	2,240										
Salmonella Paratyphi	0.23								0.075			0.151							
Salmonella Typhi	9,350								1,660			7,890							
Non−typhoidal Salmonella	17.4								17.4										
Serratia spp.	2,940	370		847		182	388	842	308										
Shigella spp.	1,190								1,190										
Staphylococcus aureus	25,200								1,300		1,870			7,650			14,000	404	
Streptococcus pneumoniae	29,700				550	8,280	688		2,900		739				2,190		14,300		
All pathogens	203,000	15,600	2,950	7,980	5,560	24,200	26,300	2,970	34,000	581	5,900	7,910	13,300	7,650	2,340	542	42,800	1,740	187

Counts

- **<2.5k**
- **2.5k − <5k**
- **5k − <10k**
- **10k − <25k**
- **25k − <75k**
- **>=150k**

Zimbabwe

DALYs (count) *associated with* bacterial antimicrobial resistance by pathogen−drug combinations, 2019

	Resistance to 1+	AG	Aminopenicillin	Anti−pseudomonal	BL−BLI	CP	3GC	4GC	FQ	Mono INH	Macrolide	MDR in S. Typhi and Paratyphi	MDR excluding XDR in TB	Methicillin	PCN	Mono RIF	TMP−SMX	Vanco	XDR in TB
Acinetobacter baumannii	35,600	11,800		33,200	19,800	16,800	32,700	24,400	22,700										
Citrobacter spp.	2,670	374		1,230		405	1,920	1,640	917										
Enterobacter spp.	19,800	4,940		5,720		3,670		11,000	6,670								16,800		
Enterococcus faecalis	9,790								9,560									756	
Enterococcus faecium	11,300								11,200									1,990	
Other enterococci	4,840								4,820									405	
Escherichia coli	105,000	15,200	98,000		61,900	9,050	54,700		41,600								91,900		
Group A Streptococcus	1,880										1,880								
Group B Streptococcus	38,300								8,140		35,200				1,940				
Haemophilus influenzae	17,400		16,700				3,210												
Klebsiella pneumoniae	157,000	66,800			138,000	1,840	135,000		79,500								140,000		
Morganella spp.	67.1						9.78	37.5	50.2										
Mycobacterium tuberculosis	65,800									18,800			46,000			251			621
Neisseria gonorrhoeae	167						0.144		166										
Proteus spp.	5,340	705	4,770				1,150		620								4,100		
Pseudomonas aeruginosa	43,700	9,390		30,500		21,200	26,700	18,700	18,600										
Salmonella Paratyphi	8.48								8.21			0.351							
Salmonella Typhi	23,000								6,160			18,500							
Non−typhoidal Salmonella	127								127										
Serratia spp.	6,370	3,870		1,040		642	2,000	3,720	1,380										
Shigella spp.	609								609										
Staphylococcus aureus	105,000								25,900		49,900			26,600			74,500	1,380	
Streptococcus pneumoniae	172,000				51,000	51,100	26,000		19,700		57,000				68,400		157,000		
All pathogens	826,000	113,000	120,000	71,700	271,000	105,000	283,000	59,600	258,000	18,800	144,000	18,500	46,000	26,600	70,300	251	485,000	4,540	621

Counts
- **<2.5k**
- **2.5k − <5k**
- **5k − <10k**
- **10k − <25k**
- **25k − <75k**
- **75k − <150k**
- **>=150k**

Zimbabwe

DALYs (count) *attributable to* bacterial antimicrobial resistance by pathogen−drug combinations, 2019

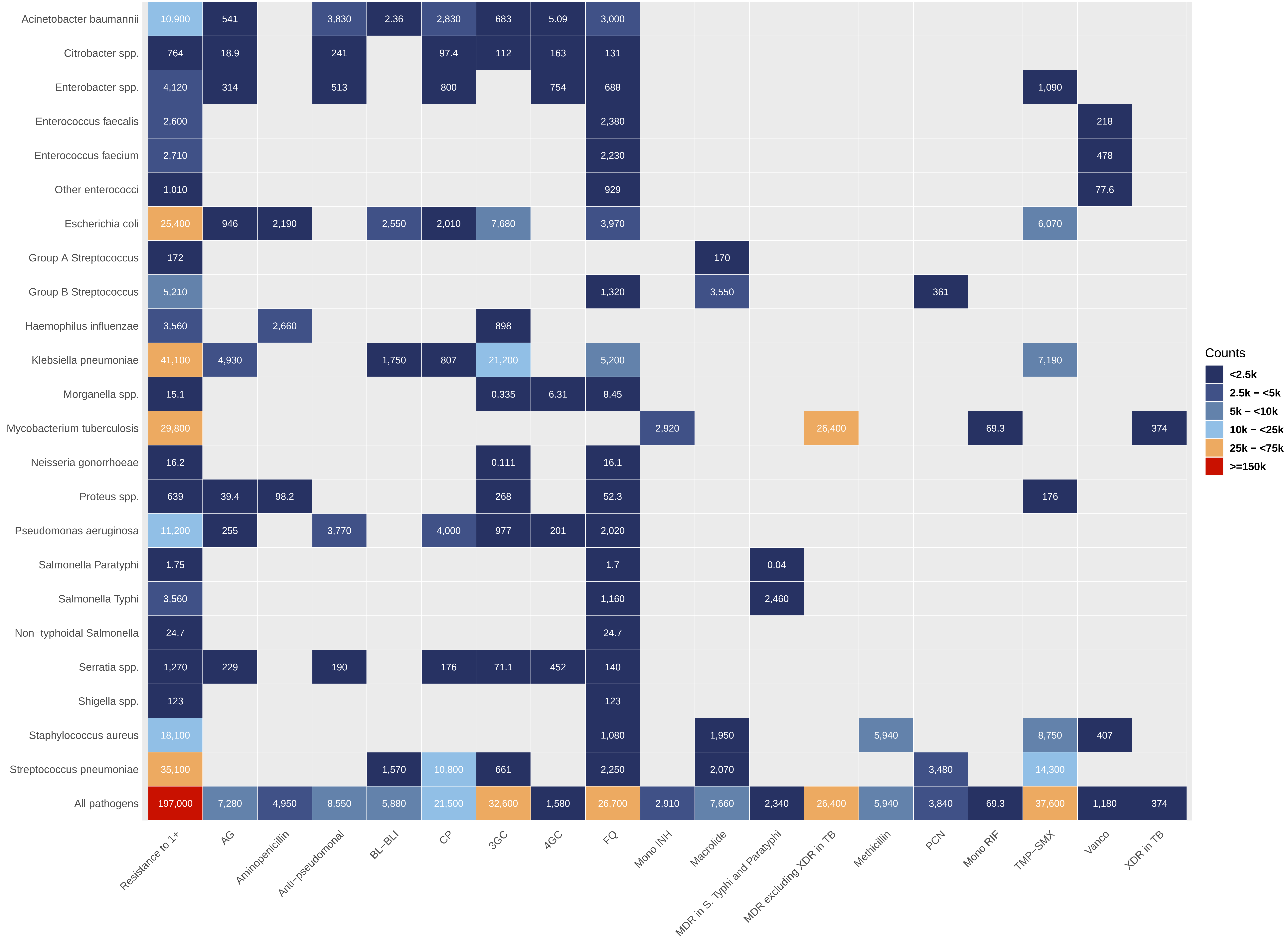

African Region

DALYs (count) *associated with* bacterial antimicrobial resistance by pathogen−drug combinations, 2019

	Resistance to 1+	AG	Aminopenicillin	Anti−pseudomonal	BL−BLI	CP	3GC	4GC	FQ	Mono INH	Macrolide	MDR in S. Typhi and Paratyphi	MDR excluding XDR in TB	Methicillin	PCN	Mono RIF	TMP−SMX	Vanco	XDR in TB
Acinetobacter baumannii	2,090,000	904,000		2,000,000	1,230,000	861,000	1,590,000	1,220,000	1,160,000										
Citrobacter spp.	381,000	126,000		166,000		72,200	330,000	281,000	178,000										
Enterobacter spp.	2,040,000	996,000		800,000		329,000		1,680,000	1,190,000								1,620,000		
Enterococcus faecalis	937,000								923,000									60,700	
Enterococcus faecium	840,000								834,000									210,000	
Other enterococci	491,000								490,000									30,800	
Escherichia coli	8,760,000	3,010,000	8,190,000		6,480,000	946,000	4,960,000		5,000,000								7,840,000		
Group A Streptococcus	238,000										238,000								
Group B Streptococcus	4,060,000								2,320,000		2,890,000				91,900				
Haemophilus influenzae	984,000		837,000				327,000												
Klebsiella pneumoniae	11,600,000	8,120,000			10,700,000	1,340,000	9,780,000		8,110,000								10,700,000		
Morganella spp.	5,100						1,210	2,870	3,880										
Mycobacterium tuberculosis	1,850,000									596,000			1,060,000			177,000			14,200
Neisseria gonorrhoeae	10,700						140		10,700										
Proteus spp.	410,000	122,000	356,000				96,400		187,000								333,000		
Pseudomonas aeruginosa	3,310,000	1,480,000		1,600,000		1,320,000	2,230,000	1,470,000	1,700,000										
Salmonella Paratyphi	7,020								5,710			1,490							
Salmonella Typhi	3,290,000								1,260,000			2,710,000							
Non−typhoidal Salmonella	99,700								99,700										
Serratia spp.	907,000	457,000		197,000		54,300	601,000	714,000	223,000										
Shigella spp.	799,000								799,000										
Staphylococcus aureus	7,580,000								1,980,000		4,110,000			3,710,000			5,220,000	123,000	
Streptococcus pneumoniae	13,600,000				2,700,000	3,130,000	1,970,000		2,950,000		3,240,000				4,460,000		12,600,000		
All pathogens	64,300,000	15,200,000	9,380,000	4,750,000	21,100,000	8,050,000	21,900,000	5,370,000	29,400,000	596,000	10,500,000	2,710,000	1,060,000	3,710,000	4,550,000	177,000	38,400,000	424,000	14,200

Counts
- **<50k**
- **50k − <100k**
- **100k − <500k**
- **500k − <1M**
- **1M − <1.5M**
- **1.5M − <2M**
- **>= 2M**

African Region

DALYs (count) *attributable to* bacterial antimicrobial resistance by pathogen−drug combinations, 2019

	Resistance to 1+	AG	Aminopenicillin	Anti−pseudomonal	BL−BLI	CP	3GC	4GC	FQ	Mono INH	Macrolide	MDR in S. Typhi and Paratyphi	MDR excluding XDR in TB	Methicillin	PCN	Mono RIF	TMP−SMX	Vanco	XDR in TB
Acinetobacter baumannii	620,000	44,000		253,000	1,630	148,000	19,700	1,530	152,000										
Citrobacter spp.	113,000	6,560		27,000		15,600	14,000	26,400	23,400										
Enterobacter spp.	484,000	53,400		64,700		65,800		115,000	110,000								74,800		
Enterococcus faecalis	247,000								230,000									16,600	
Enterococcus faecium	211,000								161,000									49,500	
Other enterococci	101,000								95,000									5,980	
Escherichia coli	2,210,000	174,000	98,100		215,000	190,000	603,000		470,000								462,000		
Group A Streptococcus	21,400										23,000								
Group B Streptococcus	682,000								403,000		256,000				19,000				
Haemophilus influenzae	217,000		124,000				93,400												
Klebsiella pneumoniae	3,170,000	578,000			133,000	293,000	1,190,000		499,000								483,000		
Morganella spp.	1,180						59.9	481	642										
Mycobacterium tuberculosis	748,000									87,000			602,000			47,500			8,470
Neisseria gonorrhoeae	1,060						32.9		1,030										
Proteus spp.	56,200	5,450	6,420				19,400		12,500								12,700		
Pseudomonas aeruginosa	824,000	41,500		176,000		225,000	151,000	43,600	186,000										
Salmonella Paratyphi	1,360								1,170			192							
Salmonella Typhi	534,000								213,000			324,000							
Non−typhoidal Salmonella	20,300								20,300										
Serratia spp.	223,000	26,400		39,300		15,800	19,400	101,000	22,000										
Shigella spp.	165,000								165,000										
Staphylococcus aureus	1,660,000								80,800		145,000			864,000			541,000	33,000	
Streptococcus pneumoniae	2,710,000				75,100	665,000	92,700		355,000		112,000				255,000		1,160,000		
All pathogens	15,000,000	929,000	228,000	560,000	425,000	1,620,000	2,200,000	287,000	3,200,000	90,600	540,000	322,000	602,000	864,000	274,000	47,500	2,730,000	105,000	8,470

Counts

- **<50k**
- **50k − <100k**
- **100k − <500k**
- **500k − <1M**
- **1M − <1.5M**
- **1.5M − <2M**
- **>= 2M**

Central sub−Saharan Africa

DALYs (count) *associated with* bacterial antimicrobial resistance by pathogen−drug combinations, 2019

	Resistance to 1+	AG	Aminopenicillin	Anti−pseudomonal	BL−BLI	CP	3GC	4GC	FQ	Mono INH	Macrolide	MDR in S. Typhi and Paratyphi	MDR excluding XDR in TB	Methicillin	PCN	Mono RIF	TMP−SMX	Vanco	XDR in TB
Acinetobacter baumannii	233,000	81,100		226,000	142,000	90,100	186,000	115,000	133,000										
Citrobacter spp.	34,200	9,110		17,500		4,220	26,000	24,400	14,900										
Enterobacter spp.	200,000	84,800		78,800		38,500		166,000	106,000								148,000		
Enterococcus faecalis	99,900								98,200									8,120	
Enterococcus faecium	84,500								84,000									21,100	
Other enterococci	44,500								44,300									3,110	
Escherichia coli	821,000	272,000	732,000		592,000	90,900	390,000		457,000								704,000		
Group A Streptococcus	29,800										29,800								
Group B Streptococcus	480,000								276,000		350,000				24,500				
Haemophilus influenzae	136,000		130,000				33,700												
Klebsiella pneumoniae	1,100,000	789,000			992,000	29,100	884,000		755,000								985,000		
Morganella spp.	653						158	409	480										
Mycobacterium tuberculosis	425,000									215,000			168,000			39,100			2,250
Neisseria gonorrhoeae	1,510						45.9		1,510										
Proteus spp.	44,000	11,800	39,500				10,600		19,100								34,500		
Pseudomonas aeruginosa	304,000	95,000		186,000		119,000	219,000	153,000	134,000										
Salmonella Paratyphi	14.4								9.65			4.93							
Salmonella Typhi	346,000								237,000			221,000							
Non−typhoidal Salmonella	23,800								23,800										
Serratia spp.	86,500	38,200		17,000		7,890	44,500	72,600	18,400										
Shigella spp.	8,160								8,160										
Staphylococcus aureus	654,000								275,000		286,000			288,000			452,000	7,520	
Streptococcus pneumoniae	1,360,000				539,000	260,000	146,000		723,000		455,000				486,000		1,220,000		
All pathogens	6,520,000	1,380,000	901,000	526,000	2,260,000	640,000	1,940,000	532,000	3,410,000	215,000	1,120,000	221,000	168,000	288,000	511,000	39,100	3,540,000	39,900	2,250

Counts
- <25k
- 25k − <50k
- 50k − <100k
- 100k − <200k
- 200k − <500k
- 500k − <1M
- >=1M

Central sub−Saharan Africa

DALYs (count) *attributable to* bacterial antimicrobial resistance by pathogen−drug combinations, 2019

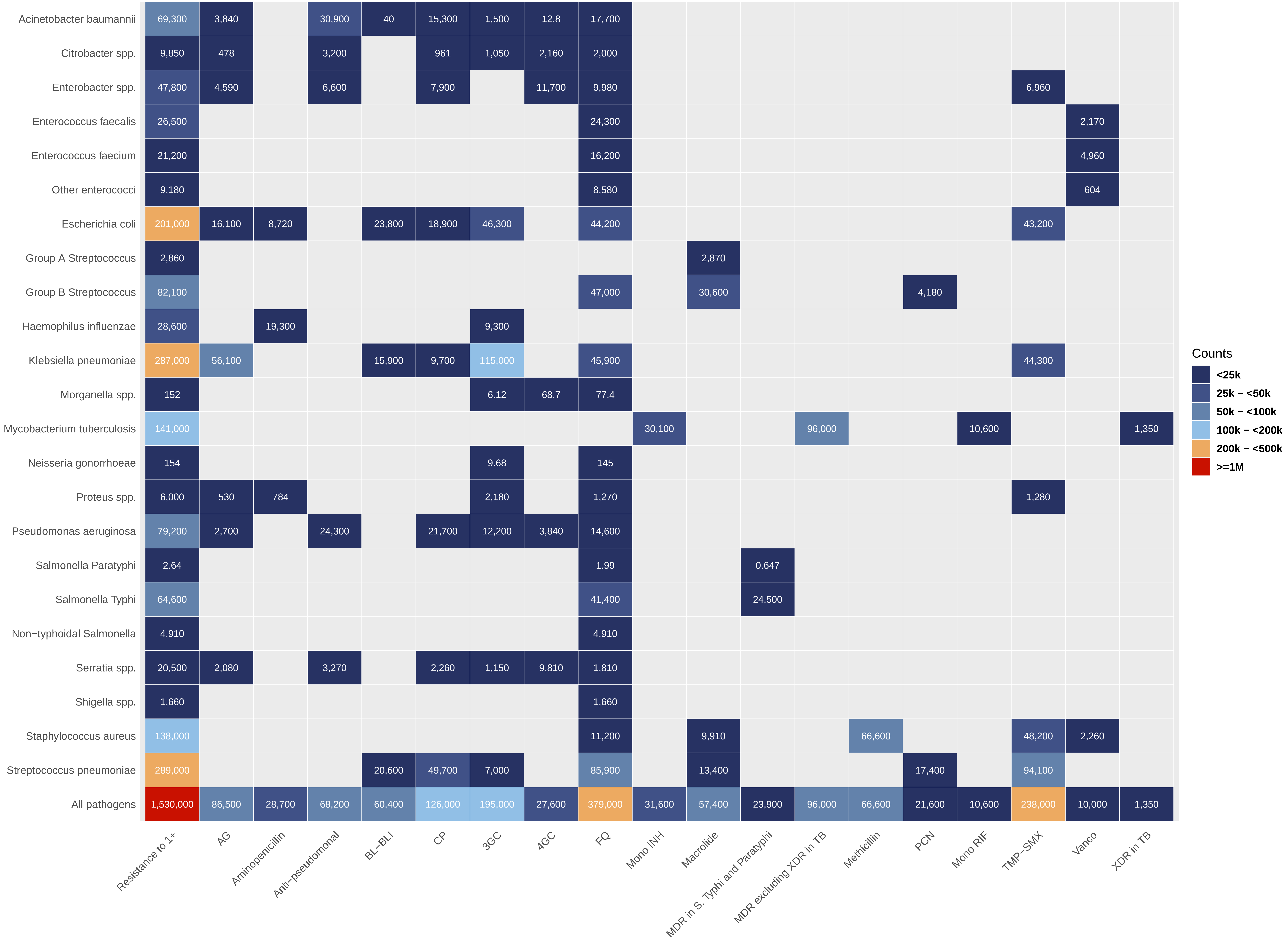

Eastern sub−Saharan Africa

DALYs (count) *associated with* bacterial antimicrobial resistance by pathogen−drug combinations, 2019

	Resistance to 1+	AG	Aminopenicillin	Anti−pseudomonal	BL−BLI	CP	3GC	4GC	FQ	Mono INH	Macrolide	MDR in S. Typhi and Paratyphi	MDR excluding XDR in TB	Methicillin	PCN	Mono RIF	TMP−SMX	Vanco	XDR in TB
Acinetobacter baumannii	716,000	249,000		645,000	508,000	252,000	606,000	409,000	406,000										
Citrobacter spp.	123,000	38,100		53,600		16,900	106,000	77,100	75,100										
Enterobacter spp.	604,000	272,000		235,000		76,100		534,000	351,000								453,000		
Enterococcus faecalis	352,000								348,000									21,900	
Enterococcus faecium	283,000								280,000									74,600	
Other enterococci	149,000								148,000									8,980	
Escherichia coli	2,610,000	601,000	2,380,000		1,780,000	261,000	1,320,000		1,280,000								2,240,000		
Group A Streptococcus	108,000										108,000								
Group B Streptococcus	1,040,000								439,000		870,000				23,400				
Haemophilus influenzae	335,000		321,000				63,600												
Klebsiella pneumoniae	3,540,000	2,570,000			3,280,000	379,000	2,950,000		2,260,000								3,190,000		
Morganella spp.	1,800						424	1,070	1,340										
Mycobacterium tuberculosis	601,000									96,400			439,000			59,300			5,860
Neisseria gonorrhoeae	3,250						78.8		3,230										
Proteus spp.	151,000	55,400	129,000				28,300		78,700								132,000		
Pseudomonas aeruginosa	910,000	269,000		362,000		244,000	715,000	299,000	406,000										
Salmonella Paratyphi	331								55.6			273							
Salmonella Typhi	1,270,000								526,000			980,000							
Non−typhoidal Salmonella	38,800								38,800										
Serratia spp.	279,000	135,000		102,000		15,300	172,000	190,000	75,400										
Shigella spp.	107,000								107,000										
Staphylococcus aureus	2,390,000								884,000		1,200,000			923,000			1,700,000	27,700	
Streptococcus pneumoniae	3,760,000				577,000	950,000	546,000		976,000		509,000				987,000		3,570,000		
All pathogens	19,400,000	4,190,000	2,830,000	1,400,000	6,150,000	2,190,000	6,510,000	1,510,000	8,690,000	96,400	2,690,000	980,000	439,000	923,000	1,010,000	59,300	11,300,000	133,000	5,860

Counts
- **<25k**
- **25k − <50k**
- **50k − <100k**
- **100k − <200k**
- **200k − <500k**
- **500k − <1M**
- **>=1M**

Eastern sub−Saharan Africa

DALYs (count) *attributable to* bacterial antimicrobial resistance by pathogen−drug combinations, 2019

	Resistance to 1+	AG	Aminopenicillin	Anti−pseudomonal	BL−BLI	CP	3GC	4GC	FQ	Mono INH	Macrolide	MDR in S. Typhi and Paratyphi	MDR excluding XDR in TB	Methicillin	PCN	Mono RIF	TMP−SMX	Vanco	XDR in TB
Acinetobacter baumannii	213,000	11,900		86,200	1,550	43,000	14,700	1,420	54,500										
Citrobacter spp.	36,100	1,820		8,640		3,260	5,640	6,440	10,400										
Enterobacter spp.	144,000	14,200		20,700		15,600		40,800	32,400								20,700		
Enterococcus faecalis	92,600								86,700									5,900	
Enterococcus faecium	71,700								53,900									17,700	
Other enterococci	30,700								28,900									1,800	
Escherichia coli	639,000	36,100	37,900		72,600	57,500	172,000		124,000								140,000		
Group A Streptococcus	9,640										10,000								
Group B Streptococcus	159,000								72,600		83,200				4,790				
Haemophilus influenzae	68,400		50,600				17,800												
Klebsiella pneumoniae	962,000	186,000			44,600	81,900	369,000		138,000								143,000		
Morganella spp.	418						18.5	179	220										
Mycobacterium tuberculosis	284,000									13,700			250,000			16,000			3,510
Neisseria gonorrhoeae	329						17.3		312										
Proteus spp.	20,300	2,430	2,480				5,540		5,210								4,740		
Pseudomonas aeruginosa	235,000	7,630		50,000		43,600	75,600	11,500	46,700										
Salmonella Paratyphi	50								13.2			35.7							
Salmonella Typhi	208,000								92,600			118,000							
Non−typhoidal Salmonella	7,810								7,810										
Serratia spp.	67,100	7,960		20,200		4,580	6,400	20,600	7,570										
Shigella spp.	21,900								21,900										
Staphylococcus aureus	477,000								35,100		42,400			211,000			181,000	7,930	
Streptococcus pneumoniae	747,000				12,100	198,000	22,100		119,000		19,200				44,600		332,000		
All pathogens	4,500,000	268,000	90,900	186,000	131,000	448,000	688,000	80,800	938,000	14,400	152,000	115,000	250,000	211,000	49,400	16,000	821,000	33,400	3,510

Counts

- **<25k**
- **25k − <50k**
- **50k − <100k**
- **100k − <200k**
- **200k − <500k**
- **500k − <1M**
- **>=1M**

North Africa and Middle East

DALYs (count) *associated with* bacterial antimicrobial resistance by pathogen−drug combinations, 2019

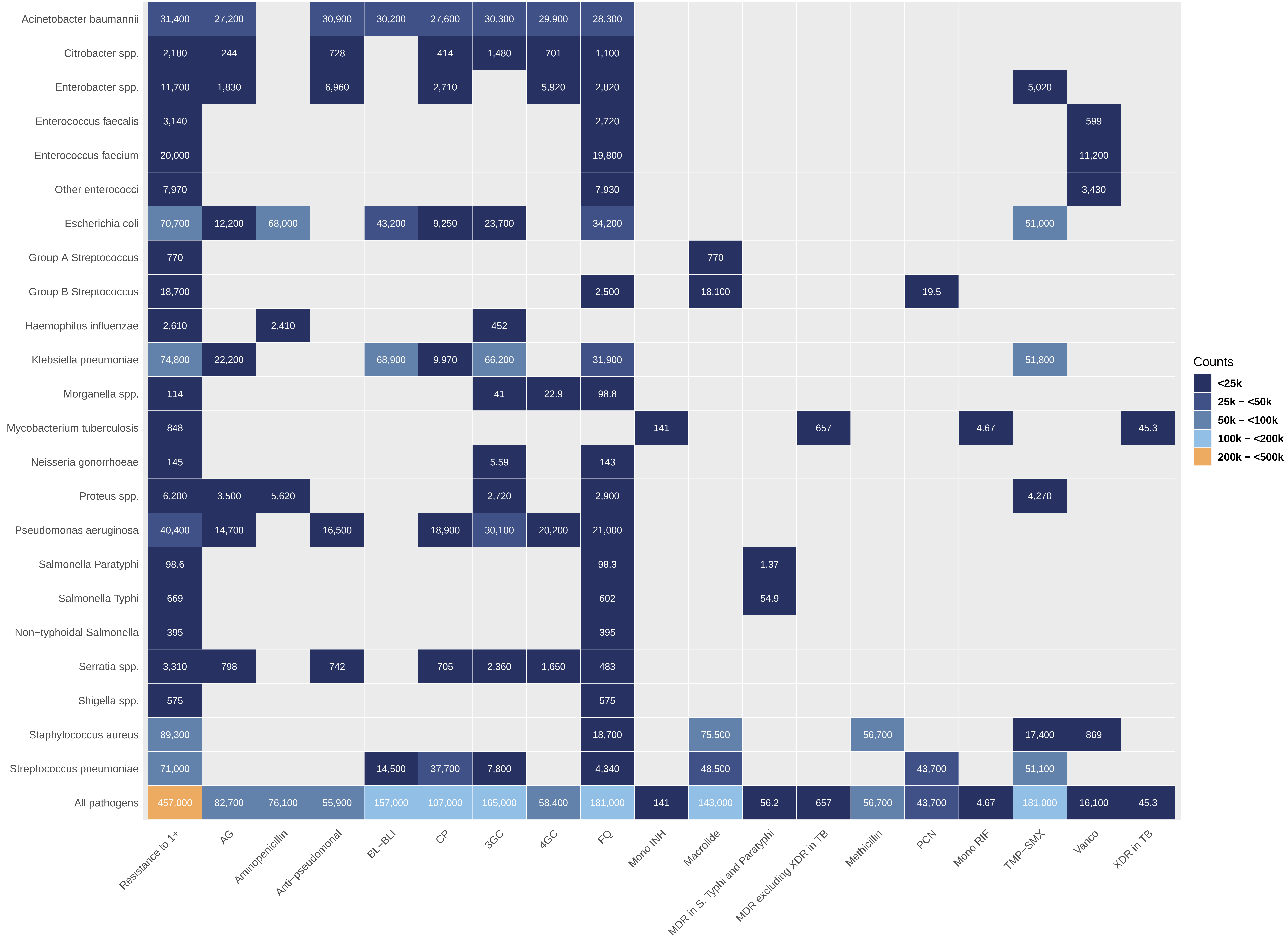

North Africa and Middle East

DALYs (count) *attributable to* bacterial antimicrobial resistance by pathogen−drug combinations, 2019

	Resistance to 1+	AG	Aminopenicillin	Anti−pseudomonal	BL−BLI	CP	3GC	4GC	FQ	Mono INH	Macrolide	MDR in S. Typhi and Paratyphi	MDR excluding XDR in TB	Methicillin	PCN	Mono RIF	TMP−SMX	Vanco	XDR in TB
Acinetobacter baumannii	10,000	1,160		829	16.7	4,500	66.3	12	3,470										
Citrobacter spp.	627	12.4		134		98.9	141	56.2	184										
Enterobacter spp.	2,690	119		918		713		325	303								314		
Enterococcus faecalis	894								675									219	
Enterococcus faecium	5,850								3,280									2,570	
Other enterococci	1,880								1,300									578	
Escherichia coli	16,800	742	2,050		2,480	2,110	2,850		3,450								3,080		
Group A Streptococcus	69.7										71.9								
Group B Streptococcus	2,170								389		1,780				18.4				
Haemophilus influenzae	532		399				132												
Klebsiella pneumoniae	21,000	1,820			1,080	2,490	10,600		2,330								2,640		
Morganella spp.	26.7						5.43	4.05	17.2										
Mycobacterium tuberculosis	380									18.3			335			1.16			25.3
Neisseria gonorrhoeae	15						1.24		13.8										
Proteus spp.	1,060	165	76.6				508		173								139		
Pseudomonas aeruginosa	10,400	390		1,430		3,320	2,190	792	2,280										
Salmonella Paratyphi	19.9								19.8			0.125							
Salmonella Typhi	134								125			9.63							
Non−typhoidal Salmonella	55.4								55.4										
Serratia spp.	871	47		162		196	221	191	52.7										
Shigella spp.	89.5								89.5										
Staphylococcus aureus	20,600								874		3,110			14,800			1,600	255	
Streptococcus pneumoniae	16,600				57.6	8,190	22.1		429		1,770				2,140		3,970		
All pathogens	113,000	4,470	2,520	3,480	3,640	21,600	16,800	1,380	19,500	18.7	6,840	9.71	335	14,800	2,150	1.16	11,700	3,630	25.3

Counts
- <25k
- 100k − <200k

Southeast Asia

DALYs (count) *associated with* bacterial antimicrobial resistance by pathogen−drug combinations, 2019

	Resistance to 1+	AG	Aminopenicillin	Anti−pseudomonal	BL−BLI	CP	3GC	4GC	FQ	Mono INH	Macrolide	MDR in S. Typhi and Paratyphi	MDR excluding XDR in TB	Methicillin	PCN	Mono RIF	TMP−SMX	Vanco	XDR in TB
Acinetobacter baumannii	2,060	1,580		1,970	1,570	1,610	1,830	1,960	1,730										
Citrobacter spp.	142	18		18.6		25.9	88.1	87.3	58										
Enterobacter spp.	747	124		355		206		310	199								472		
Enterococcus faecalis	572								545									71.9	
Enterococcus faecium	1,200								1,190									275	
Other enterococci	491								488									172	
Escherichia coli	3,710	480	3,550		2,780	494	1,750		2,150								2,520		
Group A Streptococcus	202										202								
Group B Streptococcus	426								114		358				37.2				
Haemophilus influenzae	114		104				26.2												
Klebsiella pneumoniae	2,740	351			1,970	516	2,110		1,380								2,030		
Morganella spp.	8.52						5.03	1.85	5.11										
Mycobacterium tuberculosis	27.2									19			6.32			0.746			1.1
Neisseria gonorrhoeae	11.8						0.09		11.8										
Proteus spp.	433	299	350				161		169								244		
Pseudomonas aeruginosa	1,520	449		573		1,070	773	582	671										
Salmonella Paratyphi	28.1								27.5			0.611							
Salmonella Typhi	57.9								52.2			6.23							
Non−typhoidal Salmonella	6.83								6.83										
Serratia spp.	148	22.2		46.2		32.5	102	49.1	46.7										
Shigella spp.	3.34								3.34										
Staphylococcus aureus	4,290								1,230		2,290			3,910			1,080	84.3	
Streptococcus pneumoniae	2,260				382	1,110	497		98		1,360				747		1,660		
All pathogens	21,200	3,320	4,000	2,970	6,710	5,070	7,340	3,000	10,200	19	4,210	6.84	6.32	3,910	785	0.746	8,000	604	1.1

Counts

<25k

Southeast Asia

DALYs (count) *attributable to* bacterial antimicrobial resistance by pathogen−drug combinations, 2019

	Resistance to 1+	AG	Aminopenicillin	Anti−pseudomonal	BL−BLI	CP	3GC	4GC	FQ	Mono INH	Macrolide	MDR in S. Typhi and Paratyphi	MDR excluding XDR in TB	Methicillin	PCN	Mono RIF	TMP−SMX	Vanco	XDR in TB
Acinetobacter baumannii	651	69.4		81.3	0.329	265	10.5	10.1	214										
Citrobacter spp.	39.7	0.951		3.59		6.18	8.44	11.7	8.9										
Enterobacter spp.	166	8.24		39.7		52.9		12.8	21.2								31.2		
Enterococcus faecalis	156								134									21.6	
Enterococcus faecium	298								233									64.9	
Other enterococci	113								83.7									28.9	
Escherichia coli	931	29.1	55.8		137	110	223		228								148		
Group A Streptococcus	19.4										18.2								
Group B Streptococcus	62.1								19.6		32.3				6.82				
Haemophilus influenzae	23.9		16.4				7.48												
Klebsiella pneumoniae	744	35.7			38.8	134	308		108								120		
Morganella spp.	2.17						0.898	0.411	0.866										
Mycobacterium tuberculosis	6.2									2.43			3			0.181			0.593
Neisseria gonorrhoeae	1.16						0.024		1.14										
Proteus spp.	68.8	16.1	5.01				29.8		10								7.73		
Pseudomonas aeruginosa	374	12.7		30.4		204	42.4	13.8	70.8										
Salmonella Paratyphi	5.75								5.66			0.094							
Salmonella Typhi	11.6								10.8			0.81							
Non−typhoidal Salmonella	1.27								1.27										
Serratia spp.	38.9	1.31		9.65		8.94	8.95	4.59	5.44										
Shigella spp.	0.551								0.551										
Staphylococcus aureus	1,310								55		79.5			1,060			89.9	19	
Streptococcus pneumoniae	495				1.36	253	9.9		10.8		55.7				20.2		144		
All pathogens	5,510	173	77.3	165	177	1,030	649	53.4	1,220	2.26	189	0.895	3	1,060	27.1	0.181	541	134	0.593

Counts

<25k

Southern sub−Saharan Africa

DALYs (count) *associated with* bacterial antimicrobial resistance by pathogen−drug combinations, 2019

	Resistance to 1+	AG	Aminopenicillin	Anti−pseudomonal	BL−BLI	CP	3GC	4GC	FQ	Mono INH	Macrolide	MDR in S. Typhi and Paratyphi	MDR excluding XDR in TB	Methicillin	PCN	Mono RIF	TMP−SMX	Vanco	XDR in TB
Acinetobacter baumannii	153,000	71,800		146,000	98,000	103,000	146,000	126,000	128,000										
Citrobacter spp.	8,340	779		2,980		2,520	5,120	3,560	2,830										
Enterobacter spp.	79,900	7,910		14,400		15,600		19,400	14,000								72,100		
Enterococcus faecalis	34,600								33,400									3,000	
Enterococcus faecium	59,400								59,200									6,040	
Other enterococci	30,000								29,900									3,020	
Escherichia coli	335,000	26,500	304,000		179,000	16,400	130,000		124,000								289,000		
Group A Streptococcus	4,380										4,380								
Group B Streptococcus	108,000								16,100		102,000				3,300				
Haemophilus influenzae	29,200		26,900				6,220												
Klebsiella pneumoniae	475,000	99,400			400,000	51,700	415,000		196,000								423,000		
Morganella spp.	283						32.5	62.4	256										
Mycobacterium tuberculosis	236,000									92,200			118,000			24,900			1,560
Neisseria gonorrhoeae	1,260						1.01		1,250										
Proteus spp.	24,700	1,100	22,200				5,300		2,030								18,600		
Pseudomonas aeruginosa	151,000	20,700		79,900		102,000	67,400	64,600	70,200										
Salmonella Paratyphi	33.6								33.1			0.592							
Salmonella Typhi	46,000								15,200			34,000							
Non−typhoidal Salmonella	787								787										
Serratia spp.	21,400	15,700		2,960		2,870	4,950	6,090	4,780										
Shigella spp.	2,980								2,980										
Staphylococcus aureus	360,000								103,000		227,000			166,000			172,000	5,240	
Streptococcus pneumoniae	499,000				108,000	175,000	92,500		37,500		200,000				227,000		416,000		
All pathogens	2,660,000	244,000	353,000	246,000	785,000	469,000	873,000	220,000	840,000	92,200	533,000	34,000	118,000	166,000	231,000	24,900	1,390,000	17,300	1,560

Counts
- **<25k**
- **25k − <50k**
- **50k − <100k**
- **100k − <200k**
- **200k − <500k**
- **500k − <1M**
- **>=1M**

Southern sub−Saharan Africa

DALYs (count) *attributable to* bacterial antimicrobial resistance by pathogen−drug combinations, 2019

	Resistance to 1+	AG	Aminopenicillin	Anti−pseudomonal	BL−BLI	CP	3GC	4GC	FQ	Mono INH	Macrolide	MDR in S. Typhi and Paratyphi	MDR excluding XDR in TB	Methicillin	PCN	Mono RIF	TMP−SMX	Vanco	XDR in TB
Acinetobacter baumannii	47,900	3,100		9,190	5.73	16,900	1,920	48.2	16,800										
Citrobacter spp.	2,430	45.2		559		682	382	310	453										
Enterobacter spp.	14,000	606		1,210		3,890		1,110	1,720								5,340		
Enterococcus faecalis	9,260								8,340									923	
Enterococcus faecium	13,700								12,100									1,520	
Other enterococci	6,250								5,700									549	
Escherichia coli	75,000	1,800	9,160		9,470	3,820	18,400		12,300								20,100		
Group A Streptococcus	418										410								
Group B Streptococcus	13,900								2,610		10,700				694				
Haemophilus influenzae	6,070		4,300				1,780												
Klebsiella pneumoniae	130,000	8,090			4,070	14,000	65,300		14,300								24,100		
Morganella spp.	61.2						3.24	10.4	47.6										
Mycobacterium tuberculosis	87,000									13,200			66,000			6,570			930
Neisseria gonorrhoeae	123						0.739		122										
Proteus spp.	2,870	73.5	488				1,280		190								831		
Pseudomonas aeruginosa	37,100	578		6,760		19,300	1,790	1,070	7,540										
Salmonella Paratyphi	6.83								6.76			0.069							
Salmonella Typhi	7,300								2,900			4,280							
Non−typhoidal Salmonella	150								150										
Serratia spp.	3,660	1,060		492		757	221	658	521										
Shigella spp.	610								610										
Staphylococcus aureus	74,500								4,350		8,970			41,000			18,700	1,480	
Streptococcus pneumoniae	105,000				2,410	38,500	2,320		4,290		7,310				12,800		37,600		
All pathogens	637,000	15,300	13,900	18,200	16,000	97,900	93,400	3,210	95,100	13,400	27,300	4,480	66,000	41,000	13,500	6,570	107,000	4,480	930

Counts
<25k
25k − <50k
50k − <100k
100k − <200k
500k − <1M

Western sub−Saharan Africa

DALYs (count) *associated with* bacterial antimicrobial resistance by pathogen−drug combinations, 2019

	Resistance to 1+	AG	Aminopenicillin	Anti−pseudomonal	BL−BLI	CP	3GC	4GC	FQ	Mono INH	Macrolide	MDR in S. Typhi and Paratyphi	MDR excluding XDR in TB	Methicillin	PCN	Mono RIF	TMP−SMX	Vanco	XDR in TB
Acinetobacter baumannii	959,000	473,000		946,000	455,000	387,000	618,000	540,000	460,000										
Citrobacter spp.	213,000	77,500		90,800		48,100	192,000	175,000	84,200										
Enterobacter spp.	1,150,000	629,000		464,000		196,000		959,000	713,000								938,000		
Enterococcus faecalis	446,000								440,000									27,000	
Enterococcus faecium	392,000								390,000									96,400	
Other enterococci	259,000								259,000									12,000	
Escherichia coli	4,920,000	2,100,000	4,700,000		3,880,000	568,000	3,100,000		3,100,000								4,560,000		
Group A Streptococcus	94,600										94,600								
Group B Streptococcus	2,420,000								1,590,000		1,540,000				40,700				
Haemophilus influenzae	481,000		357,000				223,000												
Klebsiella pneumoniae	6,410,000	4,630,000			6,000,000	866,000	5,460,000		4,870,000								6,100,000		
Morganella spp.	2,250						551	1,310	1,700										
Mycobacterium tuberculosis	585,000									192,000			335,000			53,100			4,460
Neisseria gonorrhoeae	4,530						8.27		4,520										
Proteus spp.	183,000	49,900	160,000				49,300		83,800								143,000		
Pseudomonas aeruginosa	1,910,000	1,080,000		951,000		832,000	1,200,000	930,000	1,070,000										
Salmonella Paratyphi	6,510								5,490			1,210							
Salmonella Typhi	1,630,000								483,000			1,470,000							
Non−typhoidal Salmonella	35,900								35,900										
Serratia spp.	517,000	267,000		74,200		27,500	377,000	443,000	124,000										
Shigella spp.	680,000								680,000										
Staphylococcus aureus	4,080,000								699,000		2,320,000			2,270,000			2,870,000	81,100	
Streptococcus pneumoniae	7,950,000				1,460,000	1,710,000	1,180,000		1,210,000		2,030,000				2,720,000		7,370,000		
All pathogens	35,300,000	9,310,000	5,220,000	2,530,000	11,800,000	4,630,000	12,400,000	3,050,000	16,300,000	192,000	5,990,000	1,470,000	335,000	2,270,000	2,760,000	53,100	22,000,000	217,000	4,460

Counts
- <25k
- 25k − <50k
- 50k − <100k
- 100k − <200k
- 200k − <500k
- 500k − <1M
- >=1M

Western sub−Saharan Africa

DALYs (count) *attributable to* bacterial antimicrobial resistance by pathogen−drug combinations, 2019

	Resistance to 1+	AG	Aminopenicillin	Anti−pseudomonal	BL−BLI	CP	3GC	4GC	FQ	Mono INH	Macrolide	MDR in S. Typhi and Paratyphi	MDR excluding XDR in TB	Methicillin	PCN	Mono RIF	TMP−SMX	Vanco	XDR in TB
Acinetobacter baumannii	279,000	23,900		126,000	10.2	68,200	1,450	23.7	59,300										
Citrobacter spp.	63,800	4,190		14,400		10,600	6,830	17,500	10,400										
Enterobacter spp.	275,000	33,900		35,300		37,600		60,800	66,000								41,300		
Enterococcus faecalis	117,000								110,000									7,380	
Enterococcus faecium	98,100								75,500									22,600	
Other enterococci	52,900								50,500									2,420	
Escherichia coli	1,280,000	119,000	40,200		107,000	107,000	363,000		286,000								256,000		
Group A Streptococcus	8,950										8,680								
Group B Streptococcus	425,000								281,000		138,000				9,260				
Haemophilus influenzae	114,000		49,100				64,400												
Klebsiella pneumoniae	1,770,000	326,000			67,100	185,000	627,000		298,000								269,000		
Morganella spp.	522						25.7	218	278										
Mycobacterium tuberculosis	236,000									28,200			190,000			14,300			2,660
Neisseria gonorrhoeae	441						3.91		437										
Proteus spp.	25,900	2,230	2,900				9,890		5,630								5,320		
Pseudomonas aeruginosa	462,000	29,300		93,500		137,000	59,500	26,400	115,000										
Salmonella Paratyphi	1,280								1,130			142							
Salmonella Typhi	253,000								76,500			179,000							
Non−typhoidal Salmonella	7,390								7,390										
Serratia spp.	131,000	14,900		15,200		7,970	11,400	69,300	12,100										
Shigella spp.	141,000								141,000										
Staphylococcus aureus	951,000								29,100		80,200			529,000			291,000	21,000	
Streptococcus pneumoniae	1,550,000				40,000	370,000	61,100		145,000		72,900				178,000		689,000		
All pathogens	8,250,000	555,000	92,200	284,000	214,000	924,000	1,210,000	174,000	1,770,000	29,200	296,000	174,000	190,000	529,000	187,000	14,300	1,550,000	53,400	2,660

Counts
- **<25k**
- **25k − <50k**
- **50k − <100k**
- **100k − <200k**
- **200k − <500k**
- **500k − <1M**
- **>=1M**

Algeria

DALYs (count) *associated with* bacterial antimicrobial resistance by pathogen−drug combinations, 2019

	Resistance to 1+	AG	Aminopenicillin	Anti−pseudomonal	BL−BLI	CP	3GC	4GC	FQ	Mono INH	Macrolide	MDR in S. Typhi and Paratyphi	MDR excluding XDR in TB	Methicillin	PCN	Mono RIF	TMP−SMX	Vanco	XDR in TB
Acinetobacter baumannii	31,400	27,200		30,900	30,200	27,600	30,300	29,900	28,300										
Citrobacter spp.	2,180	244		728		414	1,480	701	1,100										
Enterobacter spp.	11,700	1,830		6,960		2,710		5,920	2,820								5,020		
Enterococcus faecalis	3,140								2,720									599	
Enterococcus faecium	20,000								19,800									11,200	
Other enterococci	7,970								7,930									3,430	
Escherichia coli	70,700	12,200	68,000		43,200	9,250	23,700		34,200								51,000		
Group A Streptococcus	770										770								
Group B Streptococcus	18,700								2,500		18,100				19.5				
Haemophilus influenzae	2,610		2,410				452												
Klebsiella pneumoniae	74,800	22,200			68,900	9,970	66,200		31,900								51,800		
Morganella spp.	114						41	22.9	98.8										
Mycobacterium tuberculosis	848									141			657			4.67			45.3
Neisseria gonorrhoeae	145						5.59		143										
Proteus spp.	6,200	3,500	5,620				2,720		2,900								4,270		
Pseudomonas aeruginosa	40,400	14,700		16,500		18,900	30,100	20,200	21,000										
Salmonella Paratyphi	98.6								98.3			1.37							
Salmonella Typhi	669								602			54.9							
Non−typhoidal Salmonella	395								395										
Serratia spp.	3,310	798		742		705	2,360	1,650	483										
Shigella spp.	575								575										
Staphylococcus aureus	89,300								18,700		75,500			56,700			17,400	869	
Streptococcus pneumoniae	71,000				14,500	37,700	7,800		4,340		48,500				43,700		51,100		
All pathogens	457,000	82,700	76,100	55,900	157,000	107,000	165,000	58,400	181,000	141	143,000	56.2	657	56,700	43,700	4.67	181,000	16,100	45.3

Counts
- <2.5k
- 2.5k − <5k
- 5k − <10k
- 10k − <25k
- 25k − <75k
- 75k − <150k
- >=150k

Algeria

DALYs (count) *attributable to* bacterial antimicrobial resistance by pathogen−drug combinations, 2019

	Resistance to 1+	AG	Aminopenicillin	Anti−pseudomonal	BL−BLI	CP	3GC	4GC	FQ	Mono INH	Macrolide	MDR in S. Typhi and Paratyphi	MDR excluding XDR in TB	Methicillin	PCN	Mono RIF	TMP−SMX	Vanco	XDR in TB
Acinetobacter baumannii	10,000	1,160		829	16.7	4,500	66.3	12	3,470										
Citrobacter spp.	627	12.6		134		98.9	141	56.2	184										
Enterobacter spp.	2,690	119		918		713		325	303								309		
Enterococcus faecalis	894								675									219	
Enterococcus faecium	5,850								3,280									2,570	
Other enterococci	1,880								1,300									578	
Escherichia coli	16,800	742	2,050		2,480	2,110	2,850		3,450								3,080		
Group A Streptococcus	74.5										71.2								
Group B Streptococcus	2,200								389		1,820				18.4				
Haemophilus influenzae	532		399				132												
Klebsiella pneumoniae	21,000	1,820			1,080	2,490	10,600		2,330								2,640		
Morganella spp.	26.7						5.43	4.05	17.2										
Mycobacterium tuberculosis	380									19.4			335			1.16			25.3
Neisseria gonorrhoeae	15						1.24		13.8										
Proteus spp.	1,060	165	79.1				508		173								139		
Pseudomonas aeruginosa	10,400	406		1,430		3,320	2,190	792	2,280										
Salmonella Paratyphi	19.9								19.8			0.123							
Salmonella Typhi	134								125			9.45							
Non−typhoidal Salmonella	55.4								55.4										
Serratia spp.	871	48.8		162		196	221	191	52.7										
Shigella spp.	89.5								89.5										
Staphylococcus aureus	20,600								874		3,110			14,800			1,600	255	
Streptococcus pneumoniae	16,600				57.6	8,190	22.1		429		1,810				2,140		3,970		
All pathogens	113,000	4,470	2,520	3,480	3,640	21,600	16,800	1,380	19,500	20.2	6,840	9.25	335	14,800	2,150	1.16	11,700	3,630	25.3

Counts
- <2.5k
- 2.5k − <5k
- 5k − <10k
- 10k − <25k
- 75k − <150k

Angola

DALYs (count) *associated with* bacterial antimicrobial resistance by pathogen−drug combinations, 2019

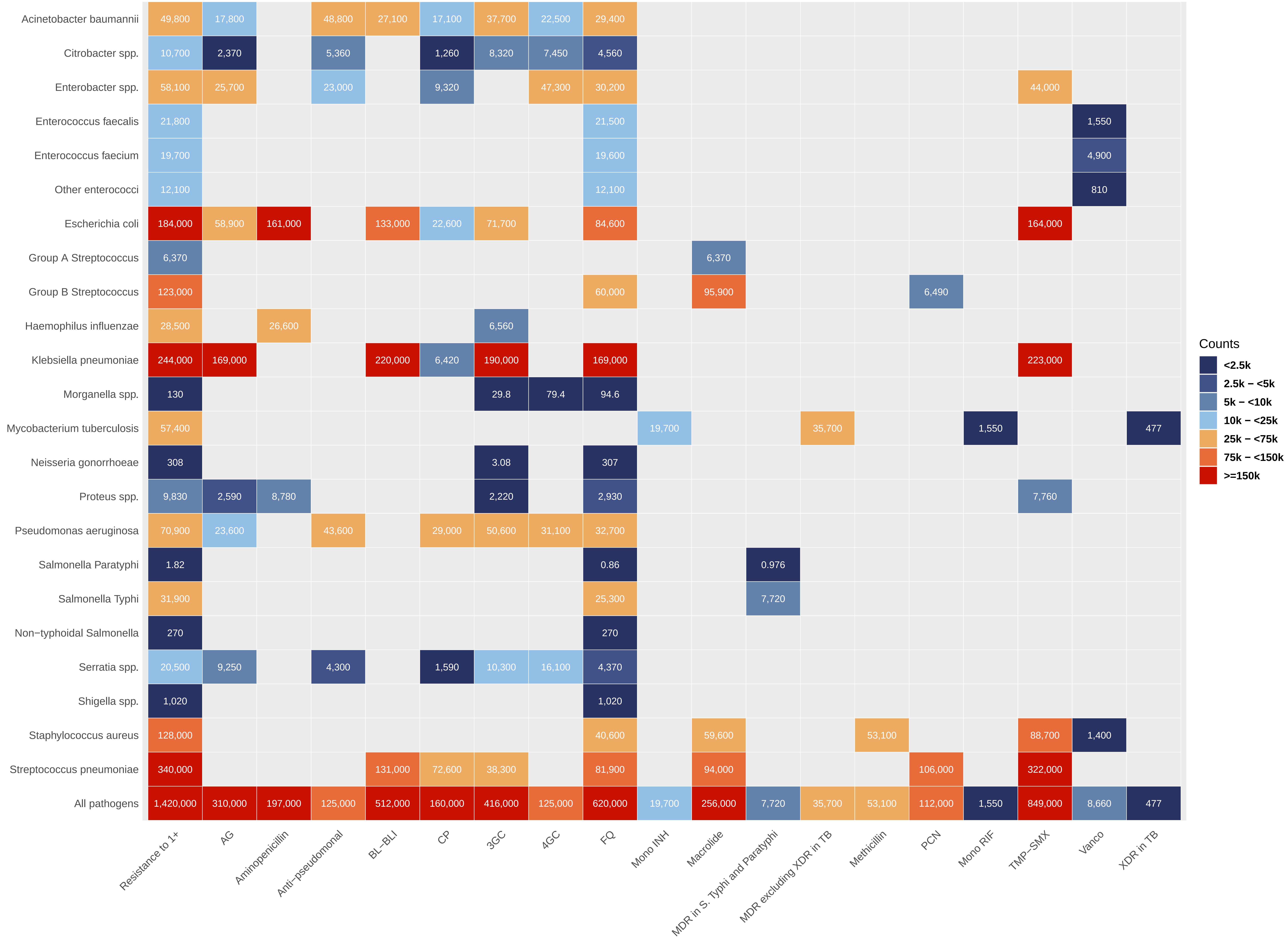

Angola

DALYs (count) *attributable to* bacterial antimicrobial resistance by pathogen−drug combinations, 2019

	Resistance to 1+	AG	Aminopenicillin	Anti−pseudomonal	BL−BLI	CP	3GC	4GC	FQ	Mono INH	Macrolide	MDR in S. Typhi and Paratyphi	MDR excluding XDR in TB	Methicillin	PCN	Mono RIF	TMP−SMX	Vanco	XDR in TB
Acinetobacter baumannii	14,700	846		6,820	0.845	2,870	194	1	3,940										
Citrobacter spp.	3,090	121		1,000		291	371	686	621										
Enterobacter spp.	13,700	1,410		2,000		1,930		3,360	2,850								2,090		
Enterococcus faecalis	5,770								5,340									425	
Enterococcus faecium	4,950								3,800									1,150	
Other enterococci	2,500								2,340									158	
Escherichia coli	43,400	3,540	2,000		6,060	4,890	8,170		7,950								10,800		
Group A Streptococcus	568										610								
Group B Streptococcus	20,100								10,200		8,840				1,140				
Haemophilus influenzae	5,980		4,130				1,840												
Klebsiella pneumoniae	62,600	11,900			3,810	2,140	24,400		10,200								10,200		
Morganella spp.	30.1						1.22	13.5	15.4										
Mycobacterium tuberculosis	24,000									3,000			20,300			419			285
Neisseria gonorrhoeae	30.8						0.874		29.9										
Proteus spp.	1,280	122	179				474		203								300		
Pseudomonas aeruginosa	18,400	643		5,450		5,230	2,940	530	3,560										
Salmonella Paratyphi	0.311								0.18			0.137							
Salmonella Typhi	6,130								5,160			956							
Non−typhoidal Salmonella	55.1								55.1										
Serratia spp.	4,750	527		845		471	343	2,130	434										
Shigella spp.	213								213										
Staphylococcus aureus	26,200								1,650		2,180			12,100			9,780	436	
Streptococcus pneumoniae	67,400				5,300	15,000	1,720		9,510		3,240				4,340		28,300		
All pathogens	326,000	19,100	6,300	16,100	15,200	32,900	40,500	6,720	68,100	2,950	14,800	981	20,300	12,100	5,480	419	61,500	2,170	285

Counts
- <2.5k
- 2.5k − <5k
- 5k − <10k
- 10k − <25k
- 25k − <75k
- >=150k

Benin

DALYs (count) *associated with* bacterial antimicrobial resistance by pathogen−drug combinations, 2019

	Resistance to 1+	AG	Aminopenicillin	Anti−pseudomonal	BL−BLI	CP	3GC	4GC	FQ	Mono INH	Macrolide	MDR in S. Typhi and Paratyphi	MDR excluding XDR in TB	Methicillin	PCN	Mono RIF	TMP−SMX	Vanco	XDR in TB
Acinetobacter baumannii	28,300	12,100		28,100	10,600	9,090	17,000	16,800	11,600										
Citrobacter spp.	4,630	1,590		2,060		732	4,170	3,940	1,970										
Enterobacter spp.	26,200	12,700		10,100		4,430		22,400	16,900								21,600		
Enterococcus faecalis	12,800								12,700									836	
Enterococcus faecium	10,700								10,600									2,430	
Other enterococci	6,660								6,640									308	
Escherichia coli	124,000	48,300	117,000		100,000	14,200	52,600		58,900								116,000		
Group A Streptococcus	2,440										2,440								
Group B Streptococcus	54,900								38,100		33,200				1,040				
Haemophilus influenzae	11,900		8,760				5,580												
Klebsiella pneumoniae	181,000	130,000			170,000	7,250	148,000		136,000								174,000		
Morganella spp.	64.6						16	39.3	47.5										
Mycobacterium tuberculosis	14,300									9,090			5,110			46			68.4
Neisseria gonorrhoeae	148						0.081		148										
Proteus spp.	5,070	1,340	4,460				1,340		1,800								3,980		
Pseudomonas aeruginosa	50,000	28,500		26,800		19,100	27,700	21,900	27,100										
Salmonella Paratyphi	11.4								9.64			1.89							
Salmonella Typhi	59,800								15,500			54,200							
Non−typhoidal Salmonella	44.8								44.8										
Serratia spp.	13,900	7,610		1,930		630	11,000	11,600	3,720										
Shigella spp.	1,690								1,690										
Staphylococcus aureus	121,000								23,500		69,400			51,000			84,200	3,880	
Streptococcus pneumoniae	190,000				37,300	47,100	22,200		17,700		46,500				15,500		185,000		
All pathogens	920,000	242,000	130,000	68,900	318,000	102,000	289,000	76,800	385,000	9,090	152,000	54,200	5,110	51,000	16,500	46	584,000	7,460	68.4

Counts

- **<2.5k**
- **2.5k − <5k**
- **5k − <10k**
- **10k − <25k**
- **25k − <75k**
- **75k − <150k**
- **>=150k**

Benin

DALYs (count) *attributable to* bacterial antimicrobial resistance by pathogen−drug combinations, 2019

	Resistance to 1+	AG	Aminopenicillin	Anti−pseudomonal	BL−BLI	CP	3GC	4GC	FQ	Mono INH	Macrolide	MDR in S. Typhi and Paratyphi	MDR excluding XDR in TB	Methicillin	PCN	Mono RIF	TMP−SMX	Vanco	XDR in TB
Acinetobacter baumannii	8,060	633		4,280	0.156	1,630	3.1	0.573	1,520										
Citrobacter spp.	1,380	84.4		353		159	122	415	247										
Enterobacter spp.	6,320	655		757		853		1,510	1,590								990		
Enterococcus faecalis	3,380								3,150									224	
Enterococcus faecium	2,640								2,070									574	
Other enterococci	1,360								1,290									62.8	
Escherichia coli	29,700	2,890	1,210		4,130	2,900	5,930		5,450								7,200		
Group A Streptococcus	240										224								
Group B Streptococcus	9,850								6,770		2,870				220				
Haemophilus influenzae	2,820		1,210				1,610												
Klebsiella pneumoniae	47,700	9,040			2,390	2,010	18,400		8,220								7,650		
Morganella spp.	15.1						0.702	6.64	7.74										
Mycobacterium tuberculosis	4,300									1,250			2,900			12.4			40.7
Neisseria gonorrhoeae	14.3						0.06		14.3										
Proteus spp.	697	61.9	78				277		123								151		
Pseudomonas aeruginosa	11,700	801		3,070		3,150	1,170	511	2,970										
Salmonella Paratyphi	2.26								2.01			0.244							
Salmonella Typhi	8,800								2,440			6,770							
Non−typhoidal Salmonella	9.1								9.1										
Serratia spp.	3,610	427		404		186	397	1,830	360										
Shigella spp.	354								354										
Staphylococcus aureus	25,100								918		2,560			11,600			9,080	985	
Streptococcus pneumoniae	34,400				1,060	10,200	599		2,100		1,680				158		18,500		
All pathogens	203,000	14,600	2,510	8,860	7,590	21,100	28,500	4,270	39,600	1,270	7,370	6,550	2,900	11,600	378	12.4	43,500	1,850	40.7

Counts
- <2.5k
- 2.5k – <5k
- 5k – <10k
- 10k – <25k
- 25k – <75k
- >=150k

Botswana

DALYs (count) *associated with* bacterial antimicrobial resistance by pathogen−drug combinations, 2019

	Resistance to 1+	AG	Aminopenicillin	Anti−pseudomonal	BL−BLI	CP	3GC	4GC	FQ	Mono INH	Macrolide	MDR in S. Typhi and Paratyphi	MDR excluding XDR in TB	Methicillin	PCN	Mono RIF	TMP−SMX	Vanco	XDR in TB
Acinetobacter baumannii	4,140	2,590		4,000	3,000	3,180	3,920	3,310	3,610										
Citrobacter spp.	320	20.2		70.6		107	216	148	114										
Enterobacter spp.	2,390	225		644		442		711	466								2,130		
Enterococcus faecalis	1,030								987									99.5	
Enterococcus faecium	2,190								2,180									490	
Other enterococci	895								893									42	
Escherichia coli	10,800	1,170	10,600		6,070	352	3,370		2,250								9,310		
Group A Streptococcus	120										120								
Group B Streptococcus	2,690								372		2,550				183				
Haemophilus influenzae	646		466				291												
Klebsiella pneumoniae	13,500	3,430			12,000	58.7	10,600		5,600								11,800		
Morganella spp.	6.87						0.986	1.12	6.42										
Mycobacterium tuberculosis	6,540									2,010			3,670			804			48.6
Neisseria gonorrhoeae	25.9						0.061		25.8										
Proteus spp.	765	53.1	642				163		159								630		
Pseudomonas aeruginosa	5,590	1,360		2,850		3,780	2,470	2,090	3,400										
Salmonella Paratyphi	0.922								0.916			0.009							
Salmonella Typhi	834								309			592							
Non−typhoidal Salmonella	5								5										
Serratia spp.	572	365		154		89.3	177	200	119										
Shigella spp.	155								155										
Staphylococcus aureus	9,590								5,060		3,130			1,140			5,720	174	
Streptococcus pneumoniae	15,400				3,570	4,990	2,430		1,090		4,720				7,440		13,200		
All pathogens	78,200	9,210	11,700	7,720	24,700	13,000	23,600	6,460	26,800	2,010	10,500	592	3,670	1,140	7,630	804	42,800	805	48.6

Counts
- **<2.5k**
- **2.5k − <5k**
- **5k − <10k**
- **10k − <25k**
- **25k − <75k**
- **75k − <150k**

Botswana

DALYs (count) *attributable to* bacterial antimicrobial resistance by pathogen−drug combinations, 2019

	Resistance to 1+	AG	Aminopenicillin	Anti−pseudomonal	BL−BLI	CP	3GC	4GC	FQ	Mono INH	Macrolide	MDR in S. Typhi and Paratyphi	MDR excluding XDR in TB	Methicillin	PCN	Mono RIF	TMP−SMX	Vanco	XDR in TB
Acinetobacter baumannii	1,310	109		186	0.257	516	37.7	0.379	460										
Citrobacter spp.	94	1.1		12		27.6	21.8	13.9	17.6										
Enterobacter spp.	433	18.2		62.4		107		32.7	56.3								157		
Enterococcus faecalis	278								246									31.8	
Enterococcus faecium	540								426									115	
Other enterococci	182								173									8.47	
Escherichia coli	2,310	78.7	422		358	85.7	505		226								638		
Group A Streptococcus	11.2										12.1								
Group B Streptococcus	363								58.8		259				32.5				
Haemophilus influenzae	152		67				84.6												
Klebsiella pneumoniae	3,400	276			257	37.9	1,780		393								656		
Morganella spp.	1.49						0.108	0.183	1.2										
Mycobacterium tuberculosis	2,580									302			2,030			215			28.7
Neisseria gonorrhoeae	2.53						0.034		2.49										
Proteus spp.	93.5	3.29	13.5				36.8		12.6								27.3		
Pseudomonas aeruginosa	1,350	33.3		186		665	63.4	26.6	374										
Salmonella Paratyphi	0.188								0.187			0.001							
Salmonella Typhi	135								59			78.2							
Non−typhoidal Salmonella	0.908								0.908										
Serratia spp.	110	22.6		27.4		23.6	6.14	17.3	12.6										
Shigella spp.	31.5								31.5										
Staphylococcus aureus	1,440								246		113			353			674	54	
Streptococcus pneumoniae	3,220				89.1	1,080	56.9		125		169				476		1,220		
All pathogens	18,000	542	503	474	704	2,550	2,590	90.9	2,920	303	554	77.8	2,030	353	509	215	3,370	209	28.7

Counts
- **<2.5k**
- **2.5k − <5k**
- **10k − <25k**

Burkina Faso

DALYs (count) *associated with* bacterial antimicrobial resistance by pathogen−drug combinations, 2019

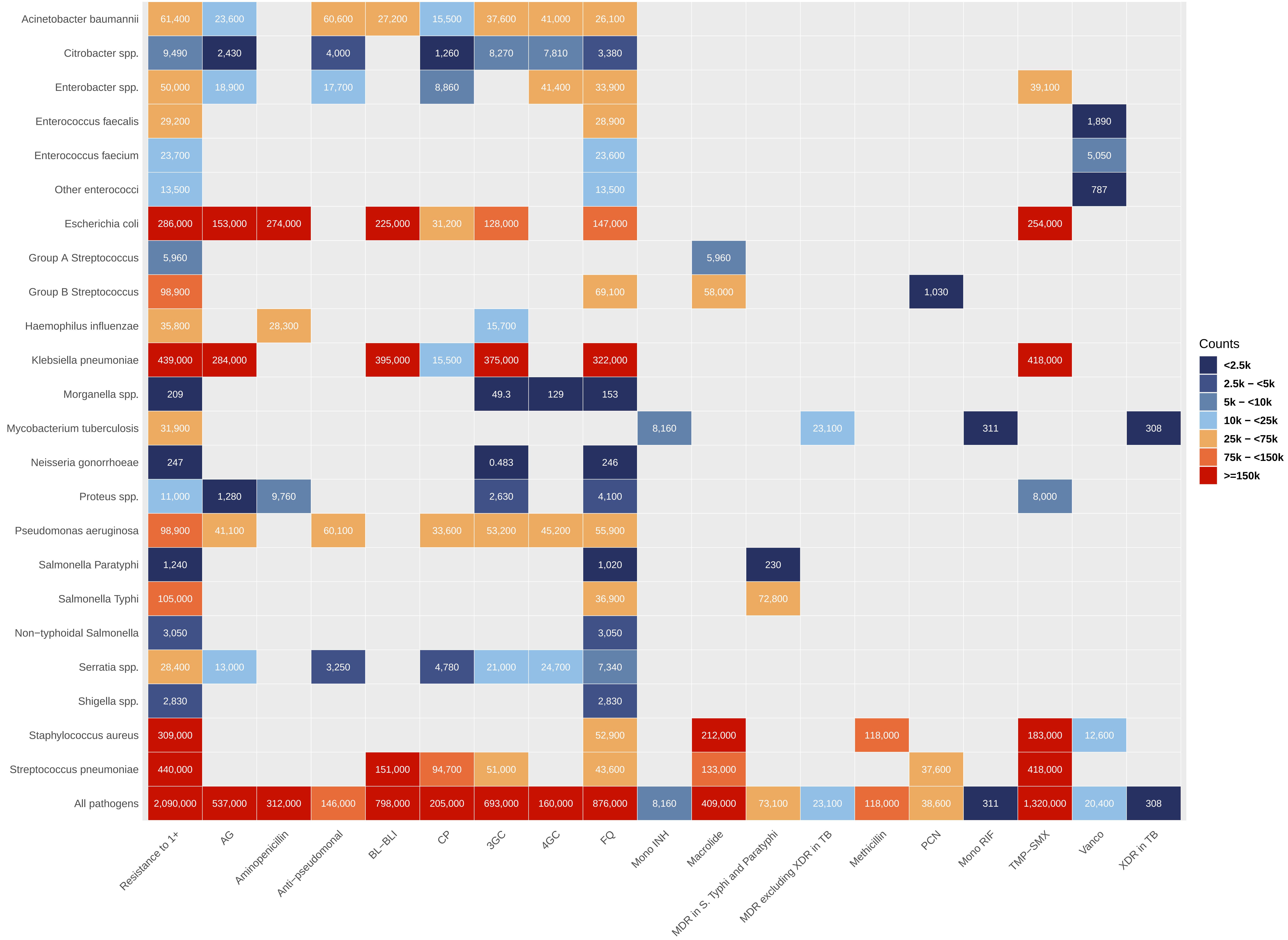

	Resistance to 1+	AG	Aminopenicillin	Anti−pseudomonal	BL−BLI	CP	3GC	4GC	FQ	Mono INH	Macrolide	MDR in S. Typhi and Paratyphi	MDR excluding XDR in TB	Methicillin	PCN	Mono RIF	TMP−SMX	Vanco	XDR in TB
Acinetobacter baumannii	61,400	23,600		60,600	27,200	15,500	37,600	41,000	26,100										
Citrobacter spp.	9,490	2,430		4,000		1,260	8,270	7,810	3,380										
Enterobacter spp.	50,000	18,900		17,700		8,860		41,400	33,900								39,100		
Enterococcus faecalis	29,200								28,900									1,890	
Enterococcus faecium	23,700								23,600									5,050	
Other enterococci	13,500								13,500									787	
Escherichia coli	286,000	153,000	274,000		225,000	31,200	128,000		147,000								254,000		
Group A Streptococcus	5,960										5,960								
Group B Streptococcus	98,900								69,100		58,000				1,030				
Haemophilus influenzae	35,800		28,300				15,700												
Klebsiella pneumoniae	439,000	284,000			395,000	15,500	375,000		322,000								418,000		
Morganella spp.	209						49.3	129	153										
Mycobacterium tuberculosis	31,900									8,160			23,100			311			308
Neisseria gonorrhoeae	247						0.483		246										
Proteus spp.	11,000	1,280	9,760				2,630		4,100								8,000		
Pseudomonas aeruginosa	98,900	41,100		60,100		33,600	53,200	45,200	55,900										
Salmonella Paratyphi	1,240								1,020			230							
Salmonella Typhi	105,000								36,900			72,800							
Non−typhoidal Salmonella	3,050								3,050										
Serratia spp.	28,400	13,000		3,250		4,780	21,000	24,700	7,340										
Shigella spp.	2,830								2,830										
Staphylococcus aureus	309,000								52,900		212,000			118,000			183,000	12,600	
Streptococcus pneumoniae	440,000				151,000	94,700	51,000		43,600		133,000				37,600		418,000		
All pathogens	2,090,000	537,000	312,000	146,000	798,000	205,000	693,000	160,000	876,000	8,160	409,000	73,100	23,100	118,000	38,600	311	1,320,000	20,400	308

Burkina Faso

DALYs (count) *attributable to* bacterial antimicrobial resistance by pathogen−drug combinations, 2019

	Resistance to 1+	AG	Aminopenicillin	Anti−pseudomonal	BL−BLI	CP	3GC	4GC	FQ	Mono INH	Macrolide	MDR in S. Typhi and Paratyphi	MDR excluding XDR in TB	Methicillin	PCN	Mono RIF	TMP−SMX	Vanco	XDR in TB
Acinetobacter baumannii	17,600	1,270		9,760	0.617	2,920	73.2	3.51	3,570										
Citrobacter spp.	2,790	134		741		292	303	896	428										
Enterobacter spp.	12,100	943		1,310		1,720		2,900	3,440								1,850		
Enterococcus faecalis	7,690								7,190									499	
Enterococcus faecium	5,840								4,630									1,210	
Other enterococci	2,790								2,640									157	
Escherichia coli	69,600	9,320	3,290		8,980	6,140	14,200		13,200								14,500		
Group A Streptococcus	582										563								
Group B Streptococcus	17,700								12,400		4,950				273				
Haemophilus influenzae	8,320		3,850				4,470												
Klebsiella pneumoniae	117,000	19,800			4,070	4,510	49,100		19,900								19,300		
Morganella spp.	48.8						1.96	21.8	25										
Mycobacterium tuberculosis	14,700									1,240			13,200			85.1			185
Neisseria gonorrhoeae	24						0.229		23.7										
Proteus spp.	1,440	67	199				575		286								317		
Pseudomonas aeruginosa	23,900	1,150		7,990		5,570	1,840	994	6,350										
Salmonella Paratyphi	244								213			31							
Salmonella Typhi	16,700								7,270			9,190							
Non−typhoidal Salmonella	633								633										
Serratia spp.	7,440	686		600		1,200	630	3,600	703										
Shigella spp.	584								584										
Staphylococcus aureus	60,800								2,230		8,300			27,400			19,400	3,380	
Streptococcus pneumoniae	80,100				5,820	20,600	2,340		5,260		4,780				556		40,400		
All pathogens	468,000	33,400	7,340	20,400	19,100	43,000	73,600	8,420	90,900	1,230	18,800	9,280	13,200	27,400	829	85.1	95,700	5,240	185

Counts
- <2.5k
- 2.5k − <5k
- 5k − <10k
- 10k − <25k
- 25k − <75k
- 75k − <150k
- >=150k

Burundi

DALYs (count) *associated with* bacterial antimicrobial resistance by pathogen−drug combinations, 2019

	Resistance to 1+	AG	Aminopenicillin	Anti−pseudomonal	BL−BLI	CP	3GC	4GC	FQ	Mono INH	Macrolide	MDR in S. Typhi and Paratyphi	MDR excluding XDR in TB	Methicillin	PCN	Mono RIF	TMP−SMX	Vanco	XDR in TB
Acinetobacter baumannii	24,900	6,640		22,400	16,700	8,130	22,400	15,600	9,070										
Citrobacter spp.	2,830	623		1,290		174	2,380	1,880	1,680										
Enterobacter spp.	15,600	7,850		6,220		2,020		14,300	8,760								11,200		
Enterococcus faecalis	11,000								10,900									642	
Enterococcus faecium	8,630								8,540									2,380	
Other enterococci	3,400								3,380									230	
Escherichia coli	104,000	23,500	98,100		73,200	7,970	37,100		39,900								86,100		
Group A Streptococcus	4,440										4,440								
Group B Streptococcus	32,700								12,200		28,700				1,100				
Haemophilus influenzae	15,400		15,300				2,040												
Klebsiella pneumoniae	122,000	95,700			114,000	1,480	86,900		59,800								109,000		
Morganella spp.	63.5						13.9	41.5	44.8										
Mycobacterium tuberculosis	30,600									3,400			25,800			1,090			346
Neisseria gonorrhoeae	57.4						1.19		56.8										
Proteus spp.	4,750	1,670	3,970				888		2,820								4,170		
Pseudomonas aeruginosa	29,000	8,870		14,400		7,500	23,800	11,300	10,100										
Salmonella Paratyphi	1.34								0.261			1.07							
Salmonella Typhi	47,700								19,700			34,400							
Non−typhoidal Salmonella	55.5								55.5										
Serratia spp.	9,830	4,450		3,170		439	6,840	8,280	2,350										
Shigella spp.	13,500								13,500										
Staphylococcus aureus	74,800								24,900		28,800			30,300			52,800	969	
Streptococcus pneumoniae	121,000				22,200	26,900	14,400		64,800		10,300				15,300		111,000		
All pathogens	676,000	149,000	117,000	47,600	226,000	54,700	197,000	51,400	293,000	3,400	72,200	34,400	25,800	30,300	16,400	1,090	375,000	4,220	346

Counts
- <2.5k
- 2.5k − <5k
- 5k − <10k
- 10k − <25k
- 25k − <75k
- 75k − <150k
- >=150k

Burundi

DALYs (count) *attributable to* bacterial antimicrobial resistance by pathogen−drug combinations, 2019

	Resistance to 1+	AG	Aminopenicillin	Anti−pseudomonal	BL−BLI	CP	3GC	4GC	FQ	Mono INH	Macrolide	MDR in S. Typhi and Paratyphi	MDR excluding XDR in TB	Methicillin	PCN	Mono RIF	TMP−SMX	Vanco	XDR in TB
Acinetobacter baumannii	7,470	360		3,540	40.3	1,590	693	3.11	1,240										
Citrobacter spp.	824	28.6		232		39.8	115	173	236										
Enterobacter spp.	3,770	416		551		411		1,120	787								476		
Enterococcus faecalis	2,890								2,720									171	
Enterococcus faecium	2,200								1,640									562	
Other enterococci	704								658									45.9	
Escherichia coli	23,600	1,450	2,060		4,080	1,790	4,800		3,840								5,600		
Group A Streptococcus	417										408								
Group B Streptococcus	4,870								1,960		2,760				208				
Haemophilus influenzae	3,030		2,470				563												
Klebsiella pneumoniae	30,500	7,120			2,790	643	11,400		3,600								4,940		
Morganella spp.	14.8						0.445	7.08	7.23										
Mycobacterium tuberculosis	15,800									502			14,800			295			207
Neisseria gonorrhoeae	5.83						0.325		5.51										
Proteus spp.	649	72	69.1				171		187								153		
Pseudomonas aeruginosa	7,620	265		2,210		1,380	2,260	365	1,130										
Salmonella Paratyphi	0.203								0.061			0.148							
Salmonella Typhi	7,840								3,640			4,040							
Non−typhoidal Salmonella	11.1								11.1										
Serratia spp.	2,480	254		637		138	187	1,030	232										
Shigella spp.	2,730								2,730										
Staphylococcus aureus	15,300								1,050		1,050			6,990			5,970	279	
Streptococcus pneumoniae	24,700				553	5,150	433		8,250		367				291		9,660		
All pathogens	157,000	9,970	4,600	7,160	7,460	11,100	20,700	2,700	33,900	491	4,540	4,170	14,800	6,990	499	295	26,800	1,060	207

Counts
- <2.5k
- 2.5k − <5k
- 5k − <10k
- 10k − <25k
- 25k − <75k
- >=150k

Cameroon

DALYs (count) *associated with* bacterial antimicrobial resistance by pathogen−drug combinations, 2019

	Resistance to 1+	AG	Aminopenicillin	Anti−pseudomonal	BL−BLI	CP	3GC	4GC	FQ	Mono INH	Macrolide	MDR in S. Typhi and Paratyphi	MDR excluding XDR in TB	Methicillin	PCN	Mono RIF	TMP−SMX	Vanco	XDR in TB
Acinetobacter baumannii	49,600	24,400		48,100	21,800	27,800	38,200	29,300	27,400										
Citrobacter spp.	7,550	2,330		3,090		857	6,660	5,080	3,140										
Enterobacter spp.	43,800	21,900		17,900		6,510		34,700	26,900								34,000		
Enterococcus faecalis	19,600								19,200									1,330	
Enterococcus faecium	20,500								20,400									5,290	
Other enterococci	12,700								12,700									711	
Escherichia coli	204,000	79,100	192,000		159,000	18,700	103,000		96,200								181,000		
Group A Streptococcus	5,180										5,180								
Group B Streptococcus	71,000								40,100		49,600				1,170				
Haemophilus influenzae	15,500		12,300				5,890												
Klebsiella pneumoniae	265,000	178,000			247,000	6,480	137,000		205,000								252,000		
Morganella spp.	148						37.6	83.6	112										
Mycobacterium tuberculosis	40,400									7,910			14,600			17,700			196
Neisseria gonorrhoeae	424						2.35		423										
Proteus spp.	9,430	1,760	8,480				2,150		3,370								6,980		
Pseudomonas aeruginosa	87,200	39,200		44,100		39,300	59,200	32,700	48,800										
Salmonella Paratyphi	85.6								83.8			2.07							
Salmonella Typhi	87,900								23,700			78,000							
Non−typhoidal Salmonella	237								237										
Serratia spp.	22,100	8,140		3,780		1,560	18,700	16,500	5,800										
Shigella spp.	13,600								13,600										
Staphylococcus aureus	187,000								39,800		89,800			98,600			128,000	1,520	
Streptococcus pneumoniae	288,000				60,800	59,400	35,100		29,900		63,300				55,800		278,000		
All pathogens	1,450,000	355,000	213,000	117,000	488,000	161,000	406,000	118,000	617,000	7,910	208,000	78,000	14,600	98,600	56,900	17,700	880,000	8,850	196

Counts
- <2.5k
- 2.5k − <5k
- 5k − <10k
- 10k − <25k
- 25k − <75k
- 75k − <150k
- >=150k

Cameroon

DALYs (count) *attributable to* bacterial antimicrobial resistance by pathogen−drug combinations, 2019

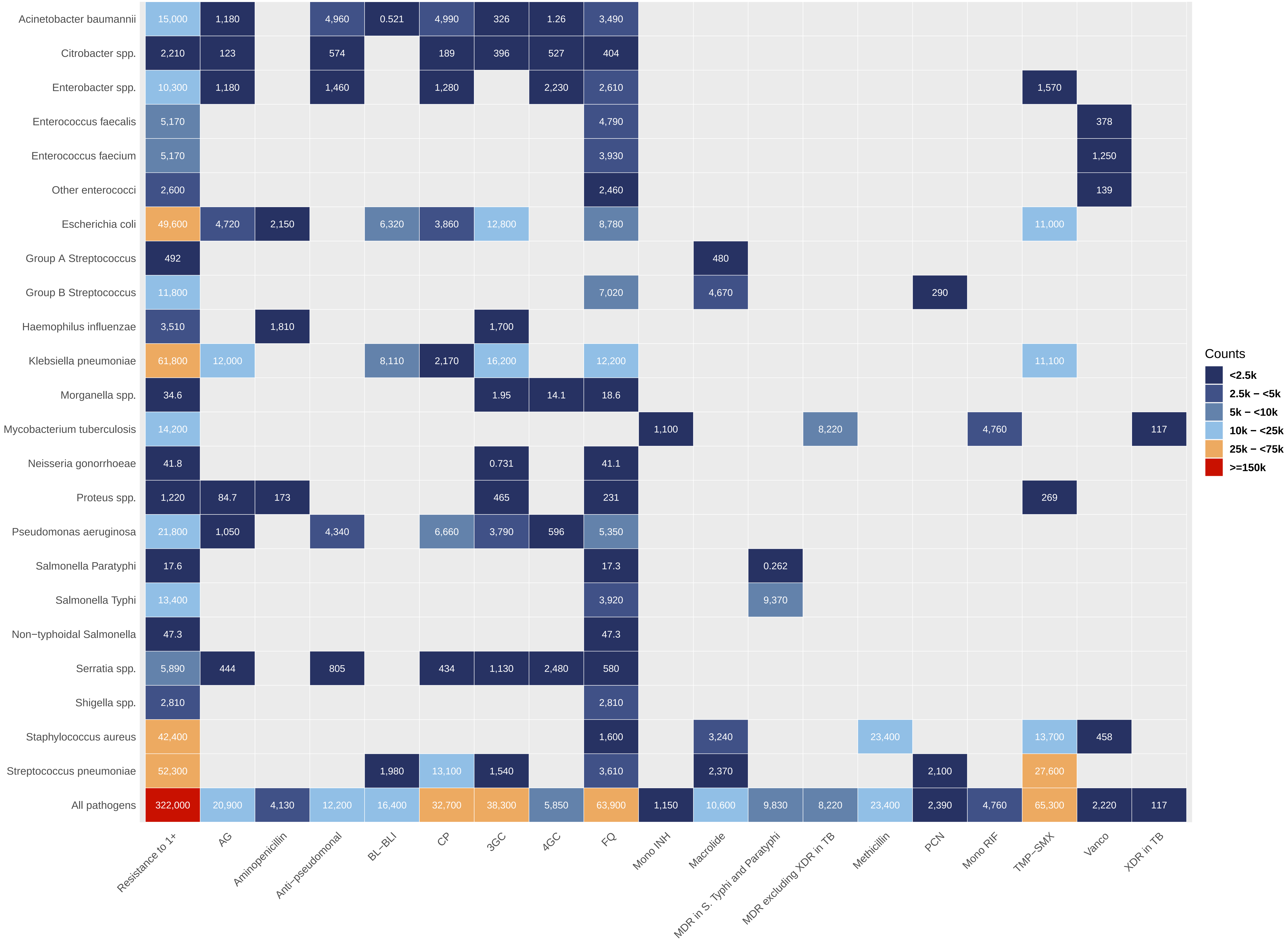

Cape Verde

DALYs (count) *associated with* bacterial antimicrobial resistance by pathogen−drug combinations, 2019

	Resistance to 1+	AG	Aminopenicillin	Anti−pseudomonal	BL−BLI	CP	3GC	4GC	FQ	Mono INH	Macrolide	MDR in S. Typhi and Paratyphi	MDR excluding XDR in TB	Methicillin	PCN	Mono RIF	TMP−SMX	Vanco	XDR in TB
Acinetobacter baumannii	1,180	887		1,160	759	914	1,030	991	1,020										
Citrobacter spp.	115	11.8		20.8		14.7	93.7	56.8	46.8										
Enterobacter spp.	715	201		289		94.2		404	273								588		
Enterococcus faecalis	145								134									23.7	
Enterococcus faecium	290								289									90.7	
Other enterococci	142								141									10.5	
Escherichia coli	1,140	325	1,080		897	42.6	568		517								1,010		
Group A Streptococcus	41.2										41.2								
Group B Streptococcus	225								55.3		203				4.12				
Haemophilus influenzae	44.9		32				19.9												
Klebsiella pneumoniae	1,230	681			1,140	36.5	986		732								1,110		
Morganella spp.	1.08						0.218	0.149	1.01										
Mycobacterium tuberculosis	176									34.1			139			1.21			1.82
Neisseria gonorrhoeae	5.98						0.012		5.97										
Proteus spp.	115	45.7	99.1				28.1		59.6								90.8		
Pseudomonas aeruginosa	665	323		291		367	360	237	449										
Salmonella Paratyphi	0.575								0.556			0.021							
Salmonella Typhi	353								68.9			303							
Non−typhoidal Salmonella	0.278								0.278										
Serratia spp.	137	33.3		28.2		14.4	116	67.1	42.4										
Shigella spp.	5.8								5.8										
Staphylococcus aureus	1,150								205		781			716			489	16.6	
Streptococcus pneumoniae	1,500				340	576	197		77.8		398				274		1,410		
All pathogens	9,370	2,510	1,210	1,790	3,130	2,060	3,400	1,760	4,120	34.1	1,420	303	139	716	278	1.21	4,690	142	1.82

Counts
- <2.5k
- 2.5k − <5k
- 5k − <10k

Cape Verde

DALYs (count) *attributable to* bacterial antimicrobial resistance by pathogen−drug combinations, 2019

	Resistance to 1+	AG	Aminopenicillin	Anti−pseudomonal	BL−BLI	CP	3GC	4GC	FQ	Mono INH	Macrolide	MDR in S. Typhi and Paratyphi	MDR excluding XDR in TB	Methicillin	PCN	Mono RIF	TMP−SMX	Vanco	XDR in TB
Acinetobacter baumannii	371	38.1		54.7	0.019	147	3.99	0.108	127										
Citrobacter spp.	33	0.589		4.39		3.33	10.6	7.36	6.74										
Enterobacter spp.	150	12.2		30.1		20.1		24.7	27.6								35.1		
Enterococcus faecalis	40.3								32.7									7.57	
Enterococcus faecium	75.2								54.2									21	
Other enterococci	29.2								27.2									2.01	
Escherichia coli	268	19.3	12.9		38.8	9.33	76.5		48.2								63.1		
Group A Streptococcus	3.92										3.9								
Group B Streptococcus	30.4								9.05		21.2				1.06				
Haemophilus influenzae	10.4		4.67				5.78												
Klebsiella pneumoniae	319	48.9			21.3	11.4	137		46.6								53		
Morganella spp.	0.237						0.026	0.025	0.186										
Mycobacterium tuberculosis	80.4									4.63			74.2			0.311			1.05
Neisseria gonorrhoeae	0.585						0.006		0.58										
Proteus spp.	16.2	1.99	1.71				5.49		3.82								3.19		
Pseudomonas aeruginosa	159	8.47		19.3		59.4	18.2	4.18	49.6										
Salmonella Paratyphi	0.118								0.115			0.003							
Salmonella Typhi	51.4								12.9			39.5							
Non−typhoidal Salmonella	0.048								0.048										
Serratia spp.	36.5	1.9		6.19		3.71	11.9	8.41	4.44										
Shigella spp.	1.06								1.06										
Staphylococcus aureus	274								9.29		30.4			182			48.3	4.42	
Streptococcus pneumoniae	299				6.7	127	1.64		8.85		14.5				4.15		136		
All pathogens	2,250	131	19.3	115	66.8	381	272	44.8	470	4.99	69.1	40.3	74.2	182	5.21	0.311	339	35	1.05

Counts

<2.5k

Central African Republic

DALYs (count) *associated with* bacterial antimicrobial resistance by pathogen−drug combinations, 2019

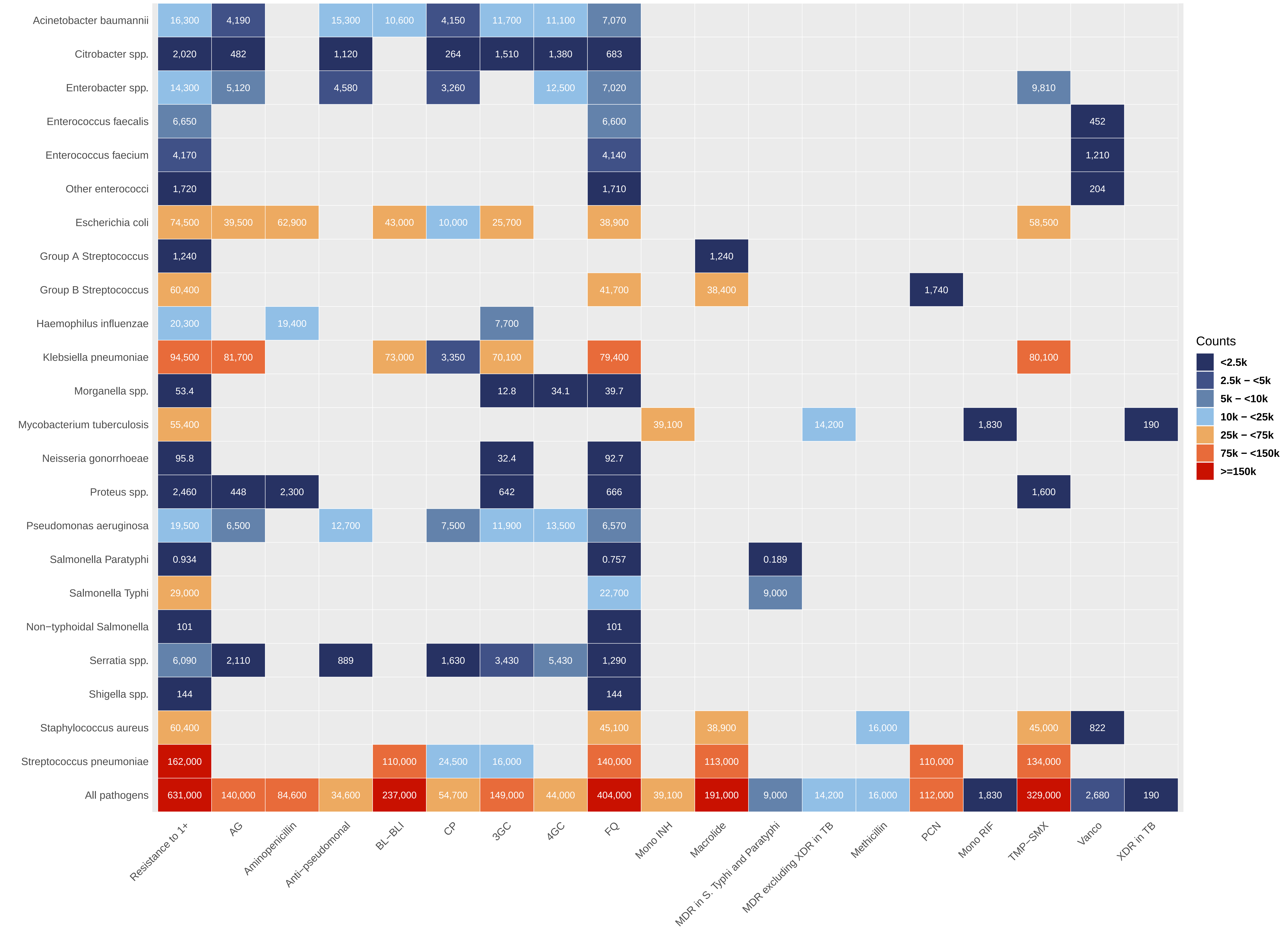

	Resistance to 1+	AG	Aminopenicillin	Anti−pseudomonal	BL−BLI	CP	3GC	4GC	FQ	Mono INH	Macrolide	MDR in S. Typhi and Paratyphi	MDR excluding XDR in TB	Methicillin	PCN	Mono RIF	TMP−SMX	Vanco	XDR in TB
Acinetobacter baumannii	16,300	4,190		15,300	10,600	4,150	11,700	11,100	7,070										
Citrobacter spp.	2,020	482		1,120		264	1,510	1,380	683										
Enterobacter spp.	14,300	5,120		4,580		3,260		12,500	7,020								9,810		
Enterococcus faecalis	6,650								6,600									452	
Enterococcus faecium	4,170								4,140									1,210	
Other enterococci	1,720								1,710									204	
Escherichia coli	74,500	39,500	62,900		43,000	10,000	25,700		38,900								58,500		
Group A Streptococcus	1,240										1,240								
Group B Streptococcus	60,400								41,700		38,400				1,740				
Haemophilus influenzae	20,300		19,400				7,700												
Klebsiella pneumoniae	94,500	81,700			73,000	3,350	70,100		79,400								80,100		
Morganella spp.	53.4						12.8	34.1	39.7										
Mycobacterium tuberculosis	55,400									39,100			14,200			1,830			190
Neisseria gonorrhoeae	95.8						32.4		92.7										
Proteus spp.	2,460	448	2,300				642		666								1,600		
Pseudomonas aeruginosa	19,500	6,500		12,700		7,500	11,900	13,500	6,570										
Salmonella Paratyphi	0.934								0.757			0.189							
Salmonella Typhi	29,000								22,700			9,000							
Non−typhoidal Salmonella	101								101										
Serratia spp.	6,090	2,110		889		1,630	3,430	5,430	1,290										
Shigella spp.	144								144										
Staphylococcus aureus	60,400								45,100		38,900			16,000			45,000	822	
Streptococcus pneumoniae	162,000				110,000	24,500	16,000		140,000		113,000				110,000		134,000		
All pathogens	631,000	140,000	84,600	34,600	237,000	54,700	149,000	44,000	404,000	39,100	191,000	9,000	14,200	16,000	112,000	1,830	329,000	2,680	190

Central African Republic

DALYs (count) *attributable to* bacterial antimicrobial resistance by pathogen−drug combinations, 2019

	Resistance to 1+	AG	Aminopenicillin	Anti−pseudomonal	BL−BLI	CP	3GC	4GC	FQ	Mono INH	Macrolide	MDR in S. Typhi and Paratyphi	MDR excluding XDR in TB	Methicillin	PCN	Mono RIF	TMP−SMX	Vanco	XDR in TB
Acinetobacter baumannii	4,750	225		2,490	25.8	816	196	7.6	996										
Citrobacter spp.	580	26.5		220		62.8	59.2	120	90.6										
Enterobacter spp.	3,490	271		364		694		1,040	660								473		
Enterococcus faecalis	1,750								1,640									112	
Enterococcus faecium	1,070								788									282	
Other enterococci	366								328									37.9	
Escherichia coli	17,800	2,670	1,280		1,940	2,120	2,660		3,700								3,440		
Group A Streptococcus	121										117								
Group B Streptococcus	10,900								7,290		3,200				314				
Haemophilus influenzae	4,530		2,440				2,090												
Klebsiella pneumoniae	24,500	6,030			845	1,050	8,290		4,940								3,360		
Morganella spp.	12.5						0.45	5.66	6.41										
Mycobacterium tuberculosis	15,200									6,320			8,310			507			115
Neisseria gonorrhoeae	13.7						5.78		7.94										
Proteus spp.	321	22	48.7				143		46.1								62.4		
Pseudomonas aeruginosa	4,970	187		1,770		1,430	325	566	689										
Salmonella Paratyphi	0.181								0.157			0.024							
Salmonella Typhi	5,570								4,530			1,080							
Non−typhoidal Salmonella	21.1								21.1										
Serratia spp.	1,550	107		141		416	82.5	678	124										
Shigella spp.	30.5								30.5										
Staphylococcus aureus	11,100								1,730		1,160			3,840			4,100	239	
Streptococcus pneumoniae	38,600				4,800	4,630	1,240		15,100		3,160				2,290		7,560		
All pathogens	147,000	9,550	3,770	4,990	7,610	11,200	15,100	2,420	42,700	5,890	7,570	1,010	8,310	3,840	2,610	507	19,000	671	115

Counts
- **<2.5k**
- **2.5k − <5k**
- **5k − <10k**
- **10k − <25k**
- **25k − <75k**
- **75k − <150k**

Chad

DALYs (count) *associated with* bacterial antimicrobial resistance by pathogen−drug combinations, 2019

	Resistance to 1+	AG	Aminopenicillin	Anti−pseudomonal	BL−BLI	CP	3GC	4GC	FQ	Mono INH	Macrolide	MDR in S. Typhi and Paratyphi	MDR excluding XDR in TB	Methicillin	PCN	Mono RIF	TMP−SMX	Vanco	XDR in TB
Acinetobacter baumannii	61,200	24,900		60,800	32,400	21,100	36,400	26,100	20,300										
Citrobacter spp.	17,500	7,090		7,460		6,840	15,500	15,300	6,440										
Enterobacter spp.	75,000	32,100		27,700		16,400		67,100	46,800								57,400		
Enterococcus faecalis	16,500								16,100									1,260	
Enterococcus faecium	13,000								12,900									3,370	
Other enterococci	10,900								10,800									624	
Escherichia coli	246,000	71,400	241,000		193,000	27,500	136,000		163,000								211,000		
Group A Streptococcus	3,930										3,930								
Group B Streptococcus	173,000								114,000		114,000				5,650				
Haemophilus influenzae	45,800		38,000				19,500												
Klebsiella pneumoniae	297,000	219,000			280,000	5,550	279,000		195,000								267,000		
Morganella spp.	95.5						23.7	61.1	69.1										
Mycobacterium tuberculosis	34,100									12,600			21,000			292			281
Neisseria gonorrhoeae	148						0.307		147										
Proteus spp.	7,400	2,690	6,380				1,950		4,320								5,850		
Pseudomonas aeruginosa	66,500	31,100		37,700		20,600	42,800	38,200	30,900										
Salmonella Paratyphi	23.6								21.7			2.08							
Salmonella Typhi	29,900								10,900			19,900							
Non−typhoidal Salmonella	301								301										
Serratia spp.	31,700	17,200		4,420		1,480	27,600	26,600	8,600										
Shigella spp.	13,300								13,300										
Staphylococcus aureus	133,000								16,200		81,700			52,000			92,600	4,190	
Streptococcus pneumoniae	571,000				106,000	121,000	57,200		191,000		158,000				73,200		533,000		
All pathogens	1,850,000	406,000	285,000	138,000	612,000	220,000	616,000	173,000	860,000	12,600	358,000	20,000	21,000	52,000	78,800	292	1,170,000	9,440	281

Counts
- <2.5k
- 2.5k − <5k
- 5k − <10k
- 10k − <25k
- 25k − <75k
- 75k − <150k
- >=150k

Chad

DALYs (count) *attributable to* bacterial antimicrobial resistance by pathogen−drug combinations, 2019

	Resistance to 1+	AG	Aminopenicillin	Anti−pseudomonal	BL−BLI	CP	3GC	4GC	FQ	Mono INH	Macrolide	MDR in S. Typhi and Paratyphi	MDR excluding XDR in TB	Methicillin	PCN	Mono RIF	TMP−SMX	Vanco	XDR in TB
Acinetobacter baumannii	17,300	1,330		9,470	0.419	3,930	2.96	1.14	2,610										
Citrobacter spp.	5,330	380		956		1,530	388	1,320	764										
Enterobacter spp.	18,600	1,630		2,040		3,200		4,820	4,390								2,540		
Enterococcus faecalis	4,370								4,020									349	
Enterococcus faecium	3,270								2,480									790	
Other enterococci	2,240								2,110									126	
Escherichia coli	63,000	4,000	2,930		6,830	5,360	16,100		15,900								11,800		
Group A Streptococcus	373										388								
Group B Streptococcus	30,600								19,900		9,730				1,010				
Haemophilus influenzae	10,600		5,060				5,540												
Klebsiella pneumoniae	81,800	15,600			1,740	1,920	39,300		11,700								11,500		
Morganella spp.	22.3						0.905	10.3	11.1										
Mycobacterium tuberculosis	14,300									2,030			12,200			79.6			169
Neisseria gonorrhoeae	14.4						0.145		14.3										
Proteus spp.	1,080	114	110				372		286								201		
Pseudomonas aeruginosa	16,500	879		5,210		3,520	2,010	1,480	3,330										
Salmonella Paratyphi	4.81								4.54			0.267							
Salmonella Typhi	4,850								2,230			2,640							
Non−typhoidal Salmonella	62.6								62.6										
Serratia spp.	8,460	962		907		420	1,030	4,280	837										
Shigella spp.	2,780								2,780										
Staphylococcus aureus	26,700								700		3,070			11,800			10,000	1,080	
Streptococcus pneumoniae	109,000				3,080	24,400	1,630		24,100		5,360				1,790		48,900		
All pathogens	422,000	24,900	8,100	18,600	11,700	44,300	66,400	11,900	98,200	1,810	18,700	2,730	12,200	11,800	2,800	79.6	85,000	2,340	169

Counts
- <2.5k
- 2.5k − <5k
- 5k − <10k
- 10k − <25k
- 25k − <75k
- 75k − <150k
- >=150k

Comoros

DALYs (count) *associated with* bacterial antimicrobial resistance by pathogen−drug combinations, 2019

	Resistance to 1+	AG	Aminopenicillin	Anti−pseudomonal	BL−BLI	CP	3GC	4GC	FQ	Mono INH	Macrolide	MDR in S. Typhi and Paratyphi	MDR excluding XDR in TB	Methicillin	PCN	Mono RIF	TMP−SMX	Vanco	XDR in TB
Acinetobacter baumannii	2,150	766		2,100	1,490	670	1,730	793	961										
Citrobacter spp.	344	130		155		64.6	314	256	205										
Enterobacter spp.	1,580	731		655		247		1,420	998								1,200		
Enterococcus faecalis	437								428									34.2	
Enterococcus faecium	412								409									94.5	
Other enterococci	244								243									15.7	
Escherichia coli	3,590	798	3,440		2,640	394	1,790		1,550								2,970		
Group A Streptococcus	118										118								
Group B Streptococcus	1,420								521		1,220				54.1				
Haemophilus influenzae	428		379				123												
Klebsiella pneumoniae	4,310	3,160			4,110	39.5	3,040		3,150								3,860		
Morganella spp.	5.14						1.5	3.05	3.79										
Mycobacterium tuberculosis	1,900									131			1,710			35.5			23
Neisseria gonorrhoeae	6.45						0.074		6.43										
Proteus spp.	256	151	205				54.6		165								229		
Pseudomonas aeruginosa	1,330	537		447		327	1,070	476	542										
Salmonella Paratyphi	0.043								0.019			0.024							
Salmonella Typhi	806								263			615							
Non−typhoidal Salmonella	2.38								2.38										
Serratia spp.	497	238		161		25.7	307	396	134										
Shigella spp.	54.1								54.1										
Staphylococcus aureus	2,580								702		1,360			886			1,840	51.4	
Streptococcus pneumoniae	6,500				943	1,930	925		1,130		819				2,300		6,240		
All pathogens	29,000	6,510	4,030	3,520	9,180	3,700	9,360	3,340	11,500	131	3,510	615	1,710	886	2,360	35.5	16,300	196	23

Counts
- <2.5k
- 2.5k − <5k
- 5k − <10k
- 10k − <25k
- 25k − <75k

Comoros

DALYs (count) *attributable to* bacterial antimicrobial resistance by pathogen−drug combinations, 2019

	Resistance to 1+	AG	Aminopenicillin	Anti−pseudomonal	BL−BLI	CP	3GC	4GC	FQ	Mono INH	Macrolide	MDR in S. Typhi and Paratyphi	MDR excluding XDR in TB	Methicillin	PCN	Mono RIF	TMP−SMX	Vanco	XDR in TB
Acinetobacter baumannii	632	39.3		338	0.429	117	11	0.048	127										
Citrobacter spp.	103	6.02		23.4		12.5	13	21.4	26.7										
Enterobacter spp.	384	37.3		53.7		48.5		99.9	92.7								52		
Enterococcus faecalis	116								106									9.42	
Enterococcus faecium	103								80									22.7	
Other enterococci	50.6								47.4									3.14	
Escherichia coli	876	48.1	55.1		117	85.7	236		148								185		
Group A Streptococcus	10.9										11.2								
Group B Streptococcus	212								85.8		116				10.1				
Haemophilus influenzae	92.1		57.1				35												
Klebsiella pneumoniae	1,080	219			102	19	383		188								164		
Morganella spp.	1.21						0.079	0.52	0.614										
Mycobacterium tuberculosis	989									19.2			946			9.51			13.6
Neisseria gonorrhoeae	0.642						0.02		0.621										
Proteus spp.	37.4	6.57	3.26				9.7		10.3								7.7		
Pseudomonas aeruginosa	337	15.9		60.3		56.8	119	23.5	61.6										
Salmonella Paratyphi	0.007								0.004			0.003							
Salmonella Typhi	125								49.2			76.9							
Non−typhoidal Salmonella	0.477								0.477										
Serratia spp.	121	13.6		31.4		7.91	8.67	46.7	13.2										
Shigella spp.	10.9								10.9										
Staphylococcus aureus	490								27.8		50.3			195			203	13.5	
Streptococcus pneumoniae	1,310				16.4	411	19.4		130		29.5				108		590		
All pathogens	7,070	386	115	506	236	758	836	192	1,210	19.5	208	76	946	195	118	9.51	1,200	48.8	13.6

Counts
- <2.5k
- 5k − <10k

Congo (Brazzaville)

DALYs (count) *associated with* bacterial antimicrobial resistance by pathogen−drug combinations, 2019

	Resistance to 1+	AG	Aminopenicillin	Anti−pseudomonal	BL−BLI	CP	3GC	4GC	FQ	Mono INH	Macrolide	MDR in S. Typhi and Paratyphi	MDR excluding XDR in TB	Methicillin	PCN	Mono RIF	TMP−SMX	Vanco	XDR in TB
Acinetobacter baumannii	8,290	3,450		8,040	4,870	4,480	7,360	5,060	5,170										
Citrobacter spp.	1,070	295		518		128	893	718	505										
Enterobacter spp.	6,240	2,480		2,680		1,290		5,030	3,220								4,770		
Enterococcus faecalis	3,330								3,270									280	
Enterococcus faecium	3,280								3,260									810	
Other enterococci	1,730								1,720									93.4	
Escherichia coli	23,800	7,910	21,400		17,300	3,270	12,200		12,800								20,700		
Group A Streptococcus	643										643								
Group B Streptococcus	10,800								6,090		7,900				490				
Haemophilus influenzae	1,830		1,550				620												
Klebsiella pneumoniae	30,000	21,400			27,400	895	24,300		23,600								27,200		
Morganella spp.	30						7.64	18.3	22.5										
Mycobacterium tuberculosis	6,750									2,730			3,710			249			50.2
Neisseria gonorrhoeae	74.6						0.534		74.5										
Proteus spp.	1,630	448	1,450				405		754								1,280		
Pseudomonas aeruginosa	9,820	4,070		5,380		4,420	6,760	4,340	4,950										
Salmonella Paratyphi	1.06								1.03			0.034							
Salmonella Typhi	5,740								3,970			2,450							
Non−typhoidal Salmonella	19.1								19.1										
Serratia spp.	2,560	1,090		461		216	1,470	2,050	596										
Shigella spp.	252								252										
Staphylococcus aureus	20,500								10,700		8,720			11,500			13,500	222	
Streptococcus pneumoniae	29,400				10,500	6,380	3,550		6,850		9,290				10,400		27,600		
All pathogens	168,000	41,100	24,500	17,100	60,100	21,100	57,600	17,200	87,800	2,730	26,600	2,450	3,710	11,500	10,900	249	95,100	1,400	50.2

Counts

- **<2.5k**
- **2.5k − <5k**
- **5k − <10k**
- **10k − <25k**
- **25k − <75k**
- **75k − <150k**
- **>=150k**

Congo (Brazzaville)

DALYs (count) *attributable to* bacterial antimicrobial resistance by pathogen−drug combinations, 2019

	Resistance to 1+	AG	Aminopenicillin	Anti−pseudomonal	BL−BLI	CP	3GC	4GC	FQ	Mono INH	Macrolide	MDR in S. Typhi and Paratyphi	MDR excluding XDR in TB	Methicillin	PCN	Mono RIF	TMP−SMX	Vanco	XDR in TB
Acinetobacter baumannii	2,540	158		887	0.107	762	65	0.207	664										
Citrobacter spp.	312	15.5		93.5		27.8	44	64.3	67.8										
Enterobacter spp.	1,490	134		229		264		324	306								237		
Enterococcus faecalis	885								810									75.8	
Enterococcus faecium	821								630									191	
Other enterococci	353								335									18.1	
Escherichia coli	5,950	462	243		654	683	1,440		1,200								1,270		
Group A Streptococcus	64.5										59.1								
Group B Streptococcus	1,830								1,040		680				84.9				
Haemophilus influenzae	406		228				178												
Klebsiella pneumoniae	7,850	1,470			406	283	3,050		1,450								1,190		
Morganella spp.	7.02						0.327	3.06	3.64										
Mycobacterium tuberculosis	2,660									433			2,130			67.5			30
Neisseria gonorrhoeae	7.41						0.151		7.26										
Proteus spp.	226	20	25.7				82.1		49.8								47		
Pseudomonas aeruginosa	2,500	116		580		779	394	91.3	537										
Salmonella Paratyphi	0.219								0.214			0.005							
Salmonella Typhi	1,050								770			269							
Non−typhoidal Salmonella	3.83								3.83										
Serratia spp.	618	62.8		92		62.5	57.7	285	59.5										
Shigella spp.	51.7								51.7										
Staphylococcus aureus	4,800								435		288			2,650			1,350	63.5	
Streptococcus pneumoniae	5,890				404	1,320	155		797		309				489		2,420		
All pathogens	40,300	2,440	498	1,880	1,460	4,180	5,470	768	9,230	485	1,370	283	2,130	2,650	574	67.5	6,510	348	30

Counts
- **<2.5k**
- **2.5k − <5k**
- **5k − <10k**
- **25k − <75k**

Cote d'Ivoire

DALYs (count) *associated with* bacterial antimicrobial resistance by pathogen−drug combinations, 2019

	Resistance to 1+	AG	Aminopenicillin	Anti−pseudomonal	BL−BLI	CP	3GC	4GC	FQ	Mono INH	Macrolide	MDR in S. Typhi and Paratyphi	MDR excluding XDR in TB	Methicillin	PCN	Mono RIF	TMP−SMX	Vanco	XDR in TB
Acinetobacter baumannii	51,800	22,800		51,500	21,600	20,700	32,800	28,000	22,700										
Citrobacter spp.	8,210	3,140		3,730		1,260	7,400	6,650	3,700										
Enterobacter spp.	50,100	26,500		20,300		9,140		42,600	31,100								41,200		
Enterococcus faecalis	24,100								23,800									1,560	
Enterococcus faecium	19,200								19,100									4,520	
Other enterococci	12,800								12,800									607	
Escherichia coli	214,000	72,200	205,000		175,000	23,800	115,000		116,000								196,000		
Group A Streptococcus	4,620										4,620								
Group B Streptococcus	113,000								75,300		73,400				2,220				
Haemophilus influenzae	18,500		13,400				8,730												
Klebsiella pneumoniae	306,000	220,000			284,000	11,300	258,000		227,000								291,000		
Morganella spp.	132						33.8	82.6	97.5										
Mycobacterium tuberculosis	34,700									12,000			22,300			106			298
Neisseria gonorrhoeae	283						0.121		282										
Proteus spp.	9,280	2,770	8,090				2,440		4,940								7,430		
Pseudomonas aeruginosa	90,200	43,800		47,100		37,300	55,600	41,500	51,600										
Salmonella Paratyphi	13								9.59			3.59							
Salmonella Typhi	91,700								14,600			84,100							
Non−typhoidal Salmonella	196								196										
Serratia spp.	26,900	13,200		3,620		1,280	21,800	24,000	6,920										
Shigella spp.	822								822										
Staphylococcus aureus	196,000								39,300		89,200			80,000			147,000	2,270	
Streptococcus pneumoniae	309,000				56,300	74,500	56,400		25,000		61,000				93,800		299,000		
All pathogens	1,580,000	404,000	226,000	126,000	537,000	179,000	558,000	143,000	675,000	12,000	228,000	84,100	22,300	80,000	96,000	106	981,000	8,950	298

Counts
- **<2.5k**
- **2.5k − <5k**
- **5k − <10k**
- **10k − <25k**
- **25k − <75k**
- **75k − <150k**
- **>=150k**

Cote d'Ivoire

DALYs (count) *attributable to* bacterial antimicrobial resistance by pathogen−drug combinations, 2019

	Resistance to 1+	AG	Aminopenicillin	Anti−pseudomonal	BL−BLI	CP	3GC	4GC	FQ	Mono INH	Macrolide	MDR in S. Typhi and Paratyphi	MDR excluding XDR in TB	Methicillin	PCN	Mono RIF	TMP−SMX	Vanco	XDR in TB
Acinetobacter baumannii	14,900	1,160		7,200	0.306	3,660	4.51	1.03	2,900										
Citrobacter spp.	2,440	159		631		268	256	656	466										
Enterobacter spp.	12,100	1,400		1,530		1,750		2,720	2,880								1,860		
Enterococcus faecalis	6,330								5,910									415	
Enterococcus faecium	4,780								3,720									1,060	
Other enterococci	2,600								2,480									121	
Escherichia coli	53,500	4,210	1,860		6,310	4,780	13,800		10,800								11,800		
Group A Streptococcus	432										417								
Group B Streptococcus	20,000								13,200		6,600				455				
Haemophilus influenzae	4,370		1,850				2,530												
Klebsiella pneumoniae	81,100	15,300			3,430	3,210	32,500		13,700								12,900		
Morganella spp.	30.8						1.35	13.8	15.7										
Mycobacterium tuberculosis	14,700									1,760			12,700			28.5			178
Neisseria gonorrhoeae	27.5						0.096		27.4										
Proteus spp.	1,340	120	145				479		326								255		
Pseudomonas aeruginosa	22,000	1,240		5,100		6,210	2,760	1,060	5,700										
Salmonella Paratyphi	2.5								2.01			0.49							
Salmonella Typhi	13,200								2,570			11,000							
Non−typhoidal Salmonella	40.1								40.1										
Serratia spp.	7,080	753		762		370	548	3,970	687										
Shigella spp.	170								170										
Staphylococcus aureus	40,000								1,530		3,240			18,200			16,400	614	
Streptococcus pneumoniae	59,300				1,290	16,400	2,840		2,860		2,360				4,180		29,400		
All pathogens	361,000	24,300	3,850	15,200	11,000	36,700	55,700	8,410	70,100	1,910	12,200	10,600	12,700	18,200	4,640	28.5	72,700	2,210	178

Counts
- <2.5k
- 2.5k − <5k
- 5k − <10k
- 10k − <25k
- 25k − <75k
- 75k − <150k
- >=150k

DR Congo

DALYs (count) *associated with* bacterial antimicrobial resistance by pathogen−drug combinations, 2019

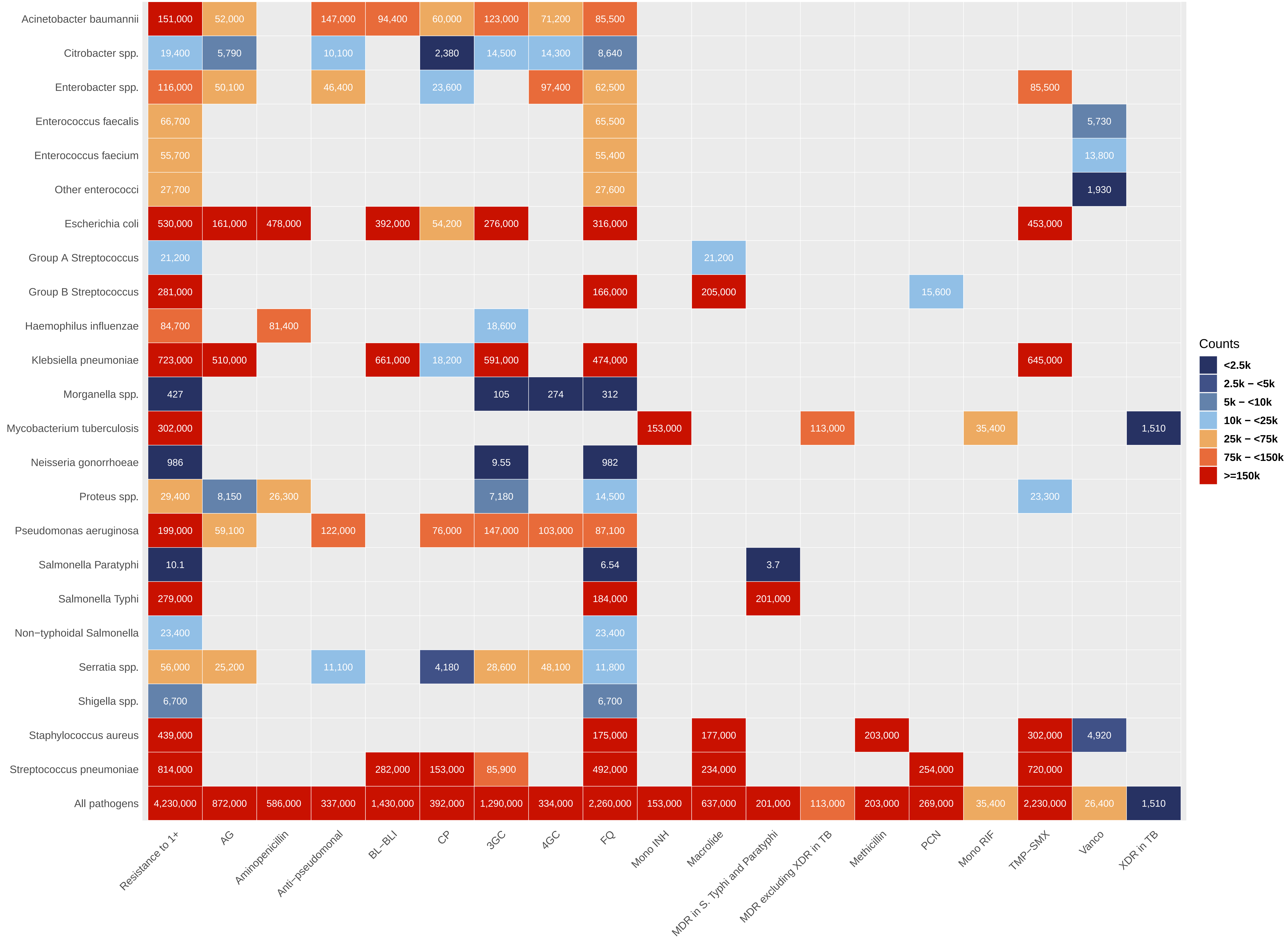

DR Congo

DALYs (count) *attributable to* bacterial antimicrobial resistance by pathogen−drug combinations, 2019

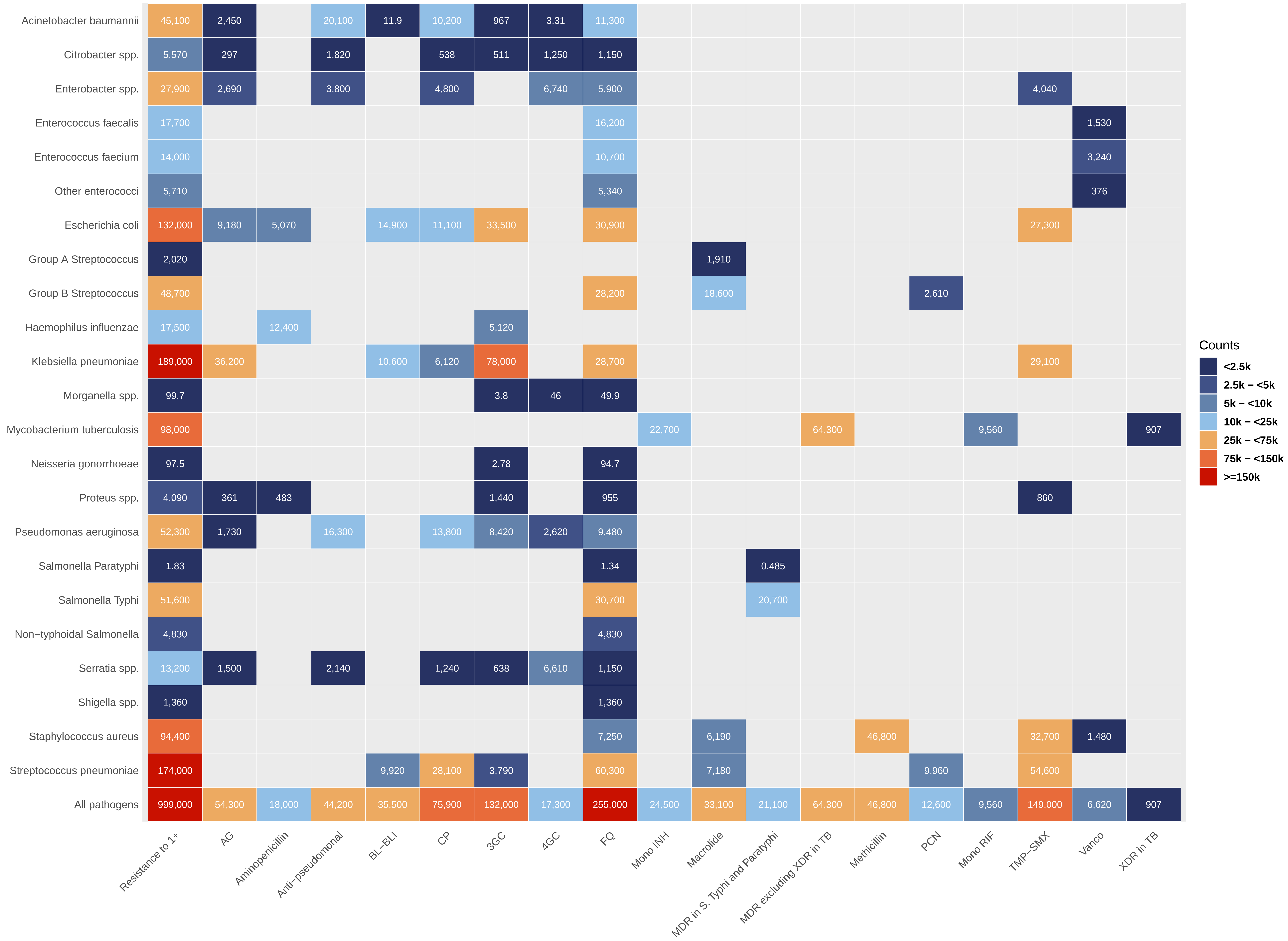

Equatorial Guinea

DALYs (count) *associated with* bacterial antimicrobial resistance by pathogen−drug combinations, 2019

	Resistance to 1+	AG	Aminopenicillin	Anti−pseudomonal	BL−BLI	CP	3GC	4GC	FQ	Mono INH	Macrolide	MDR in S. Typhi and Paratyphi	MDR excluding XDR in TB	Methicillin	PCN	Mono RIF	TMP−SMX	Vanco	XDR in TB
Acinetobacter baumannii	2,970	1,670		2,890	2,190	2,050	2,740	2,190	2,540										
Citrobacter spp.	561	45.1		213		134	426	318	277										
Enterobacter spp.	2,870	638		1,270		560		1,740	1,270								2,280		
Enterococcus faecalis	548								530									49.7	
Enterococcus faecium	642								638									172	
Other enterococci	617								616									30.9	
Escherichia coli	3,580	1,300	3,290		2,490	282	1,840		2,010								3,020		
Group A Streptococcus	133										133								
Group B Streptococcus	2,240								953		1,820				95.5				
Haemophilus influenzae	282		201				130												
Klebsiella pneumoniae	4,940	3,140			4,580	122	3,330		3,740								4,260		
Morganella spp.	3.36						0.834	0.707	3.07										
Mycobacterium tuberculosis	974									462			474			32.3			6.22
Neisseria gonorrhoeae	22.9						0.164		22.8										
Proteus spp.	298	96.7	265				62		132								234		
Pseudomonas aeruginosa	2,010	808		1,060		1,090	1,180	788	1,220										
Salmonella Paratyphi	0.244								0.222			0.025							
Salmonella Typhi	710								485			327							
Non−typhoidal Salmonella	6.38								6.38										
Serratia spp.	656	303		149		81.3	336	433	187										
Shigella spp.	13.9								13.9										
Staphylococcus aureus	2,880								1,840		839			1,740			1,740	33	
Streptococcus pneumoniae	6,850				2,300	1,540	879		1,160		2,270				3,400		6,270		
All pathogens	33,800	8,000	3,760	5,580	11,600	5,860	10,900	5,470	17,700	462	5,070	327	474	1,740	3,490	32.3	17,800	285	6.22

Counts
- <2.5k
- 2.5k − <5k
- 5k − <10k
- 10k − <25k
- 25k − <75k

Equatorial Guinea

DALYs (count) *attributable to* bacterial antimicrobial resistance by pathogen−drug combinations, 2019

	Resistance to 1+	AG	Aminopenicillin	Anti−pseudomonal	BL−BLI	CP	3GC	4GC	FQ	Mono INH	Macrolide	MDR in S. Typhi and Paratyphi	MDR excluding XDR in TB	Methicillin	PCN	Mono RIF	TMP−SMX	Vanco	XDR in TB
Acinetobacter baumannii	933	70.5		181	0.198	331	20.2	0.119	330										
Citrobacter spp.	164	2.2		34.3		30.2	28.7	27.9	40.5										
Enterobacter spp.	636	37.5		123		118		95.9	131								131		
Enterococcus faecalis	147								132									14.8	
Enterococcus faecium	163								122									40.2	
Other enterococci	126								120									5.96	
Escherichia coli	876	75.6	47.1		98.1	57.9	229		191								178		
Group A Streptococcus	12.4										13.2								
Group B Streptococcus	348								161		166				17.2				
Haemophilus influenzae	66.5		28.6				37.8												
Klebsiella pneumoniae	1,220	217			123	42.2	421		233								189		
Morganella spp.	0.758						0.092	0.116	0.55										
Mycobacterium tuberculosis	335									65.5			257			8.41			3.63
Neisseria gonorrhoeae	2.28						0.046		2.23										
Proteus spp.	39.3	4.24	4.95				12.7		8.67								8.45		
Pseudomonas aeruginosa	497	20.9		88.6		186	54.9	12.6	134										
Salmonella Paratyphi	0.049								0.046			0.003							
Salmonella Typhi	129								92.5			38.1							
Non−typhoidal Salmonella	1.31								1.31										
Serratia spp.	153	17.4		27.9		21.4	15.7	51	19.2										
Shigella spp.	2.68								2.68										
Staphylococcus aureus	699								79.3		26.7			412			172	9.7	
Streptococcus pneumoniae	1,410				86.1	323	39.6		130		81.9				209		544		
All pathogens	7,960	445	80.8	455	308	1,110	859	188	1,930	66.6	286	35.9	257	412	226	8.41	1,220	70.6	3.63

Counts
- <2.5k
- 5k − <10k

Eritrea

DALYs (count) *associated with* bacterial antimicrobial resistance by pathogen−drug combinations, 2019

	Resistance to 1+	AG	Aminopenicillin	Anti−pseudomonal	BL−BLI	CP	3GC	4GC	FQ	Mono INH	Macrolide	MDR in S. Typhi and Paratyphi	MDR excluding XDR in TB	Methicillin	PCN	Mono RIF	TMP−SMX	Vanco	XDR in TB
Acinetobacter baumannii	14,800	4,740		13,900	10,500	3,950	12,400	5,900	5,990										
Citrobacter spp.	1,400	594		677		270	1,240	1,060	832										
Enterobacter spp.	8,170	3,790		3,300		1,390		7,570	4,380								6,130		
Enterococcus faecalis	5,150								5,060									395	
Enterococcus faecium	4,450								4,410									1,020	
Other enterococci	1,450								1,450									82.4	
Escherichia coli	48,000	11,300	46,400		35,900	4,650	24,400		24,300								39,000		
Group A Streptococcus	1,630										1,630								
Group B Streptococcus	15,000								7,170		12,200				578				
Haemophilus influenzae	6,650		6,400				1,500												
Klebsiella pneumoniae	60,100	45,900			57,300	710	42,400		39,500								54,200		
Morganella spp.	38						11	23.8	27.7										
Mycobacterium tuberculosis	28,200									8,060			17,000			2,960			227
Neisseria gonorrhoeae	55						0.643		54.7										
Proteus spp.	2,550	1,080	2,100				547		1,670								2,290		
Pseudomonas aeruginosa	15,700	5,660		5,280		3,270	13,500	6,630	4,650										
Salmonella Paratyphi	0.682								0.138			0.54							
Salmonella Typhi	19,000								9,930			11,500							
Non−typhoidal Salmonella	13.6								13.6										
Serratia spp.	4,140	2,400		1,410		201	2,870	2,980	1,080										
Shigella spp.	2,150								2,150										
Staphylococcus aureus	46,500								11,800		23,300			12,600			34,800	563	
Streptococcus pneumoniae	66,800				10,600	14,800	8,720		33,200		7,500				13,200		61,400		
All pathogens	352,000	75,500	54,900	24,500	114,000	29,200	108,000	24,200	158,000	8,060	44,600	11,500	17,000	12,600	13,800	2,960	198,000	2,060	227

Counts
- <2.5k
- 2.5k − <5k
- 5k − <10k
- 10k − <25k
- 25k − <75k
- 75k − <150k
- >=150k

Eritrea

DALYs (count) *attributable to* bacterial antimicrobial resistance by pathogen−drug combinations, 2019

	Resistance to 1+	AG	Aminopenicillin	Anti−pseudomonal	BL−BLI	CP	3GC	4GC	FQ	Mono INH	Macrolide	MDR in S. Typhi and Paratyphi	MDR excluding XDR in TB	Methicillin	PCN	Mono RIF	TMP−SMX	Vanco	XDR in TB
Acinetobacter baumannii	4,360	251		2,320	22.2	720	236	0.296	810										
Citrobacter spp.	419	28.1		101		52.2	44.5	83.7	109										
Enterobacter spp.	1,990	200		282		280		578	384								274		
Enterococcus faecalis	1,360								1,260									106	
Enterococcus faecium	1,110								862									247	
Other enterococci	300								283									17.1	
Escherichia coli	11,800	667	707		1,570	984	3,160		2,360								2,350		
Group A Streptococcus	151										158								
Group B Streptococcus	2,400								1,180		1,150				106				
Haemophilus influenzae	1,380		968				412												
Klebsiella pneumoniae	15,000	3,240			1,440	308	5,400		2,330								2,340		
Morganella spp.	9						0.519	4.05	4.43										
Mycobacterium tuberculosis	11,700									1,230			9,570			798			135
Neisseria gonorrhoeae	5.45						0.183		5.27										
Proteus spp.	367	45.6	34.9				100		109								80.5		
Pseudomonas aeruginosa	4,060	176		802		602	1,540	425	514										
Salmonella Paratyphi	0.104								0.032			0.07							
Salmonella Typhi	3,270								1,900			1,340							
Non−typhoidal Salmonella	2.71								2.71										
Serratia spp.	1,020	142		271		60.3	108	332	105										
Shigella spp.	444								444										
Staphylococcus aureus	8,210								469		867			2,740			3,980	158	
Streptococcus pneumoniae	13,600				238	2,870	302		4,190		267				420		5,330		
All pathogens	83,000	4,750	1,710	3,780	3,260	5,880	11,300	1,420	17,300	1,250	2,390	1,340	9,570	2,740	526	798	14,300	527	135

Counts
- <2.5k
- 2.5k − <5k
- 5k − <10k
- 10k − <25k
- 75k − <150k

Ethiopia

DALYs (count) *associated with* bacterial antimicrobial resistance by pathogen−drug combinations, 2019

	Resistance to 1+	AG	Aminopenicillin	Anti−pseudomonal	BL−BLI	CP	3GC	4GC	FQ	Mono INH	Macrolide	MDR in S. Typhi and Paratyphi	MDR excluding XDR in TB	Methicillin	PCN	Mono RIF	TMP−SMX	Vanco	XDR in TB
Acinetobacter baumannii	184,000	66,500		183,000	175,000	62,600	176,000	173,000	124,000										
Citrobacter spp.	38,400	11,900		17,000		6,620	32,400	22,900	23,600										
Enterobacter spp.	192,000	84,300		72,700		21,800		170,000	103,000								142,000		
Enterococcus faecalis	86,300								84,700									6,010	
Enterococcus faecium	75,300								74,600									18,600	
Other enterococci	46,000								45,800									3,580	
Escherichia coli	723,000	108,000	680,000		456,000	41,600	473,000		458,000								619,000		
Group A Streptococcus	23,900										23,900								
Group B Streptococcus	333,000								124,000		289,000				9,560				
Haemophilus influenzae	69,000		65,200				12,900												
Klebsiella pneumoniae	940,000	684,000			913,000	346,000	918,000		535,000								833,000		
Morganella spp.	458						110	271	338										
Mycobacterium tuberculosis	111,000									14,000			84,000			11,800			1,110
Neisseria gonorrhoeae	540						19.4		535										
Proteus spp.	40,400	17,400	34,300				7,080		22,600								35,200		
Pseudomonas aeruginosa	224,000	67,000		91,000		61,200	183,000	77,500	94,700										
Salmonella Paratyphi	8.2								2.44			4.92							
Salmonella Typhi	197,000								116,000			95,300							
Non−typhoidal Salmonella	36,600								36,600										
Serratia spp.	70,700	29,900		26,300		3,740	40,200	44,600	19,400										
Shigella spp.	50,600								50,600										
Staphylococcus aureus	533,000								115,000		342,000			280,000			323,000	5,910	
Streptococcus pneumoniae	995,000				167,000	246,000	130,000		158,000		122,000				242,000		964,000		
All pathogens	4,970,000	1,070,000	780,000	389,000	1,710,000	789,000	1,970,000	488,000	2,190,000	14,000	777,000	95,300	84,000	280,000	251,000	11,800	2,920,000	34,100	1,110

Counts
- <2.5k
- 2.5k − <5k
- 5k − <10k
- 10k − <25k
- 25k − <75k
- 75k − <150k
- >=150k

Ethiopia

DALYs (count) *attributable to* bacterial antimicrobial resistance by pathogen−drug combinations, 2019

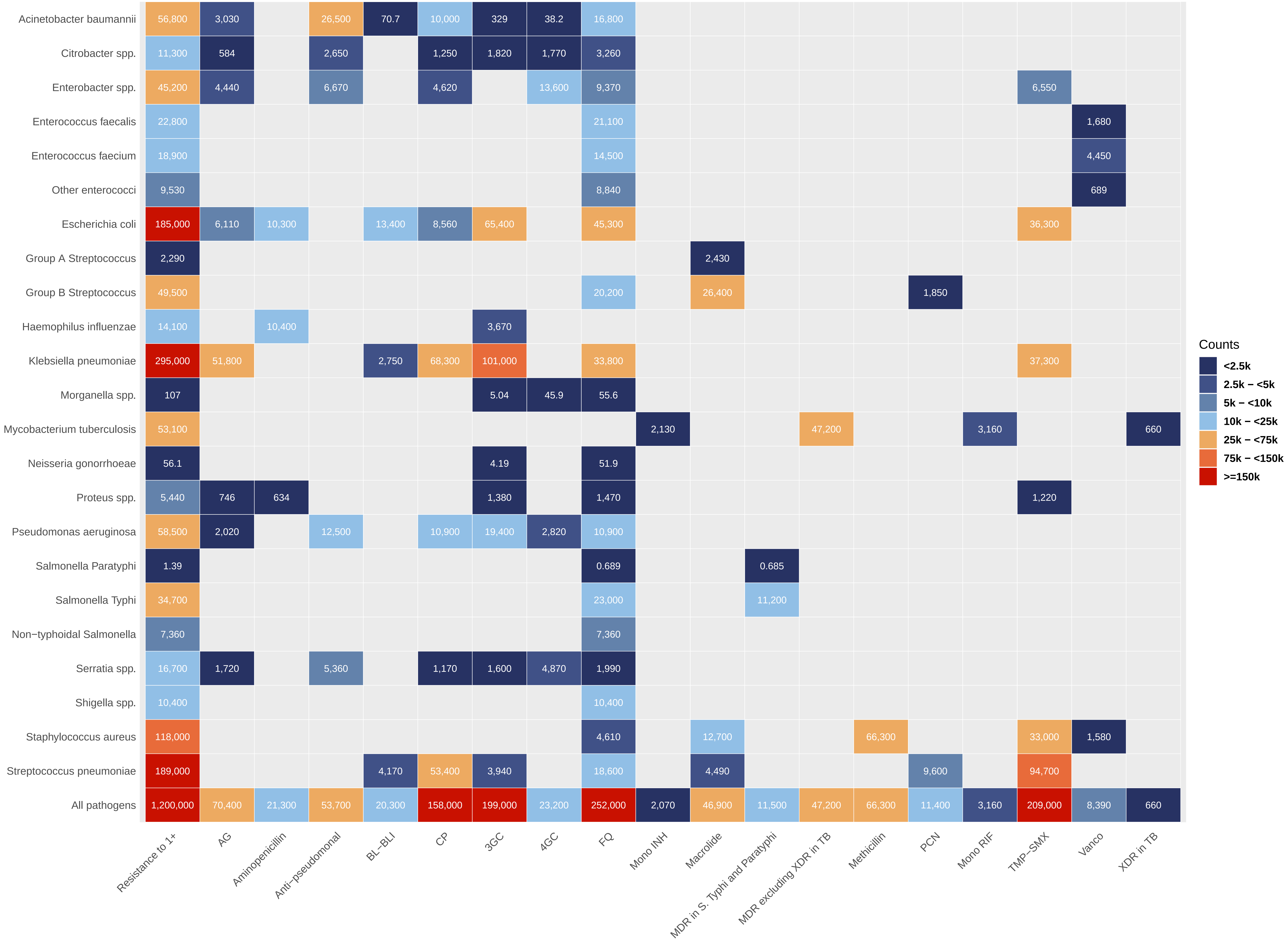

Gabon

DALYs (count) *associated with* bacterial antimicrobial resistance by pathogen−drug combinations, 2019

	Resistance to 1+	AG	Aminopenicillin	Anti−pseudomonal	BL−BLI	CP	3GC	4GC	FQ	Mono INH	Macrolide	MDR in S. Typhi and Paratyphi	MDR excluding XDR in TB	Methicillin	PCN	Mono RIF	TMP−SMX	Vanco	XDR in TB
Acinetobacter baumannii	4,120	1,980		3,900	2,730	2,310	3,680	3,110	3,190										
Citrobacter spp.	455	125		176		50.6	356	191	204										
Enterobacter spp.	2,680	769		944		462		1,680	1,320								2,070		
Enterococcus faecalis	859								844									56.8	
Enterococcus faecium	996								989									255	
Other enterococci	600								599									39.7	
Escherichia coli	5,280	2,620	4,910		3,680	522	2,360		2,570								4,300		
Group A Streptococcus	154										154								
Group B Streptococcus	1,640								567		1,380				53.2				
Haemophilus influenzae	398		347				109												
Klebsiella pneumoniae	6,170	3,660			5,390	145	4,540		4,730								5,440		
Morganella spp.	9.01						2.26	2.32	8.02										
Mycobacterium tuberculosis	1,710									512			1,140			46.3			15.2
Neisseria gonorrhoeae	27						0.168		26.9										
Proteus spp.	445	19.5	392				95		137								325		
Pseudomonas aeruginosa	2,340	861		1,250		1,180	1,330	946	1,410										
Salmonella Paratyphi	0.242								0.235			0.007							
Salmonella Typhi	613								415			267							
Non−typhoidal Salmonella	4.12								4.12										
Serratia spp.	672	243		116		192	349	506	160										
Shigella spp.	32.6								32.6										
Staphylococcus aureus	3,790								2,210		1,780			2,790			1,420	129	
Streptococcus pneumoniae	7,600				2,720	1,530	935		1,320		2,780				2,660		6,950		
All pathogens	40,600	10,300	5,650	6,380	14,500	6,390	13,800	6,430	20,700	512	6,090	267	1,140	2,790	2,710	46.3	20,500	481	15.2

Counts
- **<2.5k**
- **2.5k − <5k**
- **5k − <10k**
- **10k − <25k**
- **25k − <75k**

Gabon

DALYs (count) *attributable to* bacterial antimicrobial resistance by pathogen−drug combinations, 2019

	Resistance to 1+	AG	Aminopenicillin	Anti−pseudomonal	BL−BLI	CP	3GC	4GC	FQ	Mono INH	Macrolide	MDR in S. Typhi and Paratyphi	MDR excluding XDR in TB	Methicillin	PCN	Mono RIF	TMP−SMX	Vanco	XDR in TB
Acinetobacter baumannii	1,270	86.6		332	1.28	375	59.3	0.57	417										
Citrobacter spp.	130	7.2		33.8		10.9	31	17.4	29.8										
Enterobacter spp.	596	44.1		87		95.9		113	140								117		
Enterococcus faecalis	226								210									15.8	
Enterococcus faecium	251								191									60.2	
Other enterococci	123								116									7.66	
Escherichia coli	1,270	166	79.1		159	108	275		233								248		
Group A Streptococcus	14.5										14.6								
Group B Streptococcus	241								96.1		135				10.3				
Haemophilus influenzae	85.5		54				31.4												
Klebsiella pneumoniae	1,550	253			108	51.9	583		304								251		
Morganella spp.	2.05						0.232	0.385	1.44										
Mycobacterium tuberculosis	736									79.5			639			12.4			9.01
Neisseria gonorrhoeae	2.66						0.048		2.61										
Proteus spp.	54.9	1.31	8.54				21.7		9.99								13.7		
Pseudomonas aeruginosa	577	23.1		119		202	59.2	16.8	158										
Salmonella Paratyphi	0.05								0.049			0.001							
Salmonella Typhi	111								80.4			31.5							
Non−typhoidal Salmonella	0.809								0.809										
Serratia spp.	169	13.5		18.9		49.5	14.8	56.5	16										
Shigella spp.	6.43								6.43										
Staphylococcus aureus	1,020								96		62.6			701			132	31.9	
Streptococcus pneumoniae	1,490				112	322	49.2		156		97.4				136		616		
All pathogens	9,930	595	142	591	381	1,210	1,130	204	2,260	75.9	309	31.5	639	701	146	12.4	1,380	116	9.01

Counts
- <2.5k
- 5k − <10k

Ghana

DALYs (count) *associated with* bacterial antimicrobial resistance by pathogen−drug combinations, 2019

	Resistance to 1+	AG	Aminopenicillin	Anti−pseudomonal	BL−BLI	CP	3GC	4GC	FQ	Mono INH	Macrolide	MDR in S. Typhi and Paratyphi	MDR excluding XDR in TB	Methicillin	PCN	Mono RIF	TMP−SMX	Vanco	XDR in TB
Acinetobacter baumannii	54,500	25,100		54,200	20,700	22,700	21,300	16,900	24,800										
Citrobacter spp.	7,730	2,040		2,190		1,220	7,120	6,170	2,680										
Enterobacter spp.	38,300	19,000		13,500		9,670		31,300	26,600								29,400		
Enterococcus faecalis	22,300								21,800									1,540	
Enterococcus faecium	27,300								27,200									6,590	
Other enterococci	13,500								13,500									579	
Escherichia coli	160,000	97,900	153,000		137,000	7,270	111,000		115,000								144,000		
Group A Streptococcus	4,820										4,820								
Group B Streptococcus	38,300								14,700		31,200				594				
Haemophilus influenzae	7,020		5,100				3,080												
Klebsiella pneumoniae	231,000	181,000			213,000	3,110	191,000		164,000								218,000		
Morganella spp.	101						23.3	43.3	81.7										
Mycobacterium tuberculosis	30,200									7,120			22,700			8.79			306
Neisseria gonorrhoeae	429						0.856		428										
Proteus spp.	11,200	4,300	8,530				5,850		7,760								8,780		
Pseudomonas aeruginosa	75,000	48,600		36,700		22,200	31,000	17,700	45,600										
Salmonella Paratyphi	161								144			19.2							
Salmonella Typhi	105,000								7,450			101,000							
Non−typhoidal Salmonella	1.43								1.43										
Serratia spp.	27,900	13,500		3,640		1,530	27,700	25,300	11,700										
Shigella spp.	206								206										
Staphylococcus aureus	154,000								56,100		69,700			70,700			95,800	4,010	
Streptococcus pneumoniae	221,000				16,500	40,100	20,000		35,300		19,600				53,400		216,000		
All pathogens	1,230,000	392,000	166,000	110,000	387,000	108,000	418,000	97,500	575,000	7,120	125,000	101,000	22,700	70,700	54,000	8.79	712,000	12,700	306

Counts
- <2.5k
- 2.5k − <5k
- 5k − <10k
- 10k − <25k
- 25k − <75k
- 75k − <150k
- >=150k

Ghana

DALYs (count) *attributable to* bacterial antimicrobial resistance by pathogen−drug combinations, 2019

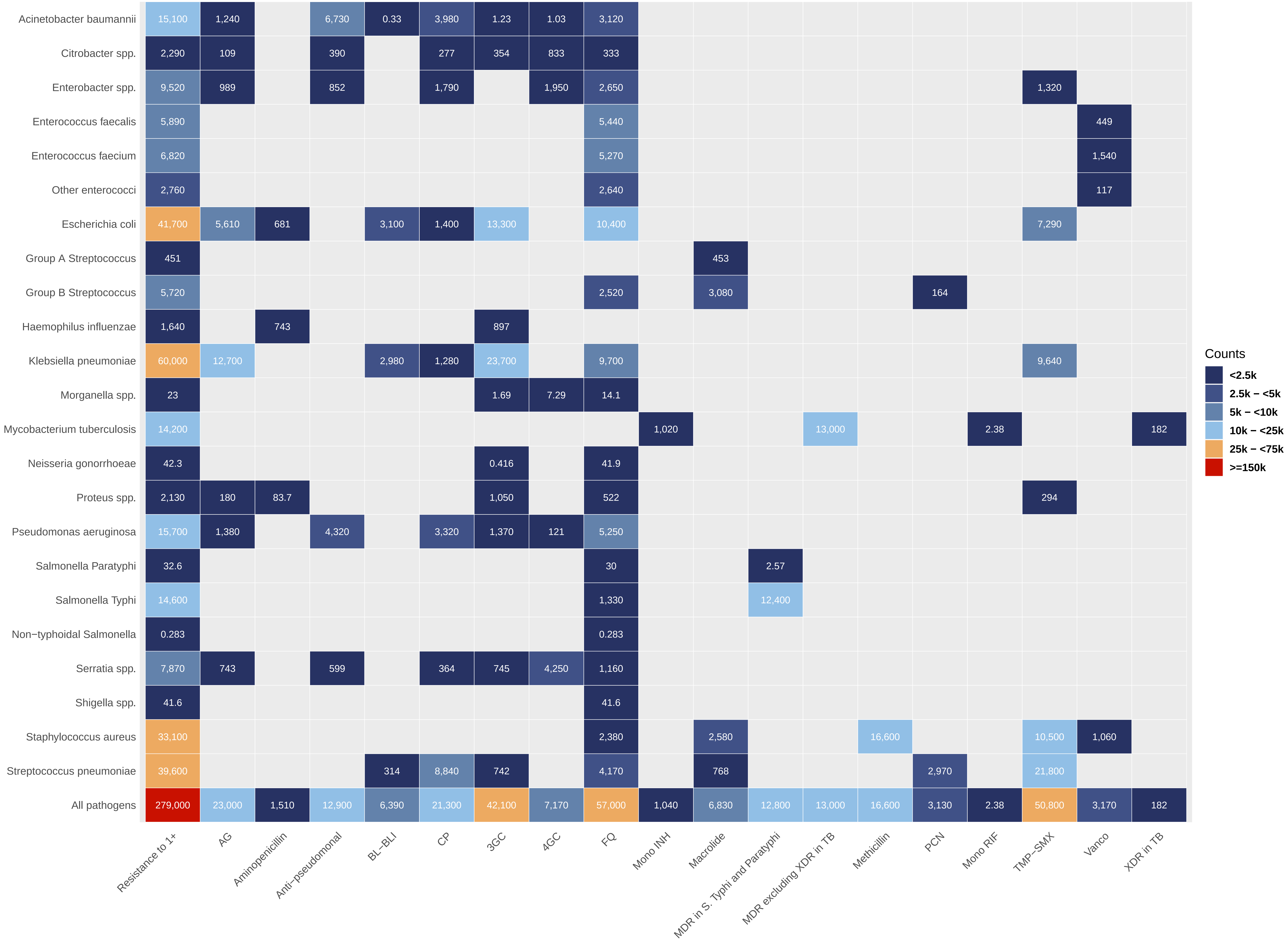

Guinea

DALYs (count) *associated with* bacterial antimicrobial resistance by pathogen−drug combinations, 2019

	Resistance to 1+	AG	Aminopenicillin	Anti−pseudomonal	BL−BLI	CP	3GC	4GC	FQ	Mono INH	Macrolide	MDR in S. Typhi and Paratyphi	MDR excluding XDR in TB	Methicillin	PCN	Mono RIF	TMP−SMX	Vanco	XDR in TB
Acinetobacter baumannii	47,400	22,700		46,600	26,700	22,100	37,100	28,600	25,700										
Citrobacter spp.	11,800	4,830		5,840		5,380	10,200	9,580	5,540										
Enterobacter spp.	49,200	24,700		20,500		10,400		43,100	28,100								38,600		
Enterococcus faecalis	13,200								13,000									859	
Enterococcus faecium	9,490								9,440									2,180	
Other enterococci	8,700								8,680									446	
Escherichia coli	122,000	35,600	118,000		95,000	14,000	63,100		66,000								109,000		
Group A Streptococcus	3,100										3,100								
Group B Streptococcus	101,000								75,100		62,100				1,870				
Haemophilus influenzae	29,100		24,100				12,200												
Klebsiella pneumoniae	178,000	128,000			168,000	6,190	148,000		123,000								165,000		
Morganella spp.	96.7						27.4	59	73.4										
Mycobacterium tuberculosis	15,800									6,370			9,300			7.75			124
Neisseria gonorrhoeae	125						0.281		125										
Proteus spp.	5,670	1,980	5,060				1,350		3,010								4,490		
Pseudomonas aeruginosa	45,600	20,600		24,400		17,300	30,300	25,100	23,800										
Salmonella Paratyphi	6.01								3.76			2.31							
Salmonella Typhi	50,700								7,120			47,300							
Non−typhoidal Salmonella	114								114										
Serratia spp.	19,700	9,050		2,810		1,080	14,600	17,500	5,210										
Shigella spp.	630								630										
Staphylococcus aureus	83,200								21,600		32,700			36,100			63,600	902	
Streptococcus pneumoniae	347,000				63,500	79,800	39,600		118,000		78,000				36,200		323,000		
All pathogens	1,140,000	248,000	147,000	100,000	353,000	156,000	357,000	124,000	535,000	6,370	176,000	47,300	9,300	36,100	38,100	7.75	704,000	4,390	124

Counts
<2.5k
2.5k − <5k
5k − <10k
10k − <25k
25k − <75k
75k − <150k
>=150k

Guinea

DALYs (count) *attributable to* bacterial antimicrobial resistance by pathogen−drug combinations, 2019

	Resistance to 1+	AG	Aminopenicillin	Anti−pseudomonal	BL−BLI	CP	3GC	4GC	FQ	Mono INH	Macrolide	MDR in S. Typhi and Paratyphi	MDR excluding XDR in TB	Methicillin	PCN	Mono RIF	TMP−SMX	Vanco	XDR in TB
Acinetobacter baumannii	14,200	1,100		5,830	0.903	3,800	142	1.09	3,300										
Citrobacter spp.	3,620	231		660		1,170	287	586	676										
Enterobacter spp.	12,100	1,310		1,580		2,050		2,820	2,550								1,740		
Enterococcus faecalis	3,470								3,240									224	
Enterococcus faecium	2,350								1,840									516	
Other enterococci	1,780								1,690									89.2	
Escherichia coli	30,500	2,070	1,480		3,650	2,830	7,620		6,250								6,550		
Group A Streptococcus	298										287								
Group B Streptococcus	18,600								13,200		4,920				369				
Haemophilus influenzae	6,690		3,230				3,460												
Klebsiella pneumoniae	47,200	9,020			2,490	1,810	19,200		7,440								7,270		
Morganella spp.	22.8						1.26	9.76	11.8										
Mycobacterium tuberculosis	6,390									989			5,320			2.12			74.2
Neisseria gonorrhoeae	12.2						0.125		12.1										
Proteus spp.	796	85	91.2				266		196								155		
Pseudomonas aeruginosa	11,400	571		2,920		2,940	1,510	859	2,590										
Salmonella Paratyphi	1.11								0.794			0.309							
Salmonella Typhi	7,100								1,190			5,790							
Non−typhoidal Salmonella	23.8								23.8										
Serratia spp.	5,060	506		586		312	367	2,780	510										
Shigella spp.	128								128										
Staphylococcus aureus	17,500								845		1,140			8,200			7,080	258	
Streptococcus pneumoniae	67,100				1,680	16,100	1,080		15,000		2,780				561		29,900		
All pathogens	256,000	14,900	4,800	11,600	7,820	31,100	33,900	7,060	60,600	980	9,300	5,930	5,320	8,200	930	2.12	52,700	1,090	74.2

Counts
- <2.5k
- 2.5k − <5k
- 5k − <10k
- 10k − <25k
- 25k − <75k
- >=150k

Guinea−Bissau

DALYs (count) *associated with* bacterial antimicrobial resistance by pathogen−drug combinations, 2019

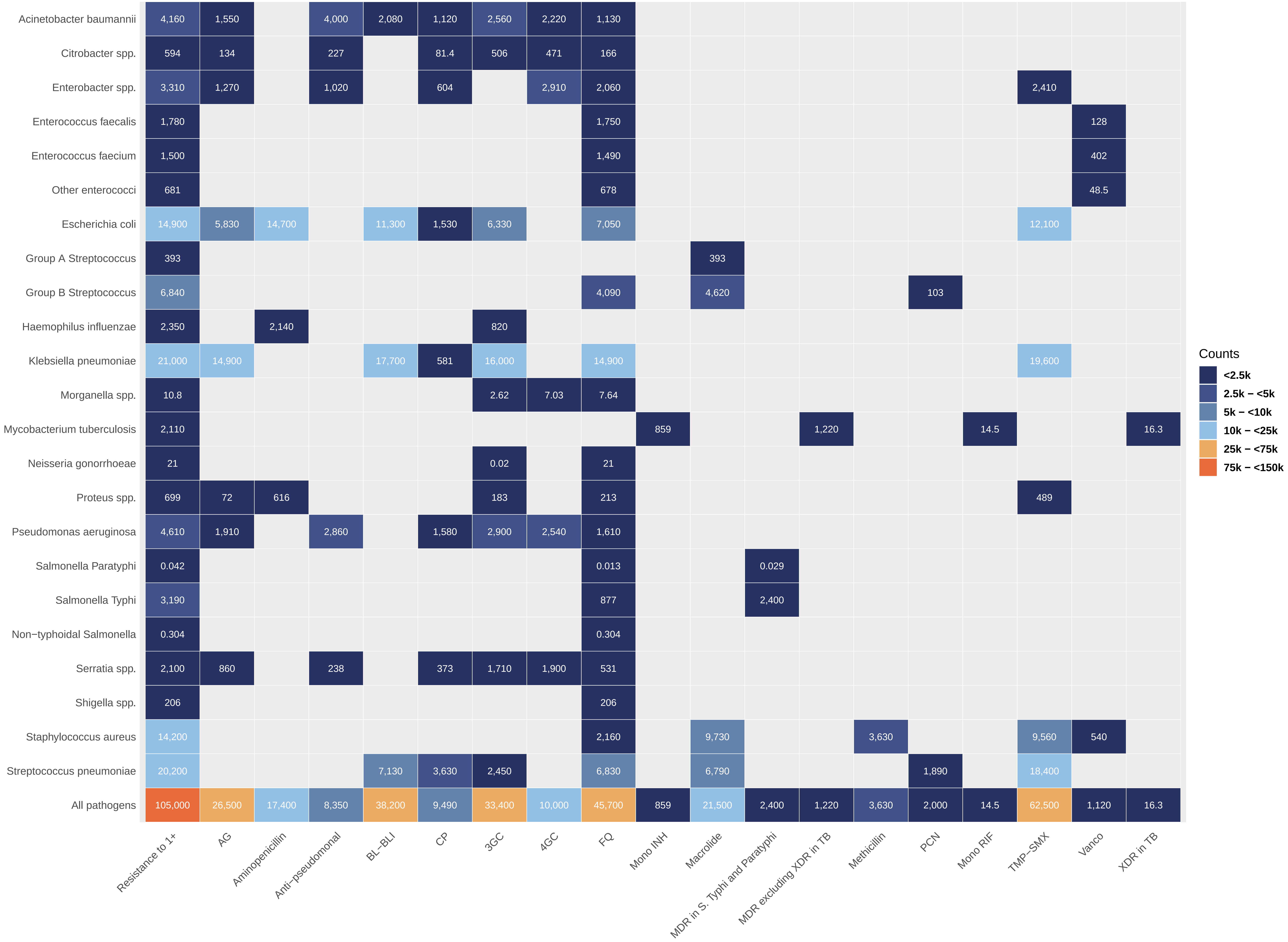

Guinea−Bissau

DALYs (count) *attributable to* bacterial antimicrobial resistance by pathogen−drug combinations, 2019

	Resistance to 1+	AG	Aminopenicillin	Anti−pseudomonal	BL−BLI	CP	3GC	4GC	FQ	Mono INH	Macrolide	MDR in S. Typhi and Paratyphi	MDR excluding XDR in TB	Methicillin	PCN	Mono RIF	TMP−SMX	Vanco	XDR in TB
Acinetobacter baumannii	1,180	91		661	1.37	238	29.8	0.16	163										
Citrobacter spp.	173	7.23		43.9		19.7	23.5	57.7	20.8										
Enterobacter spp.	806	64.2		77.7		121		237	199								107		
Enterococcus faecalis	470								435									34.8	
Enterococcus faecium	379								285									94.3	
Other enterococci	141								132									9.58	
Escherichia coli	3,590	356	268		533	317	761		661								696		
Group A Streptococcus	37.9										36.7								
Group B Streptococcus	1,160								718		440				26.2				
Haemophilus influenzae	520		292				227												
Klebsiella pneumoniae	5,360	1,050			271	194	2,020		905								914		
Morganella spp.	2.53						0.097	1.2	1.23										
Mycobacterium tuberculosis	840									126			697			3.97			9.83
Neisseria gonorrhoeae	2.04						0.013		2.03										
Proteus spp.	92.2	3.93	13				40.7		15.1								19.7		
Pseudomonas aeruginosa	1,150	56.4		418		295	126	85.1	171										
Salmonella Paratyphi	0.007								0.003			0.004							
Salmonella Typhi	500								181			317							
Non−typhoidal Salmonella	0.063								0.063										
Serratia spp.	566	47.7		43.6		92.3	41.3	290	51.6										
Shigella spp.	42.9								42.9										
Staphylococcus aureus	2,490								89.3		376			848			1,030	146	
Streptococcus pneumoniae	3,890				272	743	138		856		236				35.8		1,610		
All pathogens	23,400	1,680	573	1,240	1,080	2,020	3,410	671	4,930	128	1,060	317	697	848	62	3.97	4,380	285	9.83

Counts
- **<2.5k**
- **2.5k − <5k**
- **5k − <10k**
- **10k − <25k**

Kenya

DALYs (count) *associated with* bacterial antimicrobial resistance by pathogen−drug combinations, 2019

	Resistance to 1+	AG	Aminopenicillin	Anti−pseudomonal	BL−BLI	CP	3GC	4GC	FQ	Mono INH	Macrolide	MDR in S. Typhi and Paratyphi	MDR excluding XDR in TB	Methicillin	PCN	Mono RIF	TMP−SMX	Vanco	XDR in TB
Acinetobacter baumannii	71,100	27,400		68,000	49,900	37,600	65,400	39,600	47,500										
Citrobacter spp.	10,600	3,040		4,530		2,210	9,200	6,100	7,440										
Enterobacter spp.	56,600	24,200		24,900		8,300		48,700	33,600								43,100		
Enterococcus faecalis	39,900								39,500									1,990	
Enterococcus faecium	35,800								35,500									10,100	
Other enterococci	15,000								15,000									592	
Escherichia coli	271,000	84,700	264,000		142,000	10,600	140,000		129,000								239,000		
Group A Streptococcus	7,240										7,240								
Group B Streptococcus	87,700								43,100		70,900				764				
Haemophilus influenzae	25,100		23,800				5,010												
Klebsiella pneumoniae	302,000	204,000			249,000	9,980	221,000		213,000								286,000		
Morganella spp.	231						51	130	180										
Mycobacterium tuberculosis	57,500									26,600			23,700			6,870			315
Neisseria gonorrhoeae	398						0.32		398										
Proteus spp.	18,000	6,720	15,500				3,290		10,500								15,800		
Pseudomonas aeruginosa	83,700	20,900		61,600		35,600	55,500	33,200	25,000										
Salmonella Paratyphi	281								48			232							
Salmonella Typhi	172,000								103,000			119,000							
Non−typhoidal Salmonella	280								280										
Serratia spp.	22,800	11,800		7,670		1,610	13,700	13,100	7,330										
Shigella spp.	14,100								14,100										
Staphylococcus aureus	213,000								58,800		116,000			61,900			144,000	2,900	
Streptococcus pneumoniae	309,000				24,200	108,000	29,100		24,100		74,200				161,000		295,000		
All pathogens	1,810,000	383,000	303,000	167,000	465,000	214,000	542,000	141,000	808,000	26,600	268,000	119,000	23,700	61,900	161,000	6,870	1,020,000	15,600	315

Counts
- **<2.5k**
- **2.5k − <5k**
- **5k − <10k**
- **10k − <25k**
- **25k − <75k**
- **75k − <150k**
- **>=150k**

Kenya

DALYs (count) *attributable to* bacterial antimicrobial resistance by pathogen−drug combinations, 2019

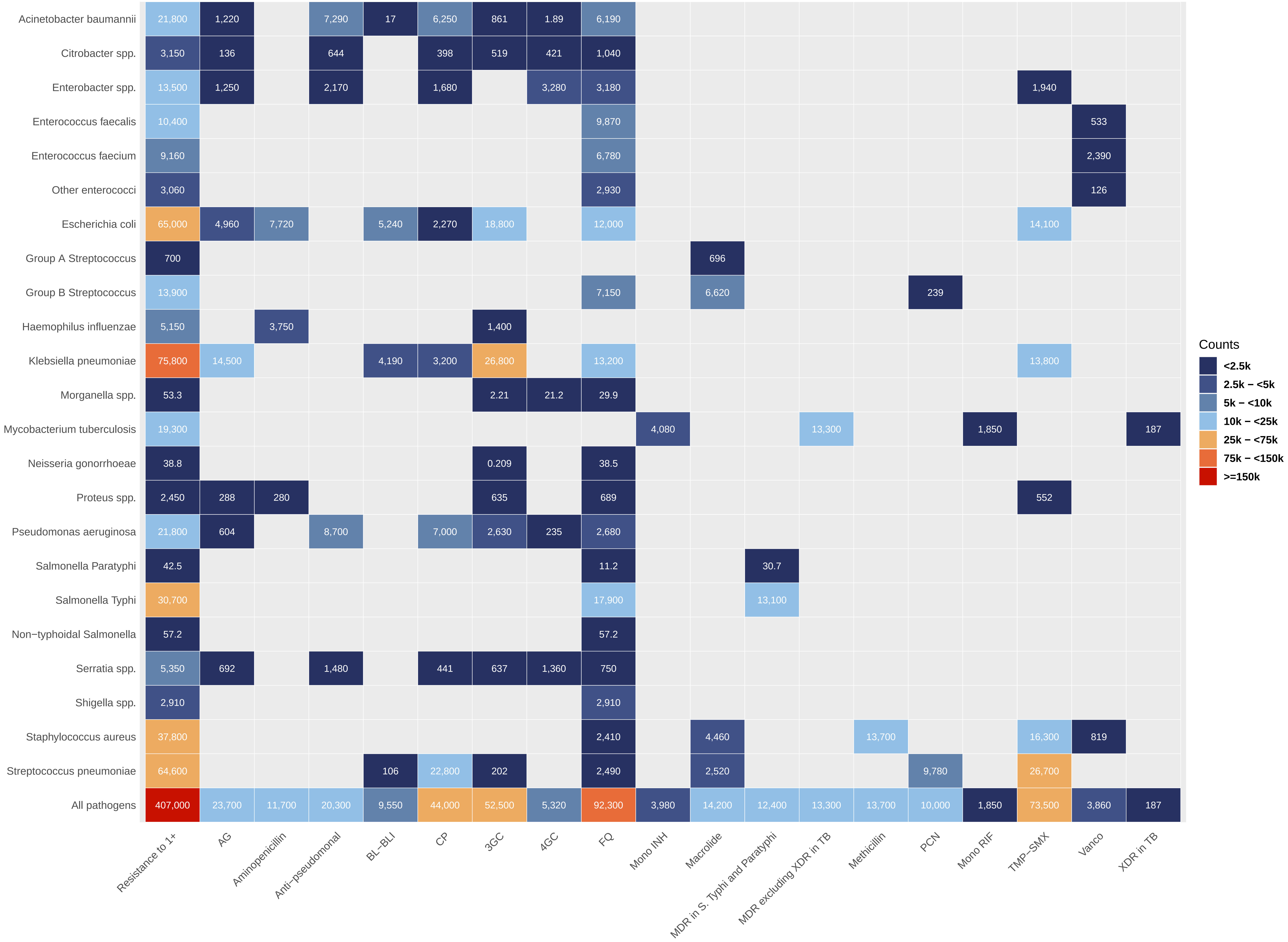

Lesotho

DALYs (count) *associated with* bacterial antimicrobial resistance by pathogen−drug combinations, 2019

	Resistance to 1+	AG	Aminopenicillin	Anti−pseudomonal	BL−BLI	CP	3GC	4GC	FQ	Mono INH	Macrolide	MDR in S. Typhi and Paratyphi	MDR excluding XDR in TB	Methicillin	PCN	Mono RIF	TMP−SMX	Vanco	XDR in TB
Acinetobacter baumannii	6,880	2,020		6,480	3,950	3,710	6,090	5,550	4,380										
Citrobacter spp.	272	53.8		140		60.7	186	117	97.9										
Enterobacter spp.	2,310	451		627		424		1,010	850								1,950		
Enterococcus faecalis	1,770								1,730									126	
Enterococcus faecium	2,110								2,100									458	
Other enterococci	735								731									82.1	
Escherichia coli	15,000	2,650	14,200		10,100	786	5,720		3,250								12,000		
Group A Streptococcus	280										280								
Group B Streptococcus	4,840								1,980		4,010				109				
Haemophilus influenzae	1,570		1,500				237												
Klebsiella pneumoniae	22,300	8,370			18,000	316	20,800		12,600								18,300		
Morganella spp.	13.7						2.13	7.44	10.4										
Mycobacterium tuberculosis	18,100									4,480			10,700			2,720			145
Neisseria gonorrhoeae	34.6						0.078		34.5										
Proteus spp.	1,000	60	913				245		124								707		
Pseudomonas aeruginosa	6,830	2,030		4,480		3,770	3,720	3,040	3,450										
Salmonella Paratyphi	1.21								1.2			0.016							
Salmonella Typhi	4,040								1,780			2,680							
Non−typhoidal Salmonella	11.5								11.5										
Serratia spp.	877	566		190		200	312	346	184										
Shigella spp.	562								562										
Staphylococcus aureus	18,400								5,570		13,200			4,870			9,010	580	
Streptococcus pneumoniae	21,900				8,000	5,530	2,850		3,490		8,120				8,700		19,800		
All pathogens	130,000	16,200	16,600	11,900	40,100	14,800	40,100	10,100	42,900	4,480	25,600	2,680	10,700	4,870	8,800	2,720	61,700	1,250	145

Counts
- <2.5k
- 2.5k − <5k
- 5k − <10k
- 10k − <25k
- 25k − <75k
- 75k − <150k

Lesotho

DALYs (count) *attributable to* bacterial antimicrobial resistance by pathogen−drug combinations, 2019

	Resistance to 1+	AG	Aminopenicillin	Anti−pseudomonal	BL−BLI	CP	3GC	4GC	FQ	Mono INH	Macrolide	MDR in S. Typhi and Paratyphi	MDR excluding XDR in TB	Methicillin	PCN	Mono RIF	TMP−SMX	Vanco	XDR in TB
Acinetobacter baumannii	2,120	94.6		665	0.673	657	109	6.09	582										
Citrobacter spp.	79.2	3.09		27.4		15.2	10.1	8.87	14.6										
Enterobacter spp.	465	29.9		57.4		94.9		60.9	94								128		
Enterococcus faecalis	467								432									35.1	
Enterococcus faecium	518								411									107	
Other enterococci	155								140									15.2	
Escherichia coli	3,320	174	345		605	189	855		319								835		
Group A Streptococcus	26.6										26.3								
Group B Streptococcus	737								333		363				22.6				
Haemophilus influenzae	315		247				67.6												
Klebsiella pneumoniae	6,070	615			112	129	3,450		842								918		
Morganella spp.	3.09						0.084	1.25	1.76										
Mycobacterium tuberculosis	7,740									689			6,220			740			87.1
Neisseria gonorrhoeae	3.39						0.042		3.35										
Proteus spp.	123	3.96	19.4				57.9		10.5								31.5		
Pseudomonas aeruginosa	1,710	54.2		466		685	97.8	38	372										
Salmonella Paratyphi	0.249								0.247			0.002							
Salmonella Typhi	672								337			338							
Non−typhoidal Salmonella	2.23								2.23										
Serratia spp.	180	34.2		31.7		52.3	12.8	29.2	19										
Shigella spp.	116								116										
Staphylococcus aureus	3,090								229		536			1,180			973	168	
Streptococcus pneumoniae	4,440				311	1,140	105		407		287				450		1,740		
All pathogens	32,300	1,010	612	1,250	1,030	2,970	4,770	144	4,660	682	1,230	323	6,220	1,180	472	740	4,620	326	87.1

Counts
- **<2.5k**
- **2.5k − <5k**
- **5k − <10k**
- **25k − <75k**

Liberia

DALYs (count) *associated with* bacterial antimicrobial resistance by pathogen−drug combinations, 2019

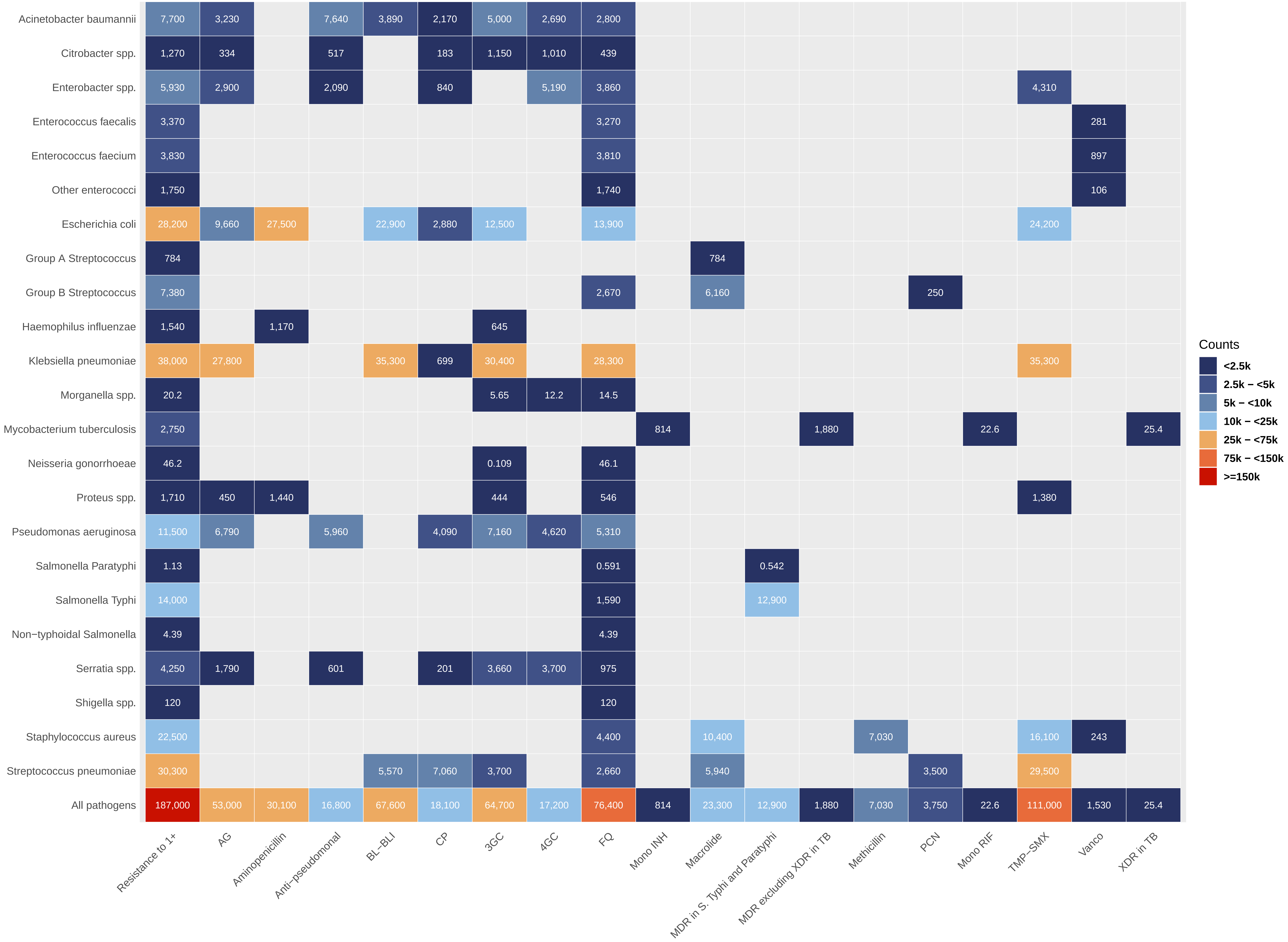

Liberia

DALYs (count) *attributable to* bacterial antimicrobial resistance by pathogen−drug combinations, 2019

	Resistance to 1+	AG	Aminopenicillin	Anti−pseudomonal	BL−BLI	CP	3GC	4GC	FQ	Mono INH	Macrolide	MDR in S. Typhi and Paratyphi	MDR excluding XDR in TB	Methicillin	PCN	Mono RIF	TMP−SMX	Vanco	XDR in TB
Acinetobacter baumannii	2,180	172		1,260	0.056	383	1.52	0.172	362										
Citrobacter spp.	376	18		95.2		41.8	49.2	117	54.7										
Enterobacter spp.	1,430	150		165		165		396	370								187		
Enterococcus faecalis	898								816									82.5	
Enterococcus faecium	953								741									212	
Other enterococci	360								339									21.4	
Escherichia coli	6,820	575	354		1,040	592	1,510		1,310								1,440		
Group A Streptococcus	73.4										76.3								
Group B Streptococcus	1,100								452		584				47.6				
Haemophilus influenzae	355		168				187												
Klebsiella pneumoniae	9,850	1,930			564	259	3,860		1,700								1,540		
Morganella spp.	4.74						0.288	2.1	2.35										
Mycobacterium tuberculosis	1,200									125			1,060			5.95			15
Neisseria gonorrhoeae	4.57						0.052		4.51										
Proteus spp.	233	21.2	26.3				92		38.2								54.2		
Pseudomonas aeruginosa	2,710	199		718		694	421	105	574										
Salmonella Paratyphi	0.199								0.125			0.075							
Salmonella Typhi	1,960								290			1,650							
Non−typhoidal Salmonella	0.891								0.891										
Serratia spp.	1,140	104		130		58.8	123	625	98.3										
Shigella spp.	24.6								24.6										
Staphylococcus aureus	4,140								179		405			1,610			1,880	74	
Streptococcus pneumoniae	5,450				158	1,560	127		316		224				66.8		3,000		
All pathogens	41,300	3,170	549	2,370	1,770	3,750	6,360	1,250	7,670	108	1,300	1,640	1,060	1,610	114	5.95	8,100	390	15

Counts
- <2.5k
- 2.5k – <5k
- 5k – <10k
- 25k – <75k

Madagascar

DALYs (count) *associated with* bacterial antimicrobial resistance by pathogen−drug combinations, 2019

	Resistance to 1+	AG	Aminopenicillin	Anti−pseudomonal	BL−BLI	CP	3GC	4GC	FQ	Mono INH	Macrolide	MDR in S. Typhi and Paratyphi	MDR excluding XDR in TB	Methicillin	PCN	Mono RIF	TMP−SMX	Vanco	XDR in TB
Acinetobacter baumannii	56,900	24,900		21,800	55,400	15,800	51,700	30,600	31,300										
Citrobacter spp.	6,330	2,070		2,790		214	5,200	4,480	4,640										
Enterobacter spp.	36,200	17,000		8,650		3,310		31,400	26,300								29,500		
Enterococcus faecalis	24,800								24,600									1,230	
Enterococcus faecium	15,200								15,000									4,380	
Other enterococci	6,380								6,350									443	
Escherichia coli	207,000	62,000	196,000		150,000	11,700	75,300		116,000								167,000		
Group A Streptococcus	9,540										9,540								
Group B Streptococcus	89,400								29,100		83,000				1,960				
Haemophilus influenzae	35,000		34,800				4,380												
Klebsiella pneumoniae	233,000	147,000			228,000	1,160	173,000		136,000								167,000		
Morganella spp.	113						27.8	71.1	80.3										
Mycobacterium tuberculosis	38,900									5,630			31,800			980			423
Neisseria gonorrhoeae	138						8.93		135										
Proteus spp.	9,630	2,140	7,590				1,030		3,780								9,140		
Pseudomonas aeruginosa	82,000	18,300		8,580		5,380	68,500	20,000	51,700										
Salmonella Paratyphi	0.979								0.068			0.838							
Salmonella Typhi	45,500								21,800			26,100							
Non−typhoidal Salmonella	352								352										
Serratia spp.	21,000	11,200		10,600		735	17,900	15,700	6,140										
Shigella spp.	940								940										
Staphylococcus aureus	167,000								66,700		77,200			52,600			121,000	1,930	
Streptococcus pneumoniae	272,000				52,400	57,500	29,000		193,000		8,350				63,400		243,000		
All pathogens	1,360,000	285,000	239,000	52,500	486,000	95,900	426,000	102,000	733,000	5,630	178,000	26,100	31,800	52,600	65,300	980	737,000	7,970	423

Counts
- <2.5k
- 2.5k − <5k
- 5k − <10k
- 10k − <25k
- 25k − <75k
- 75k − <150k
- >=150k

Madagascar

DALYs (count) *attributable to* bacterial antimicrobial resistance by pathogen−drug combinations, 2019

	Resistance to 1+	AG	Aminopenicillin	Anti−pseudomonal	BL−BLI	CP	3GC	4GC	FQ	Mono INH	Macrolide	MDR in S. Typhi and Paratyphi	MDR excluding XDR in TB	Methicillin	PCN	Mono RIF	TMP−SMX	Vanco	XDR in TB
Acinetobacter baumannii	16,500	1,150		1,260	875	2,480	5,440	1,370	3,930										
Citrobacter spp.	1,840	92.8		451		51.7	197	385	664										
Enterobacter spp.	8,590	823		647		652		2,700	2,530								1,230		
Enterococcus faecalis	6,450								6,130									320	
Enterococcus faecium	3,910								2,870									1,040	
Other enterococci	1,330								1,240									88.9	
Escherichia coli	48,100	3,630	3,630		7,930	2,490	8,990		11,400								10,000		
Group A Streptococcus	857										855								
Group B Streptococcus	12,800								4,520		7,580				391				
Haemophilus influenzae	6,850		5,640				1,210												
Klebsiella pneumoniae	58,600	10,800			7,160	735	24,100		8,580								7,180		
Morganella spp.	26.3						1.15	12.2	13										
Mycobacterium tuberculosis	19,200									848			17,900			264			252
Neisseria gonorrhoeae	14.7						1.81		12.9										
Proteus spp.	1,120	102	147				224		271								381		
Pseudomonas aeruginosa	21,100	450		1,410		1,010	10,500	1,830	5,990										
Salmonella Paratyphi	0.147								0.031			0.113							
Salmonella Typhi	7,680								4,330			3,400							
Non−typhoidal Salmonella	71.4								71.4										
Serratia spp.	5,560	647		2,110		231	553	1,410	609										
Shigella spp.	194								194										
Staphylococcus aureus	30,900								2,750		2,750			11,500			13,400	561	
Streptococcus pneumoniae	58,800				1,370	10,300	812		24,700		343				2,290		18,900		
All pathogens	311,000	17,700	9,420	5,880	17,300	18,000	52,000	7,700	80,800	820	11,800	3,250	17,900	11,500	2,680	264	51,100	2,010	252

Counts
- <2.5k
- 2.5k − <5k
- 5k − <10k
- 10k − <25k
- 25k − <75k
- 75k − <150k
- >=150k

Malawi

DALYs (count) *associated with* bacterial antimicrobial resistance by pathogen−drug combinations, 2019

	Resistance to 1+	AG	Aminopenicillin	Anti−pseudomonal	BL−BLI	CP	3GC	4GC	FQ	Mono INH	Macrolide	MDR in S. Typhi and Paratyphi	MDR excluding XDR in TB	Methicillin	PCN	Mono RIF	TMP−SMX	Vanco	XDR in TB
Acinetobacter baumannii	34,000	11,400		28,200	11,400	7,810	33,200	11,900	17,200										
Citrobacter spp.	4,870	1,290		2,040		258	4,850	2,950	993										
Enterobacter spp.	23,600	13,400		9,320		2,750		21,000	15,200								17,100		
Enterococcus faecalis	18,000								17,800									940	
Enterococcus faecium	12,900								12,700									3,500	
Other enterococci	5,740								5,720									314	
Escherichia coli	130,000	33,400	123,000		114,000	18,100	47,000		44,000								120,000		
Group A Streptococcus	6,850										6,850								
Group B Streptococcus	32,800								17,500		22,900				722				
Haemophilus influenzae	19,000		18,700				2,590												
Klebsiella pneumoniae	169,000	123,000			146,000	1,880	146,000		78,500								156,000		
Morganella spp.	87.8						21.4	53.5	63.8										
Mycobacterium tuberculosis	21,900									4,750			14,200			2,700			190
Neisseria gonorrhoeae	24.9						3.14		22.8										
Proteus spp.	6,850	3,610	6,030				2,410		4,310								4,750		
Pseudomonas aeruginosa	44,000	15,500		16,400		12,400	34,700	15,000	20,800										
Salmonella Paratyphi	0.629								0.12			0.501							
Salmonella Typhi	69,500								15,700			67,300							
Non−typhoidal Salmonella	26.2								26.2										
Serratia spp.	11,300	4,900		4,290		672	2,270	8,990	2,290										
Shigella spp.	1,020								1,020										
Staphylococcus aureus	107,000								25,300		55,000			34,900			73,200	1,350	
Streptococcus pneumoniae	186,000				35,600	41,400	12,200		27,300		18,700				57,900		184,000		
All pathogens	904,000	206,000	147,000	60,200	307,000	85,300	285,000	59,900	307,000	4,750	104,000	67,300	14,200	34,900	58,600	2,700	555,000	6,110	190

Counts
- <2.5k
- 2.5k − <5k
- 5k − <10k
- 10k − <25k
- 25k − <75k
- 75k − <150k
- >=150k

Malawi

DALYs (count) *attributable to* bacterial antimicrobial resistance by pathogen−drug combinations, 2019

	Resistance to 1+	AG	Aminopenicillin	Anti−pseudomonal	BL−BLI	CP	3GC	4GC	FQ	Mono INH	Macrolide	MDR in S. Typhi and Paratyphi	MDR excluding XDR in TB	Methicillin	PCN	Mono RIF	TMP−SMX	Vanco	XDR in TB
Acinetobacter baumannii	10,200	557		4,190	0.511	1,340	1,840	1.26	2,290										
Citrobacter spp.	1,450	74.6		434		63.5	408	347	119										
Enterobacter spp.	5,670	710		774		543		1,530	1,400								730		
Enterococcus faecalis	4,690								4,450									247	
Enterococcus faecium	3,280								2,440									834	
Other enterococci	1,190								1,120									64.7	
Escherichia coli	30,500	2,100	883		5,620	4,030	5,540		4,200								8,080		
Group A Streptococcus	653										640								
Group B Streptococcus	5,400								3,090		2,080				165				
Haemophilus influenzae	3,760		3,040				712												
Klebsiella pneumoniae	44,500	9,130			1,450	838	20,800		4,770								7,500		
Morganella spp.	20.5						0.939	9.12	10.4										
Mycobacterium tuberculosis	9,740									715			8,180			730			114
Neisseria gonorrhoeae	2.89						0.717		2.17										
Proteus spp.	1,110	161	88.6				436		278								149		
Pseudomonas aeruginosa	11,200	470		2,080		2,130	3,620	582	2,390										
Salmonella Paratyphi	0.097								0.029			0.067							
Salmonella Typhi	10,400								2,310			8,340							
Non−typhoidal Salmonella	5.42								5.42										
Serratia spp.	2,370	259		749		211	2.64	926	217										
Shigella spp.	209								209										
Staphylococcus aureus	19,800								1,030		2,120			7,920			8,350	384	
Streptococcus pneumoniae	34,800				1,220	8,890	162		2,990		683				3,060		17,800		
All pathogens	201,000	13,500	4,020	8,230	8,300	18,000	33,600	3,390	33,300	694	5,600	8,000	8,180	7,920	3,220	730	42,600	1,530	114

Counts
- <2.5k
- 2.5k − <5k
- 5k − <10k
- 10k − <25k
- 25k − <75k
- >=150k

Mali

DALYs (count) *associated with* bacterial antimicrobial resistance by pathogen−drug combinations, 2019

	Resistance to 1+	AG	Aminopenicillin	Anti−pseudomonal	BL−BLI	CP	3GC	4GC	FQ	Mono INH	Macrolide	MDR in S. Typhi and Paratyphi	MDR excluding XDR in TB	Methicillin	PCN	Mono RIF	TMP−SMX	Vanco	XDR in TB
Acinetobacter baumannii	70,300	31,100		69,700	38,600	26,900	44,900	44,000	34,600										
Citrobacter spp.	16,800	7,650		7,760		6,990	15,000	14,900	7,100										
Enterobacter spp.	69,100	31,900		27,400		14,600		59,200	44,400								56,500		
Enterococcus faecalis	37,200								36,700									2,520	
Enterococcus faecium	28,200								28,000									6,640	
Other enterococci	21,900								21,900									992	
Escherichia coli	306,000	104,000	291,000		231,000	57,100	149,000		194,000								292,000		
Group A Streptococcus	7,810										7,810								
Group B Streptococcus	152,000								109,000		92,600				3,130				
Haemophilus influenzae	29,800		19,300				17,000												
Klebsiella pneumoniae	484,000	349,000			451,000	16,300	433,000		362,000								461,000		
Morganella spp.	144						36.5	87.2	108										
Mycobacterium tuberculosis	22,400									6,760			15,200			215			204
Neisseria gonorrhoeae	215						0.422		214										
Proteus spp.	13,900	4,880	12,100				3,510		7,600								11,000		
Pseudomonas aeruginosa	113,000	55,000		63,700		43,200	63,700	56,700	65,400										
Salmonella Paratyphi	22.6								11.5			11.4							
Salmonella Typhi	145,000								26,400			130,000							
Non−typhoidal Salmonella	148								148										
Serratia spp.	46,000	25,800		6,460		2,250	36,400	39,700	13,300										
Shigella spp.	2,260								2,260										
Staphylococcus aureus	243,000								43,900		114,000			122,000			161,000	8,850	
Streptococcus pneumoniae	336,000				84,200	117,000	52,300		39,000		123,000				40,600		271,000		
All pathogens	2,150,000	608,000	323,000	175,000	805,000	285,000	816,000	214,000	1,040,000	6,760	337,000	130,000	15,200	122,000	43,700	215	1,250,000	19,000	204

Counts
- <2.5k
- 2.5k − <5k
- 5k − <10k
- 10k − <25k
- 25k − <75k
- 75k − <150k
- >=150k

Mali

DALYs (count) *attributable to* bacterial antimicrobial resistance by pathogen−drug combinations, 2019

	Resistance to 1+	AG	Aminopenicillin	Anti−pseudomonal	BL−BLI	CP	3GC	4GC	FQ	Mono INH	Macrolide	MDR in S. Typhi and Paratyphi	MDR excluding XDR in TB	Methicillin	PCN	Mono RIF	TMP−SMX	Vanco	XDR in TB
Acinetobacter baumannii	20,400	1,560		9,650	1.06	4,690	21.4	1.57	4,510										
Citrobacter spp.	5,140	417		926		1,520	317	1,130	843										
Enterobacter spp.	16,900	1,640		1,980		2,800		3,790	4,210								2,570		
Enterococcus faecalis	9,820								9,150									668	
Enterococcus faecium	7,000								5,440									1,560	
Other enterococci	4,470								4,270									199	
Escherichia coli	78,700	5,910	3,460		7,580	11,100	15,100		18,400								17,100		
Group A Streptococcus	763										724								
Group B Streptococcus	27,600								19,200		7,590				616				
Haemophilus influenzae	7,380		2,470				4,920												
Klebsiella pneumoniae	131,000	24,200			3,770	4,720	55,900		21,900								20,300		
Morganella spp.	33.7						1.59	14.5	17.6										
Mycobacterium tuberculosis	9,890									1,050			8,700			58.3			122
Neisseria gonorrhoeae	21						0.203		20.7										
Proteus spp.	1,990	207	213				683		498								407		
Pseudomonas aeruginosa	27,300	1,490		7,570		7,130	2,300	1,520	7,260										
Salmonella Paratyphi	3.92								2.39			1.56							
Salmonella Typhi	20,700								4,630			16,300							
Non−typhoidal Salmonella	31.4								31.4										
Serratia spp.	11,900	1,420		1,320		644	1,050	6,180	1,290										
Shigella spp.	464								464										
Staphylococcus aureus	54,900								1,830		4,200			29,200			17,500	2,240	
Streptococcus pneumoniae	66,600				2,220	26,500	1,130		5,260		4,960				358		26,100		
All pathogens	503,000	36,900	6,130	21,400	13,500	59,100	81,400	12,600	109,000	957	17,800	16,100	8,700	29,200	974	58.3	84,000	4,680	122

Counts
- <2.5k
- 2.5k − <5k
- 5k − <10k
- 10k − <25k
- 25k − <75k
- 75k − <150k
- >=150k

Mauritania

DALYs (count) *associated with* bacterial antimicrobial resistance by pathogen−drug combinations, 2019

	Resistance to 1+	AG	Aminopenicillin	Anti−pseudomonal	BL−BLI	CP	3GC	4GC	FQ	Mono INH	Macrolide	MDR in S. Typhi and Paratyphi	MDR excluding XDR in TB	Methicillin	PCN	Mono RIF	TMP−SMX	Vanco	XDR in TB
Acinetobacter baumannii	5,470	2,980		5,440	3,070	2,580	3,390	2,970	3,110										
Citrobacter spp.	941	296		329		152	830	776	363										
Enterobacter spp.	5,310	1,970		2,040		986		4,030	3,250								4,330		
Enterococcus faecalis	2,340								2,290									175	
Enterococcus faecium	2,600								2,580									626	
Other enterococci	1,550								1,550									96.2	
Escherichia coli	19,500	8,940	18,900		14,900	1,860	8,300		7,690								17,300		
Group A Streptococcus	434										434								
Group B Streptococcus	7,030								3,310		5,390				203				
Haemophilus influenzae	934		647				447												
Klebsiella pneumoniae	23,700	13,500			21,900	1,010	18,900		16,300								22,000		
Morganella spp.	13.8						3.37	3.84	12.3										
Mycobacterium tuberculosis	1,310									417			866			11.5			11.5
Neisseria gonorrhoeae	40.6						0.078		40.5										
Proteus spp.	1,120	636	916				286		595								897		
Pseudomonas aeruginosa	8,460	4,550		4,140		3,510	4,520	2,930	5,380										
Salmonella Paratyphi	2.33								1.96			0.386							
Salmonella Typhi	5,310								1,290			4,380							
Non−typhoidal Salmonella	3.99								3.99										
Serratia spp.	2,510	1,230		388		189	1,950	1,980	755										
Shigella spp.	506								506										
Staphylococcus aureus	15,700								3,220		9,060			9,150			8,380	616	
Streptococcus pneumoniae	20,400				3,550	6,060	2,650		1,440		6,020				2,770		19,600		
All pathogens	125,000	34,100	20,400	12,300	43,400	16,300	41,300	12,700	53,700	417	20,900	4,380	866	9,150	2,970	11.5	72,500	1,510	11.5

Counts
- <2.5k
- 2.5k − <5k
- 5k − <10k
- 10k − <25k
- 25k − <75k
- 75k − <150k

Mauritania

DALYs (count) *attributable to* bacterial antimicrobial resistance by pathogen−drug combinations, 2019

	Resistance to 1+	AG	Aminopenicillin	Anti−pseudomonal	BL−BLI	CP	3GC	4GC	FQ	Mono INH	Macrolide	MDR in S. Typhi and Paratyphi	MDR excluding XDR in TB	Methicillin	PCN	Mono RIF	TMP−SMX	Vanco	XDR in TB
Acinetobacter baumannii	1,600	143		635	0.042	428	0.279	0.12	391										
Citrobacter spp.	278	15.5		56.8		33.8	32.4	93.5	45.4										
Enterobacter spp.	1,250	104		160		193		256	327								215		
Enterococcus faecalis	622								572									49.5	
Enterococcus faecium	648								500									147	
Other enterococci	317								299									18.3	
Escherichia coli	4,600	552	297		652	383	989		689								1,040		
Group A Streptococcus	40.7										41.7								
Group B Streptococcus	1,120								572		522				39.3				
Haemophilus influenzae	222		91.8				130												
Klebsiella pneumoniae	6,160	953			370	283	2,500		1,020								1,040		
Morganella spp.	3.13						0.324	0.629	2.18										
Mycobacterium tuberculosis	553									64.1			482			3.06			6.74
Neisseria gonorrhoeae	3.98						0.039		3.94										
Proteus spp.	164	29.6	15				52.7		36.9								30.3		
Pseudomonas aeruginosa	1,970	123		406		557	227	49.7	605										
Salmonella Paratyphi	0.461								0.409			0.051							
Salmonella Typhi	803								244			550							
Non−typhoidal Salmonella	0.769								0.769										
Serratia spp.	649	67.5		78.1		51	88.7	289	73.7										
Shigella spp.	103								103										
Staphylococcus aureus	3,740								137		341			2,250			859	155	
Streptococcus pneumoniae	3,820				78.2	1,300	51.3		171		219				45.4		1,950		
All pathogens	28,700	1,990	403	1,340	1,100	3,230	4,070	689	5,800	58.3	1,110	543	482	2,250	84.7	3.06	5,120	370	6.74

Counts
- <2.5k
- 2.5k − <5k
- 5k − <10k
- 25k − <75k

Mauritius

DALYs (count) *associated with* bacterial antimicrobial resistance by pathogen−drug combinations, 2019

	Resistance to 1+	AG	Aminopenicillin	Anti−pseudomonal	BL−BLI	CP	3GC	4GC	FQ	Mono INH	Macrolide	MDR in S. Typhi and Paratyphi	MDR excluding XDR in TB	Methicillin	PCN	Mono RIF	TMP−SMX	Vanco	XDR in TB
Acinetobacter baumannii	1,860	1,430		1,790	1,440	1,460	1,680	1,770	1,560										
Citrobacter spp.	123	12.8		15.8		22	76.1	73.3	52.2										
Enterobacter spp.	660	101		327		177		273	169								413		
Enterococcus faecalis	514								489									65.7	
Enterococcus faecium	1,110								1,110									261	
Other enterococci	451								448									158	
Escherichia coli	3,380	436	3,230		2,550	455	1,620		1,990								2,300		
Group A Streptococcus	187										187								
Group B Streptococcus	398								108		333				35.6				
Haemophilus influenzae	88.9		81				19.7												
Klebsiella pneumoniae	2,510	317			1,800	470	1,940		1,260								1,890		
Morganella spp.	7.76						4.61	1.72	4.61										
Mycobacterium tuberculosis	25.9									18.4			5.79			0.652			1.01
Neisseria gonorrhoeae	10.6						0.082		10.6										
Proteus spp.	402	294	320				153		159								228		
Pseudomonas aeruginosa	1,400	412		530		990	718	534	620										
Salmonella Paratyphi	26.3								25.8			0.548							
Salmonella Typhi	36.1								33.2			3.16							
Non−typhoidal Salmonella	0.5								0.5										
Serratia spp.	132	19.4		43.6		30.2	94.3	39.2	40.3										
Shigella spp.	3.11								3.11										
Staphylococcus aureus	3,980								1,100		2,100			3,650			1,050	64.1	
Streptococcus pneumoniae	1,910				323	990	378		84.3		1,160				667		1,390		
All pathogens	19,200	3,030	3,630	2,700	6,110	4,590	6,680	2,690	9,270	18.4	3,770	3.71	5.79	3,650	702	0.652	7,280	548	1.01

Counts
- **<2.5k**
- **2.5k – <5k**
- **5k – <10k**
- **10k – <25k**

Mauritius

DALYs (count) *attributable to* bacterial antimicrobial resistance by pathogen−drug combinations, 2019

	Resistance to 1+	AG	Aminopenicillin	Anti−pseudomonal	BL−BLI	CP	3GC	4GC	FQ	Mono INH	Macrolide	MDR in S. Typhi and Paratyphi	MDR excluding XDR in TB	Methicillin	PCN	Mono RIF	TMP−SMX	Vanco	XDR in TB
Acinetobacter baumannii	587	62.9		73.8	0.307	238	9.99	7.57	194										
Citrobacter spp.	34.4	0.669		3.09		5.26	7.57	9.75	8.12										
Enterobacter spp.	146	6.76		37.2		45.6		11.2	18								27		
Enterococcus faecalis	140								120									19.8	
Enterococcus faecium	277								216									61.3	
Other enterococci	103								76.8									26.5	
Escherichia coli	851	26.4	48.3		123	101	206		211								135		
Group A Streptococcus	18.2										17								
Group B Streptococcus	58.2								18.6		32.6				6.51				
Haemophilus influenzae	18.6		12.9				5.65												
Klebsiella pneumoniae	682	32.4			34.4	122	283		98.3								112		
Morganella spp.	1.98						0.822	0.381	0.78										
Mycobacterium tuberculosis	5.76									2.33			2.73			0.157			0.539
Neisseria gonorrhoeae	1.05						0.022		1.02										
Proteus spp.	64.8	15.9	4.25				28		9.37								7.14		
Pseudomonas aeruginosa	345	11.4		28.1		188	39.8	12.4	65.5										
Salmonella Paratyphi	5.38								5.3			0.085							
Salmonella Typhi	7.3								6.88			0.416							
Non−typhoidal Salmonella	0.089								0.089										
Serratia spp.	35.1	1.12		9.13		8.31	8.46	3.33	4.69										
Shigella spp.	0.511								0.511										
Staphylococcus aureus	1,220								49.4		72.2			992			87.9	14.1	
Streptococcus pneumoniae	423				0.983	226	1.97		9.28		44.8				18.5		120		
All pathogens	5,020	157	65.7	151	159	935	592	44.6	1,110	2.37	170	0.508	2.73	992	25.1	0.157	489	122	0.539

Counts
- **<2.5k**
- **5k − <10k**

Mozambique

DALYs (count) *associated with* bacterial antimicrobial resistance by pathogen−drug combinations, 2019

	Resistance to 1+	AG	Aminopenicillin	Anti−pseudomonal	BL−BLI	CP	3GC	4GC	FQ	Mono INH	Macrolide	MDR in S. Typhi and Paratyphi	MDR excluding XDR in TB	Methicillin	PCN	Mono RIF	TMP−SMX	Vanco	XDR in TB
Acinetobacter baumannii	69,200	20,400		59,300	44,300	11,300	56,200	27,300	24,600										
Citrobacter spp.	9,600	3,130		4,530		625	8,380	6,830	5,490										
Enterobacter spp.	45,700	22,100		16,600		5,490		41,600	27,900								34,600		
Enterococcus faecalis	35,100								34,600									2,640	
Enterococcus faecium	24,000								23,700									6,140	
Other enterococci	11,100								11,000									689	
Escherichia coli	213,000	60,100	196,000		145,000	15,300	71,900		76,000								168,000		
Group A Streptococcus	16,000										16,000								
Group B Streptococcus	82,200								34,800		69,300				2,320				
Haemophilus influenzae	39,100		38,500				6,570												
Klebsiella pneumoniae	362,000	264,000			327,000	1,170	334,000		247,000								352,000		
Morganella spp.	191						44.5	120	139										
Mycobacterium tuberculosis	119,000									9,630			97,900			9,860			1,320
Neisseria gonorrhoeae	173						1.59		172										
Proteus spp.	13,800	3,850	11,500				2,740		5,430								12,300		
Pseudomonas aeruginosa	82,000	22,500		32,100		20,300	70,000	32,400	29,300										
Salmonella Paratyphi	3.01								0.125			2.68							
Salmonella Typhi	176,000								38,100			159,000							
Non−typhoidal Salmonella	1,160								1,160										
Serratia spp.	29,900	14,800		11,700		1,550	17,900	24,400	7,410										
Shigella spp.	2,600								2,600										
Staphylococcus aureus	235,000								64,000		39,100			101,000			201,000	2,850	
Streptococcus pneumoniae	337,000				63,000	72,500	41,300		160,000		34,300				64,900		313,000		
All pathogens	1,900,000	411,000	246,000	124,000	579,000	128,000	609,000	133,000	794,000	9,630	159,000	159,000	97,900	101,000	67,200	9,860	1,080,000	12,300	1,320

Counts
- <2.5k
- 2.5k − <5k
- 5k − <10k
- 10k − <25k
- 25k − <75k
- 75k − <150k
- >=150k

Mozambique

DALYs (count) *attributable to* bacterial antimicrobial resistance by pathogen−drug combinations, 2019

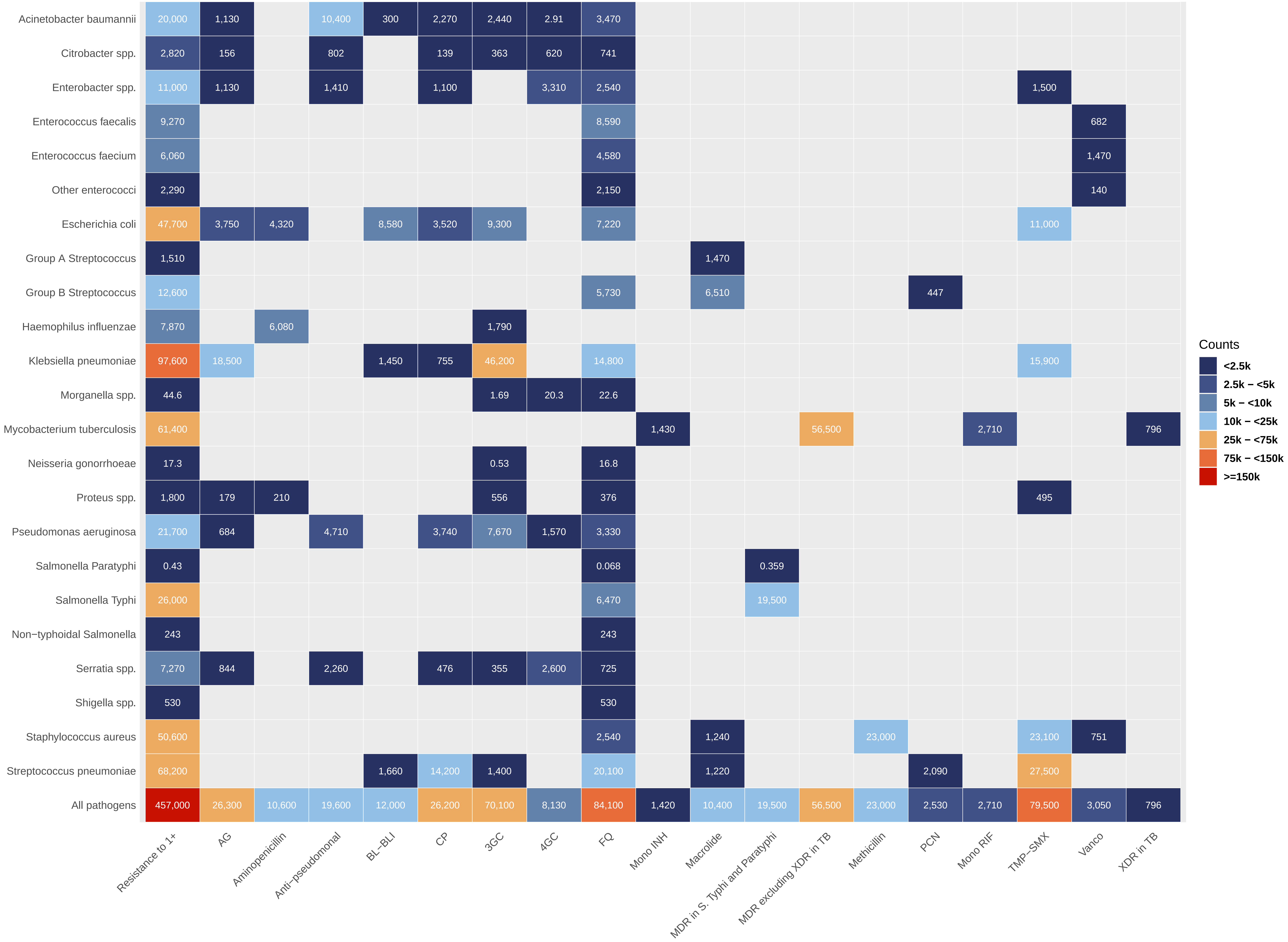

Namibia

DALYs (count) *associated with* bacterial antimicrobial resistance by pathogen−drug combinations, 2019

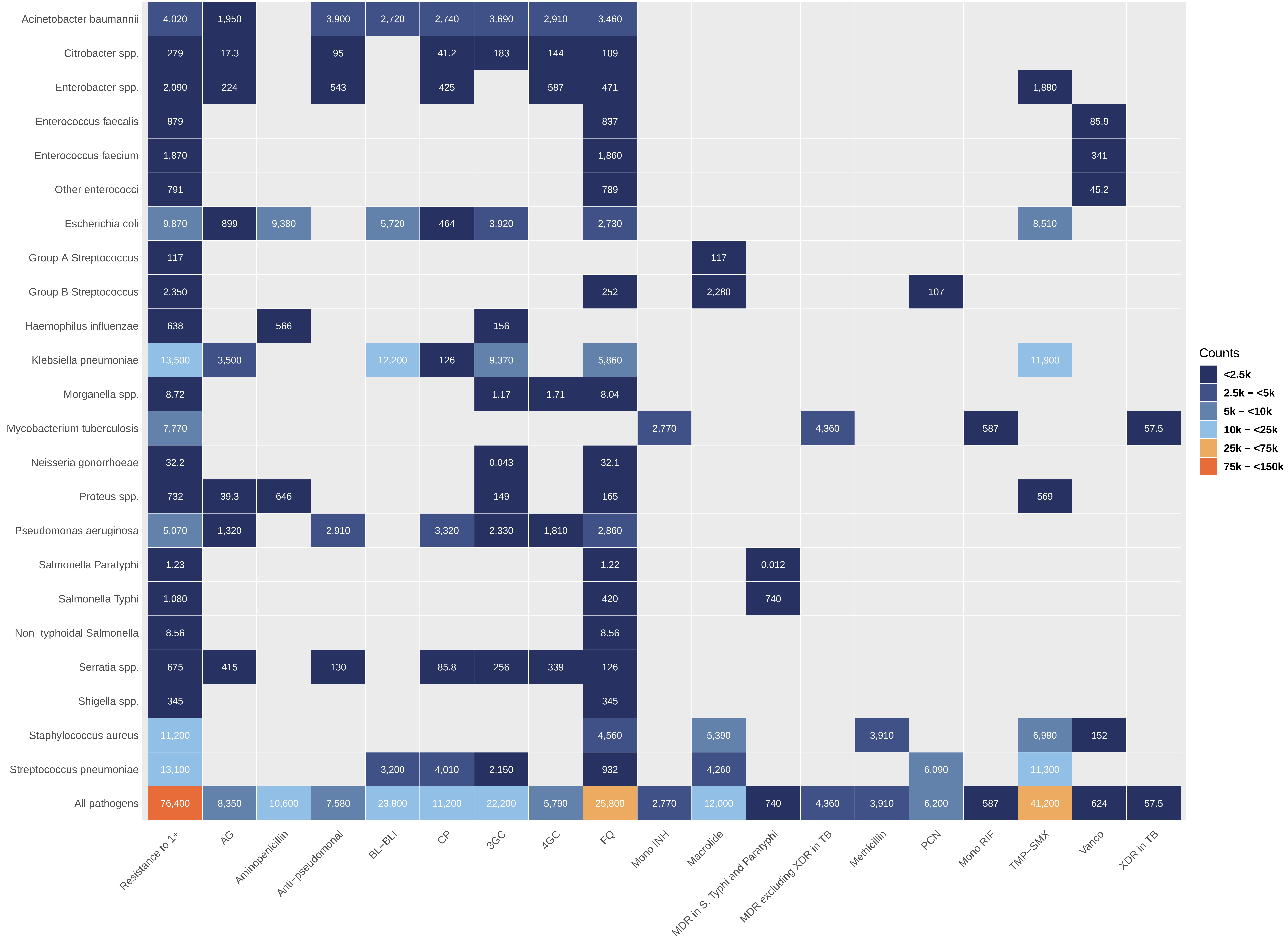

Namibia

DALYs (count) *attributable to* bacterial antimicrobial resistance by pathogen−drug combinations, 2019

	Resistance to 1+	AG	Aminopenicillin	Anti−pseudomonal	BL−BLI	CP	3GC	4GC	FQ	Mono INH	Macrolide	MDR in S. Typhi and Paratyphi	MDR excluding XDR in TB	Methicillin	PCN	Mono RIF	TMP−SMX	Vanco	XDR in TB
Acinetobacter baumannii	1,260	83.5		244	0.118	445	31	0.147	455										
Citrobacter spp.	78.8	0.939		19		10.2	15.1	15.9	17.6										
Enterobacter spp.	384	17.5		48.8		102		24	56.1								137		
Enterococcus faecalis	237								209									28	
Enterococcus faecium	451								370									81.4	
Other enterococci	162								153									9.04	
Escherichia coli	2,200	60.7	287		307	109	580		271								589		
Group A Streptococcus	11.4										11.2								
Group B Streptococcus	288								39.3		233				19.6				
Haemophilus influenzae	136		90.6				45.1												
Klebsiella pneumoniae	3,260	279			363	61.6	1,490		409								657		
Morganella spp.	1.9						0.12	0.279	1.5										
Mycobacterium tuberculosis	2,970									374			2,380			154			33.7
Neisseria gonorrhoeae	3.15						0.028		3.12										
Proteus spp.	87.9	2.55	13.9				34.3		12.8								24.6		
Pseudomonas aeruginosa	1,240	33.1		223		593	57.6	17.3	311										
Salmonella Paratyphi	0.251								0.249			0.002							
Salmonella Typhi	176								82.4			90.7							
Non−typhoidal Salmonella	1.56								1.56										
Serratia spp.	137	25.3		24.3		22.9	12.7	38.4	12.9										
Shigella spp.	70.5								70.5										
Staphylococcus aureus	2,120								198		203			894			776	44.2	
Streptococcus pneumoniae	2,700				85.7	866	61.8		107		154				382		1,050		
All pathogens	18,000	503	392	559	756	2,210	2,330	96.1	2,780	364	602	92.8	2,380	894	402	154	3,230	163	33.7

Counts
- <2.5k
- 2.5k − <5k
- 10k − <25k

Niger

DALYs (count) *associated with* bacterial antimicrobial resistance by pathogen−drug combinations, 2019

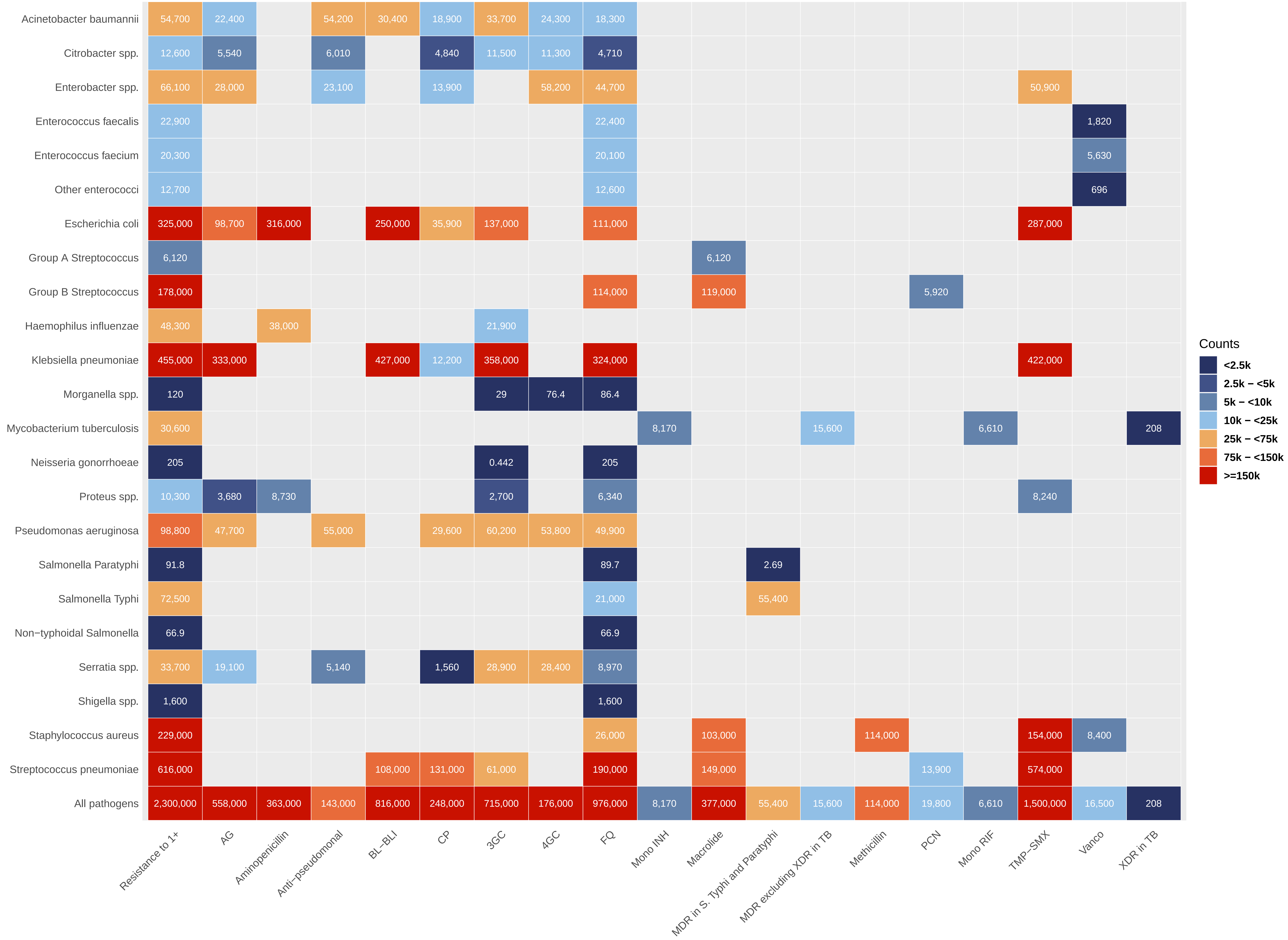

	Resistance to 1+	AG	Aminopenicillin	Anti−pseudomonal	BL−BLI	CP	3GC	4GC	FQ	Mono INH	Macrolide	MDR in S. Typhi and Paratyphi	MDR excluding XDR in TB	Methicillin	PCN	Mono RIF	TMP−SMX	Vanco	XDR in TB
Acinetobacter baumannii	54,700	22,400		54,200	30,400	18,900	33,700	24,300	18,300										
Citrobacter spp.	12,600	5,540		6,010		4,840	11,500	11,300	4,710										
Enterobacter spp.	66,100	28,000		23,100		13,900		58,200	44,700								50,900		
Enterococcus faecalis	22,900								22,400									1,820	
Enterococcus faecium	20,300								20,100									5,630	
Other enterococci	12,700								12,600									696	
Escherichia coli	325,000	98,700	316,000		250,000	35,900	137,000		111,000								287,000		
Group A Streptococcus	6,120										6,120								
Group B Streptococcus	178,000								114,000		119,000				5,920				
Haemophilus influenzae	48,300		38,000				21,900												
Klebsiella pneumoniae	455,000	333,000			427,000	12,200	358,000		324,000								422,000		
Morganella spp.	120						29	76.4	86.4										
Mycobacterium tuberculosis	30,600									8,170			15,600			6,610			208
Neisseria gonorrhoeae	205						0.442		205										
Proteus spp.	10,300	3,680	8,730				2,700		6,340								8,240		
Pseudomonas aeruginosa	98,800	47,700		55,000		29,600	60,200	53,800	49,900										
Salmonella Paratyphi	91.8								89.7			2.69							
Salmonella Typhi	72,500								21,000			55,400							
Non−typhoidal Salmonella	66.9								66.9										
Serratia spp.	33,700	19,100		5,140		1,560	28,900	28,400	8,970										
Shigella spp.	1,600								1,600										
Staphylococcus aureus	229,000								26,000		103,000			114,000			154,000	8,400	
Streptococcus pneumoniae	616,000				108,000	131,000	61,000		190,000		149,000				13,900		574,000		
All pathogens	2,300,000	558,000	363,000	143,000	816,000	248,000	715,000	176,000	976,000	8,170	377,000	55,400	15,600	114,000	19,800	6,610	1,500,000	16,500	208

Niger

DALYs (count) *attributable to* bacterial antimicrobial resistance by pathogen−drug combinations, 2019

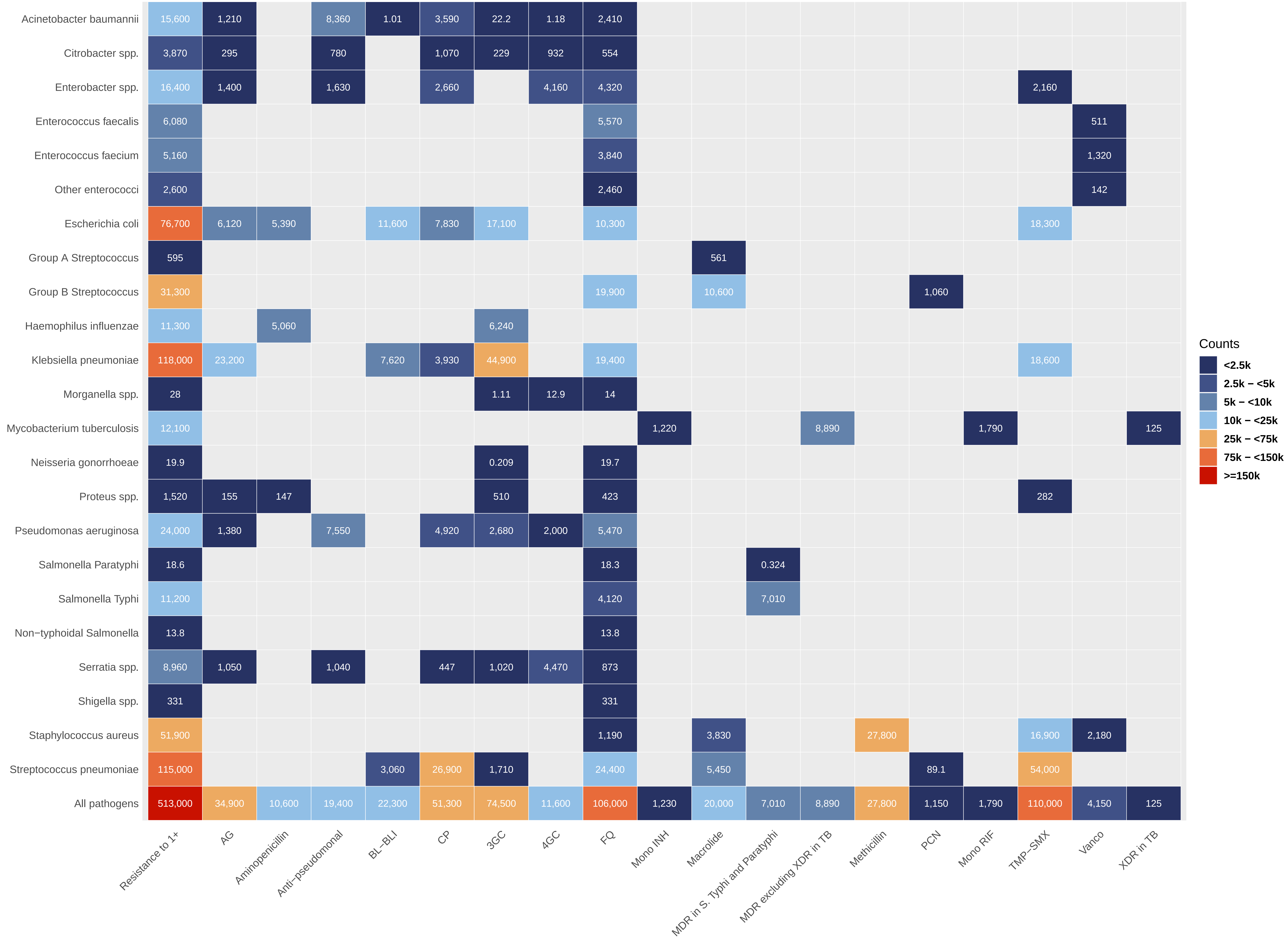

Nigeria

DALYs (count) *associated with* bacterial antimicrobial resistance by pathogen−drug combinations, 2019

	Resistance to 1+	AG	Aminopenicillin	Anti−pseudomonal	BL−BLI	CP	3GC	4GC	FQ	Mono INH	Macrolide	MDR in S. Typhi and Paratyphi	MDR excluding XDR in TB	Methicillin	PCN	Mono RIF	TMP−SMX	Vanco	XDR in TB
Acinetobacter baumannii	396,000	227,000		390,000	186,000	173,000	268,000	244,000	213,000										
Citrobacter spp.	105,000	36,700		43,600		16,000	95,400	84,400	41,000										
Enterobacter spp.	615,000	384,000		262,000		90,500		505,000	373,000								520,000		
Enterococcus faecalis	214,000								212,000									10,900	
Enterococcus faecium	186,000								185,000									45,700	
Other enterococci	128,000								128,000									5,340	
Escherichia coli	2,630,000	1,220,000	2,500,000		2,070,000	305,000	1,970,000		1,880,000								2,490,000		
Group A Streptococcus	43,300										43,300								
Group B Streptococcus	1,330,000								883,000		839,000				15,400				
Haemophilus influenzae	216,000		152,000				103,000												
Klebsiella pneumoniae	3,150,000	2,310,000			2,960,000	768,000	2,870,000		2,500,000								3,040,000		
Morganella spp.	897						214	501	693										
Mycobacterium tuberculosis	294,000									102,000			163,000			26,700			2,160
Neisseria gonorrhoeae	1,880						2.06		1,880										
Proteus spp.	83,800	20,500	74,300				21,500		32,800								65,700		
Pseudomonas aeruginosa	1,060,000	667,000		491,000		523,000	703,000	544,000	602,000										
Salmonella Paratyphi	4,820								4,070			925							
Salmonella Typhi	787,000								300,000			751,000							
Non−typhoidal Salmonella	31,700								31,700										
Serratia spp.	228,000	122,000		34,000		9,010	138,000	196,000	42,000										
Shigella spp.	640,000								640,000										
Staphylococcus aureus	2,140,000								328,000		1,320,000			1,400,000			1,570,000	26,800	
Streptococcus pneumoniae	4,240,000				698,000	849,000	725,000		476,000		1,110,000				2,250,000		3,890,000		
All pathogens	18,500,000	5,000,000	2,720,000	1,220,000	5,920,000	2,730,000	6,890,000	1,570,000	8,870,000	102,000	3,310,000	752,000	163,000	1,400,000	2,260,000	26,700	11,600,000	88,800	2,160

Counts
- <2.5k
- 2.5k − <5k
- 5k − <10k
- 10k − <25k
- 25k − <75k
- 75k − <150k
- >=150k

Nigeria

DALYs (count) *attributable to* bacterial antimicrobial resistance by pathogen−drug combinations, 2019

	Resistance to 1+	AG	Aminopenicillin	Anti−pseudomonal	BL−BLI	CP	3GC	4GC	FQ	Mono INH	Macrolide	MDR in S. Typhi and Paratyphi	MDR excluding XDR in TB	Methicillin	PCN	Mono RIF	TMP−SMX	Vanco	XDR in TB
Acinetobacter baumannii	117,000	11,300		47,500	2.96	29,700	792	9.33	27,300										
Citrobacter spp.	31,200	1,940		7,640		3,520	3,790	9,190	5,100										
Enterobacter spp.	146,000	21,300		20,300		17,300		30,200	33,500								21,900		
Enterococcus faecalis	56,000								53,100									2,960	
Enterococcus faecium	46,400								35,700									10,700	
Other enterococci	26,000								24,900									1,070	
Escherichia coli	711,000	67,600	13,600		37,600	54,400	233,000		172,000								134,000		
Group A Streptococcus	4,060										4,050								
Group B Streptococcus	234,000								157,000		70,500				4,280				
Haemophilus influenzae	51,300		21,500				29,800												
Klebsiella pneumoniae	924,000	165,000			20,100	155,000	295,000		156,000								134,000		
Morganella spp.	207						10.3	82.7	114										
Mycobacterium tuberculosis	115,000									15,200			91,700			7,220			1,280
Neisseria gonorrhoeae	183						1.28		181										
Proteus spp.	11,500	947	1,330				4,430		2,220								2,480		
Pseudomonas aeruginosa	258,000	18,100		37,900		86,200	36,100	16,000	63,500										
Salmonella Paratyphi	943								830			114							
Salmonella Typhi	128,000								43,300			86,800							
Non−typhoidal Salmonella	6,510								6,510										
Serratia spp.	54,800	7,140		7,150		2,940	3,430	30,100	4,110										
Shigella spp.	132,000								132,000										
Staphylococcus aureus	539,000								13,800		42,900			326,000			149,000	6,780	
Streptococcus pneumoniae	857,000				17,400	186,000	44,600		53,600		37,500				164,000		353,000		
All pathogens	4,450,000	293,000	36,500	121,000	75,100	536,000	650,000	85,600	984,000	15,400	159,000	85,600	91,700	326,000	168,000	7,220	796,000	21,500	1,280

Counts
- <2.5k
- 2.5k − <5k
- 5k − <10k
- 10k − <25k
- 25k − <75k
- 75k − <150k
- >=150k

Rwanda

DALYs (count) *associated with* bacterial antimicrobial resistance by pathogen−drug combinations, 2019

	Resistance to 1+	AG	Aminopenicillin	Anti−pseudomonal	BL−BLI	CP	3GC	4GC	FQ	Mono INH	Macrolide	MDR in S. Typhi and Paratyphi	MDR excluding XDR in TB	Methicillin	PCN	Mono RIF	TMP−SMX	Vanco	XDR in TB
Acinetobacter baumannii	19,900	6,530		19,100	12,200	12,300	18,300	12,900	12,700										
Citrobacter spp.	2,390	494		906		135	2,040	1,120	1,530										
Enterobacter spp.	14,000	7,930		6,310		1,000		13,300	8,020								9,800		
Enterococcus faecalis	9,340								9,240									473	
Enterococcus faecium	8,810								8,710									2,440	
Other enterococci	3,860								3,850									217	
Escherichia coli	68,300	18,100	65,000		51,500	3,470	39,300		31,900								57,900		
Group A Streptococcus	1,710										1,710								
Group B Streptococcus	19,700								8,860		16,100				158				
Haemophilus influenzae	5,240		4,930				925												
Klebsiella pneumoniae	94,100	69,900			92,900	4,300	89,300		59,800								84,000		
Morganella spp.	61.3						12.9	33.4	47.8										
Mycobacterium tuberculosis	13,500									1,610			10,900			814			145
Neisseria gonorrhoeae	152						0.881		152										
Proteus spp.	4,450	1,140	3,890				750		2,050								3,760		
Pseudomonas aeruginosa	33,200	19,800		8,420		13,000	26,400	8,050	12,400										
Salmonella Paratyphi	2.11								0.597			1.54							
Salmonella Typhi	34,000								14,600			27,200							
Non−typhoidal Salmonella	77.5								77.5										
Serratia spp.	5,510	2,820		1,510		300	3,700	2,480	1,750										
Shigella spp.	1,010								1,010										
Staphylococcus aureus	75,400								25,700		39,600			34,700			53,700	805	
Streptococcus pneumoniae	86,300				8,250	22,800	11,800		12,600		13,000				23,600		83,800		
All pathogens	501,000	127,000	73,800	36,200	165,000	57,300	193,000	37,900	215,000	1,610	70,300	27,200	10,900	34,700	23,700	814	293,000	3,930	145

Counts
- <2.5k
- 2.5k − <5k
- 5k − <10k
- 10k − <25k
- 25k − <75k
- 75k − <150k
- >=150k

Rwanda

DALYs (count) *attributable to* bacterial antimicrobial resistance by pathogen−drug combinations, 2019

	Resistance to 1+	AG	Aminopenicillin	Anti−pseudomonal	BL−BLI	CP	3GC	4GC	FQ	Mono INH	Macrolide	MDR in S. Typhi and Paratyphi	MDR excluding XDR in TB	Methicillin	PCN	Mono RIF	TMP−SMX	Vanco	XDR in TB
Acinetobacter baumannii	6,160	292		1,810	0.678	2,180	233	0.528	1,640										
Citrobacter spp.	697	22.6		164		28.4	160	99.7	223										
Enterobacter spp.	3,350	426		609		208		1,010	693								400		
Enterococcus faecalis	2,440								2,310									130	
Enterococcus faecium	2,240								1,670									578	
Other enterococci	791								747									43.9	
Escherichia coli	16,700	1,070	814		2,000	741	5,530		2,990								3,560		
Group A Streptococcus	166										166								
Group B Streptococcus	3,040								1,480		1,500				53.7				
Haemophilus influenzae	1,070		803				266												
Klebsiella pneumoniae	26,300	5,010			619	1,090	12,400		3,590								3,580		
Morganella spp.	14.1						0.595	5.51	7.98										
Mycobacterium tuberculosis	6,740									249			6,190			218			86.6
Neisseria gonorrhoeae	15						0.269		14.7										
Proteus spp.	565	51.8	77				156		139								141		
Pseudomonas aeruginosa	8,180	584		719		2,310	3,000	275	1,300										
Salmonella Paratyphi	0.335								0.128			0.207							
Salmonella Typhi	5,600								2,450			3,190							
Non−typhoidal Salmonella	15.4								15.4										
Serratia spp.	1,300	166		309		84.5	284	277	181										
Shigella spp.	206								206										
Staphylococcus aureus	16,000								995		1,380			7,770			5,640	241	
Streptococcus pneumoniae	16,500				98.9	4,930	327		1,460		473				1,030		8,210		
All pathogens	118,000	7,600	1,690	3,610	2,720	11,600	22,400	1,670	22,100	232	3,530	3,070	6,190	7,770	1,090	218	21,500	992	86.6

Counts

- <2.5k
- 2.5k − <5k
- 5k − <10k
- 10k − <25k
- 25k − <75k
- 75k − <150k

Sao Tome and Principe

DALYs (count) *associated with* bacterial antimicrobial resistance by pathogen−drug combinations, 2019

	Resistance to 1+	AG	Aminopenicillin	Anti−pseudomonal	BL−BLI	CP	3GC	4GC	FQ	Mono INH	Macrolide	MDR in S. Typhi and Paratyphi	MDR excluding XDR in TB	Methicillin	PCN	Mono RIF	TMP−SMX	Vanco	XDR in TB
Acinetobacter baumannii	304	185		296	199	213	266	197	251										
Citrobacter spp.	29.8	3.11		10.3		3.42	24.5	17.5	14.6										
Enterobacter spp.	171	54.7		76		27.7		113	83.3								139		
Enterococcus faecalis	98.5								95.3									9.47	
Enterococcus faecium	142								141									36.9	
Other enterococci	61.3								61.2									4.63	
Escherichia coli	730	265	706		583	43	272		284								644		
Group A Streptococcus	20.9										20.9								
Group B Streptococcus	174								74.3		140				2.37				
Haemophilus influenzae	30		21.6				13.2												
Klebsiella pneumoniae	981	626			919	26.9	806		694								897		
Morganella spp.	1.09						0.25	0.22	1										
Mycobacterium tuberculosis	70.4									14.6			55			0.179			0.718
Neisseria gonorrhoeae	2.37						0.004		2.37										
Proteus spp.	64.3	46	54.5				15.6		29.9								48		
Pseudomonas aeruginosa	411	190		201		203	263	133	266										
Salmonella Paratyphi	0.256								0.243			0.014							
Salmonella Typhi	261								53.9			224							
Non−typhoidal Salmonella	0.172								0.172										
Serratia spp.	75	24.8		13.7		5.48	62	44.6	23										
Shigella spp.	1.99								1.99										
Staphylococcus aureus	962								275		578			530			574	12.1	
Streptococcus pneumoniae	869				158	285	116		55.5		267				169		833		
All pathogens	5,460	1,400	782	597	1,860	807	1,840	506	2,410	14.6	1,010	224	55	530	172	0.179	3,140	63	0.718

Counts
- <2.5k
- 2.5k − <5k
- 5k − <10k

Sao Tome and Principe

DALYs (count) *attributable to* bacterial antimicrobial resistance by pathogen−drug combinations, 2019

	Resistance to 1+	AG	Aminopenicillin	Anti−pseudomonal	BL−BLI	CP	3GC	4GC	FQ	Mono INH	Macrolide	MDR in S. Typhi and Paratyphi	MDR excluding XDR in TB	Methicillin	PCN	Mono RIF	TMP−SMX	Vanco	XDR in TB
Acinetobacter baumannii	94.7	7.96		18.4	0.01	34.4	2.01	0.009	31.9										
Citrobacter spp.	8.65	0.145		1.94		0.758	1.83	1.9	2.08										
Enterobacter spp.	38.4	3.1		7.22		5.66		6.49	8.35								7.51		
Enterococcus faecalis	26.4								23.6									2.79	
Enterococcus faecium	35.7								27.2									8.59	
Other enterococci	12.6								11.8									0.858	
Escherichia coli	166	16.2	10.6		29.7	9.16	34.2		26.3								40.1		
Group A Streptococcus	1.95										1.93								
Group B Streptococcus	26.5								12.6		13.3				0.654				
Haemophilus influenzae	7.02		3.17				3.85												
Klebsiella pneumoniae	257	43.7			14.5	8.55	107		42.8								40.4		
Morganella spp.	0.246						0.027	0.036	0.183										
Mycobacterium tuberculosis	32.6									2.08			30.1			0.047			0.418
Neisseria gonorrhoeae	0.231						0.002		0.229										
Proteus spp.	9.3	2.27	0.932				2.87		1.74								1.54		
Pseudomonas aeruginosa	102	5.03		16.6		33	15.9	1.79	29.4										
Salmonella Paratyphi	0.053								0.051			0.002							
Salmonella Typhi	38.7								10.2			29.5							
Non−typhoidal Salmonella	0.033								0.033										
Serratia spp.	19.7	1.36		2.98		1.49	5.34	6.13	2.35										
Shigella spp.	0.365								0.365										
Staphylococcus aureus	219								10.9		21.1			125			58.5	3.3	
Streptococcus pneumoniae	168				3.25	61.3	1.63		6.41		9.94				3.93		81.5		
All pathogens	1,260	79.8	14.6	47.2	47.5	154	175	16.4	248	2.06	46.1	27.9	30.1	125	4.58	0.047	230	15.5	0.418

Counts

<2.5k

Senegal

DALYs (count) *associated with* bacterial antimicrobial resistance by pathogen−drug combinations, 2019

	Resistance to 1+	AG	Aminopenicillin	Anti−pseudomonal	BL−BLI	CP	3GC	4GC	FQ	Mono INH	Macrolide	MDR in S. Typhi and Paratyphi	MDR excluding XDR in TB	Methicillin	PCN	Mono RIF	TMP−SMX	Vanco	XDR in TB
Acinetobacter baumannii	27,000	12,000		26,800	10,000	9,270	15,500	15,300	11,800										
Citrobacter spp.	3,410	1,290		1,510		532	3,110	2,860	1,380										
Enterobacter spp.	19,000	9,630		7,570		3,440		16,200	12,000								15,700		
Enterococcus faecalis	12,300								12,100									808	
Enterococcus faecium	11,200								11,200									2,630	
Other enterococci	5,930								5,920									268	
Escherichia coli	97,000	37,200	92,600		79,300	10,300	43,400		52,300								90,600		
Group A Streptococcus	2,190										2,190								
Group B Streptococcus	32,200								20,700		21,000				652				
Haemophilus influenzae	5,750		4,060				2,800												
Klebsiella pneumoniae	130,000	93,900			123,000	5,260	58,400		98,700								124,000		
Morganella spp.	95						23.5	60.9	68.5										
Mycobacterium tuberculosis	12,400									4,100			7,190			997			96.3
Neisseria gonorrhoeae	117						0.243		117										
Proteus spp.	5,230	1,450	4,540				1,410		2,820								4,120		
Pseudomonas aeruginosa	38,300	20,200		20,600		15,100	21,800	16,400	22,000										
Salmonella Paratyphi	20.9								17.8			3.35							
Salmonella Typhi	4,960								3,790			1,190							
Non−typhoidal Salmonella	61.5								61.5										
Serratia spp.	12,200	6,270		1,570		535	10,400	11,000	3,280										
Shigella spp.	1,250								1,250										
Staphylococcus aureus	91,300								20,500		52,700			33,000			62,700	3,010	
Streptococcus pneumoniae	103,000				18,100	23,900	13,400		9,820		23,700				14,000		99,900		
All pathogens	615,000	182,000	101,000	58,100	231,000	68,300	170,000	61,700	290,000	4,100	99,600	1,200	7,190	33,000	14,600	997	397,000	6,720	96.3

Counts
- <2.5k
- 2.5k − <5k
- 5k − <10k
- 10k − <25k
- 25k − <75k
- 75k − <150k
- >=150k

Senegal

DALYs (count) *attributable to* bacterial antimicrobial resistance by pathogen−drug combinations, 2019

	Resistance to 1+	AG	Aminopenicillin	Anti−pseudomonal	BL−BLI	CP	3GC	4GC	FQ	Mono INH	Macrolide	MDR in S. Typhi and Paratyphi	MDR excluding XDR in TB	Methicillin	PCN	Mono RIF	TMP−SMX	Vanco	XDR in TB
Acinetobacter baumannii	7,670	614		3,920	0.148	1,610	1.11	0.516	1,530										
Citrobacter spp.	1,020	70.6		259		116	99.4	303	170										
Enterobacter spp.	4,600	504		567		659		1,050	1,120								687		
Enterococcus faecalis	3,240								3,020									217	
Enterococcus faecium	2,790								2,170									619	
Other enterococci	1,210								1,160									53.7	
Escherichia coli	23,600	2,180	952		3,160	2,060	4,900		4,880								5,440		
Group A Streptococcus	208										214								
Group B Streptococcus	5,610								3,640		1,830				135				
Haemophilus influenzae	1,370		560				809												
Klebsiella pneumoniae	29,900	6,320			4,550	1,490	6,620		5,730								5,220		
Morganella spp.	22.2						0.889	10.3	11										
Mycobacterium tuberculosis	5,000									577			4,060			267			57.2
Neisseria gonorrhoeae	11.5						0.114		11.4										
Proteus spp.	756	63	74.6				277		188								147		
Pseudomonas aeruginosa	9,100	566		2,300		2,470	972	353	2,440										
Salmonella Paratyphi	4.15								3.71			0.432							
Salmonella Typhi	955								793			167							
Non−typhoidal Salmonella	12								12										
Serratia spp.	3,260	358		327		152	244	1,850	324										
Shigella spp.	256								256										
Staphylococcus aureus	17,800								799		1,980			7,360			6,870	791	
Streptococcus pneumoniae	18,600				489	5,240	493		1,170		846				318		10,000		
All pathogens	137,000	10,700	1,590	7,380	8,210	13,800	14,400	3,570	29,400	620	4,900	161	4,060	7,360	453	267	28,400	1,680	57.2

Counts

- <2.5k
- 2.5k − <5k
- 5k − <10k
- 10k − <25k
- 25k − <75k
- 75k − <150k

Seychelles

DALYs (count) *associated with* bacterial antimicrobial resistance by pathogen−drug combinations, 2019

	Resistance to 1+	AG	Aminopenicillin	Anti−pseudomonal	BL−BLI	CP	3GC	4GC	FQ	Mono INH	Macrolide	MDR in S. Typhi and Paratyphi	MDR excluding XDR in TB	Methicillin	PCN	Mono RIF	TMP−SMX	Vanco	XDR in TB
Acinetobacter baumannii	206	145		189	126	157	151	198	167										
Citrobacter spp.	19	5.15		2.77		3.86	12	14.1	5.84										
Enterobacter spp.	87.3	22.7		28.1		28.9		36.6	29.9								59.6		
Enterococcus faecalis	57.8								55.9									6.18	
Enterococcus faecium	84.8								84.4									14.9	
Other enterococci	40.3								40									14.4	
Escherichia coli	328	43.9	321		234	39.4	129		160								217		
Group A Streptococcus	14.8										14.8								
Group B Streptococcus	28.1								5.96		24.6				1.63				
Haemophilus influenzae	25.2		23.3				6.54												
Klebsiella pneumoniae	223	33.4			175	45.9	170		117								136		
Morganella spp.	0.765						0.419	0.137	0.497										
Mycobacterium tuberculosis	1.33									0.609			0.533			0.095			0.095
Neisseria gonorrhoeae	1.19						0.008		1.19										
Proteus spp.	31.2	5.14	29.9				7.87		9.62								15.8		
Pseudomonas aeruginosa	118	37.3		42.9		83.9	54.7	48.7	50.8										
Salmonella Paratyphi	1.79								1.73			0.063							
Salmonella Typhi	21.8								19			3.07							
Non−typhoidal Salmonella	6.33								6.33										
Serratia spp.	15.8	2.77		2.6		2.31	7.71	9.87	6.45										
Shigella spp.	0.232								0.232										
Staphylococcus aureus	309								127		195			265			24.3	20.2	
Streptococcus pneumoniae	349				58.5	123	119		13.7		201				80.5		269		
All pathogens	1,970	296	374	265	594	485	657	307	902	0.609	436	3.13	0.533	265	82.1	0.095	722	55.7	0.095

Counts
<2.5k

Seychelles

DALYs (count) *attributable to* bacterial antimicrobial resistance by pathogen−drug combinations, 2019

	Resistance to 1+	AG	Aminopenicillin	Anti−pseudomonal	BL−BLI	CP	3GC	4GC	FQ	Mono INH	Macrolide	MDR in S. Typhi and Paratyphi	MDR excluding XDR in TB	Methicillin	PCN	Mono RIF	TMP−SMX	Vanco	XDR in TB
Acinetobacter baumannii	64.1	6.47		7.47	0.022	26.3	0.516	2.51	20.8										
Citrobacter spp.	5.3	0.295		0.499		0.922	0.872	1.93	0.779										
Enterobacter spp.	19.9	1.48		2.49		7.31		1.61	3.26								3.81		
Enterococcus faecalis	15.5								13.8									1.78	
Enterococcus faecium	20.5								16.8									3.61	
Other enterococci	9.26								6.85									2.41	
Escherichia coli	79.2	2.73	7.47		13.5	9.08	16.7		16.6								13.1		
Group A Streptococcus	1.3										1.41								
Group B Streptococcus	3.86								1.03		2.52				0.309				
Haemophilus influenzae	5.33		3.5				1.83												
Klebsiella pneumoniae	61.5	3.28			4.47	12.2	24.6		9.25								7.62		
Morganella spp.	0.192						0.076	0.03	0.086										
Mycobacterium tuberculosis	0.435									0.085			0.274			0.024			0.054
Neisseria gonorrhoeae	0.118						0.002		0.115										
Proteus spp.	3.99	0.258	0.691				1.81		0.661								0.583		
Pseudomonas aeruginosa	28.6	1.04		2.29		16	2.55	1.41	5.34										
Salmonella Paratyphi	0.365								0.356			0.009							
Salmonella Typhi	4.28								3.89			0.4							
Non−typhoidal Salmonella	1.18								1.18										
Serratia spp.	3.81	0.151		0.525		0.632	0.493	1.26	0.746										
Shigella spp.	0.04								0.04										
Staphylococcus aureus	91.2								5.56		7.31			71.5			1.97	4.87	
Streptococcus pneumoniae	71.2				0.382	27.2	7.93		1.55		8.33				1.7		24.4		
All pathogens	491	15.7	11.6	13.3	18.4	99.6	57.3	8.75	109	0.087	19.4	0.4	0.274	71.5	2.01	0.024	51.5	12.7	0.054

Counts
<2.5k

Sierra Leone

DALYs (count) *associated with* bacterial antimicrobial resistance by pathogen−drug combinations, 2019

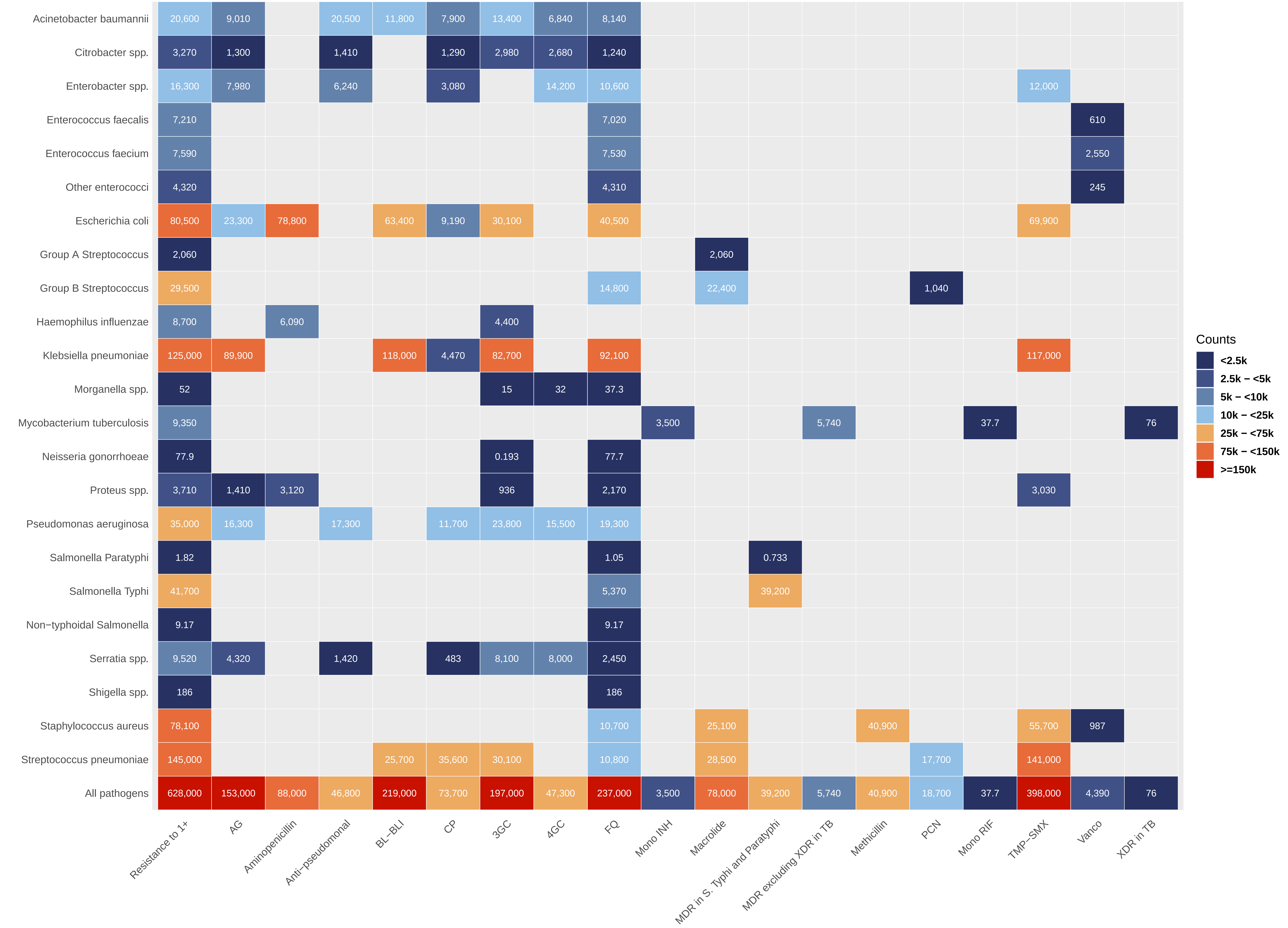

Sierra Leone

DALYs (count) *attributable to* bacterial antimicrobial resistance by pathogen−drug combinations, 2019

	Resistance to 1+	AG	Aminopenicillin	Anti−pseudomonal	BL−BLI	CP	3GC	4GC	FQ	Mono INH	Macrolide	MDR in S. Typhi and Paratyphi	MDR excluding XDR in TB	Methicillin	PCN	Mono RIF	TMP−SMX	Vanco	XDR in TB
Acinetobacter baumannii	5,910	463		3,000	0.168	1,420	1.98	0.456	1,030										
Citrobacter spp.	998	66.7		182		283	103	215	147										
Enterobacter spp.	4,000	414		471		595		988	1,010								522		
Enterococcus faecalis	1,920								1,750									176	
Enterococcus faecium	1,990								1,400									591	
Other enterococci	885								836									49.1	
Escherichia coli	19,200	1,380	1,290		3,100	1,910	3,450		3,900								4,220		
Group A Streptococcus	200										196								
Group B Streptococcus	4,790								2,540		1,990				189				
Haemophilus influenzae	2,090		819				1,270												
Klebsiella pneumoniae	31,100	6,220			3,100	1,320	9,860		5,500								5,100		
Morganella spp.	12.3						0.75	5.5	6.01										
Mycobacterium tuberculosis	3,830									527			3,260			10.1			45.4
Neisseria gonorrhoeae	7.65						0.088		7.56										
Proteus spp.	540	60.2	53.2				178		143								99.7		
Pseudomonas aeruginosa	8,640	450		2,080		1,920	1,570	469	2,150										
Salmonella Paratyphi	0.33								0.226			0.106							
Salmonella Typhi	5,950								902			5,110							
Non−typhoidal Salmonella	1.9								1.9										
Serratia spp.	2,530	246		300		140	317	1,290	243										
Shigella spp.	38.5								38.5										
Staphylococcus aureus	18,000								483		910			10,100			6,270	259	
Streptococcus pneumoniae	27,000				484	7,880	1,660		1,260		1,040				237		14,400		
All pathogens	140,000	9,300	2,160	6,030	6,680	15,500	18,400	2,970	23,300	508	4,250	4,800	3,260	10,100	426	10.1	30,600	1,080	45.4

Counts
- **<2.5k**
- **2.5k − <5k**
- **5k − <10k**
- **10k − <25k**
- **25k − <75k**
- **75k − <150k**

South Africa

DALYs (count) *associated with* bacterial antimicrobial resistance by pathogen−drug combinations, 2019

	Resistance to 1+	AG	Aminopenicillin	Anti−pseudomonal	BL−BLI	CP	3GC	4GC	FQ	Mono INH	Macrolide	MDR in S. Typhi and Paratyphi	MDR excluding XDR in TB	Methicillin	PCN	Mono RIF	TMP−SMX	Vanco	XDR in TB
Acinetobacter baumannii	99,100	52,400		95,300	66,800	74,900	96,700	88,000	91,300										
Citrobacter spp.	4,620	293		1,380		1,870	2,500	1,400	1,540										
Enterobacter spp.	52,100	1,840		6,470		10,400		5,620	5,190								48,300		
Enterococcus faecalis	20,600								19,700									1,880	
Enterococcus faecium	41,000								40,900									2,580	
Other enterococci	22,300								22,300									2,410	
Escherichia coli	187,000	4,860	165,000		92,200	5,320	59,600		72,400								162,000		
Group A Streptococcus	1,890										1,890								
Group B Streptococcus	57,800								5,160		56,100				874				
Haemophilus influenzae	8,360		7,160				2,180												
Klebsiella pneumoniae	260,000	13,600			211,000	49,400	232,000		87,700								232,000		
Morganella spp.	179						17.5	11.5	176										
Mycobacterium tuberculosis	130,000									62,700			46,800			19,700			613
Neisseria gonorrhoeae	981						0.645		979										
Proteus spp.	16,400	207	14,800				3,500		916								12,300		
Pseudomonas aeruginosa	87,400	5,730		37,500		68,500	30,600	37,800	40,600										
Salmonella Paratyphi	21.1								21			0.169							
Salmonella Typhi	16,100								6,120			10,800							
Non−typhoidal Salmonella	630								630										
Serratia spp.	12,500	10,200		1,370		1,820	2,070	1,260	2,880										
Shigella spp.	1,020								1,020										
Staphylococcus aureus	208,000								59,400		153,000			127,000			69,800	2,860	
Streptococcus pneumoniae	267,000				39,700	106,000	57,600		11,300		122,000				133,000		205,000		
All pathogens	1,500,000	89,100	187,000	142,000	410,000	319,000	486,000	134,000	470,000	62,700	333,000	10,800	46,800	127,000	133,000	19,700	730,000	9,740	613

Counts
- <2.5k
- 2.5k − <5k
- 5k − <10k
- 10k − <25k
- 25k − <75k
- 75k − <150k
- >=150k

South Africa

DALYs (count) *attributable to* bacterial antimicrobial resistance by pathogen−drug combinations, 2019

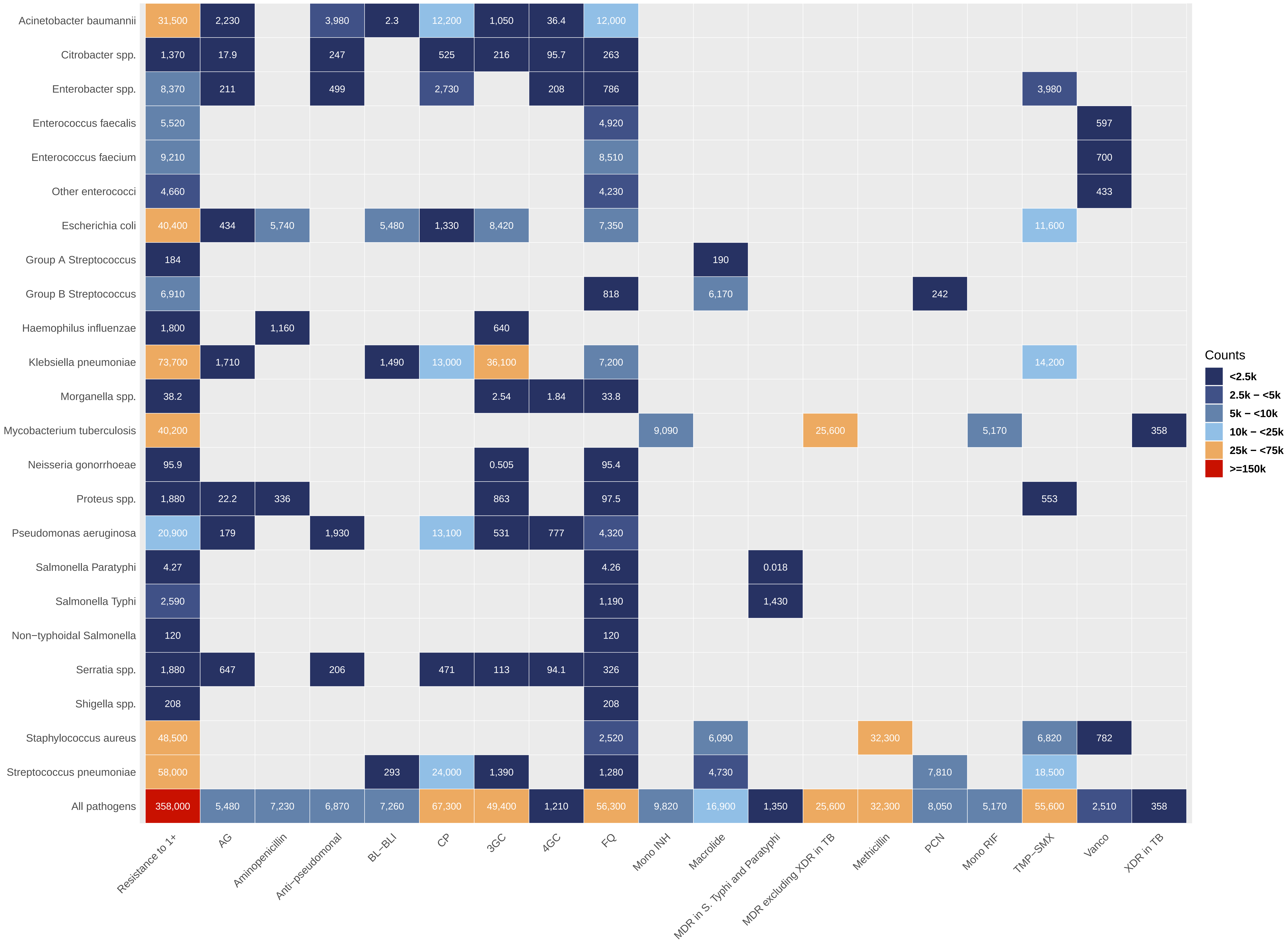

South Sudan

DALYs (count) *associated with* bacterial antimicrobial resistance by pathogen−drug combinations, 2019

	Resistance to 1+	AG	Aminopenicillin	Anti−pseudomonal	BL−BLI	CP	3GC	4GC	FQ	Mono INH	Macrolide	MDR in S. Typhi and Paratyphi	MDR excluding XDR in TB	Methicillin	PCN	Mono RIF	TMP−SMX	Vanco	XDR in TB
Acinetobacter baumannii	31,800	10,300		30,500	21,600	7,950	23,300	13,500	10,400										
Citrobacter spp.	9,100	3,900		3,780		1,790	8,120	7,640	4,970										
Enterobacter spp.	33,300	13,700		12,400		5,710		31,100	18,600								25,100		
Enterococcus faecalis	8,960								8,840									606	
Enterococcus faecium	5,900								5,860									1,290	
Other enterococci	4,700								4,670									367	
Escherichia coli	97,300	25,000	94,000		70,800	11,500	36,700		39,600								80,100		
Group A Streptococcus	3,120										3,120								
Group B Streptococcus	62,900								36,000		47,500				2,540				
Haemophilus influenzae	27,600		26,300				7,490												
Klebsiella pneumoniae	127,000	96,600			123,000	1,440	85,500		76,300								110,000		
Morganella spp.	49.3						12.4	31.7	35.4										
Mycobacterium tuberculosis	20,400									3,000			16,600			660			222
Neisseria gonorrhoeae	89.9						0.919		89.6										
Proteus spp.	3,940	1,980	3,210				879		2,500								3,550		
Pseudomonas aeruginosa	27,400	10,100		9,810		5,950	22,000	14,000	7,180										
Salmonella Paratyphi	1.88								0.146			1.73							
Salmonella Typhi	14,900								7,410			8,090							
Non−typhoidal Salmonella	20.8								20.8										
Serratia spp.	14,400	7,970		4,230		641	10,300	11,700	3,910										
Shigella spp.	9,660								9,660										
Staphylococcus aureus	62,500								14,500		36,600			18,800			47,500	1,250	
Streptococcus pneumoniae	248,000				40,500	67,200	41,300		128,000		35,000				36,500		227,000		
All pathogens	812,000	170,000	124,000	60,800	256,000	102,000	235,000	77,900	378,000	3,000	122,000	8,090	16,600	18,800	39,000	660	493,000	3,510	222

Counts
- <2.5k
- 2.5k − <5k
- 5k − <10k
- 10k − <25k
- 25k − <75k
- 75k − <150k
- >=150k

South Sudan

DALYs (count) *attributable to* bacterial antimicrobial resistance by pathogen−drug combinations, 2019

	Resistance to 1+	AG	Aminopenicillin	Anti−pseudomonal	BL−BLI	CP	3GC	4GC	FQ	Mono INH	Macrolide	MDR in S. Typhi and Paratyphi	MDR excluding XDR in TB	Methicillin	PCN	Mono RIF	TMP−SMX	Vanco	XDR in TB
Acinetobacter baumannii	9,170	579		5,320	31.4	1,560	254	0.786	1,430										
Citrobacter spp.	2,720	184		563		361	235	734	636										
Enterobacter spp.	8,170	710		1,040		1,150		2,510	1,650								1,130		
Enterococcus faecalis	2,360								2,200									161	
Enterococcus faecium	1,460								1,150									311	
Other enterococci	978								907									71.6	
Escherichia coli	22,900	1,530	1,910		3,700	2,530	4,480		3,790								5,000		
Group A Streptococcus	282										288								
Group B Streptococcus	10,700								6,060		4,240				455				
Haemophilus influenzae	5,860		3,810				2,050												
Klebsiella pneumoniae	31,400	6,950			3,610	624	11,000		4,520								4,710		
Morganella spp.	11.5						0.481	5.39	5.67										
Mycobacterium tuberculosis	10,300									437			9,490			181			133
Neisseria gonorrhoeae	8.92						0.258		8.66										
Proteus spp.	574	84.5	52.9				159		160								123		
Pseudomonas aeruginosa	6,980	326		1,500		1,130	2,260	989	782										
Salmonella Paratyphi	0.262								0.034			0.224							
Salmonella Typhi	2,540								1,500			1,000							
Non−typhoidal Salmonella	4.3								4.3										
Serratia spp.	3,630	446		822		195	320	1,450	382										
Shigella spp.	1,980								1,980										
Staphylococcus aureus	11,500								546		1,320			4,050			5,230	331	
Streptococcus pneumoniae	52,100				653	12,700	1,240		16,200		1,150				537		19,500		
All pathogens	186,000	10,800	5,770	9,250	7,990	20,300	22,000	5,700	43,900	472	6,970	996	9,490	4,050	992	181	35,700	874	133

Counts

- **<2.5k**
- **2.5k − <5k**
- **5k − <10k**
- **10k − <25k**
- **25k − <75k**
- **>=150k**

Swaziland

DALYs (count) *associated with* bacterial antimicrobial resistance by pathogen−drug combinations, 2019

	Resistance to 1+	AG	Aminopenicillin	Anti−pseudomonal	BL−BLI	CP	3GC	4GC	FQ	Mono INH	Macrolide	MDR in S. Typhi and Paratyphi	MDR excluding XDR in TB	Methicillin	PCN	Mono RIF	TMP−SMX	Vanco	XDR in TB
Acinetobacter baumannii	2,840	1,060		2,790	1,690	1,630	2,590	2,030	2,070										
Citrobacter spp.	178	20.8		64.4		28.3	115	113	61.6										
Enterobacter spp.	1,200	229		363		221		518	346								1,050		
Enterococcus faecalis	615								598									45.9	
Enterococcus faecium	958								955									171	
Other enterococci	397								396									30.7	
Escherichia coli	6,140	1,650	5,940		3,530	424	2,720		1,700								5,090		
Group A Streptococcus	93.6										93.6								
Group B Streptococcus	1,630								220		1,550				88.5				
Haemophilus influenzae	561		493				153												
Klebsiella pneumoniae	9,180	3,720			8,050	37.8	7,900		4,570								8,410		
Morganella spp.	6.67						0.981	3.16	5.24										
Mycobacterium tuberculosis	8,310									1,390			6,000			840			80.6
Neisseria gonorrhoeae	14.3						0.038		14.2										
Proteus spp.	425	32.1	378				90.5		49.9								325		
Pseudomonas aeruginosa	2,740	859		1,690		1,480	1,590	1,110	1,310										
Salmonella Paratyphi	0.603								0.575			0.034							
Salmonella Typhi	967								378			645							
Non−typhoidal Salmonella	4.43								4.43										
Serratia spp.	416	257		69.4		36.4	132	221	97.1										
Shigella spp.	294								294										
Staphylococcus aureus	7,040								2,000		2,170			1,660			5,720	90.2	
Streptococcus pneumoniae	9,980				2,230	2,850	1,520		1,020		3,470				4,210		9,030		
All pathogens	54,000	7,820	6,810	4,970	15,500	6,700	16,800	3,990	16,100	1,390	7,280	645	6,000	1,660	4,300	840	29,600	338	80.6

Counts
- <2.5k
- 2.5k − <5k
- 5k − <10k
- 10k − <25k
- 25k − <75k

Swaziland

DALYs (count) *attributable to* bacterial antimicrobial resistance by pathogen−drug combinations, 2019

	Resistance to 1+	AG	Aminopenicillin	Anti−pseudomonal	BL−BLI	CP	3GC	4GC	FQ	Mono INH	Macrolide	MDR in S. Typhi and Paratyphi	MDR excluding XDR in TB	Methicillin	PCN	Mono RIF	TMP−SMX	Vanco	XDR in TB
Acinetobacter baumannii	878	46.5		278	0.024	268	14.2	0.089	272										
Citrobacter spp.	50.1	1.12		12.4		6.98	7.68	12.7	9.21										
Enterobacter spp.	236	15.6		34.2		49.8		29.2	37.5								69.3		
Enterococcus faecalis	163								149									14.1	
Enterococcus faecium	231								190									41	
Other enterococci	82.2								76.3									5.91	
Escherichia coli	1,420	104	179		171	95.1	386		158								326		
Group A Streptococcus	8.83										8.42								
Group B Streptococcus	205								34.8		161				16.2				
Haemophilus influenzae	121		76.7				43.9												
Klebsiella pneumoniae	2,390	275			94.1	23.5	1,250		300								441		
Morganella spp.	1.5						0.05	0.532	0.916										
Mycobacterium tuberculosis	3,820									209			3,330			226			48.1
Neisseria gonorrhoeae	1.39						0.019		1.37										
Proteus spp.	50.1	2.01	8.23				21.3		4.29								13.9		
Pseudomonas aeruginosa	691	23.6		177		272	62.1	14.4	141										
Salmonella Paratyphi	0.122								0.118			0.004							
Salmonella Typhi	157								73.8			85.6							
Non−typhoidal Salmonella	0.849								0.849										
Serratia spp.	81.2	15.6		12.9		10.2	5.29	26.7	10.1										
Shigella spp.	60.4								60.4										
Staphylococcus aureus	1,230								84.1		78.6			361			685	26	
Streptococcus pneumoniae	2,020				59	602	43.6		118		122				244		828		
All pathogens	13,900	484	264	514	324	1,330	1,840	83.7	1,720	201	369	82.2	3,330	361	260	226	2,360	86.9	48.1

Counts

<2.5k

2.5k − <5k

10k − <25k

Tanzania

DALYs (count) *associated with* bacterial antimicrobial resistance by pathogen−drug combinations, 2019

	Resistance to 1+	AG	Aminopenicillin	Anti−pseudomonal	BL−BLI	CP	3GC	4GC	FQ	Mono INH	Macrolide	MDR in S. Typhi and Paratyphi	MDR excluding XDR in TB	Methicillin	PCN	Mono RIF	TMP−SMX	Vanco	XDR in TB
Acinetobacter baumannii	110,000	45,000		103,000	86,800	62,200	100,000	54,600	93,100										
Citrobacter spp.	17,100	6,090		7,570		3,480	14,600	9,670	12,100										
Enterobacter spp.	85,800	34,100		37,700		14,200		75,500	47,300								65,000		
Enterococcus faecalis	62,900								62,300									3,750	
Enterococcus faecium	46,300								45,900									12,400	
Other enterococci	26,000								25,900									1,110	
Escherichia coli	376,000	85,400	354,000		292,000	42,700	187,000		142,000								320,000		
Group A Streptococcus	15,000										15,000								
Group B Streptococcus	144,000								77,300		111,000				671				
Haemophilus influenzae	45,000		40,600				12,200												
Klebsiella pneumoniae	587,000	427,000			553,000	4,620	407,000		437,000								551,000		
Morganella spp.	269						60.9	153	211										
Mycobacterium tuberculosis	76,000									7,230			48,900			19,200			653
Neisseria gonorrhoeae	902						27.1		900										
Proteus spp.	23,800	8,690	21,100				4,440		13,100								20,600		
Pseudomonas aeruginosa	172,000	53,100		60,300		49,500	122,000	54,000	112,000										
Salmonella Paratyphi	25.5								1.02			24.3							
Salmonella Typhi	283,000								135,000			238,000							
Non−typhoidal Salmonella	13								13										
Serratia spp.	39,400	20,300		12,900		2,580	24,400	24,100	11,300										
Shigella spp.	644								644										
Staphylococcus aureus	502,000								369,000		252,000			215,000			361,000	4,950	
Streptococcus pneumoniae	621,000				55,300	160,000	80,900		85,600		116,000				234,000		600,000		
All pathogens	3,230,000	680,000	416,000	221,000	987,000	340,000	952,000	218,000	1,670,000	7,230	494,000	238,000	48,900	215,000	235,000	19,200	1,920,000	22,200	653

Counts
- **<2.5k**
- **2.5k − <5k**
- **5k − <10k**
- **10k − <25k**
- **25k − <75k**
- **75k − <150k**
- **>=150k**

Tanzania

DALYs (count) *attributable to* bacterial antimicrobial resistance by pathogen−drug combinations, 2019

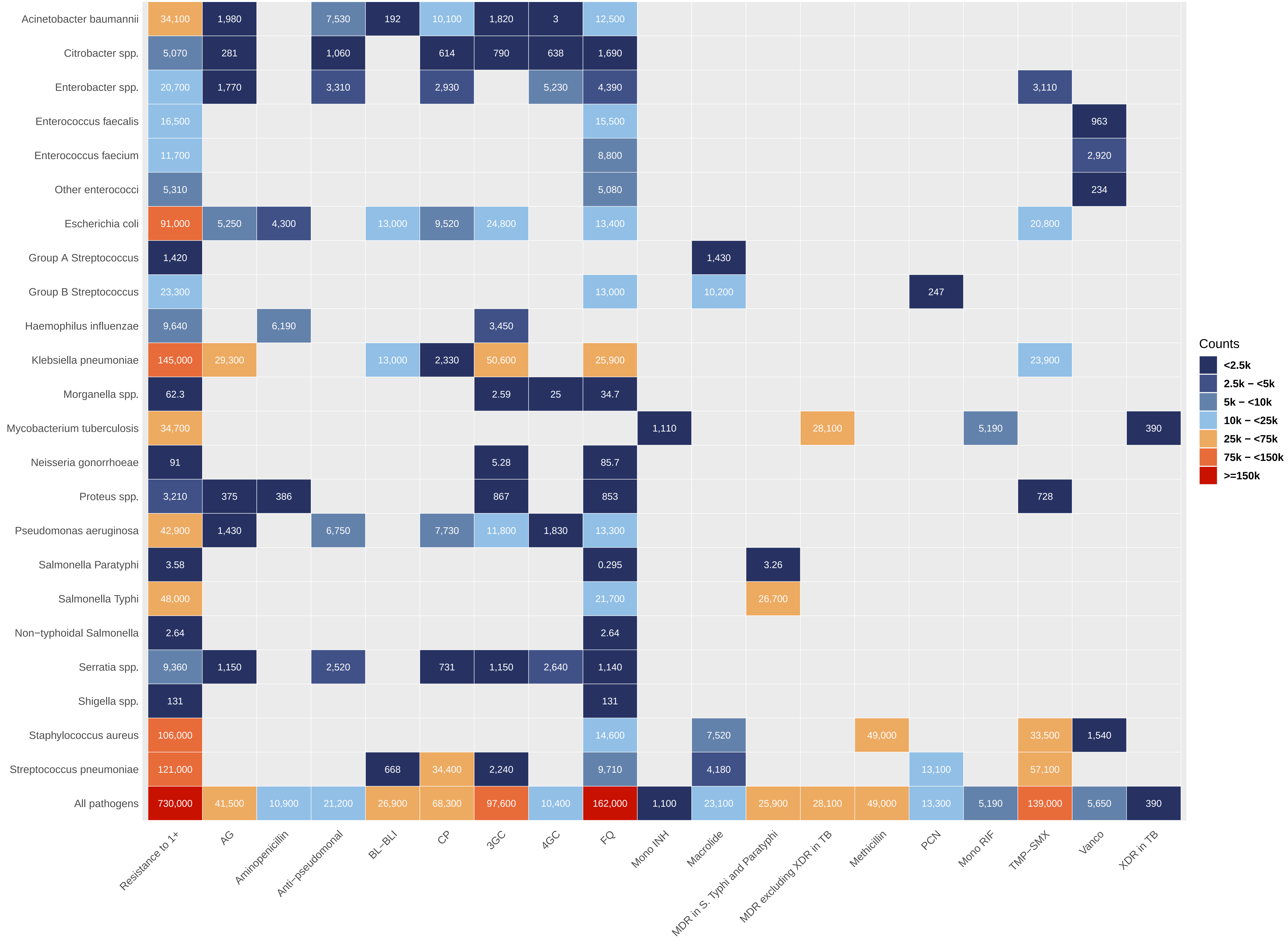

The Gambia

DALYs (count) *associated with* bacterial antimicrobial resistance by pathogen−drug combinations, 2019

	Resistance to 1+	AG	Aminopenicillin	Anti−pseudomonal	BL−BLI	CP	3GC	4GC	FQ	Mono INH	Macrolide	MDR in S. Typhi and Paratyphi	MDR excluding XDR in TB	Methicillin	PCN	Mono RIF	TMP−SMX	Vanco	XDR in TB
Acinetobacter baumannii	4,280	1,930		4,260	2,330	1,610	2,510	1,510	1,760										
Citrobacter spp.	540	219		239		221	501	448	207										
Enterobacter spp.	2,730	1,290		1,060		542		2,350	1,740								2,120		
Enterococcus faecalis	1,530								1,490									132	
Enterococcus faecium	1,770								1,760									426	
Other enterococci	883								881									43.9	
Escherichia coli	12,800	3,380	12,500		9,960	1,290	2,230		4,300								11,100		
Group A Streptococcus	343										343								
Group B Streptococcus	3,810								1,900		2,870				132				
Haemophilus influenzae	642		351				409												
Klebsiella pneumoniae	17,900	11,800			17,700	376	8,630		9,980								13,500		
Morganella spp.	10.7						3.03	6.38	7.83										
Mycobacterium tuberculosis	1,930									432			1,390			85.2			18.5
Neisseria gonorrhoeae	23.6						0.052		23.5										
Proteus spp.	813	315	684				61.1		483								661		
Pseudomonas aeruginosa	5,120	2,420		2,920		1,930	2,940	2,330	2,820										
Salmonella Paratyphi	2.37								2.11			0.278							
Salmonella Typhi	4,110								799			3,440							
Non−typhoidal Salmonella	0.236								0.236										
Serratia spp.	1,870	960		277		84.8	1,590	1,560	491										
Shigella spp.	69.2								69.2										
Staphylococcus aureus	9,300								964		2,140			2,210			7,400	456	
Streptococcus pneumoniae	18,500				2,510	3,520	2,010		889		840				230		18,400		
All pathogens	89,000	22,300	13,600	8,750	32,500	9,570	20,900	8,200	30,600	432	6,190	3,440	1,390	2,210	362	85.2	53,100	1,060	18.5

Counts
- <2.5k
- 2.5k − <5k
- 5k − <10k
- 10k − <25k
- 25k − <75k
- 75k − <150k

The Gambia

DALYs (count) *attributable to* bacterial antimicrobial resistance by pathogen−drug combinations, 2019

	Resistance to 1+	AG	Aminopenicillin	Anti−pseudomonal	BL−BLI	CP	3GC	4GC	FQ	Mono INH	Macrolide	MDR in S. Typhi and Paratyphi	MDR excluding XDR in TB	Methicillin	PCN	Mono RIF	TMP−SMX	Vanco	XDR in TB
Acinetobacter baumannii	1,220	98.5		612	0.032	282	0.185	0.093	223										
Citrobacter spp.	166	11.4		29.9		48.4	16.7	34.9	24.4										
Enterobacter spp.	667	66.3		78.6		105		158	165								93.9		
Enterococcus faecalis	409								370									38.4	
Enterococcus faecium	442								341									100	
Other enterococci	181								172									8.92	
Escherichia coli	2,780	209	263		638	287	249		416								721		
Group A Streptococcus	32.5										32.7								
Group B Streptococcus	617								328		273				24				
Haemophilus influenzae	164		44.3				119												
Klebsiella pneumoniae	4,130	857			877	137	1,080		609								566		
Morganella spp.	2.51						0.156	1.09	1.26										
Mycobacterium tuberculosis	870									63.5			773			22.4			10.9
Neisseria gonorrhoeae	2.3						0.024		2.28										
Proteus spp.	96.7	13.4	15.1				13.7		32.2								23.6		
Pseudomonas aeruginosa	1,240	68.3		356		324	122	53.5	314										
Salmonella Paratyphi	0.466								0.43			0.037							
Salmonella Typhi	584								158			433							
Non−typhoidal Salmonella	0.048								0.048										
Serratia spp.	495	56.5		57.6		24.7	61	249	48.1										
Shigella spp.	13.9								13.9										
Staphylococcus aureus	1,720								41.3		85.7			523			936	136	
Streptococcus pneumoniae	3,060				64.8	802	81.8		103		34.4				2.2		1,980		
All pathogens	18,900	1,380	322	1,130	1,580	2,010	1,750	497	3,360	60.1	419	468	773	523	26.3	22.4	4,320	284	10.9

Counts
- **<2.5k**
- **2.5k − <5k**
- **10k − <25k**

Togo

DALYs (count) *associated with* bacterial antimicrobial resistance by pathogen−drug combinations, 2019

	Resistance to 1+	AG	Aminopenicillin	Anti−pseudomonal	BL−BLI	CP	3GC	4GC	FQ	Mono INH	Macrolide	MDR in S. Typhi and Paratyphi	MDR excluding XDR in TB	Methicillin	PCN	Mono RIF	TMP−SMX	Vanco	XDR in TB
Acinetobacter baumannii	12,900	5,780		12,700	5,140	4,150	7,970	8,380	5,950										
Citrobacter spp.	1,680	577		725		255	1,490	1,370	669										
Enterobacter spp.	10,100	3,940		3,660		1,620		8,080	6,610								8,060		
Enterococcus faecalis	5,600								5,520									364	
Enterococcus faecium	5,430								5,400									1,240	
Other enterococci	2,770								2,760									131	
Escherichia coli	53,200	28,400	51,000		40,700	6,250	25,000		26,600								47,500		
Group A Streptococcus	1,050										1,050								
Group B Streptococcus	16,100								10,000		10,300				257				
Haemophilus influenzae	3,670		2,880				1,500												
Klebsiella pneumoniae	64,700	43,000			60,100	1,600	54,700		50,000								61,700		
Morganella spp.	33.7						7.64	20	25										
Mycobacterium tuberculosis	7,040									2,250			4,730			2.19			63
Neisseria gonorrhoeae	85.4						0.163		85.2										
Proteus spp.	2,360	325	2,090				576		846								1,760		
Pseudomonas aeruginosa	18,700	7,880		10,400		7,820	10,700	7,790	11,200										
Salmonella Paratyphi	2.3								1.56			0.767							
Salmonella Typhi	19,400								5,120			16,500							
Non−typhoidal Salmonella	29.7								29.7										
Serratia spp.	5,340	2,260		589		493	4,290	4,490	1,350										
Shigella spp.	803								803										
Staphylococcus aureus	50,700								9,380		28,500			25,800			34,500	1,770	
Streptococcus pneumoniae	57,900				12,400	13,200	8,330		6,700		13,900				9,040		55,700		
All pathogens	340,000	92,200	56,000	28,100	118,000	35,400	115,000	30,100	149,000	2,250	53,800	16,500	4,730	25,800	9,300	2.19	209,000	3,500	63

Counts
- **<2.5k**
- **2.5k − <5k**
- **5k − <10k**
- **10k − <25k**
- **25k − <75k**
- **75k − <150k**
- **>=150k**

Togo

DALYs (count) *attributable to* bacterial antimicrobial resistance by pathogen−drug combinations, 2019

	Resistance to 1+	AG	Aminopenicillin	Anti−pseudomonal	BL−BLI	CP	3GC	4GC	FQ	Mono INH	Macrolide	MDR in S. Typhi and Paratyphi	MDR excluding XDR in TB	Methicillin	PCN	Mono RIF	TMP−SMX	Vanco	XDR in TB
Acinetobacter baumannii	3,710	301		1,850	0.083	749	15.4	0.397	789										
Citrobacter spp.	497	30.8		127		56.5	53.6	146	83.7										
Enterobacter spp.	2,400	202		282		316		554	666								374		
Enterococcus faecalis	1,470								1,370									98.8	
Enterococcus faecium	1,350								1,050									293	
Other enterococci	563								537									26.3	
Escherichia coli	13,000	1,740	659		1,560	1,230	2,790		2,360								2,700		
Group A Streptococcus	98.9										101								
Group B Streptococcus	2,780								1,780		919				64.4				
Haemophilus influenzae	843		411				432												
Klebsiella pneumoniae	17,100	2,960			704	514	7,120		3,070								2,760		
Morganella spp.	7.78						0.325	3.37	4.08										
Mycobacterium tuberculosis	3,040									345			2,660			0.59			37.4
Neisseria gonorrhoeae	8.32						0.079		8.24										
Proteus spp.	312	16.5	42.3				125		59								70.3		
Pseudomonas aeruginosa	4,570	212		1,170		1,300	478	148	1,250										
Salmonella Paratyphi	0.436								0.332			0.106							
Salmonella Typhi	2,970								917			2,180							
Non−typhoidal Salmonella	5.97								5.97										
Serratia spp.	1,410	126		124		131	173	723	133										
Shigella spp.	166								166										
Staphylococcus aureus	11,400								377		1,020			5,990			3,590	432	
Streptococcus pneumoniae	10,700				361	2,870	356		813		509				237		5,520		
All pathogens	78,500	5,590	1,110	3,550	2,620	7,180	11,500	1,580	15,500	342	2,560	2,030	2,660	5,990	301	0.59	15,000	850	37.4

Counts
- <2.5k
- 2.5k − <5k
- 5k − <10k
- 10k − <25k
- 75k − <150k

Uganda

DALYs (count) *associated with* bacterial antimicrobial resistance by pathogen−drug combinations, 2019

	Resistance to 1+	AG	Aminopenicillin	Anti−pseudomonal	BL−BLI	CP	3GC	4GC	FQ	Mono INH	Macrolide	MDR in S. Typhi and Paratyphi	MDR excluding XDR in TB	Methicillin	PCN	Mono RIF	TMP−SMX	Vanco	XDR in TB
Acinetobacter baumannii	64,300	13,800		63,700	4,370	6,990	16,200	4,750	12,900										
Citrobacter spp.	14,700	3,870		6,430		837	12,600	9,240	8,670										
Enterobacter spp.	67,400	33,100		26,000		7,160		58,600	43,000								50,600		
Enterococcus faecalis	35,100								34,500									2,340	
Enterococcus faecium	30,100								29,800									7,990	
Other enterococci	18,000								17,900									989	
Escherichia coli	251,000	39,700	158,000		167,000	51,800	139,000		132,000								230,000		
Group A Streptococcus	13,900										13,900								
Group B Streptococcus	103,000								37,700		88,700				2,240				
Haemophilus influenzae	35,300		34,100				5,880												
Klebsiella pneumoniae	373,000	266,000			316,000	5,940	316,000		267,000								323,000		
Morganella spp.	147						35.3	87.8	107										
Mycobacterium tuberculosis	52,200									8,070			43,200			315			577
Neisseria gonorrhoeae	579						13.1		576										
Proteus spp.	15,600	3,950	13,100				2,810		6,640								13,700		
Pseudomonas aeruginosa	73,300	11,600		37,400		14,500	62,700	12,800	18,200										
Salmonella Paratyphi	4.14								2.31			1.86							
Salmonella Typhi	147,000								33,100			131,000							
Non−typhoidal Salmonella	112								112										
Serratia spp.	36,800	17,900		14,100		2,200	23,300	26,100	9,200										
Shigella spp.	4,960								4,960										
Staphylococcus aureus	234,000								71,900		136,000			41,600			163,000	2,820	
Streptococcus pneumoniae	353,000				73,400	92,300	125,000		63,700		49,100				25,800		327,000		
All pathogens	1,920,000	390,000	205,000	148,000	560,000	182,000	704,000	112,000	791,000	8,070	288,000	131,000	43,200	41,600	28,000	315	1,110,000	14,100	577

Counts
- <2.5k
- 2.5k − <5k
- 5k − <10k
- 10k − <25k
- 25k − <75k
- 75k − <150k
- >=150k

Uganda

DALYs (count) *attributable to* bacterial antimicrobial resistance by pathogen−drug combinations, 2019

	Resistance to 1+	AG	Aminopenicillin	Anti−pseudomonal	BL−BLI	CP	3GC	4GC	FQ	Mono INH	Macrolide	MDR in S. Typhi and Paratyphi	MDR excluding XDR in TB	Methicillin	PCN	Mono RIF	TMP−SMX	Vanco	XDR in TB
Acinetobacter baumannii	16,400	838		11,700	0.802	1,740	4.11	2.63	2,030										
Citrobacter spp.	4,300	175		1,160		189	703	854	1,210										
Enterobacter spp.	16,000	1,710		2,210		1,450		4,340	4,080								2,180		
Enterococcus faecalis	9,260								8,600									661	
Enterococcus faecium	7,640								5,730									1,910	
Other enterococci	3,710								3,510									203	
Escherichia coli	64,300	2,360	336		4,610	11,400	16,500		13,200								15,900		
Group A Streptococcus	1,360										1,420								
Group B Streptococcus	15,200								6,230		8,600				467				
Haemophilus influenzae	7,110		5,470				1,640												
Klebsiella pneumoniae	98,200	18,900			3,810	2,550	41,800		16,400								14,700		
Morganella spp.	34.1						1.63	15	17.4										
Mycobacterium tuberculosis	26,500									1,190			24,800			85.1			346
Neisseria gonorrhoeae	59						3.07		55.9										
Proteus spp.	2,000	183	249				577		461								531		
Pseudomonas aeruginosa	19,800	358		6,630		2,940	7,670	141	2,090										
Salmonella Paratyphi	0.74								0.49			0.251							
Salmonella Typhi	22,000								5,700			16,300							
Non−typhoidal Salmonella	22.3								22.3										
Serratia spp.	8,950	1,100		2,770		653	797	2,780	915										
Shigella spp.	1,010								1,010										
Staphylococcus aureus	37,100								2,860		5,160			9,860			18,300	879	
Streptococcus pneumoniae	72,400				759	19,900	10,400		7,680		1,830				91.6		31,700		
All pathogens	433,000	25,600	6,050	24,500	9,180	40,900	80,000	8,130	81,800	1,210	16,900	16,000	24,800	9,860	558	85.1	83,300	3,650	346

Counts
- <2.5k
- 2.5k − <5k
- 5k − <10k
- 10k − <25k
- 25k − <75k
- 75k − <150k
- >=150k

Zambia

DALYs (count) *associated with* bacterial antimicrobial resistance by pathogen−drug combinations, 2019

	Resistance to 1+	AG	Aminopenicillin	Anti−pseudomonal	BL−BLI	CP	3GC	4GC	FQ	Mono INH	Macrolide	MDR in S. Typhi and Paratyphi	MDR excluding XDR in TB	Methicillin	PCN	Mono RIF	TMP−SMX	Vanco	XDR in TB
Acinetobacter baumannii	32,000	10,200		30,100	18,300	14,500	29,200	17,700	16,000										
Citrobacter spp.	4,890	965		1,900		268	4,340	2,990	2,950										
Enterobacter spp.	24,000	10,300		10,300		2,690		20,500	14,100								18,600		
Enterococcus faecalis	15,700								15,400									848	
Enterococcus faecium	15,300								15,100									4,310	
Other enterococci	7,010								6,990									351	
Escherichia coli	119,000	49,700	99,400		83,100	41,100	48,200		43,700								106,000		
Group A Streptococcus	4,290										4,290								
Group B Streptococcus	33,500								11,100		29,600				733				
Haemophilus influenzae	12,200		11,700				1,980												
Klebsiella pneumoniae	163,000	146,000			156,000	838	130,000		112,000								159,000		
Morganella spp.	86.5						21.3	47.9	65.7										
Mycobacterium tuberculosis	30,000									4,200			23,500			2,010			314
Neisseria gonorrhoeae	137						1.44		136										
Proteus spp.	7,420	3,040	6,180				1,370		3,120								6,600		
Pseudomonas aeruginosa	42,000	14,800		16,500		14,800	32,000	13,400	19,800										
Salmonella Paratyphi	1.48								0.286			1.15							
Salmonella Typhi	66,700								10,500			63,400							
Non−typhoidal Salmonella	87.1								87.1										
Serratia spp.	12,200	6,300		4,190		600	8,050	7,590	3,070										
Shigella spp.	5,820								5,820										
Staphylococcus aureus	137,000								35,500		57,100			38,200			123,000	1,340	
Streptococcus pneumoniae	153,000				24,200	38,600	21,300		25,100		20,800				47,200		148,000		
All pathogens	886,000	242,000	117,000	63,000	282,000	113,000	277,000	62,200	341,000	4,200	112,000	63,400	23,500	38,200	47,900	2,010	562,000	6,840	314

Counts
- <2.5k
- 2.5k − <5k
- 5k − <10k
- 10k − <25k
- 25k − <75k
- 75k − <150k
- >=150k

Zambia

DALYs (count) *attributable to* bacterial antimicrobial resistance by pathogen−drug combinations, 2019

	Resistance to 1+	AG	Aminopenicillin	Anti−pseudomonal	BL−BLI	CP	3GC	4GC	FQ	Mono INH	Macrolide	MDR in S. Typhi and Paratyphi	MDR excluding XDR in TB	Methicillin	PCN	Mono RIF	TMP−SMX	Vanco	XDR in TB
Acinetobacter baumannii	9,720	495		3,980	2.54	2,590	545	0.776	2,100										
Citrobacter spp.	1,440	43.3		346		60.5	277	297	412										
Enterobacter spp.	5,660	533		936		562		1,430	1,340								859		
Enterococcus faecalis	4,100								3,860									239	
Enterococcus faecium	3,900								2,880									1,020	
Other enterococci	1,440								1,370									72.8	
Escherichia coli	31,400	3,170	934		2,780	9,490	4,250		3,900								6,850		
Group A Streptococcus	402										375								
Group B Streptococcus	4,820								1,810		2,790				152				
Haemophilus influenzae	2,460		1,900				557												
Klebsiella pneumoniae	42,400	10,400			2,230	447	16,200		6,520								6,560		
Morganella spp.	20.1						1.12	8.07	10.9										
Mycobacterium tuberculosis	14,600									633			13,300			542			187
Neisseria gonorrhoeae	13.5						0.411		13.1										
Proteus spp.	975	137	104				272		208								244		
Pseudomonas aeruginosa	10,700	400		1,880		2,610	3,180	395	2,240										
Salmonella Paratyphi	0.23								0.075			0.151							
Salmonella Typhi	9,350								1,660			7,890							
Non−typhoidal Salmonella	17.4								17.4										
Serratia spp.	2,940	370		847		182	388	842	308										
Shigella spp.	1,190								1,190										
Staphylococcus aureus	25,200								1,300		1,870			7,650			14,000	404	
Streptococcus pneumoniae	29,700				550	8,280	688		2,900		739				2,190		14,300		
All pathogens	203,000	15,600	2,950	7,980	5,560	24,200	26,300	2,970	34,000	581	5,900	7,910	13,300	7,650	2,340	542	42,800	1,740	187

Counts
- <2.5k
- 2.5k − <5k
- 5k − <10k
- 10k − <25k
- 25k − <75k
- >=150k

Zimbabwe

DALYs (count) *associated with* bacterial antimicrobial resistance by pathogen−drug combinations, 2019

	Resistance to 1+	AG	Aminopenicillin	Anti−pseudomonal	BL−BLI	CP	3GC	4GC	FQ	Mono INH	Macrolide	MDR in S. Typhi and Paratyphi	MDR excluding XDR in TB	Methicillin	PCN	Mono RIF	TMP−SMX	Vanco	XDR in TB
Acinetobacter baumannii	35,600	11,800		33,200	19,800	16,800	32,700	24,400	22,700										
Citrobacter spp.	2,670	374		1,230		405	1,920	1,640	917										
Enterobacter spp.	19,800	4,940		5,720		3,670		11,000	6,670								16,800		
Enterococcus faecalis	9,790								9,560									756	
Enterococcus faecium	11,300								11,200									1,990	
Other enterococci	4,840								4,820									405	
Escherichia coli	105,000	15,200	98,000		61,900	9,050	54,700		41,600								91,900		
Group A Streptococcus	1,880										1,880								
Group B Streptococcus	38,300								8,140		35,200				1,940				
Haemophilus influenzae	17,400		16,700				3,210												
Klebsiella pneumoniae	157,000	66,800			138,000	1,840	135,000		79,500								140,000		
Morganella spp.	67.1						9.78	37.5	50.2										
Mycobacterium tuberculosis	65,800									18,800			46,000			251			621
Neisseria gonorrhoeae	167						0.144		166										
Proteus spp.	5,340	705	4,770				1,150		620								4,100		
Pseudomonas aeruginosa	43,700	9,390		30,500		21,200	26,700	18,700	18,600										
Salmonella Paratyphi	8.48								8.21			0.351							
Salmonella Typhi	23,000								6,160			18,500							
Non−typhoidal Salmonella	127								127										
Serratia spp.	6,370	3,870		1,040		642	2,000	3,720	1,380										
Shigella spp.	609								609										
Staphylococcus aureus	105,000								25,900		49,900			26,600			74,500	1,380	
Streptococcus pneumoniae	172,000				51,000	51,100	26,000		19,700		57,000				68,400		157,000		
All pathogens	826,000	113,000	120,000	71,700	271,000	105,000	283,000	59,600	258,000	18,800	144,000	18,500	46,000	26,600	70,300	251	485,000	4,540	621

Counts
- <2.5k
- 2.5k − <5k
- 5k − <10k
- 10k − <25k
- 25k − <75k
- 75k − <150k
- >=150k

Zimbabwe

DALYs (count) *attributable to* bacterial antimicrobial resistance by pathogen−drug combinations, 2019

	Resistance to 1+	AG	Aminopenicillin	Anti−pseudomonal	BL−BLI	CP	3GC	4GC	FQ	Mono INH	Macrolide	MDR in S. Typhi and Paratyphi	MDR excluding XDR in TB	Methicillin	PCN	Mono RIF	TMP−SMX	Vanco	XDR in TB
Acinetobacter baumannii	10,900	541		3,830	2.36	2,830	683	5.09	3,000										
Citrobacter spp.	764	18.9		241		97.4	112	163	131										
Enterobacter spp.	4,120	314		513		800		754	688								1,090		
Enterococcus faecalis	2,600								2,380									218	
Enterococcus faecium	2,710								2,230									478	
Other enterococci	1,010								929									77.6	
Escherichia coli	25,400	946	2,190		2,550	2,010	7,680		3,970								6,070		
Group A Streptococcus	172										170								
Group B Streptococcus	5,210								1,320		3,550				361				
Haemophilus influenzae	3,560		2,660				898												
Klebsiella pneumoniae	41,100	4,930			1,750	807	21,200		5,200								7,190		
Morganella spp.	15.1						0.335	6.31	8.45										
Mycobacterium tuberculosis	29,800									2,920			26,400			69.3			374
Neisseria gonorrhoeae	16.2						0.111		16.1										
Proteus spp.	639	39.4	98.2				268		52.3								176		
Pseudomonas aeruginosa	11,200	255		3,770		4,000	977	201	2,020										
Salmonella Paratyphi	1.75								1.7			0.04							
Salmonella Typhi	3,560								1,160			2,460							
Non−typhoidal Salmonella	24.7								24.7										
Serratia spp.	1,270	229		190		176	71.1	452	140										
Shigella spp.	123								123										
Staphylococcus aureus	18,100								1,080		1,950			5,940			8,750	407	
Streptococcus pneumoniae	35,100				1,570	10,800	661		2,250		2,070				3,480		14,300		
All pathogens	197,000	7,280	4,950	8,550	5,880	21,500	32,600	1,580	26,700	2,910	7,660	2,340	26,400	5,940	3,840	69.3	37,600	1,180	374

Counts
- **<2.5k**
- **2.5k − <5k**
- **5k − <10k**
- **10k − <25k**
- **25k − <75k**
- **>=150k**
